# Aging-regulated PNUTS maintains endothelial barrier function via SEMA3B suppression

**DOI:** 10.1038/s42003-024-06230-5

**Published:** 2024-05-07

**Authors:** Noelia Lozano-Vidal, Laura Stanicek, Diewertje I. Bink, Rio P. Juni, Aukie Hooglugt, Veerle Kremer, Philippa Phelp, Anke van Bergen, Alyson W. MacInnes, Stefanie Dimmeler, Reinier A. Boon

**Affiliations:** 1 Department of Physiology, Amsterdam UMC, VU University, De Boelelaan 1117, 1081 HV Amsterdam, the Netherlands; 2 Amsterdam Cardiovascular Sciences, Microcirculation, De Boelelaan 1117, 1081 HV Amsterdam, The Netherlands; 3 Institute of Cardiovascular Regeneration, Goethe University, Theodor-Stern-Kai 7, 60590 Frankfurt am Main, Germany; 4 Department of Medical Biochemistry, Amsterdam UMC, University of Amsterdam, Meibergdreef 9, 1105 AZ Amsterdam, The Netherlands; 5 Laboratory Genetic Metabolic Diseases, Amsterdam UMC, University of Amsterdam, Meibergdreef 9, 1105 AZ Amsterdam, the Netherlands; 6 German Center for Cardiovascular Research (DZHK), Partner Site Rhein-Main, Potsdamer Strasse 58, 10785 Berlin, Germany

**Keywords:** Cardiovascular biology, Ageing

## Abstract

Age-related diseases pose great challenges to health care systems worldwide. During aging, endothelial senescence increases the risk for cardiovascular disease. Recently, it was described that Phosphatase 1 Nuclear Targeting Subunit (PNUTS) has a central role in cardiomyocyte aging and homeostasis. Here, we determine the role of PNUTS in endothelial cell aging. We confirm that PNUTS is repressed in senescent endothelial cells (ECs). Moreover, PNUTS silencing elicits several of the hallmarks of endothelial aging: senescence, reduced angiogenesis and loss of barrier function. Findings are validate in vivo using endothelial-specific inducible PNUTS-deficient mice (Cdh5-CreERT2;PNUTS^fl/fl^), termed PNUTS^EC-KO^. Two weeks after PNUTS deletion, PNUTS^EC-KO^ mice present severe multiorgan failure and vascular leakage. Transcriptomic analysis of PNUTS-silenced HUVECs and lungs of PNUTS^EC-KO^ mice reveal that the PNUTS-PP1 axis tightly regulates the expression of semaphorin 3B (SEMA3B). Indeed, silencing of SEMA3B completely restores barrier function after PNUTS loss-of-function. These results reveal a pivotal role for PNUTS in endothelial homeostasis through a SEMA3B downstream pathway that provides a potential target against the effects of aging in ECs.

## Abstract (web summary)

PNUTS-deficient mice exhibit multiorgan failure and vascular leakage. PNUTS regulates SEMA3B, suggesting a target for endothelial aging.

## Introduction

Cellular aging is characterized by hallmarks such as epigenetic changes, genomic instability, mitochondrial stress and cellular senescence that promote a certain number of processes leading to physiological dysfunction and multiple pathologies^1^. The progressive aging of the population in Western countries has led to an increase of age-related diseases, a new health care challenge^2^. Of those, cardiovascular diseases (CVD) have the highest prevalence in the elderly, both in women and men. Therefore, aging is the principal risk factor for cardiovascular diseases, which is also reflected in for instance the Framingham risk score, in which the relative contribution of age is at least three times higher than all other risk factors^3,4^.

The vascular endothelium serves as a barrier between the blood and the surrounding tissues, and has other critical functions for homeostasis, such as maintaining vascular tone and controlling platelet activity, leukocyte adhesion and angiogenesis^5^. Aged endothelial cells present lower proliferative and migration capacity, higher sensitivity to apoptotic signals and cellular senescence^6–8^. As a result, endothelial aging is correlated with impaired angiogenesis, reduced capillary density, increased vascular permeability and a pro-thrombotic phenotype in vascular beds, all leading to CVD^9,10^.

Vascular permeability is controlled by different mechanisms involving intrinsic and extrinsic factors. Among these, there are changes in the composition of intercellular adherens and tight junctions^11,12^, the contractile function of the actin-myosin cytoskeleton^13^, paracrine signaling^14^ and variations in calcium transients^15^. An important regulator of vascular integrity is the family of axon guidance proteins, specifically the semaphorin subfamily. Initially identified as determinants of axon growth and polarization, semaphorins are shown now to have an important role in regulating vascular patterning and endothelial function^16–18^.

Phosphatase 1 Nuclear Targeting Subunit (PNUTS, *PPP1R10*) is a nuclear protein described to act as a binding platform for the PP1 phosphatase complex^19^. PNUTS is implicated in cell cycle, cancer cell proliferation and protection against DNA damage response and apoptosis^20^. Recently, we showed that PNUTS expression is markedly repressed in the heart during aging and CVD, and the mechanism by which PNUTS downregulation induces post-ischemic heart failure includes telomere attrition in cardiomyocytes, a hallmark of aging^21^. PNUTS protein contains multiple binding domains, serving as partner for several targets, as PP1, TRF2, PTEN and nucleic acids, which determine PNUTS function in different contexts^22–24^. The PNUTS-PP1 complex is formed by interaction through the canonical RxVF motif. By binding to PP1, PNUTS is implicated in the dephosphorylation and activity of several PP1 targets, such as RNA polymerase II^25^, Myc^26^, Rb^27^ and Spt5^28^. miR-34a, a regulator of PNUTS expression^21^, has been linked to endothelial permeability and tight junctions^29,30^. However, whether PNUTS plays a role in endothelial aging or barrier function is not known.

Here, we show that PNUTS is an aging-regulated gene in the endothelium, and that PNUTS depletion mimics an aging endothelial phenotype. Also, we describe an endothelial-specific PNUTS knockout animal model which presents severe endothelial dysfunction similar to PNUTS depletion in cultured human endothelial cells. Particularly, endothelial PNUTS loss triggers a dramatic increase in endothelial permeability, but has no effect on adherens junction composition. RNA-sequencing shows how PNUTS loss leads to a dysregulation of gene expression, particularly in axon guidance genes. Finally, using the PP1-binding mutant PNUTS^W401A^, we show that aberrant SEMA3B expression is the main mediator of the loss of barrier function after PNUTS depletion in a PP1-dependent manner.

## Methods

### Cell culture and cellular assays

Human umbilical vein endothelial cells (HUVEC) were purchased from Lonza and cultured in endothelial cell medium containing supplements and 5% FBS (ECM; ScienceCell). Cells were cultured at 37 °C with 5% CO_2_. HUVECs were transfected with control or PNUTS siRNA for 48 h (except for sprouting assays, see below). For proliferation assays, cells were incubated with 10 μM EdU for 4 h using the Click-iT EdU microplate Kit (Invitrogen) according to the manufacturer’s protocol. Apoptosis was assessed by incubating the cells with 200 nM Staurosporin or medium for 4 h and the caspase 3/7 activity was assayed using the ApoOne Caspase 3/7 Assay (Promega). PP1 was inhibited by stimulation with Tautomycetin (166 nM, R&D Systems). Senescence associated β-Galactosidase activity was analyzed with the Senescence Associated β-Galactosidase Staining Kit (Cell Signaling Technologies). Images were taken with a bright-field microscope (Axio Observer Z1.0 microscope, Zeiss) and the number of total cells, as well as the number of stained cells was determined in 4 images for per condition and experiment.

### Transfection and lentiviral overexpression

HUVECs were transfected with siRNAs (10nmol/l) with lipofectamine RNAiMax (Life Technologies, Carlsbad, CA), as described before^31^. The siRNAs used were siPNUTS (Sigma-Aldrich Hs01_00133264), siSEMA3B (Dharmacon, J-007754-09-0005) and siMYC (Dharmacon L-003282-02-0005), siRNA Universal Control 1 (Sigma-Aldrich SIC001) was used as control. Lentiviral overexpression of PNUTS was achieved by cloning the full-length human cDNA of PNUTS into pLenti4/v5-DEST (Life technologies). The PP1 non-binding mutant PNUTS (W401A) was kindly provided by Dr. Thomas Küntziger^20^ and cloned into pLenti4/v5. All these PNUTS sequences were modified by directed mutagenesis to introduce 3 silent mutations in the seed sequence of the siRNA against PNUTS (see Table 1 for primers). pLenti-CMV-MYC was provided by Dr. Linda Penn^26^. Lentiviral particles were generated as described earlier^32^.

**Table 1:** Primers used in this study

Use	Gene	Forward primer (5ʹ-3ʹ)	Reverse primer (5ʹ-3ʹ)
qPCR	Human PNUTS	GGGTTCGGGTCCCATAGACCCC	GCCGCCAACGTCAATAAATTTGAC
	Human p21	AGTCAGTTCCTTGTGGAGCC	CATTAGCGCATCACAGTCGC
	Human RPSA	ATAAGGACGTCATTTCCTGCC	TTTAAGTTACGACGGGAATCCAGAA
	Human RPLP0	TCGACAATGGCAGCATCTAC	ATCCGTCTCCACAGACAAGG
	Human SEMA3B	TGTGCTCCGGAGACTCGT	CGCTGGAAAGTCCACTCCAC
	Mouse Pnuts	TGGGCAGTGGACATCGTTCTCA	GGCGGTTTGACATGTCTCCTCCA
	Mouse Sema3b	CACCTCCAGTGGTGTCTTCC	AAAGGTCCCAAGAAGGCTCG
	Mouse Rplp0	GCGTCCTGGCATTGTCTGT	GAAGGCCTTGACCTTTTCAGTAA
Mutagenesis	PNUTS	TGGATGAAACAGAGCGGGTAAATGTGA	TCACATTTACCCGCTCCGTTTCATCCA
Genotyping	Cre	GCGGTCTGGCAGTAAAAACTATC	GTGAAACAGCATTGCTGTCACTT
	Cpxm1	ACTGGGATCTTCGAACTCTTTGGAC	GATGTTGGGGCACTGCTCATTCACC
	PNUTSfl	CATGACAATATGGAGGAGAAAGTGCCTTG	AGGAGAAAGAAGAGAACAGGGGACCATAAT

### Sprouting angiogenesis and immunofluorescence

Endothelial angiogenesis was studied by spheroid sprouting assay. HUVECs were first transfected with the indicated siRNAs for 24 h. Then, cells were trypsinized and added to a mixture of culture medium and methylcellulose (80%:20%) and transferred to a 96-well plate to allow the formation of spheroids for 24 h at 37 °C. The spheroids were collected, added to methylcellulose with FBS (80%:20%) and embedded in a collagen type I (BD Biosciences) gel. Following incubation for 24 h at 37 °C with 50 ng/ml VEGF the gels were fixed with formaldehyde and microscope images were taken at 10x magnification (Axio Observer Z1.0 microscope, Zeiss). The cumulative length of sprouts was quantified using image analysis software (AxioVision 4.8, Zeiss). For immunofluorescence (IF), 48 h after transfection cells were fixed with PFA 4% and incubated with anti-CD31 (1:40, BD Biosciences 550389) or anti-VE-Cadherin (1:400, CST 2500XP), and actin fibers were stained with Acti-stain 555 (Cytoskeleton Inc). Images were obtained in a Nikon A1R confocal microscope. Endothelial gaps were measured by quantifying the intercellular area versus the total area in 4 fields per image, 3 images per experiment.

### Endothelial cell impedance and transwell assays

After lentiviral transduction and/or transfection, HUVECs were plated in 96-well arrays (96W10idf, Applied Biophysics) at 40,000 cells/well and endothelial cell impedance was measured using the multi-frequency mode for 48 h (ECIS Zθ, Applied Biophysics). Endothelial multifrequency resistance was modeled for the calculation of cell-cell adhesion (R_b_). Transwell assay was performed as explained elsewhere^33^.

### Flow cytometry

Cell surface expression of VE-Cadherin and PECAM1 was analyzed in HUVECs by flow cytometry. Briefly, transfected HUVECs were detached with Accutase and washed in cold incubation buffer (0.1% BSA in PBS). Cells were blocked (5% BSA in PBS) for 30 min on ice and subsequently incubated with fluorophore-labeled antibodies for 30 min on ice. Protein cell surface expression was analyzed using a FACSCalibur™ device (BD Biosciences).

### qRT-PCR and RNA Sequencing

Total RNA from cultured cells was isolated with Direct-zol RNA miniprep (Zymogen) and from tissues using miRNeasy Mini kit (Qiagen) according to the manufacturer’s protocols. Intima RNA was isolated by flushing 200 μl of Trizol through the lumen of isolated aortas. Total RNA was reverse transcribed using random hexamer primers and iScript cDNA synthesis kit (BioRad). qPCR was performed with SYBR Green Master Mix on CFX384 Real Time PCR Detection System (BioRad), using primers listed in Table 1. Gene expression analysis was done using the 2^-ΔCT^ method. RNA obtained from HUVECs and mouse lung tissue was sequenced by Exiqon (whole transcriptome sequencing, 50 bp, 30 M reads) and data analysis was performed using DAVID functional annotation tools and Gene Set Enrichment Analysis.

### Western blot

Total cell lysates were collected 48 h after HUVEC transfection or 5 days after lentiviral treatment and western blot (WB) carried as described before^34^. The primary antibodies used were anti-PNUTS (R&D Systems, AF21581), anti-CD31 (Invitrogen, 37-0700), anti-VE-Cadherin (Sigma-Aldrich, V1514), anti-MYC (CST, 9402), anti-phospho-MYC-T58 (abcam, ab185655), anti-tubulin (Thermo Fisher, RB-9281-P) and anti-β-actin (Sigma-Aldrich, A-5441). Uncropped Western blots are depicted in Supplementary Fig. S7.

### Polysome profiling

siControl and siPNUTS-transfected cells were trypsinized, pelleted and snap-frozen. Polysome profiling was performed as previously described^35^. Briefly, cell pellets were lysed in polysome lysis buffer (gradient buffer containing 100 mM KCl, 10 mM MgCl2, 0.1% NP-40, 2 mM DTT, and 40 U/ml RNasin; Promega, Leiden, Netherlands), and onto 17–50% sucrose gradients and ultracentrifuged for 2 h at 40,000 rpm. The gradients were displaced into a UA6 absorbance reader and absorbance was recorded at an OD of 254 nm.

### ELISA

HUVECs were transfected with siRNA in a 6 well plate as described above. The transfection mix was replaced with 1 ml full medium 4 h post transfection. Supernatants were collected 72 h post transfection and spun down (1000 *×* *g*, 20 min at 4 °C) to eliminate cell debris. In order to quantify SEMA3B levels, 300 µl supernatant was applied to a human SEMA3B ELISA plate (DLdevelop, Shuigoutou, China), the SEMA3B protein standard was diluted in full medium. The following procedure was performed according to the manufacturer’s instructions.

### Animal procedures

All mice experiments were carried out in accordance with the principles of laboratory animal care as well as according to the German and Dutch national laws. The studies have been approved by the local ethical committees and performed in accordance with the ethical standards laid down in the Declaration of Helsinki. PNUTS^flox/flox^ mice were generated by inserting LoxP sequences, flanking *Ppp1r10* exons 9 to 14, at 69 bp downstream of exon 8 and 139 bp upstream exon 15 (Genoway, France). PNUTS^flox/flox^ mice were crossed with the Cdh5-CreERT2 line to obtain the endothelial-specific inducible PNUTS knockout strain^36^. Genotyping was performed using primers from Table 1. Cdh5-CreERT2x*Pnuts*^flox/flox^ (PNUTS^EC-KO^) and *Pnuts*^flox/flox^ (WT) littermates were injected intraperitoneally with 2 mg/day tamoxifen dissolved in peanut oil (Sigma-Aldrich) for 5 consecutive days and on the 8th and 10th day after the first injection, and then sacrificed for organ harvest at day 15^th^. All mice were 2–12 months old at the start of the experiments and the total number of mice used in this study is 15 mice per group. Aortic rings were obtained as described^37^ and incubated for 48 h with 10 ng/μl VEGF. After fixation, images were taken at 5x magnification and stitched with a Zeiss Axiovert 100 and the endothelial sprouts length were quantified with an image analysis software (AxioVision 4.8, Zeiss). The Evans Blue Extravasation assay was performed on day 14th adapting the protocol by Radu et al. ^38^ Briefly, PNUTS^EC-KO^ and WT mice were injected in the tail vein with 25 mg/Kg of Evans Blue solution and observed for 1 h before euthanasia. Then, organs were harvested and incubated for 48 h in formamide to extract the extravasated dye, which was quantified in a spectrophotometer against a standard curve of known concentrations of Evans Blue.

### Histology

Organs were fixed in formalin and embedded in paraffin. 4 μm sections were obtained and stained with Periodic-Acid Schiff (PAS, Sigma-Aldrich, 395B) and hematoxylin-eosin. Images were obtained in a Leica microscope. Kidney sections were antigen retrieved with citrate buffer and immunostained with an anti-CD31 antibody (1:20, Dianova, DIA-310). Images were taken in a Nikon A1R confocal microscope. Assessment of the capillary, mesangial and glomerular areas was performed on PAS-staining kidney sections using ImageJ software. The capillary surface area was defined as the surface area within the glomerulus tuft covered by capillary loops. The mesangial surface area was defined as a PAS–positive and nuclei-free area in the mesangium. Both quantifications were based on 8 glomeruli per group.

### Statistics

GraphPad 7 (GraphPad Software) was used for statistical analyses. Comparison of two different conditions was analyzed by two-tailed Student’s *t* test or Mann–Whitney, multiple comparisons were performed by one-way ANOVA using Dunnett’s, Bonferroni or Tukey’s correction. Data are expressed as means ± SEM, *p*  <  0.05 was considered as statistically significant (*p  <  0.05; **p  <  0.01; ***p  <  0.001). The sample size n states the number of independent experiments, unless denoted otherwise. All results were reproduced in at least three technically independent replicates.

### Reporting summary

Further information on research design is available in the Nature Portfolio Reporting Summary linked to this article.

## Results

### PNUTS KD induces partial senescence of endothelial cells in vitro

**Fig. 1:**
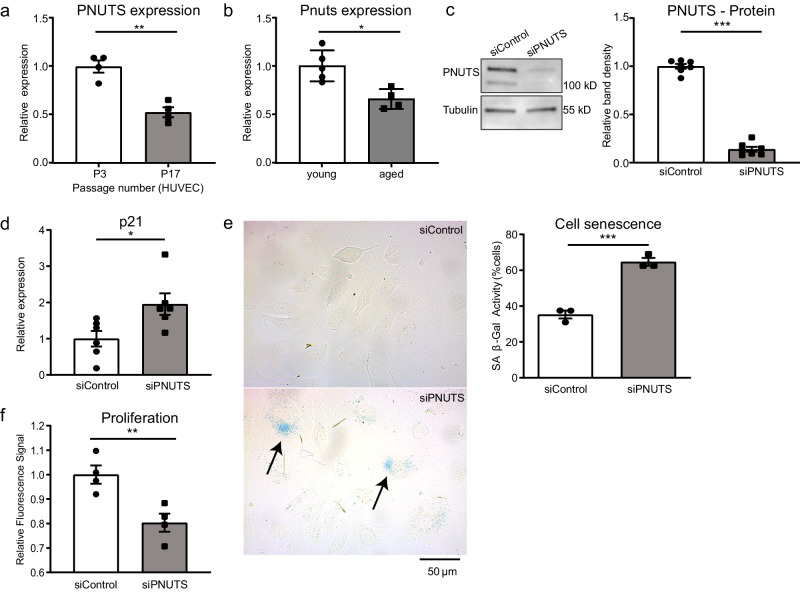
PNUTS is an aging-regulated protein and regulates senescence in endothelial cells. **a** HUVECs were cultured for 3 or 17 passages (P) and PNUTS expression was measured by qRT-PCR (*n* = 4). **b** Aortic intima was isolated from young (8–12 weeks old) and aged (18–20 months old) mice and immediately lysed for RNA isolation. Intima RNA was sequenced. **c**–**f** HUVECs were transfected with siRNAs targeting PNUTS or a control sequence. **c** PNUTS protein levels were determined by WB at 48 h after transfection, relative to alpha-tubulin. Densitometric quantification is depicted on the right (*n* = 7). **d** Changes in p21 expression were assessed by qRT-PCR. Expression values are relative to siControl-treated HUVECs and normalized to RPSA mRNA (*n* = 6). **e** The percentage of senescent HUVECs was analyzed by staining for senescence-associated β-Galactosidase (SA-(β-Gal) 72 h after transfection. Images were taken (4 fields per sample) and the percentage of senescent cells (blue) over the total number of cells in each field was calculated in *n* = 3 independent experiments, 2–3 biological replicates per group and experiment). **f** Cell proliferation was assayed by testing the incorporation of EdU after transfection (*n* = 4). *p < 0.05, **p < 0.01, ***p < 0.001. Error bars depict the standard error of the mean (SEM).

The first question we sought to answer was whether PNUTS is repressed during aging in the endothelium, as we had described previously for the myocardium, where PNUTS regulates telomere length through TRF2, one of its binding partners^21^. Therefore, we used a model of serial passaging of HUVECs^39,40^. PNUTS expression was repressed by 50% in this cell culture-induced aging model (Fig. 1a). Furthermore, we assessed Pnuts mRNA expression in the aortic intima of young (8—12 weeks old) and aged mice (18–20 months old), showing a marked reduction of Pnuts expression in the aortic endothelium (Fig. 1b). Consequently, we hypothesized that PNUTS may have a role in maintaining endothelial homeostasis which is lost during aging. For this purpose, we used an siRNA-based knockdown (KD) strategy in HUVECs. We selected an siRNA against PNUTS which caused a decrease of PNUTS protein levels of nearly 90% (Fig. 1c). Loss of PNUTS provoked an increase in the expression of p21, p16^INK4A^, β-galactosidase activity and several senescence-associated secretory protein genes (SASP), but not all SASP genes we measured (Fig. 1d, e, S1b). Next to senescence, aging can be accompanied by an increase in apoptosis and a decrease in proliferation. Accordingly, PNUTS KD significantly decreased proliferation (Fig. 1f), while apoptosis was not affected (Fig S1a). However, PNUTS KD increased cell susceptibility to a pro-apoptotic stimulus (Fig S1a), one of the hallmarks of endothelial aging^8^. These results indicate a role for PNUTS in partial inhibition of endothelial cell senescence and aging.

### PNUTS is necessary for endothelial cell sprouting in vitro and in vivo

**Fig. 2:**
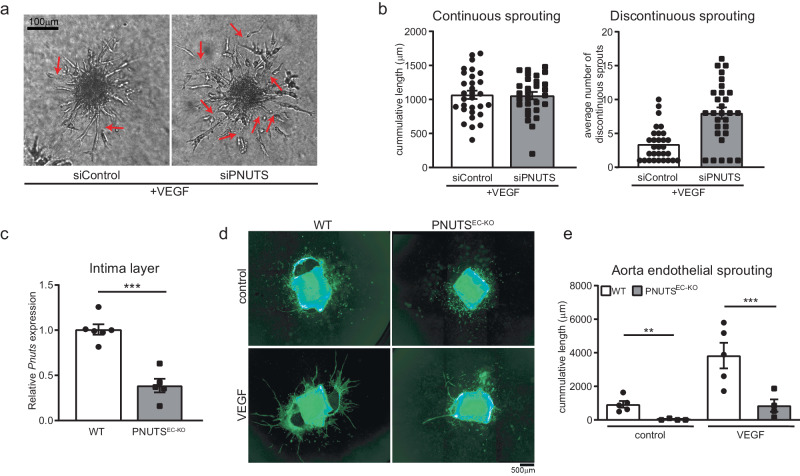
PNUTS is necessary for normal angiogenic sprouting. **a** In vitro sprouting was analyzed under VEGF (50 ng/ml) stimulation. Representative images are shown, red arrows indicate discontinuous sprouts. **b** Quantification of cumulative sprout length (left) and number of discontinuous sprouts (right) (*n* = 30 spheroids per group in 3 independent experiments were measured). **c** Endothelial *Pnuts* expression of PNUTS^EC-KO^ and WT mice was assessed by RT-qPCR in intima samples 15 days after initiating tamoxifen treatment and normalized to *Rplp0* mRNA (*n* = 3). **d** In vivo angiogenesis was tested by aortic ring assay in WT and PNUTS^EC-KO^ mice in basal conditions or upon 48 h of VEGF (10 ng/ml) stimulation. **e** Quantification of aortic ring sprouting. *n* = 4–5 mice per group. *p < 0.05, **p < 0.01, ***p < 0.001. Error bars depict the standard error of the mean (SEM).

As angiogenesis is typically reduced during aging^10^, we performed a spheroid sprouting assay to check the angiogenic capability of PNUTS KD HUVECs. The results indicate that the total length of sprouts, from the surface of the spheroid to the tip cell, was similar in control and KD cells, but in the latter the sprouts showed an aberrant pattern characterized by numerous discontinuities (Fig. 2a, b). These results suggest that loss of PNUTS induces loss of stalk cell function or cell-cell adhesions.

To assess the physiological role of PNUTS in ECs we sought to use an in vivo model of PNUTS loss. To this moment, no loss-of-function mouse model had been described for PNUTS, therefore we generated an endothelial-specific inducible PNUTS knockout mouse line, Cdh5-CreERT2x*Pnuts*^flox/flox^ (PNUTS^EC-KO^), which undergoes efficient loss of PNUTS in the endothelial layer two weeks after tamoxifen treatment compared to PNUTS^fl/fl^ (WT) (Fig. 2c, Fig S2a, b). Using this mouse model, we assessed the angiogenic capability of ECs lacking PNUTS. PNUTS^EC-KO^ aortas were subjected to an aortic ring assay, which showed that the sprouting of aortic rings in PNUTS^EC-KO^ mice was abrogated compared to WT (Fig. 2d, e), confirming the findings obtained in vitro and suggesting a role for PNUTS in maintaining endothelial cell function.

### PNUTS EC-knockout mice present multiorgan failure and vascular leakage

**Fig. 3:**
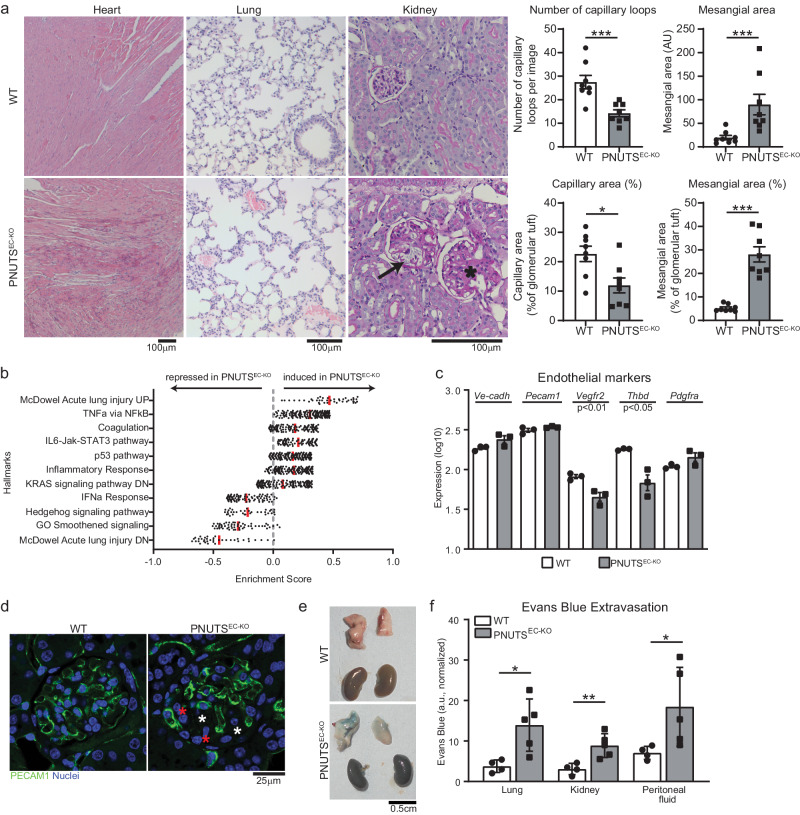
Induction of endothelial-specific PNUTS KO in mice provokes vascular leakage and multiorgan failure. **a** Histopathological study of different tissues in PNUTS^EC-KO^ mice compared to WT mice: left, heart samples (haematoxylin-eosin) presenting edema; middle, lung samples (haematoxylin-eosin) presenting thrombi; right, kidney samples (PAS staining) showing glomerulosclerosis (asterisk) and capillary dilatation (black arrow) in renal glomeruli. Quantifications of the kidney sections show the absolute and relative contribution of capillaries and mesangial tissue in glomeruli (*n* = 8 per group). **b** RNA-seq was performed with lung tissue of WT and PNUTS^EC-KO^ mice and was analysed for differentially regulated pathways using Gene Set Enrichment Analysis. Enrichment scores of the indicated pathways are plotted on the *x* axis (*n* = 3). **c** The expression levels of the endothelial markers *Ve-cadherin*, *Pecam1*, *Vegfr2*, *Thbd* and *Pdgfra* in WT and PNUTS^EC-KO^ lung samples were confirmed by RT-qPCR and normalized to *Rplp0* mRNA (*n* = 3). **d** The presence of ECs in the glomerular capillary network was investigated by PECAM1 immunostaining in kidneys of WT and PNUTS^EC-KO^ mice. Red and white asterisks indicate potential mesangial proliferation and glomerulosclerotic areas, respectively. **e**, **f** An Evans Blue (EB) extravasation assay was performed to measure the vascular extravasation in different organs. **e** Representative lung and kidney images 1 h after intravenous administration of EB at 25 mg/kg. **f** Colorimetric measurement of extravasated EB into lung, kidney and peritoneal fluid (*n* = 4–5 mice per group). *p < 0.05, **p < 0.01, ***p < 0.001. Error bars depict the standard error of the mean (SEM).

We carefully monitored PNUTS^EC-KO^ mice after initiation of PNUTS excision by tamoxifen treatment to assess the physiological role of endothelial PNUTS. Within 15 days after tamoxifen treatment, all PNUTS^EC-KO^ mice died, accompanied by a severe accumulation of fluids in peritoneal and pleural cavities. Pathological analysis of kidneys, lungs and hearts showed that PNUTS^EC-KO^ mice suffered from glomerulosclerosis, capillary dilation and pulmonary and cardiac edema (Fig. 3a). Image analysis of PAS-stained kidney sections showed that PNUTS^EC-KO^ mice present a marked decrease in the number and area of capillary loops as well as an expansion of the mesangial area and fraction in their renal glomeruli (Fig. 3a). As our findings pointed that the loss of PNUTS induced a major change in the phenotypical characteristics of ECs, we assessed global gene expression by RNA sequencing (RNA-seq) in lungs of PNUTS^EC-KO^ and WT mice (Fig. 3b, Supplementary data 2). The RNA-seq data showed an increase in inflammatory response and coagulation pathways and changes in genes related to endothelial cell-cell and cell-matrix interaction. Intriguingly, while the endothelial cell markers *Pecam1* and *VE-Cadherin* were unaltered, angiogenesis- and coagulation-related genes *Vegfr2* and *Thbd* showed a slight but significant decreased expression, indicating a potential decrease in endothelial cell function rather than a loss of endothelial cells (Fig. 3c). *p21* and several SASP genes were upregulated, suggesting a role for PNUTS in endothelial senescence also in vivo (Fig S2c). Presence of ECs in PNUTS^EC-KO^ kidneys was confirmed by Immunofluorescence of PECAM1 in renal glomeruli and confirmed the loss of capillary organization in glomeruli, as observed in Fig. 3a. (Fig. 3d). Intriguingly, the phenotype observed in several organs, including the presence of fluid in the cavities, pointed to increased microvascular permeability. Therefore, we investigated whether PNUTS^EC-KO^ mice suffered from vascular leakage. We performed Evans Blue extravasation assays and found that the tracer was able to cross the endothelial barrier to the tissue in several organs in PNUTS^EC-KO^ mice, as well as the peritoneal cavity, pointing to a loss of barrier integrity in vivo (Fig. 3e, f). These results suggest that endothelial PNUTS is critical for maintenance of endothelial barrier and its loss is incompatible with life due to severe vascular leakage.

### PNUTS loss compromises the endothelial barrier by disrupting cell-cell interaction

**Fig. 4:**
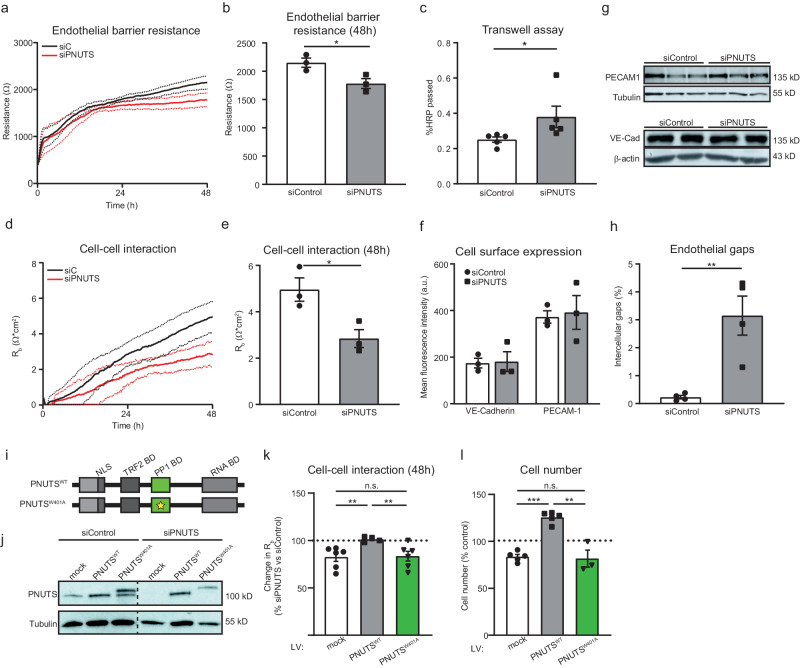
PNUTS is necessary for endothelial barrier function independently of cell junction gene expression. **a**–**h** HUVECs were treated with siRNA (si) targeting PNUTS or a control sequence. a-b) HUVEC barrier resistance was assessed by ECIS at 4000 Hz for 48 h, presented as average resistance ± SEM of *n* = 3 experiments, 8 biological replicates per group and experiment. **c** HUVECs were seeded in transwells and HRP passage through the endothelial monolayer was assessed by absorption measurements (450 nm) and shown as percentage of total HRP (*n*  =  5). **d**, **e** Cell-cell interaction was assessed by modeling of data obtained by ECIS measured as R_b_ (Ω*cm^2^); presented as average resistance ± SEM of *n* = 3 experiments, 8 biological replicates per group and experiment. **f** Cell surface presence of PECAM1 and VE-cadherin was assessed by flow cytometry 48 h after siRNA transfection (*n* = 3). **g** Total PECAM1 and VE-cadherin levels were assessed by Western Blotting (WB). Tubulin and β-actin expression was used as loading control. **h** Cells were grown into confluence and immuno-stained for VE-cadherin, PECAM1 and F-actin. DAPI was used to stain nuclei. The presence of intercellular gaps in endothelial monolayers was measured by quantifying the intercellular areas versus the total area in 4 fields per image, 3 images per experiment, *n* = 4. **i** Schematic representation of the PNUTS lentiviral vectors used for the barrier rescue experiments. Both vectors included silent mutations in the seed sequence of siPNUTS. HUVECs were transduced with the indicated constructs for 8–10 days and later transfected with siControl or siPNUTS to silence endogenous expression of PNUTS, before subjecting them to ECIS and cell counting (4–6 independent experiments, 4 biological replicates per group and experiment). **j** PNUTS expression in total cell lysates of HUVECs treated with indicated vectors and siRNAs was analyzed by WB, Tubulin was used as a loading control. Representative WB is shown. **k** Change in cell-cell interaction measurement from ECIS 48 h after cell seeding, measured as variation of R_b_ of siPNUTS- vs siControl-treated cells. **l** Cell proliferation of ECIS-assayed HUVECs, measured as % number of cells relative to control. *p < 0.05, **p < 0.01, ***p < 0.001. Error bars depict the standard error of the mean (SEM).

The endothelial monolayer is responsible for regulating the trafficking of solutes and cells from the blood to the surrounding tissues, forming a selective barrier between both. In light of our in vivo results, we confirmed whether PNUTS is necessary for this function. We measured the impedance of HUVEC monolayers using an electrical cell impedance system (ECIS) and found that loss of PNUTS decreased the resistance of the endothelial monolayer (Fig. 4a, b), suggesting an increase in endothelial permeability. As a confirmation of these results, we performed a transwell assay, which showed an increased leakage through the monolayer of PNUTS-silenced cells, as measured by passage of HRP (Fig. 4c). This decrease in endothelial barrier function is mainly due to a loss of endothelial cell-cell interaction, as indicated by multifrequency modeling of R_b_ (Fig. 4d, e). Western blot and flow cytometry measurements of HUVECs showed that the effects of PNUTS depletion on endothelial barrier function are not due to decreased levels or delocalization of adherens junction proteins (Fig. 4f, g). However, IF analysis of VE-Cadherin and PECAM1 in PNUTS-silenced HUVECs reveal the presence of junctional defects and an increase of intercellular gaps that might explain the inability to form an efficient barrier in the absence of PNUTS (Fig. 4h, S3). Altogether, this suggests that PNUTS is necessary to maintain a functional endothelial monolayer through a mechanism independent of adherens junction protein expression.

Previous studies describe PNUTS as a binding partner and activator for PP1^22^. We therefore aimed to assess the potential role of PP1 for PNUTS function by using several lentiviral vectors to overexpress siRNA-resistant wild-type (PNUTS^WT^) and PP1-non-binding mutant PNUTS (PNUTS^W401a)^ (Fig. 4i). All of them proved to be resistant to siPNUTS treatment (Fig. 4j) and were used to test the relevance of the PP1-binding domain for the role of PNUTS in barrier maintenance by ECIS. As seen in Fig. 4k, only the overexpression of PNUTS^WT^ was able to rescue the drop in barrier function caused by PNUTS KD, while the PP1-binding mutant was not. Moreover, the PP1-binding is important for endothelial survival, as the overexpression of the PNUTS^W401A^ mutant decreased EC proliferation to the same levels as siPNUTS treatment, while PNUTS^WT^ had a positive effect on proliferation of ECs (Fig. 4l).

### PNUTS loss triggers a complex transcriptional profile change in endothelial cells

In order to more elaborately study the mechanism by which PNUTS controls EC function, we studied the gene profile elicited by PNUTS loss. We performed RNA-seq of siControl and siPNUTS-treated HUVECs. We found 7034 transcripts significantly up- or downregulated (Supplementary data 3). KEGG pathway functional annotation identified several affected pathways, among which mRNAs encoding ribosomal proteins stood out (Fig S4a). Sixty-eight ribosomal protein-encoding genes were significantly altered, of which 62 were strongly downregulated (Fig S4b). In line with this finding, a polysome profiling of siControl- and siPNUTS-treated HUVECs showed that PNUTS KD provokes a reduction of ribosomal content in ECs (Fig S4c).

Gene set enrichment analysis showed a decrease in expression of Myc-dependent targets (Fig S5). MYC has been described to have roles in endothelial proliferation and angiogenesis^41^ and it has been recently suggested that MYC phosphorylation and subsequent degradation might be controlled by the PNUTS^26^. MYC protein levels were decreased in PNUTS KD cells, while phosphorylation of MYC at Threonine 58 was increased, which induces ubiquitination and proteasomal degradation of MYC (Fig S5c). However, siRNA-mediated KD of MYC had no effect on endothelial barrier function, while lentiviral-mediated overexpression of MYC had a negative effect, opposite to expected (Fig S5d). Therefore, MYC is a negative regulator of the endothelial barrier in a PNUTS-independent manner.

### PNUTS regulates barrier function via suppression of SEMA3B expression

**Fig. 5:**
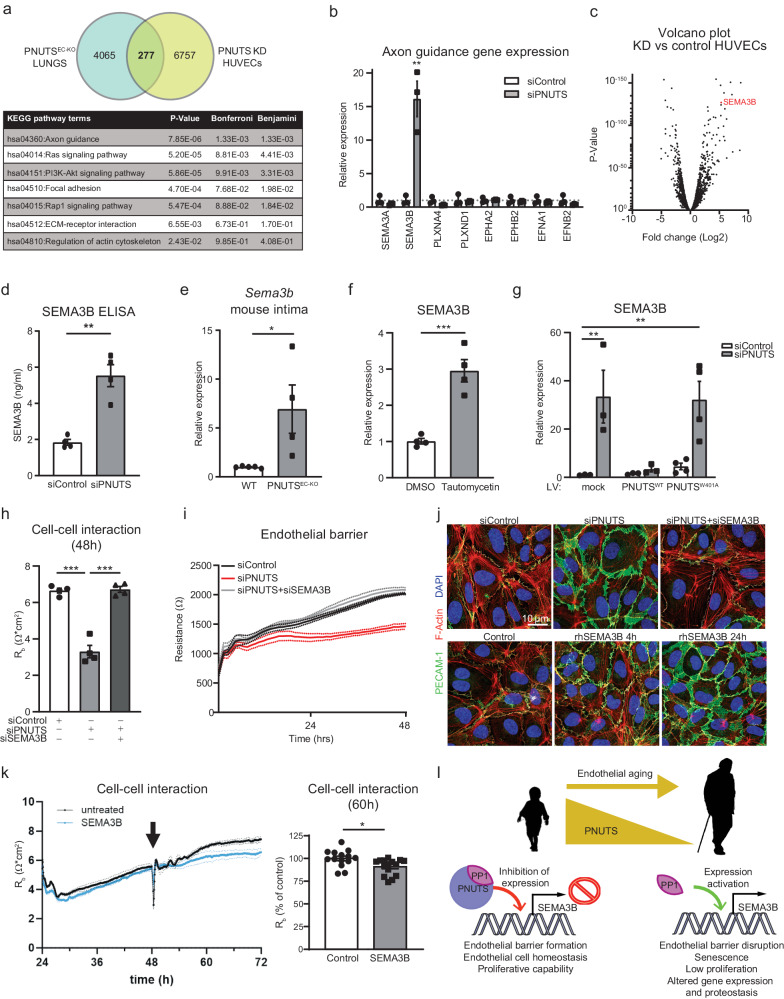
PNUTS KD induces transcriptomic changes in HUVECs. **a** The two subsets of RNAseq data we obtained, from endothelial-depleted PNUTS mouse lungs and PNUTS knockdown (KD) HUVECs, were compared to find common targets of PNUTS depletion. The Venn diagram depicts the finding of 277 common transcripts, which were functionally analysed using KEGG pathways analysis, shown in the table below. **b** Changes in axon guidance gene expression after PNUTS silencing was assessed by RT-qPCR. Expression values are relative siControl-treated HUVECs and normalized to RPSA mRNA (*n* = 6). **c** Volcano plot showing the distribution of gene expression in PNUTS KD versus control HUVECs. SEMA3B is marked in red. **d** Supernatant SEMA3B concentration was determined by ELISA 72 h after PNUTS silencing (*n* = 4). **e** Expression levels of *Sema3b* mRNA in intima samples of PNUTS^EC-KO^ mice was assessed by RT-qPCR, relative to WT samples and normalized to *Rplp0* mRNA. **f** HUVECs were treated with vehicle or Tautomycetin (166 nM) for 48 h and mRNA was analyzed by RT-qPCR for expression of SEMA3B, normalized to *RPSA* mRNA (*n* = 3). **g** Expression of SEMA3B was measured in mRNA samples of HUVECs assayed in Fig. 4j–l, relative to control cells and normalized to *RPSA* mRNA. **h**-**i** HUVECs were co-transfected with siPNUTS and/or siSEMA3B were subjected to ECIS for 48 h (*n* = 4 independent experiments, 4 biological replicates per group and experiment). **h** Cell-cell interaction was modeled. **i** Endothelial resistance was measured at 4000 Hz. **j** Top panel: siSEMA3B rescues the effect of PNUTS silencing on adherence junctions (shown as PECAM1 IF staining). Bottom panel: PECAM1 IF staining shows time course of change in adherens junctions upon stimulation with recombinant human SEMA3B. **k** Cell-cell interaction was modeled using ECIS. The arrow indicates the 48 h time point at which recombinant SEMA3B was added to the medium (or not; untreated). Quantification was performed at 60 h (*n* = 4 biological replicates and 3–4 technical replicates). **l** Graphic summary of the proposed mechanism. In young individuals, PNUTS interacts and promotes activity of PP1, which represses the expression of SEMA3B. Endothelial cells are in homeostasis and maintain their barrier function. During aging, PNUTS is repressed in endothelial cells. The absence of PNUTS inhibits PP1 function at the SEMA3B promoter activating SEMA3B expression. SEMA3B exerts repulsive signals between endothelial cells, promoting intercellular gaps and disrupting the barrier. This provokes a series of critical changes in the cells leading to cellular senescence. *p < 0.05, **p < 0.01, ***p < 0.001. Error bars depict the standard error of the mean (SEM).

The complexity of the data obtained by RNA-seq led us to compare the gene expression profile after PNUTS loss in HUVECs and mouse lungs. A total of 277 common genes were found to be regulated in both datasets. KEGG pathway analysis of these revealed a significant presence of axon guidance pathway genes (Fig. 5a). We confirmed the RNA-seq data by qPCR in PNUTS-silenced HUVECs, finding a dramatic induction of SEMA3B expression (Fig. 5b), one of the highest upregulated genes after PNUTS KD (Fig. 5c, Supplementary data 3). SEMA3B protein was also found to be increased in the supernatant of siPNUTS-treated cells (Fig. 5d), as quantified by ELISA. Furthermore, the expression of *Sema3b* in the aortic intima of PNUTS^EC-KO^ mice is increased 7-fold compared to WT mice (Fig. 5e). SEMA3B has been described previously as a secreted protein that causes repulsion signals in ECs which interferes with correct angiogenesis^42^, but has not yet been linked to endothelial barrier formation.

PP1 has been described to exert a broad role in regulating gene expression, thus we studied SEMA3B expression after treating HUVECs with the PP1 inhibitor drug Tautomycetin a highly specific inhibitor of PP1. Tautomycetin blocks PP1 enzymatic activity by binding PP1 Cys127, in a higher selective manner compared to other PP inhibitors as okadaic acid or TF-23A^43,44^. Indeed, PP1 inhibition triggered a significant induction of SEMA3B expression (Fig. 5f). Furthermore, the induction of SEMA3B expression after PNUTS silencing is rescued by overexpression of PNUTS^WT^, but not by overexpression of the PP1-binding mutant PNUTS^W401A^ (Fig. 5g). Finally, we hypothesized that SEMA3B might be the causal mechanism triggered by loss of PNUTS leading to a disruption of barrier function. To test this, we co-treated HUVECs with siPNUTS and siSEMA3B and found that SEMA3B knockdown completely abolished the effect of PNUTS loss on cell-cell interaction and endothelial resistance (Fig. 5h-i) and rescued the effect of siPNUTS on adherens junctions (Fig. 5j, top panel). To further test the effect of this semaphorin on cell-cell interactions, we cultured HUVECs with medium containing recombinant human SEMA3B and found that SEMA3B induces changes in PECAM1 membrane pattern similar to those of siPNUTS (Fig. 5j, bottom panel) and that SEMA3B reduces cell-cell interactions, as measured by ECIS (Fig. 5k). Altogether, these results point to the axon repulsion signal SEMA3B as a disruptor of endothelial monolayer integrity which is repressed by PNUTS in homeostatic situations through PP1 binding and activity (Fig. 5l).

Of note, the previously described regulator of PNUTS, miR-34a, has been shown to promote endothelial permeability through targeting tight junction-related protein kinase Cε^29,30^. Since miR-34a plays a role in cardiac aging and disease by post-transcriptional regulation of PNUTS^21^, we hypothesized that miR-34a could be upstream of PNUTS and its role in endothelial permeability as well. Transfection of miR-34a in HUVECs provoked a decrease in barrier function measured by ECIS. However, overexpressing PNUTS (using a construct that does not contain a miR-34a binding site) in these cells was not able to rescue the barrier (Fig S6a). Moreover, overexpression of miR-34a only causes a mild downregulation of PNUTS in HUVECs, and not a significant increase of SEMA3B expression (Fig S6b). These results suggest that the role of PNUTS on endothelial cell aging and function is independent of miR-34a.

## Discussion

This study describes the aging-regulated protein PNUTS as a critical element to maintain endothelial homeostasis, as loss of endothelial PNUTS elicits a phenotype of endothelial leakage compromising homeostasis. PNUTS, through its PP1-binding domain, is necessary for endothelial barrier integrity. Loss of PNUTS deregulates the signature gene expression profile in ECs and triggers the induction of SEMA3B, a member of the axon guidance family of proteins that acts as a repulsion signal for ECs.

PNUTS structure and function have been mainly studied in vitro in cell lines, while the role of PNUTS in physiology and disease has been largely unknown. In particular, the role of PNUTS in the cardiovascular system or aging has been described previously only in the myocardium, where PNUTS regulates telomere length through TRF2 in cardiomyocytes and is downregulated during aging or after myocardial infarction^21^. In the present work, we sought to assess the role of PNUTS in the endothelium. As seen in cardiomyocytes, endothelial PNUTS is aging-regulated and critical to maintain cell functionality in ECs. In fact, PNUTS loss recapitulates many of the hallmarks of endothelial aging, such as expression of senescence markers, decreased proliferation, gene expression deregulation and endothelial dysfunction (Figs. 1, 2, 4 and S1). However, PNUTS loss in the myocardium provoked an increase in DNA damage and apoptosis through the aforementioned mechanism, whilst in the endothelium PNUTS has a role in maintaining endothelial integrity via regulation of SEMA3B expression. This suggests that PNUTS function and mechanism of action is cell type- and binding partner-specific. PNUTS was initially described as a binding platform for PP1, and although several other binding partners of PNUTS have been described over time^45^, the main body of knowledge on PNUTS function continues to be related to this phosphatase. The PNUTS-PP1 axis has been suggested to be responsible for DNA damage response^20^, MYC activation^26^, cell cycle entry^46^, apoptosis^27^, gene expression regulation^25^ and transcription^28^. None of these studies report, however, a role for PNUTS-PP1 in physiological processes, with the exception of the work of Ciurciu and colleagues^25^, which modeled their findings in the embryonic development of *D. melanogaster*. Our work shows the contribution of PNUTS in a physiological context in mammals.

However, vascular permeability is controlled by complex mechanisms, for instance by haemodynamic changes and lymphatic drainage. These mechanisms are difficult to separate in vivo and cannot be well addressed in an in vitro setting. This is an important limitation of our study. Another limitation is that the assays we use to study endothelial permeability do not distinguish between transcellular and paracellular leakage. Even though the data suggest that SEMA3B-mediated repulsion results in paracellular leakage after reduction of PNUTS, a contribution of transcellular leakage cannot be excluded.

The decline of PNUTS protein levels with ageing has been shown before in the heart^21^, where miR-34a induction results in reduction of PNUTS levels. Our experiments show that miR-34a can also target PNUTS in ECs, but that this reduction is relatively mild (Fig S6). It is therefore likely that other mechanisms contribute to the decline of PNUTS levels with ageing. One potential mechanism could be the recently identified alternative splicing regulation of PNUTS that results in a non-coding transcript^47^, in which an increase of the non-coding RNA would inherently results in a decrease of the PNUTS mRNA. One of the functions of ECs is to form a barrier between the blood and the underlying tissues that allows correct exchange of nutrients and waste products. This important function is also impaired by aging, which induces hyperpermeability in the peripheral tissues and eventually edema formation due to an increase in transcellular and paracellular permeability^48^. Notably, our results in PNUTS^EC-KO^ mice and PNUTS KD HUVECs point to endothelial barrier dysfunction independent of adherens junctions (Figs. 3a, e, f and 4a–g), which we concluded was the main consequence of PNUTS loss in the endothelium. Interestingly, even though PNUTS silencing induces gaps in between cells, these events are rather rare, and the increase in permeability seems to be restricted to smaller molecules, suggesting that dynamic repulsive signals may cause the barrier disruption. These effects are mediated by via the PP1 binding domain of PNUTS (Fig. 4h–l). Previously, PP1 had been described to have a role in endothelial permeability by dephosphorylation of cytoskeleton proteins^49^, but here we provide evidence that PP1 function in ECs is linked to a nuclear co-factor (PNUTS).

Hence, we considered a nuclear mechanism that provoked cells to be less prone to form proper interactions. PNUTS loss elicits a complex response in gene expression both in vivo (Fig. 3b) and in vitro (Fig S4a, S5a), which led to the identification of SEMA3B as potential target of the PNUTS-PP1 axis. This secreted semaphorin, as others of the axon guidance family of genes, was historically studied for its repulsion role in the guidance of neuronal growth in the developing neural system^50^, but new roles in endothelial barrier maintenance, preeclampsia and inhibition of angiogenesis have emerged^17,42,51,52^, which aligns with our findings. Little is known about the regulation of SEMA3B expression. Our study suggests that SEMA3B is induced in the absence of a working PNUTS-PP1 complex (Fig. 5b–g), and that it is the main actor in impeding the formation of a healthy endothelial barrier (Fig. 5h–j) upon loss of PNUTS. Finally, our results highlight a concept that explains loss of barrier function during aging in which dropping PNUTS levels result in aberrant activation of SEMA3B expression that induces repulsion of ECs.

In conclusion, this study provides evidence that PNUTS is critical for endothelial function during aging. Our data highlight the importance of PNUTS in providing a tight control of the endothelial gene expression profile, which is necessary to maintain barrier functionality through the regulation of the semaphorin family member SEMA3B, and as a potential clinical target against aging-related vascular hyperpermeability.

### Supplementary information

Supplementary information

Description of Additional Supplementary Files

Supplementary Data 1

Supplementary Data 2

Supplementary Data 3

Reporting Summary

## Data Availability

The RNA sequencing data can be found at the gene expression omnibus of NCBI under accession numbers GSE263531 and GSE263742. The source data behind the graphs in the paper can be found in Supplementary data 1. All additional data are available from the corresponding author on reasonable request.
