# Supplementary material for: Aging-regulated PNUTS maintains endothelial barrier function via SEMA3B suppression
Source: Commun Biol. 2024 May 7;7:541. doi: 10.1038/s42003-024-06230-5 (PMC11076560; doi:10.1038/s42003-024-06230-5)
Supplement: Supplementary file 1 — Supplementary information [file 42003_2024_6230_MOESM1_ESM.pdf]

Supplemental Figure S1

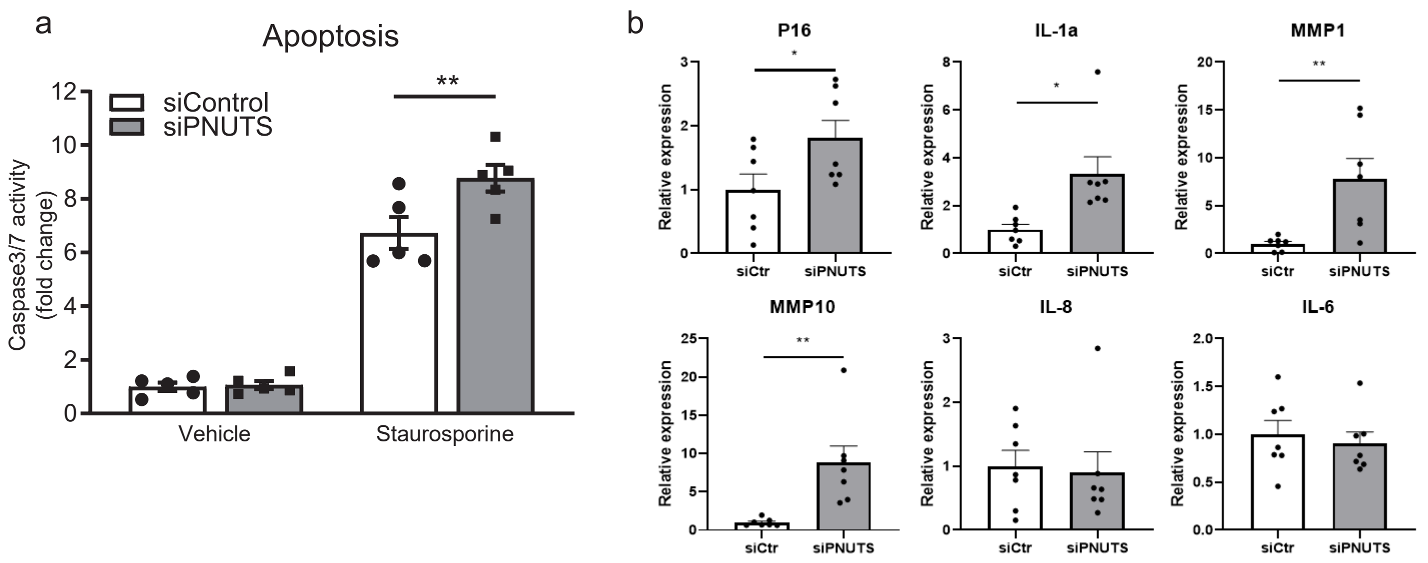

Supplemental Figure S2

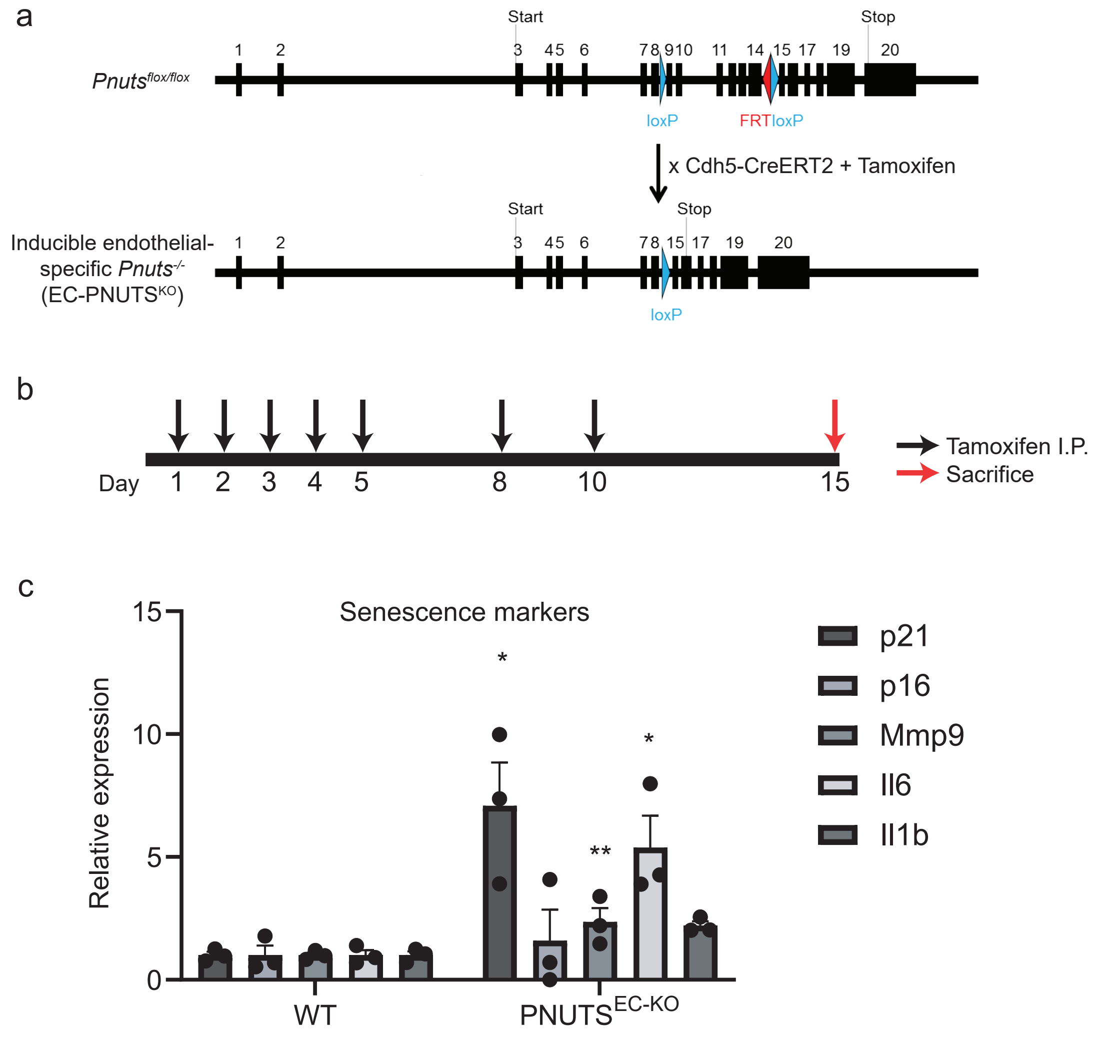

Supplemental Figure S3

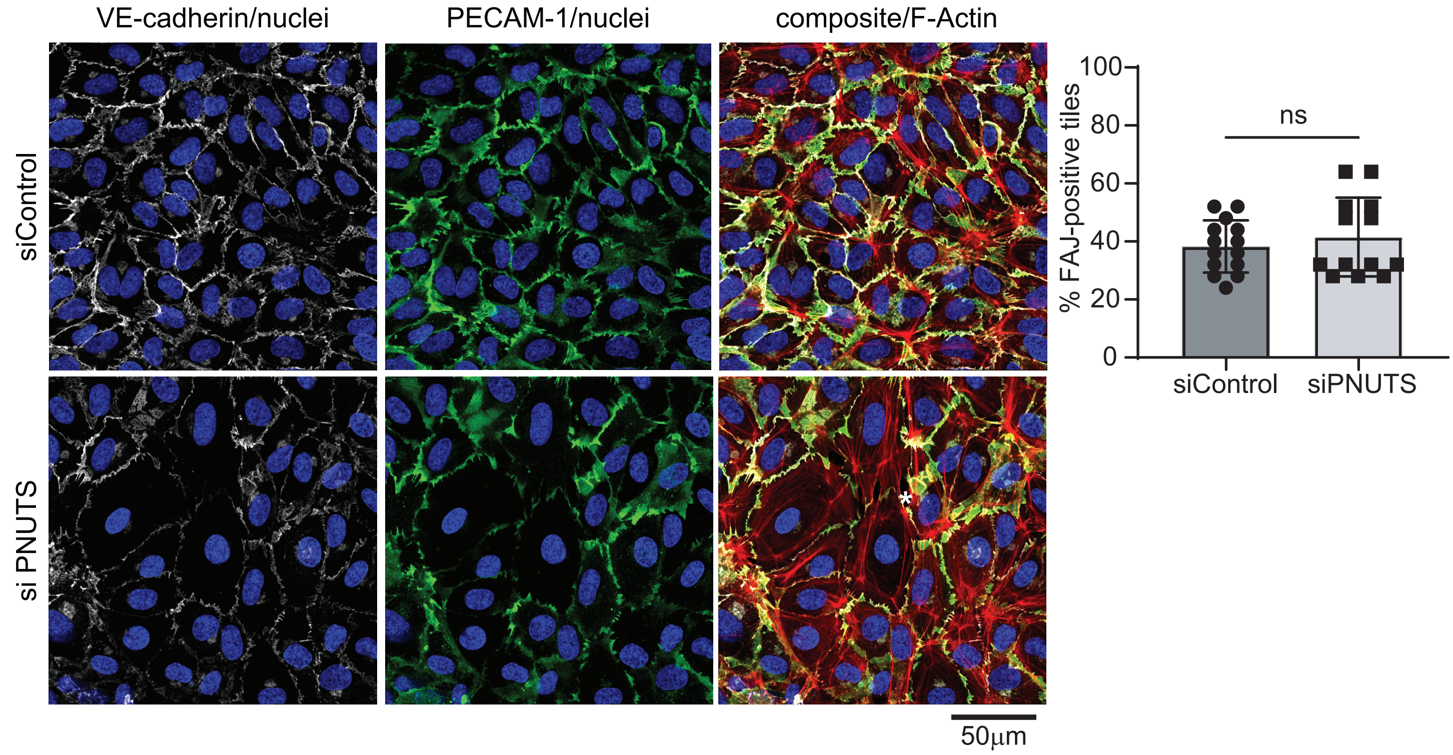

Supplemental Figure S4

a

KEGG pathway terms	P-Value	Bonferroni	Benjamini
hsa05203:Viral carcinogenesis	3.58E-11	1.06E-08	1.06E-08
hsa01100: Metabolic pathways	1.96E-09	5.79E-07	2.90E-07
hsa04110: Cell cycle	4.71E-09	1.39E-06	4.63E-07
hsa04142: Lysosome	1.99E-07	5.86E-05	1.17E-05
hsa04510: Focal adhesion	5.18E-07	1.53E-04	2.55E-05
hsa05212: Pancreatic cancer	9.97E-06	2.94E-03	3.67E-04
hsa04520: Adherens junction	5.99E-05	1.75E-02	1.77E-03
hsa03010: Ribosome	9.04E-05	2.63E-02	2.22E-03
hsa05222:Small cell lung cancer	0.00016	4.60E-02	3.62E-03
hsa04210: Apoptosis	0.000162	4.68E-02	3.41E-03

b

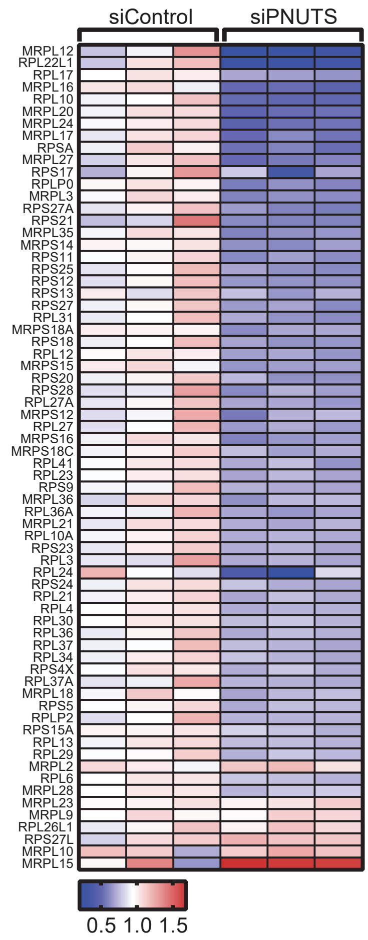

c

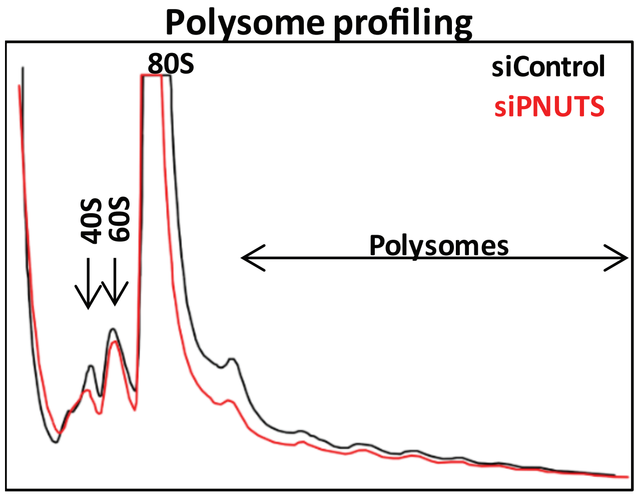

Supplemental Figure S5

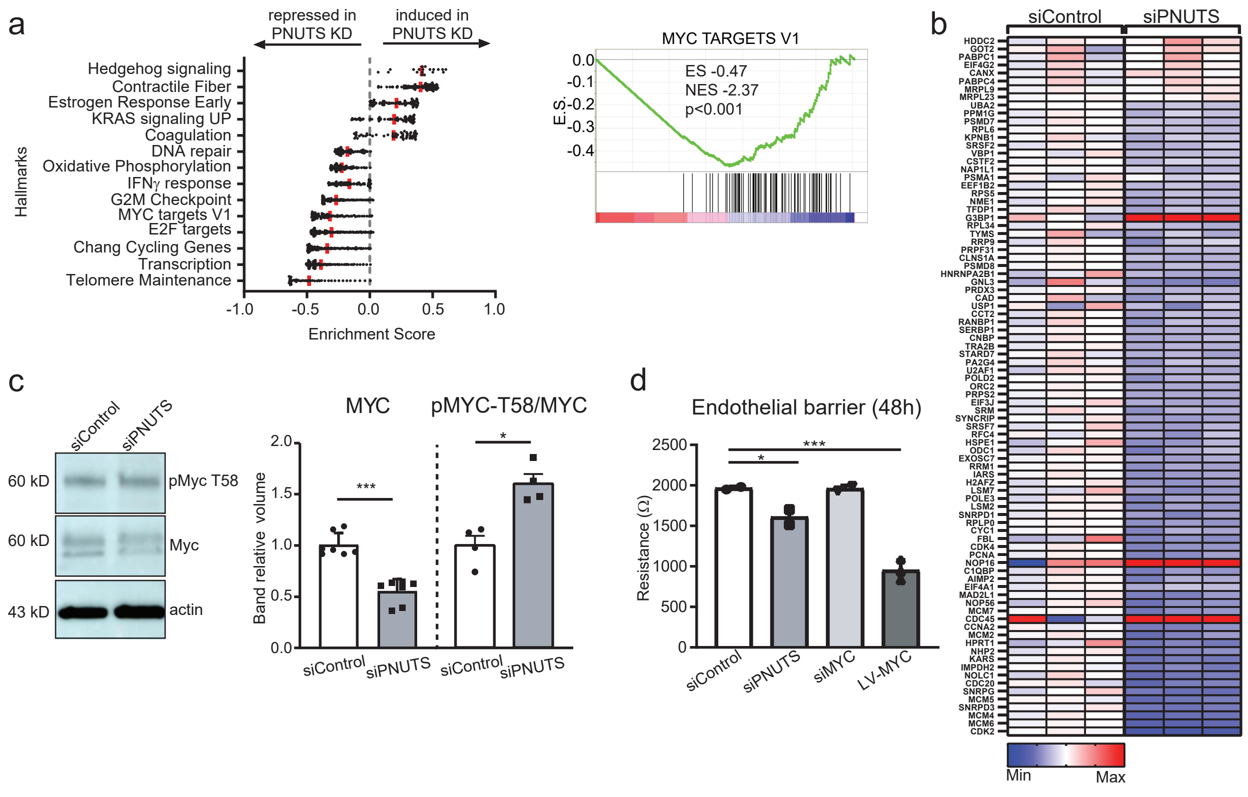

Supplemental Figure S6

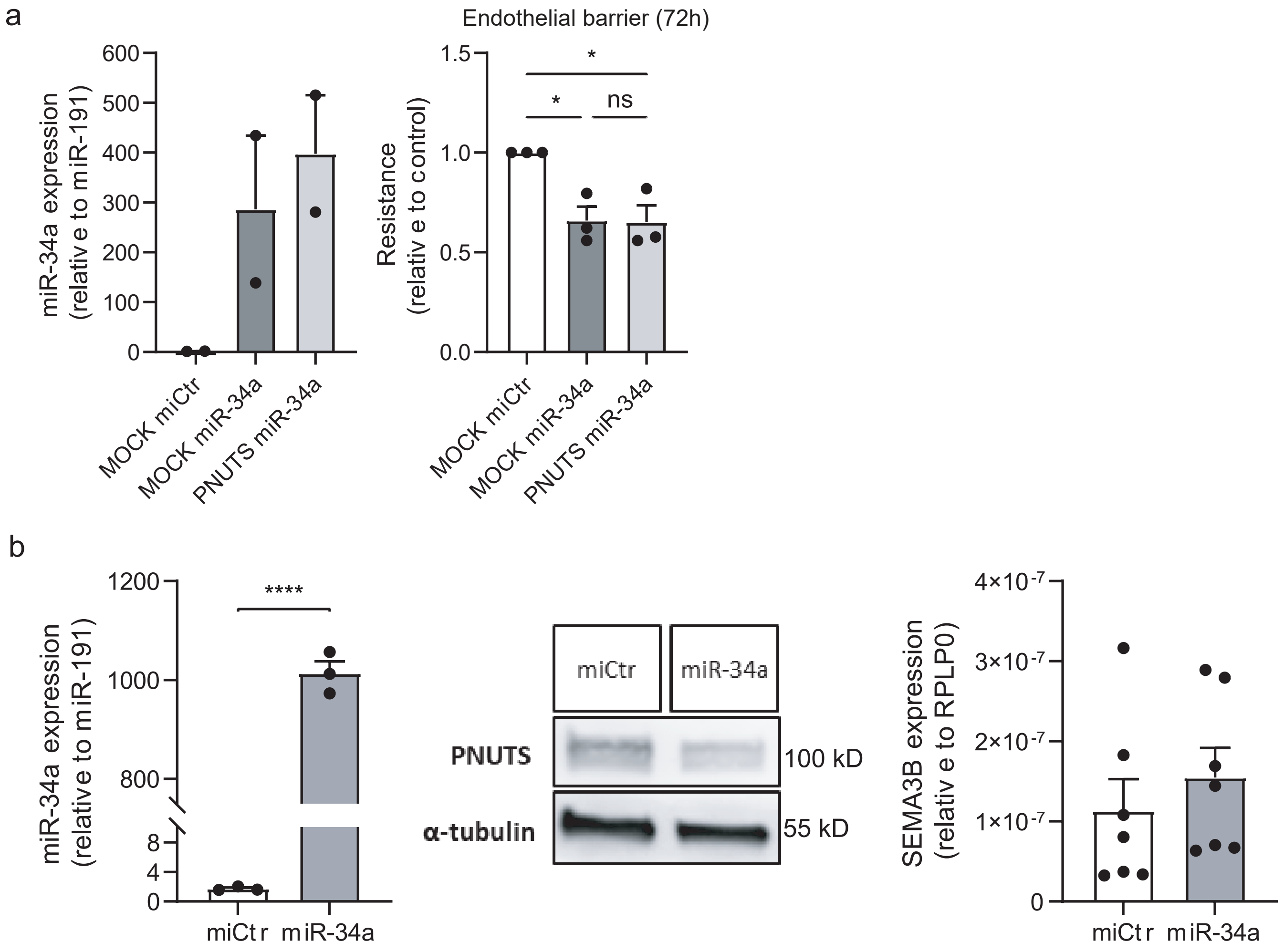

Supplemental figure S7

1c

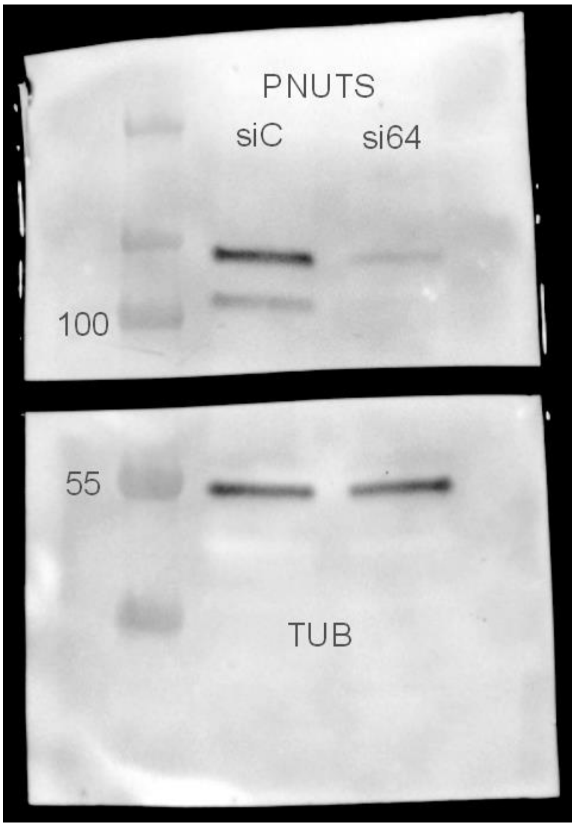

4g

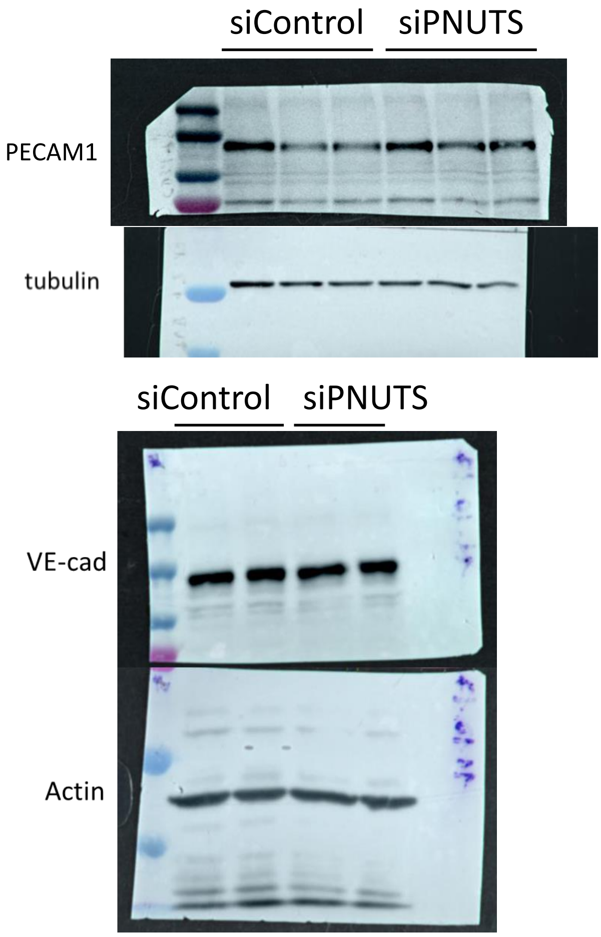

4j

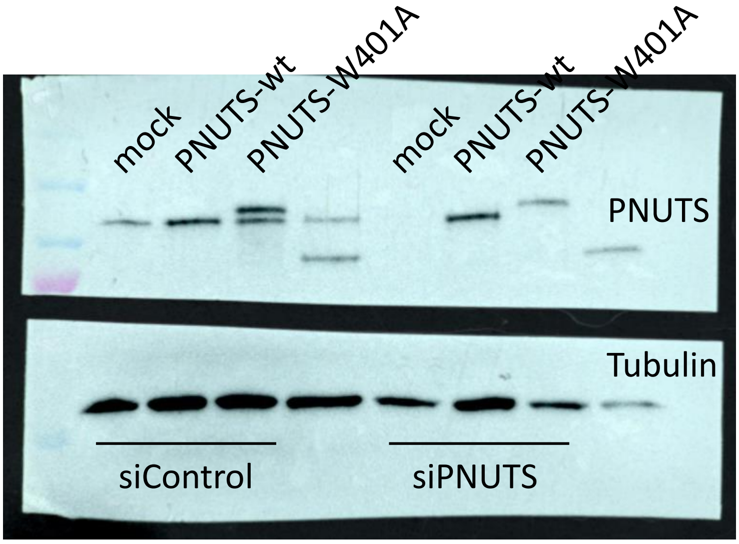

Suppl fig S5c

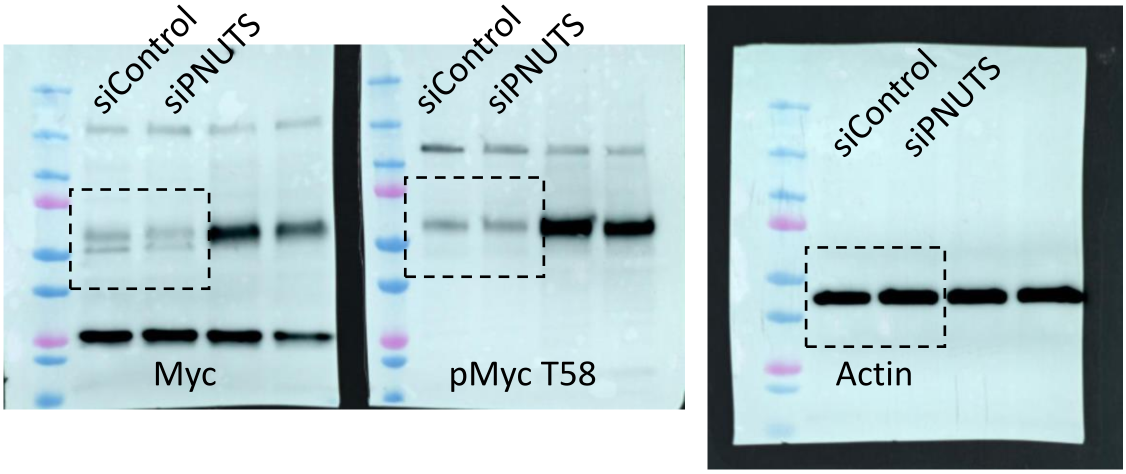

Suppl fig S6b

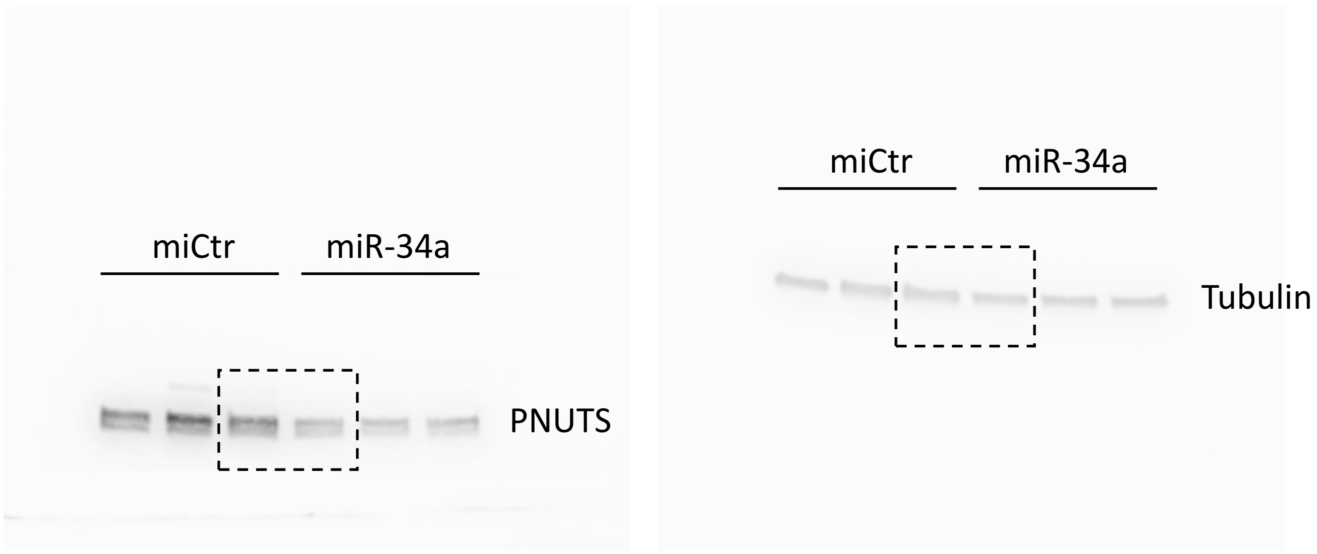

Supplemental Figure S1. a) HUVECs were transfected with siControl and siPNUTS for 48 h and subsequently treated with vehicle or Staurosporin 200 nM. Apoptosis was assayed by determining their Caspase 3/7 activity (n = 5). b) qPCR to measure senescence marker expression was performed 48 h after siRNA-mediated silencing of PNUTS and control HUVECs. n=7 per group. *p<0.05, **p<0.01. Error bars depict the standard error of the mean (SEM).

Supplemental Figure S2. a) Schematic representation of the PNUTS-floxed allele and further recombination of LoxP regions after crossing the $PNUTS^{fl/fl}$ mice with the inducible endothelial-specific Cdh5-CreERT2 strain and treatment with tamoxifen. b) Time course of the experimental work in mice. c) RNA-seq results of expression levels of senescence markers, performed in whole lung tissue of WT and $PNUTS^{EC\text{-}KO}$ mice. n=3 per group. *p<0.05 **p<0.01. Error bars depict the standard error of the mean (SEM).

Supplemental Figure S3. HUVECs were transfected with Control and anti-PNUTS siRNA (si). 72 h after transfection, full-grown monolayers were analyzed by IF staining using anti-VE-cadherin (white), anti-PECAM1 (green) and F-actin (red). DAPI (blue) was used to stain nuclei. Representative images are shown. * indicates an intercellular gap. Focal adherens junctions (FAJ) were visualized by VE-cadherin staining and are characterized as finger-like structures protruding into the cell body, more crosswise to the cell-cell border. F-actin staining was used to validate whether these structures are indeed focal adherens junctions, by looking for stress fibers spanning both cells and passing through the finger-like junction. Bar graph shows the percentage of FAJ-positive junctions between siControl and siPNUTS conditions. Junctions were divided in tiles of 38x38µm and are FAJ-positive if atleast one FAJ is present in the tile. Error bars depict the standard error of the mean (SEM).

Supplemental Figure S4. HUVECs were transfected with Control and anti-PNUTS siRNA (si) for 48 h. Total RNA was isolated and the transcriptome was studied by RNA-seq (n=3). a) Functional analysis of RNA-seq data clustered as KEGG pathway terms shows the differentially expressed pathways. b) Heat map of differentially regulated genes encoding for ribosomal proteins upon PNUTS KD. c) Polysome profiling of siControl (black line) and siPNUTS (red line) HUVECs. The consecutive peaks show the presence of 40S and 60S ribosome subunits, 80S ribosome (isolated ribosomes, normally inactive) and polysomes (two or more ribosomes bound to a single mRNA molecule, actively translating into protein) (n=3).

Supplemental Figure S5. a) Left, RNA-seq from PNUTS-silenced HUVECs was also analysed for differentially regulated pathways using GSEA. Enrichment scores of the indicated pathways are plotted on the *x* axis. Right, GSEA of MYC target datasets in PNUTS KD cells. ES, enrichment score; NES, normalized enrichment score. b) Heat map of differentially regulated MYC signature genes upon PNUTS KD. c) Left, representative WB of total cell lysates blotted with anti-MYC and anti-pMYC-T58 antibodies, anti-actin was used as loading control; right, densitometry quantification of MYC bands and pMYC-T58/MYC ratio in siControl and siPNUTS HUVECs. d) HUVECs were transfected with siRNAs against PNUTS and MYC or transduced with a lentiviral vector to overexpress MYC, and endothelial barrier resistance was assessed by ECIS (n=3, 4 biological replicates per group and experiment). *p<0.05, ***p<0.001. Error bars depict the standard error of the mean (SEM).

Supplemental Figure S6. a) miR-34a reduces endothelial barrier function independently of PNUTS. HUVECs were transduced with miR-34a-resistant lenti-PNUTS or lenti-Mock 5 days before measurement and transfected with miR-34a at 72 h before ECIS measurements. N=3 per group.

*p<0.05. b) miR-34a only mildly affects PNUTS and SEMA3B expression. HUVECs were transfected with miR-34a and PNUTS and SEMA3B expression were measured at 48h after transfection by Western blot and qPCR, respectively. n=7 per group. ****p<0.0001. Error bars depict the standard error of the mean (SEM).
