# Supplementary material for: Aging-regulated PNUTS maintains endothelial barrier function via SEMA3B suppression
Source: Commun Biol. 2024 May 7;7:541. doi: 10.1038/s42003-024-06230-5 (PMC11076560; doi:10.1038/s42003-024-06230-5)
Supplement: Supplementary file 2 — Description of Additional Supplementary Files [file 42003_2024_6230_MOESM2_ESM.pdf]

Description of Additional Supplementary Files

File name: Supplementary Data 1
Description: The source data behind the graphs in the paper.

File name: Supplementary Data 2
Description: RNA sequencing lungs of PNUTS$^{EC\text{-}KO}$ mice vs lungs of PNUTS^{WT} mice.

File name: Supplementary Data 3
Description: RNA sequencing siPNUTS (KD) vs siControl (Ctr) HUVECs.
