# Supplementary material for: Aging-regulated PNUTS maintains endothelial barrier function via SEMA3B suppression
Source: Commun Biol. 2024 May 7;7:541. doi: 10.1038/s42003-024-06230-5 (PMC11076560; doi:10.1038/s42003-024-06230-5)
Supplement: Supplementary file 4 — Supplementary Data 2 [file 42003_2024_6230_MOESM4_ESM.pdf]

ID	names	Ratio KO/WT	Fold change KO/WT	log2ratio	P-value	R-factor	Abs R
ENSMUSG00000024653	Scgb1a1	3.79E+11	3.79E+11	38.47	0.0048	5.46E+12	5.46E+12
ENSMUSG00000064351	mt-Co1	2.17E+10	2.17E+10	34.34	0.0064	2.72E+11	2.72E+11
ENSMUSG00000102333	Gm37235	7.91E-11	-1.26E+10	-33.56	0.0193	-9.10E+10	9.10E+10
ENSMUSG00000069833	Ahnak	3.50E+09	3.50E+09	31.71	0.0055	4.73E+10	4.73E+10
ENSMUSG00000064366	mt-Tl2	2.42E+09	2.42E+09	31.17	0.0105	2.35E+10	2.35E+10
ENSMUSG00000064823	Snord82	4.06E-10	-2.46E+09	-31.20	0.0468	-1.14E+10	1.14E+10
ENSMUSG00000104794	mmu-mir-28c	5.58E-10	-1.79E+09	-30.74	0.0287	-1.06E+10	1.06E+10
ENSMUSG00000088169	Gm22059	3.52E-09	-2.84E+08	-28.08	0.0019	-6.61E+09	6.61E+09
ENSMUSG00000098827	Gm27399	1.17E-09	-8.54E+08	-29.67	0.0201	-6.02E+09	6.02E+09
ENSMUSG00000088879	Gm25867	5.67E+08	5.67E+08	29.08	0.0248	3.60E+09	3.60E+09
ENSMUSG00000098539	Mir133c	1.67E-09	-5.99E+08	-29.16	0.0428	-2.90E+09	2.90E+09
ENSMUSG00000077158	Gm24578	3.43E+08	3.43E+08	28.36	0.0214	2.35E+09	2.35E+09
ENSMUSG00000092906	Gm25736	6.96E-09	-1.44E+08	-27.10	0.0057	-1.91E+09	1.91E+09
ENSMUSG00000080227	Gm13343	8.28E-09	-1.21E+08	-26.85	0.0057	-1.60E+09	1.60E+09
ENSMUSG00000077602	Gm22672	1.61E+08	1.61E+08	27.26	0.0109	1.54E+09	1.54E+09
ENSMUSG00000088713	Gm22729	4.86E-09	-2.06E+08	-27.62	0.0436	-9.86E+08	9.86E+08
ENSMUSG00000089188	Gm22942	1.68E-08	-5.94E+07	-25.82	0.0057	-7.89E+08	7.89E+08
ENSMUSG00000077278	Gm22778	1.92E-08	-5.21E+07	-25.64	0.0057	-6.93E+08	6.93E+08
ENSMUSG00000103930	Gm36961	2.14E-08	-4.67E+07	-25.48	0.0048	-6.73E+08	6.73E+08
ENSMUSG00000082864	Gm13358	1.65E+07	1.65E+07	23.97	0.0006	6.64E+08	6.64E+08
ENSMUSG00000104001	Gm38334	1.01E+07	1.01E+07	23.26	0.0005	4.36E+08	4.36E+08
ENSMUSG00000095891	Gm10717	6.26E-08	-1.60E+07	-23.93	0.0023	-3.36E+08	3.36E+08
ENSMUSG00000065371	Gm22739	1.43E-08	-7.01E+07	-26.06	0.0444	-3.33E+08	3.33E+08
ENSMUSG00000089309	Gm24838	1.56E-08	-6.40E+07	-25.93	0.0420	-3.13E+08	3.13E+08
ENSMUSG00000093616	1700055C04Rik	2.66E+07	2.66E+07	24.67	0.0101	2.65E+08	2.65E+08
ENSMUSG00000082670	Gm14050	1.50E+07	1.50E+07	23.84	0.0092	1.57E+08	1.57E+08
ENSMUSG00000097987	Gm17786	1.21E+07	1.21E+07	23.52	0.0101	1.20E+08	1.20E+08
ENSMUSG00000082741	Gm9703	1.57E+07	1.57E+07	23.91	0.0202	1.11E+08	1.11E+08
ENSMUSG00000055403	4933427D06Rik	1.84E+07	1.84E+07	24.13	0.0283	1.09E+08	1.09E+08
ENSMUSG00000100101	Gm29591	2.00E+07	2.00E+07	24.25	0.0433	9.61E+07	9.61E+07
ENSMUSG00000086732	Gm6117	1.36E+07	1.36E+07	23.70	0.0247	8.68E+07	8.68E+07
ENSMUSG00000102909	Gm2453	1.29E+07	1.29E+07	23.63	0.0228	8.56E+07	8.56E+07
ENSMUSG00000080820	Gm13197	1.56E-07	-6.40E+06	-22.61	0.0057	-8.51E+07	8.51E+07
ENSMUSG00000098162	Gm26976	1.60E+06	1.60E+06	20.61	0.0005	6.90E+07	6.90E+07
ENSMUSG00000056716	Gm5420	1.17E+07	1.17E+07	23.48	0.0311	6.62E+07	6.62E+07
ENSMUSG00000101603	Gm28730	1.88E-07	-5.33E+06	-22.35	0.0066	-6.58E+07	6.58E+07
ENSMUSG00000087148	1700030C14Rik	9.01E+06	9.01E+06	23.10	0.0275	5.43E+07	5.43E+07
ENSMUSG00000044286	Olfr221	7.34E+06	7.34E+06	22.81	0.0193	5.28E+07	5.28E+07
ENSMUSG00000040013	Fkbp6	3.29E+06	3.29E+06	21.65	0.0045	4.92E+07	4.92E+07
ENSMUSG00000105154	RP23-413C3.1	5.89E+06	5.89E+06	22.49	0.0152	4.77E+07	4.77E+07
ENSMUSG00000085074	Gm11494	4.58E+06	4.58E+06	22.13	0.0101	4.57E+07	4.57E+07
ENSMUSG00000107322	RP23-201C9.3	6.39E+06	6.39E+06	22.61	0.0247	4.06E+07	4.06E+07
ENSMUSG00000064281	Rpl19-ps1	3.22E+06	3.22E+06	21.62	0.0079	3.63E+07	3.63E+07
ENSMUSG00000050936	Hist2h2bb	7.76E+06	7.76E+06	22.89	0.0477	3.55E+07	3.55E+07
ENSMUSG00000053654	Krt42	4.43E+06	4.43E+06	22.08	0.0159	3.51E+07	3.51E+07
ENSMUSG00000042184	1700069L16Rik	6.16E+06	6.16E+06	22.55	0.0366	3.22E+07	3.22E+07
ENSMUSG00000101997	Gm28722	5.25E+06	5.25E+06	22.32	0.0286	3.10E+07	3.10E+07
ENSMUSG00000066723	Vmn1r183	3.43E+06	3.43E+06	21.71	0.0127	3.04E+07	3.04E+07
ENSMUSG00000025405	Inhbc	3.91E+06	3.91E+06	21.90	0.0172	2.98E+07	2.98E+07
ENSMUSG00000068117	Mei1	5.39E+05	5.39E+05	19.04	0.0004	2.78E+07	2.78E+07
ENSMUSG00000085017	Gm13412	5.67E+06	5.67E+06	22.43	0.0439	2.71E+07	2.71E+07
ENSMUSG00000051952	Olfr371	2.16E+06	2.16E+06	21.04	0.0069	2.61E+07	2.61E+07
ENSMUSG00000100837	1700063D05Rik	3.92E+06	3.92E+06	21.90	0.0237	2.55E+07	2.55E+07
ENSMUSG00000081524	Gm11658	2.05E+06	2.05E+06	20.97	0.0070	2.46E+07	2.46E+07
ENSMUSG00000085949	Gm14275	3.66E-07	-2.73E+06	-21.38	0.0129	-2.41E+07	2.41E+07
ENSMUSG00000105602	RP24-364E17.1	5.07E+05	5.07E+05	18.95	0.0005	2.19E+07	2.19E+07
ENSMUSG00000087559	Gm13802	2.25E+06	2.25E+06	21.10	0.0105	2.19E+07	2.19E+07
ENSMUSG00000044084	4933402P03Rik	4.31E+06	4.31E+06	22.04	0.0420	2.10E+07	2.10E+07
ENSMUSG00000092565	Gm20477	2.35E-07	-4.25E+06	-22.02	0.0420	-2.07E+07	2.07E+07
ENSMUSG00000071147	Tas2r140	3.17E+06	3.17E+06	21.59	0.0248	2.01E+07	2.01E+07
ENSMUSG00000086001	Gm13448	3.54E+06	3.54E+06	21.75	0.0332	1.94E+07	1.94E+07
ENSMUSG00000082924	Gm11462	3.87E+06	3.87E+06	21.88	0.0404	1.92E+07	1.92E+07
ENSMUSG00000082093	Gm15928	2.52E-07	-3.97E+06	-21.92	0.0428	-1.92E+07	1.92E+07
ENSMUSG00000099718	Gm2087	3.75E-07	-2.66E+06	-21.35	0.0201	-1.88E+07	1.88E+07
ENSMUSG00000102688	Gm37218	1.85E+06	1.85E+06	20.82	0.0105	1.80E+07	1.80E+07
ENSMUSG00000082967	Gm16082	4.00E-07	-2.50E+06	-21.25	0.0201	-1.76E+07	1.76E+07

ENSMUSG00000037390	Muc3	1.65E+06	1.65E+06	20.65	0.0088	1.76E+07	1.76E+07
ENSMUSG00000105650	RP23-123I10.3	6.41E-07	-1.56E+06	-20.57	0.0085	-1.69E+07	1.69E+07
ENSMUSG00000013928	Thap8	3.38E+06	3.38E+06	21.69	0.0467	1.56E+07	1.56E+07
ENSMUSG00000061877	BC048679	2.60E+06	2.60E+06	21.31	0.0294	1.52E+07	1.52E+07
ENSMUSG00000104397	Gm37958	1.93E+06	1.93E+06	20.88	0.0168	1.49E+07	1.49E+07
ENSMUSG00000082943	Gm13355	3.29E-07	-3.04E+06	-21.53	0.0420	-1.48E+07	1.48E+07
ENSMUSG00000050933	Vmn1r231	2.05E+06	2.05E+06	20.96	0.0198	1.45E+07	1.45E+07
ENSMUSG00000078586	Gm11735	2.67E+06	2.67E+06	21.35	0.0350	1.43E+07	1.43E+07
ENSMUSG00000071303	Rps8-ps1	9.41E-07	-1.06E+06	-20.02	0.0057	-1.41E+07	1.41E+07
ENSMUSG00000075524	4930407I10Rik	2.26E+06	2.26E+06	21.11	0.0262	1.39E+07	1.39E+07
ENSMUSG00000102295	Ighv1-57	4.16E-07	-2.40E+06	-21.20	0.0428	-1.16E+07	1.16E+07
ENSMUSG00000103007	Gm20690	4.33E-07	-2.31E+06	-21.14	0.0420	-1.13E+07	1.13E+07
ENSMUSG00000086649	Gm15286	2.00E+06	2.00E+06	20.93	0.0333	1.09E+07	1.09E+07
ENSMUSG00000035946	Gsx2	2.28E+06	2.28E+06	21.12	0.0476	1.05E+07	1.05E+07
ENSMUSG00000091707	Gm8224	6.77E-07	-1.48E+06	-20.49	0.0201	-1.04E+07	1.04E+07
ENSMUSG00000072707	Olfr31	2.27E+06	2.27E+06	21.11	0.0492	1.02E+07	1.02E+07
ENSMUSG00000097384	Gm26815	1.64E+06	1.64E+06	20.64	0.0274	9.89E+06	9.89E+06
ENSMUSG00000097981	Gm7583	2.12E+06	2.12E+06	21.02	0.0492	9.58E+06	9.58E+06
ENSMUSG00000019146	Cacng2	1.72E+06	1.72E+06	20.72	0.0377	8.87E+06	8.87E+06
ENSMUSG00000095030	Olfr1513	1.23E+06	1.23E+06	20.23	0.0214	8.43E+06	8.43E+06
ENSMUSG00000080948	Gm14481	1.59E-06	-6.30E+05	-19.26	0.0057	-8.37E+06	8.37E+06
ENSMUSG00000102858	Gm37086	1.35E+06	1.35E+06	20.37	0.0263	8.36E+06	8.36E+06
ENSMUSG00000104098	AA619741	1.65E-06	-6.06E+05	-19.21	0.0057	-8.04E+06	8.04E+06
ENSMUSG00000102372	A430050A11Rik	6.31E-07	-1.59E+06	-20.60	0.0428	-7.66E+06	7.66E+06
ENSMUSG00000045019	Acer1	1.09E+06	1.09E+06	20.05	0.0223	7.26E+06	7.26E+06
ENSMUSG00000098159	Gm21614	1.02E-06	-9.77E+05	-19.90	0.0189	-7.11E+06	7.11E+06
ENSMUSG00000029663	Gngt1	1.50E+06	1.50E+06	20.52	0.0447	7.09E+06	7.09E+06
ENSMUSG00000042774	Olfr1353	1.21E+06	1.21E+06	20.21	0.0313	6.84E+06	6.84E+06
ENSMUSG00000021961	4930578I06Rik	1.42E+06	1.42E+06	20.44	0.0431	6.83E+06	6.83E+06
ENSMUSG00000085931	Gm12648	1.04E-06	-9.57E+05	-19.87	0.0201	-6.75E+06	6.75E+06
ENSMUSG00000103631	Gm38060	7.43E-07	-1.35E+06	-20.36	0.0420	-6.57E+06	6.57E+06
ENSMUSG00000050763	Olfr1395	1.36E+06	1.36E+06	20.38	0.0433	6.55E+06	6.55E+06
ENSMUSG00000097265	Gm26803	8.41E+05	8.41E+05	19.68	0.0198	5.97E+06	5.97E+06
ENSMUSG00000022061	Nkx3-1	6.02E+05	6.02E+05	19.20	0.0118	5.56E+06	5.56E+06
ENSMUSG00000041670	Rims1	2.71E-06	-3.70E+05	-18.50	0.0047	-5.42E+06	5.42E+06
ENSMUSG00000105206	RP23-158A20.2	1.12E+06	1.12E+06	20.10	0.0431	5.41E+06	5.41E+06
ENSMUSG00000021907	Msmb	9.52E-07	-1.05E+06	-20.00	0.0420	-5.13E+06	5.13E+06
ENSMUSG00000075072	Olfr48	2.62E-06	-3.81E+05	-18.54	0.0057	-5.06E+06	5.06E+06
ENSMUSG00000097102	2310069G16Rik	1.47E-06	-6.79E+05	-19.37	0.0201	-4.79E+06	4.79E+06
ENSMUSG00000078300	Gm2606	2.98E-06	-3.35E+05	-18.36	0.0057	-4.46E+06	4.46E+06
ENSMUSG00000061561	Olfr373	8.87E+05	8.87E+05	19.76	0.0461	4.13E+06	4.13E+06
ENSMUSG00000102404	5530400K19Rik	7.04E+05	7.04E+05	19.43	0.0298	4.08E+06	4.08E+06
ENSMUSG00000040138	Ndp	1.62E-06	-6.17E+05	-19.24	0.0229	-4.07E+06	4.07E+06
ENSMUSG00000082039	Gm15653	8.05E+05	8.05E+05	19.62	0.0427	3.89E+06	3.89E+06
ENSMUSG00000104417	Gm37068	1.24E-06	-8.03E+05	-19.62	0.0428	-3.88E+06	3.88E+06
ENSMUSG00000085540	Gm16036	1.30E-06	-7.71E+05	-19.56	0.0428	-3.73E+06	3.73E+06
ENSMUSG00000087668	Gm11186	3.64E-06	-2.74E+05	-18.07	0.0057	-3.65E+06	3.65E+06
ENSMUSG00000040420	Cdh18	4.81E-06	-2.08E+05	-17.66	0.0033	-3.62E+06	3.62E+06
ENSMUSG00000106419	RP23-287M3.1	2.02E-06	-4.96E+05	-18.92	0.0201	-3.50E+06	3.50E+06
ENSMUSG00000081749	Gm12241	1.49E-06	-6.70E+05	-19.35	0.0420	-3.27E+06	3.27E+06
ENSMUSG00000085924	Gm8980	5.48E+05	5.48E+05	19.06	0.0288	3.23E+06	3.23E+06
ENSMUSG00000097237	Gm26519	6.94E+05	6.94E+05	19.40	0.0479	3.17E+06	3.17E+06
ENSMUSG00000105248	RP23-29F15.2	4.88E-06	-2.05E+05	-17.64	0.0057	-2.72E+06	2.72E+06
ENSMUSG00000101563	Gm7364	5.00E+05	5.00E+05	18.93	0.0386	2.55E+06	2.55E+06
ENSMUSG00000102508	Gm37367	2.77E-06	-3.61E+05	-18.46	0.0201	-2.55E+06	2.55E+06
ENSMUSG00000089822	Gm15759	4.28E+05	4.28E+05	18.71	0.0296	2.49E+06	2.49E+06
ENSMUSG00000085748	Gm12280	3.07E+05	3.07E+05	18.23	0.0168	2.36E+06	2.36E+06
ENSMUSG00000104148	Pcdha2	4.82E-06	-2.08E+05	-17.66	0.0083	-2.27E+06	2.27E+06
ENSMUSG00000104905	RP23-192D21.2	2.36E-06	-4.24E+05	-18.69	0.0428	-2.05E+06	2.05E+06
ENSMUSG00000107117	RP23-72G21.1	4.50E-06	-2.22E+05	-17.76	0.0133	-1.92E+06	1.92E+06
ENSMUSG00000067144	Slc22a7	1.91E+05	1.91E+05	17.54	0.0105	1.86E+06	1.86E+06
ENSMUSG00000098854	Gm5118	2.18E+05	2.18E+05	17.73	0.0168	1.68E+06	1.68E+06
ENSMUSG00000063953	Amd2	6.93E-06	-1.44E+05	-17.14	0.0201	-1.02E+06	1.02E+06
ENSMUSG00000106694	RP24-559P3.1	7.37E-06	-1.36E+05	-17.05	0.0201	-9.58E+05	9.58E+05
ENSMUSG00000041138	Nme8	6.48E-06	-1.54E+05	-17.24	0.0261	-9.55E+05	9.55E+05
ENSMUSG00000060780	Lrrtm1	7.15E-06	-1.40E+05	-17.09	0.0227	-9.28E+05	9.28E+05
ENSMUSG00000106677	Ugt2a1	1.30E-04	-7.71E+03	-12.91	0.0002	-5.35E+05	5.35E+05

ENSMUSG00000060371	Caln1	2.61E-05	-3.84E+04	-15.23	0.0420	-1.87E+05	1.87E+05
ENSMUSG00000106232	RP24-362N9.4	3.30E-04	-3.03E+03	-11.57	0.0418	-1.48E+04	1.48E+04
ENSMUSG00000096764	Gm21985	1.87E+03	1.87E+03	10.87	0.0194	1.34E+04	1.34E+04
ENSMUSG00000107162	RP23-382I10.3	3.28E+01	3.28E+01	5.04	0.0000	5.26E+03	5.26E+03
ENSMUSG00000049350	Zg16	1.42E+01	1.42E+01	3.82	0.0000	2.09E+03	2.09E+03
ENSMUSG00000075070	4932412D23Rik	3.24E+02	3.24E+02	8.34	0.0369	1.68E+03	1.68E+03
ENSMUSG00000081445	Rpl23a-ps4	1.26E+01	1.26E+01	3.66	0.0001	1.44E+03	1.44E+03
ENSMUSG00000035506	Slc12a8	1.05E+01	1.05E+01	3.39	0.0001	1.32E+03	1.32E+03
ENSMUSG00000089789	Rdh1	6.92E+00	6.92E+00	2.79	0.0000	1.09E+03	1.09E+03
ENSMUSG00000042672	Dcst1	5.65E+00	5.65E+00	2.50	0.0000	1.01E+03	1.01E+03
ENSMUSG00000105311	RP23-214I6.5	1.28E+02	1.28E+02	7.01	0.0225	8.57E+02	8.57E+02
ENSMUSG00000090107	Gm11492	4.56E+01	4.56E+01	5.51	0.0030	8.37E+02	8.37E+02
ENSMUSG00000078235	Fam43b	1.75E+01	1.75E+01	4.13	0.0005	7.99E+02	7.99E+02
ENSMUSG00000082786	Gm14489	1.05E+02	1.05E+02	6.71	0.0181	7.78E+02	7.78E+02
ENSMUSG00000079584	Gm364	1.59E+01	1.59E+01	3.99	0.0004	7.72E+02	7.72E+02
ENSMUSG00000044903	Psg22	2.78E+01	2.78E+01	4.80	0.0014	7.44E+02	7.44E+02
ENSMUSG00000024223	Armc12	1.22E+02	1.22E+02	6.94	0.0279	7.32E+02	7.32E+02
ENSMUSG00000091402	Rd3l	5.71E+01	5.71E+01	5.83	0.0065	7.06E+02	7.06E+02
ENSMUSG00000000632	Sez6	1.47E+02	1.47E+02	7.20	0.0453	6.90E+02	6.90E+02
ENSMUSG00000106271	RP24-105J12.3	3.47E+01	3.47E+01	5.12	0.0027	6.68E+02	6.68E+02
ENSMUSG00000022487	Gtsf1	1.96E+01	1.96E+01	4.30	0.0009	6.61E+02	6.61E+02
ENSMUSG00000097100	9230104M06Rik	4.81E+00	4.81E+00	2.27	0.0001	6.46E+02	6.46E+02
ENSMUSG00000039342	Ankar	1.08E+02	1.08E+02	6.75	0.0295	6.26E+02	6.26E+02
ENSMUSG00000097604	Gm17322	8.70E+01	8.70E+01	6.44	0.0202	6.12E+02	6.12E+02
ENSMUSG00000081857	Gm13624	1.20E+01	1.20E+01	3.58	0.0004	6.07E+02	6.07E+02
ENSMUSG00000031766	Slc12a3	1.03E+01	1.03E+01	3.36	0.0003	6.04E+02	6.04E+02
ENSMUSG00000092246	Gm20493	4.12E+01	4.12E+01	5.36	0.0049	5.89E+02	5.89E+02
ENSMUSG00000041644	Slc5a12	6.86E-02	-1.46E+01	-3.86	0.0006	-5.88E+02	5.88E+02
ENSMUSG00000087399	Gm11899	6.71E-02	-1.49E+01	-3.90	0.0007	-5.71E+02	5.71E+02
ENSMUSG00000100625	1700016G22Rik	1.26E+02	1.26E+02	6.98	0.0496	5.66E+02	5.66E+02
ENSMUSG00000030495	Slc7a10	1.27E-01	-7.89E+00	-2.98	0.0002	-5.48E+02	5.48E+02
ENSMUSG00000014686	Ceacam16	6.34E+00	6.34E+00	2.66	0.0001	5.44E+02	5.44E+02
ENSMUSG00000028661	Epha8	7.70E+01	7.70E+01	6.27	0.0202	5.42E+02	5.42E+02
ENSMUSG00000106999	RP23-153H17.3	3.67E+01	3.67E+01	5.20	0.0054	4.98E+02	4.98E+02
ENSMUSG00000051036	Ttc24	8.55E+01	8.55E+01	6.42	0.0306	4.89E+02	4.89E+02
ENSMUSG00000087443	Ppp1r18os	2.35E+01	2.35E+01	4.56	0.0025	4.67E+02	4.67E+02
ENSMUSG00000104416	Gm37067	2.52E-02	-3.96E+01	-5.31	0.0074	-4.60E+02	4.60E+02
ENSMUSG00000101588	Gm28265	2.91E+01	2.91E+01	4.86	0.0042	4.49E+02	4.49E+02
ENSMUSG00000043747	1520401A03Rik	9.69E+01	9.69E+01	6.60	0.0499	4.34E+02	4.34E+02
ENSMUSG00000029235	Pdcl2	9.95E+00	9.95E+00	3.31	0.0006	4.14E+02	4.14E+02
ENSMUSG00000100334	C230024C17Rik	7.18E+01	7.18E+01	6.17	0.0315	4.05E+02	4.05E+02
ENSMUSG00000099452	Gm28502	4.37E+01	4.37E+01	5.45	0.0118	4.02E+02	4.02E+02
ENSMUSG00000025433	Crisp3	7.72E-02	-1.30E+01	-3.70	0.0010	-4.01E+02	4.01E+02
ENSMUSG00000072625	Gdf2	8.35E-02	-1.20E+01	-3.58	0.0010	-3.88E+02	3.88E+02
ENSMUSG00000020712	Tcam1	5.12E+01	5.12E+01	5.68	0.0174	3.88E+02	3.88E+02
ENSMUSG00000086125	Gm15942	9.43E-02	-1.06E+01	-3.41	0.0008	-3.84E+02	3.84E+02
ENSMUSG00000078444	Gm10941	9.88E+00	9.88E+00	3.30	0.0007	3.80E+02	3.80E+02
ENSMUSG00000090129	Olfr287	8.39E+01	8.39E+01	6.39	0.0499	3.75E+02	3.75E+02
ENSMUSG00000046490	Rnf222	1.11E+01	1.11E+01	3.48	0.0009	3.70E+02	3.70E+02
ENSMUSG00000085307	Gm11525	1.32E+01	1.32E+01	3.72	0.0013	3.68E+02	3.68E+02
ENSMUSG00000050334	C130071C03Rik	1.98E+01	1.98E+01	4.31	0.0032	3.50E+02	3.50E+02
ENSMUSG00000044172	Ptx4	5.36E+01	5.36E+01	5.74	0.0236	3.49E+02	3.49E+02
ENSMUSG00000083178	Gm12187	7.75E+00	7.75E+00	2.95	0.0005	3.46E+02	3.46E+02
ENSMUSG00000011463	Cpb1	2.21E-01	-4.52E+00	-2.18	0.0002	-3.41E+02	3.41E+02
ENSMUSG00000097848	Gm807	2.92E+01	2.92E+01	4.87	0.0080	3.27E+02	3.27E+02
ENSMUSG00000057606	Colq	1.20E-01	-8.34E+00	-3.06	0.0007	-3.11E+02	3.11E+02
ENSMUSG00000021200	Asb2	2.46E-01	-4.07E+00	-2.02	0.0002	-3.04E+02	3.04E+02
ENSMUSG00000042078	Svop	4.41E+01	4.41E+01	5.46	0.0212	3.03E+02	3.03E+02
ENSMUSG00000103457	Gm37042	8.44E+00	8.44E+00	3.08	0.0008	3.00E+02	3.00E+02
ENSMUSG00000083090	Gm11736	5.07E+01	5.07E+01	5.66	0.0298	2.94E+02	2.94E+02
ENSMUSG00000107167	RP23-446N16.6	5.32E+01	5.32E+01	5.73	0.0331	2.92E+02	2.92E+02
ENSMUSG00000101414	Gm29101	4.88E-02	-2.05E+01	-4.36	0.0050	-2.90E+02	2.90E+02
ENSMUSG00000002289	Angptl4	2.72E+00	2.72E+00	1.44	0.0001	2.89E+02	2.89E+02
ENSMUSG00000010086	Rnf112	7.78E-02	-1.29E+01	-3.68	0.0020	-2.89E+02	2.89E+02
ENSMUSG00000055523	Gucy2g	6.17E+00	6.17E+00	2.63	0.0005	2.83E+02	2.83E+02
ENSMUSG00000086321	Gm11413	2.06E+01	2.06E+01	4.36	0.0053	2.82E+02	2.82E+02
ENSMUSG00000078640	Gm11627	6.33E-02	-1.58E+01	-3.98	0.0033	-2.77E+02	2.77E+02

ENSMUSG00000097676	Gm26612	2.96E+01	2.96E+01	4.89	0.0118	2.72E+02	2.72E+02
ENSMUSG00000036790	Slitrk2	2.12E-02	-4.72E+01	-5.56	0.0305	-2.70E+02	2.70E+02
ENSMUSG00000071532	Gm10335	8.71E-02	-1.15E+01	-3.52	0.0019	-2.65E+02	2.65E+02
ENSMUSG00000069189	Zdhhc11	4.66E+01	4.66E+01	5.54	0.0311	2.64E+02	2.64E+02
ENSMUSG00000107208	RP23-319P12.6	4.79E-01	-2.09E+00	-1.06	0.0001	-2.60E+02	2.60E+02
ENSMUSG00000023045	Soat2	4.57E+00	4.57E+00	2.19	0.0003	2.54E+02	2.54E+02
ENSMUSG00000041293	Adgrf1	2.52E+01	2.52E+01	4.66	0.0099	2.53E+02	2.53E+02
ENSMUSG00000085976	Gm13816	1.53E-01	-6.51E+00	-2.70	0.0007	-2.52E+02	2.52E+02
ENSMUSG00000097184	4632428C04Rik	2.86E-01	-3.50E+00	-1.81	0.0002	-2.47E+02	2.47E+02
ENSMUSG00000082360	Gm8814	1.94E+01	1.94E+01	4.28	0.0062	2.47E+02	2.47E+02
ENSMUSG00000055061	4931431C16Rik	4.34E+00	4.34E+00	2.12	0.0003	2.41E+02	2.41E+02
ENSMUSG00000041552	Ptchd1	6.84E-02	-1.46E+01	-3.87	0.0037	-2.40E+02	2.40E+02
ENSMUSG00000103473	Gm37696	4.49E+00	4.49E+00	2.17	0.0004	2.37E+02	2.37E+02
ENSMUSG00000099924	Gm28320	2.93E-01	-3.41E+00	-1.77	0.0002	-2.33E+02	2.33E+02
ENSMUSG00000027233	Patl2	2.80E+01	2.80E+01	4.81	0.0144	2.33E+02	2.33E+02
ENSMUSG00000040314	Ctsg	8.86E+00	8.86E+00	3.15	0.0015	2.32E+02	2.32E+02
ENSMUSG00000095642	Ighv14-3	5.64E+00	5.64E+00	2.50	0.0006	2.30E+02	2.30E+02
ENSMUSG00000098778	Gm27231	2.19E-02	-4.56E+01	-5.51	0.0399	-2.29E+02	2.29E+02
ENSMUSG00000026824	Kcnj3	1.32E-01	-7.60E+00	-2.93	0.0012	-2.23E+02	2.23E+02
ENSMUSG00000060332	Tmc2	7.98E+00	7.98E+00	3.00	0.0013	2.23E+02	2.23E+02
ENSMUSG00000004542	Psg19	2.88E+01	2.88E+01	4.85	0.0170	2.21E+02	2.21E+02
ENSMUSG00000086595	Gm12214	8.59E+00	8.59E+00	3.10	0.0016	2.17E+02	2.17E+02
ENSMUSG00000067279	Ppp1r3c	1.74E-01	-5.75E+00	-2.52	0.0007	-2.14E+02	2.14E+02
ENSMUSG00000066319	Rtp3	1.14E-01	-8.79E+00	-3.14	0.0017	-2.13E+02	2.13E+02
ENSMUSG00000104875	RP23-301L9.4	3.35E+01	3.35E+01	5.06	0.0272	2.03E+02	2.03E+02
ENSMUSG00000029848	Stra8	4.04E+01	4.04E+01	5.34	0.0402	2.02E+02	2.02E+02
ENSMUSG00000097036	1110036E04Rik	4.40E+01	4.40E+01	5.46	0.0480	2.01E+02	2.01E+02
ENSMUSG00000086275	1700121C08Rik	2.12E+01	2.12E+01	4.40	0.0111	2.01E+02	2.01E+02
ENSMUSG00000025902	Sox17	2.14E-01	-4.67E+00	-2.22	0.0006	-1.99E+02	1.99E+02
ENSMUSG00000019230	Lhx9	2.73E+01	2.73E+01	4.77	0.0194	1.96E+02	1.96E+02
ENSMUSG00000031957	Ctrb1	3.37E+01	3.37E+01	5.07	0.0302	1.94E+02	1.94E+02
ENSMUSG00000053461	Hhipl2	8.73E+00	8.73E+00	3.13	0.0021	1.92E+02	1.92E+02
ENSMUSG00000048626	Klf17	3.83E+01	3.83E+01	5.26	0.0403	1.91E+02	1.91E+02
ENSMUSG00000059540	Tcea2	2.36E+00	2.36E+00	1.24	0.0002	1.90E+02	1.90E+02
ENSMUSG00000100129	Gm29064	9.31E+00	9.31E+00	3.22	0.0024	1.89E+02	1.89E+02
ENSMUSG00000049001	Ndnf	2.94E-01	-3.40E+00	-1.77	0.0003	-1.88E+02	1.88E+02
ENSMUSG00000036907	C1ql2	5.35E-02	-1.87E+01	-4.22	0.0100	-1.87E+02	1.87E+02
ENSMUSG00000028946	Hes3	2.10E+01	2.10E+01	4.40	0.0131	1.84E+02	1.84E+02
ENSMUSG00000105444	RP24-291E8.3	7.31E+00	7.31E+00	2.87	0.0016	1.81E+02	1.81E+02
ENSMUSG00000061911	Myt1l	1.91E+01	1.91E+01	4.25	0.0112	1.81E+02	1.81E+02
ENSMUSG00000078921	Tgtp2	2.84E-01	-3.52E+00	-1.81	0.0004	-1.79E+02	1.79E+02
ENSMUSG00000032921	Odf4	1.35E+01	1.35E+01	3.75	0.0056	1.79E+02	1.79E+02
ENSMUSG00000084785	Gm13972	2.14E+01	2.14E+01	4.42	0.0150	1.75E+02	1.75E+02
ENSMUSG00000078721	Gm12429	4.31E+00	4.31E+00	2.11	0.0006	1.73E+02	1.73E+02
ENSMUSG00000039760	Il22ra2	3.91E+00	3.91E+00	1.97	0.0005	1.71E+02	1.71E+02
ENSMUSG00000026413	Pkp1	3.21E+01	3.21E+01	5.00	0.0356	1.70E+02	1.70E+02
ENSMUSG00000097369	Gm26545	5.98E+00	5.98E+00	2.58	0.0012	1.69E+02	1.69E+02
ENSMUSG00000034145	Tmem63c	3.91E+00	3.91E+00	1.97	0.0006	1.67E+02	1.67E+02
ENSMUSG00000105352	RP23-195B3.5	1.45E+01	1.45E+01	3.86	0.0079	1.64E+02	1.64E+02
ENSMUSG00000033044	Dhrs7c	1.17E-01	-8.54E+00	-3.09	0.0027	-1.63E+02	1.63E+02
ENSMUSG00000005892	Trh	9.71E+00	9.71E+00	3.28	0.0036	1.63E+02	1.63E+02
ENSMUSG00000101828	D130051D11Rik	1.61E+01	1.61E+01	4.01	0.0098	1.63E+02	1.63E+02
ENSMUSG00000079355	Ackr4	4.48E-01	-2.23E+00	-1.16	0.0002	-1.62E+02	1.62E+02
ENSMUSG00000103727	Gm37097	2.34E+01	2.34E+01	4.55	0.0228	1.55E+02	1.55E+02
ENSMUSG00000022249	Ttc23l	2.52E+01	2.52E+01	4.66	0.0266	1.55E+02	1.55E+02
ENSMUSG00000084382	Gm3267	4.83E-02	-2.07E+01	-4.37	0.0180	-1.54E+02	1.54E+02
ENSMUSG00000038193	Hand2	9.17E-02	-1.09E+01	-3.45	0.0051	-1.53E+02	1.53E+02
ENSMUSG00000097974	Gm10605	1.01E+01	1.01E+01	3.34	0.0045	1.51E+02	1.51E+02
ENSMUSG00000093536	Smim17	1.62E-01	-6.19E+00	-2.63	0.0017	-1.50E+02	1.50E+02
ENSMUSG00000079460	4933403O08Rik	7.74E-02	-1.29E+01	-3.69	0.0074	-1.50E+02	1.50E+02
ENSMUSG00000105443	RP23-339I6.3	1.43E+01	1.43E+01	3.84	0.0092	1.49E+02	1.49E+02
ENSMUSG00000103824	Gm38177	1.85E-01	-5.41E+00	-2.44	0.0013	-1.49E+02	1.49E+02
ENSMUSG00000107256	RP24-297N9.13	2.45E+01	2.45E+01	4.61	0.0273	1.48E+02	1.48E+02
ENSMUSG00000088363	Gm23497	4.85E-02	-2.06E+01	-4.36	0.0196	-1.47E+02	1.47E+02
ENSMUSG00000097915	A330009N23Rik	1.48E-01	-6.76E+00	-2.76	0.0021	-1.46E+02	1.46E+02
ENSMUSG00000097268	Gm26805	1.05E+01	1.05E+01	3.39	0.0052	1.46E+02	1.46E+02
ENSMUSG00000009292	Trpm2	2.33E+00	2.33E+00	1.22	0.0003	1.45E+02	1.45E+02

ENSMUSG00000030017	Reg3g	4.95E+00	4.95E+00	2.31	0.0012	1.44E+02	1.44E+02
ENSMUSG00000103686	Gm37073	2.31E+01	2.31E+01	4.53	0.0257	1.44E+02	1.44E+02
ENSMUSG00000031394	Opn1mw	2.15E+01	2.15E+01	4.43	0.0225	1.44E+02	1.44E+02
ENSMUSG00000073380	Arrdc5	2.02E+01	2.02E+01	4.34	0.0198	1.43E+02	1.43E+02
ENSMUSG00000070436	Serpinh1	4.13E-01	-2.42E+00	-1.27	0.0003	-1.43E+02	1.43E+02
ENSMUSG00000039239	Tgfb2	3.77E-01	-2.65E+00	-1.41	0.0004	-1.41E+02	1.41E+02
ENSMUSG00000020875	Hoxb9	1.72E+01	1.72E+01	4.11	0.0152	1.40E+02	1.40E+02
ENSMUSG00000102715	Gm6209	3.82E+00	3.82E+00	1.93	0.0008	1.39E+02	1.39E+02
ENSMUSG00000015843	Rxrg	6.23E-02	-1.60E+01	-4.00	0.0139	-1.36E+02	1.36E+02
ENSMUSG00000072423	Psmb11	1.22E+01	1.22E+01	3.60	0.0081	1.35E+02	1.35E+02
ENSMUSG00000031870	Pgr	2.47E-01	-4.05E+00	-2.02	0.0009	-1.33E+02	1.33E+02
ENSMUSG00000085185	BC028777	3.51E+00	3.51E+00	1.81	0.0007	1.33E+02	1.33E+02
ENSMUSG00000084198	Gm13711	4.73E+00	4.73E+00	2.24	0.0013	1.32E+02	1.32E+02
ENSMUSG00000107037	RP24-544J6.3	2.43E+01	2.43E+01	4.60	0.0340	1.32E+02	1.32E+02
ENSMUSG00000088527	Gm26086	1.30E-01	-7.72E+00	-2.95	0.0035	-1.31E+02	1.31E+02
ENSMUSG00000010064	Slc38a3	2.09E+01	2.09E+01	4.39	0.0257	1.30E+02	1.30E+02
ENSMUSG00000001865	Cpa3	2.20E-01	-4.55E+00	-2.18	0.0012	-1.30E+02	1.30E+02
ENSMUSG00000103408	Gm37933	6.32E+00	6.32E+00	2.66	0.0024	1.30E+02	1.30E+02
ENSMUSG00000059639	Clec4a4	4.32E+00	4.32E+00	2.11	0.0011	1.30E+02	1.30E+02
ENSMUSG00000057173	Rfx8	2.54E+01	2.54E+01	4.67	0.0386	1.30E+02	1.30E+02
ENSMUSG00000025646	Atrip	3.49E+00	3.49E+00	1.80	0.0007	1.30E+02	1.30E+02
ENSMUSG00000030324	Rho	3.86E+00	3.86E+00	1.95	0.0009	1.29E+02	1.29E+02
ENSMUSG00000038074	Fkbp14	3.18E+00	3.18E+00	1.67	0.0006	1.29E+02	1.29E+02
ENSMUSG00000033644	Piwil2	3.17E+00	3.17E+00	1.67	0.0006	1.29E+02	1.29E+02
ENSMUSG00000086701	Gm13595	6.14E-02	-1.63E+01	-4.03	0.0161	-1.28E+02	1.28E+02
ENSMUSG00000046916	Myct1	2.15E-01	-4.66E+00	-2.22	0.0013	-1.27E+02	1.27E+02
ENSMUSG00000097430	Gm10544	2.02E-01	-4.95E+00	-2.31	0.0015	-1.27E+02	1.27E+02
ENSMUSG00000079163	Gm15498	1.83E+01	1.83E+01	4.19	0.0208	1.27E+02	1.27E+02
ENSMUSG00000034837	Gnat1	1.36E+01	1.36E+01	3.76	0.0115	1.26E+02	1.26E+02
ENSMUSG00000054958	Nt5c1a	1.66E-01	-6.03E+00	-2.59	0.0023	-1.25E+02	1.25E+02
ENSMUSG00000062329	Cytl1	2.67E-01	-3.75E+00	-1.91	0.0009	-1.25E+02	1.25E+02
ENSMUSG00000084011	Gm12969	8.77E+00	8.77E+00	3.13	0.0050	1.24E+02	1.24E+02
ENSMUSG00000102825	Gm36990	9.87E+00	9.87E+00	3.30	0.0064	1.24E+02	1.24E+02
ENSMUSG00000034818	Celf5	1.21E+01	1.21E+01	3.59	0.0095	1.23E+02	1.23E+02
ENSMUSG00000028332	Hemgn	2.04E+01	2.04E+01	4.35	0.0274	1.23E+02	1.23E+02
ENSMUSG00000073494	Sh2d1b2	2.06E+01	2.06E+01	4.37	0.0282	1.23E+02	1.23E+02
ENSMUSG00000091336	Gm17157	1.04E-01	-9.62E+00	-3.27	0.0062	-1.22E+02	1.22E+02
ENSMUSG00000031111	Igsf1	1.83E-01	-5.46E+00	-2.45	0.0020	-1.22E+02	1.22E+02
ENSMUSG00000008590	Htr3b	2.11E+01	2.11E+01	4.40	0.0301	1.21E+02	1.21E+02
ENSMUSG00000065582	Mir194-2	2.40E+01	2.40E+01	4.59	0.0402	1.20E+02	1.20E+02
ENSMUSG00000086526	Gm12762	8.75E+00	8.75E+00	3.13	0.0054	1.19E+02	1.19E+02
ENSMUSG00000038704	Aspdh	1.16E-01	-8.59E+00	-3.10	0.0052	-1.19E+02	1.19E+02
ENSMUSG00000077567	Gm23200	8.97E-02	-1.11E+01	-3.48	0.0090	-1.17E+02	1.17E+02
ENSMUSG00000040258	Nxph4	1.29E+01	1.29E+01	3.69	0.0121	1.17E+02	1.17E+02
ENSMUSG00000001027	Scn4a	1.51E+01	1.51E+01	3.91	0.0169	1.16E+02	1.16E+02
ENSMUSG00000036334	Igsf10	1.85E-01	-5.42E+00	-2.44	0.0022	-1.16E+02	1.16E+02
ENSMUSG00000086542	Gm13782	6.58E+00	6.58E+00	2.72	0.0033	1.15E+02	1.15E+02
ENSMUSG00000054258	Gm5082	5.94E+00	5.94E+00	2.57	0.0027	1.15E+02	1.15E+02
ENSMUSG00000101895	Gm28981	2.18E+01	2.18E+01	4.45	0.0359	1.15E+02	1.15E+02
ENSMUSG00000102414	Gm36938	2.28E+01	2.28E+01	4.51	0.0396	1.15E+02	1.15E+02
ENSMUSG00000043496	Tril	1.80E-01	-5.56E+00	-2.47	0.0024	-1.14E+02	1.14E+02
ENSMUSG00000040657	1700063H04Rik	1.65E+01	1.65E+01	4.05	0.0212	1.14E+02	1.14E+02
ENSMUSG00000006204	5430419D17Rik	8.86E-02	-1.13E+01	-3.50	0.0099	-1.13E+02	1.13E+02
ENSMUSG00000072679	D6Ertd474e	2.36E+01	2.36E+01	4.56	0.0434	1.13E+02	1.13E+02
ENSMUSG00000023247	Guca2a	2.29E+01	2.29E+01	4.52	0.0411	1.13E+02	1.13E+02
ENSMUSG00000001131	Timp1	5.68E+00	5.68E+00	2.51	0.0025	1.13E+02	1.13E+02
ENSMUSG00000031170	Slc38a5	1.74E-01	-5.75E+00	-2.52	0.0026	-1.12E+02	1.12E+02
ENSMUSG00000042985	Upk3b	2.47E-01	-4.05E+00	-2.02	0.0013	-1.12E+02	1.12E+02
ENSMUSG00000024421	Lama3	3.25E-01	-3.07E+00	-1.62	0.0008	-1.10E+02	1.10E+02
ENSMUSG00000087646	Gm1667	7.19E-02	-1.39E+01	-3.80	0.0160	-1.10E+02	1.10E+02
ENSMUSG00000081076	Rpsa-ps4	1.40E+01	1.40E+01	3.80	0.0161	1.10E+02	1.10E+02
ENSMUSG00000104237	Gm33533	1.40E+01	1.40E+01	3.80	0.0161	1.10E+02	1.10E+02
ENSMUSG00000101754	Gm2987	1.22E-01	-8.22E+00	-3.04	0.0056	-1.10E+02	1.10E+02
ENSMUSG00000045928	4933440M02Rik	4.89E+00	4.89E+00	2.29	0.0020	1.10E+02	1.10E+02
ENSMUSG00000040430	Pitpnc1	4.01E-01	-2.50E+00	-1.32	0.0005	-1.09E+02	1.09E+02
ENSMUSG00000104701	RP23-336O18.4	2.16E+01	2.16E+01	4.43	0.0400	1.08E+02	1.08E+02
ENSMUSG00000021902	Phf7	3.94E+00	3.94E+00	1.98	0.0013	1.08E+02	1.08E+02

ENSMUSG00000033872	Best4-ps	6.51E+00	6.51E+00	2.70	0.0037	1.08E+02	1.08E+02
ENSMUSG00000086904	Gm13404	6.94E-02	-1.44E+01	-3.85	0.0180	-1.07E+02	1.07E+02
ENSMUSG00000048126	Col6a3	1.99E-01	-5.01E+00	-2.33	0.0022	-1.07E+02	1.07E+02
ENSMUSG00000082570	Gm15711	3.11E-01	-3.22E+00	-1.69	0.0009	-1.07E+02	1.07E+02
ENSMUSG00000055865	Fam19a3	9.19E+00	9.19E+00	3.20	0.0074	1.07E+02	1.07E+02
ENSMUSG00000098608	Mir6981	4.78E-02	-2.09E+01	-4.39	0.0394	-1.06E+02	1.06E+02
ENSMUSG00000084947	Gm15594	2.03E-01	-4.93E+00	-2.30	0.0022	-1.05E+02	1.05E+02
ENSMUSG00000043770	Gm12481	4.84E-01	-2.07E+00	-1.05	0.0004	-1.04E+02	1.04E+02
ENSMUSG00000022103	Gfra2	1.58E-01	-6.35E+00	-2.67	0.0037	-1.04E+02	1.04E+02
ENSMUSG00000038691	Mbd3l1	8.96E+00	8.96E+00	3.16	0.0074	1.04E+02	1.04E+02
ENSMUSG00000091648	C2cd4d	1.16E-01	-8.62E+00	-3.11	0.0069	-1.04E+02	1.04E+02
ENSMUSG00000094686	Ccl21a	1.13E-01	-8.84E+00	-3.14	0.0073	-1.03E+02	1.03E+02
ENSMUSG00000084915	C230037L18Rik	6.01E+00	6.01E+00	2.59	0.0034	1.03E+02	1.03E+02
ENSMUSG00000066170	E230001N04Rik	8.55E+00	8.55E+00	3.10	0.0069	1.03E+02	1.03E+02
ENSMUSG00000097260	Gm5706	7.35E-02	-1.36E+01	-3.77	0.0175	-1.03E+02	1.03E+02
ENSMUSG00000070111	Gm10286	1.53E+01	1.53E+01	3.94	0.0223	1.03E+02	1.03E+02
ENSMUSG00000028862	Map3k6	4.35E+00	4.35E+00	2.12	0.0018	1.02E+02	1.02E+02
ENSMUSG00000030270	Cpne9	6.77E+00	6.77E+00	2.76	0.0044	1.02E+02	1.02E+02
ENSMUSG00000097298	A130057D12Rik	9.91E+00	9.91E+00	3.31	0.0095	1.02E+02	1.02E+02
ENSMUSG00000046881	Olfr374	7.12E+00	7.12E+00	2.83	0.0050	1.00E+02	1.00E+02
ENSMUSG00000062561	Gm10118	2.00E-01	-5.00E+00	-2.32	0.0025	-9.95E+01	9.95E+01
ENSMUSG00000100714	2010308F09Rik	9.93E+00	9.93E+00	3.31	0.0101	9.89E+01	9.89E+01
ENSMUSG00000101082	Gm28069	5.69E+00	5.69E+00	2.51	0.0034	9.81E+01	9.81E+01
ENSMUSG00000086709	Gm16263	1.08E-01	-9.22E+00	-3.21	0.0090	-9.70E+01	9.70E+01
ENSMUSG00000027500	Stmn2	1.76E-01	-5.68E+00	-2.51	0.0034	-9.69E+01	9.69E+01
ENSMUSG00000026592	Tex35	4.77E+00	4.77E+00	2.25	0.0024	9.66E+01	9.66E+01
ENSMUSG00000099240	Gm27539	7.88E-02	-1.27E+01	-3.67	0.0175	-9.60E+01	9.60E+01
ENSMUSG00000032357	Tinag	1.55E-01	-6.45E+00	-2.69	0.0046	-9.55E+01	9.55E+01
ENSMUSG00000051965	Nanos2	2.03E+01	2.03E+01	4.35	0.0455	9.54E+01	9.54E+01
ENSMUSG00000059434	Gckr	7.26E+00	7.26E+00	2.86	0.0058	9.54E+01	9.54E+01
ENSMUSG00000105403	RP24-267D17.1	2.40E-01	-4.16E+00	-2.06	0.0019	-9.48E+01	9.48E+01
ENSMUSG00000082816	Gm11953	1.75E+01	1.75E+01	4.13	0.0346	9.42E+01	9.42E+01
ENSMUSG00000094174	Ighv6-4	1.08E-01	-9.29E+00	-3.22	0.0101	-9.26E+01	9.26E+01
ENSMUSG00000059657	Stfa2l1	3.18E+00	3.18E+00	1.67	0.0012	9.24E+01	9.24E+01
ENSMUSG00000051177	Plcb1	2.47E-01	-4.04E+00	-2.02	0.0019	-9.23E+01	9.23E+01
ENSMUSG00000028068	Iqgap3	6.74E+00	6.74E+00	2.75	0.0054	9.16E+01	9.16E+01
ENSMUSG00000106518	RP23-178N8.3	3.55E+00	3.55E+00	1.83	0.0015	9.16E+01	9.16E+01
ENSMUSG00000085196	Gm14963	1.07E+01	1.07E+01	3.43	0.0140	9.08E+01	9.08E+01
ENSMUSG00000030781	Slc5a2	1.06E+01	1.06E+01	3.40	0.0138	9.00E+01	9.00E+01
ENSMUSG00000056271	Lman1l	7.55E+00	7.55E+00	2.92	0.0071	8.98E+01	8.98E+01
ENSMUSG00000051359	Ncald	2.51E-01	-3.98E+00	-1.99	0.0020	-8.91E+01	8.91E+01
ENSMUSG00000037411	Serpine1	5.08E+00	5.08E+00	2.35	0.0033	8.90E+01	8.90E+01
ENSMUSG00000098043	Gm26980	1.42E+01	1.42E+01	3.83	0.0260	8.82E+01	8.82E+01
ENSMUSG00000045381	Olfr433	5.62E-02	-1.78E+01	-4.15	0.0412	-8.77E+01	8.77E+01
ENSMUSG00000039883	Lrrc17	1.97E-01	-5.08E+00	-2.34	0.0034	-8.74E+01	8.74E+01
ENSMUSG00000082941	Gm12850	1.83E-01	-5.47E+00	-2.45	0.0039	-8.73E+01	8.73E+01
ENSMUSG00000086767	Gm13070	2.74E+00	2.74E+00	1.45	0.0010	8.73E+01	8.73E+01
ENSMUSG00000022586	Ly6i	5.62E+00	5.62E+00	2.49	0.0042	8.69E+01	8.69E+01
ENSMUSG00000057182	Scn3a	1.80E-01	-5.54E+00	-2.47	0.0042	-8.60E+01	8.60E+01
ENSMUSG00000021743	Fezf2	1.03E+01	1.03E+01	3.37	0.0145	8.59E+01	8.59E+01
ENSMUSG00000054003	Tdrd9	1.58E+01	1.58E+01	3.98	0.0340	8.57E+01	8.57E+01
ENSMUSG00000070337	Gpr179	3.01E+00	3.01E+00	1.59	0.0012	8.56E+01	8.56E+01
ENSMUSG00000074783	AU019990	1.41E+01	1.41E+01	3.82	0.0272	8.54E+01	8.54E+01
ENSMUSG00000086313	Gm15940	1.21E-01	-8.30E+00	-3.05	0.0095	-8.50E+01	8.50E+01
ENSMUSG00000023828	Slc22a3	2.90E-01	-3.45E+00	-1.79	0.0017	-8.49E+01	8.49E+01
ENSMUSG00000040405	Havcr1	1.93E-01	-5.19E+00	-2.38	0.0037	-8.49E+01	8.49E+01
ENSMUSG00000020123	Avpr1a	2.22E-01	-4.50E+00	-2.17	0.0028	-8.48E+01	8.48E+01
ENSMUSG00000028339	Col15a1	3.66E-01	-2.73E+00	-1.45	0.0010	-8.47E+01	8.47E+01
ENSMUSG00000074224	4932431P20Rik	1.73E+01	1.73E+01	4.11	0.0416	8.46E+01	8.46E+01
ENSMUSG00000073018	Gm1070	1.39E+01	1.39E+01	3.79	0.0271	8.41E+01	8.41E+01
ENSMUSG00000026175	Vil1	7.72E+00	7.72E+00	2.95	0.0084	8.41E+01	8.41E+01
ENSMUSG00000057719	Sh3rf2	3.70E-01	-2.70E+00	-1.43	0.0010	-8.36E+01	8.36E+01
ENSMUSG00000045008	9030612E09Rik	1.28E+01	1.28E+01	3.68	0.0238	8.33E+01	8.33E+01
ENSMUSG00000106838	RP24-556H22.2	7.60E+00	7.60E+00	2.93	0.0084	8.30E+01	8.30E+01
ENSMUSG00000030895	Hpx	2.28E+00	2.28E+00	1.19	0.0008	8.28E+01	8.28E+01
ENSMUSG00000097610	A930012L18Rik	2.66E-01	-3.76E+00	-1.91	0.0021	-8.23E+01	8.23E+01
ENSMUSG00000086283	2810433D01Rik	1.13E-01	-8.85E+00	-3.15	0.0116	-8.22E+01	8.22E+01

ENSMUSG00000076392	Gm25992	1.93E-01	-5.19E+00	-2.38	0.0040	-8.20E+01	8.20E+01
ENSMUSG00000033578	Tmem35	2.58E-01	-3.88E+00	-1.95	0.0023	-8.13E+01	8.13E+01
ENSMUSG00000004540	Psg17	5.25E+00	5.25E+00	2.39	0.0042	8.11E+01	8.11E+01
ENSMUSG00000092545	Gm20319	3.91E+00	3.91E+00	1.97	0.0023	8.11E+01	8.11E+01
ENSMUSG00000090643	Gm3453	9.85E+00	9.85E+00	3.30	0.0148	8.11E+01	8.11E+01
ENSMUSG00000085275	Gm14487	5.03E+00	5.03E+00	2.33	0.0039	8.08E+01	8.08E+01
ENSMUSG00000103642	Gm37769	1.35E-01	-7.43E+00	-2.89	0.0085	-8.08E+01	8.08E+01
ENSMUSG00000079258	Calhm1	9.85E+00	9.85E+00	3.30	0.0149	8.07E+01	8.07E+01
ENSMUSG00000023094	Msrb2	2.88E-01	-3.47E+00	-1.79	0.0019	-8.05E+01	8.05E+01
ENSMUSG00000000381	Wap	1.14E+01	1.14E+01	3.51	0.0201	8.01E+01	8.01E+01
ENSMUSG00000017754	Pltp	2.82E-01	-3.55E+00	-1.83	0.0020	-8.01E+01	8.01E+01
ENSMUSG00000048752	Prss50	6.13E+00	6.13E+00	2.62	0.0059	8.00E+01	8.00E+01
ENSMUSG00000104507	A430027H14Rik	1.94E-01	-5.17E+00	-2.37	0.0042	-7.99E+01	7.99E+01
ENSMUSG00000049281	Scn3b	3.25E-01	-3.08E+00	-1.62	0.0015	-7.98E+01	7.98E+01
ENSMUSG00000103043	Gm37306	1.29E-01	-7.77E+00	-2.96	0.0095	-7.97E+01	7.97E+01
ENSMUSG00000024222	Fkbp5	1.06E+01	1.06E+01	3.40	0.0177	7.96E+01	7.96E+01
ENSMUSG00000100303	2600014E21Rik	1.74E+01	1.74E+01	4.12	0.0480	7.95E+01	7.95E+01
ENSMUSG00000084316	Gm8668	8.24E-02	-1.21E+01	-3.60	0.0233	-7.94E+01	7.94E+01
ENSMUSG00000084890	A830036E02Rik	5.67E+00	5.67E+00	2.50	0.0052	7.89E+01	7.89E+01
ENSMUSG00000020150	Gamt	2.72E-01	-3.67E+00	-1.88	0.0022	-7.88E+01	7.88E+01
ENSMUSG00000079343	C1s2	4.98E+00	4.98E+00	2.32	0.0040	7.88E+01	7.88E+01
ENSMUSG00000060180	Myh13	1.77E-01	-5.64E+00	-2.50	0.0052	-7.86E+01	7.86E+01
ENSMUSG00000098052	Gm5300	1.27E+01	1.27E+01	3.67	0.0263	7.85E+01	7.85E+01
ENSMUSG00000067702	Tuba3a	1.66E+01	1.66E+01	4.06	0.0451	7.83E+01	7.83E+01
ENSMUSG00000053199	Arhgap20	2.93E-01	-3.41E+00	-1.77	0.0019	-7.80E+01	7.80E+01
ENSMUSG00000025380	Fscn2	1.08E+01	1.08E+01	3.44	0.0195	7.76E+01	7.76E+01
ENSMUSG00000086127	Gm11934	1.11E+01	1.11E+01	3.47	0.0206	7.73E+01	7.73E+01
ENSMUSG00000060187	Lrrc10	4.74E-01	-2.11E+00	-1.08	0.0007	-7.72E+01	7.72E+01
ENSMUSG00000080993	Gm12903	9.01E-02	-1.11E+01	-3.47	0.0208	-7.70E+01	7.70E+01
ENSMUSG00000092505	Gm19246	1.96E-01	-5.10E+00	-2.35	0.0044	-7.65E+01	7.65E+01
ENSMUSG00000102652	Gm37078	7.89E+00	7.89E+00	2.98	0.0107	7.65E+01	7.65E+01
ENSMUSG00000054423	Cadps	1.67E+01	1.67E+01	4.06	0.0478	7.62E+01	7.62E+01
ENSMUSG00000029517	Ankrd7	1.63E+01	1.63E+01	4.03	0.0462	7.59E+01	7.59E+01
ENSMUSG00000099464	Gm5260	2.24E-01	-4.46E+00	-2.16	0.0035	-7.55E+01	7.55E+01
ENSMUSG00000089730	1700007P06Rik	9.07E-02	-1.10E+01	-3.46	0.0213	-7.55E+01	7.55E+01
ENSMUSG00000075023	Accsl	1.64E+01	1.64E+01	4.04	0.0479	7.49E+01	7.49E+01
ENSMUSG00000085276	Gm15812	4.03E-01	-2.48E+00	-1.31	0.0011	-7.48E+01	7.48E+01
ENSMUSG00000055489	Ano5	1.61E-01	-6.19E+00	-2.63	0.0069	-7.47E+01	7.47E+01
ENSMUSG00000041479	Syt15	2.08E-01	-4.80E+00	-2.26	0.0042	-7.42E+01	7.42E+01
ENSMUSG00000084826	AI847159	4.36E+00	4.36E+00	2.13	0.0035	7.42E+01	7.42E+01
ENSMUSG00000026109	Tmeff2	2.91E-01	-3.44E+00	-1.78	0.0021	-7.42E+01	7.42E+01
ENSMUSG00000090222	Gm16340	9.21E-02	-1.09E+01	-3.44	0.0214	-7.42E+01	7.42E+01
ENSMUSG00000103040	Gm37304	9.06E-02	-1.10E+01	-3.46	0.0222	-7.41E+01	7.41E+01
ENSMUSG00000039691	Tspan10	9.84E+00	9.84E+00	3.30	0.0177	7.41E+01	7.41E+01
ENSMUSG00000040891	Foxa3	4.43E+00	4.43E+00	2.15	0.0036	7.38E+01	7.38E+01
ENSMUSG00000023951	Vegfa	3.53E-01	-2.83E+00	-1.50	0.0015	-7.38E+01	7.38E+01
ENSMUSG00000073094	Smim9	1.21E+01	1.21E+01	3.59	0.0270	7.35E+01	7.35E+01
ENSMUSG00000066687	Zbtb16	3.44E+00	3.44E+00	1.78	0.0022	7.33E+01	7.33E+01
ENSMUSG00000049796	Crh	5.08E+00	5.08E+00	2.35	0.0048	7.32E+01	7.32E+01
ENSMUSG00000020286	1700093K21Rik	1.63E+01	1.63E+01	4.03	0.0499	7.30E+01	7.30E+01
ENSMUSG00000070687	Htr1d	1.21E+01	1.21E+01	3.60	0.0276	7.28E+01	7.28E+01
ENSMUSG00000020469	Myl7	2.96E-01	-3.38E+00	-1.76	0.0022	-7.28E+01	7.28E+01
ENSMUSG00000104708	RP23-151C5.3	8.91E+00	8.91E+00	3.15	0.0151	7.24E+01	7.24E+01
ENSMUSG00000105873	RP23-49G7.2	1.41E-01	-7.11E+00	-2.83	0.0097	-7.23E+01	7.23E+01
ENSMUSG00000047161	Chst9	1.37E-01	-7.30E+00	-2.87	0.0103	-7.21E+01	7.21E+01
ENSMUSG00000056032	BC018473	1.59E+01	1.59E+01	3.99	0.0492	7.17E+01	7.17E+01
ENSMUSG00000078117	Gm16485	2.55E-01	-3.92E+00	-1.97	0.0030	-7.15E+01	7.15E+01
ENSMUSG00000063727	Tnfrsf11b	2.44E-01	-4.10E+00	-2.04	0.0033	-7.14E+01	7.14E+01
ENSMUSG00000081909	Gm11341	1.54E+01	1.54E+01	3.95	0.0471	7.11E+01	7.11E+01
ENSMUSG00000064194	Zfp936	1.54E+01	1.54E+01	3.95	0.0471	7.11E+01	7.11E+01
ENSMUSG00000087589	D430040D24Rik	3.41E+00	3.41E+00	1.77	0.0023	7.10E+01	7.10E+01
ENSMUSG00000092183	4930515G01Rik	7.61E+00	7.61E+00	2.93	0.0115	7.09E+01	7.09E+01
ENSMUSG00000041567	Serpina12	1.32E-01	-7.57E+00	-2.92	0.0114	-7.08E+01	7.08E+01
ENSMUSG00000079555	Haus3	2.48E+00	2.48E+00	1.31	0.0012	7.06E+01	7.06E+01
ENSMUSG00000009281	Rarres2	3.92E-01	-2.55E+00	-1.35	0.0013	-7.05E+01	7.05E+01
ENSMUSG00000106583	RP24-182A8.1	1.50E-01	-6.69E+00	-2.74	0.0090	-7.04E+01	7.04E+01
ENSMUSG00000102322	4930566N20Rik	1.34E-01	-7.49E+00	-2.91	0.0115	-6.99E+01	6.99E+01

ENSMUSG00000084686	Gm22027	1.01E-01	-9.90E+00	-3.31	0.0204	-6.93E+01	6.93E+01
ENSMUSG00000007279	Scube2	2.18E-01	-4.59E+00	-2.20	0.0044	-6.92E+01	6.92E+01
ENSMUSG00000001827	Folr1	5.98E+00	5.98E+00	2.58	0.0075	6.91E+01	6.91E+01
ENSMUSG00000038994	Hils1	1.39E+01	1.39E+01	3.80	0.0407	6.89E+01	6.89E+01
ENSMUSG00000097165	2210008F06Rik	9.09E+00	9.09E+00	3.18	0.0174	6.88E+01	6.88E+01
ENSMUSG00000078670	Fam174b	4.03E-01	-2.48E+00	-1.31	0.0013	-6.85E+01	6.85E+01
ENSMUSG00000059411	Olfr434	9.21E+00	9.21E+00	3.20	0.0181	6.85E+01	6.85E+01
ENSMUSG00000035283	Adrb1	3.68E-01	-2.72E+00	-1.44	0.0016	-6.85E+01	6.85E+01
ENSMUSG00000083518	Gm9429	7.35E+00	7.35E+00	2.88	0.0115	6.84E+01	6.84E+01
ENSMUSG00000097015	Gm26705	1.83E-01	-5.45E+00	-2.45	0.0064	-6.83E+01	6.83E+01
ENSMUSG00000030523	Trpm1	7.91E+00	7.91E+00	2.98	0.0134	6.82E+01	6.82E+01
ENSMUSG00000052934	Fbxo31	2.14E+00	2.14E+00	1.10	0.0010	6.81E+01	6.81E+01
ENSMUSG00000097465	Gm16619	3.71E+00	3.71E+00	1.89	0.0030	6.80E+01	6.80E+01
ENSMUSG00000105402	RP24-448C16.7	2.20E-01	-4.54E+00	-2.18	0.0045	-6.78E+01	6.78E+01
ENSMUSG00000107045	RP23-387P23.4	6.60E+00	6.60E+00	2.72	0.0095	6.77E+01	6.77E+01
ENSMUSG00000069890	1700024J04Rik	5.03E+00	5.03E+00	2.33	0.0056	6.72E+01	6.72E+01
ENSMUSG00000097766	5730420D15Rik	2.27E+00	2.27E+00	1.18	0.0011	6.72E+01	6.72E+01
ENSMUSG00000056155	Nanos3	1.15E+01	1.15E+01	3.53	0.0295	6.71E+01	6.71E+01
ENSMUSG00000075427	Olfr288	1.07E+01	1.07E+01	3.42	0.0256	6.67E+01	6.67E+01
ENSMUSG00000103012	Gm37548	1.10E-01	-9.12E+00	-3.19	0.0187	-6.67E+01	6.67E+01
ENSMUSG00000106247	Gpc2	4.91E+00	4.91E+00	2.30	0.0054	6.66E+01	6.66E+01
ENSMUSG00000070504	Fcrl6	4.72E+00	4.72E+00	2.24	0.0050	6.65E+01	6.65E+01
ENSMUSG00000021509	Slc25a48	1.42E+01	1.42E+01	3.83	0.0457	6.65E+01	6.65E+01
ENSMUSG00000087202	Gm15813	4.07E-01	-2.46E+00	-1.30	0.0014	-6.64E+01	6.64E+01
ENSMUSG00000106840	WI1-535J9.1	5.84E+00	5.84E+00	2.55	0.0077	6.63E+01	6.63E+01
ENSMUSG00000091511	Vmn2r87	1.31E-01	-7.62E+00	-2.93	0.0133	-6.61E+01	6.61E+01
ENSMUSG00000048814	Lonrf2	2.62E-01	-3.82E+00	-1.93	0.0034	-6.59E+01	6.59E+01
ENSMUSG00000083289	Gm8812	2.57E+00	2.57E+00	1.36	0.0015	6.58E+01	6.58E+01
ENSMUSG00000100510	AV026068	2.04E-01	-4.90E+00	-2.29	0.0055	-6.57E+01	6.57E+01
ENSMUSG00000048582	Gja3	1.35E-01	-7.41E+00	-2.89	0.0127	-6.57E+01	6.57E+01
ENSMUSG00000020642	Rnf144a	2.44E-01	-4.10E+00	-2.04	0.0039	-6.56E+01	6.56E+01
ENSMUSG00000098575	Gm27446	2.00E-01	-4.99E+00	-2.32	0.0058	-6.55E+01	6.55E+01
ENSMUSG00000011751	Sptbn4	7.09E+00	7.09E+00	2.82	0.0117	6.54E+01	6.54E+01
ENSMUSG00000036158	Prickle1	3.00E-01	-3.34E+00	-1.74	0.0026	-6.54E+01	6.54E+01
ENSMUSG00000087452	Gm11998	1.42E-01	-7.05E+00	-2.82	0.0117	-6.52E+01	6.52E+01
ENSMUSG00000097360	9430065F17Rik	1.89E-01	-5.29E+00	-2.40	0.0066	-6.52E+01	6.52E+01
ENSMUSG00000044320	1700001O22Rik	2.82E+00	2.82E+00	1.50	0.0019	6.50E+01	6.50E+01
ENSMUSG00000023914	Mep1a	1.30E+01	1.30E+01	3.70	0.0402	6.46E+01	6.46E+01
ENSMUSG00000028718	Stil	3.82E+00	3.82E+00	1.93	0.0035	6.42E+01	6.42E+01
ENSMUSG00000105391	RP23-298M23.3	4.00E+00	4.00E+00	2.00	0.0039	6.36E+01	6.36E+01
ENSMUSG00000016262	Sertad4	3.45E-01	-2.90E+00	-1.53	0.0021	-6.34E+01	6.34E+01
ENSMUSG00000051788	4930564D02Rik	1.37E+01	1.37E+01	3.78	0.0471	6.32E+01	6.32E+01
ENSMUSG00000084061	Gm4996	7.40E-02	-1.35E+01	-3.76	0.0458	-6.31E+01	6.31E+01
ENSMUSG00000106220	RP24-488I7.1	2.70E-01	-3.70E+00	-1.89	0.0035	-6.28E+01	6.28E+01
ENSMUSG00000086947	4930522O17Rik	1.30E-01	-7.70E+00	-2.94	0.0151	-6.27E+01	6.27E+01
ENSMUSG00000016552	Foxred2	3.32E+00	3.32E+00	1.73	0.0028	6.26E+01	6.26E+01
ENSMUSG00000076431	Sox4	3.80E-01	-2.63E+00	-1.39	0.0018	-6.23E+01	6.23E+01
ENSMUSG00000101476	Gm29570	8.38E+00	8.38E+00	3.07	0.0184	6.19E+01	6.19E+01
ENSMUSG00000029695	Aass	2.22E-01	-4.50E+00	-2.17	0.0053	-6.18E+01	6.18E+01
ENSMUSG00000015665	Awat1	8.47E+00	8.47E+00	3.08	0.0188	6.18E+01	6.18E+01
ENSMUSG00000074217	2210011C24Rik	3.18E-01	-3.14E+00	-1.65	0.0026	-6.17E+01	6.17E+01
ENSMUSG00000029669	Tspan12	3.69E-01	-2.71E+00	-1.44	0.0019	-6.17E+01	6.17E+01
ENSMUSG00000089352	Gm25489	1.48E-01	-6.75E+00	-2.76	0.0120	-6.17E+01	6.17E+01
ENSMUSG00000091457	Gm17171	2.11E-01	-4.73E+00	-2.24	0.0059	-6.17E+01	6.17E+01
ENSMUSG00000054422	Fabp1	1.62E-01	-6.17E+00	-2.63	0.0100	-6.17E+01	6.17E+01
ENSMUSG00000020916	Krt36	1.48E-01	-6.76E+00	-2.76	0.0120	-6.16E+01	6.16E+01
ENSMUSG00000024440	Pcdh12	2.47E-01	-4.04E+00	-2.02	0.0044	-6.12E+01	6.12E+01
ENSMUSG00000041708	Mpped1	3.34E+00	3.34E+00	1.74	0.0030	6.11E+01	6.11E+01
ENSMUSG00000067949	Vmn1r-ps144	8.53E+00	8.53E+00	3.09	0.0197	6.08E+01	6.08E+01
ENSMUSG00000015354	Pcolce2	3.20E-01	-3.12E+00	-1.64	0.0026	-6.07E+01	6.07E+01
ENSMUSG00000044624	Gm4922	2.11E-01	-4.73E+00	-2.24	0.0061	-6.06E+01	6.06E+01
ENSMUSG00000061974	Cldn24	8.33E+00	8.33E+00	3.06	0.0190	6.05E+01	6.05E+01
ENSMUSG00000055333	Fat2	3.13E-01	-3.20E+00	-1.68	0.0028	-6.04E+01	6.04E+01
ENSMUSG00000104519	Gm37161	2.70E-01	-3.70E+00	-1.89	0.0038	-6.01E+01	6.01E+01
ENSMUSG00000007944	Ttc9b	6.70E+00	6.70E+00	2.74	0.0124	6.00E+01	6.00E+01
ENSMUSG00000054196	Cthrc1	2.33E-01	-4.29E+00	-2.10	0.0051	-5.98E+01	5.98E+01
ENSMUSG00000092171	4833427F10Rik	3.67E+00	3.67E+00	1.87	0.0038	5.97E+01	5.97E+01

ENSMUSG00000053441	Adamts19	2.00E-01	-5.00E+00	-2.32	0.0071	-5.95E+01	5.95E+01
ENSMUSG00000101505	1700109G14Rik	6.11E+00	6.11E+00	2.61	0.0106	5.94E+01	5.94E+01
ENSMUSG00000088171	Gm25220	1.47E-01	-6.78E+00	-2.76	0.0132	-5.91E+01	5.91E+01
ENSMUSG00000098130	Gm17797	2.85E-01	-3.51E+00	-1.81	0.0035	-5.90E+01	5.90E+01
ENSMUSG00000038233	Fam198a	2.03E-01	-4.93E+00	-2.30	0.0070	-5.90E+01	5.90E+01
ENSMUSG00000104882	RP23-397C5.5	4.72E-01	-2.12E+00	-1.08	0.0013	-5.90E+01	5.90E+01
ENSMUSG00000081106	Gm7155	1.27E-01	-7.88E+00	-2.98	0.0179	-5.89E+01	5.89E+01
ENSMUSG00000027209	Fam227b	2.58E+00	2.58E+00	1.37	0.0019	5.88E+01	5.88E+01
ENSMUSG00000075480	Gm10840	8.80E+00	8.80E+00	3.14	0.0225	5.86E+01	5.86E+01
ENSMUSG00000036574	1700019O17Rik	4.68E+00	4.68E+00	2.23	0.0064	5.86E+01	5.86E+01
ENSMUSG00000028794	A3galt2	6.05E+00	6.05E+00	2.60	0.0107	5.85E+01	5.85E+01
ENSMUSG00000097847	4930478K11Rik	5.03E+00	5.03E+00	2.33	0.0074	5.85E+01	5.85E+01
ENSMUSG00000063060	Sox7	2.99E-01	-3.35E+00	-1.74	0.0033	-5.81E+01	5.81E+01
ENSMUSG00000026616	Cr2	4.36E-01	-2.30E+00	-1.20	0.0016	-5.81E+01	5.81E+01
ENSMUSG00000086405	9330198N18Rik	7.88E+00	7.88E+00	2.98	0.0187	5.77E+01	5.77E+01
ENSMUSG00000103061	Gm36970	1.36E-01	-7.35E+00	-2.88	0.0162	-5.77E+01	5.77E+01
ENSMUSG00000039178	Tbc1d19	4.72E-01	-2.12E+00	-1.08	0.0014	-5.74E+01	5.74E+01
ENSMUSG00000081636	Gm15901	1.46E-01	-6.87E+00	-2.78	0.0143	-5.74E+01	5.74E+01
ENSMUSG00000031881	Cdh16	2.93E-01	-3.41E+00	-1.77	0.0036	-5.71E+01	5.71E+01
ENSMUSG00000082767	Gm11622	1.06E+01	1.06E+01	3.41	0.0351	5.66E+01	5.66E+01
ENSMUSG00000097292	A230107N01Rik	1.74E-01	-5.74E+00	-2.52	0.0103	-5.65E+01	5.65E+01
ENSMUSG00000033350	Chst2	3.38E-01	-2.96E+00	-1.57	0.0028	-5.62E+01	5.62E+01
ENSMUSG00000035576	L3mbtl1	7.78E+00	7.78E+00	2.96	0.0193	5.61E+01	5.61E+01
ENSMUSG00000055555	4930502E18Rik	2.23E-01	-4.48E+00	-2.16	0.0064	-5.60E+01	5.60E+01
ENSMUSG00000085843	Kank4os	1.71E-01	-5.83E+00	-2.54	0.0109	-5.59E+01	5.59E+01
ENSMUSG00000107252	RP24-183G19.6	5.45E+00	5.45E+00	2.45	0.0096	5.57E+01	5.57E+01
ENSMUSG00000105251	RP23-195B3.3	1.13E+01	1.13E+01	3.50	0.0414	5.57E+01	5.57E+01
ENSMUSG00000103015	Gm37549	3.92E+00	3.92E+00	1.97	0.0051	5.51E+01	5.51E+01
ENSMUSG00000068745	Mybphl	1.52E-01	-6.57E+00	-2.72	0.0145	-5.46E+01	5.46E+01
ENSMUSG00000043289	Mei4	1.55E-01	-6.46E+00	-2.69	0.0142	-5.42E+01	5.42E+01
ENSMUSG00000045545	Krt14	1.46E-01	-6.84E+00	-2.77	0.0160	-5.41E+01	5.41E+01
ENSMUSG00000105203	RP24-255E8.1	4.35E+00	4.35E+00	2.12	0.0065	5.39E+01	5.39E+01
ENSMUSG00000099192	Gm27373	1.13E-01	-8.87E+00	-3.15	0.0272	-5.38E+01	5.38E+01
ENSMUSG00000053030	Spink2	2.96E-01	-3.38E+00	-1.76	0.0040	-5.38E+01	5.38E+01
ENSMUSG00000086265	Marcksl1-ps4	2.14E-01	-4.67E+00	-2.22	0.0075	-5.38E+01	5.38E+01
ENSMUSG00000073293	Nudt10	3.79E-01	-2.64E+00	-1.40	0.0024	-5.38E+01	5.38E+01
ENSMUSG00000058806	Col13a1	4.96E-01	-2.02E+00	-1.01	0.0014	-5.37E+01	5.37E+01
ENSMUSG00000000416	Cttnbp2	2.32E-01	-4.30E+00	-2.11	0.0065	-5.36E+01	5.36E+01
ENSMUSG00000102207	Gm10344	7.80E+00	7.80E+00	2.96	0.0212	5.36E+01	5.36E+01
ENSMUSG00000046491	C1qtnf2	2.47E-01	-4.04E+00	-2.02	0.0057	-5.34E+01	5.34E+01
ENSMUSG00000029636	Wasf3	4.56E-01	-2.19E+00	-1.13	0.0017	-5.34E+01	5.34E+01
ENSMUSG00000054690	Emcn	3.13E-01	-3.19E+00	-1.68	0.0036	-5.34E+01	5.34E+01
ENSMUSG00000073295	Nudt11	4.54E-01	-2.20E+00	-1.14	0.0017	-5.30E+01	5.30E+01
ENSMUSG00000074218	Cox7a1	4.10E-01	-2.44E+00	-1.29	0.0021	-5.30E+01	5.30E+01
ENSMUSG00000027597	Ahcy	2.32E+00	2.32E+00	1.21	0.0019	5.27E+01	5.27E+01
ENSMUSG00000086791	Gm12147	1.05E+01	1.05E+01	3.40	0.0400	5.27E+01	5.27E+01
ENSMUSG00000037492	Zmat4	1.38E-01	-7.27E+00	-2.86	0.0195	-5.21E+01	5.21E+01
ENSMUSG00000105212	RP23-286C5.2	2.78E-01	-3.59E+00	-1.84	0.0048	-5.21E+01	5.21E+01
ENSMUSG00000046793	Gpr61	4.95E+00	4.95E+00	2.31	0.0091	5.19E+01	5.19E+01
ENSMUSG00000036144	Meox2	2.64E-01	-3.79E+00	-1.92	0.0054	-5.16E+01	5.16E+01
ENSMUSG00000019831	Wasf1	1.58E-01	-6.35E+00	-2.67	0.0151	-5.16E+01	5.16E+01
ENSMUSG00000028011	Tdo2	3.97E+00	3.97E+00	1.99	0.0059	5.15E+01	5.15E+01
ENSMUSG00000089874	9230117E06Rik	2.55E-01	-3.92E+00	-1.97	0.0058	-5.14E+01	5.14E+01
ENSMUSG00000039304	Tnfsf10	2.35E-01	-4.25E+00	-2.09	0.0069	-5.13E+01	5.13E+01
ENSMUSG00000027612	Mmp24	5.18E+00	5.18E+00	2.37	0.0102	5.13E+01	5.13E+01
ENSMUSG00000105130	RP23-434H17.5	2.49E+00	2.49E+00	1.31	0.0024	5.11E+01	5.11E+01
ENSMUSG00000100318	Gm18799	1.10E-01	-9.08E+00	-3.18	0.0315	-5.11E+01	5.11E+01
ENSMUSG00000001802	Lrp3	3.19E-01	-3.13E+00	-1.65	0.0038	-5.10E+01	5.10E+01
ENSMUSG00000091014	Gm5244	1.01E+01	1.01E+01	3.33	0.0394	5.09E+01	5.09E+01
ENSMUSG00000086765	Gm11827	7.39E+00	7.39E+00	2.89	0.0211	5.08E+01	5.08E+01
ENSMUSG00000089635	Gm16559	2.87E-01	-3.48E+00	-1.80	0.0047	-5.08E+01	5.08E+01
ENSMUSG00000020877	Scrn2	3.31E-01	-3.02E+00	-1.59	0.0035	-5.08E+01	5.08E+01
ENSMUSG00000107281	AC113302.1	7.33E+00	7.33E+00	2.87	0.0210	5.05E+01	5.05E+01
ENSMUSG00000099422	Gm4275	9.93E+00	9.93E+00	3.31	0.0388	5.04E+01	5.04E+01
ENSMUSG00000097789	Gm2115	3.62E-01	-2.76E+00	-1.47	0.0030	-5.03E+01	5.03E+01
ENSMUSG00000023336	Wfdc1	3.79E-01	-2.64E+00	-1.40	0.0028	-5.03E+01	5.03E+01
ENSMUSG00000060548	Tnfrsf19	3.23E-01	-3.10E+00	-1.63	0.0038	-5.03E+01	5.03E+01

ENSMUSG00000085329	2810404F17Rik	1.04E+01	1.04E+01	3.38	0.0429	5.02E+01	5.02E+01
ENSMUSG00000105401	RP23-239A13.2	7.57E+00	7.57E+00	2.92	0.0228	5.01E+01	5.01E+01
ENSMUSG00000038583	Pln	3.65E-01	-2.74E+00	-1.45	0.0030	-5.00E+01	5.00E+01
ENSMUSG00000085982	9530051G07Rik	1.72E-01	-5.81E+00	-2.54	0.0135	-4.99E+01	4.99E+01
ENSMUSG00000084595	n-R5s188	2.00E-01	-5.01E+00	-2.32	0.0101	-4.98E+01	4.98E+01
ENSMUSG00000033006	Sox10	3.17E+00	3.17E+00	1.66	0.0040	4.98E+01	4.98E+01
ENSMUSG00000071317	Bves	2.73E-01	-3.66E+00	-1.87	0.0054	-4.98E+01	4.98E+01
ENSMUSG00000025856	Pdgfa	4.32E-01	-2.32E+00	-1.21	0.0022	-4.98E+01	4.98E+01
ENSMUSG00000084674	Gm26027	2.92E-01	-3.42E+00	-1.78	0.0047	-4.97E+01	4.97E+01
ENSMUSG00000031841	Cdh13	4.15E-01	-2.41E+00	-1.27	0.0024	-4.96E+01	4.96E+01
ENSMUSG00000097050	Gm9918	6.06E+00	6.06E+00	2.60	0.0150	4.95E+01	4.95E+01
ENSMUSG00000074467	Gm10702	4.16E+00	4.16E+00	2.06	0.0071	4.94E+01	4.94E+01
ENSMUSG00000087192	4930412L05Rik	9.13E+00	9.13E+00	3.19	0.0341	4.94E+01	4.94E+01
ENSMUSG00000082800	Gm13560	1.06E-01	-9.46E+00	-3.24	0.0367	-4.94E+01	4.94E+01
ENSMUSG00000081957	Ak3l2-ps	3.29E+00	3.29E+00	1.72	0.0044	4.94E+01	4.94E+01
ENSMUSG00000028047	Thbs3	3.33E-01	-3.00E+00	-1.59	0.0037	-4.93E+01	4.93E+01
ENSMUSG00000059921	Unc5c	4.03E-01	-2.48E+00	-1.31	0.0026	-4.91E+01	4.91E+01
ENSMUSG00000098153	Gm27024	6.57E+00	6.57E+00	2.72	0.0180	4.89E+01	4.89E+01
ENSMUSG00000086666	Gm12249	9.94E+00	9.94E+00	3.31	0.0417	4.87E+01	4.87E+01
ENSMUSG00000011632	Pinlyp	9.94E+00	9.94E+00	3.31	0.0417	4.87E+01	4.87E+01
ENSMUSG00000010342	Tex14	3.59E+00	3.59E+00	1.84	0.0055	4.85E+01	4.85E+01
ENSMUSG00000038295	Atg9b	9.40E+00	9.40E+00	3.23	0.0377	4.84E+01	4.84E+01
ENSMUSG00000054409	Tmem74	9.58E-02	-1.04E+01	-3.38	0.0466	-4.84E+01	4.84E+01
ENSMUSG00000100782	Gm28231	1.37E-01	-7.32E+00	-2.87	0.0230	-4.83E+01	4.83E+01
ENSMUSG00000058656	Samd12	1.74E-01	-5.76E+00	-2.53	0.0142	-4.83E+01	4.83E+01
ENSMUSG00000033278	Ptprm	4.80E-01	-2.08E+00	-1.06	0.0019	-4.82E+01	4.82E+01
ENSMUSG00000048385	Scrt1	1.05E+01	1.05E+01	3.39	0.0472	4.82E+01	4.82E+01
ENSMUSG00000051504	Siglech	2.29E-01	-4.37E+00	-2.13	0.0083	-4.81E+01	4.81E+01
ENSMUSG00000097403	9230116N13Rik	2.19E+00	2.19E+00	1.13	0.0021	4.80E+01	4.80E+01
ENSMUSG00000032099	Pate4	1.07E+01	1.07E+01	3.41	0.0497	4.78E+01	4.78E+01
ENSMUSG00000040432	Ltb4r2	1.03E+01	1.03E+01	3.37	0.0467	4.77E+01	4.77E+01
ENSMUSG00000029735	Tpk1	4.89E-01	-2.04E+00	-1.03	0.0018	-4.76E+01	4.76E+01
ENSMUSG00000050395	Tnfsf15	3.05E-01	-3.27E+00	-1.71	0.0047	-4.75E+01	4.75E+01
ENSMUSG00000097087	Gm26878	3.42E-01	-2.93E+00	-1.55	0.0038	-4.75E+01	4.75E+01
ENSMUSG00000056148	Rdh9	5.71E+00	5.71E+00	2.51	0.0146	4.72E+01	4.72E+01
ENSMUSG00000032128	Robo3	4.45E+00	4.45E+00	2.16	0.0089	4.71E+01	4.71E+01
ENSMUSG00000104765	RP23-443N7.10	3.11E+00	3.11E+00	1.64	0.0044	4.71E+01	4.71E+01
ENSMUSG00000028217	Cdh17	2.81E+00	2.81E+00	1.49	0.0036	4.70E+01	4.70E+01
ENSMUSG00000028972	Car6	8.78E+00	8.78E+00	3.13	0.0350	4.69E+01	4.69E+01
ENSMUSG00000106032	RP23-298M23.7	2.99E-01	-3.34E+00	-1.74	0.0051	-4.69E+01	4.69E+01
ENSMUSG00000045680	Tcf21	3.18E-01	-3.15E+00	-1.65	0.0045	-4.67E+01	4.67E+01
ENSMUSG00000068154	Insm1	7.34E+00	7.34E+00	2.88	0.0248	4.66E+01	4.66E+01
ENSMUSG00000084789	Gm12974	5.97E+00	5.97E+00	2.58	0.0165	4.65E+01	4.65E+01
ENSMUSG00000090843	Gm17673	4.97E+00	4.97E+00	2.31	0.0114	4.65E+01	4.65E+01
ENSMUSG00000057337	Chst3	3.63E-01	-2.75E+00	-1.46	0.0035	-4.64E+01	4.64E+01
ENSMUSG00000085434	Gm11725	4.77E+00	4.77E+00	2.25	0.0106	4.62E+01	4.62E+01
ENSMUSG00000106444	RP23-247I11.7	2.29E-01	-4.36E+00	-2.12	0.0089	-4.62E+01	4.62E+01
ENSMUSG00000093650	Gm20631	2.07E-01	-4.83E+00	-2.27	0.0110	-4.61E+01	4.61E+01
ENSMUSG00000019865	Nmbr	1.64E-01	-6.11E+00	-2.61	0.0176	-4.61E+01	4.61E+01
ENSMUSG00000091618	H60c	1.93E-01	-5.18E+00	-2.37	0.0126	-4.61E+01	4.61E+01
ENSMUSG00000036782	Klhl13	3.88E-01	-2.58E+00	-1.37	0.0031	-4.60E+01	4.60E+01
ENSMUSG00000081250	Gm15554	1.55E-01	-6.45E+00	-2.69	0.0196	-4.60E+01	4.60E+01
ENSMUSG00000015134	Aldh1a3	2.29E+00	2.29E+00	1.20	0.0025	4.59E+01	4.59E+01
ENSMUSG00000027977	Ndst3	9.06E+00	9.06E+00	3.18	0.0390	4.58E+01	4.58E+01
ENSMUSG00000087200	Lrp8os3	1.38E-01	-7.26E+00	-2.86	0.0254	-4.56E+01	4.56E+01
ENSMUSG00000100158	Gm28119	1.66E-01	-6.03E+00	-2.59	0.0176	-4.54E+01	4.54E+01
ENSMUSG00000099693	Gm29284	2.00E+00	2.00E+00	1.00	0.0020	4.52E+01	4.52E+01
ENSMUSG00000098143	Gm26937	1.14E-01	-8.76E+00	-3.13	0.0376	-4.52E+01	4.52E+01
ENSMUSG00000035785	Cmtm2b	6.01E+00	6.01E+00	2.59	0.0179	4.49E+01	4.49E+01
ENSMUSG00000092035	Peg10	2.35E+00	2.35E+00	1.23	0.0028	4.48E+01	4.48E+01
ENSMUSG00000068522	Aard	2.38E-01	-4.20E+00	-2.07	0.0089	-4.46E+01	4.46E+01
ENSMUSG00000031494	Cd209a	2.72E-01	-3.67E+00	-1.88	0.0068	-4.46E+01	4.46E+01
ENSMUSG00000002633	Shh	4.59E-01	-2.18E+00	-1.12	0.0024	-4.44E+01	4.44E+01
ENSMUSG00000038044	Cct8l1	3.03E+00	3.03E+00	1.60	0.0047	4.41E+01	4.41E+01
ENSMUSG00000001774	Chordc1	4.63E-01	-2.16E+00	-1.11	0.0024	-4.41E+01	4.41E+01
ENSMUSG00000099182	Gm8676	6.01E+00	6.01E+00	2.59	0.0190	4.36E+01	4.36E+01
ENSMUSG00000106845	RP23-270L23.1	6.67E+00	6.67E+00	2.74	0.0236	4.35E+01	4.35E+01

ENSMUSG00000107029	RP23-423A22.3	7.98E+00	7.98E+00	3.00	0.0339	4.33E+01	4.33E+01
ENSMUSG00000050505	Pcdh20	4.13E+00	4.13E+00	2.05	0.0092	4.32E+01	4.32E+01
ENSMUSG00000076666	Ighv14-4	7.81E+00	7.81E+00	2.97	0.0328	4.32E+01	4.32E+01
ENSMUSG00000100890	1700085C21Rik	2.28E-01	-4.39E+00	-2.14	0.0104	-4.31E+01	4.31E+01
ENSMUSG00000097816	Gm26811	4.05E-01	-2.47E+00	-1.30	0.0033	-4.31E+01	4.31E+01
ENSMUSG00000104279	Gm38300	5.23E+00	5.23E+00	2.39	0.0148	4.31E+01	4.31E+01
ENSMUSG00000027249	F2	7.11E+00	7.11E+00	2.83	0.0273	4.31E+01	4.31E+01
ENSMUSG00000027217	Tspan18	2.73E-01	-3.66E+00	-1.87	0.0073	-4.30E+01	4.30E+01
ENSMUSG00000067338	Tuba3b	4.23E+00	4.23E+00	2.08	0.0097	4.29E+01	4.29E+01
ENSMUSG00000104222	Gm7292	6.63E+00	6.63E+00	2.73	0.0241	4.27E+01	4.27E+01
ENSMUSG00000022099	Dmtn	3.85E-01	-2.60E+00	-1.38	0.0037	-4.27E+01	4.27E+01
ENSMUSG00000095895	Gm21963	1.67E-01	-6.00E+00	-2.58	0.0198	-4.26E+01	4.26E+01
ENSMUSG00000025473	Adam8	2.33E+00	2.33E+00	1.22	0.0030	4.26E+01	4.26E+01
ENSMUSG00000103532	Gm4430	3.15E+00	3.15E+00	1.66	0.0055	4.25E+01	4.25E+01
ENSMUSG00000036853	Mcoln3	2.53E-01	-3.95E+00	-1.98	0.0086	-4.25E+01	4.25E+01
ENSMUSG00000022445	Cyp2d26	4.59E+00	4.59E+00	2.20	0.0117	4.25E+01	4.25E+01
ENSMUSG00000061702	Tmem91	6.68E+00	6.68E+00	2.74	0.0248	4.24E+01	4.24E+01
ENSMUSG00000027224	Duoxa1	7.85E+00	7.85E+00	2.97	0.0343	4.24E+01	4.24E+01
ENSMUSG00000021364	Elovl2	4.03E+00	4.03E+00	2.01	0.0091	4.23E+01	4.23E+01
ENSMUSG00000097187	Gm19426	3.39E-01	-2.95E+00	-1.56	0.0049	-4.22E+01	4.22E+01
ENSMUSG00000072584	Gm7489	1.81E-01	-5.53E+00	-2.47	0.0173	-4.20E+01	4.20E+01
ENSMUSG00000092085	Gm17224	7.90E+00	7.90E+00	2.98	0.0357	4.19E+01	4.19E+01
ENSMUSG00000102697	Pcdhac2	1.90E-01	-5.28E+00	-2.40	0.0159	-4.18E+01	4.18E+01
ENSMUSG00000082907	Gm14832	1.80E-01	-5.55E+00	-2.47	0.0176	-4.18E+01	4.18E+01
ENSMUSG00000028031	Dkk2	1.93E-01	-5.17E+00	-2.37	0.0154	-4.17E+01	4.17E+01
ENSMUSG00000063767	S100a7a	1.66E-01	-6.02E+00	-2.59	0.0209	-4.16E+01	4.16E+01
ENSMUSG00000025037	Maoa	4.14E-01	-2.41E+00	-1.27	0.0034	-4.16E+01	4.16E+01
ENSMUSG00000027932	Slc27a3	3.29E-01	-3.04E+00	-1.60	0.0053	-4.16E+01	4.16E+01
ENSMUSG00000105096	Gbp10	4.25E-01	-2.36E+00	-1.24	0.0032	-4.14E+01	4.14E+01
ENSMUSG00000027070	Lrp2	3.01E-01	-3.32E+00	-1.73	0.0065	-4.14E+01	4.14E+01
ENSMUSG00000058402	Zfp420	4.64E-01	-2.15E+00	-1.11	0.0027	-4.13E+01	4.13E+01
ENSMUSG00000104745	RP23-394K8.3	2.27E-01	-4.41E+00	-2.14	0.0114	-4.13E+01	4.13E+01
ENSMUSG00000099803	Gm28863	7.12E+00	7.12E+00	2.83	0.0297	4.13E+01	4.13E+01
ENSMUSG00000022596	Slurp1	2.66E-01	-3.76E+00	-1.91	0.0083	-4.13E+01	4.13E+01
ENSMUSG00000081597	Gm14506	8.06E+00	8.06E+00	3.01	0.0382	4.12E+01	4.12E+01
ENSMUSG00000049097	Ankrd34a	3.53E+00	3.53E+00	1.82	0.0073	4.12E+01	4.12E+01
ENSMUSG00000053360	F730035P03Rik	6.15E+00	6.15E+00	2.62	0.0223	4.12E+01	4.12E+01
ENSMUSG00000041592	Sdk2	4.46E+00	4.46E+00	2.16	0.0117	4.11E+01	4.11E+01
ENSMUSG00000084816	Platr29	7.60E+00	7.60E+00	2.93	0.0345	4.09E+01	4.09E+01
ENSMUSG00000061728	Btnl7-ps	4.32E+00	4.32E+00	2.11	0.0111	4.09E+01	4.09E+01
ENSMUSG00000062933	Gm10123	1.17E-01	-8.52E+00	-3.09	0.0438	-4.07E+01	4.07E+01
ENSMUSG00000020734	Grin2c	3.52E-01	-2.84E+00	-1.51	0.0049	-4.07E+01	4.07E+01
ENSMUSG00000040809	Chil3	2.30E+00	2.30E+00	1.20	0.0032	4.07E+01	4.07E+01
ENSMUSG00000028125	Abca4	7.44E+00	7.44E+00	2.90	0.0337	4.05E+01	4.05E+01
ENSMUSG00000017002	Slpi	2.64E+00	2.64E+00	1.40	0.0043	4.05E+01	4.05E+01
ENSMUSG00000001901	Kcnh6	9.01E+00	9.01E+00	3.17	0.0495	4.05E+01	4.05E+01
ENSMUSG00000097652	Mhrt	1.74E-01	-5.74E+00	-2.52	0.0202	-4.04E+01	4.04E+01
ENSMUSG00000094910	D430019H16Rik	3.08E-01	-3.25E+00	-1.70	0.0065	-4.02E+01	4.02E+01
ENSMUSG00000054046	Klk13	2.60E-01	-3.85E+00	-1.94	0.0092	-4.01E+01	4.01E+01
ENSMUSG00000047228	BC048546	2.52E+00	2.52E+00	1.33	0.0039	4.01E+01	4.01E+01
ENSMUSG00000087365	C430049B03Rik	1.90E-01	-5.25E+00	-2.39	0.0172	-4.01E+01	4.01E+01
ENSMUSG00000093079	Gm25953	2.12E-01	-4.72E+00	-2.24	0.0139	-4.00E+01	4.00E+01
ENSMUSG00000086459	1700030C12Rik	6.83E+00	6.83E+00	2.77	0.0292	3.99E+01	3.99E+01
ENSMUSG00000081965	Gm11620	6.42E+00	6.42E+00	2.68	0.0259	3.99E+01	3.99E+01
ENSMUSG00000102398	Gm37580	3.78E+00	3.78E+00	1.92	0.0090	3.99E+01	3.99E+01
ENSMUSG00000045257	Morn2	4.07E-01	-2.46E+00	-1.30	0.0038	-3.99E+01	3.99E+01
ENSMUSG00000063626	Unc5d	2.72E-01	-3.68E+00	-1.88	0.0085	-3.98E+01	3.98E+01
ENSMUSG00000022875	Kng1	8.18E+00	8.18E+00	3.03	0.0423	3.98E+01	3.98E+01
ENSMUSG00000017679	Ttpal	3.43E+00	3.43E+00	1.78	0.0074	3.98E+01	3.98E+01
ENSMUSG00000024979	Tectb	7.98E+00	7.98E+00	3.00	0.0404	3.97E+01	3.97E+01
ENSMUSG00000087115	Pcsk2os2	7.13E+00	7.13E+00	2.83	0.0321	3.97E+01	3.97E+01
ENSMUSG00000082476	Gm12242	5.79E+00	5.79E+00	2.53	0.0213	3.97E+01	3.97E+01
ENSMUSG00000106491	RP23-342M4.2	2.90E-01	-3.44E+00	-1.78	0.0076	-3.96E+01	3.96E+01
ENSMUSG00000032400	Zwilch	4.99E+00	4.99E+00	2.32	0.0159	3.96E+01	3.96E+01
ENSMUSG00000043773	1700048O20Rik	3.18E+00	3.18E+00	1.67	0.0065	3.96E+01	3.96E+01
ENSMUSG00000070342	Gm10287	2.99E-01	-3.34E+00	-1.74	0.0072	-3.94E+01	3.94E+01
ENSMUSG00000097805	Gm17473	1.86E-01	-5.36E+00	-2.42	0.0186	-3.94E+01	3.94E+01

ENSMUSG00000033854	Kcnk10	1.96E-01	-5.10E+00	-2.35	0.0169	-3.93E+01	3.93E+01
ENSMUSG00000004988	Fxyd4	2.49E+00	2.49E+00	1.31	0.0040	3.92E+01	3.92E+01
ENSMUSG00000074403	Hist2h3b	2.47E-01	-4.05E+00	-2.02	0.0108	-3.91E+01	3.91E+01
ENSMUSG00000103377	Gm37180	3.93E-01	-2.55E+00	-1.35	0.0043	-3.91E+01	3.91E+01
ENSMUSG00000022272	Myo10	4.29E-01	-2.33E+00	-1.22	0.0036	-3.90E+01	3.90E+01
ENSMUSG00000020599	Rgs9	4.36E-01	-2.29E+00	-1.20	0.0035	-3.89E+01	3.89E+01
ENSMUSG00000032698	Lmo2	4.09E-01	-2.45E+00	-1.29	0.0039	-3.89E+01	3.89E+01
ENSMUSG00000068686	Cd59b	2.30E-01	-4.35E+00	-2.12	0.0125	-3.89E+01	3.89E+01
ENSMUSG00000074491	Clec4g	2.18E+00	2.18E+00	1.12	0.0031	3.88E+01	3.88E+01
ENSMUSG00000031220	Awat2	4.79E-01	-2.09E+00	-1.06	0.0029	-3.87E+01	3.87E+01
ENSMUSG00000024044	Epb4.1l3	5.00E-01	-2.00E+00	-1.00	0.0027	-3.87E+01	3.87E+01
ENSMUSG00000101848	4933417E11Rik	1.71E-01	-5.86E+00	-2.55	0.0231	-3.86E+01	3.86E+01
ENSMUSG00000041722	Khdc1c	1.71E-01	-5.86E+00	-2.55	0.0231	-3.86E+01	3.86E+01
ENSMUSG00000021123	Rdh12	8.35E+00	8.35E+00	3.06	0.0469	3.85E+01	3.85E+01
ENSMUSG00000028399	Ptprd	4.82E-01	-2.08E+00	-1.05	0.0029	-3.85E+01	3.85E+01
ENSMUSG00000074505	Fat3	2.20E-01	-4.54E+00	-2.18	0.0140	-3.84E+01	3.84E+01
ENSMUSG00000020884	Asgr1	4.03E-01	-2.48E+00	-1.31	0.0042	-3.84E+01	3.84E+01
ENSMUSG00000062591	Tubb4a	3.43E-01	-2.91E+00	-1.54	0.0058	-3.84E+01	3.84E+01
ENSMUSG00000084399	Gm15666	3.28E-01	-3.05E+00	-1.61	0.0064	-3.82E+01	3.82E+01
ENSMUSG00000105750	RP23-10F17.2	3.85E-01	-2.59E+00	-1.38	0.0046	-3.81E+01	3.81E+01
ENSMUSG00000063632	Sox11	1.89E-01	-5.29E+00	-2.40	0.0193	-3.81E+01	3.81E+01
ENSMUSG00000044707	Ccnjl	3.95E-01	-2.53E+00	-1.34	0.0044	-3.81E+01	3.81E+01
ENSMUSG00000023886	Smoc2	2.31E+00	2.31E+00	1.21	0.0037	3.81E+01	3.81E+01
ENSMUSG00000105022	RP23-151D16.2	2.54E+00	2.54E+00	1.35	0.0045	3.80E+01	3.80E+01
ENSMUSG00000081490	Gm11830	6.20E+00	6.20E+00	2.63	0.0268	3.79E+01	3.79E+01
ENSMUSG00000034771	Tle2	2.77E-01	-3.61E+00	-1.85	0.0091	-3.78E+01	3.78E+01
ENSMUSG00000070699	Sars2	3.82E-01	-2.62E+00	-1.39	0.0048	-3.77E+01	3.77E+01
ENSMUSG00000102548	Gm16701	5.69E+00	5.69E+00	2.51	0.0228	3.77E+01	3.77E+01
ENSMUSG00000024028	Tff2	2.96E-01	-3.37E+00	-1.75	0.0080	-3.76E+01	3.76E+01
ENSMUSG00000048230	Fbxo43	7.88E+00	7.88E+00	2.98	0.0441	3.75E+01	3.75E+01
ENSMUSG00000038388	Mpp6	2.73E+00	2.73E+00	1.45	0.0053	3.75E+01	3.75E+01
ENSMUSG00000085177	Gm11209	2.41E-01	-4.15E+00	-2.05	0.0122	-3.75E+01	3.75E+01
ENSMUSG00000027656	Wisp2	3.42E-01	-2.93E+00	-1.55	0.0061	-3.74E+01	3.74E+01
ENSMUSG00000067224	Gm3695	2.28E-01	-4.39E+00	-2.13	0.0138	-3.74E+01	3.74E+01
ENSMUSG00000003469	Phyhip	2.24E+00	2.24E+00	1.17	0.0036	3.74E+01	3.74E+01
ENSMUSG00000021388	Aspn	3.54E-01	-2.82E+00	-1.50	0.0057	-3.73E+01	3.73E+01
ENSMUSG00000075829	Gm23692	3.75E-01	-2.66E+00	-1.41	0.0051	-3.73E+01	3.73E+01
ENSMUSG00000021708	Rasgrf2	3.37E-01	-2.97E+00	-1.57	0.0064	-3.73E+01	3.73E+01
ENSMUSG00000105135	RP23-418O21.3	3.92E-01	-2.55E+00	-1.35	0.0047	-3.72E+01	3.72E+01
ENSMUSG00000019817	Plagl1	4.86E-01	-2.06E+00	-1.04	0.0031	-3.72E+01	3.72E+01
ENSMUSG00000050097	Ces2b	1.41E-01	-7.08E+00	-2.82	0.0363	-3.71E+01	3.71E+01
ENSMUSG00000052516	Robo2	3.53E-01	-2.83E+00	-1.50	0.0058	-3.71E+01	3.71E+01
ENSMUSG00000085606	Gm15792	6.84E+00	6.84E+00	2.77	0.0341	3.71E+01	3.71E+01
ENSMUSG00000022676	Snai2	2.58E-01	-3.88E+00	-1.96	0.0109	-3.71E+01	3.71E+01
ENSMUSG00000085936	2610307P16Rik	2.68E-01	-3.73E+00	-1.90	0.0102	-3.70E+01	3.70E+01
ENSMUSG00000031673	Cdh11	3.69E-01	-2.71E+00	-1.44	0.0054	-3.70E+01	3.70E+01
ENSMUSG00000101268	2010310C07Rik	2.35E+00	2.35E+00	1.23	0.0040	3.70E+01	3.70E+01
ENSMUSG00000100235	Gm28557	3.95E-01	-2.53E+00	-1.34	0.0047	-3.69E+01	3.69E+01
ENSMUSG00000004842	Pou1f1	8.05E+00	8.05E+00	3.01	0.0479	3.68E+01	3.68E+01
ENSMUSG00000083698	Gm12687	1.71E-01	-5.85E+00	-2.55	0.0254	-3.67E+01	3.67E+01
ENSMUSG00000028780	Sema3c	2.86E-01	-3.50E+00	-1.81	0.0092	-3.66E+01	3.66E+01
ENSMUSG00000032649	Colgalt2	4.22E-01	-2.37E+00	-1.24	0.0042	-3.66E+01	3.66E+01
ENSMUSG00000028417	Tal2	3.94E+00	3.94E+00	1.98	0.0118	3.64E+01	3.64E+01
ENSMUSG00000054753	AU018091	7.32E+00	7.32E+00	2.87	0.0407	3.63E+01	3.63E+01
ENSMUSG00000103834	Gm37613	2.45E-01	-4.08E+00	-2.03	0.0127	-3.63E+01	3.63E+01
ENSMUSG00000025933	Tmem14a	2.65E-01	-3.77E+00	-1.91	0.0108	-3.62E+01	3.62E+01
ENSMUSG00000028262	Clca3a2	4.56E+00	4.56E+00	2.19	0.0159	3.62E+01	3.62E+01
ENSMUSG00000021280	Exoc3l4	4.09E-01	-2.45E+00	-1.29	0.0046	-3.61E+01	3.61E+01
ENSMUSG00000042834	Nrep	3.13E-01	-3.19E+00	-1.67	0.0078	-3.61E+01	3.61E+01
ENSMUSG00000097134	1110002J07Rik	7.12E+00	7.12E+00	2.83	0.0389	3.61E+01	3.61E+01
ENSMUSG00000041731	Pgm5	3.69E-01	-2.71E+00	-1.44	0.0056	-3.60E+01	3.60E+01
ENSMUSG00000022996	Wnt10b	2.57E-01	-3.90E+00	-1.96	0.0117	-3.60E+01	3.60E+01
ENSMUSG00000025666	Tmem47	4.08E-01	-2.45E+00	-1.29	0.0047	-3.59E+01	3.59E+01
ENSMUSG00000061863	Gm6822	7.40E+00	7.40E+00	2.89	0.0425	3.59E+01	3.59E+01
ENSMUSG00000029255	Gnrhr	4.02E+00	4.02E+00	2.01	0.0126	3.58E+01	3.58E+01
ENSMUSG00000021609	Slc6a3	7.39E+00	7.39E+00	2.89	0.0427	3.58E+01	3.58E+01
ENSMUSG00000031070	Mrgprf	3.34E-01	-3.00E+00	-1.58	0.0071	-3.56E+01	3.56E+01

ENSMUSG00000081389	Gm13368	5.25E+00	5.25E+00	2.39	0.0218	3.56E+01	3.56E+01
ENSMUSG00000036123	Slc9a3	4.17E+00	4.17E+00	2.06	0.0138	3.55E+01	3.55E+01
ENSMUSG00000079852	Klra4	1.92E-01	-5.22E+00	-2.38	0.0218	-3.53E+01	3.53E+01
ENSMUSG00000040447	Spns2	4.57E-01	-2.19E+00	-1.13	0.0038	-3.53E+01	3.53E+01
ENSMUSG00000097467	Gm26737	5.74E+00	5.74E+00	2.52	0.0266	3.52E+01	3.52E+01
ENSMUSG00000086482	1700012C08Rik	2.80E-01	-3.57E+00	-1.84	0.0103	-3.52E+01	3.52E+01
ENSMUSG00000052921	Arhgef15	2.83E-01	-3.54E+00	-1.82	0.0101	-3.51E+01	3.51E+01
ENSMUSG00000106749	RP24-79P8.1	4.46E-01	-2.24E+00	-1.16	0.0041	-3.50E+01	3.50E+01
ENSMUSG00000037071	Scd1	2.80E-01	-3.57E+00	-1.83	0.0104	-3.50E+01	3.50E+01
ENSMUSG00000083226	Gm7831	7.23E+00	7.23E+00	2.85	0.0428	3.50E+01	3.50E+01
ENSMUSG00000099465	Gm3830	6.85E+00	6.85E+00	2.78	0.0385	3.49E+01	3.49E+01
ENSMUSG00000047420	Fam180a	2.02E-01	-4.95E+00	-2.31	0.0202	-3.48E+01	3.48E+01
ENSMUSG00000037940	Inpp4b	3.45E-01	-2.90E+00	-1.54	0.0069	-3.48E+01	3.48E+01
ENSMUSG00000023978	Prph2	6.65E+00	6.65E+00	2.73	0.0367	3.47E+01	3.47E+01
ENSMUSG00000031842	Pde4c	5.19E+00	5.19E+00	2.38	0.0223	3.47E+01	3.47E+01
ENSMUSG00000037379	Spon2	2.11E-01	-4.75E+00	-2.25	0.0187	-3.47E+01	3.47E+01
ENSMUSG00000026874	Hc	2.86E-01	-3.50E+00	-1.81	0.0102	-3.46E+01	3.46E+01
ENSMUSG00000103577	9330162B11Rik	2.13E-01	-4.69E+00	-2.23	0.0185	-3.44E+01	3.44E+01
ENSMUSG00000103145	Gm37744	5.56E+00	5.56E+00	2.47	0.0261	3.44E+01	3.44E+01
ENSMUSG00000076471	Trbv14	1.93E-01	-5.19E+00	-2.38	0.0227	-3.44E+01	3.44E+01
ENSMUSG00000055102	Zfp819	7.05E+00	7.05E+00	2.82	0.0420	3.44E+01	3.44E+01
ENSMUSG00000034810	Scn7a	3.52E-01	-2.84E+00	-1.51	0.0068	-3.44E+01	3.44E+01
ENSMUSG00000083674	Zfp133-ps	3.99E-01	-2.51E+00	-1.33	0.0053	-3.43E+01	3.43E+01
ENSMUSG00000103959	Gm37720	2.35E-01	-4.26E+00	-2.09	0.0154	-3.43E+01	3.43E+01
ENSMUSG00000029564	4930519G04Rik	5.92E+00	5.92E+00	2.57	0.0298	3.43E+01	3.43E+01
ENSMUSG00000062444	Ap3b2	3.41E+00	3.41E+00	1.77	0.0100	3.42E+01	3.42E+01
ENSMUSG00000050215	Olfr70	6.91E+00	6.91E+00	2.79	0.0409	3.42E+01	3.42E+01
ENSMUSG00000058101	1700084J12Rik	3.36E-01	-2.98E+00	-1.57	0.0076	-3.42E+01	3.42E+01
ENSMUSG00000005320	Fgfr4	3.66E-01	-2.73E+00	-1.45	0.0065	-3.40E+01	3.40E+01
ENSMUSG00000068689	Rps19-ps9	5.53E+00	5.53E+00	2.47	0.0265	3.39E+01	3.39E+01
ENSMUSG00000083250	Gm5403	4.72E-01	-2.12E+00	-1.08	0.0039	-3.39E+01	3.39E+01
ENSMUSG00000105160	RP23-272P19.10	2.17E-01	-4.60E+00	-2.20	0.0185	-3.38E+01	3.38E+01
ENSMUSG00000044086	Lmod3	3.08E-01	-3.25E+00	-1.70	0.0093	-3.37E+01	3.37E+01
ENSMUSG00000103401	Gm37929	1.42E-01	-7.05E+00	-2.82	0.0439	-3.37E+01	3.37E+01
ENSMUSG00000045915	Ccdc42	1.96E-01	-5.10E+00	-2.35	0.0230	-3.37E+01	3.37E+01
ENSMUSG00000106536	RP23-394K8.5	2.51E-01	-3.99E+00	-2.00	0.0141	-3.36E+01	3.36E+01
ENSMUSG00000000402	Egfl6	3.30E-01	-3.03E+00	-1.60	0.0081	-3.36E+01	3.36E+01
ENSMUSG00000020695	Mrc2	3.35E-01	-2.99E+00	-1.58	0.0079	-3.36E+01	3.36E+01
ENSMUSG00000095306	Gm21807	2.24E-01	-4.47E+00	-2.16	0.0177	-3.36E+01	3.36E+01
ENSMUSG00000086843	E030013I19Rik	2.05E-01	-4.89E+00	-2.29	0.0214	-3.34E+01	3.34E+01
ENSMUSG00000086105	Gm11636	1.37E-01	-7.28E+00	-2.86	0.0477	-3.33E+01	3.33E+01
ENSMUSG00000063820	Arl9	6.15E+00	6.15E+00	2.62	0.0341	3.33E+01	3.33E+01
ENSMUSG00000101926	Gm28904	2.79E-01	-3.59E+00	-1.84	0.0117	-3.32E+01	3.32E+01
ENSMUSG00000031099	Smarca1	4.07E-01	-2.46E+00	-1.30	0.0055	-3.32E+01	3.32E+01
ENSMUSG00000079507	H2-Q1	2.77E-01	-3.60E+00	-1.85	0.0119	-3.31E+01	3.31E+01
ENSMUSG00000028082	Sh3d19	3.67E-01	-2.73E+00	-1.45	0.0068	-3.31E+01	3.31E+01
ENSMUSG00000097058	4930556M19Rik	6.75E+00	6.75E+00	2.76	0.0418	3.30E+01	3.30E+01
ENSMUSG00000097401	Gm26744	4.37E-01	-2.29E+00	-1.19	0.0048	-3.30E+01	3.30E+01
ENSMUSG00000027380	Acoxl	1.96E-01	-5.09E+00	-2.35	0.0239	-3.30E+01	3.30E+01
ENSMUSG00000106734	RP24-328P2.5	3.04E-01	-3.28E+00	-1.72	0.0099	-3.30E+01	3.30E+01
ENSMUSG00000040289	Hey1	2.93E-01	-3.41E+00	-1.77	0.0107	-3.29E+01	3.29E+01
ENSMUSG00000022372	Sla	2.82E+00	2.82E+00	1.50	0.0074	3.29E+01	3.29E+01
ENSMUSG00000040485	Lrrc52	2.24E-01	-4.47E+00	-2.16	0.0185	-3.29E+01	3.29E+01
ENSMUSG00000087264	Gad1os	2.49E-01	-4.01E+00	-2.00	0.0149	-3.29E+01	3.29E+01
ENSMUSG00000104823	RP23-336O18.2	4.91E+00	4.91E+00	2.29	0.0224	3.28E+01	3.28E+01
ENSMUSG00000097714	Gm20109	7.06E+00	7.06E+00	2.82	0.0467	3.27E+01	3.27E+01
ENSMUSG00000036928	Stag3	2.70E+00	2.70E+00	1.43	0.0069	3.26E+01	3.26E+01
ENSMUSG00000026418	Tnni1	4.25E+00	4.25E+00	2.09	0.0171	3.25E+01	3.25E+01
ENSMUSG00000106265	RP23-201E13.1	4.33E-01	-2.31E+00	-1.21	0.0051	-3.24E+01	3.24E+01
ENSMUSG00000097305	Gm17276	2.35E-01	-4.25E+00	-2.09	0.0174	-3.23E+01	3.23E+01
ENSMUSG00000048538	Gm9826	2.88E+00	2.88E+00	1.53	0.0080	3.23E+01	3.23E+01
ENSMUSG00000097448	Platr22	3.08E-01	-3.24E+00	-1.70	0.0101	-3.22E+01	3.22E+01
ENSMUSG00000024027	Glp1r	1.79E-01	-5.58E+00	-2.48	0.0302	-3.21E+01	3.21E+01
ENSMUSG00000018776	Slc35g3	2.06E+00	2.06E+00	1.04	0.0041	3.21E+01	3.21E+01
ENSMUSG00000068417	Pnp2	3.45E+00	3.45E+00	1.79	0.0117	3.20E+01	3.20E+01
ENSMUSG00000074001	Klhl40	5.74E+00	5.74E+00	2.52	0.0322	3.20E+01	3.20E+01
ENSMUSG00000097736	9530059O14Rik	1.83E-01	-5.46E+00	-2.45	0.0293	-3.19E+01	3.19E+01

ENSMUSG00000072875	Gpr27	1.86E-01	-5.36E+00	-2.42	0.0283	-3.19E+01	3.19E+01
ENSMUSG00000090104	Slmapos2	2.39E+00	2.39E+00	1.25	0.0057	3.17E+01	3.17E+01
ENSMUSG00000062309	Rpp25	3.01E-01	-3.32E+00	-1.73	0.0110	-3.16E+01	3.16E+01
ENSMUSG00000037833	Sh2d4b	3.23E+00	3.23E+00	1.69	0.0105	3.16E+01	3.16E+01
ENSMUSG00000080851	Gm12094	1.85E-01	-5.41E+00	-2.43	0.0293	-3.16E+01	3.16E+01
ENSMUSG00000071856	Mcc	2.86E-01	-3.49E+00	-1.80	0.0122	-3.16E+01	3.16E+01
ENSMUSG00000031147	Magix	3.47E-01	-2.88E+00	-1.53	0.0084	-3.14E+01	3.14E+01
ENSMUSG00000101905	4930439D14Rik	1.51E-01	-6.64E+00	-2.73	0.0449	-3.13E+01	3.13E+01
ENSMUSG00000104445	Rhbg	4.78E-01	-2.09E+00	-1.07	0.0045	-3.13E+01	3.13E+01
ENSMUSG00000006390	Elovl1	4.42E-01	-2.26E+00	-1.18	0.0052	-3.13E+01	3.13E+01
ENSMUSG00000102781	Pla2r1	2.77E-01	-3.61E+00	-1.85	0.0134	-3.12E+01	3.12E+01
ENSMUSG00000024598	Fbn2	2.86E-01	-3.49E+00	-1.80	0.0126	-3.11E+01	3.11E+01
ENSMUSG00000037974	Muc5ac	2.68E+00	2.68E+00	1.42	0.0075	3.10E+01	3.10E+01
ENSMUSG00000073739	Gm16287	5.81E+00	5.81E+00	2.54	0.0354	3.09E+01	3.09E+01
ENSMUSG00000044461	Shisa2	1.92E-01	-5.22E+00	-2.38	0.0286	-3.09E+01	3.09E+01
ENSMUSG00000086807	Platr21	4.09E-01	-2.45E+00	-1.29	0.0063	-3.09E+01	3.09E+01
ENSMUSG00000065086	Gm26495	2.26E-01	-4.42E+00	-2.14	0.0206	-3.08E+01	3.08E+01
ENSMUSG00000026401	Daf2	2.53E-01	-3.95E+00	-1.98	0.0164	-3.08E+01	3.08E+01
ENSMUSG00000035228	Ccdc106	5.35E+00	5.35E+00	2.42	0.0302	3.08E+01	3.08E+01
ENSMUSG00000040147	Maob	3.94E-01	-2.54E+00	-1.34	0.0068	-3.08E+01	3.08E+01
ENSMUSG00000078365	Mos	2.97E+00	2.97E+00	1.57	0.0093	3.07E+01	3.07E+01
ENSMUSG00000102759	Gm10463	6.63E+00	6.63E+00	2.73	0.0468	3.06E+01	3.06E+01
ENSMUSG00000059187	Fam19a1	4.16E-01	-2.40E+00	-1.26	0.0062	-3.06E+01	3.06E+01
ENSMUSG00000103291	Gm38235	4.39E-01	-2.28E+00	-1.19	0.0055	-3.06E+01	3.06E+01
ENSMUSG00000036196	Slc26a8	4.49E+00	4.49E+00	2.17	0.0216	3.06E+01	3.06E+01
ENSMUSG00000085442	Gm3362	4.00E-01	-2.50E+00	-1.32	0.0067	-3.06E+01	3.06E+01
ENSMUSG00000021314	Amph	3.40E-01	-2.94E+00	-1.55	0.0092	-3.05E+01	3.05E+01
ENSMUSG00000020032	Nuak1	3.52E-01	-2.84E+00	-1.51	0.0087	-3.05E+01	3.05E+01
ENSMUSG00000033774	Npbwr1	3.17E+00	3.17E+00	1.66	0.0108	3.05E+01	3.05E+01
ENSMUSG00000082128	Gm11824	5.27E+00	5.27E+00	2.40	0.0298	3.05E+01	3.05E+01
ENSMUSG00000101179	Gm29455	3.61E-01	-2.77E+00	-1.47	0.0083	-3.05E+01	3.05E+01
ENSMUSG00000082100	Glns-ps1	2.69E+00	2.69E+00	1.43	0.0078	3.04E+01	3.04E+01
ENSMUSG00000088389	Gm24685	2.68E-01	-3.74E+00	-1.90	0.0151	-3.04E+01	3.04E+01
ENSMUSG00000097082	4933440J02Rik	1.89E-01	-5.29E+00	-2.40	0.0304	-3.04E+01	3.04E+01
ENSMUSG00000097068	Gm26760	2.28E-01	-4.38E+00	-2.13	0.0209	-3.03E+01	3.03E+01
ENSMUSG00000050234	Gja4	3.34E-01	-3.00E+00	-1.58	0.0098	-3.03E+01	3.03E+01
ENSMUSG00000107184	Gm18222	4.11E+00	4.11E+00	2.04	0.0184	3.03E+01	3.03E+01
ENSMUSG00000105975	RP24-372J8.3	2.49E-01	-4.01E+00	-2.00	0.0175	-3.03E+01	3.03E+01
ENSMUSG00000049690	Nckap5	2.77E-01	-3.62E+00	-1.85	0.0143	-3.03E+01	3.03E+01
ENSMUSG00000043383	Olfr1342	1.68E-01	-5.94E+00	-2.57	0.0387	-3.02E+01	3.02E+01
ENSMUSG00000097263	Gm26804	2.34E-01	-4.27E+00	-2.09	0.0200	-3.02E+01	3.02E+01
ENSMUSG00000105993	RP23-280E8.2	3.72E+00	3.72E+00	1.89	0.0152	3.01E+01	3.01E+01
ENSMUSG00000020829	Slc46a1	3.59E-01	-2.78E+00	-1.48	0.0086	-3.01E+01	3.01E+01
ENSMUSG00000050241	Klre1	3.45E-01	-2.90E+00	-1.53	0.0093	-3.01E+01	3.01E+01
ENSMUSG00000050288	Fzd2	3.54E-01	-2.82E+00	-1.50	0.0089	-3.00E+01	3.00E+01
ENSMUSG00000041757	Plekha6	3.79E-01	-2.64E+00	-1.40	0.0078	-3.00E+01	3.00E+01
ENSMUSG00000041426	Hibch	4.59E-01	-2.18E+00	-1.12	0.0053	-2.99E+01	2.99E+01
ENSMUSG00000034762	Glis1	4.87E+00	4.87E+00	2.28	0.0267	2.98E+01	2.98E+01
ENSMUSG00000100490	Gm28455	3.78E+00	3.78E+00	1.92	0.0161	2.98E+01	2.98E+01
ENSMUSG00000026816	Gtf3c5	4.57E-01	-2.19E+00	-1.13	0.0054	-2.97E+01	2.97E+01
ENSMUSG00000025766	D3Ertd751e	3.54E-01	-2.83E+00	-1.50	0.0091	-2.97E+01	2.97E+01
ENSMUSG00000071335	G630090E17Rik	2.33E-01	-4.30E+00	-2.10	0.0210	-2.97E+01	2.97E+01
ENSMUSG00000091556	Gm14569	3.62E-01	-2.76E+00	-1.46	0.0087	-2.97E+01	2.97E+01
ENSMUSG00000030134	Rasgef1a	3.36E-01	-2.97E+00	-1.57	0.0101	-2.96E+01	2.96E+01
ENSMUSG00000097636	5830416P10Rik	2.43E+00	2.43E+00	1.28	0.0067	2.96E+01	2.96E+01
ENSMUSG00000025789	St8sia2	4.49E-01	-2.23E+00	-1.16	0.0057	-2.96E+01	2.96E+01
ENSMUSG00000101433	Gm29441	5.35E+00	5.35E+00	2.42	0.0329	2.95E+01	2.95E+01
ENSMUSG00000097930	C330002G04Rik	2.23E+00	2.23E+00	1.16	0.0057	2.95E+01	2.95E+01
ENSMUSG00000052435	Cebpe	3.92E+00	3.92E+00	1.97	0.0176	2.95E+01	2.95E+01
ENSMUSG00000017007	Rbpjl	2.86E-01	-3.50E+00	-1.81	0.0141	-2.94E+01	2.94E+01
ENSMUSG00000047104	Pbp2	3.88E-01	-2.57E+00	-1.36	0.0077	-2.93E+01	2.93E+01
ENSMUSG00000082223	Gm15079	2.01E+00	2.01E+00	1.01	0.0047	2.93E+01	2.93E+01
ENSMUSG00000083618	Gm11771	4.75E+00	4.75E+00	2.25	0.0263	2.93E+01	2.93E+01
ENSMUSG00000102526	Gm37785	2.52E+00	2.52E+00	1.33	0.0074	2.93E+01	2.93E+01
ENSMUSG00000074415	2610203C20Rik	3.98E-01	-2.51E+00	-1.33	0.0074	-2.92E+01	2.92E+01
ENSMUSG00000068574	Olfr458	2.68E+00	2.68E+00	1.42	0.0084	2.92E+01	2.92E+01
ENSMUSG00000093673	Gm20644	6.45E+00	6.45E+00	2.69	0.0489	2.92E+01	2.92E+01

ENSMUSG00000090105	Gm15890	2.57E+00	2.57E+00	1.36	0.0078	2.92E+01	2.92E+01
ENSMUSG00000087027	Gm13206	5.30E+00	5.30E+00	2.41	0.0331	2.92E+01	2.92E+01
ENSMUSG00000099989	Gm20281	2.27E-01	-4.41E+00	-2.14	0.0229	-2.91E+01	2.91E+01
ENSMUSG00000005360	Slc1a3	4.94E-01	-2.02E+00	-1.02	0.0048	-2.91E+01	2.91E+01
ENSMUSG00000084240	Gm15383	3.88E-01	-2.58E+00	-1.36	0.0078	-2.91E+01	2.91E+01
ENSMUSG00000092622	Khdc3	2.68E+00	2.68E+00	1.42	0.0086	2.89E+01	2.89E+01
ENSMUSG00000081769	Gm12216	4.09E-01	-2.45E+00	-1.29	0.0072	-2.89E+01	2.89E+01
ENSMUSG00000085135	Gm13713	2.11E-01	-4.75E+00	-2.25	0.0271	-2.88E+01	2.88E+01
ENSMUSG00000033420	Antxr1	3.44E-01	-2.91E+00	-1.54	0.0102	-2.88E+01	2.88E+01
ENSMUSG00000087397	Rapgef3os2	4.69E+00	4.69E+00	2.23	0.0266	2.88E+01	2.88E+01
ENSMUSG00000039714	Cplx3	5.88E+00	5.88E+00	2.56	0.0420	2.87E+01	2.87E+01
ENSMUSG00000107290	RP24-383P24.2	4.46E-01	-2.24E+00	-1.17	0.0061	-2.87E+01	2.87E+01
ENSMUSG00000037440	Vnn1	2.78E-01	-3.60E+00	-1.85	0.0158	-2.86E+01	2.86E+01
ENSMUSG00000063063	Ctnna2	1.66E-01	-6.02E+00	-2.59	0.0442	-2.86E+01	2.86E+01
ENSMUSG00000053388	Trim50	5.04E+00	5.04E+00	2.33	0.0310	2.86E+01	2.86E+01
ENSMUSG00000085514	Bcas3os2	3.20E-01	-3.13E+00	-1.65	0.0120	-2.86E+01	2.86E+01
ENSMUSG00000020546	Stxbp4	2.58E-01	-3.87E+00	-1.95	0.0184	-2.86E+01	2.86E+01
ENSMUSG00000022774	Ncbp2	4.05E-01	-2.47E+00	-1.30	0.0075	-2.85E+01	2.85E+01
ENSMUSG00000070511	Gm10295	1.89E-01	-5.29E+00	-2.40	0.0344	-2.85E+01	2.85E+01
ENSMUSG00000031274	Col4a5	3.84E-01	-2.60E+00	-1.38	0.0084	-2.85E+01	2.85E+01
ENSMUSG00000097120	Gm26887	3.18E+00	3.18E+00	1.67	0.0125	2.84E+01	2.84E+01
ENSMUSG00000041012	Cmtm8	4.19E-01	-2.39E+00	-1.26	0.0071	-2.84E+01	2.84E+01
ENSMUSG00000022762	Ncam2	3.35E-01	-2.99E+00	-1.58	0.0111	-2.84E+01	2.84E+01
ENSMUSG00000032015	Pou2f3	5.22E+00	5.22E+00	2.39	0.0340	2.84E+01	2.84E+01
ENSMUSG00000025488	Cox8b	3.63E-01	-2.76E+00	-1.46	0.0095	-2.83E+01	2.83E+01
ENSMUSG00000058145	Adamts17	2.61E-01	-3.82E+00	-1.94	0.0183	-2.83E+01	2.83E+01
ENSMUSG00000025893	Kbtbd3	4.61E-01	-2.17E+00	-1.12	0.0059	-2.83E+01	2.83E+01
ENSMUSG00000086369	E330017L17Rik	3.69E+00	3.69E+00	1.88	0.0172	2.82E+01	2.82E+01
ENSMUSG00000105471	RP23-396K22.3	1.77E-01	-5.64E+00	-2.50	0.0402	-2.81E+01	2.81E+01
ENSMUSG00000101464	Gm7436	4.48E-01	-2.23E+00	-1.16	0.0063	-2.81E+01	2.81E+01
ENSMUSG00000075588	Hoxb2	4.13E-01	-2.42E+00	-1.27	0.0074	-2.80E+01	2.80E+01
ENSMUSG00000026573	Xcl1	2.16E-01	-4.62E+00	-2.21	0.0272	-2.80E+01	2.80E+01
ENSMUSG00000052525	Spdya	2.09E+00	2.09E+00	1.06	0.0056	2.80E+01	2.80E+01
ENSMUSG00000098093	Gm8707	1.95E-01	-5.14E+00	-2.36	0.0337	-2.80E+01	2.80E+01
ENSMUSG00000038515	Grtp1	2.91E-01	-3.44E+00	-1.78	0.0153	-2.78E+01	2.78E+01
ENSMUSG00000097336	Fendrr	4.22E-01	-2.37E+00	-1.25	0.0073	-2.77E+01	2.77E+01
ENSMUSG00000006369	Fbln1	4.42E-01	-2.26E+00	-1.18	0.0067	-2.77E+01	2.77E+01
ENSMUSG00000031431	Tsc22d3	2.36E+00	2.36E+00	1.24	0.0073	2.77E+01	2.77E+01
ENSMUSG00000028024	Enpep	4.91E-01	-2.04E+00	-1.03	0.0054	-2.77E+01	2.77E+01
ENSMUSG00000038239	Hrc	3.36E-01	-2.97E+00	-1.57	0.0116	-2.76E+01	2.76E+01
ENSMUSG00000106820	RP24-544J6.4	5.84E+00	5.84E+00	2.55	0.0449	2.76E+01	2.76E+01
ENSMUSG00000084939	Gm830	2.12E-01	-4.72E+00	-2.24	0.0292	-2.76E+01	2.76E+01
ENSMUSG00000078722	Gm12394	2.20E+00	2.20E+00	1.13	0.0063	2.76E+01	2.76E+01
ENSMUSG00000055357	4933400A11Rik	2.25E-01	-4.45E+00	-2.15	0.0260	-2.76E+01	2.76E+01
ENSMUSG00000031294	D630029K05Rik	4.07E-01	-2.45E+00	-1.30	0.0080	-2.75E+01	2.75E+01
ENSMUSG00000103308	Gm37800	4.44E+00	4.44E+00	2.15	0.0263	2.74E+01	2.74E+01
ENSMUSG00000102723	Gm37936	2.66E-01	-3.76E+00	-1.91	0.0189	-2.74E+01	2.74E+01
ENSMUSG00000053117	E330013P04Rik	3.54E+00	3.54E+00	1.83	0.0168	2.73E+01	2.73E+01
ENSMUSG00000051716	Apon	3.69E+00	3.69E+00	1.88	0.0183	2.73E+01	2.73E+01
ENSMUSG00000103609	Gm37022	4.01E+00	4.01E+00	2.00	0.0216	2.73E+01	2.73E+01
ENSMUSG00000053835	H2-T24	3.30E-01	-3.03E+00	-1.60	0.0124	-2.72E+01	2.72E+01
ENSMUSG00000043943	Naalad2	4.81E-01	-2.08E+00	-1.05	0.0058	-2.72E+01	2.72E+01
ENSMUSG00000016942	Tmprss6	2.66E+00	2.66E+00	1.41	0.0095	2.72E+01	2.72E+01
ENSMUSG00000087300	Gm16725	2.61E-01	-3.84E+00	-1.94	0.0200	-2.71E+01	2.71E+01
ENSMUSG00000072878	1700123L14Rik	2.14E-01	-4.68E+00	-2.23	0.0298	-2.71E+01	2.71E+01
ENSMUSG00000043421	Hilpda	4.65E-01	-2.15E+00	-1.10	0.0063	-2.71E+01	2.71E+01
ENSMUSG00000024617	Camk2a	3.04E-01	-3.29E+00	-1.72	0.0148	-2.71E+01	2.71E+01
ENSMUSG00000074628	Tldc2	3.76E+00	3.76E+00	1.91	0.0194	2.70E+01	2.70E+01
ENSMUSG00000031385	Plxnb3	3.20E+00	3.20E+00	1.68	0.0141	2.69E+01	2.69E+01
ENSMUSG00000104211	Gm37985	4.88E+00	4.88E+00	2.29	0.0329	2.69E+01	2.69E+01
ENSMUSG00000073888	Ccl27a	3.12E-01	-3.21E+00	-1.68	0.0144	-2.67E+01	2.67E+01
ENSMUSG00000048284	Tas2r126	3.00E+00	3.00E+00	1.58	0.0126	2.67E+01	2.67E+01
ENSMUSG00000032496	Ltf	4.97E+00	4.97E+00	2.31	0.0348	2.66E+01	2.66E+01
ENSMUSG00000090564	A430057M04Rik	5.61E+00	5.61E+00	2.49	0.0444	2.66E+01	2.66E+01
ENSMUSG00000044026	Slc35g1	2.06E+00	2.06E+00	1.04	0.0060	2.66E+01	2.66E+01
ENSMUSG00000106417	RP24-140K12.2	3.56E-01	-2.81E+00	-1.49	0.0112	-2.66E+01	2.66E+01
ENSMUSG00000030114	Klrg1	2.01E-01	-4.97E+00	-2.31	0.0351	-2.65E+01	2.65E+01

ENSMUSG00000030156	Cd69	2.20E+00	2.20E+00	1.14	0.0069	2.65E+01	2.65E+01
ENSMUSG00000001227	Sema6b	2.40E+00	2.40E+00	1.26	0.0082	2.64E+01	2.64E+01
ENSMUSG00000042436	Mfap4	3.34E-01	-2.99E+00	-1.58	0.0129	-2.64E+01	2.64E+01
ENSMUSG00000003199	Mpnd	4.23E-01	-2.37E+00	-1.24	0.0081	-2.64E+01	2.64E+01
ENSMUSG00000031893	Tsnaxip1	3.20E-01	-3.12E+00	-1.64	0.0140	-2.64E+01	2.64E+01
ENSMUSG00000085527	Gm15535	2.57E-01	-3.90E+00	-1.96	0.0220	-2.63E+01	2.63E+01
ENSMUSG00000106212	RP23-114F4.2	4.55E-01	-2.20E+00	-1.14	0.0070	-2.62E+01	2.62E+01
ENSMUSG00000031150	Ccdc120	2.52E+00	2.52E+00	1.34	0.0093	2.62E+01	2.62E+01
ENSMUSG00000028883	Sema3a	4.17E-01	-2.40E+00	-1.26	0.0084	-2.62E+01	2.62E+01
ENSMUSG00000041482	Piezo2	3.05E-01	-3.28E+00	-1.71	0.0158	-2.61E+01	2.61E+01
ENSMUSG00000104903	RP23-225M12.2	4.89E-01	-2.05E+00	-1.03	0.0061	-2.61E+01	2.61E+01
ENSMUSG00000079415	Cntf	2.01E+00	2.01E+00	1.01	0.0060	2.61E+01	2.61E+01
ENSMUSG00000101156	Gm29114	3.96E-01	-2.52E+00	-1.34	0.0094	-2.61E+01	2.61E+01
ENSMUSG00000100396	Gm29367	2.77E-01	-3.62E+00	-1.85	0.0193	-2.61E+01	2.61E+01
ENSMUSG00000010841	1700006E09Rik	3.98E+00	3.98E+00	1.99	0.0233	2.60E+01	2.60E+01
ENSMUSG00000020218	Wif1	3.52E-01	-2.84E+00	-1.50	0.0119	-2.60E+01	2.60E+01
ENSMUSG00000021773	Comtd1	4.42E-01	-2.26E+00	-1.18	0.0076	-2.60E+01	2.60E+01
ENSMUSG00000027398	Il1b	2.20E+00	2.20E+00	1.14	0.0072	2.60E+01	2.60E+01
ENSMUSG00000097387	4930563E18Rik	4.08E+00	4.08E+00	2.03	0.0247	2.60E+01	2.60E+01
ENSMUSG00000039194	Rlbp1	5.15E+00	5.15E+00	2.36	0.0393	2.60E+01	2.60E+01
ENSMUSG00000069727	Gm5595	4.37E-01	-2.29E+00	-1.19	0.0078	-2.59E+01	2.59E+01
ENSMUSG00000104964	RP24-104D12.2	3.90E-01	-2.57E+00	-1.36	0.0098	-2.59E+01	2.59E+01
ENSMUSG00000078869	Gm14409	2.34E-01	-4.27E+00	-2.09	0.0272	-2.59E+01	2.59E+01
ENSMUSG00000031519	Asb5	3.26E+00	3.26E+00	1.70	0.0158	2.59E+01	2.59E+01
ENSMUSG00000039763	Dnajc28	4.13E-01	-2.42E+00	-1.28	0.0088	-2.59E+01	2.59E+01
ENSMUSG00000033585	Ndn	3.37E-01	-2.96E+00	-1.57	0.0132	-2.58E+01	2.58E+01
ENSMUSG00000007097	Atp1a2	4.03E-01	-2.48E+00	-1.31	0.0092	-2.58E+01	2.58E+01
ENSMUSG00000040841	Six5	3.84E-01	-2.61E+00	-1.38	0.0102	-2.58E+01	2.58E+01
ENSMUSG00000097632	4930552P12Rik	5.00E+00	5.00E+00	2.32	0.0376	2.58E+01	2.58E+01
ENSMUSG00000042638	Gucy2c	4.63E+00	4.63E+00	2.21	0.0323	2.58E+01	2.58E+01
ENSMUSG00000040653	Ppp1r14c	4.05E-01	-2.47E+00	-1.30	0.0092	-2.58E+01	2.58E+01
ENSMUSG00000038695	Josd2	3.59E-01	-2.79E+00	-1.48	0.0117	-2.58E+01	2.58E+01
ENSMUSG00000048827	Pkd1l3	3.36E+00	3.36E+00	1.75	0.0170	2.57E+01	2.57E+01
ENSMUSG00000026839	Upp2	2.55E-01	-3.92E+00	-1.97	0.0233	-2.57E+01	2.57E+01
ENSMUSG00000097325	Gm16897	2.97E+00	2.97E+00	1.57	0.0134	2.57E+01	2.57E+01
ENSMUSG00000026768	Itga8	3.59E-01	-2.78E+00	-1.48	0.0118	-2.56E+01	2.56E+01
ENSMUSG00000104876	Trdc	2.50E-01	-3.99E+00	-2.00	0.0243	-2.56E+01	2.56E+01
ENSMUSG00000080780	Gm11252	2.36E-01	-4.24E+00	-2.08	0.0276	-2.55E+01	2.55E+01
ENSMUSG00000107234	RP23-319P12.5	4.27E-01	-2.34E+00	-1.23	0.0084	-2.55E+01	2.55E+01
ENSMUSG00000043664	Tmem221	3.13E-01	-3.20E+00	-1.68	0.0157	-2.55E+01	2.55E+01
ENSMUSG00000103552	Gm38217	1.81E-01	-5.52E+00	-2.46	0.0468	-2.55E+01	2.55E+01
ENSMUSG00000086379	1700026D11Rik	3.51E+00	3.51E+00	1.81	0.0190	2.55E+01	2.55E+01
ENSMUSG00000012405	Rpl15	4.92E-01	-2.03E+00	-1.02	0.0064	-2.54E+01	2.54E+01
ENSMUSG00000105287	RP23-282L6.1	2.80E-01	-3.57E+00	-1.84	0.0199	-2.53E+01	2.53E+01
ENSMUSG00000085134	Gm11659	2.33E-01	-4.30E+00	-2.10	0.0290	-2.53E+01	2.53E+01
ENSMUSG00000022018	Rgcc	3.62E+00	3.62E+00	1.86	0.0205	2.53E+01	2.53E+01
ENSMUSG00000066677	Pydc3	3.61E-01	-2.77E+00	-1.47	0.0120	-2.52E+01	2.52E+01
ENSMUSG00000059891	Tsks	4.32E+00	4.32E+00	2.11	0.0293	2.52E+01	2.52E+01
ENSMUSG00000102524	Ighv1-2	2.37E-01	-4.22E+00	-2.08	0.0279	-2.52E+01	2.52E+01
ENSMUSG00000029516	Cit	2.45E+00	2.45E+00	1.29	0.0094	2.52E+01	2.52E+01
ENSMUSG00000046636	Gm7729	1.87E-01	-5.36E+00	-2.42	0.0457	-2.51E+01	2.51E+01
ENSMUSG00000044338	Aplnr	2.43E-01	-4.12E+00	-2.04	0.0269	-2.51E+01	2.51E+01
ENSMUSG00000075304	Sp5	3.83E-01	-2.61E+00	-1.39	0.0109	-2.50E+01	2.50E+01
ENSMUSG00000038264	Sema7a	3.82E+00	3.82E+00	1.93	0.0234	2.50E+01	2.50E+01
ENSMUSG00000033082	Clec1a	2.31E-01	-4.33E+00	-2.11	0.0302	-2.49E+01	2.49E+01
ENSMUSG00000049929	Lpar4	3.83E-01	-2.61E+00	-1.38	0.0110	-2.49E+01	2.49E+01
ENSMUSG00000061210	Olfr47	5.45E+00	5.45E+00	2.45	0.0482	2.48E+01	2.48E+01
ENSMUSG00000030472	Ceacam18	4.41E+00	4.41E+00	2.14	0.0317	2.48E+01	2.48E+01
ENSMUSG00000084897	Gm14226	2.83E+00	2.83E+00	1.50	0.0131	2.47E+01	2.47E+01
ENSMUSG00000104517	Gm18407	5.34E+00	5.34E+00	2.42	0.0465	2.47E+01	2.47E+01
ENSMUSG00000075416	Gm14488	2.82E+00	2.82E+00	1.50	0.0130	2.47E+01	2.47E+01
ENSMUSG00000038402	Foxf2	3.90E-01	-2.56E+00	-1.36	0.0108	-2.47E+01	2.47E+01
ENSMUSG00000030491	Tdrd12	2.71E+00	2.71E+00	1.44	0.0121	2.47E+01	2.47E+01
ENSMUSG00000073491	Pydc4	3.40E-01	-2.94E+00	-1.56	0.0142	-2.47E+01	2.47E+01
ENSMUSG00000021567	Nkd2	3.20E-01	-3.12E+00	-1.64	0.0160	-2.47E+01	2.47E+01
ENSMUSG00000027832	Ptx3	3.12E+00	3.12E+00	1.64	0.0160	2.46E+01	2.46E+01
ENSMUSG00000093590	Gm20643	2.05E-01	-4.88E+00	-2.29	0.0392	-2.46E+01	2.46E+01

ENSMUSG00000023829	Slc22a1	3.52E-01	-2.84E+00	-1.51	0.0133	-2.46E+01	2.46E+01
ENSMUSG00000092051	Gm17229	4.57E-01	-2.19E+00	-1.13	0.0079	-2.46E+01	2.46E+01
ENSMUSG00000007989	Fzd3	4.84E-01	-2.07E+00	-1.05	0.0071	-2.46E+01	2.46E+01
ENSMUSG00000025082	Vwa2	2.53E-01	-3.95E+00	-1.98	0.0258	-2.46E+01	2.46E+01
ENSMUSG00000105375	RP23-397C5.1	4.80E+00	4.80E+00	2.26	0.0381	2.46E+01	2.46E+01
ENSMUSG00000025645	Ccdc51	2.10E+00	2.10E+00	1.07	0.0073	2.45E+01	2.45E+01
ENSMUSG00000037708	Spag6l	3.45E+00	3.45E+00	1.79	0.0197	2.45E+01	2.45E+01
ENSMUSG00000078787	Cyp2t4	2.44E+00	2.44E+00	1.29	0.0099	2.45E+01	2.45E+01
ENSMUSG00000085631	9630028H03Rik	5.21E+00	5.21E+00	2.38	0.0452	2.45E+01	2.45E+01
ENSMUSG00000085042	Abhd11os	4.20E-01	-2.38E+00	-1.25	0.0095	-2.45E+01	2.45E+01
ENSMUSG00000092418	Gm20406	2.71E+00	2.71E+00	1.44	0.0123	2.44E+01	2.44E+01
ENSMUSG00000042804	Gpr153	4.06E-01	-2.46E+00	-1.30	0.0103	-2.43E+01	2.43E+01
ENSMUSG00000049420	Tmem200a	2.46E-01	-4.06E+00	-2.02	0.0281	-2.42E+01	2.42E+01
ENSMUSG00000080384	Gm22105	2.86E-01	-3.50E+00	-1.81	0.0211	-2.41E+01	2.41E+01
ENSMUSG00000032352	Lrrc1	4.99E-01	-2.00E+00	-1.00	0.0069	-2.41E+01	2.41E+01
ENSMUSG00000019916	P4ha1	4.42E-01	-2.26E+00	-1.18	0.0088	-2.40E+01	2.40E+01
ENSMUSG00000052767	Gm12703	3.33E+00	3.33E+00	1.73	0.0192	2.40E+01	2.40E+01
ENSMUSG00000078817	Nlrp12	3.07E+00	3.07E+00	1.62	0.0164	2.40E+01	2.40E+01
ENSMUSG00000038422	Hdhd3	4.47E-01	-2.24E+00	-1.16	0.0087	-2.40E+01	2.40E+01
ENSMUSG00000029375	Cxcl15	4.34E-01	-2.30E+00	-1.20	0.0092	-2.40E+01	2.40E+01
ENSMUSG00000106350	RP23-307I19.5	4.49E+00	4.49E+00	2.17	0.0352	2.39E+01	2.39E+01
ENSMUSG00000099338	2810030D12Rik	2.28E-01	-4.39E+00	-2.14	0.0337	-2.39E+01	2.39E+01
ENSMUSG00000106411	RP23-342M4.6	2.88E-01	-3.47E+00	-1.79	0.0212	-2.38E+01	2.38E+01
ENSMUSG00000072621	Slfn10-ps	2.93E+00	2.93E+00	1.55	0.0151	2.38E+01	2.38E+01
ENSMUSG00000022240	Ctnnd2	3.76E-01	-2.66E+00	-1.41	0.0125	-2.38E+01	2.38E+01
ENSMUSG00000102198	Gm38173	3.45E+00	3.45E+00	1.78	0.0210	2.38E+01	2.38E+01
ENSMUSG00000083505	Gm7541	3.56E+00	3.56E+00	1.83	0.0224	2.38E+01	2.38E+01
ENSMUSG00000052426	Gm9877	4.03E-01	-2.48E+00	-1.31	0.0110	-2.37E+01	2.37E+01
ENSMUSG00000020682	Mmp28	3.56E-01	-2.81E+00	-1.49	0.0141	-2.37E+01	2.37E+01
ENSMUSG00000030790	Adm	2.48E+00	2.48E+00	1.31	0.0110	2.36E+01	2.36E+01
ENSMUSG00000079001	4930404H24Rik	2.80E-01	-3.57E+00	-1.84	0.0231	-2.35E+01	2.35E+01
ENSMUSG00000032502	Stac	3.37E-01	-2.97E+00	-1.57	0.0160	-2.35E+01	2.35E+01
ENSMUSG00000083100	Gm6044	2.75E+00	2.75E+00	1.46	0.0137	2.34E+01	2.34E+01
ENSMUSG00000104696	RP23-354G14.1	3.35E-01	-2.99E+00	-1.58	0.0163	-2.34E+01	2.34E+01
ENSMUSG00000070469	Adamtsl3	3.69E-01	-2.71E+00	-1.44	0.0134	-2.34E+01	2.34E+01
ENSMUSG00000022941	Ripply3	3.42E-01	-2.93E+00	-1.55	0.0157	-2.34E+01	2.34E+01
ENSMUSG00000056313	1810011O10Rik	2.64E-01	-3.79E+00	-1.92	0.0264	-2.33E+01	2.33E+01
ENSMUSG00000029602	Rasal1	2.11E-01	-4.74E+00	-2.24	0.0413	-2.33E+01	2.33E+01
ENSMUSG00000023011	Faim2	3.12E-01	-3.21E+00	-1.68	0.0189	-2.33E+01	2.33E+01
ENSMUSG00000085883	Gm3848	2.62E-01	-3.81E+00	-1.93	0.0268	-2.33E+01	2.33E+01
ENSMUSG00000086133	Gm16331	2.70E-01	-3.70E+00	-1.89	0.0253	-2.32E+01	2.32E+01
ENSMUSG00000097774	Gm26565	4.46E-01	-2.24E+00	-1.17	0.0093	-2.32E+01	2.32E+01
ENSMUSG00000067928	Zfp760	3.60E-01	-2.78E+00	-1.47	0.0143	-2.32E+01	2.32E+01
ENSMUSG00000092077	Olfr101	3.43E+00	3.43E+00	1.78	0.0218	2.32E+01	2.32E+01
ENSMUSG00000082190	Gm13641	2.46E-01	-4.07E+00	-2.02	0.0307	-2.32E+01	2.32E+01
ENSMUSG00000103748	Gm38243	2.80E-01	-3.57E+00	-1.84	0.0237	-2.32E+01	2.32E+01
ENSMUSG00000073971	Olfr554	2.84E-01	-3.52E+00	-1.81	0.0230	-2.32E+01	2.32E+01
ENSMUSG00000106204	RP24-388K6.6	2.59E-01	-3.86E+00	-1.95	0.0276	-2.32E+01	2.32E+01
ENSMUSG00000106206	RP23-4P1.2	3.96E-01	-2.53E+00	-1.34	0.0120	-2.31E+01	2.31E+01
ENSMUSG00000047146	Tet1	3.50E-01	-2.86E+00	-1.52	0.0154	-2.30E+01	2.30E+01
ENSMUSG00000024665	Fads2	3.78E-01	-2.64E+00	-1.40	0.0132	-2.30E+01	2.30E+01
ENSMUSG00000084192	Gm13282	3.54E+00	3.54E+00	1.83	0.0238	2.30E+01	2.30E+01
ENSMUSG00000084717	Gm24630	2.04E-01	-4.90E+00	-2.29	0.0455	-2.30E+01	2.30E+01
ENSMUSG00000030865	Chp2	2.70E-01	-3.70E+00	-1.89	0.0260	-2.30E+01	2.30E+01
ENSMUSG00000001021	S100a3	2.83E+00	2.83E+00	1.50	0.0152	2.30E+01	2.30E+01
ENSMUSG00000047037	Nipa1	4.70E-01	-2.13E+00	-1.09	0.0086	-2.29E+01	2.29E+01
ENSMUSG00000063698	Sfxn4	3.70E-01	-2.70E+00	-1.43	0.0139	-2.29E+01	2.29E+01
ENSMUSG00000006777	Krt23	3.19E-01	-3.14E+00	-1.65	0.0188	-2.29E+01	2.29E+01
ENSMUSG00000100090	Gm29199	4.54E-01	-2.20E+00	-1.14	0.0093	-2.29E+01	2.29E+01
ENSMUSG00000026575	Nme7	4.94E-01	-2.02E+00	-1.02	0.0078	-2.29E+01	2.29E+01
ENSMUSG00000031138	F9	2.73E-01	-3.66E+00	-1.87	0.0257	-2.29E+01	2.29E+01
ENSMUSG00000106435	RP23-131D6.3	3.47E+00	3.47E+00	1.79	0.0230	2.29E+01	2.29E+01
ENSMUSG00000096975	Gm16386	4.36E-01	-2.29E+00	-1.20	0.0101	-2.28E+01	2.28E+01
ENSMUSG00000073590	3222401L13Rik	4.02E-01	-2.49E+00	-1.32	0.0119	-2.28E+01	2.28E+01
ENSMUSG00000001334	Fndc5	3.15E-01	-3.18E+00	-1.67	0.0194	-2.28E+01	2.28E+01
ENSMUSG00000037686	Aspg	3.08E+00	3.08E+00	1.62	0.0183	2.28E+01	2.28E+01
ENSMUSG00000037754	Ppp1r16b	3.85E-01	-2.60E+00	-1.38	0.0130	-2.28E+01	2.28E+01

ENSMUSG00000072294	Klf12	3.51E-01	-2.85E+00	-1.51	0.0156	-2.28E+01	2.28E+01
ENSMUSG00000057123	Gja5	2.48E-01	-4.04E+00	-2.01	0.0315	-2.27E+01	2.27E+01
ENSMUSG00000050541	Adra1b	3.15E-01	-3.17E+00	-1.67	0.0195	-2.27E+01	2.27E+01
ENSMUSG00000083496	Gm11263	2.11E-01	-4.73E+00	-2.24	0.0435	-2.27E+01	2.27E+01
ENSMUSG00000033453	Adamts15	3.40E+00	3.40E+00	1.77	0.0225	2.27E+01	2.27E+01
ENSMUSG00000028347	Tmeff1	4.13E-01	-2.42E+00	-1.28	0.0115	-2.26E+01	2.26E+01
ENSMUSG00000037892	Pcdh18	3.34E-01	-3.00E+00	-1.58	0.0175	-2.26E+01	2.26E+01
ENSMUSG00000098016	Gm9211	2.90E+00	2.90E+00	1.53	0.0164	2.26E+01	2.26E+01
ENSMUSG00000004035	Gstm7	4.66E-01	-2.15E+00	-1.10	0.0090	-2.26E+01	2.26E+01
ENSMUSG00000047383	Gm33589	3.22E+00	3.22E+00	1.69	0.0203	2.26E+01	2.26E+01
ENSMUSG00000020577	Tspan13	3.43E-01	-2.92E+00	-1.55	0.0167	-2.26E+01	2.26E+01
ENSMUSG00000059900	Tmem40	2.39E+00	2.39E+00	1.26	0.0112	2.25E+01	2.25E+01
ENSMUSG00000066235	Pomgnt2	3.65E-01	-2.74E+00	-1.45	0.0148	-2.25E+01	2.25E+01
ENSMUSG00000089601	Gm22094	2.84E-01	-3.53E+00	-1.82	0.0246	-2.25E+01	2.25E+01
ENSMUSG00000101798	Gm28995	2.11E-01	-4.73E+00	-2.24	0.0444	-2.25E+01	2.25E+01
ENSMUSG00000086707	Gm12088	4.24E+00	4.24E+00	2.08	0.0356	2.25E+01	2.25E+01
ENSMUSG00000028327	Stra6l	2.15E+00	2.15E+00	1.11	0.0092	2.25E+01	2.25E+01
ENSMUSG00000045871	Slitrk6	3.53E-01	-2.83E+00	-1.50	0.0159	-2.24E+01	2.24E+01
ENSMUSG00000063130	Calml3	4.88E-01	-2.05E+00	-1.04	0.0084	-2.24E+01	2.24E+01
ENSMUSG00000052403	Fcnaos	2.68E+00	2.68E+00	1.42	0.0144	2.24E+01	2.24E+01
ENSMUSG00000064343	mt-Tq	2.78E-01	-3.60E+00	-1.85	0.0260	-2.23E+01	2.23E+01
ENSMUSG00000031465	Angpt2	4.62E-01	-2.16E+00	-1.11	0.0094	-2.23E+01	2.23E+01
ENSMUSG00000033826	Dnah8	2.99E-01	-3.35E+00	-1.74	0.0225	-2.23E+01	2.23E+01
ENSMUSG00000058260	Serpina9	3.38E-01	-2.96E+00	-1.57	0.0177	-2.23E+01	2.23E+01
ENSMUSG00000105062	RP23-114F4.3	4.55E-01	-2.20E+00	-1.14	0.0097	-2.23E+01	2.23E+01
ENSMUSG00000084748	Gm25289	4.75E+00	4.75E+00	2.25	0.0457	2.22E+01	2.22E+01
ENSMUSG00000019528	Gyg	4.33E-01	-2.31E+00	-1.21	0.0108	-2.22E+01	2.22E+01
ENSMUSG00000004371	Il11	3.96E+00	3.96E+00	1.99	0.0318	2.22E+01	2.22E+01
ENSMUSG00000052566	Hook2	2.79E+00	2.79E+00	1.48	0.0159	2.22E+01	2.22E+01
ENSMUSG00000005973	Rcn1	4.34E-01	-2.31E+00	-1.20	0.0108	-2.22E+01	2.22E+01
ENSMUSG00000105261	RP23-102H7.9	3.16E+00	3.16E+00	1.66	0.0205	2.21E+01	2.21E+01
ENSMUSG00000048349	Pou4f1	3.42E-01	-2.92E+00	-1.55	0.0175	-2.21E+01	2.21E+01
ENSMUSG00000105926	RP24-176O2.4	2.20E+00	2.20E+00	1.14	0.0099	2.21E+01	2.21E+01
ENSMUSG00000020303	Stc2	4.56E-01	-2.19E+00	-1.13	0.0099	-2.21E+01	2.21E+01
ENSMUSG00000047528	Als2cr12	3.92E+00	3.92E+00	1.97	0.0316	2.21E+01	2.21E+01
ENSMUSG00000026473	Glul	2.77E+00	2.77E+00	1.47	0.0158	2.21E+01	2.21E+01
ENSMUSG00000100764	Gm29155	3.05E+00	3.05E+00	1.61	0.0193	2.20E+01	2.20E+01
ENSMUSG00000041991	Hrnr	2.71E+00	2.71E+00	1.44	0.0154	2.19E+01	2.19E+01
ENSMUSG00000030693	Klk10	3.00E-01	-3.33E+00	-1.74	0.0231	-2.19E+01	2.19E+01
ENSMUSG00000085876	Gm12409	2.65E-01	-3.77E+00	-1.92	0.0297	-2.19E+01	2.19E+01
ENSMUSG00000062524	Ncr1	2.18E-01	-4.58E+00	-2.20	0.0438	-2.19E+01	2.19E+01
ENSMUSG00000043488	Frmd8os	3.25E+00	3.25E+00	1.70	0.0221	2.19E+01	2.19E+01
ENSMUSG00000025196	Cpn1	4.94E-01	-2.02E+00	-1.02	0.0086	-2.19E+01	2.19E+01
ENSMUSG00000081320	Gm15034	2.60E-01	-3.85E+00	-1.94	0.0310	-2.18E+01	2.18E+01
ENSMUSG00000091492	Gm17025	2.69E-01	-3.71E+00	-1.89	0.0291	-2.18E+01	2.18E+01
ENSMUSG00000096847	Tmem151b	4.36E+00	4.36E+00	2.12	0.0403	2.17E+01	2.17E+01
ENSMUSG00000035407	Kank4	2.25E-01	-4.45E+00	-2.15	0.0422	-2.17E+01	2.17E+01
ENSMUSG00000037206	Islr	4.67E-01	-2.14E+00	-1.10	0.0098	-2.17E+01	2.17E+01
ENSMUSG00000036019	Tmtc2	3.54E-01	-2.82E+00	-1.50	0.0171	-2.16E+01	2.16E+01
ENSMUSG00000003500	Impdh1	3.33E-01	-3.00E+00	-1.59	0.0193	-2.16E+01	2.16E+01
ENSMUSG00000106498	RP23-350E1.2	3.74E-01	-2.68E+00	-1.42	0.0154	-2.16E+01	2.16E+01
ENSMUSG00000056468	5730596B20Rik	3.60E-01	-2.78E+00	-1.47	0.0165	-2.16E+01	2.16E+01
ENSMUSG00000020811	Wscd1	2.89E-01	-3.45E+00	-1.79	0.0256	-2.16E+01	2.16E+01
ENSMUSG00000107264	AC127262.1	2.42E-01	-4.13E+00	-2.05	0.0366	-2.16E+01	2.16E+01
ENSMUSG00000066842	Hmcn1	3.42E-01	-2.93E+00	-1.55	0.0184	-2.16E+01	2.16E+01
ENSMUSG00000031576	Kcnu1	3.49E+00	3.49E+00	1.80	0.0263	2.15E+01	2.15E+01
ENSMUSG00000076473	Trbv16	3.02E-01	-3.31E+00	-1.73	0.0236	-2.15E+01	2.15E+01
ENSMUSG00000038194	Lhb	4.22E-01	-2.37E+00	-1.24	0.0121	-2.15E+01	2.15E+01
ENSMUSG00000032377	Plscr4	3.48E-01	-2.88E+00	-1.52	0.0179	-2.15E+01	2.15E+01
ENSMUSG00000085633	Bloc1s6os	3.04E+00	3.04E+00	1.60	0.0201	2.14E+01	2.14E+01
ENSMUSG00000072972	Adam4	2.64E-01	-3.79E+00	-1.92	0.0313	-2.14E+01	2.14E+01
ENSMUSG00000022615	Tymp	3.46E-01	-2.89E+00	-1.53	0.0182	-2.14E+01	2.14E+01
ENSMUSG00000072974	Gm4787	2.82E-01	-3.55E+00	-1.83	0.0274	-2.14E+01	2.14E+01
ENSMUSG00000103811	Gm38004	2.78E+00	2.78E+00	1.47	0.0168	2.14E+01	2.14E+01
ENSMUSG00000038539	Atf5	4.44E-01	-2.25E+00	-1.17	0.0111	-2.14E+01	2.14E+01
ENSMUSG00000019647	Sema6a	2.92E-01	-3.43E+00	-1.78	0.0257	-2.14E+01	2.14E+01
ENSMUSG00000044206	Vsig4	4.15E+00	4.15E+00	2.05	0.0377	2.13E+01	2.13E+01

ENSMUSG00000020312	Shc2	3.92E-01	-2.55E+00	-1.35	0.0143	-2.13E+01	2.13E+01
ENSMUSG00000032034	Kcnj5	4.14E-01	-2.41E+00	-1.27	0.0128	-2.13E+01	2.13E+01
ENSMUSG00000071042	Rasgrp3	3.83E-01	-2.61E+00	-1.39	0.0150	-2.13E+01	2.13E+01
ENSMUSG00000052957	Gas1	4.25E-01	-2.36E+00	-1.24	0.0122	-2.13E+01	2.13E+01
ENSMUSG00000104869	RP23-54L16.2	3.72E+00	3.72E+00	1.89	0.0305	2.13E+01	2.13E+01
ENSMUSG00000026656	Fcgr2b	3.62E+00	3.62E+00	1.86	0.0289	2.13E+01	2.13E+01
ENSMUSG00000097332	2610020F03Rik	2.41E-01	-4.15E+00	-2.05	0.0381	-2.13E+01	2.13E+01
ENSMUSG00000079669	Gm17396	4.17E-01	-2.40E+00	-1.26	0.0127	-2.12E+01	2.12E+01
ENSMUSG00000026394	Atp6v1g3	2.69E-01	-3.71E+00	-1.89	0.0305	-2.12E+01	2.12E+01
ENSMUSG00000026456	Cyb5r1	3.93E-01	-2.55E+00	-1.35	0.0144	-2.12E+01	2.12E+01
ENSMUSG00000103732	Gm38315	3.75E-01	-2.67E+00	-1.42	0.0158	-2.12E+01	2.12E+01
ENSMUSG00000097612	Gm26509	2.43E-01	-4.11E+00	-2.04	0.0375	-2.12E+01	2.12E+01
ENSMUSG00000070331	Qrich2	4.10E+00	4.10E+00	2.04	0.0374	2.12E+01	2.12E+01
ENSMUSG00000076490	Trbc1	4.90E-01	-2.04E+00	-1.03	0.0093	-2.12E+01	2.12E+01
ENSMUSG00000089437	Gm23199	4.95E-01	-2.02E+00	-1.01	0.0091	-2.12E+01	2.12E+01
ENSMUSG00000029211	Gabra4	3.63E-01	-2.76E+00	-1.46	0.0170	-2.12E+01	2.12E+01
ENSMUSG00000030465	Psd3	4.24E-01	-2.36E+00	-1.24	0.0125	-2.11E+01	2.11E+01
ENSMUSG00000020598	Nrcam	2.19E-01	-4.56E+00	-2.19	0.0466	-2.11E+01	2.11E+01
ENSMUSG00000023132	Gzma	2.21E-01	-4.53E+00	-2.18	0.0461	-2.11E+01	2.11E+01
ENSMUSG00000033508	Asprv1	3.77E+00	3.77E+00	1.91	0.0319	2.11E+01	2.11E+01
ENSMUSG00000046818	Ddit4l	4.70E-01	-2.13E+00	-1.09	0.0102	-2.11E+01	2.11E+01
ENSMUSG00000009900	Wnt3a	3.91E-01	-2.56E+00	-1.36	0.0147	-2.11E+01	2.11E+01
ENSMUSG00000047798	Cd300lf	3.84E+00	3.84E+00	1.94	0.0333	2.11E+01	2.11E+01
ENSMUSG00000042289	Hsd3b7	4.12E-01	-2.43E+00	-1.28	0.0134	-2.10E+01	2.10E+01
ENSMUSG00000097681	Gm26643	2.67E+00	2.67E+00	1.42	0.0162	2.10E+01	2.10E+01
ENSMUSG00000087690	Gm16031	3.30E-01	-3.03E+00	-1.60	0.0210	-2.09E+01	2.09E+01
ENSMUSG00000063522	2010109I03Rik	3.17E+00	3.17E+00	1.66	0.0230	2.09E+01	2.09E+01
ENSMUSG00000041594	Tmtc4	3.15E-01	-3.17E+00	-1.67	0.0231	-2.09E+01	2.09E+01
ENSMUSG00000037982	Gm9725	2.93E+00	2.93E+00	1.55	0.0198	2.08E+01	2.08E+01
ENSMUSG00000019256	Ahr	4.06E-01	-2.46E+00	-1.30	0.0140	-2.08E+01	2.08E+01
ENSMUSG00000031877	Ces2g	4.07E-01	-2.46E+00	-1.30	0.0139	-2.08E+01	2.08E+01
ENSMUSG00000103002	Gm38075	4.49E-01	-2.23E+00	-1.16	0.0115	-2.08E+01	2.08E+01
ENSMUSG00000063681	Crb1	3.12E-01	-3.20E+00	-1.68	0.0237	-2.08E+01	2.08E+01
ENSMUSG00000097303	3110083C13Rik	2.70E-01	-3.71E+00	-1.89	0.0319	-2.08E+01	2.08E+01
ENSMUSG00000035615	Frmpd1	3.30E-01	-3.03E+00	-1.60	0.0212	-2.08E+01	2.08E+01
ENSMUSG00000081731	Calr-ps	4.91E-01	-2.04E+00	-1.03	0.0096	-2.07E+01	2.07E+01
ENSMUSG00000049872	Fam26e	3.95E-01	-2.53E+00	-1.34	0.0149	-2.07E+01	2.07E+01
ENSMUSG00000052212	Cd177	3.06E+00	3.06E+00	1.62	0.0219	2.07E+01	2.07E+01
ENSMUSG00000037913	Tmem156	3.40E+00	3.40E+00	1.77	0.0271	2.07E+01	2.07E+01
ENSMUSG00000047631	Apof	3.40E+00	3.40E+00	1.76	0.0270	2.07E+01	2.07E+01
ENSMUSG00000105512	RP23-199B2.8	3.45E+00	3.45E+00	1.79	0.0279	2.06E+01	2.06E+01
ENSMUSG00000027442	Cst8	2.21E-01	-4.52E+00	-2.18	0.0482	-2.06E+01	2.06E+01
ENSMUSG00000030357	Fkbp4	4.72E-01	-2.12E+00	-1.08	0.0106	-2.06E+01	2.06E+01
ENSMUSG00000097756	A730056A06Rik	2.70E-01	-3.71E+00	-1.89	0.0325	-2.06E+01	2.06E+01
ENSMUSG00000031489	Adrb3	2.65E-01	-3.77E+00	-1.92	0.0338	-2.05E+01	2.05E+01
ENSMUSG00000098708	Gm27252	3.86E+00	3.86E+00	1.95	0.0354	2.05E+01	2.05E+01
ENSMUSG00000099879	Gm29123	3.66E+00	3.66E+00	1.87	0.0321	2.04E+01	2.04E+01
ENSMUSG00000031444	F10	2.49E+00	2.49E+00	1.32	0.0149	2.04E+01	2.04E+01
ENSMUSG00000083500	Gm15470	3.83E-01	-2.61E+00	-1.38	0.0163	-2.04E+01	2.04E+01
ENSMUSG00000070527	Mkrn3	4.18E-01	-2.39E+00	-1.26	0.0137	-2.04E+01	2.04E+01
ENSMUSG00000016327	Atp1b4	4.04E+00	4.04E+00	2.01	0.0393	2.04E+01	2.04E+01
ENSMUSG00000047959	Kcna3	4.62E-01	-2.16E+00	-1.11	0.0113	-2.04E+01	2.04E+01
ENSMUSG00000086952	Gm12596	3.21E+00	3.21E+00	1.68	0.0248	2.04E+01	2.04E+01
ENSMUSG00000100522	Gm19280	2.56E+00	2.56E+00	1.35	0.0158	2.03E+01	2.03E+01
ENSMUSG00000051599	Pcdhb2	2.85E-01	-3.51E+00	-1.81	0.0299	-2.03E+01	2.03E+01
ENSMUSG00000089242	Gm24056	2.25E-01	-4.45E+00	-2.15	0.0481	-2.03E+01	2.03E+01
ENSMUSG00000044986	Tst	3.95E-01	-2.53E+00	-1.34	0.0157	-2.02E+01	2.02E+01
ENSMUSG00000048728	Zfp454	2.45E-01	-4.08E+00	-2.03	0.0406	-2.02E+01	2.02E+01
ENSMUSG00000081217	Gm15268	3.18E-01	-3.14E+00	-1.65	0.0242	-2.02E+01	2.02E+01
ENSMUSG00000105228	RP23-180G16.2	4.29E+00	4.29E+00	2.10	0.0451	2.02E+01	2.02E+01
ENSMUSG00000097021	4933433G15Rik	4.30E+00	4.30E+00	2.10	0.0453	2.02E+01	2.02E+01
ENSMUSG00000030084	Plxna1	4.93E-01	-2.03E+00	-1.02	0.0101	-2.02E+01	2.02E+01
ENSMUSG00000082508	Rpl15-ps4	2.30E-01	-4.35E+00	-2.12	0.0464	-2.02E+01	2.02E+01
ENSMUSG00000032890	Rims3	3.29E+00	3.29E+00	1.72	0.0267	2.01E+01	2.01E+01
ENSMUSG00000104626	RP23-10F17.4	3.91E-01	-2.56E+00	-1.36	0.0161	-2.01E+01	2.01E+01
ENSMUSG00000041596	Nlrp5-ps	3.58E-01	-2.79E+00	-1.48	0.0193	-2.01E+01	2.01E+01
ENSMUSG00000025197	Cyp2c44	2.23E-01	-4.48E+00	-2.16	0.0497	-2.01E+01	2.01E+01

ENSMUSG00000107216	RP23-353L10.2	3.64E-01	-2.75E+00	-1.46	0.0187	-2.01E+01	2.01E+01
ENSMUSG00000107148	RP23-114M24.1	4.20E-01	-2.38E+00	-1.25	0.0140	-2.01E+01	2.01E+01
ENSMUSG00000030641	Ddias	4.22E+00	4.22E+00	2.08	0.0441	2.01E+01	2.01E+01
ENSMUSG00000068270	Shroom4	4.92E-01	-2.03E+00	-1.02	0.0102	-2.01E+01	2.01E+01
ENSMUSG00000054477	Kcnn2	2.91E-01	-3.43E+00	-1.78	0.0293	-2.00E+01	2.00E+01
ENSMUSG00000025582	Nptx1	3.95E-01	-2.53E+00	-1.34	0.0160	-2.00E+01	2.00E+01
ENSMUSG00000043631	Ecm2	3.11E-01	-3.22E+00	-1.69	0.0259	-2.00E+01	2.00E+01
ENSMUSG00000102700	Gm38312	3.86E-01	-2.59E+00	-1.37	0.0168	-2.00E+01	2.00E+01
ENSMUSG00000090124	Ugt1a7c	2.05E+00	2.05E+00	1.04	0.0106	2.00E+01	2.00E+01
ENSMUSG00000074340	Ovgp1	4.23E+00	4.23E+00	2.08	0.0450	1.99E+01	1.99E+01
ENSMUSG00000089712	Gm15889	2.72E+00	2.72E+00	1.45	0.0187	1.99E+01	1.99E+01
ENSMUSG00000027954	Efna1	4.55E-01	-2.20E+00	-1.14	0.0123	-1.98E+01	1.98E+01
ENSMUSG00000031698	Mylk3	3.08E-01	-3.24E+00	-1.70	0.0269	-1.98E+01	1.98E+01
ENSMUSG00000084755	Gm16276	2.57E-01	-3.89E+00	-1.96	0.0388	-1.97E+01	1.97E+01
ENSMUSG00000107312	RP23-319P12.1	4.07E-01	-2.45E+00	-1.30	0.0155	-1.97E+01	1.97E+01
ENSMUSG00000087721	Gm26280	3.84E-01	-2.61E+00	-1.38	0.0175	-1.97E+01	1.97E+01
ENSMUSG00000055313	Pgbd1	3.63E-01	-2.75E+00	-1.46	0.0196	-1.97E+01	1.97E+01
ENSMUSG00000030302	Atp2b2	3.91E-01	-2.56E+00	-1.35	0.0169	-1.97E+01	1.97E+01
ENSMUSG00000025578	Cbx8	3.82E-01	-2.62E+00	-1.39	0.0177	-1.97E+01	1.97E+01
ENSMUSG00000059588	Calcrl	2.93E-01	-3.41E+00	-1.77	0.0302	-1.97E+01	1.97E+01
ENSMUSG00000102277	A130050O07Rik	4.13E-01	-2.42E+00	-1.27	0.0152	-1.97E+01	1.97E+01
ENSMUSG00000022220	Adcy4	3.75E-01	-2.66E+00	-1.41	0.0184	-1.96E+01	1.96E+01
ENSMUSG00000026640	Plxna2	2.99E+00	2.99E+00	1.58	0.0231	1.96E+01	1.96E+01
ENSMUSG00000059791	Nrm	2.61E+00	2.61E+00	1.38	0.0176	1.96E+01	1.96E+01
ENSMUSG00000097884	Gm26543	2.68E-01	-3.74E+00	-1.90	0.0362	-1.96E+01	1.96E+01
ENSMUSG00000100825	Itpa-ps2	2.98E-01	-3.35E+00	-1.75	0.0293	-1.96E+01	1.96E+01
ENSMUSG00000028678	Kif2c	2.31E+00	2.31E+00	1.21	0.0140	1.96E+01	1.96E+01
ENSMUSG00000078532	Nkain1	3.36E-01	-2.98E+00	-1.57	0.0233	-1.95E+01	1.95E+01
ENSMUSG00000019893	Ros1	2.79E-01	-3.59E+00	-1.84	0.0338	-1.95E+01	1.95E+01
ENSMUSG00000106107	RP23-381G13.3	3.38E+00	3.38E+00	1.76	0.0300	1.95E+01	1.95E+01
ENSMUSG00000105156	RP23-258C13.1	2.89E-01	-3.46E+00	-1.79	0.0315	-1.95E+01	1.95E+01
ENSMUSG00000030116	Mfap5	4.49E-01	-2.22E+00	-1.15	0.0131	-1.94E+01	1.94E+01
ENSMUSG00000087013	2610027K06Rik	4.35E-01	-2.30E+00	-1.20	0.0140	-1.94E+01	1.94E+01
ENSMUSG00000042988	Notum	3.69E-01	-2.71E+00	-1.44	0.0195	-1.94E+01	1.94E+01
ENSMUSG00000011154	1700026D08Rik	3.99E-01	-2.50E+00	-1.32	0.0166	-1.94E+01	1.94E+01
ENSMUSG00000034487	Kdelc2	4.73E-01	-2.12E+00	-1.08	0.0119	-1.94E+01	1.94E+01
ENSMUSG00000074780	Anapc15-ps	2.07E+00	2.07E+00	1.05	0.0114	1.94E+01	1.94E+01
ENSMUSG00000102232	Gm19035	2.82E-01	-3.55E+00	-1.83	0.0336	-1.94E+01	1.94E+01
ENSMUSG00000100876	2810454H06Rik	2.07E+00	2.07E+00	1.05	0.0114	1.94E+01	1.94E+01
ENSMUSG00000044244	Il20rb	3.55E+00	3.55E+00	1.83	0.0335	1.94E+01	1.94E+01
ENSMUSG00000041912	Tdrkh	3.45E-01	-2.89E+00	-1.53	0.0223	-1.94E+01	1.94E+01
ENSMUSG00000086418	Gm13362	3.40E-01	-2.94E+00	-1.56	0.0230	-1.94E+01	1.94E+01
ENSMUSG00000087575	Gm12976	4.17E+00	4.17E+00	2.06	0.0465	1.93E+01	1.93E+01
ENSMUSG00000104835	RP24-500I24.7	2.37E+00	2.37E+00	1.24	0.0150	1.93E+01	1.93E+01
ENSMUSG00000039004	Bmp6	3.35E-01	-2.99E+00	-1.58	0.0240	-1.93E+01	1.93E+01
ENSMUSG00000030979	Uros	4.24E-01	-2.36E+00	-1.24	0.0150	-1.93E+01	1.93E+01
ENSMUSG00000042379	Esm1	4.86E-01	-2.06E+00	-1.04	0.0114	-1.93E+01	1.93E+01
ENSMUSG00000071104	Ccdc110	4.72E-01	-2.12E+00	-1.08	0.0121	-1.92E+01	1.92E+01
ENSMUSG00000102494	Gm36988	3.46E-01	-2.89E+00	-1.53	0.0226	-1.92E+01	1.92E+01
ENSMUSG00000027797	Dclk1	4.38E-01	-2.28E+00	-1.19	0.0141	-1.92E+01	1.92E+01
ENSMUSG00000040724	Kcna2	3.80E-01	-2.63E+00	-1.40	0.0188	-1.92E+01	1.92E+01
ENSMUSG00000097146	Gm4211	2.01E+00	2.01E+00	1.01	0.0109	1.92E+01	1.92E+01
ENSMUSG00000046971	Pla2g4f	4.12E-01	-2.43E+00	-1.28	0.0159	-1.92E+01	1.92E+01
ENSMUSG00000031292	Cdkl5	4.09E-01	-2.44E+00	-1.29	0.0162	-1.92E+01	1.92E+01
ENSMUSG00000103791	Gm38095	3.35E-01	-2.98E+00	-1.58	0.0242	-1.92E+01	1.92E+01
ENSMUSG00000024990	Rbp4	4.60E-01	-2.17E+00	-1.12	0.0129	-1.91E+01	1.91E+01
ENSMUSG00000102249	4930423C22Rik	2.50E-01	-4.00E+00	-2.00	0.0439	-1.91E+01	1.91E+01
ENSMUSG00000015001	Oc90	4.15E+00	4.15E+00	2.05	0.0473	1.91E+01	1.91E+01
ENSMUSG00000070336	Fbxo47	2.45E+00	2.45E+00	1.29	0.0165	1.91E+01	1.91E+01
ENSMUSG00000093485	Gm20708	2.41E+00	2.41E+00	1.27	0.0159	1.91E+01	1.91E+01
ENSMUSG00000089989	Flt3l	4.98E-01	-2.01E+00	-1.01	0.0111	-1.90E+01	1.90E+01
ENSMUSG00000034579	Pla2g3	3.84E+00	3.84E+00	1.94	0.0407	1.90E+01	1.90E+01
ENSMUSG00000086863	Gm15543	2.49E-01	-4.02E+00	-2.01	0.0447	-1.90E+01	1.90E+01
ENSMUSG00000080878	Gm15618	2.99E-01	-3.34E+00	-1.74	0.0309	-1.90E+01	1.90E+01
ENSMUSG00000064215	Ifi27	4.97E-01	-2.01E+00	-1.01	0.0112	-1.90E+01	1.90E+01
ENSMUSG00000046719	Nxph3	4.25E-01	-2.35E+00	-1.24	0.0154	-1.90E+01	1.90E+01
ENSMUSG00000074743	Thbd	3.99E-01	-2.50E+00	-1.32	0.0174	-1.90E+01	1.90E+01

ENSMUSG00000000794	Kcnn3	4.66E-01	-2.14E+00	-1.10	0.0129	-1.89E+01	1.89E+01
ENSMUSG00000022048	Dpysl2	3.85E-01	-2.60E+00	-1.38	0.0189	-1.89E+01	1.89E+01
ENSMUSG00000040037	Negr1	3.74E-01	-2.68E+00	-1.42	0.0202	-1.89E+01	1.89E+01
ENSMUSG00000064063	BC048507	4.36E-01	-2.30E+00	-1.20	0.0148	-1.88E+01	1.88E+01
ENSMUSG00000016918	Sulf1	3.94E-01	-2.54E+00	-1.34	0.0182	-1.88E+01	1.88E+01
ENSMUSG00000096145	Vkorc1	4.64E-01	-2.16E+00	-1.11	0.0132	-1.87E+01	1.87E+01
ENSMUSG00000035829	Ppp1r26	3.98E-01	-2.51E+00	-1.33	0.0180	-1.87E+01	1.87E+01
ENSMUSG00000014846	Tppp3	3.53E-01	-2.83E+00	-1.50	0.0229	-1.87E+01	1.87E+01
ENSMUSG00000050762	Prss27	2.29E+00	2.29E+00	1.20	0.0151	1.87E+01	1.87E+01
ENSMUSG00000005540	Fcer2a	2.81E-01	-3.56E+00	-1.83	0.0362	-1.87E+01	1.87E+01
ENSMUSG00000051705	Senp8	2.57E-01	-3.88E+00	-1.96	0.0431	-1.87E+01	1.87E+01
ENSMUSG00000057286	St6galnac2	4.02E-01	-2.49E+00	-1.32	0.0178	-1.87E+01	1.87E+01
ENSMUSG00000098837	Gm20111	3.50E-01	-2.86E+00	-1.52	0.0235	-1.87E+01	1.87E+01
ENSMUSG00000059027	9630013D21Rik	3.79E-01	-2.64E+00	-1.40	0.0200	-1.86E+01	1.86E+01
ENSMUSG00000087060	Eldr	3.33E-01	-3.00E+00	-1.59	0.0260	-1.86E+01	1.86E+01
ENSMUSG00000101671	Gm28221	4.81E-01	-2.08E+00	-1.06	0.0125	-1.86E+01	1.86E+01
ENSMUSG00000069184	Zfp72	3.25E-01	-3.07E+00	-1.62	0.0274	-1.86E+01	1.86E+01
ENSMUSG00000081992	Gm13408	3.16E-01	-3.17E+00	-1.66	0.0292	-1.86E+01	1.86E+01
ENSMUSG00000101716	Gm12057	2.22E+00	2.22E+00	1.15	0.0144	1.85E+01	1.85E+01
ENSMUSG00000024430	Cabyr	2.39E+00	2.39E+00	1.26	0.0167	1.85E+01	1.85E+01
ENSMUSG00000059810	Rgs3	4.47E-01	-2.24E+00	-1.16	0.0146	-1.85E+01	1.85E+01
ENSMUSG00000068638	4930403O15Rik	4.86E-01	-2.06E+00	-1.04	0.0124	-1.85E+01	1.85E+01
ENSMUSG00000078816	Prkcg	4.13E+00	4.13E+00	2.05	0.0498	1.85E+01	1.85E+01
ENSMUSG00000028185	Dnase2b	3.45E-01	-2.90E+00	-1.54	0.0245	-1.85E+01	1.85E+01
ENSMUSG00000039133	9330171B17Rik	4.45E-01	-2.25E+00	-1.17	0.0147	-1.85E+01	1.85E+01
ENSMUSG00000022993	4930415O20Rik	2.82E+00	2.82E+00	1.49	0.0232	1.85E+01	1.85E+01
ENSMUSG00000014776	Nol3	4.41E-01	-2.27E+00	-1.18	0.0150	-1.85E+01	1.85E+01
ENSMUSG00000022346	Myc	2.14E+00	2.14E+00	1.10	0.0134	1.85E+01	1.85E+01
ENSMUSG00000032374	Plod2	4.81E-01	-2.08E+00	-1.06	0.0127	-1.84E+01	1.84E+01
ENSMUSG00000081496	Gm13428	3.82E+00	3.82E+00	1.93	0.0430	1.84E+01	1.84E+01
ENSMUSG00000060227	Casc4	4.42E-01	-2.26E+00	-1.18	0.0151	-1.84E+01	1.84E+01
ENSMUSG00000052544	St6galnac3	4.40E-01	-2.28E+00	-1.19	0.0153	-1.84E+01	1.84E+01
ENSMUSG00000021186	Fbln5	4.70E-01	-2.13E+00	-1.09	0.0134	-1.84E+01	1.84E+01
ENSMUSG00000096606	Gm4980	3.20E+00	3.20E+00	1.68	0.0303	1.84E+01	1.84E+01
ENSMUSG00000106244	RP23-187D22.10	4.08E-01	-2.45E+00	-1.29	0.0178	-1.83E+01	1.83E+01
ENSMUSG00000032036	Kirrel3	2.33E+00	2.33E+00	1.22	0.0162	1.83E+01	1.83E+01
ENSMUSG00000021835	Bmp4	4.50E-01	-2.22E+00	-1.15	0.0148	-1.83E+01	1.83E+01
ENSMUSG00000056203	Tas2r135	3.03E+00	3.03E+00	1.60	0.0274	1.83E+01	1.83E+01
ENSMUSG00000085689	Gm16838	2.48E+00	2.48E+00	1.31	0.0184	1.83E+01	1.83E+01
ENSMUSG00000087213	2810408I11Rik	3.76E+00	3.76E+00	1.91	0.0423	1.83E+01	1.83E+01
ENSMUSG00000073753	Gm10571	2.43E+00	2.43E+00	1.28	0.0177	1.83E+01	1.83E+01
ENSMUSG00000073758	Sh3d21	2.90E+00	2.90E+00	1.53	0.0251	1.83E+01	1.83E+01
ENSMUSG00000028020	Glrb	2.89E-01	-3.47E+00	-1.79	0.0360	-1.83E+01	1.83E+01
ENSMUSG00000049176	Frmpd4	2.28E+00	2.28E+00	1.19	0.0156	1.82E+01	1.82E+01
ENSMUSG00000103038	Gm37124	3.52E-01	-2.84E+00	-1.51	0.0243	-1.82E+01	1.82E+01
ENSMUSG00000097301	AW121686	3.22E+00	3.22E+00	1.69	0.0313	1.82E+01	1.82E+01
ENSMUSG00000027309	4930402H24Rik	3.18E-01	-3.15E+00	-1.65	0.0298	-1.82E+01	1.82E+01
ENSMUSG00000021750	Fam107a	4.01E+00	4.01E+00	2.00	0.0485	1.82E+01	1.82E+01
ENSMUSG00000073771	Btbd19	2.00E+00	2.00E+00	1.00	0.0121	1.82E+01	1.82E+01
ENSMUSG00000045672	Col27a1	3.42E-01	-2.92E+00	-1.55	0.0259	-1.82E+01	1.82E+01
ENSMUSG00000044367	Slc16a13	2.33E+00	2.33E+00	1.22	0.0165	1.82E+01	1.82E+01
ENSMUSG00000078528	Gm17068	4.27E-01	-2.34E+00	-1.23	0.0166	-1.82E+01	1.82E+01
ENSMUSG00000058073	Gm11189	2.72E-01	-3.68E+00	-1.88	0.0411	-1.81E+01	1.81E+01
ENSMUSG00000053297	AI854703	3.01E+00	3.01E+00	1.59	0.0277	1.81E+01	1.81E+01
ENSMUSG00000104123	Gm37483	2.49E-01	-4.02E+00	-2.01	0.0495	-1.81E+01	1.81E+01
ENSMUSG00000083334	Gm13579	3.63E-01	-2.76E+00	-1.46	0.0233	-1.81E+01	1.81E+01
ENSMUSG00000007039	Ddah2	3.76E-01	-2.66E+00	-1.41	0.0218	-1.80E+01	1.80E+01
ENSMUSG00000074207	Adh1	4.03E-01	-2.48E+00	-1.31	0.0191	-1.79E+01	1.79E+01
ENSMUSG00000060530	A930017M01Rik	2.68E-01	-3.74E+00	-1.90	0.0435	-1.79E+01	1.79E+01
ENSMUSG00000030554	Synm	3.53E-01	-2.83E+00	-1.50	0.0251	-1.79E+01	1.79E+01
ENSMUSG00000040969	Arhgef38	3.97E-01	-2.52E+00	-1.33	0.0198	-1.79E+01	1.79E+01
ENSMUSG00000081988	Gm15746	3.28E-01	-3.05E+00	-1.61	0.0292	-1.79E+01	1.79E+01
ENSMUSG00000080793	Cbx3-ps3	2.69E-01	-3.71E+00	-1.89	0.0432	-1.79E+01	1.79E+01
ENSMUSG00000042284	Itga1	3.66E-01	-2.73E+00	-1.45	0.0235	-1.78E+01	1.78E+01
ENSMUSG00000026459	Myog	3.82E+00	3.82E+00	1.93	0.0459	1.78E+01	1.78E+01
ENSMUSG00000092300	Cdk3-ps	3.86E+00	3.86E+00	1.95	0.0470	1.78E+01	1.78E+01
ENSMUSG00000019124	Scrn1	4.92E-01	-2.03E+00	-1.02	0.0131	-1.78E+01	1.78E+01

ENSMUSG00000024925	Rnaseh2c	3.83E-01	-2.61E+00	-1.38	0.0216	-1.78E+01	1.78E+01
ENSMUSG00000029321	Slc10a6	2.50E+00	2.50E+00	1.32	0.0198	1.78E+01	1.78E+01
ENSMUSG00000047757	Fancb	3.57E-01	-2.80E+00	-1.49	0.0250	-1.77E+01	1.77E+01
ENSMUSG00000050069	Grem2	3.87E-01	-2.59E+00	-1.37	0.0213	-1.77E+01	1.77E+01
ENSMUSG00000040740	Slc25a34	3.81E+00	3.81E+00	1.93	0.0463	1.77E+01	1.77E+01
ENSMUSG00000092097	Gm5819	2.67E-01	-3.74E+00	-1.90	0.0447	-1.77E+01	1.77E+01
ENSMUSG00000042708	Shcbp1l	3.58E+00	3.58E+00	1.84	0.0409	1.77E+01	1.77E+01
ENSMUSG00000083609	Gm16007	2.60E-01	-3.85E+00	-1.94	0.0472	-1.77E+01	1.77E+01
ENSMUSG00000101693	Gm19461	4.59E-01	-2.18E+00	-1.12	0.0153	-1.76E+01	1.76E+01
ENSMUSG00000020160	Meis1	4.44E-01	-2.25E+00	-1.17	0.0163	-1.76E+01	1.76E+01
ENSMUSG00000025153	Fasn	4.40E-01	-2.27E+00	-1.18	0.0166	-1.76E+01	1.76E+01
ENSMUSG00000086500	Gm15875	3.21E-01	-3.12E+00	-1.64	0.0314	-1.76E+01	1.76E+01
ENSMUSG00000038009	Dnajc22	2.77E-01	-3.61E+00	-1.85	0.0421	-1.76E+01	1.76E+01
ENSMUSG00000078868	Gm14412	3.60E-01	-2.78E+00	-1.47	0.0250	-1.76E+01	1.76E+01
ENSMUSG00000047501	Cldn4	2.60E+00	2.60E+00	1.38	0.0220	1.76E+01	1.76E+01
ENSMUSG00000087507	Gm16141	3.97E-01	-2.52E+00	-1.33	0.0206	-1.75E+01	1.75E+01
ENSMUSG00000027424	Mgme1	4.62E-01	-2.17E+00	-1.12	0.0153	-1.75E+01	1.75E+01
ENSMUSG00000034533	Scn10a	3.18E-01	-3.14E+00	-1.65	0.0323	-1.75E+01	1.75E+01
ENSMUSG00000026888	Grb14	4.76E-01	-2.10E+00	-1.07	0.0145	-1.75E+01	1.75E+01
ENSMUSG00000018672	Copz2	4.84E-01	-2.07E+00	-1.05	0.0140	-1.75E+01	1.75E+01
ENSMUSG00000000628	Hk2	2.14E+00	2.14E+00	1.10	0.0150	1.75E+01	1.75E+01
ENSMUSG00000097744	D030040B21Rik	3.67E+00	3.67E+00	1.88	0.0442	1.74E+01	1.74E+01
ENSMUSG00000022056	Adam7	3.56E-01	-2.81E+00	-1.49	0.0260	-1.74E+01	1.74E+01
ENSMUSG00000045075	Gm9796	3.60E+00	3.60E+00	1.85	0.0427	1.74E+01	1.74E+01
ENSMUSG00000030443	Zfp583	2.74E-01	-3.66E+00	-1.87	0.0441	-1.74E+01	1.74E+01
ENSMUSG00000041134	Cyyr1	4.63E-01	-2.16E+00	-1.11	0.0154	-1.74E+01	1.74E+01
ENSMUSG00000072640	Lyrm9	4.97E-01	-2.01E+00	-1.01	0.0134	-1.74E+01	1.74E+01
ENSMUSG00000086877	A230072C01Rik	4.72E-01	-2.12E+00	-1.08	0.0149	-1.74E+01	1.74E+01
ENSMUSG00000106515	RP23-473G18.1	3.05E-01	-3.28E+00	-1.71	0.0355	-1.74E+01	1.74E+01
ENSMUSG00000104719	RP23-431M7.4	2.41E+00	2.41E+00	1.27	0.0193	1.74E+01	1.74E+01
ENSMUSG00000104684	RP24-270K7.3	3.60E-01	-2.78E+00	-1.47	0.0256	-1.74E+01	1.74E+01
ENSMUSG00000018411	Mapt	3.02E-01	-3.32E+00	-1.73	0.0365	-1.73E+01	1.73E+01
ENSMUSG00000020461	Clhc1	3.58E+00	3.58E+00	1.84	0.0427	1.73E+01	1.73E+01
ENSMUSG00000080115	Mettl21b	2.37E+00	2.37E+00	1.25	0.0187	1.73E+01	1.73E+01
ENSMUSG00000029477	Morn3	3.61E-01	-2.77E+00	-1.47	0.0255	-1.73E+01	1.73E+01
ENSMUSG00000018893	Mb	3.63E-01	-2.76E+00	-1.46	0.0253	-1.73E+01	1.73E+01
ENSMUSG00000085028	Slc2a4rg-ps	4.45E-01	-2.25E+00	-1.17	0.0168	-1.73E+01	1.73E+01
ENSMUSG00000022802	Lmln	2.32E+00	2.32E+00	1.21	0.0180	1.73E+01	1.73E+01
ENSMUSG00000105971	RP23-372C7.8	4.37E-01	-2.29E+00	-1.20	0.0176	-1.73E+01	1.73E+01
ENSMUSG00000104848	SNORA43	3.17E-01	-3.16E+00	-1.66	0.0335	-1.73E+01	1.73E+01
ENSMUSG00000106414	RP23-309G12.1	3.32E-01	-3.01E+00	-1.59	0.0304	-1.73E+01	1.73E+01
ENSMUSG00000074607	Tox2	2.24E+00	2.24E+00	1.16	0.0168	1.72E+01	1.72E+01
ENSMUSG00000103952	Gm37268	4.18E-01	-2.39E+00	-1.26	0.0193	-1.72E+01	1.72E+01
ENSMUSG00000105344	RP23-286C5.1	2.60E-01	-3.84E+00	-1.94	0.0497	-1.72E+01	1.72E+01
ENSMUSG00000071226	Cecr2	2.10E+00	2.10E+00	1.07	0.0149	1.72E+01	1.72E+01
ENSMUSG00000050248	Evc2	4.97E-01	-2.01E+00	-1.01	0.0137	-1.72E+01	1.72E+01
ENSMUSG00000030098	Grip2	4.90E-01	-2.04E+00	-1.03	0.0140	-1.72E+01	1.72E+01
ENSMUSG00000089672	Gp49a	3.30E+00	3.30E+00	1.72	0.0368	1.72E+01	1.72E+01
ENSMUSG00000084085	Gm16140	3.49E-01	-2.87E+00	-1.52	0.0278	-1.72E+01	1.72E+01
ENSMUSG00000057729	Prtn3	3.27E+00	3.27E+00	1.71	0.0364	1.72E+01	1.72E+01
ENSMUSG00000002265	Peg3	2.33E+00	2.33E+00	1.22	0.0185	1.72E+01	1.72E+01
ENSMUSG00000082857	Gm12293	2.96E-01	-3.38E+00	-1.76	0.0388	-1.72E+01	1.72E+01
ENSMUSG00000095753	Igkv4-53	3.44E+00	3.44E+00	1.78	0.0402	1.71E+01	1.71E+01
ENSMUSG00000081016	Olfr1397-ps1	3.30E-01	-3.03E+00	-1.60	0.0314	-1.71E+01	1.71E+01
ENSMUSG00000086620	Rspo4os	2.68E+00	2.68E+00	1.42	0.0246	1.71E+01	1.71E+01
ENSMUSG00000070802	Pnmal2	3.45E-01	-2.90E+00	-1.54	0.0287	-1.71E+01	1.71E+01
ENSMUSG00000050014	Apol10b	2.85E-01	-3.51E+00	-1.81	0.0423	-1.71E+01	1.71E+01
ENSMUSG00000029575	Mmab	4.84E-01	-2.07E+00	-1.05	0.0147	-1.70E+01	1.70E+01
ENSMUSG00000047996	Prrg1	4.67E-01	-2.14E+00	-1.10	0.0159	-1.70E+01	1.70E+01
ENSMUSG00000071691	Gm960	2.72E+00	2.72E+00	1.44	0.0256	1.70E+01	1.70E+01
ENSMUSG00000096546	Smlr1	2.35E+00	2.35E+00	1.23	0.0190	1.70E+01	1.70E+01
ENSMUSG00000019539	Rcn3	4.32E-01	-2.32E+00	-1.21	0.0185	-1.70E+01	1.70E+01
ENSMUSG00000075591	Gm10874	3.44E+00	3.44E+00	1.78	0.0408	1.70E+01	1.70E+01
ENSMUSG00000088853	Gm24730	3.53E-01	-2.83E+00	-1.50	0.0277	-1.70E+01	1.70E+01
ENSMUSG00000040693	Slco4c1	4.50E-01	-2.22E+00	-1.15	0.0171	-1.70E+01	1.70E+01
ENSMUSG00000060445	Sycp2	2.13E+00	2.13E+00	1.09	0.0157	1.70E+01	1.70E+01
ENSMUSG00000079071	Gm14085	3.96E-01	-2.53E+00	-1.34	0.0221	-1.70E+01	1.70E+01

ENSMUSG00000104402	Gm38012	4.97E-01	-2.01E+00	-1.01	0.0140	-1.70E+01	1.70E+01
ENSMUSG00000041577	Prelp	3.66E-01	-2.73E+00	-1.45	0.0259	-1.70E+01	1.70E+01
ENSMUSG00000006386	Tek	3.77E-01	-2.65E+00	-1.41	0.0246	-1.69E+01	1.69E+01
ENSMUSG00000038980	Rbbp8nl	3.20E-01	-3.12E+00	-1.64	0.0342	-1.69E+01	1.69E+01
ENSMUSG00000028443	Nudt2	4.15E-01	-2.41E+00	-1.27	0.0203	-1.69E+01	1.69E+01
ENSMUSG00000075036	Gm10805	3.16E+00	3.16E+00	1.66	0.0351	1.69E+01	1.69E+01
ENSMUSG00000071176	Arhgef10	3.61E-01	-2.77E+00	-1.47	0.0269	-1.69E+01	1.69E+01
ENSMUSG00000060188	Cxcl17	2.44E+00	2.44E+00	1.29	0.0209	1.69E+01	1.69E+01
ENSMUSG00000064061	Dzip3	4.42E-01	-2.26E+00	-1.18	0.0180	-1.69E+01	1.69E+01
ENSMUSG00000089924	Gm15689	2.85E-01	-3.51E+00	-1.81	0.0433	-1.69E+01	1.69E+01
ENSMUSG00000055612	Cdca7	4.59E-01	-2.18E+00	-1.12	0.0168	-1.68E+01	1.68E+01
ENSMUSG00000101406	Gm29200	3.38E-01	-2.96E+00	-1.57	0.0310	-1.68E+01	1.68E+01
ENSMUSG00000106073	RP24-370B22.3	4.47E-01	-2.24E+00	-1.16	0.0177	-1.68E+01	1.68E+01
ENSMUSG00000084948	1700061H18Rik	4.51E-01	-2.22E+00	-1.15	0.0174	-1.68E+01	1.68E+01
ENSMUSG00000086704	Gm14582	4.41E-01	-2.27E+00	-1.18	0.0182	-1.68E+01	1.68E+01
ENSMUSG00000029122	Evc	3.61E-01	-2.77E+00	-1.47	0.0272	-1.68E+01	1.68E+01
ENSMUSG00000096986	4930509E16Rik	2.16E+00	2.16E+00	1.11	0.0166	1.67E+01	1.67E+01
ENSMUSG00000090576	Gm17055	3.13E+00	3.13E+00	1.65	0.0350	1.67E+01	1.67E+01
ENSMUSG00000063018	2010204K13Rik	4.02E-01	-2.49E+00	-1.31	0.0221	-1.67E+01	1.67E+01
ENSMUSG00000086410	Gm16158	2.51E+00	2.51E+00	1.33	0.0226	1.67E+01	1.67E+01
ENSMUSG00000030748	Il4ra	2.10E+00	2.10E+00	1.07	0.0158	1.67E+01	1.67E+01
ENSMUSG00000024773	Atg2a	3.38E+00	3.38E+00	1.76	0.0411	1.67E+01	1.67E+01
ENSMUSG00000085539	Gm15561	3.14E-01	-3.19E+00	-1.67	0.0367	-1.66E+01	1.66E+01
ENSMUSG00000025020	Slit1	3.28E+00	3.28E+00	1.71	0.0389	1.66E+01	1.66E+01
ENSMUSG00000045100	Slc25a26	4.75E-01	-2.10E+00	-1.07	0.0161	-1.66E+01	1.66E+01
ENSMUSG00000106633	RP23-265M4.3	4.63E-01	-2.16E+00	-1.11	0.0170	-1.66E+01	1.66E+01
ENSMUSG00000104851	RP24-496C22.5	3.52E-01	-2.84E+00	-1.51	0.0295	-1.66E+01	1.66E+01
ENSMUSG00000067998	Bpifb9a	3.17E-01	-3.15E+00	-1.66	0.0363	-1.65E+01	1.65E+01
ENSMUSG00000104376	Gm37516	3.33E-01	-3.01E+00	-1.59	0.0330	-1.65E+01	1.65E+01
ENSMUSG00000027820	Mme	4.61E-01	-2.17E+00	-1.12	0.0172	-1.65E+01	1.65E+01
ENSMUSG00000025511	Tspan4	2.64E+00	2.64E+00	1.40	0.0255	1.65E+01	1.65E+01
ENSMUSG00000053603	4930442H23Rik	2.52E+00	2.52E+00	1.33	0.0233	1.65E+01	1.65E+01
ENSMUSG00000104118	Gm37298	4.02E-01	-2.49E+00	-1.32	0.0228	-1.65E+01	1.65E+01
ENSMUSG00000060206	Zfp462	4.63E-01	-2.16E+00	-1.11	0.0171	-1.65E+01	1.65E+01
ENSMUSG00000030125	Lrrc23	3.63E-01	-2.75E+00	-1.46	0.0280	-1.64E+01	1.64E+01
ENSMUSG00000000489	Pdgfb	3.17E-01	-3.15E+00	-1.66	0.0368	-1.64E+01	1.64E+01
ENSMUSG00000107363	RP23-319P12.4	4.39E-01	-2.28E+00	-1.19	0.0192	-1.64E+01	1.64E+01
ENSMUSG00000094282	Ccdc42b	2.95E-01	-3.38E+00	-1.76	0.0426	-1.64E+01	1.64E+01
ENSMUSG00000052549	Arl13a	2.67E+00	2.67E+00	1.42	0.0265	1.64E+01	1.64E+01
ENSMUSG00000024063	Lbh	4.25E-01	-2.35E+00	-1.23	0.0206	-1.64E+01	1.64E+01
ENSMUSG00000090779	Gm17110	3.26E-01	-3.06E+00	-1.62	0.0350	-1.64E+01	1.64E+01
ENSMUSG00000036813	Entpd8	2.58E+00	2.58E+00	1.37	0.0250	1.64E+01	1.64E+01
ENSMUSG00000066607	6030419C18Rik	4.21E-01	-2.37E+00	-1.25	0.0211	-1.64E+01	1.64E+01
ENSMUSG00000102300	Gm34106	2.74E+00	2.74E+00	1.45	0.0280	1.63E+01	1.63E+01
ENSMUSG00000103828	2610001A08Rik	4.08E-01	-2.45E+00	-1.29	0.0225	-1.63E+01	1.63E+01
ENSMUSG00000002617	Zfp40	3.88E-01	-2.58E+00	-1.37	0.0249	-1.63E+01	1.63E+01
ENSMUSG00000040883	Tmem205	4.35E-01	-2.30E+00	-1.20	0.0198	-1.63E+01	1.63E+01
ENSMUSG00000105694	RP23-387F14.1	4.34E-01	-2.31E+00	-1.20	0.0200	-1.63E+01	1.63E+01
ENSMUSG00000085496	4930517E11Rik	2.94E-01	-3.40E+00	-1.76	0.0434	-1.63E+01	1.63E+01
ENSMUSG00000072473	1700024G13Rik	3.16E-01	-3.16E+00	-1.66	0.0379	-1.63E+01	1.63E+01
ENSMUSG00000025262	Fam120c	4.60E-01	-2.17E+00	-1.12	0.0179	-1.62E+01	1.62E+01
ENSMUSG00000005674	Tomm40l	3.64E-01	-2.74E+00	-1.46	0.0286	-1.62E+01	1.62E+01
ENSMUSG00000067203	H2-K2	4.26E-01	-2.35E+00	-1.23	0.0211	-1.62E+01	1.62E+01
ENSMUSG00000000530	Acvrl1	3.62E-01	-2.76E+00	-1.47	0.0292	-1.62E+01	1.62E+01
ENSMUSG00000097504	4930516B21Rik	4.03E-01	-2.48E+00	-1.31	0.0238	-1.61E+01	1.61E+01
ENSMUSG00000091906	1700099I09Rik	3.90E-01	-2.56E+00	-1.36	0.0255	-1.61E+01	1.61E+01
ENSMUSG00000097509	B230322F03Rik	2.20E+00	2.20E+00	1.13	0.0188	1.60E+01	1.60E+01
ENSMUSG00000047686	Zcchc5	3.07E-01	-3.26E+00	-1.70	0.0414	-1.60E+01	1.60E+01
ENSMUSG00000094796	BC147527	2.97E-01	-3.37E+00	-1.75	0.0444	-1.60E+01	1.60E+01
ENSMUSG00000101007	Gm29541	3.49E+00	3.49E+00	1.80	0.0480	1.59E+01	1.59E+01
ENSMUSG00000047592	Nxpe5	2.53E+00	2.53E+00	1.34	0.0252	1.59E+01	1.59E+01
ENSMUSG00000089639	Gm2862	3.44E-01	-2.90E+00	-1.54	0.0332	-1.59E+01	1.59E+01
ENSMUSG00000040181	Fmo1	3.00E-01	-3.34E+00	-1.74	0.0439	-1.59E+01	1.59E+01
ENSMUSG00000050675	Gp1ba	2.55E+00	2.55E+00	1.35	0.0257	1.59E+01	1.59E+01
ENSMUSG00000031897	Psmb10	4.67E-01	-2.14E+00	-1.10	0.0181	-1.59E+01	1.59E+01
ENSMUSG00000105954	RP24-299A7.2	2.14E+00	2.14E+00	1.10	0.0181	1.59E+01	1.59E+01
ENSMUSG00000037801	Iqch	3.30E-01	-3.03E+00	-1.60	0.0365	-1.59E+01	1.59E+01

ENSMUSG00000044633	B530045E10Rik	3.38E-01	-2.96E+00	-1.56	0.0347	-1.59E+01	1.59E+01
ENSMUSG00000050650	Mrgpra1	2.22E+00	2.22E+00	1.15	0.0197	1.59E+01	1.59E+01
ENSMUSG00000001156	Mxd1	2.21E+00	2.21E+00	1.14	0.0194	1.59E+01	1.59E+01
ENSMUSG00000029821	Dfna5	2.40E+00	2.40E+00	1.26	0.0229	1.58E+01	1.58E+01
ENSMUSG00000044390	Tigd3	3.42E+00	3.42E+00	1.77	0.0467	1.58E+01	1.58E+01
ENSMUSG00000039069	Mtg2	4.06E-01	-2.46E+00	-1.30	0.0243	-1.58E+01	1.58E+01
ENSMUSG00000056073	Grik2	2.93E-01	-3.41E+00	-1.77	0.0465	-1.58E+01	1.58E+01
ENSMUSG00000090019	Gimap1	4.96E-01	-2.02E+00	-1.01	0.0163	-1.58E+01	1.58E+01
ENSMUSG00000058454	Dhcr7	3.96E-01	-2.52E+00	-1.34	0.0255	-1.58E+01	1.58E+01
ENSMUSG00000107215	RP23-253G12.9	3.83E-01	-2.61E+00	-1.38	0.0273	-1.58E+01	1.58E+01
ENSMUSG00000106603	RP24-496C22.6	3.66E-01	-2.73E+00	-1.45	0.0301	-1.58E+01	1.58E+01
ENSMUSG00000099934	Gm17983	3.25E+00	3.25E+00	1.70	0.0427	1.57E+01	1.57E+01
ENSMUSG00000013236	Ptprs	4.82E-01	-2.07E+00	-1.05	0.0173	-1.57E+01	1.57E+01
ENSMUSG00000005686	Ampd3	2.96E+00	2.96E+00	1.57	0.0355	1.57E+01	1.57E+01
ENSMUSG00000024736	Tmem132a	3.72E-01	-2.69E+00	-1.43	0.0292	-1.57E+01	1.57E+01
ENSMUSG00000020774	Aspa	4.98E-01	-2.01E+00	-1.01	0.0163	-1.57E+01	1.57E+01
ENSMUSG00000086598	Btbd18	3.38E+00	3.38E+00	1.76	0.0463	1.57E+01	1.57E+01
ENSMUSG00000051648	Kctd19	3.36E+00	3.36E+00	1.75	0.0460	1.57E+01	1.57E+01
ENSMUSG00000102316	Gm37629	3.46E+00	3.46E+00	1.79	0.0487	1.57E+01	1.57E+01
ENSMUSG00000028642	4930538K18Rik	3.23E+00	3.23E+00	1.69	0.0426	1.57E+01	1.57E+01
ENSMUSG00000059751	Rps3a3	3.73E-01	-2.68E+00	-1.42	0.0293	-1.57E+01	1.57E+01
ENSMUSG00000024299	Adamts10	4.75E-01	-2.10E+00	-1.07	0.0181	-1.57E+01	1.57E+01
ENSMUSG00000048988	Elfn1	4.52E-01	-2.21E+00	-1.15	0.0200	-1.56E+01	1.56E+01
ENSMUSG00000006711	D130043K22Rik	3.80E-01	-2.63E+00	-1.40	0.0283	-1.56E+01	1.56E+01
ENSMUSG00000091818	Rpl19-ps4	3.25E+00	3.25E+00	1.70	0.0433	1.56E+01	1.56E+01
ENSMUSG00000085170	Lrrc75aos1	3.15E-01	-3.18E+00	-1.67	0.0413	-1.56E+01	1.56E+01
ENSMUSG00000042918	Mamstr	2.20E+00	2.20E+00	1.14	0.0199	1.56E+01	1.56E+01
ENSMUSG00000025731	0610011F06Rik	4.87E-01	-2.05E+00	-1.04	0.0173	-1.56E+01	1.56E+01
ENSMUSG00000093490	Gm19932	2.95E-01	-3.39E+00	-1.76	0.0472	-1.56E+01	1.56E+01
ENSMUSG00000027015	Cybrd1	4.30E-01	-2.32E+00	-1.22	0.0222	-1.56E+01	1.56E+01
ENSMUSG00000085173	Gm2869	2.47E+00	2.47E+00	1.31	0.0252	1.56E+01	1.56E+01
ENSMUSG00000039065	Fam173b	4.33E-01	-2.31E+00	-1.21	0.0220	-1.56E+01	1.56E+01
ENSMUSG00000085247	4930545L23Rik	3.61E-01	-2.77E+00	-1.47	0.0317	-1.56E+01	1.56E+01
ENSMUSG00000077903	Mir294	2.33E+00	2.33E+00	1.22	0.0225	1.55E+01	1.55E+01
ENSMUSG00000053965	Pde5a	3.76E-01	-2.66E+00	-1.41	0.0294	-1.55E+01	1.55E+01
ENSMUSG00000074417	Gm14548	2.90E+00	2.90E+00	1.54	0.0349	1.55E+01	1.55E+01
ENSMUSG00000077493	Snord91a	3.17E-01	-3.16E+00	-1.66	0.0414	-1.55E+01	1.55E+01
ENSMUSG00000003872	Lin7b	2.17E+00	2.17E+00	1.12	0.0196	1.55E+01	1.55E+01
ENSMUSG00000087361	0610043K17Rik	3.37E+00	3.37E+00	1.75	0.0474	1.55E+01	1.55E+01
ENSMUSG00000022288	4930447A16Rik	3.07E-01	-3.26E+00	-1.70	0.0442	-1.55E+01	1.55E+01
ENSMUSG00000084250	Gm13550	3.27E-01	-3.06E+00	-1.61	0.0391	-1.55E+01	1.55E+01
ENSMUSG00000042826	Fgf11	4.67E-01	-2.14E+00	-1.10	0.0192	-1.55E+01	1.55E+01
ENSMUSG00000022639	5330426P16Rik	3.96E-01	-2.53E+00	-1.34	0.0267	-1.55E+01	1.55E+01
ENSMUSG00000010362	Rdm1	4.04E-01	-2.48E+00	-1.31	0.0257	-1.54E+01	1.54E+01
ENSMUSG00000075302	Erich2	3.89E-01	-2.57E+00	-1.36	0.0278	-1.54E+01	1.54E+01
ENSMUSG00000031443	F7	3.30E-01	-3.03E+00	-1.60	0.0387	-1.54E+01	1.54E+01
ENSMUSG00000084960	B430010I23Rik	4.15E-01	-2.41E+00	-1.27	0.0245	-1.54E+01	1.54E+01
ENSMUSG00000032596	Uba7	3.64E-01	-2.75E+00	-1.46	0.0320	-1.54E+01	1.54E+01
ENSMUSG00000103768	Gm37856	2.06E+00	2.06E+00	1.04	0.0179	1.54E+01	1.54E+01
ENSMUSG00000106590	RP23-181P9.3	2.31E+00	2.31E+00	1.21	0.0227	1.53E+01	1.53E+01
ENSMUSG00000104798	RP23-59B8.1	2.24E+00	2.24E+00	1.16	0.0213	1.53E+01	1.53E+01
ENSMUSG00000020781	Tsen54	2.29E+00	2.29E+00	1.19	0.0223	1.53E+01	1.53E+01
ENSMUSG00000035200	Chrnb4	2.43E+00	2.43E+00	1.28	0.0252	1.53E+01	1.53E+01
ENSMUSG00000038751	Ptk6	2.53E+00	2.53E+00	1.34	0.0275	1.53E+01	1.53E+01
ENSMUSG00000073599	Ecscr	3.59E-01	-2.78E+00	-1.48	0.0332	-1.53E+01	1.53E+01
ENSMUSG00000094655	Gm25360	3.32E+00	3.32E+00	1.73	0.0474	1.53E+01	1.53E+01
ENSMUSG00000051671	Coa6	4.74E-01	-2.11E+00	-1.08	0.0192	-1.52E+01	1.52E+01
ENSMUSG00000083372	Gm11235	4.77E-01	-2.09E+00	-1.07	0.0189	-1.52E+01	1.52E+01
ENSMUSG00000093910	Zfp853	3.99E-01	-2.51E+00	-1.33	0.0272	-1.52E+01	1.52E+01
ENSMUSG00000042433	Pih1h3b	3.22E-01	-3.10E+00	-1.63	0.0417	-1.52E+01	1.52E+01
ENSMUSG00000036214	Znrd1as	4.26E-01	-2.35E+00	-1.23	0.0239	-1.52E+01	1.52E+01
ENSMUSG00000004473	Clec11a	4.62E-01	-2.17E+00	-1.11	0.0203	-1.52E+01	1.52E+01
ENSMUSG00000035126	Wdr78	3.48E-01	-2.87E+00	-1.52	0.0359	-1.52E+01	1.52E+01
ENSMUSG00000041845	Rhod	4.54E-01	-2.20E+00	-1.14	0.0211	-1.52E+01	1.52E+01
ENSMUSG00000033187	BC016579	3.28E+00	3.28E+00	1.72	0.0470	1.52E+01	1.52E+01
ENSMUSG00000045629	Sh3tc2	4.48E-01	-2.23E+00	-1.16	0.0217	-1.51E+01	1.51E+01
ENSMUSG00000017453	Pipox	2.13E+00	2.13E+00	1.09	0.0199	1.51E+01	1.51E+01

ENSMUSG00000050370	Ch25h	3.12E+00	3.12E+00	1.64	0.0425	1.51E+01	1.51E+01
ENSMUSG00000034110	Kctd7	4.87E-01	-2.05E+00	-1.04	0.0186	-1.51E+01	1.51E+01
ENSMUSG00000078861	Zfp931	4.30E-01	-2.32E+00	-1.22	0.0238	-1.51E+01	1.51E+01
ENSMUSG00000024905	Mtl5	2.95E+00	2.95E+00	1.56	0.0384	1.50E+01	1.50E+01
ENSMUSG00000042515	Mum1l1	3.50E-01	-2.86E+00	-1.51	0.0361	-1.50E+01	1.50E+01
ENSMUSG00000006642	Tcf23	2.31E+00	2.31E+00	1.21	0.0236	1.50E+01	1.50E+01
ENSMUSG00000007908	Hmgcll1	3.92E-01	-2.55E+00	-1.35	0.0290	-1.50E+01	1.50E+01
ENSMUSG00000093656	Gm20628	2.66E+00	2.66E+00	1.41	0.0315	1.50E+01	1.50E+01
ENSMUSG00000085069	Gm13111	3.10E-01	-3.23E+00	-1.69	0.0465	-1.50E+01	1.50E+01
ENSMUSG00000095794	Igkv6-17	2.33E+00	2.33E+00	1.22	0.0243	1.49E+01	1.49E+01
ENSMUSG00000033610	Pank1	4.05E-01	-2.47E+00	-1.30	0.0273	-1.49E+01	1.49E+01
ENSMUSG00000071037	Camkmt	3.61E-01	-2.77E+00	-1.47	0.0344	-1.49E+01	1.49E+01
ENSMUSG00000094595	Fsbp	2.44E+00	2.44E+00	1.29	0.0267	1.49E+01	1.49E+01
ENSMUSG00000065642	Snora69	3.32E-01	-3.01E+00	-1.59	0.0408	-1.49E+01	1.49E+01
ENSMUSG00000104709	RP24-231P10.4	3.08E-01	-3.25E+00	-1.70	0.0476	-1.49E+01	1.49E+01
ENSMUSG00000027524	Edn3	3.80E-01	-2.63E+00	-1.40	0.0314	-1.49E+01	1.49E+01
ENSMUSG00000027490	E2f1	4.24E-01	-2.36E+00	-1.24	0.0252	-1.49E+01	1.49E+01
ENSMUSG00000084839	Gm14097	4.39E-01	-2.28E+00	-1.19	0.0235	-1.48E+01	1.48E+01
ENSMUSG00000099553	Gm29538	3.36E-01	-2.98E+00	-1.57	0.0402	-1.48E+01	1.48E+01
ENSMUSG00000043153	Ispd	3.90E-01	-2.57E+00	-1.36	0.0299	-1.48E+01	1.48E+01
ENSMUSG00000102200	Gm36957	2.10E+00	2.10E+00	1.07	0.0201	1.48E+01	1.48E+01
ENSMUSG00000042439	Zfp532	4.82E-01	-2.08E+00	-1.05	0.0196	-1.48E+01	1.48E+01
ENSMUSG00000083725	Gm11865	3.10E-01	-3.23E+00	-1.69	0.0475	-1.48E+01	1.48E+01
ENSMUSG00000051490	Foxd4	3.07E+00	3.07E+00	1.62	0.0431	1.48E+01	1.48E+01
ENSMUSG00000035868	3110052M02Rik	4.68E-01	-2.14E+00	-1.10	0.0211	-1.47E+01	1.47E+01
ENSMUSG00000087017	4930417H01Rik	3.16E+00	3.16E+00	1.66	0.0462	1.47E+01	1.47E+01
ENSMUSG00000093695	Gm20717	4.16E-01	-2.40E+00	-1.27	0.0268	-1.47E+01	1.47E+01
ENSMUSG00000099847	Gm29458	2.78E+00	2.78E+00	1.48	0.0361	1.47E+01	1.47E+01
ENSMUSG00000045968	Teddm2	2.27E+00	2.27E+00	1.18	0.0240	1.46E+01	1.46E+01
ENSMUSG00000024921	Smarca2	4.13E-01	-2.42E+00	-1.28	0.0275	-1.46E+01	1.46E+01
ENSMUSG00000062794	Zfp599	2.71E+00	2.71E+00	1.44	0.0346	1.46E+01	1.46E+01
ENSMUSG00000053963	6330403A02Rik	2.05E+00	2.05E+00	1.04	0.0198	1.46E+01	1.46E+01
ENSMUSG00000032300	1700017B05Rik	3.10E+00	3.10E+00	1.63	0.0453	1.46E+01	1.46E+01
ENSMUSG00000090255	4921534H16Rik	3.32E-01	-3.01E+00	-1.59	0.0427	-1.46E+01	1.46E+01
ENSMUSG00000023345	Poc1a	2.76E+00	2.76E+00	1.46	0.0358	1.46E+01	1.46E+01
ENSMUSG00000053886	Sh2d4a	4.15E-01	-2.41E+00	-1.27	0.0275	-1.45E+01	1.45E+01
ENSMUSG00000037169	Mycn	3.83E-01	-2.61E+00	-1.38	0.0323	-1.45E+01	1.45E+01
ENSMUSG00000103940	Gm38227	3.14E+00	3.14E+00	1.65	0.0469	1.45E+01	1.45E+01
ENSMUSG00000032271	Nnmt	2.28E+00	2.28E+00	1.19	0.0249	1.45E+01	1.45E+01
ENSMUSG00000091183	Gm5141	4.17E-01	-2.40E+00	-1.26	0.0275	-1.45E+01	1.45E+01
ENSMUSG00000061601	Pclo	3.56E-01	-2.81E+00	-1.49	0.0380	-1.44E+01	1.44E+01
ENSMUSG00000105700	RP24-158C2.3	3.96E-01	-2.52E+00	-1.34	0.0307	-1.44E+01	1.44E+01
ENSMUSG00000058159	T2	3.28E-01	-3.05E+00	-1.61	0.0449	-1.44E+01	1.44E+01
ENSMUSG00000020264	Slc36a2	3.77E-01	-2.65E+00	-1.41	0.0339	-1.44E+01	1.44E+01
ENSMUSG00000015627	Gata5	4.25E-01	-2.35E+00	-1.24	0.0268	-1.44E+01	1.44E+01
ENSMUSG00000021684	Pde8b	4.21E-01	-2.38E+00	-1.25	0.0273	-1.44E+01	1.44E+01
ENSMUSG00000019880	Rspo3	3.83E-01	-2.61E+00	-1.38	0.0330	-1.44E+01	1.44E+01
ENSMUSG00000026494	Kif26b	4.70E-01	-2.13E+00	-1.09	0.0220	-1.44E+01	1.44E+01
ENSMUSG00000025810	Nrp1	4.01E-01	-2.49E+00	-1.32	0.0302	-1.43E+01	1.43E+01
ENSMUSG00000024593	Megf10	3.33E-01	-3.01E+00	-1.59	0.0440	-1.43E+01	1.43E+01
ENSMUSG00000093651	Gm5873	4.61E-01	-2.17E+00	-1.12	0.0230	-1.43E+01	1.43E+01
ENSMUSG00000061371	Zfp873	4.08E-01	-2.45E+00	-1.29	0.0293	-1.43E+01	1.43E+01
ENSMUSG00000040086	Tnni3k	4.42E-01	-2.26E+00	-1.18	0.0250	-1.43E+01	1.43E+01
ENSMUSG00000090659	Zfp493	3.20E-01	-3.12E+00	-1.64	0.0479	-1.43E+01	1.43E+01
ENSMUSG00000031725	Ces1f	3.81E-01	-2.62E+00	-1.39	0.0339	-1.43E+01	1.43E+01
ENSMUSG00000038077	Kcna6	4.26E-01	-2.34E+00	-1.23	0.0272	-1.42E+01	1.42E+01
ENSMUSG00000106010	RP24-296L6.2	3.05E+00	3.05E+00	1.61	0.0461	1.42E+01	1.42E+01
ENSMUSG00000037624	Kcnk2	3.72E-01	-2.69E+00	-1.43	0.0358	-1.42E+01	1.42E+01
ENSMUSG00000007659	Bcl2l1	2.20E+00	2.20E+00	1.14	0.0240	1.42E+01	1.42E+01
ENSMUSG00000106371	RP24-535M14.1	4.60E-01	-2.17E+00	-1.12	0.0235	-1.42E+01	1.42E+01
ENSMUSG00000030237	Slco1a4	2.91E+00	2.91E+00	1.54	0.0421	1.42E+01	1.42E+01
ENSMUSG00000069763	Tmem100	4.54E-01	-2.20E+00	-1.14	0.0240	-1.42E+01	1.42E+01
ENSMUSG00000105366	RP23-192D21.1	3.59E-01	-2.79E+00	-1.48	0.0386	-1.42E+01	1.42E+01
ENSMUSG00000033182	Kbtbd12	4.22E-01	-2.37E+00	-1.25	0.0280	-1.42E+01	1.42E+01
ENSMUSG00000045555	Mettl24	4.24E-01	-2.36E+00	-1.24	0.0277	-1.42E+01	1.42E+01
ENSMUSG00000025931	Paqr8	3.81E-01	-2.63E+00	-1.39	0.0346	-1.41E+01	1.41E+01
ENSMUSG00000077438	Gm22595	3.30E-01	-3.03E+00	-1.60	0.0462	-1.41E+01	1.41E+01

ENSMUSG00000097954	Gm4217	2.54E+00	2.54E+00	1.34	0.0324	1.41E+01	1.41E+01
ENSMUSG00000001168	Oas1h	4.32E-01	-2.32E+00	-1.21	0.0270	-1.41E+01	1.41E+01
ENSMUSG00000076499	Trbv31	3.23E-01	-3.10E+00	-1.63	0.0486	-1.41E+01	1.41E+01
ENSMUSG00000039116	Adgrg6	3.82E-01	-2.62E+00	-1.39	0.0347	-1.41E+01	1.41E+01
ENSMUSG00000031458	Coprs	4.65E-01	-2.15E+00	-1.10	0.0235	-1.40E+01	1.40E+01
ENSMUSG00000104206	Gm32250	2.83E+00	2.83E+00	1.50	0.0407	1.40E+01	1.40E+01
ENSMUSG00000097232	Gm26854	3.30E-01	-3.03E+00	-1.60	0.0468	-1.40E+01	1.40E+01
ENSMUSG00000034872	Gipc3	3.36E-01	-2.98E+00	-1.57	0.0451	-1.40E+01	1.40E+01
ENSMUSG00000018340	Anxa6	4.48E-01	-2.23E+00	-1.16	0.0253	-1.40E+01	1.40E+01
ENSMUSG00000047485	Klhl34	4.29E-01	-2.33E+00	-1.22	0.0278	-1.40E+01	1.40E+01
ENSMUSG00000087104	Tmem132cos	3.74E-01	-2.67E+00	-1.42	0.0365	-1.40E+01	1.40E+01
ENSMUSG00000074466	Gm15417	2.90E+00	2.90E+00	1.54	0.0430	1.40E+01	1.40E+01
ENSMUSG00000097558	Gm26902	2.46E+00	2.46E+00	1.30	0.0309	1.40E+01	1.40E+01
ENSMUSG00000105041	RP23-10F17.3	3.87E-01	-2.58E+00	-1.37	0.0342	-1.40E+01	1.40E+01
ENSMUSG00000031075	Ano1	4.97E-01	-2.01E+00	-1.01	0.0208	-1.39E+01	1.39E+01
ENSMUSG00000078867	Gm14418	4.97E-01	-2.01E+00	-1.01	0.0209	-1.39E+01	1.39E+01
ENSMUSG00000000359	Rem1	4.15E-01	-2.41E+00	-1.27	0.0300	-1.39E+01	1.39E+01
ENSMUSG00000053964	Lgals4	2.39E+00	2.39E+00	1.26	0.0297	1.39E+01	1.39E+01
ENSMUSG00000021886	Gpr65	2.47E+00	2.47E+00	1.30	0.0316	1.39E+01	1.39E+01
ENSMUSG00000058192	Zfp846	4.48E-01	-2.23E+00	-1.16	0.0259	-1.39E+01	1.39E+01
ENSMUSG00000070282	3000002C10Rik	3.28E-01	-3.05E+00	-1.61	0.0483	-1.39E+01	1.39E+01
ENSMUSG00000030401	Rtn2	4.07E-01	-2.46E+00	-1.30	0.0316	-1.38E+01	1.38E+01
ENSMUSG00000100747	1700084E18Rik	2.56E+00	2.56E+00	1.35	0.0343	1.38E+01	1.38E+01
ENSMUSG00000106446	RP23-50E10.1	3.38E-01	-2.96E+00	-1.56	0.0460	-1.38E+01	1.38E+01
ENSMUSG00000045776	Lrtm1	3.54E-01	-2.82E+00	-1.50	0.0420	-1.38E+01	1.38E+01
ENSMUSG00000097742	Gm26535	2.27E+00	2.27E+00	1.18	0.0273	1.38E+01	1.38E+01
ENSMUSG00000097484	Gm26807	2.85E+00	2.85E+00	1.51	0.0430	1.37E+01	1.37E+01
ENSMUSG00000086181	C230034O21Rik	3.48E-01	-2.87E+00	-1.52	0.0438	-1.37E+01	1.37E+01
ENSMUSG00000073403	Gm10499	3.52E-01	-2.84E+00	-1.51	0.0430	-1.37E+01	1.37E+01
ENSMUSG00000074158	9830147E19Rik	4.58E-01	-2.19E+00	-1.13	0.0254	-1.37E+01	1.37E+01
ENSMUSG00000094786	Gm14403	3.93E-01	-2.54E+00	-1.35	0.0344	-1.37E+01	1.37E+01
ENSMUSG00000005672	Kit	3.44E-01	-2.91E+00	-1.54	0.0450	-1.37E+01	1.37E+01
ENSMUSG00000089889	0610040B10Rik	2.16E+00	2.16E+00	1.11	0.0250	1.37E+01	1.37E+01
ENSMUSG00000098076	Gm6981	4.23E-01	-2.37E+00	-1.24	0.0299	-1.37E+01	1.37E+01
ENSMUSG00000029097	Trmt44	2.28E+00	2.28E+00	1.19	0.0279	1.37E+01	1.37E+01
ENSMUSG00000002504	Slc9a3r2	3.58E-01	-2.80E+00	-1.48	0.0419	-1.37E+01	1.37E+01
ENSMUSG00000100313	Gm28323	3.89E-01	-2.57E+00	-1.36	0.0354	-1.37E+01	1.37E+01
ENSMUSG00000086687	4930547E08Rik	3.02E+00	3.02E+00	1.59	0.0488	1.36E+01	1.36E+01
ENSMUSG00000020137	Thap2	4.10E-01	-2.44E+00	-1.29	0.0320	-1.36E+01	1.36E+01
ENSMUSG00000106197	RP23-200E11.2	4.22E-01	-2.37E+00	-1.24	0.0302	-1.36E+01	1.36E+01
ENSMUSG00000025083	Afap1l2	3.99E-01	-2.50E+00	-1.32	0.0340	-1.36E+01	1.36E+01
ENSMUSG00000074874	Ctla2b	2.67E+00	2.67E+00	1.41	0.0386	1.36E+01	1.36E+01
ENSMUSG00000026610	Esrrg	3.82E-01	-2.62E+00	-1.39	0.0374	-1.36E+01	1.36E+01
ENSMUSG00000106944	RP23-16J8.6	3.43E-01	-2.91E+00	-1.54	0.0464	-1.35E+01	1.35E+01
ENSMUSG00000093740	Gm18417	2.99E+00	2.99E+00	1.58	0.0487	1.35E+01	1.35E+01
ENSMUSG00000045414	1190002N15Rik	4.39E-01	-2.28E+00	-1.19	0.0284	-1.35E+01	1.35E+01
ENSMUSG00000034127	Tspan8	4.40E-01	-2.27E+00	-1.18	0.0283	-1.35E+01	1.35E+01
ENSMUSG00000105771	RP24-253A20.1	3.95E-01	-2.53E+00	-1.34	0.0353	-1.35E+01	1.35E+01
ENSMUSG00000055370	Gm9968	4.25E-01	-2.36E+00	-1.24	0.0305	-1.35E+01	1.35E+01
ENSMUSG00000038305	Spats2l	3.74E-01	-2.67E+00	-1.42	0.0395	-1.34E+01	1.34E+01
ENSMUSG00000021294	Kif26a	4.20E-01	-2.38E+00	-1.25	0.0315	-1.34E+01	1.34E+01
ENSMUSG00000093772	4931403E22Rik	3.61E-01	-2.77E+00	-1.47	0.0426	-1.34E+01	1.34E+01
ENSMUSG00000028573	Fggy	3.79E-01	-2.64E+00	-1.40	0.0386	-1.34E+01	1.34E+01
ENSMUSG00000073242	Dnmt3aos	4.22E-01	-2.37E+00	-1.24	0.0312	-1.34E+01	1.34E+01
ENSMUSG00000022376	Adcy8	3.85E-01	-2.60E+00	-1.38	0.0374	-1.34E+01	1.34E+01
ENSMUSG00000103885	Gm37006	4.64E-01	-2.16E+00	-1.11	0.0258	-1.34E+01	1.34E+01
ENSMUSG00000105357	RP23-7K18.3	3.44E-01	-2.91E+00	-1.54	0.0472	-1.34E+01	1.34E+01
ENSMUSG00000030048	Gkn3	3.64E-01	-2.75E+00	-1.46	0.0423	-1.34E+01	1.34E+01
ENSMUSG00000061540	Orm2	2.72E+00	2.72E+00	1.44	0.0413	1.34E+01	1.34E+01
ENSMUSG00000046318	Ccbe1	2.34E+00	2.34E+00	1.22	0.0306	1.34E+01	1.34E+01
ENSMUSG00000105877	RP23-358D12.3	3.74E-01	-2.67E+00	-1.42	0.0402	-1.33E+01	1.33E+01
ENSMUSG00000017344	Vtn	4.87E-01	-2.06E+00	-1.04	0.0238	-1.33E+01	1.33E+01
ENSMUSG00000029335	Bmp3	4.93E-01	-2.03E+00	-1.02	0.0232	-1.33E+01	1.33E+01
ENSMUSG00000047787	Flrt1	4.44E-01	-2.25E+00	-1.17	0.0286	-1.33E+01	1.33E+01
ENSMUSG00000040690	Col16a1	3.96E-01	-2.52E+00	-1.34	0.0360	-1.33E+01	1.33E+01
ENSMUSG00000106839	RP23-438D3.4	3.94E-01	-2.54E+00	-1.34	0.0365	-1.33E+01	1.33E+01
ENSMUSG00000022656	Pvrl3	4.63E-01	-2.16E+00	-1.11	0.0265	-1.33E+01	1.33E+01

ENSMUSG00000097795	Gm7678	4.13E-01	-2.42E+00	-1.28	0.0333	-1.33E+01	1.33E+01
ENSMUSG00000081375	Gm14686	2.72E+00	2.72E+00	1.44	0.0420	1.33E+01	1.33E+01
ENSMUSG00000053080	2700081O15Rik	4.57E-01	-2.19E+00	-1.13	0.0273	-1.32E+01	1.32E+01
ENSMUSG00000040855	Reps2	3.78E-01	-2.65E+00	-1.40	0.0402	-1.32E+01	1.32E+01
ENSMUSG00000085280	Gm16151	4.04E-01	-2.47E+00	-1.31	0.0351	-1.32E+01	1.32E+01
ENSMUSG00000096215	Smim22	3.41E-01	-2.94E+00	-1.55	0.0496	-1.32E+01	1.32E+01
ENSMUSG00000086479	Gm16014	2.38E+00	2.38E+00	1.25	0.0325	1.32E+01	1.32E+01
ENSMUSG00000056185	Snx32	4.48E-01	-2.23E+00	-1.16	0.0287	-1.32E+01	1.32E+01
ENSMUSG00000062905	Vmn1r32	2.44E+00	2.44E+00	1.29	0.0346	1.31E+01	1.31E+01
ENSMUSG00000020135	Apc2	4.45E-01	-2.25E+00	-1.17	0.0293	-1.31E+01	1.31E+01
ENSMUSG00000097885	5031434O11Rik	2.05E+00	2.05E+00	1.04	0.0244	1.31E+01	1.31E+01
ENSMUSG00000089662	Gm14057	4.30E-01	-2.33E+00	-1.22	0.0315	-1.31E+01	1.31E+01
ENSMUSG00000098482	Mir6363	3.66E-01	-2.73E+00	-1.45	0.0436	-1.31E+01	1.31E+01
ENSMUSG00000087395	Gm12705	4.18E-01	-2.39E+00	-1.26	0.0335	-1.31E+01	1.31E+01
ENSMUSG00000030263	Lrmp	2.16E+00	2.16E+00	1.11	0.0273	1.31E+01	1.31E+01
ENSMUSG00000032134	Muc16	3.80E-01	-2.63E+00	-1.40	0.0406	-1.31E+01	1.31E+01
ENSMUSG00000035295	Wdr38	3.90E-01	-2.56E+00	-1.36	0.0386	-1.31E+01	1.31E+01
ENSMUSG00000082394	Gm4596	2.43E+00	2.43E+00	1.28	0.0348	1.30E+01	1.30E+01
ENSMUSG00000049526	Tmem202	2.27E+00	2.27E+00	1.18	0.0302	1.30E+01	1.30E+01
ENSMUSG00000040987	Mill2	4.48E-01	-2.23E+00	-1.16	0.0294	-1.30E+01	1.30E+01
ENSMUSG00000036551	Akap14	4.12E-01	-2.42E+00	-1.28	0.0347	-1.30E+01	1.30E+01
ENSMUSG00000104713	Gbp6	4.19E-01	-2.39E+00	-1.26	0.0337	-1.30E+01	1.30E+01
ENSMUSG00000083287	Gm13502	3.47E-01	-2.88E+00	-1.53	0.0490	-1.30E+01	1.30E+01
ENSMUSG00000038390	Gpr162	4.92E-01	-2.03E+00	-1.02	0.0244	-1.30E+01	1.30E+01
ENSMUSG00000073609	D2hgdh	4.86E-01	-2.06E+00	-1.04	0.0251	-1.30E+01	1.30E+01
ENSMUSG00000021013	Ttc8	4.28E-01	-2.34E+00	-1.22	0.0325	-1.30E+01	1.30E+01
ENSMUSG00000038406	Scaf1	2.72E+00	2.72E+00	1.44	0.0440	1.30E+01	1.30E+01
ENSMUSG00000037313	Tacc3	2.13E+00	2.13E+00	1.09	0.0270	1.30E+01	1.30E+01
ENSMUSG00000064925	Snora62	2.23E+00	2.23E+00	1.15	0.0296	1.30E+01	1.30E+01
ENSMUSG00000029718	Pcolce	4.32E-01	-2.32E+00	-1.21	0.0321	-1.29E+01	1.29E+01
ENSMUSG00000081585	Gm12926	4.66E-01	-2.14E+00	-1.10	0.0276	-1.29E+01	1.29E+01
ENSMUSG00000098889	Gm27206	4.81E-01	-2.08E+00	-1.06	0.0261	-1.29E+01	1.29E+01
ENSMUSG00000099875	Gm15453	2.70E+00	2.70E+00	1.43	0.0439	1.29E+01	1.29E+01
ENSMUSG00000042066	Tmcc2	4.62E-01	-2.17E+00	-1.12	0.0285	-1.28E+01	1.28E+01
ENSMUSG00000015224	Cyp2j9	4.47E-01	-2.24E+00	-1.16	0.0305	-1.28E+01	1.28E+01
ENSMUSG00000019945	1700040L02Rik	4.90E-01	-2.04E+00	-1.03	0.0255	-1.28E+01	1.28E+01
ENSMUSG00000024960	Plcb3	4.66E-01	-2.15E+00	-1.10	0.0283	-1.28E+01	1.28E+01
ENSMUSG00000032076	Cadm1	4.51E-01	-2.22E+00	-1.15	0.0302	-1.28E+01	1.28E+01
ENSMUSG00000105784	RP23-113O9.4	2.26E+00	2.26E+00	1.17	0.0314	1.27E+01	1.27E+01
ENSMUSG00000048022	Tmem229a	3.73E-01	-2.68E+00	-1.42	0.0444	-1.27E+01	1.27E+01
ENSMUSG00000102959	Gm37015	2.42E+00	2.42E+00	1.27	0.0362	1.27E+01	1.27E+01
ENSMUSG00000102326	Gm37788	4.11E-01	-2.43E+00	-1.28	0.0365	-1.27E+01	1.27E+01
ENSMUSG00000097254	C430042M11Rik	2.03E+00	2.03E+00	1.02	0.0254	1.27E+01	1.27E+01
ENSMUSG00000097461	Gm26735	4.59E-01	-2.18E+00	-1.12	0.0294	-1.27E+01	1.27E+01
ENSMUSG00000024391	Apom	2.79E+00	2.79E+00	1.48	0.0482	1.27E+01	1.27E+01
ENSMUSG00000101951	Gm28721	2.83E+00	2.83E+00	1.50	0.0497	1.27E+01	1.27E+01
ENSMUSG00000089376	Gm22136	3.78E-01	-2.65E+00	-1.40	0.0435	-1.27E+01	1.27E+01
ENSMUSG00000044229	Nxpe4	4.29E-01	-2.33E+00	-1.22	0.0338	-1.27E+01	1.27E+01
ENSMUSG00000068762	Gstm6	4.35E-01	-2.30E+00	-1.20	0.0330	-1.27E+01	1.27E+01
ENSMUSG00000044921	Rassf9	4.49E-01	-2.23E+00	-1.16	0.0310	-1.27E+01	1.27E+01
ENSMUSG00000103427	Gm37534	3.87E-01	-2.58E+00	-1.37	0.0418	-1.26E+01	1.26E+01
ENSMUSG00000033065	Pfkm	4.86E-01	-2.06E+00	-1.04	0.0268	-1.26E+01	1.26E+01
ENSMUSG00000039887	Alg14	4.12E-01	-2.43E+00	-1.28	0.0373	-1.26E+01	1.26E+01
ENSMUSG00000097707	Gm26742	4.72E-01	-2.12E+00	-1.08	0.0284	-1.26E+01	1.26E+01
ENSMUSG00000033863	Klf9	2.32E+00	2.32E+00	1.21	0.0342	1.26E+01	1.26E+01
ENSMUSG00000019853	Hebp2	4.04E-01	-2.48E+00	-1.31	0.0389	-1.26E+01	1.26E+01
ENSMUSG00000043251	Exoc3l	4.05E-01	-2.47E+00	-1.30	0.0387	-1.26E+01	1.26E+01
ENSMUSG00000001663	Gstt1	4.22E-01	-2.37E+00	-1.24	0.0357	-1.25E+01	1.25E+01
ENSMUSG00000027574	Nkain4	4.26E-01	-2.35E+00	-1.23	0.0352	-1.25E+01	1.25E+01
ENSMUSG00000086930	Frs3os	4.53E-01	-2.21E+00	-1.14	0.0313	-1.25E+01	1.25E+01
ENSMUSG00000052776	Oas1a	4.09E-01	-2.44E+00	-1.29	0.0386	-1.24E+01	1.24E+01
ENSMUSG00000045031	Cetn4	4.29E-01	-2.33E+00	-1.22	0.0351	-1.24E+01	1.24E+01
ENSMUSG00000069743	Zfp820	4.90E-01	-2.04E+00	-1.03	0.0270	-1.24E+01	1.24E+01
ENSMUSG00000031825	Crispld2	2.10E+00	2.10E+00	1.07	0.0287	1.24E+01	1.24E+01
ENSMUSG00000032988	Slc16a8	2.40E+00	2.40E+00	1.26	0.0374	1.24E+01	1.24E+01
ENSMUSG00000039699	Batf2	3.80E-01	-2.63E+00	-1.40	0.0449	-1.24E+01	1.24E+01
ENSMUSG00000002763	Pex6	4.42E-01	-2.26E+00	-1.18	0.0332	-1.24E+01	1.24E+01

ENSMUSG00000038742	Angptl6	4.08E-01	-2.45E+00	-1.29	0.0393	-1.24E+01	1.24E+01
ENSMUSG00000047557	Lxn	4.88E-01	-2.05E+00	-1.03	0.0275	-1.24E+01	1.24E+01
ENSMUSG00000071757	Zhx2	3.87E-01	-2.58E+00	-1.37	0.0437	-1.23E+01	1.23E+01
ENSMUSG00000058056	Palld	4.55E-01	-2.20E+00	-1.14	0.0319	-1.23E+01	1.23E+01
ENSMUSG00000052373	Mpp3	2.08E+00	2.08E+00	1.06	0.0287	1.23E+01	1.23E+01
ENSMUSG00000084202	Gm5398	2.27E+00	2.27E+00	1.18	0.0345	1.22E+01	1.22E+01
ENSMUSG00000028354	Fmn2	4.15E-01	-2.41E+00	-1.27	0.0390	-1.22E+01	1.22E+01
ENSMUSG00000009772	Nuak2	2.32E+00	2.32E+00	1.21	0.0362	1.22E+01	1.22E+01
ENSMUSG00000045930	Clec14a	4.51E-01	-2.22E+00	-1.15	0.0333	-1.22E+01	1.22E+01
ENSMUSG00000021048	Mthfd1	4.26E-01	-2.35E+00	-1.23	0.0373	-1.22E+01	1.22E+01
ENSMUSG00000035932	Olfr750	4.38E-01	-2.28E+00	-1.19	0.0353	-1.22E+01	1.22E+01
ENSMUSG00000076677	Ighv6-3	3.90E-01	-2.56E+00	-1.36	0.0446	-1.21E+01	1.21E+01
ENSMUSG00000103808	Gm37060	4.25E-01	-2.35E+00	-1.23	0.0377	-1.21E+01	1.21E+01
ENSMUSG00000062861	Zfp28	4.44E-01	-2.25E+00	-1.17	0.0346	-1.21E+01	1.21E+01
ENSMUSG00000035711	Dok3	2.04E+00	2.04E+00	1.03	0.0284	1.21E+01	1.21E+01
ENSMUSG00000051851	Cxx1c	3.75E-01	-2.67E+00	-1.42	0.0486	-1.21E+01	1.21E+01
ENSMUSG00000106957	RP24-168A7.7	4.52E-01	-2.21E+00	-1.15	0.0335	-1.21E+01	1.21E+01
ENSMUSG00000084306	Gm11743	4.76E-01	-2.10E+00	-1.07	0.0302	-1.21E+01	1.21E+01
ENSMUSG00000083121	Gm6341	4.15E-01	-2.41E+00	-1.27	0.0397	-1.21E+01	1.21E+01
ENSMUSG00000089785	Slc25a5-ps	2.37E+00	2.37E+00	1.25	0.0387	1.21E+01	1.21E+01
ENSMUSG00000102501	Gm37372	3.89E-01	-2.57E+00	-1.36	0.0455	-1.21E+01	1.21E+01
ENSMUSG00000101555	Gm28731	2.42E+00	2.42E+00	1.27	0.0402	1.21E+01	1.21E+01
ENSMUSG00000039577	Nphp4	4.49E-01	-2.23E+00	-1.16	0.0344	-1.20E+01	1.20E+01
ENSMUSG00000033460	Armcx1	3.90E-01	-2.56E+00	-1.36	0.0454	-1.20E+01	1.20E+01
ENSMUSG00000103656	Gm37205	4.26E-01	-2.35E+00	-1.23	0.0383	-1.20E+01	1.20E+01
ENSMUSG00000088856	Gm24727	2.63E+00	2.63E+00	1.40	0.0482	1.20E+01	1.20E+01
ENSMUSG00000030219	Erp27	2.58E+00	2.58E+00	1.37	0.0463	1.20E+01	1.20E+01
ENSMUSG00000008035	Mid1ip1	4.74E-01	-2.11E+00	-1.08	0.0309	-1.20E+01	1.20E+01
ENSMUSG00000025817	Nudt5	4.45E-01	-2.24E+00	-1.17	0.0351	-1.20E+01	1.20E+01
ENSMUSG00000040136	Abcc8	4.37E-01	-2.29E+00	-1.19	0.0365	-1.20E+01	1.20E+01
ENSMUSG00000039246	Lyplal1	3.84E-01	-2.60E+00	-1.38	0.0474	-1.20E+01	1.20E+01
ENSMUSG00000026938	Fcna	2.55E+00	2.55E+00	1.35	0.0456	1.20E+01	1.20E+01
ENSMUSG00000104034	2900092N22Rik	4.99E-01	-2.00E+00	-1.00	0.0281	-1.19E+01	1.19E+01
ENSMUSG00000033715	Akr1c14	2.39E+00	2.39E+00	1.26	0.0401	1.19E+01	1.19E+01
ENSMUSG00000047604	Frat2	2.19E+00	2.19E+00	1.13	0.0337	1.19E+01	1.19E+01
ENSMUSG00000054426	A930005H10Rik	2.07E+00	2.07E+00	1.05	0.0303	1.19E+01	1.19E+01
ENSMUSG00000054021	Sirt5	3.84E-01	-2.60E+00	-1.38	0.0478	-1.19E+01	1.19E+01
ENSMUSG00000011382	Dhdh	4.28E-01	-2.34E+00	-1.22	0.0386	-1.19E+01	1.19E+01
ENSMUSG00000047735	Samd9l	4.81E-01	-2.08E+00	-1.06	0.0306	-1.19E+01	1.19E+01
ENSMUSG00000032006	Pdgfd	3.78E-01	-2.65E+00	-1.40	0.0497	-1.19E+01	1.19E+01
ENSMUSG00000065899	Gm24523	2.35E+00	2.35E+00	1.23	0.0391	1.19E+01	1.19E+01
ENSMUSG00000033111	3830406C13Rik	4.85E-01	-2.06E+00	-1.04	0.0302	-1.19E+01	1.19E+01
ENSMUSG00000009246	Trpm5	2.20E+00	2.20E+00	1.14	0.0346	1.18E+01	1.18E+01
ENSMUSG00000074283	Zfp109	4.33E-01	-2.31E+00	-1.21	0.0383	-1.18E+01	1.18E+01
ENSMUSG00000105733	RP23-50E10.4	4.44E-01	-2.25E+00	-1.17	0.0366	-1.18E+01	1.18E+01
ENSMUSG00000081733	Gm12852	4.18E-01	-2.39E+00	-1.26	0.0414	-1.18E+01	1.18E+01
ENSMUSG00000107325	RP23-342A15.2	2.28E+00	2.28E+00	1.19	0.0375	1.18E+01	1.18E+01
ENSMUSG00000023176	Cpn2	2.34E+00	2.34E+00	1.23	0.0396	1.18E+01	1.18E+01
ENSMUSG00000042770	Hebp1	4.81E-01	-2.08E+00	-1.06	0.0313	-1.18E+01	1.18E+01
ENSMUSG00000017188	Coa3	4.71E-01	-2.12E+00	-1.09	0.0326	-1.18E+01	1.18E+01
ENSMUSG00000030544	Mesp1	4.30E-01	-2.33E+00	-1.22	0.0392	-1.18E+01	1.18E+01
ENSMUSG00000060470	Adgrg3	2.02E+00	2.02E+00	1.01	0.0295	1.18E+01	1.18E+01
ENSMUSG00000069874	Irgm2	4.49E-01	-2.23E+00	-1.15	0.0359	-1.17E+01	1.17E+01
ENSMUSG00000102205	9430092D12Rik	4.23E-01	-2.37E+00	-1.24	0.0406	-1.17E+01	1.17E+01
ENSMUSG00000025453	Nnt	4.28E-01	-2.34E+00	-1.22	0.0396	-1.17E+01	1.17E+01
ENSMUSG00000014158	Trpv4	2.17E+00	2.17E+00	1.12	0.0343	1.17E+01	1.17E+01
ENSMUSG00000097890	4930547M16Rik	4.09E-01	-2.45E+00	-1.29	0.0440	-1.17E+01	1.17E+01
ENSMUSG00000103403	Gm37931	2.11E+00	2.11E+00	1.08	0.0329	1.17E+01	1.17E+01
ENSMUSG00000078864	Gm14322	4.14E-01	-2.42E+00	-1.27	0.0429	-1.17E+01	1.17E+01
ENSMUSG00000056553	Ptprn2	2.16E+00	2.16E+00	1.11	0.0343	1.16E+01	1.16E+01
ENSMUSG00000102344	9430053O09Rik	4.90E-01	-2.04E+00	-1.03	0.0309	-1.16E+01	1.16E+01
ENSMUSG00000035112	Wnk4	2.24E+00	2.24E+00	1.16	0.0372	1.16E+01	1.16E+01
ENSMUSG00000020604	Arsg	2.12E+00	2.12E+00	1.08	0.0333	1.16E+01	1.16E+01
ENSMUSG00000032066	Bco2	4.25E-01	-2.35E+00	-1.24	0.0412	-1.16E+01	1.16E+01
ENSMUSG00000106461	RP23-112C14.1	4.59E-01	-2.18E+00	-1.12	0.0353	-1.16E+01	1.16E+01
ENSMUSG00000083679	Gm12892	4.95E-01	-2.02E+00	-1.01	0.0307	-1.15E+01	1.15E+01
ENSMUSG00000072115	Ang	4.67E-01	-2.14E+00	-1.10	0.0345	-1.15E+01	1.15E+01

ENSMUSG00000070000	Fcho1	2.23E+00	2.23E+00	1.16	0.0376	1.15E+01	1.15E+01
ENSMUSG00000057751	Megf6	4.91E-01	-2.04E+00	-1.03	0.0313	-1.15E+01	1.15E+01
ENSMUSG00000038248	Sobp	2.15E+00	2.15E+00	1.10	0.0351	1.15E+01	1.15E+01
ENSMUSG00000063450	Syne2	4.86E-01	-2.06E+00	-1.04	0.0322	-1.15E+01	1.15E+01
ENSMUSG00000048087	Gm4737	2.47E+00	2.47E+00	1.31	0.0466	1.15E+01	1.15E+01
ENSMUSG00000020486	Sep4	4.46E-01	-2.24E+00	-1.16	0.0387	-1.14E+01	1.14E+01
ENSMUSG00000029524	Sirt4	4.65E-01	-2.15E+00	-1.11	0.0357	-1.14E+01	1.14E+01
ENSMUSG00000083356	Gm11708	2.03E+00	2.03E+00	1.02	0.0317	1.14E+01	1.14E+01
ENSMUSG00000103553	Gm38218	2.18E+00	2.18E+00	1.12	0.0368	1.14E+01	1.14E+01
ENSMUSG00000048534	Amica1	2.20E+00	2.20E+00	1.14	0.0377	1.14E+01	1.14E+01
ENSMUSG00000074867	Zfp808	4.20E-01	-2.38E+00	-1.25	0.0440	-1.13E+01	1.13E+01
ENSMUSG00000096054	Syne1	4.49E-01	-2.23E+00	-1.16	0.0387	-1.13E+01	1.13E+01
ENSMUSG00000014303	Glis2	4.80E-01	-2.08E+00	-1.06	0.0339	-1.13E+01	1.13E+01
ENSMUSG00000105895	RP23-407I6.2	2.15E+00	2.15E+00	1.10	0.0360	1.13E+01	1.13E+01
ENSMUSG00000054404	Slfn5	4.46E-01	-2.24E+00	-1.16	0.0393	-1.13E+01	1.13E+01
ENSMUSG00000038576	Susd4	4.44E-01	-2.25E+00	-1.17	0.0397	-1.13E+01	1.13E+01
ENSMUSG00000085204	Gm15327	2.23E+00	2.23E+00	1.16	0.0391	1.13E+01	1.13E+01
ENSMUSG00000055691	Gja6	4.26E-01	-2.35E+00	-1.23	0.0434	-1.13E+01	1.13E+01
ENSMUSG00000045193	Cirbp	2.32E+00	2.32E+00	1.21	0.0425	1.13E+01	1.13E+01
ENSMUSG00000086785	Gm6081	4.26E-01	-2.35E+00	-1.23	0.0434	-1.13E+01	1.13E+01
ENSMUSG00000031775	Pllp	4.49E-01	-2.23E+00	-1.15	0.0392	-1.12E+01	1.12E+01
ENSMUSG00000038403	Hfe2	4.59E-01	-2.18E+00	-1.12	0.0376	-1.12E+01	1.12E+01
ENSMUSG00000007480	Mc5r	2.50E+00	2.50E+00	1.32	0.0495	1.12E+01	1.12E+01
ENSMUSG00000045094	Arhgef37	2.47E+00	2.47E+00	1.30	0.0483	1.12E+01	1.12E+01
ENSMUSG00000060509	Xcr1	4.02E-01	-2.48E+00	-1.31	0.0494	-1.12E+01	1.12E+01
ENSMUSG00000087256	Gm15990	2.42E+00	2.42E+00	1.27	0.0468	1.12E+01	1.12E+01
ENSMUSG00000042116	Vwa1	4.52E-01	-2.21E+00	-1.15	0.0393	-1.12E+01	1.12E+01
ENSMUSG00000044258	Ctla2a	2.48E+00	2.48E+00	1.31	0.0493	1.12E+01	1.12E+01
ENSMUSG00000047658	Gal3st3	2.30E+00	2.30E+00	1.20	0.0426	1.11E+01	1.11E+01
ENSMUSG00000087475	4933406I18Rik	2.04E+00	2.04E+00	1.03	0.0338	1.11E+01	1.11E+01
ENSMUSG00000013584	Aldh1a2	4.85E-01	-2.06E+00	-1.04	0.0345	-1.11E+01	1.11E+01
ENSMUSG00000023959	Clic5	4.83E-01	-2.07E+00	-1.05	0.0348	-1.11E+01	1.11E+01
ENSMUSG00000098201	Gm26983	4.28E-01	-2.34E+00	-1.23	0.0445	-1.11E+01	1.11E+01
ENSMUSG00000031886	Ces2e	4.65E-01	-2.15E+00	-1.10	0.0376	-1.11E+01	1.11E+01
ENSMUSG00000086434	Gm15200	2.01E+00	2.01E+00	1.00	0.0329	1.11E+01	1.11E+01
ENSMUSG00000045467	Ttll13	2.35E+00	2.35E+00	1.23	0.0451	1.11E+01	1.11E+01
ENSMUSG00000086279	Gm15634	4.54E-01	-2.20E+00	-1.14	0.0398	-1.11E+01	1.11E+01
ENSMUSG00000002341	Ncan	2.17E+00	2.17E+00	1.12	0.0384	1.11E+01	1.11E+01
ENSMUSG00000035275	Raver2	4.93E-01	-2.03E+00	-1.02	0.0338	-1.10E+01	1.10E+01
ENSMUSG00000031904	Slc7a6	2.23E+00	2.23E+00	1.16	0.0413	1.10E+01	1.10E+01
ENSMUSG00000106251	RP24-503L4.4	2.20E+00	2.20E+00	1.14	0.0403	1.10E+01	1.10E+01
ENSMUSG00000106337	RP23-358D12.2	4.50E-01	-2.22E+00	-1.15	0.0411	-1.10E+01	1.10E+01
ENSMUSG00000033684	Qsox1	2.24E+00	2.24E+00	1.16	0.0419	1.10E+01	1.10E+01
ENSMUSG00000105485	RP23-342M4.3	4.13E-01	-2.42E+00	-1.28	0.0490	-1.10E+01	1.10E+01
ENSMUSG00000079889	Gm11951	2.43E+00	2.43E+00	1.28	0.0492	1.09E+01	1.09E+01
ENSMUSG00000104052	Gm38125	4.83E-01	-2.07E+00	-1.05	0.0359	-1.09E+01	1.09E+01
ENSMUSG00000088875	Gm25862	2.25E+00	2.25E+00	1.17	0.0426	1.09E+01	1.09E+01
ENSMUSG00000080747	Gm14016	2.04E+00	2.04E+00	1.03	0.0352	1.09E+01	1.09E+01
ENSMUSG00000018727	Cpsf4l	4.59E-01	-2.18E+00	-1.12	0.0401	-1.09E+01	1.09E+01
ENSMUSG00000104524	Gm37333	4.50E-01	-2.22E+00	-1.15	0.0417	-1.09E+01	1.09E+01
ENSMUSG00000014426	Map3k4	4.81E-01	-2.08E+00	-1.06	0.0366	-1.09E+01	1.09E+01
ENSMUSG00000048603	Gm9828	2.19E+00	2.19E+00	1.13	0.0407	1.09E+01	1.09E+01
ENSMUSG00000049744	Arhgap15	2.22E+00	2.22E+00	1.15	0.0417	1.09E+01	1.09E+01
ENSMUSG00000106673	RP23-319P12.3	4.72E-01	-2.12E+00	-1.08	0.0381	-1.09E+01	1.09E+01
ENSMUSG00000100707	Gm28523	4.32E-01	-2.31E+00	-1.21	0.0454	-1.09E+01	1.09E+01
ENSMUSG00000030157	Clec2d	4.47E-01	-2.24E+00	-1.16	0.0425	-1.09E+01	1.09E+01
ENSMUSG00000022479	Vdr	2.25E+00	2.25E+00	1.17	0.0434	1.08E+01	1.08E+01
ENSMUSG00000053644	Aldh7a1	4.26E-01	-2.35E+00	-1.23	0.0471	-1.08E+01	1.08E+01
ENSMUSG00000054966	Lmntd1	4.55E-01	-2.20E+00	-1.14	0.0414	-1.08E+01	1.08E+01
ENSMUSG00000090778	Gm3235	4.74E-01	-2.11E+00	-1.08	0.0384	-1.08E+01	1.08E+01
ENSMUSG00000073002	Vamp5	4.63E-01	-2.16E+00	-1.11	0.0406	-1.07E+01	1.07E+01
ENSMUSG00000020744	Slc25a19	4.98E-01	-2.01E+00	-1.01	0.0351	-1.07E+01	1.07E+01
ENSMUSG00000022836	Mylk	4.76E-01	-2.10E+00	-1.07	0.0386	-1.07E+01	1.07E+01
ENSMUSG00000073492	Gm10521	2.20E+00	2.20E+00	1.14	0.0424	1.07E+01	1.07E+01
ENSMUSG00000021120	Pigh	4.68E-01	-2.14E+00	-1.09	0.0401	-1.07E+01	1.07E+01
ENSMUSG00000030782	Tgfb1i1	4.61E-01	-2.17E+00	-1.12	0.0414	-1.06E+01	1.06E+01
ENSMUSG00000099419	1700001D01Rik	4.27E-01	-2.34E+00	-1.23	0.0488	-1.06E+01	1.06E+01

ENSMUSG00000004347	Pde1c	4.61E-01	-2.17E+00	-1.12	0.0420	-1.06E+01	1.06E+01
ENSMUSG00000057897	Camk2b	2.15E+00	2.15E+00	1.10	0.0412	1.06E+01	1.06E+01
ENSMUSG00000040621	Gemin8	4.40E-01	-2.27E+00	-1.18	0.0465	-1.05E+01	1.05E+01
ENSMUSG00000004098	Col5a3	4.34E-01	-2.30E+00	-1.20	0.0480	-1.05E+01	1.05E+01
ENSMUSG00000087403	Kantr	4.47E-01	-2.24E+00	-1.16	0.0453	-1.05E+01	1.05E+01
ENSMUSG00000040717	Il17rd	4.67E-01	-2.14E+00	-1.10	0.0416	-1.05E+01	1.05E+01
ENSMUSG00000041773	Enc1	2.31E+00	2.31E+00	1.21	0.0484	1.05E+01	1.05E+01
ENSMUSG00000008384	Sertad1	2.29E+00	2.29E+00	1.20	0.0478	1.05E+01	1.05E+01
ENSMUSG00000020638	Cmpk2	4.43E-01	-2.26E+00	-1.17	0.0465	-1.05E+01	1.05E+01
ENSMUSG00000030515	Tarsl2	4.65E-01	-2.15E+00	-1.11	0.0423	-1.05E+01	1.05E+01
ENSMUSG00000024085	Man2a1	2.29E+00	2.29E+00	1.20	0.0482	1.04E+01	1.04E+01
ENSMUSG00000090387	Gm17056	2.28E+00	2.28E+00	1.19	0.0477	1.04E+01	1.04E+01
ENSMUSG00000028040	Efna4	2.30E+00	2.30E+00	1.20	0.0488	1.04E+01	1.04E+01
ENSMUSG00000034324	Tmem132c	4.36E-01	-2.29E+00	-1.20	0.0486	-1.04E+01	1.04E+01
ENSMUSG00000074577	Fam65c	2.21E+00	2.21E+00	1.14	0.0454	1.04E+01	1.04E+01
ENSMUSG00000104066	Gm37955	4.62E-01	-2.17E+00	-1.12	0.0437	-1.04E+01	1.04E+01
ENSMUSG00000035759	Bbs10	4.65E-01	-2.15E+00	-1.11	0.0434	-1.03E+01	1.03E+01
ENSMUSG00000060771	Tsga10	4.65E-01	-2.15E+00	-1.10	0.0434	-1.03E+01	1.03E+01
ENSMUSG00000027950	Chrnb2	2.30E+00	2.30E+00	1.20	0.0499	1.03E+01	1.03E+01
ENSMUSG00000002845	Tmem39a	2.06E+00	2.06E+00	1.04	0.0400	1.03E+01	1.03E+01
ENSMUSG00000102142	Gm26930	4.49E-01	-2.23E+00	-1.16	0.0471	-1.03E+01	1.03E+01
ENSMUSG00000081648	Gm13423	2.15E+00	2.15E+00	1.11	0.0442	1.02E+01	1.02E+01
ENSMUSG00000090141	Gm614	2.02E+00	2.02E+00	1.02	0.0391	1.02E+01	1.02E+01
ENSMUSG00000032105	Pdzd3	2.02E+00	2.02E+00	1.01	0.0390	1.02E+01	1.02E+01
ENSMUSG00000090685	Gm9047	2.13E+00	2.13E+00	1.09	0.0436	1.02E+01	1.02E+01
ENSMUSG00000043110	Lrrn4	4.82E-01	-2.08E+00	-1.05	0.0414	-1.02E+01	1.02E+01
ENSMUSG00000024426	Atat1	4.87E-01	-2.05E+00	-1.04	0.0406	-1.02E+01	1.02E+01
ENSMUSG00000081801	Dnmt3l-ps1	2.02E+00	2.02E+00	1.01	0.0392	1.02E+01	1.02E+01
ENSMUSG00000083771	Gm15988	4.54E-01	-2.20E+00	-1.14	0.0476	-1.01E+01	1.01E+01
ENSMUSG00000023939	Mrpl14	4.95E-01	-2.02E+00	-1.01	0.0400	-1.01E+01	1.01E+01
ENSMUSG00000098488	Pla2g4b	2.20E+00	2.20E+00	1.14	0.0479	1.01E+01	1.01E+01
ENSMUSG00000023795	Pisd-ps2	4.80E-01	-2.09E+00	-1.06	0.0432	-1.00E+01	1.00E+01
ENSMUSG00000083013	Gm12978	4.49E-01	-2.23E+00	-1.15	0.0495	-1.00E+01	1.00E+01
ENSMUSG00000005465	Il27ra	4.80E-01	-2.08E+00	-1.06	0.0435	-9.99E+00	9.99E+00
ENSMUSG00000038065	Mturn	4.91E-01	-2.04E+00	-1.03	0.0417	-9.98E+00	9.98E+00
ENSMUSG00000022286	Grhl2	4.64E-01	-2.15E+00	-1.11	0.0470	-9.93E+00	9.93E+00
ENSMUSG00000062284	Gm6030	2.12E+00	2.12E+00	1.09	0.0462	9.88E+00	9.88E+00
ENSMUSG00000022439	Parvg	2.08E+00	2.08E+00	1.06	0.0447	9.84E+00	9.84E+00
ENSMUSG00000039252	Lgi2	4.89E-01	-2.05E+00	-1.03	0.0433	-9.83E+00	9.83E+00
ENSMUSG00000086992	Gm15941	4.60E-01	-2.17E+00	-1.12	0.0492	-9.80E+00	9.80E+00
ENSMUSG00000004500	Zfp324	4.92E-01	-2.03E+00	-1.02	0.0432	-9.79E+00	9.79E+00
ENSMUSG00000105864	RP23-407F12.1	4.70E-01	-2.13E+00	-1.09	0.0474	-9.77E+00	9.77E+00
ENSMUSG00000022763	Aifm3	4.68E-01	-2.14E+00	-1.10	0.0481	-9.74E+00	9.74E+00
ENSMUSG00000025171	Ubtd1	2.03E+00	2.03E+00	1.02	0.0439	9.71E+00	9.71E+00
ENSMUSG00000052974	Cyp2f2	4.86E-01	-2.06E+00	-1.04	0.0453	-9.68E+00	9.68E+00
ENSMUSG00000067424	Zfp563	4.84E-01	-2.06E+00	-1.05	0.0456	-9.67E+00	9.67E+00
ENSMUSG00000090231	Cfb	2.03E+00	2.03E+00	1.02	0.0445	9.64E+00	9.64E+00
ENSMUSG00000104145	D130019J16Rik	4.79E-01	-2.09E+00	-1.06	0.0469	-9.63E+00	9.63E+00
ENSMUSG00000032278	Paqr5	4.99E-01	-2.00E+00	-1.00	0.0442	-9.54E+00	9.54E+00
ENSMUSG00000036962	Cfap221	4.90E-01	-2.04E+00	-1.03	0.0460	-9.52E+00	9.52E+00
ENSMUSG00000015647	Lama5	2.02E+00	2.02E+00	1.01	0.0448	9.52E+00	9.52E+00
ENSMUSG00000037101	Ttc29	4.91E-01	-2.04E+00	-1.03	0.0461	-9.48E+00	9.48E+00
ENSMUSG00000038506	Dcun1d2	4.95E-01	-2.02E+00	-1.01	0.0457	-9.45E+00	9.45E+00
ENSMUSG00000003934	Efnb3	2.06E+00	2.06E+00	1.04	0.0473	9.45E+00	9.45E+00
ENSMUSG00000022496	Tnfrsf17	4.85E-01	-2.06E+00	-1.04	0.0482	-9.40E+00	9.40E+00
ENSMUSG00000024654	Asrgl1	2.09E+00	2.09E+00	1.06	0.0494	9.38E+00	9.38E+00
ENSMUSG00000044952	Kctd21	4.87E-01	-2.06E+00	-1.04	0.0484	-9.34E+00	9.34E+00
ENSMUSG00000051373	Ppapdc3	4.85E-01	-2.06E+00	-1.04	0.0491	-9.31E+00	9.31E+00
ENSMUSG00000000318	Clec10a	2.02E+00	2.02E+00	1.01	0.0482	9.20E+00	9.20E+00
ENSMUSG00000024132	Eci1	4.95E-01	-2.02E+00	-1.02	0.0487	-9.16E+00	9.16E+00
ENSMUSG00000033327	Tnxb	4.97E-01	-2.01E+00	-1.01	0.0490	-9.08E+00	9.08E+00
