# Supplementary material for: Aging-regulated PNUTS maintains endothelial barrier function via SEMA3B suppression
Source: Commun Biol. 2024 May 7;7:541. doi: 10.1038/s42003-024-06230-5 (PMC11076560; doi:10.1038/s42003-024-06230-5)
Supplement: Supplementary file 5 — Supplementary Data 3 [file 42003_2024_6230_MOESM5_ESM.pdf]

Table S2: RNA sequencing siPNUTS (KD) vs siControl (Ctr) HUVECs

ID	names	Ratio KD/Ctr	Fold change KD/Ctr	log2Ratio	PValue	R-factor	Abs R
ENSG00000007314	SCN4A	842.06	842.06	9.72	0.00E+00	#iDIV/0!	#iDIV/0!
ENSG00000198691	ABCA4	15.79	15.79	3.98	3.43E-164	8.52E+82	8.52E+82
ENSG00000144821	MYH15	21.65	21.65	4.44	3.63E-155	3.59E+78	3.59E+78
ENSG00000161281	COX7A1	0.04	-23.73	-4.57	1.24E-154	-2.13E+78	2.13E+78
ENSG00000111405	ENDOU	414.24	414.24	8.69	5.25E-151	5.72E+77	5.72E+77
ENSG00000255202	RP4-541C22.5	122.38	122.38	6.94	1.52E-151	3.14E+77	3.14E+77
ENSG00000002079	MYH16	37.46	37.46	5.23	5.53E-148	1.59E+75	1.59E+75
ENSG00000105825	TFPI2	0.11	-9.22	-3.20	1.61E-144	-7.27E+72	7.27E+72
ENSG00000244242	IFITM10	56.03	56.03	5.81	4.38E-139	8.47E+70	8.47E+70
ENSG00000135436	FAM186B	143.84	143.84	7.17	5.34E-137	1.97E+70	1.97E+70
ENSG00000103490	PYCARD	0.05	-19.75	-4.30	3.01E-130	-1.14E+66	1.14E+66
ENSG00000012171	SEMA3B	40.03	40.03	5.32	9.87E-128	1.27E+65	1.27E+65
ENSG00000110876	SELPLG	39.40	39.40	5.30	7.06E-125	4.69E+63	4.69E+63
ENSG00000248187	RP11-184M15.1	0.06	-15.42	-3.95	1.63E-122	-1.21E+62	1.21E+62
ENSG00000065618	COL17A1	7.99	7.99	3.00	2.38E-122	5.18E+61	5.18E+61
ENSG00000197558	SSPO	62.50	62.50	5.97	2.91E-120	3.66E+61	3.66E+61
ENSG00000169418	NPR1	0.10	-10.18	-3.35	2.78E-121	-1.93E+61	1.93E+61
ENSG00000203814	HIST2H2BF	0.13	-7.69	-2.94	3.54E-119	-1.29E+60	1.29E+60
ENSG00000107611	CUBN	8.35	8.35	3.06	1.15E-117	2.47E+59	2.47E+59
ENSG00000253741	CTD-2292P10.4	38.49	38.49	5.27	2.68E-112	2.35E+57	2.35E+57
ENSG00000183091	NEB	16.08	16.08	4.01	1.67E-110	1.25E+56	1.25E+56
ENSG00000146469	VIP	20.59	20.59	4.36	7.89E-110	7.33E+55	7.33E+55
ENSG00000151023	ENKUR	76.68	76.68	6.26	2.89E-108	4.51E+55	4.51E+55
ENSG00000243710	WDR65	10.75	10.75	3.43	6.05E-108	4.37E+54	4.37E+54
ENSG00000105499	PLA2G4C	8.93	8.93	3.16	1.28E-106	7.89E+53	7.89E+53
ENSG00000204947	ZNF425	8.71	8.71	3.12	6.06E-102	3.54E+51	3.54E+51
ENSG00000157343	ARMC12	53.06	53.06	5.73	7.24E-100	1.97E+51	1.97E+51
ENSG00000154734	ADAMTS1	0.18	-5.54	-2.47	3.63E-101	-9.20E+50	9.20E+50
ENSG00000178445	GLDC	5.60	5.60	2.48	1.37E-100	4.77E+50	4.77E+50
ENSG00000109193	SULT1E1	0.12	-8.42	-3.07	6.91E-100	-3.20E+50	3.20E+50
ENSG00000114270	COL7A1	28.41	28.41	4.83	1.05E-97	8.77E+49	8.77E+49
ENSG00000100342	APOL1	0.05	-19.60	-4.29	1.80E-96	-1.46E+49	1.46E+49
ENSG00000154864	PIEZO2	0.17	-6.02	-2.59	7.65E-96	-2.18E+48	2.18E+48
ENSG00000279686	ECSCR	0.18	-5.62	-2.49	2.70E-94	-3.42E+47	3.42E+47
ENSG00000246898	CTD-2258A20.4	10.03	10.03	3.33	2.52E-92	6.31E+46	6.31E+46
ENSG00000163491	NEK10	7.28	7.28	2.86	4.97E-92	3.26E+46	3.26E+46
ENSG00000068079	IFI35	0.05	-19.45	-4.28	7.35E-91	-2.27E+46	2.27E+46
ENSG00000221843	C2orf16	23.58	23.58	4.56	7.10E-90	8.85E+45	8.85E+45
ENSG00000264954	RP11-214C8.2	15.79	15.79	3.98	2.34E-89	3.26E+45	3.26E+45
ENSG00000241399	CD302	0.17	-5.93	-2.57	9.50E-90	-1.92E+45	1.92E+45
ENSG00000174059	CD34	5.03	5.03	2.33	7.33E-90	1.86E+45	1.86E+45
ENSG00000228857	AC104653.1	54.75	54.75	5.77	1.29E-87	1.53E+45	1.53E+45
ENSG00000050344	NFE2L3	0.18	-5.53	-2.47	3.24E-89	-9.72E+44	9.72E+44
ENSG00000169692	AGPAT2	0.09	-11.43	-3.51	1.67E-88	-8.84E+44	8.84E+44
ENSG00000178852	EFCAB13	5.87	5.87	2.55	1.99E-88	4.16E+44	4.16E+44
ENSG00000138449	SLC40A1	0.17	-5.84	-2.55	4.33E-86	-2.81E+43	2.81E+43
ENSG00000108515	ENO3	13.25	13.25	3.73	7.29E-85	1.55E+43	1.55E+43
ENSG00000113600	C9	61.66	61.66	5.95	7.32E-83	7.21E+42	7.21E+42
ENSG00000226496	LINC00323	33.48	33.48	5.07	8.47E-83	3.64E+42	3.64E+42
ENSG00000042832	TG	33.90	33.90	5.08	1.41E-82	2.85E+42	2.85E+42
ENSG00000163814	CDCP1	4.91	4.91	2.30	1.70E-81	1.19E+41	1.19E+41
ENSG00000113555	PCDH12	4.43	4.43	2.15	8.62E-80	1.51E+40	1.51E+40
ENSG00000197409	HIST1H3D	0.20	-4.91	-2.30	1.37E-79	-1.33E+40	1.33E+40
ENSG00000115594	IL1R1	0.21	-4.71	-2.24	6.69E-79	-5.76E+39	5.76E+39
ENSG00000110675	ELMOD1	0.22	-4.60	-2.20	6.83E-79	-5.57E+39	5.57E+39
ENSG00000266680	RP5-1148A21.3	7.57	7.57	2.92	2.05E-78	5.29E+39	5.29E+39
ENSG00000213344	PCNPP3	20.61	20.61	4.37	2.03E-77	4.58E+39	4.58E+39
ENSG00000154134	ROBO3	0.10	-10.15	-3.34	6.53E-77	-1.26E+39	1.26E+39
ENSG00000274911	RP11-12A20.7	7.41	7.41	2.89	4.08E-77	1.16E+39	1.16E+39
ENSG00000167874	TMEM88	0.12	-8.57	-3.10	6.50E-76	-3.36E+38	3.36E+38
ENSG00000115602	IL1RL1	0.22	-4.64	-2.21	3.64E-76	-2.43E+38	2.43E+38
ENSG00000184270	HIST2H2AB	0.22	-4.57	-2.19	2.19E-75	-9.76E+37	9.76E+37
ENSG00000122861	PLAU	4.70	4.70	2.23	2.35E-75	9.70E+37	9.70E+37
ENSG00000143107	FNDC7	375.95	375.95	8.55	2.27E-71	7.89E+37	7.89E+37
ENSG00000102575	ACP5	0.03	-32.84	-5.04	2.92E-73	-6.08E+37	6.08E+37
ENSG00000124875	CXCL6	0.15	-6.60	-2.72	1.50E-73	-1.71E+37	1.71E+37
ENSG00000198910	L1CAM	158.40	158.40	7.31	1.59E-69	3.98E+36	3.98E+36
ENSG00000135452	TSPAN31	0.19	-5.27	-2.40	3.84E-72	-2.69E+36	2.69E+36
ENSG00000168679	SLC16A4	0.10	-9.71	-3.28	1.82E-71	-2.28E+36	2.28E+36
ENSG00000170323	FABP4	0.16	-6.14	-2.62	1.10E-70	-5.86E+35	5.86E+35
ENSG00000072952	MRVI1	7.76	7.76	2.96	4.92E-70	3.50E+35	3.50E+35
ENSG00000189221	MAOA	0.25	-4.00	-2.00	3.51E-70	-2.13E+35	2.13E+35
ENSG00000196569	LAMA2	7.07	7.07	2.82	1.31E-69	1.95E+35	1.95E+35
ENSG00000163739	CXCL1	0.25	-3.95	-1.98	6.20E-70	-1.59E+35	1.59E+35
ENSG00000163810	TGM4	14.55	14.55	3.86	9.94E-69	1.46E+35	1.46E+35
ENSG00000146674	IGFBP3	0.05	-19.65	-4.30	3.34E-68	-1.08E+35	1.08E+35

ENSG00000179168	GGN	13.71	13.71	3.78	4.40E-68	6.54E+34	6.54E+34
ENSG00000186283	TOR3A	0.20	-4.95	-2.31	6.54E-69	-6.13E+34	6.13E+34
ENSG00000168490	PHYHIP	7.53	7.53	2.91	1.07E-66	7.28E+33	7.28E+33
ENSG00000134575	ACP2	0.23	-4.36	-2.12	7.83E-67	-4.93E+33	4.93E+33
ENSG00000082126	MPP4	4.06	4.06	2.02	1.25E-66	3.63E+33	3.63E+33
ENSG00000130783	CCDC62	10.24	10.24	3.36	1.04E-65	3.18E+33	3.18E+33
ENSG00000185291	IL3RA	0.07	-14.73	-3.88	1.62E-64	-1.16E+33	1.16E+33
ENSG00000185432	METTL7A	0.27	-3.74	-1.90	1.09E-65	-1.13E+33	1.13E+33
ENSG00000069812	HES2	4.84	4.84	2.28	2.42E-65	9.83E+32	9.83E+32
ENSG00000166670	MMP10	5.49	5.49	2.46	7.02E-65	6.56E+32	6.56E+32
ENSG00000139899	CBLN3	7.96	7.96	2.99	2.31E-64	5.24E+32	5.24E+32
ENSG00000186765	FSCN2	77.47	77.47	6.28	3.05E-62	4.44E+32	4.44E+32
ENSG00000138172	CALHM2	0.17	-5.79	-2.53	1.91E-64	-4.18E+32	4.18E+32
ENSG00000085465	OVGP1	13.93	13.93	3.80	2.20E-63	2.97E+32	2.97E+32
ENSG00000167191	GPRC5B	3.77	3.77	1.91	1.00E-63	1.19E+32	1.19E+32
ENSG00000133110	POSTN	0.03	-32.60	-5.03	1.60E-61	-8.14E+31	8.14E+31
ENSG00000246695	RP11-877E17.2	4.88	4.88	2.29	5.14E-63	6.81E+31	6.81E+31
ENSG00000169100	SLC25A6	0.16	-6.18	-2.63	8.58E-63	-6.67E+31	6.67E+31
ENSG00000023839	ABCC2	6.17	6.17	2.62	3.08E-62	3.51E+31	3.51E+31
ENSG00000119630	PGF	4.70	4.70	2.23	2.79E-62	2.82E+31	2.82E+31
ENSG00000065413	ANKRD44	4.36	4.36	2.13	3.77E-62	2.25E+31	2.25E+31
ENSG00000265972	TXNIP	0.22	-4.48	-2.16	5.11E-62	-1.98E+31	1.98E+31
ENSG00000125968	ID1	0.28	-3.58	-1.84	3.52E-62	-1.91E+31	1.91E+31
ENSG00000227811	RP4-773A18.4	9.00	9.00	3.17	3.38E-61	1.55E+31	1.55E+31
ENSG00000140873	ADAMTS18	0.23	-4.29	-2.10	2.18E-61	-9.18E+30	9.18E+30
ENSG00000188818	ZDHHC11	27.11	27.11	4.76	9.27E-60	8.91E+30	8.91E+30
ENSG00000188596	C12orf55	5.48	5.48	2.45	5.17E-61	7.62E+30	7.62E+30
ENSG00000260941	LINC00622	13.39	13.39	3.74	3.94E-60	6.75E+30	6.75E+30
ENSG00000133800	LYVE1	0.23	-4.43	-2.15	4.60E-61	-6.53E+30	6.53E+30
ENSG00000175182	FAM131A	0.24	-4.22	-2.08	5.76E-61	-5.57E+30	5.57E+30
ENSG00000185313	SCN10A	27.35	27.35	4.77	3.49E-59	4.63E+30	4.63E+30
ENSG00000089847	ANKRD24	25.01	25.01	4.64	5.71E-59	3.31E+30	3.31E+30
ENSG00000146678	IGFBP1	0.14	-7.33	-2.87	5.08E-60	-3.25E+30	3.25E+30
ENSG00000116299	KIAA1324	5.94	5.94	2.57	7.44E-60	2.18E+30	2.18E+30
ENSG00000134245	WNT2B	6.13	6.13	2.61	1.01E-59	1.93E+30	1.93E+30
ENSG00000168477	TNXB	30.32	30.32	4.92	3.62E-58	1.59E+30	1.59E+30
ENSG00000167272	POP5	0.23	-4.36	-2.12	1.76E-59	-1.04E+30	1.04E+30
ENSG00000169169	CPT1C	0.24	-4.09	-2.03	2.11E-59	-8.90E+29	8.90E+29
ENSG00000135407	AVIL	7.99	7.99	3.00	3.46E-58	4.30E+29	4.30E+29
ENSG00000240929	HIST2H2BB	0.16	-6.43	-2.69	2.26E-58	-4.28E+29	4.28E+29
ENSG00000123374	CDK2	0.29	-3.51	-1.81	3.04E-58	-2.01E+29	2.01E+29
ENSG00000138185	ENTPD1	0.29	-3.49	-1.80	3.27E-58	-1.93E+29	1.93E+29
ENSG00000232926	AC000078.5	14.66	14.66	3.87	2.29E-56	9.68E+28	9.68E+28
ENSG00000103024	NME3	0.21	-4.81	-2.27	5.74E-57	-6.34E+28	6.34E+28
ENSG00000204482	LST1	17.01	17.01	4.09	1.07E-55	5.19E+28	5.19E+28
ENSG00000189060	H1F0	0.23	-4.39	-2.13	8.23E-57	-4.84E+28	4.84E+28
ENSG00000154639	CXADR	4.07	4.07	2.02	9.71E-57	4.13E+28	4.13E+28
ENSG00000115129	TP53I3	0.30	-3.37	-1.75	8.69E-57	-3.62E+28	3.62E+28
ENSG00000275379	HIST1H3I	0.23	-4.41	-2.14	1.77E-56	-3.31E+28	3.31E+28
ENSG00000184381	PLA2G6	6.11	6.11	2.61	4.83E-56	2.78E+28	2.78E+28
ENSG00000129255	MPDU1	0.29	-3.42	-1.78	1.61E-56	-2.69E+28	2.69E+28
ENSG00000136928	GABBR2	0.29	-3.44	-1.78	5.00E-56	-1.54E+28	1.54E+28
ENSG00000140465	CYP1A1	0.23	-4.43	-2.15	8.61E-56	-1.51E+28	1.51E+28
ENSG00000183598	HIST2H3D	0.18	-5.42	-2.44	2.01E-55	-1.21E+28	1.21E+28
ENSG00000137434	C6orf52	18.40	18.40	4.20	2.48E-54	1.17E+28	1.17E+28
ENSG00000203818	HIST2H3PS2	0.14	-7.15	-2.84	1.26E-54	-6.37E+27	6.37E+27
ENSG00000049769	PPP1R3F	5.82	5.82	2.54	8.61E-55	6.27E+27	6.27E+27
ENSG00000275713	HIST1H2BH	0.27	-3.73	-1.90	4.19E-54	-1.82E+27	1.82E+27
ENSG00000102760	RGCC	3.42	3.42	1.77	7.74E-54	1.23E+27	1.23E+27
ENSG00000135480	KRT7	4.33	4.33	2.12	1.34E-53	1.19E+27	1.19E+27
ENSG00000125995	ROMO1	0.28	-3.63	-1.86	1.21E-53	-1.04E+27	1.04E+27
ENSG00000171703	TCEA2	3.66	3.66	1.87	4.22E-53	5.64E+26	5.64E+26
ENSG00000164946	FREM1	10.68	10.68	3.42	9.65E-52	3.44E+26	3.44E+26
ENSG00000108846	ABCC3	8.50	8.50	3.09	1.73E-51	2.05E+26	2.05E+26
ENSG00000121966	CXCR4	4.69	4.69	2.23	1.33E-51	1.29E+26	1.29E+26
ENSG00000063127	SLC6A16	11.98	11.98	3.58	1.58E-50	9.53E+25	9.53E+25
ENSG00000125775	SDCBP2	4.26	4.26	2.09	6.76E-51	5.18E+25	5.18E+25
ENSG00000108379	WNT3	4.15	4.15	2.05	8.36E-51	4.54E+25	4.54E+25
ENSG00000257086	RP11-783K16.13	5.97	5.97	2.58	2.04E-50	4.18E+25	4.18E+25
ENSG00000188042	ARL4C	5.34	5.34	2.42	2.77E-50	3.21E+25	3.21E+25
ENSG00000156265	C21orf7	3.93	3.93	1.97	1.75E-50	2.97E+25	2.97E+25
ENSG00000262001	DLGAP1-AS2	3.19	3.19	1.67	1.23E-50	2.87E+25	2.87E+25
ENSG00000278637	HIST1H4A	0.25	-3.99	-2.00	1.95E-50	-2.86E+25	2.86E+25
ENSG00000169604	ANTXR1	4.60	4.60	2.20	5.73E-50	1.92E+25	1.92E+25
ENSG00000149476	DAK	3.57	3.57	1.84	1.61E-49	8.91E+24	8.91E+24
ENSG00000112208	BAG2	0.31	-3.22	-1.69	1.42E-49	-8.54E+24	8.54E+24
ENSG00000058085	LAMC2	4.28	4.28	2.10	5.16E-49	5.96E+24	5.96E+24
ENSG00000186854	TRABD2A	9.59	9.59	3.26	3.20E-48	5.36E+24	5.36E+24
ENSG00000113583	C5orf15	0.30	-3.36	-1.75	6.04E-49	-4.32E+24	4.32E+24

ENSG00000120437	ACAT2	0.31	-3.20	-1.68	6.45E-49	-3.98E+24	3.98E+24
ENSG00000272167	PROX1-AS1	8.36	8.36	3.06	5.46E-48	3.58E+24	3.58E+24
ENSG00000272031	ANKRD34A	8.95	8.95	3.16	6.59E-48	3.49E+24	3.49E+24
ENSG00000134070	IRAK2	3.24	3.24	1.70	1.58E-48	2.58E+24	2.58E+24
ENSG00000185860	C1orf110	3.92	3.92	1.97	2.69E-48	2.39E+24	2.39E+24
ENSG00000134516	DOCK2	5.16	5.16	2.37	6.47E-48	2.03E+24	2.03E+24
ENSG00000010295	IFFO1	0.26	-3.90	-1.96	4.82E-48	-1.77E+24	1.77E+24
ENSG00000232759	AC002480.3	0.24	-4.21	-2.07	7.12E-48	-1.58E+24	1.58E+24
ENSG00000278828	HIST1H3H	0.25	-3.98	-1.99	1.74E-47	-9.53E+23	9.53E+23
ENSG00000158373	HIST1H2BD	0.26	-3.78	-1.92	3.55E-47	-6.34E+23	6.34E+23
ENSG00000185000	DGAT1	0.26	-3.86	-1.95	4.81E-47	-5.57E+23	5.57E+23
ENSG00000091137	SLC26A4	8.33	8.33	3.06	3.03E-46	4.78E+23	4.78E+23
ENSG00000118777	ABCG2	0.30	-3.37	-1.75	5.67E-47	-4.47E+23	4.47E+23
ENSG00000072778	ACADVL	0.29	-3.47	-1.80	6.99E-47	-4.15E+23	4.15E+23
ENSG00000169715	MT1E	0.29	-3.44	-1.78	7.16E-47	-4.07E+23	4.07E+23
ENSG00000196890	HIST3H2BB	0.18	-5.57	-2.48	1.87E-46	-4.07E+23	4.07E+23
ENSG00000145476	CYP4V2	5.38	5.38	2.43	3.41E-46	2.91E+23	2.91E+23
ENSG00000187325	TAF9B	0.26	-3.81	-1.93	1.81E-46	-2.83E+23	2.83E+23
ENSG00000240583	AQP1	0.27	-3.69	-1.88	1.71E-46	-2.82E+23	2.82E+23
ENSG00000107821	KAZALD1	6.29	6.29	2.65	5.64E-46	2.65E+23	2.65E+23
ENSG00000158683	PKD1L1	4.85	4.85	2.28	3.48E-46	2.60E+23	2.60E+23
ENSG00000157653	C9orf43	6.16	6.16	2.62	1.93E-45	1.40E+23	1.40E+23
ENSG00000165092	ALDH1A1	0.32	-3.16	-1.66	6.33E-46	-1.25E+23	1.25E+23
ENSG00000276180	HIST1H4I	0.25	-4.05	-2.02	1.58E-45	-1.02E+23	1.02E+23
ENSG00000254860	TMEM9B-AS1	7.77	7.77	2.96	2.31E-44	5.11E+22	5.11E+22
ENSG00000204390	HSPA1L	7.99	7.99	3.00	3.53E-44	4.26E+22	4.26E+22
ENSG00000180198	RCC1	3.13	3.13	1.64	8.44E-45	3.40E+22	3.40E+22
ENSG00000103034	NDRG4	0.35	-2.89	-1.53	8.39E-45	-3.16E+22	3.16E+22
ENSG00000082074	FYB	22.95	22.95	4.52	7.73E-43	2.61E+22	2.61E+22
ENSG00000227603	RP11-165J3.6	16.47	16.47	4.04	5.25E-43	2.27E+22	2.27E+22
ENSG00000172379	ARNT2	13.27	13.27	3.73	4.84E-43	1.91E+22	1.91E+22
ENSG00000153707	PTPRD	0.34	-2.92	-1.55	2.73E-44	-1.77E+22	1.77E+22
ENSG00000234684	SDCBP2-AS1	8.72	8.72	3.12	3.10E-43	1.57E+22	1.57E+22
ENSG00000177675	CD163L1	3.43	3.43	1.78	5.73E-44	1.43E+22	1.43E+22
ENSG00000189050	RNFT1	0.23	-4.28	-2.10	9.48E-44	-1.39E+22	1.39E+22
ENSG00000163661	PTX3	0.28	-3.58	-1.84	7.68E-44	-1.29E+22	1.29E+22
ENSG00000160207	HSF2BP	8.01	8.01	3.00	5.50E-43	1.08E+22	1.08E+22
ENSG00000262814	MRPL12	0.18	-5.41	-2.44	2.63E-43	-1.05E+22	1.05E+22
ENSG00000205981	DNAJC19	0.33	-3.06	-1.61	1.22E-43	-8.76E+21	8.76E+21
ENSG00000160050	CCDC28B	0.21	-4.73	-2.24	3.37E-43	-8.14E+21	8.14E+21
ENSG00000204311	DFNB59	6.91	6.91	2.79	1.28E-42	6.10E+21	6.10E+21
ENSG00000153094	BCL2L11	3.41	3.41	1.77	3.24E-43	5.99E+21	5.99E+21
ENSG00000221829	FANCG	0.33	-3.07	-1.62	3.40E-43	-5.27E+21	5.27E+21
ENSG00000081087	OSTM1	2.85	2.85	1.51	2.97E-43	5.23E+21	5.23E+21
ENSG00000076554	TPD52	0.30	-3.35	-1.74	4.12E-43	-5.22E+21	5.22E+21
ENSG00000244694	PTCHD4	3.56	3.56	1.83	1.28E-42	3.14E+21	3.14E+21
ENSG00000196611	MMP1	4.19	4.19	2.07	1.81E-42	3.12E+21	3.12E+21
ENSG00000111684	LPCAT3	0.28	-3.59	-1.84	2.78E-42	-2.15E+21	2.15E+21
ENSG00000261253	AC137932.6	7.99	7.99	3.00	3.30E-41	1.39E+21	1.39E+21
ENSG00000125864	BFSP1	3.23	3.23	1.69	1.52E-41	8.30E+20	8.30E+20
ENSG00000172638	EFEMP2	0.35	-2.88	-1.52	1.34E-41	-7.85E+20	7.85E+20
ENSG00000163584	RPL22L1	0.31	-3.21	-1.68	1.74E-41	-7.68E+20	7.68E+20
ENSG00000204248	COL11A2	10.09	10.09	3.34	2.71E-40	6.14E+20	6.14E+20
ENSG00000067445	TRO	0.29	-3.43	-1.78	4.52E-41	-5.10E+20	5.10E+20
ENSG00000196074	SYCP2	10.73	10.73	3.42	4.91E-40	4.84E+20	4.84E+20
ENSG00000129646	QRICH2	4.21	4.21	2.07	8.12E-41	4.67E+20	4.67E+20
ENSG00000273703	HIST1H2BM	0.33	-3.05	-1.61	4.53E-41	-4.53E+20	4.53E+20
ENSG00000123689	G0S2	0.24	-4.25	-2.09	9.66E-41	-4.33E+20	4.33E+20
ENSG00000261903.1	RP1-59D14.6	6.91	6.91	2.79	3.01E-40	3.98E+20	3.98E+20
ENSG00000126749	EMG1	0.23	-4.35	-2.12	1.20E-40	-3.97E+20	3.97E+20
ENSG00000182179	UBA7	0.31	-3.18	-1.67	6.99E-41	-3.80E+20	3.80E+20
ENSG00000277157	HIST1H4D	0.33	-3.01	-1.59	1.46E-40	-2.49E+20	2.49E+20
ENSG00000142252	GEMIN7	0.29	-3.39	-1.76	1.94E-40	-2.43E+20	2.43E+20
ENSG00000255775	RP3-454B23.1	8.32	8.32	3.06	1.22E-39	2.38E+20	2.38E+20
ENSG00000274290	HIST1H2BE	0.33	-3.02	-1.59	2.06E-40	-2.10E+20	2.10E+20
ENSG00000175264	CHST1	3.08	3.08	1.62	2.55E-40	1.93E+20	1.93E+20
ENSG00000135414	GDF11	0.28	-3.62	-1.86	4.30E-40	-1.75E+20	1.75E+20
ENSG00000137090	DMRT1	6.24	6.24	2.64	1.33E-39	1.72E+20	1.72E+20
ENSG00000163945	UVSSA	3.31	3.31	1.72	5.54E-40	1.40E+20	1.40E+20
ENSG00000184441	AP001062.7	7.16	7.16	2.84	3.42E-39	1.22E+20	1.22E+20
ENSG00000165511	C10orf25	3.60	3.60	1.85	1.31E-39	9.96E+19	9.96E+19
ENSG00000145247	OCIAD2	3.03	3.03	1.60	1.59E-39	7.59E+19	7.59E+19
ENSG00000113319	RASGRF2	6.41	6.41	2.68	7.42E-39	7.44E+19	7.44E+19
ENSG00000245648	RP11-277P12.20	5.64	5.64	2.50	6.51E-39	6.99E+19	6.99E+19
ENSG00000144354	CDCA7	0.33	-3.02	-1.59	1.94E-39	-6.86E+19	6.86E+19
ENSG00000176697	BDNF	0.24	-4.09	-2.03	3.61E-39	-6.80E+19	6.80E+19
ENSG00000236252	RP11-15J10.8	9.63	9.63	3.27	2.38E-38	6.24E+19	6.24E+19
ENSG00000267858	AC016629.8	6.05	6.05	2.60	1.25E-38	5.41E+19	5.41E+19
ENSG00000182257	C22orf26	9.08	9.08	3.18	3.17E-38	5.10E+19	5.10E+19

ENSG00000257732	RP11-818F20.5	5.76	5.76	2.53	1.30E-38	5.05E+19	5.05E+19
ENSG00000132563	REEP2	0.13	-7.58	-2.92	3.04E-38	-4.35E+19	4.35E+19
ENSG00000243587	C6orf183	10.81	10.81	3.43	6.25E-38	4.32E+19	4.32E+19
ENSG00000198832	RP3-412A9.11	0.23	-4.42	-2.14	1.42E-38	-3.71E+19	3.71E+19
ENSG00000145349	CAMK2D	0.37	-2.72	-1.45	6.34E-39	-3.42E+19	3.42E+19
ENSG00000196169	KIF19	8.07	8.07	3.01	5.62E-38	3.41E+19	3.41E+19
ENSG00000102048	ASB9	0.27	-3.66	-1.87	1.26E-38	-3.25E+19	3.25E+19
ENSG00000114841	DNAH1	3.66	3.66	1.87	1.57E-38	2.92E+19	2.92E+19
ENSG00000107833	NPM3	0.35	-2.86	-1.51	2.40E-38	-1.84E+19	1.84E+19
ENSG00000213433	RP11-54C4.1	9.42	9.42	3.24	2.64E-37	1.83E+19	1.83E+19
ENSG00000149926	FAM57B	10.25	10.25	3.36	3.20E-37	1.81E+19	1.81E+19
ENSG00000278588	HIST1H2BI	0.34	-2.92	-1.55	3.87E-38	-1.48E+19	1.48E+19
ENSG00000117152	RGS4	0.33	-3.00	-1.59	4.48E-38	-1.42E+19	1.42E+19
ENSG00000108602	ALDH3A1	11.24	11.24	3.49	6.56E-37	1.39E+19	1.39E+19
ENSG00000134873	CLDN10	0.11	-9.41	-3.23	5.06E-37	-1.32E+19	1.32E+19
ENSG00000122971	ACADS	0.25	-4.05	-2.02	9.85E-38	-1.29E+19	1.29E+19
ENSG00000090861	AARS	0.36	-2.78	-1.48	6.52E-38	-1.09E+19	1.09E+19
ENSG00000276644	DACH1	3.27	3.27	1.71	1.07E-37	9.99E+18	9.99E+18
ENSG00000117620	SLC35A3	0.35	-2.88	-1.53	8.64E-38	-9.81E+18	9.81E+18
ENSG00000128567	PODXL	3.17	3.17	1.67	1.49E-37	8.23E+18	8.23E+18
ENSG00000067836	ROGDI	0.26	-3.83	-1.94	2.20E-37	-8.17E+18	8.17E+18
ENSG00000173581	CCDC106	0.26	-3.85	-1.94	3.87E-37	-6.18E+18	6.18E+18
ENSG00000146007	ZMAT2	0.38	-2.63	-1.39	2.31E-37	-5.47E+18	5.47E+18
ENSG00000163346	PBXIP1	0.38	-2.65	-1.40	2.54E-37	-5.25E+18	5.25E+18
ENSG00000204839	C8orf73	8.99	8.99	3.17	3.40E-36	4.87E+18	4.87E+18
ENSG00000171848	RRM2	0.33	-3.05	-1.61	4.38E-37	-4.60E+18	4.60E+18
ENSG00000185130	HIST1H2BL	0.38	-2.64	-1.40	3.34E-37	-4.57E+18	4.57E+18
ENSG00000179397	C1orf101	5.07	5.07	2.34	1.26E-36	4.52E+18	4.52E+18
ENSG00000120832	MTERFD3	0.31	-3.25	-1.70	5.21E-37	-4.50E+18	4.50E+18
ENSG00000131480	AOC2	8.25	8.25	3.04	3.37E-36	4.49E+18	4.49E+18
ENSG00000036448	MYOM2	8.96	8.96	3.16	6.19E-36	3.60E+18	3.60E+18
ENSG00000178538	CA8	0.29	-3.39	-1.76	1.04E-36	-3.32E+18	3.32E+18
ENSG00000258727	RP11-66N24.3	3.27	3.27	1.71	1.01E-36	3.25E+18	3.25E+18
ENSG00000183496	MEX3B	2.79	2.79	1.48	7.37E-37	3.25E+18	3.25E+18
ENSG00000070444	MNT	3.48	3.48	1.80	1.16E-36	3.23E+18	3.23E+18
ENSG00000205352	PRR13	0.37	-2.69	-1.43	8.49E-37	-2.92E+18	2.92E+18
ENSG00000260604	RP1-140K8.5	2.56	2.56	1.36	8.25E-37	2.82E+18	2.82E+18
ENSG00000173818	ENDOV	2.91	2.91	1.54	1.41E-36	2.45E+18	2.45E+18
ENSG00000141682	PMAIP1	2.64	2.64	1.40	1.31E-36	2.31E+18	2.31E+18
ENSG00000136383	ALPK3	0.35	-2.82	-1.50	1.87E-36	-2.06E+18	2.06E+18
ENSG00000107960	OBFC1	2.65	2.65	1.41	2.23E-36	1.77E+18	1.77E+18
ENSG00000110077	MS4A6A	0.22	-4.58	-2.19	9.75E-36	-1.47E+18	1.47E+18
ENSG00000102385	DRP2	5.01	5.01	2.32	1.37E-35	1.35E+18	1.35E+18
ENSG00000169429	IL8	0.37	-2.69	-1.43	4.74E-36	-1.24E+18	1.24E+18
ENSG00000005238	FAM214B	2.61	2.61	1.38	5.50E-36	1.11E+18	1.11E+18
ENSG00000137573	SULF1	0.16	-6.27	-2.65	3.44E-35	-1.07E+18	1.07E+18
ENSG00000143772	ITPKB	2.74	2.74	1.45	9.52E-36	8.88E+17	8.88E+17
ENSG00000170522	ELOVL6	0.35	-2.82	-1.50	1.42E-35	-7.48E+17	7.48E+17
ENSG00000203778	C6orf225	0.28	-3.60	-1.85	2.33E-35	-7.46E+17	7.46E+17
ENSG00000132199	ENOSF1	0.37	-2.70	-1.43	1.63E-35	-6.68E+17	6.68E+17
	RP11-77I22.3	3.21	3.21	1.68	3.22E-35	5.65E+17	5.65E+17
ENSG00000138772	ANXA3	2.68	2.68	1.42	2.98E-35	4.90E+17	4.90E+17
ENSG00000100784	RPS6KA5	0.31	-3.18	-1.67	5.69E-35	-4.21E+17	4.21E+17
ENSG00000050438	SLC4A8	2.59	2.59	1.37	3.90E-35	4.15E+17	4.15E+17
ENSG00000226149	RP1-69D17.4	4.82	4.82	2.27	1.52E-34	3.91E+17	3.91E+17
ENSG00000163638	ADAMTS9	0.37	-2.74	-1.45	6.32E-35	-3.44E+17	3.44E+17
ENSG00000170421	KRT8	2.57	2.57	1.36	7.15E-35	3.04E+17	3.04E+17
ENSG00000135218	CD36	0.24	-4.25	-2.09	2.19E-34	-2.87E+17	2.87E+17
ENSG00000198715	C1orf85	0.39	-2.53	-1.34	8.22E-35	-2.79E+17	2.79E+17
	RP1-180E22.3	4.97	4.97	2.31	3.39E-34	2.70E+17	2.70E+17
ENSG00000156804	FBXO32	3.43	3.43	1.78	2.82E-34	2.04E+17	2.04E+17
ENSG00000169592	INO80E	0.40	-2.50	-1.32	1.88E-34	-1.83E+17	1.83E+17
ENSG00000244588	RAD21L1	10.67	10.67	3.42	3.80E-33	1.73E+17	1.73E+17
ENSG00000091947	TMEM101	0.38	-2.62	-1.39	3.43E-34	-1.42E+17	1.42E+17
ENSG00000056558	TRAF1	4.24	4.24	2.08	1.41E-33	1.13E+17	1.13E+17
ENSG00000167740	CYB5D2	2.54	2.54	1.34	5.95E-34	1.04E+17	1.04E+17
ENSG00000048342	CC2D2A	0.38	-2.63	-1.39	6.66E-34	-1.02E+17	1.02E+17
ENSG00000156042	TTC18	7.25	7.25	2.86	5.13E-33	1.01E+17	1.01E+17
ENSG00000137463	MGARP	0.32	-3.11	-1.64	1.04E-33	-9.66E+16	9.66E+16
ENSG00000112855	HARS2	0.37	-2.67	-1.42	8.97E-34	-8.91E+16	8.91E+16
ENSG00000197140	ADAM32	3.08	3.08	1.62	1.41E-33	8.22E+16	8.22E+16
ENSG00000228649	AC005682.5	0.25	-3.96	-1.99	2.34E-33	-8.19E+16	8.19E+16
ENSG00000196562	SULF2	2.74	2.74	1.46	1.80E-33	6.47E+16	6.47E+16
ENSG00000230753	RP4-553F4.6	5.73	5.73	2.52	1.27E-32	5.08E+16	5.08E+16
ENSG00000115221	ITGB6	14.71	14.71	3.88	8.41E-32	5.07E+16	5.07E+16
ENSG00000007312	CD79B	4.84	4.84	2.28	9.30E-33	5.02E+16	5.02E+16
ENSG00000151062	CACNA2D4	6.05	6.05	2.60	2.33E-32	3.96E+16	3.96E+16
ENSG00000038945	MSR1	7.81	7.81	2.97	4.11E-32	3.85E+16	3.85E+16
ENSG00000134072	CAMK1	0.39	-2.54	-1.34	4.35E-33	-3.85E+16	3.85E+16

ENSG00000244879	GABPB1-AS1	2.95	2.95	1.56	7.34E-33	3.45E+16	3.45E+16
ENSG00000172500	FIBP	0.39	-2.55	-1.35	6.27E-33	-3.22E+16	3.22E+16
ENSG00000148297	MED22	0.39	-2.57	-1.36	6.72E-33	-3.13E+16	3.13E+16
ENSG00000124641	MED20	0.41	-2.44	-1.29	6.39E-33	-3.05E+16	3.05E+16
ENSG00000108423	TUBD1	0.29	-3.46	-1.79	1.43E-32	-2.89E+16	2.89E+16
ENSG00000137033	IL33	0.20	-4.90	-2.29	6.57E-32	-1.91E+16	1.91E+16
ENSG00000143416	SELENBP1	0.35	-2.87	-1.52	2.38E-32	-1.86E+16	1.86E+16
ENSG00000197927	C2orf27A	3.31	3.31	1.73	3.37E-32	1.80E+16	1.80E+16
ENSG00000144476	CXCR7	0.34	-2.93	-1.55	3.22E-32	-1.64E+16	1.64E+16
ENSG00000226742	HSBP1L1	0.14	-7.02	-2.81	2.15E-31	-1.51E+16	1.51E+16
ENSG00000127533	F2RL3	6.80	6.80	2.76	2.07E-31	1.49E+16	1.49E+16
ENSG00000179818	PCBP1-AS1	2.81	2.81	1.49	3.65E-32	1.47E+16	1.47E+16
ENSG00000115604	IL18R1	0.39	-2.57	-1.36	3.29E-32	-1.42E+16	1.42E+16
ENSG00000148290	SURF1	0.33	-3.05	-1.61	6.42E-32	-1.20E+16	1.20E+16
ENSG00000100116	GCAT	0.36	-2.76	-1.47	5.50E-32	-1.18E+16	1.18E+16
ENSG00000167767	KRT80	2.54	2.54	1.35	4.71E-32	1.17E+16	1.17E+16
ENSG00000138380	ALS2CR8	3.48	3.48	1.80	9.69E-32	1.12E+16	1.12E+16
ENSG00000155363	MOV10	0.39	-2.54	-1.35	5.27E-32	-1.11E+16	1.11E+16
ENSG00000163702	IL17RC	0.34	-2.91	-1.54	7.27E-32	-1.08E+16	1.08E+16
ENSG00000137727	ARHGAP20	0.32	-3.16	-1.66	8.72E-32	-1.07E+16	1.07E+16
ENSG00000157404	KIT	3.29	3.29	1.72	1.16E-31	9.64E+15	9.64E+15
ENSG00000182118	FAM89A	2.95	2.95	1.56	1.43E-31	7.78E+15	7.78E+15
ENSG00000148288	GBGT1	0.34	-2.98	-1.58	1.50E-31	-7.69E+15	7.69E+15
ENSG00000168890	TMEM150A	0.32	-3.11	-1.64	1.70E-31	-7.54E+15	7.54E+15
ENSG00000144583	MARCH4	2.83	2.83	1.50	1.55E-31	7.18E+15	7.18E+15
ENSG00000164088	PPM1M	0.35	-2.83	-1.50	1.62E-31	-7.04E+15	7.04E+15
ENSG00000136449	MYCBPAP	5.36	5.36	2.42	6.10E-31	6.85E+15	6.85E+15
ENSG00000184678	HIST2H2BE	0.38	-2.61	-1.38	1.72E-31	-6.30E+15	6.30E+15
ENSG00000245025	RP11-875O11.1	5.50	5.50	2.46	8.78E-31	5.87E+15	5.87E+15
ENSG00000213462	ERV3-1	2.98	2.98	1.57	2.85E-31	5.58E+15	5.58E+15
ENSG00000140398	NEIL1	0.29	-3.50	-1.81	4.15E-31	-5.43E+15	5.43E+15
ENSG00000203667	COX20	0.38	-2.65	-1.41	2.66E-31	-5.13E+15	5.13E+15
ENSG00000224428.2	RP11-182L21.2	2.67	2.67	1.42	2.75E-31	5.10E+15	5.10E+15
ENSG00000110844	PRPF40B	0.26	-3.88	-1.96	5.90E-31	-5.05E+15	5.05E+15
	AC009236.1	9.97	9.97	3.32	3.92E-30	5.04E+15	5.04E+15
ENSG00000108641	B9D1	0.36	-2.82	-1.49	3.28E-31	-4.91E+15	4.91E+15
ENSG00000135324	MRAP2	0.36	-2.79	-1.48	3.41E-31	-4.77E+15	4.77E+15
ENSG00000188921	PTPLAD2	0.28	-3.54	-1.82	5.62E-31	-4.72E+15	4.72E+15
ENSG00000107537	PHYH	2.53	2.53	1.34	2.90E-31	4.69E+15	4.69E+15
ENSG00000129667	RHBDF2	3.83	3.83	1.94	7.38E-31	4.46E+15	4.46E+15
ENSG00000114395	CYB561D2	0.30	-3.31	-1.73	6.29E-31	-4.18E+15	4.18E+15
ENSG00000162062	C16orf59	0.27	-3.72	-1.89	9.10E-31	-3.90E+15	3.90E+15
ENSG00000141338	ABCA8	0.18	-5.63	-2.49	2.12E-30	-3.87E+15	3.87E+15
ENSG00000008130	NADK	0.41	-2.46	-1.30	5.68E-31	-3.27E+15	3.27E+15
ENSG00000135482	ZC3H10	0.30	-3.36	-1.75	1.12E-30	-3.18E+15	3.18E+15
ENSG00000128815	WDFY4	2.55	2.55	1.35	7.50E-31	2.94E+15	2.94E+15
ENSG00000188277	C15orf62	8.82	8.82	3.14	9.03E-30	2.93E+15	2.93E+15
ENSG00000142002	DPP9	2.71	2.71	1.44	9.37E-31	2.80E+15	2.80E+15
ENSG00000225339	RP11-513I15.6	4.95	4.95	2.31	4.01E-30	2.47E+15	2.47E+15
ENSG00000136840	ST6GALNAC4	0.42	-2.36	-1.24	9.35E-31	-2.44E+15	2.44E+15
ENSG00000197696	NMB	0.28	-3.55	-1.83	2.32E-30	-2.33E+15	2.33E+15
ENSG00000136982	DSCC1	0.35	-2.82	-1.50	1.57E-30	-2.25E+15	2.25E+15
ENSG00000077585	GPR137B	2.75	2.75	1.46	1.54E-30	2.22E+15	2.22E+15
ENSG00000261597	RP11-353B9.1	6.29	6.29	2.65	9.65E-30	2.02E+15	2.02E+15
ENSG00000170801	HTRA3	2.83	2.83	1.50	2.08E-30	1.96E+15	1.96E+15
ENSG00000198270	TMEM116	4.18	4.18	2.06	4.57E-30	1.95E+15	1.95E+15
ENSG00000163879	DNALI1	0.22	-4.64	-2.21	5.80E-30	-1.93E+15	1.93E+15
	RP5-1014C4.3	6.41	6.41	2.68	1.11E-29	1.92E+15	1.92E+15
ENSG00000111266	DUSP16	2.45	2.45	1.29	1.64E-30	1.92E+15	1.92E+15
ENSG00000203791	METTL10	0.31	-3.23	-1.69	3.13E-30	-1.83E+15	1.83E+15
ENSG00000162779	AXDND1	5.25	5.25	2.39	8.51E-30	1.80E+15	1.80E+15
ENSG00000196468	FGF16	2.84	2.84	1.51	2.81E-30	1.70E+15	1.70E+15
ENSG00000230487	PSMG3-AS1	3.63	3.63	1.86	5.05E-30	1.62E+15	1.62E+15
ENSG00000143224	PPOX	0.34	-2.91	-1.54	3.40E-30	-1.58E+15	1.58E+15
ENSG00000102879	CORO1A	0.15	-6.89	-2.78	1.99E-29	-1.55E+15	1.55E+15
ENSG00000104524	PYCRL	0.35	-2.84	-1.51	3.89E-30	-1.44E+15	1.44E+15
ENSG00000107175	CREB3	0.42	-2.36	-1.24	3.25E-30	-1.31E+15	1.31E+15
ENSG00000162066	AMDHD2	0.37	-2.74	-1.45	5.00E-30	-1.22E+15	1.22E+15
ENSG00000108528	SLC25A11	0.43	-2.35	-1.23	3.89E-30	-1.19E+15	1.19E+15
ENSG00000130513	GDF15	2.82	2.82	1.50	5.76E-30	1.18E+15	1.18E+15
ENSG00000242259	C22orf39	0.39	-2.58	-1.37	4.97E-30	-1.16E+15	1.16E+15
ENSG00000136630	HLX	3.03	3.03	1.60	8.33E-30	1.05E+15	1.05E+15
ENSG00000276410	HIST1H2BB	0.38	-2.64	-1.40	6.98E-30	-9.99E+14	9.99E+14
ENSG00000197153	HIST1H3J	0.40	-2.48	-1.31	6.26E-30	-9.93E+14	9.93E+14
ENSG00000197837	HIST4H4	0.35	-2.83	-1.50	1.03E-29	-8.83E+14	8.83E+14
ENSG00000236824	BCYRN1	0.32	-3.10	-1.63	1.85E-29	-7.21E+14	7.21E+14
ENSG00000091972	CD200	0.28	-3.52	-1.82	2.54E-29	-6.98E+14	6.98E+14
ENSG00000250565	ATP6V1E2	3.08	3.08	1.62	2.10E-29	6.72E+14	6.72E+14
ENSG00000124721	DNAH8	3.87	3.87	1.95	4.05E-29	6.09E+14	6.09E+14

ENSG00000149823	VPS51	0.41	-2.44	-1.29	2.25E-29	-5.15E+14	5.15E+14
ENSG00000197989	SNHG12	0.31	-3.25	-1.70	5.65E-29	-4.33E+14	4.33E+14
ENSG00000149781	FERMT3	0.39	-2.56	-1.36	4.78E-29	-3.70E+14	3.70E+14
ENSG00000235217	TSPY26P	0.26	-3.83	-1.94	1.11E-28	-3.63E+14	3.63E+14
ENSG00000139880	CDH24	0.30	-3.29	-1.72	8.27E-29	-3.62E+14	3.62E+14
ENSG00000155465	SLC7A7	2.45	2.45	1.29	4.66E-29	3.59E+14	3.59E+14
ENSG00000091879	ANGPT2	3.01	3.01	1.59	7.11E-29	3.56E+14	3.56E+14
ENSG00000184785	CXorf69	0.27	-3.74	-1.90	1.11E-28	-3.55E+14	3.55E+14
ENSG00000162733	DDR2	0.41	-2.41	-1.27	4.81E-29	-3.48E+14	3.48E+14
ENSG00000139625	MAP3K12	0.40	-2.47	-1.31	5.40E-29	-3.37E+14	3.37E+14
ENSG00000160325	CACFD1	2.66	2.66	1.41	6.61E-29	3.27E+14	3.27E+14
ENSG00000214021	TTLL3	2.62	2.62	1.39	6.79E-29	3.18E+14	3.18E+14
ENSG00000236671	RP11-573I11.2	5.62	5.62	2.49	3.33E-28	3.08E+14	3.08E+14
ENSG00000172738	TMEM217	3.07	3.07	1.62	1.18E-28	2.83E+14	2.83E+14
ENSG00000232453	RP4-794H19.1	5.61	5.61	2.49	3.97E-28	2.81E+14	2.81E+14
ENSG00000103202	NME4	0.43	-2.35	-1.23	9.19E-29	-2.45E+14	2.45E+14
ENSG00000143799	PARP1	0.41	-2.43	-1.28	1.02E-28	-2.40E+14	2.40E+14
ENSG00000197555	SIPA1L1	2.51	2.51	1.33	1.31E-28	2.19E+14	2.19E+14
ENSG00000171346	KRT15	3.35	3.35	1.74	2.43E-28	2.15E+14	2.15E+14
ENSG00000027847	B4GALT7	0.40	-2.48	-1.31	1.35E-28	-2.14E+14	2.14E+14
ENSG00000118473	SGIP1	2.39	2.39	1.26	1.54E-28	1.93E+14	1.93E+14
ENSG00000213672	NCKIPSD	0.24	-4.23	-2.08	5.03E-28	-1.89E+14	1.89E+14
ENSG00000172927	MYEOV	3.68	3.68	1.88	4.33E-28	1.77E+14	1.77E+14
ENSG00000125458	NT5C	0.40	-2.50	-1.32	2.10E-28	-1.72E+14	1.72E+14
ENSG00000185522	C11orf35	5.81	5.81	2.54	1.28E-27	1.62E+14	1.62E+14
ENSG00000135469	COQ10A	0.31	-3.24	-1.70	4.24E-28	-1.57E+14	1.57E+14
ENSG00000235888	AF064858.8	6.13	6.13	2.62	1.66E-27	1.50E+14	1.50E+14
ENSG00000160781	PAQR6	5.67	5.67	2.50	1.54E-27	1.45E+14	1.45E+14
ENSG00000114554	PLXNA1	2.51	2.51	1.33	3.07E-28	1.43E+14	1.43E+14
ENSG00000076003	MCM6	0.43	-2.34	-1.23	2.86E-28	-1.38E+14	1.38E+14
ENSG00000244187	TMEM141	0.41	-2.42	-1.27	3.40E-28	-1.31E+14	1.31E+14
ENSG00000169499	PLEKHA2	0.34	-2.98	-1.57	7.23E-28	-1.11E+14	1.11E+14
ENSG00000167969	ECI1	0.37	-2.73	-1.45	6.34E-28	-1.08E+14	1.08E+14
ENSG00000008382	MPND	0.21	-4.80	-2.26	2.26E-27	-1.01E+14	1.01E+14
ENSG00000135074	ADAM19	2.30	2.30	1.20	5.34E-28	9.97E+13	9.97E+13
ENSG00000196329	GIMAP5	0.34	-2.96	-1.56	9.29E-28	-9.70E+13	9.70E+13
ENSG00000099203	TMED1	0.42	-2.41	-1.27	7.16E-28	-8.99E+13	8.99E+13
ENSG00000185928	PAGR1	0.40	-2.49	-1.32	7.97E-28	-8.82E+13	8.82E+13
ENSG00000221866	PLXNA4	0.35	-2.88	-1.53	1.16E-27	-8.43E+13	8.43E+13
ENSG00000166337	TAF10	0.44	-2.29	-1.20	8.49E-28	-7.86E+13	7.86E+13
ENSG00000138696	BMPR1B	0.29	-3.44	-1.78	2.06E-27	-7.57E+13	7.57E+13
ENSG00000023902	PLEKHO1	2.47	2.47	1.30	1.23E-27	7.02E+13	7.02E+13
ENSG00000115290	GRB14	0.40	-2.50	-1.32	2.00E-27	-5.58E+13	5.58E+13
ENSG00000158234	FAIM	0.38	-2.61	-1.38	2.26E-27	-5.49E+13	5.49E+13
ENSG00000130724	CHMP2A	0.44	-2.25	-1.17	1.81E-27	-5.30E+13	5.30E+13
ENSG00000163629	PTPN13	0.34	-2.91	-1.54	3.57E-27	-4.86E+13	4.86E+13
ENSG00000165283	STOML2	0.42	-2.35	-1.23	2.48E-27	-4.72E+13	4.72E+13
ENSG00000063438	AHRR	2.53	2.53	1.34	3.37E-27	4.36E+13	4.36E+13
ENSG00000184867	ARMCX2	0.43	-2.34	-1.23	2.97E-27	-4.29E+13	4.29E+13
ENSG00000234155	RP11-30P6.6	5.22	5.22	2.39	1.76E-26	3.94E+13	3.94E+13
ENSG00000214279	RP11-108K14.4	4.42	4.42	2.14	1.30E-26	3.87E+13	3.87E+13
ENSG00000106336	FBXO24	7.25	7.25	2.86	3.82E-26	3.71E+13	3.71E+13
ENSG00000114316	USP4	0.37	-2.70	-1.43	5.80E-27	-3.54E+13	3.54E+13
ENSG00000143545	RAB13	0.39	-2.59	-1.38	5.57E-27	-3.48E+13	3.48E+13
ENSG00000198892	SHISA4	0.35	-2.88	-1.53	6.94E-27	-3.46E+13	3.46E+13
ENSG00000111052	LIN7A	0.28	-3.56	-1.83	1.12E-26	-3.37E+13	3.37E+13
ENSG00000047617	ANO2	2.29	2.29	1.19	4.92E-27	3.26E+13	3.26E+13
ENSG00000258947	TUBB3	2.31	2.31	1.21	5.03E-27	3.25E+13	3.25E+13
ENSG00000132359	RAP1GAP2	3.36	3.36	1.75	1.38E-26	2.86E+13	2.86E+13
ENSG00000167536	DHRS13	0.31	-3.21	-1.68	1.60E-26	-2.53E+13	2.53E+13
ENSG00000100372	SLC25A17	0.43	-2.35	-1.23	8.86E-27	-2.49E+13	2.49E+13
ENSG00000140990	NDUFB10	0.45	-2.24	-1.17	9.08E-27	-2.35E+13	2.35E+13
ENSG00000183638	RP1L1	5.01	5.01	2.32	4.93E-26	2.26E+13	2.26E+13
ENSG00000099953	MMP11	2.66	2.66	1.41	1.69E-26	2.05E+13	2.05E+13
ENSG00000111674	ENO2	0.32	-3.14	-1.65	2.51E-26	-1.98E+13	1.98E+13
ENSG00000240990	HOXA11-AS	2.63	2.63	1.39	3.01E-26	1.51E+13	1.51E+13
ENSG00000185267	CDNF	6.23	6.23	2.64	1.80E-25	1.47E+13	1.47E+13
ENSG00000163071	SPATA18	2.28	2.28	1.19	2.52E-26	1.44E+13	1.44E+13
ENSG00000260337	RP11-386M24.6	5.63	5.63	2.49	1.58E-25	1.42E+13	1.42E+13
ENSG00000168237	GLYCTK	0.39	-2.56	-1.35	3.79E-26	-1.31E+13	1.31E+13
ENSG00000166803	KIAA0101	0.44	-2.29	-1.20	3.20E-26	-1.28E+13	1.28E+13
ENSG00000137872	SEMA6D	0.38	-2.64	-1.40	5.01E-26	-1.18E+13	1.18E+13
ENSG00000157227	MMP14	2.38	2.38	1.25	4.38E-26	1.14E+13	1.14E+13
ENSG00000186567	CEACAM19	3.29	3.29	1.72	8.74E-26	1.11E+13	1.11E+13
ENSG00000185022	MAFF	2.39	2.39	1.26	5.94E-26	9.81E+12	9.81E+12
ENSG00000106852	LHX6	2.37	2.37	1.25	6.02E-26	9.67E+12	9.67E+12
ENSG00000277972	CISD3	0.40	-2.51	-1.33	6.77E-26	-9.64E+12	9.64E+12
ENSG00000172031	EPHX4	2.38	2.38	1.25	6.17E-26	9.59E+12	9.59E+12
ENSG00000237870	AC073130.1	2.80	2.80	1.49	8.77E-26	9.46E+12	9.46E+12

ENSG00000112297	AIM1	2.33	2.33	1.22	7.03E-26	8.78E+12	8.78E+12
ENSG00000225377	RP5-1103G7.4	3.33	3.33	1.74	1.47E-25	8.68E+12	8.68E+12
ENSG00000131153	GINS2	0.46	-2.18	-1.13	7.16E-26	-8.15E+12	8.15E+12
ENSG00000166510	CCDC68	0.35	-2.86	-1.51	1.24E-25	-8.12E+12	8.12E+12
ENSG00000232533	AC093673.5	2.36	2.36	1.24	8.76E-26	7.98E+12	7.98E+12
ENSG00000169026	MFSD7	0.17	-5.80	-2.54	5.93E-25	-7.53E+12	7.53E+12
ENSG00000109771	LRP2BP	3.37	3.37	1.75	2.15E-25	7.26E+12	7.26E+12
ENSG00000260083	MIR4519	3.83	3.83	1.94	3.06E-25	6.91E+12	6.91E+12
ENSG00000130758	MAP3K10	2.54	2.54	1.35	1.59E-25	6.37E+12	6.37E+12
ENSG00000094804	CDC6	0.45	-2.23	-1.16	1.36E-25	-6.06E+12	6.06E+12
ENSG00000145911	N4BP3	2.29	2.29	1.19	1.48E-25	5.95E+12	5.95E+12
ENSG00000103507	BCKDK	0.41	-2.47	-1.30	1.84E-25	-5.75E+12	5.75E+12
ENSG00000156398	SFXN2	0.40	-2.47	-1.31	1.93E-25	-5.62E+12	5.62E+12
ENSG00000198336	MYL4	5.78	5.78	2.53	1.13E-24	5.43E+12	5.43E+12
ENSG00000114383	TUSC2	0.46	-2.20	-1.14	1.95E-25	-4.97E+12	4.97E+12
ENSG00000241170	RP11-147I3.1	6.20	6.20	2.63	2.05E-24	4.33E+12	4.33E+12
ENSG00000173267	SNCG	2.25	2.25	1.17	3.17E-25	3.99E+12	3.99E+12
ENSG00000173442	EHBP1L1	0.42	-2.37	-1.24	3.55E-25	-3.98E+12	3.98E+12
ENSG00000100938	GMPR2	0.45	-2.21	-1.14	3.11E-25	-3.95E+12	3.95E+12
ENSG00000100918	REC8	0.29	-3.40	-1.77	7.67E-25	-3.89E+12	3.89E+12
ENSG00000205038	PKHD1L1	0.17	-5.74	-2.52	2.80E-24	-3.43E+12	3.43E+12
ENSG00000075399	VPS9D1	2.52	2.52	1.34	5.61E-25	3.37E+12	3.37E+12
ENSG00000146859	TMEM140	0.34	-2.94	-1.56	8.32E-25	-3.22E+12	3.22E+12
ENSG00000100592	DAAM1	2.17	2.17	1.12	4.54E-25	3.22E+12	3.22E+12
ENSG00000169282	KCNAB1	0.37	-2.70	-1.43	7.76E-25	-3.06E+12	3.06E+12
ENSG00000161981	SNRNP25	0.44	-2.26	-1.18	5.67E-25	-3.00E+12	3.00E+12
ENSG00000146021	KLHL3	0.44	-2.27	-1.18	5.81E-25	-2.97E+12	2.97E+12
ENSG00000205502	C2CD4B	2.78	2.78	1.47	8.77E-25	2.97E+12	2.97E+12
ENSG00000260023	RP11-49C24.1	4.28	4.28	2.10	2.11E-24	2.94E+12	2.94E+12
ENSG00000102096	PIM2	0.31	-3.22	-1.69	1.22E-24	-2.92E+12	2.92E+12
ENSG00000166478	ZNF143	2.31	2.31	1.21	6.54E-25	2.86E+12	2.86E+12
ENSG00000111254	AKAP3	7.20	7.20	2.85	7.08E-24	2.70E+12	2.70E+12
ENSG00000141298	SSH2	2.26	2.26	1.18	7.61E-25	2.59E+12	2.59E+12
ENSG00000170271	C5orf4	0.36	-2.79	-1.48	1.32E-24	-2.42E+12	2.42E+12
ENSG00000108786	HSD17B1	4.57	4.57	2.19	3.60E-24	2.41E+12	2.41E+12
ENSG00000171202	TMEM126A	0.42	-2.37	-1.25	1.01E-24	-2.35E+12	2.35E+12
ENSG00000111341	MGP	0.42	-2.36	-1.24	1.09E-24	-2.26E+12	2.26E+12
ENSG00000006756	ARSD	2.41	2.41	1.27	1.18E-24	2.22E+12	2.22E+12
ENSG00000172530	BANP	2.50	2.50	1.32	1.42E-24	2.10E+12	2.10E+12
ENSG00000135318	NT5E	2.33	2.33	1.22	1.40E-24	1.96E+12	1.96E+12
ENSG00000149582	TMEM25	0.36	-2.77	-1.47	2.04E-24	-1.94E+12	1.94E+12
ENSG00000174276	ZNHIT2	0.30	-3.28	-1.71	3.03E-24	-1.88E+12	1.88E+12
ENSG00000196704	AMZ2	2.21	2.21	1.15	1.55E-24	1.78E+12	1.78E+12
ENSG00000214756	METTL12	0.30	-3.37	-1.75	3.62E-24	-1.77E+12	1.77E+12
ENSG00000253669	KB-1732A1.1	4.00	4.00	2.00	5.35E-24	1.73E+12	1.73E+12
ENSG00000116771	AGMAT	0.19	-5.14	-2.36	8.99E-24	-1.71E+12	1.71E+12
ENSG00000129038	LOXL1	3.20	3.20	1.68	3.54E-24	1.70E+12	1.70E+12
ENSG00000133466	C1QTNF6	2.36	2.36	1.24	2.14E-24	1.61E+12	1.61E+12
ENSG00000277775	HIST1H3F	0.42	-2.36	-1.24	2.18E-24	-1.60E+12	1.60E+12
ENSG00000166997	CNPY4	0.46	-2.17	-1.12	2.01E-24	-1.53E+12	1.53E+12
ENSG00000170011	MYRIP	0.45	-2.25	-1.17	2.37E-24	-1.46E+12	1.46E+12
ENSG00000171621	SPSB1	2.13	2.13	1.09	2.22E-24	1.43E+12	1.43E+12
ENSG00000169750	RAC3	0.46	-2.19	-1.13	2.63E-24	-1.35E+12	1.35E+12
ENSG00000107719	PALD1	2.32	2.32	1.22	2.99E-24	1.34E+12	1.34E+12
ENSG00000250091	DNAH10OS	6.01	6.01	2.59	2.24E-23	1.27E+12	1.27E+12
ENSG00000168569	TMEM223	0.39	-2.58	-1.37	4.25E-24	-1.25E+12	1.25E+12
ENSG00000277734	TRAC	5.89	5.89	2.56	2.28E-23	1.23E+12	1.23E+12
ENSG00000267317	CTB-25B13.12	4.48	4.48	2.16	1.37E-23	1.21E+12	1.21E+12
ENSG00000143554	SLC27A3	0.39	-2.54	-1.34	4.63E-24	-1.18E+12	1.18E+12
ENSG00000234883	MIR155HG	0.31	-3.20	-1.68	7.85E-24	-1.14E+12	1.14E+12
ENSG00000187024	PTRH1	0.39	-2.53	-1.34	5.05E-24	-1.13E+12	1.13E+12
ENSG00000131374	TBC1D5	2.24	2.24	1.16	3.97E-24	1.12E+12	1.12E+12
ENSG00000205403	CFI	0.44	-2.28	-1.19	4.40E-24	-1.09E+12	1.09E+12
ENSG00000196139	AKR1C3	0.41	-2.44	-1.28	5.19E-24	-1.07E+12	1.07E+12
ENSG00000136877	FPGS	0.46	-2.19	-1.13	4.44E-24	-1.04E+12	1.04E+12
ENSG00000065621	GSTO2	5.57	5.57	2.48	2.89E-23	1.04E+12	1.04E+12
ENSG00000197375	SLC22A5	2.52	2.52	1.33	5.93E-24	1.04E+12	1.04E+12
ENSG00000152926	ZNF117	3.31	3.31	1.73	1.05E-23	1.02E+12	1.02E+12
ENSG00000167705	RILP	0.33	-2.99	-1.58	8.68E-24	-1.02E+12	1.02E+12
ENSG00000230266	XXYLT1-AS2	2.36	2.36	1.24	6.22E-24	9.48E+11	9.48E+11
ENSG00000069702	TGFBR3	0.44	-2.27	-1.19	6.25E-24	-9.10E+11	9.10E+11
ENSG00000072609	CHFR	0.46	-2.19	-1.13	5.92E-24	-9.00E+11	9.00E+11
ENSG00000178695	KCTD12	0.44	-2.26	-1.18	6.39E-24	-8.94E+11	8.94E+11
ENSG00000116106	EPHA4	2.20	2.20	1.14	6.10E-24	8.91E+11	8.91E+11
ENSG00000177483	RBM44	3.62	3.62	1.86	1.84E-23	8.43E+11	8.43E+11
ENSG00000003400	CASP10	0.47	-2.13	-1.09	6.59E-24	-8.28E+11	8.28E+11
ENSG00000126878	AIF1L	0.36	-2.78	-1.47	1.18E-23	-8.08E+11	8.08E+11
ENSG00000274750	HIST1H3E	0.41	-2.42	-1.28	9.94E-24	-7.69E+11	7.69E+11
ENSG00000109458	GAB1	2.14	2.14	1.09	8.61E-24	7.28E+11	7.28E+11

ENSG00000215845	TSTD1	0.33	-3.03	-1.60	1.80E-23	-7.14E+11	7.14E+11
ENSG00000137877	SPTBN5	2.29	2.29	1.19	1.08E-23	6.97E+11	6.97E+11
ENSG00000160179	ABCG1	2.12	2.12	1.08	9.33E-24	6.94E+11	6.94E+11
ENSG00000177663	IL17RA	2.11	2.11	1.08	1.03E-23	6.57E+11	6.57E+11
ENSG00000135951	TSGA10	4.94	4.94	2.31	5.83E-23	6.47E+11	6.47E+11
ENSG00000156253	RWDD2B	0.45	-2.24	-1.17	1.22E-23	-6.42E+11	6.42E+11
ENSG00000174365	SNHG11	2.57	2.57	1.36	1.76E-23	6.14E+11	6.14E+11
ENSG00000148291	SURF2	0.35	-2.89	-1.53	2.24E-23	-6.11E+11	6.11E+11
ENSG00000185813	PCYT2	0.46	-2.17	-1.12	1.49E-23	-5.63E+11	5.63E+11
ENSG00000186174	BCL9L	2.61	2.61	1.38	2.33E-23	5.41E+11	5.41E+11
ENSG00000156453	PCDH1	2.40	2.40	1.26	2.00E-23	5.37E+11	5.37E+11
ENSG00000100350	FOXRED2	0.36	-2.81	-1.49	2.79E-23	-5.31E+11	5.31E+11
ENSG00000185507	IRF7	0.28	-3.53	-1.82	5.08E-23	-4.96E+11	4.96E+11
ENSG00000105655	ISYNA1	0.33	-3.01	-1.59	3.68E-23	-4.95E+11	4.95E+11
ENSG00000109501	WFS1	2.60	2.60	1.38	2.85E-23	4.88E+11	4.88E+11
ENSG00000175745	NR2F1	0.40	-2.51	-1.33	3.04E-23	-4.55E+11	4.55E+11
ENSG00000160753	RUSC1	0.45	-2.23	-1.16	3.09E-23	-4.01E+11	4.01E+11
ENSG00000163590	PPM1L	2.71	2.71	1.44	4.66E-23	3.97E+11	3.97E+11
ENSG00000007384	RHBDF1	2.26	2.26	1.17	3.41E-23	3.87E+11	3.87E+11
ENSG00000235750	KIAA0040	2.25	2.25	1.17	3.51E-23	3.79E+11	3.79E+11
ENSG00000078487	ZCWPW1	4.15	4.15	2.05	1.39E-22	3.52E+11	3.52E+11
ENSG00000179532	DNHD1	3.88	3.88	1.96	1.26E-22	3.45E+11	3.45E+11
ENSG00000117877	CD3EAP	0.46	-2.16	-1.11	4.40E-23	-3.25E+11	3.25E+11
ENSG00000130303	BST2	0.44	-2.27	-1.18	4.93E-23	-3.23E+11	3.23E+11
ENSG00000106804	C5	2.62	2.62	1.39	6.61E-23	3.22E+11	3.22E+11
ENSG00000126803	HSPA2	0.35	-2.82	-1.50	7.69E-23	-3.22E+11	3.22E+11
ENSG00000175274	TP53I11	2.10	2.10	1.07	4.29E-23	3.20E+11	3.20E+11
ENSG00000161912	ADCY10P1	3.97	3.97	1.99	1.54E-22	3.20E+11	3.20E+11
ENSG00000274618	HIST1H4F	0.43	-2.34	-1.23	5.83E-23	-3.06E+11	3.06E+11
ENSG00000092330	TINF2	0.48	-2.10	-1.07	4.80E-23	-3.03E+11	3.03E+11
ENSG00000147642	SYBU	0.21	-4.71	-2.24	2.64E-22	-2.90E+11	2.90E+11
ENSG00000132763	MMACHC	0.35	-2.84	-1.51	9.60E-23	-2.90E+11	2.90E+11
ENSG00000213888	AC005003.1	3.57	3.57	1.84	1.70E-22	2.74E+11	2.74E+11
ENSG00000121005	CRISPLD1	0.43	-2.33	-1.22	7.52E-23	-2.68E+11	2.68E+11
ENSG00000144645	OSBPL10	2.32	2.32	1.21	8.26E-23	2.55E+11	2.55E+11
ENSG00000163689	C3orf67	2.32	2.32	1.22	8.74E-23	2.48E+11	2.48E+11
ENSG00000168961	LGALS9	0.36	-2.82	-1.49	1.29E-22	-2.47E+11	2.47E+11
ENSG00000085063	CD59	0.46	-2.19	-1.13	8.35E-23	-2.40E+11	2.40E+11
ENSG00000173334	TRIB1	2.23	2.23	1.16	8.87E-23	2.37E+11	2.37E+11
ENSG00000029534	ANK1	3.87	3.87	1.95	3.12E-22	2.19E+11	2.19E+11
	RP11-521C20.4	4.13	4.13	2.04	3.70E-22	2.14E+11	2.14E+11
ENSG00000239521	GATS	2.80	2.80	1.48	1.97E-22	1.99E+11	1.99E+11
ENSG00000156928	MALSU1	0.39	-2.57	-1.36	1.71E-22	-1.97E+11	1.97E+11
ENSG00000165898	ISCA2	0.43	-2.34	-1.23	1.69E-22	-1.80E+11	1.80E+11
ENSG00000073756	PTGS2	0.45	-2.24	-1.17	1.79E-22	-1.68E+11	1.68E+11
ENSG00000275221	HIST1H2AK	0.47	-2.12	-1.08	1.61E-22	-1.67E+11	1.67E+11
ENSG00000107185	RGP1	2.18	2.18	1.12	1.85E-22	1.60E+11	1.60E+11
ENSG00000159423	ALDH4A1	0.38	-2.65	-1.41	2.77E-22	-1.59E+11	1.59E+11
ENSG00000221949	C12orf61	3.57	3.57	1.84	5.12E-22	1.58E+11	1.58E+11
ENSG00000144749	LRIG1	2.36	2.36	1.24	2.26E-22	1.57E+11	1.57E+11
ENSG00000114698	PLSCR4	0.47	-2.15	-1.10	1.89E-22	-1.56E+11	1.56E+11
ENSG00000113360	DROSHA	2.25	2.25	1.17	2.29E-22	1.49E+11	1.49E+11
ENSG00000198286	CARD11	2.18	2.18	1.13	2.21E-22	1.47E+11	1.47E+11
ENSG00000263528	IKBKE	0.43	-2.33	-1.22	2.67E-22	-1.43E+11	1.43E+11
ENSG00000241945	PWP2	0.49	-2.05	-1.04	2.24E-22	-1.37E+11	1.37E+11
ENSG00000242125	SNHG3	0.46	-2.16	-1.11	2.54E-22	-1.36E+11	1.36E+11
ENSG00000132141	CCT6B	4.85	4.85	2.28	1.32E-21	1.33E+11	1.33E+11
ENSG00000103647	CORO2B	2.91	2.91	1.54	5.02E-22	1.30E+11	1.30E+11
ENSG00000166897	ELFN2	3.36	3.36	1.75	6.90E-22	1.28E+11	1.28E+11
ENSG00000173531	MST1	3.50	3.50	1.81	8.50E-22	1.20E+11	1.20E+11
ENSG00000109046	WSB1	0.45	-2.21	-1.15	3.50E-22	-1.18E+11	1.18E+11
ENSG00000110200	ANAPC15	2.08	2.08	1.06	3.29E-22	1.15E+11	1.15E+11
ENSG00000116574	RHOU	0.21	-4.65	-2.22	1.68E-21	-1.13E+11	1.13E+11
ENSG00000131386	GALNTL2	0.20	-5.12	-2.36	2.13E-21	-1.11E+11	1.11E+11
ENSG00000173821	RNF213	2.76	2.76	1.47	6.96E-22	1.05E+11	1.05E+11
ENSG00000135069	PSAT1	0.47	-2.11	-1.08	5.32E-22	-9.15E+10	9.15E+10
ENSG00000161179	YDJC	0.42	-2.40	-1.26	8.16E-22	-8.41E+10	8.41E+10
ENSG00000119986	AVPI1	0.26	-3.88	-1.96	2.24E-21	-8.20E+10	8.20E+10
ENSG00000177542	SLC25A22	0.39	-2.55	-1.35	9.78E-22	-8.15E+10	8.15E+10
ENSG00000150764	DIXDC1	0.46	-2.16	-1.11	7.28E-22	-8.01E+10	8.01E+10
ENSG00000236263	RP11-263K19.6	4.47	4.47	2.16	3.14E-21	7.97E+10	7.97E+10
ENSG00000258976	CTD-2207P18.2	4.47	4.47	2.16	3.21E-21	7.88E+10	7.88E+10
ENSG00000167680	SEMA6B	2.25	2.25	1.17	8.37E-22	7.78E+10	7.78E+10
ENSG00000169926	KLF13	2.12	2.12	1.08	8.41E-22	7.31E+10	7.31E+10
ENSG00000136244	IL6	0.40	-2.51	-1.33	1.19E-21	-7.28E+10	7.28E+10
ENSG00000171720	HDAC3	0.47	-2.13	-1.09	9.82E-22	-6.80E+10	6.80E+10
ENSG00000113621	TXNDC15	0.46	-2.16	-1.11	1.14E-21	-6.38E+10	6.38E+10
ENSG00000048052	HDAC9	4.28	4.28	2.10	5.58E-21	5.73E+10	5.73E+10
ENSG00000144824	PHLDB2	2.26	2.26	1.17	1.73E-21	5.43E+10	5.43E+10

ENSG00000101412	E2F1	0.48	-2.08	-1.06	1.48E-21	-5.42E+10	5.42E+10
ENSG00000183621	ZNF438	2.03	2.03	1.02	1.49E-21	5.26E+10	5.26E+10
ENSG00000120051	CCDC147	4.68	4.68	2.23	8.09E-21	5.20E+10	5.20E+10
ENSG00000230438	RP11-420G6.4	2.54	2.54	1.34	2.44E-21	5.14E+10	5.14E+10
ENSG00000002330	BAD	0.46	-2.17	-1.11	1.93E-21	-4.93E+10	4.93E+10
ENSG00000176978	DPP7	0.48	-2.09	-1.06	1.89E-21	-4.80E+10	4.80E+10
ENSG00000167483	FAM129C	3.62	3.62	1.85	5.85E-21	4.73E+10	4.73E+10
ENSG00000006837	CDKL3	3.02	3.02	1.59	4.66E-21	4.42E+10	4.42E+10
ENSG00000184470	TXNRD2	2.10	2.10	1.07	2.29E-21	4.38E+10	4.38E+10
ENSG00000130479	MAP1S	2.27	2.27	1.18	3.24E-21	3.98E+10	3.98E+10
ENSG00000182511	FES	0.49	-2.05	-1.04	2.93E-21	-3.79E+10	3.79E+10
ENSG00000221926	TRIM16	2.14	2.14	1.10	3.36E-21	3.69E+10	3.69E+10
ENSG00000100445	SDR39U1	0.39	-2.55	-1.35	4.92E-21	-3.64E+10	3.64E+10
ENSG00000164647	STEAP1	0.43	-2.35	-1.23	4.21E-21	-3.62E+10	3.62E+10
ENSG00000138028	CGREF1	0.27	-3.75	-1.91	1.33E-20	-3.25E+10	3.25E+10
ENSG00000276903	HIST1H2AL	0.45	-2.20	-1.14	4.75E-21	-3.20E+10	3.20E+10
	CTD-2611K5.6	4.46	4.46	2.16	1.97E-20	3.18E+10	3.18E+10
ENSG00000132622	HSPA12B	2.03	2.03	1.02	4.26E-21	3.11E+10	3.11E+10
ENSG00000163170	BOLA3	0.48	-2.10	-1.07	4.60E-21	-3.09E+10	3.09E+10
ENSG00000180834	MAP6D1	0.23	-4.34	-2.12	2.02E-20	-3.05E+10	3.05E+10
ENSG00000172748	ZNF596	2.73	2.73	1.45	8.92E-21	2.89E+10	2.89E+10
ENSG00000120278	PLEKHG1	2.68	2.68	1.42	8.72E-21	2.87E+10	2.87E+10
ENSG00000198517	MAFK	2.17	2.17	1.12	5.74E-21	2.86E+10	2.86E+10
ENSG00000171345	KRT19	2.14	2.14	1.09	5.64E-21	2.84E+10	2.84E+10
ENSG00000108176	DNAJC12	0.28	-3.59	-1.84	1.64E-20	-2.80E+10	2.80E+10
ENSG00000005108	THSD7A	0.45	-2.21	-1.15	6.37E-21	-2.77E+10	2.77E+10
ENSG00000136783	NIPSNAP3A	0.49	-2.04	-1.03	5.65E-21	-2.72E+10	2.72E+10
ENSG00000153208	MERTK	2.17	2.17	1.12	6.37E-21	2.72E+10	2.72E+10
ENSG00000079482	OPHN1	0.47	-2.13	-1.09	6.70E-21	-2.60E+10	2.60E+10
ENSG00000126522	ASL	0.49	-2.06	-1.04	6.40E-21	-2.57E+10	2.57E+10
ENSG00000186815	TPCN1	2.34	2.34	1.23	1.07E-20	2.26E+10	2.26E+10
ENSG00000114023	FAM162A	0.48	-2.09	-1.07	9.03E-21	-2.20E+10	2.20E+10
ENSG00000183624	C3orf37	0.47	-2.12	-1.09	9.42E-21	-2.19E+10	2.19E+10
	RP11-182J1.9	5.14	5.14	2.36	6.17E-20	2.07E+10	2.07E+10
ENSG00000174130	TLR6	2.48	2.48	1.31	1.57E-20	1.98E+10	1.98E+10
ENSG00000103876	FAH	0.47	-2.12	-1.09	1.27E-20	-1.88E+10	1.88E+10
ENSG00000131016	AKAP12	2.18	2.18	1.12	1.40E-20	1.84E+10	1.84E+10
ENSG00000139055	ERP27	4.10	4.10	2.04	5.11E-20	1.81E+10	1.81E+10
ENSG00000176422	SPRYD4	0.42	-2.38	-1.25	1.75E-20	-1.80E+10	1.80E+10
ENSG00000145703	IQGAP2	2.92	2.92	1.55	2.92E-20	1.71E+10	1.71E+10
ENSG00000104738	MCM4	0.48	-2.09	-1.06	1.69E-20	-1.61E+10	1.61E+10
ENSG00000007908	SELE	5.55	5.55	2.47	1.32E-19	1.53E+10	1.53E+10
ENSG00000145949	MYLK4	3.74	3.74	1.90	6.05E-20	1.52E+10	1.52E+10
ENSG00000185386	MAPK11	0.45	-2.24	-1.16	2.25E-20	-1.49E+10	1.49E+10
ENSG00000017483	SLC38A5	0.49	-2.06	-1.04	1.94E-20	-1.48E+10	1.48E+10
ENSG00000171552	BCL2L1	2.09	2.09	1.07	2.05E-20	1.46E+10	1.46E+10
ENSG00000111863	ADTRP	2.01	2.01	1.01	1.94E-20	1.44E+10	1.44E+10
ENSG00000196465	MYL6B	0.48	-2.10	-1.07	2.17E-20	-1.43E+10	1.43E+10
ENSG00000163803	PLB1	3.86	3.86	1.95	8.26E-20	1.34E+10	1.34E+10
ENSG00000099889	ARVCF	1.98	1.98	0.99	2.29E-20	1.31E+10	1.31E+10
ENSG00000178878	APOLD1	1.99	1.99	1.00	2.60E-20	1.24E+10	1.24E+10
ENSG00000128311	TST	2.03	2.03	1.02	2.73E-20	1.23E+10	1.23E+10
ENSG00000197321	SVIL	2.13	2.13	1.09	3.02E-20	1.23E+10	1.23E+10
ENSG00000100196	KDELR3	0.45	-2.23	-1.15	3.29E-20	-1.23E+10	1.23E+10
ENSG00000170264	FAM161A	0.29	-3.42	-1.78	8.04E-20	-1.21E+10	1.21E+10
ENSG00000128052	KDR	2.19	2.19	1.13	3.61E-20	1.15E+10	1.15E+10
ENSG00000188157	AGRN	2.06	2.06	1.04	3.24E-20	1.14E+10	1.14E+10
ENSG00000162496	DHRS3	1.98	1.98	0.99	3.06E-20	1.13E+10	1.13E+10
ENSG00000186318	BACE1	2.24	2.24	1.16	4.22E-20	1.09E+10	1.09E+10
ENSG00000233818	AP000695.4	2.01	2.01	1.01	3.47E-20	1.08E+10	1.08E+10
ENSG00000148057	IDNK	2.37	2.37	1.25	5.45E-20	1.02E+10	1.02E+10
ENSG00000070785	EIF2B3	0.49	-2.05	-1.04	4.11E-20	-1.01E+10	1.01E+10
ENSG00000092010	PSME1	0.49	-2.04	-1.03	4.46E-20	-9.66E+09	9.66E+09
ENSG00000187134	AKR1C1	0.26	-3.85	-1.95	1.75E-19	-9.21E+09	9.21E+09
ENSG00000130997	POLN	3.16	3.16	1.66	1.31E-19	8.74E+09	8.74E+09
ENSG00000179387	ELMOD2	2.54	2.54	1.35	8.58E-20	8.68E+09	8.68E+09
ENSG00000189134	NKAPL	3.94	3.94	1.98	2.15E-19	8.49E+09	8.49E+09
ENSG00000124575	HIST1H1D	0.48	-2.09	-1.06	6.27E-20	-8.35E+09	8.35E+09
ENSG00000099899	TRMT2A	0.50	-2.00	-1.00	5.84E-20	-8.29E+09	8.29E+09
ENSG00000278615	C11orf48	0.49	-2.02	-1.02	5.99E-20	-8.27E+09	8.27E+09
ENSG00000229320	KRT8P12	3.64	3.64	1.86	2.04E-19	8.06E+09	8.06E+09
ENSG00000136305	CIDEB	0.35	-2.82	-1.50	1.36E-19	-7.65E+09	7.65E+09
ENSG00000242498	C15orf38	0.43	-2.35	-1.23	9.50E-20	-7.63E+09	7.63E+09
ENSG00000103152	MPG	2.04	2.04	1.03	7.61E-20	7.41E+09	7.41E+09
ENSG00000156273	BACH1	2.23	2.23	1.16	9.30E-20	7.32E+09	7.32E+09
ENSG00000081041	CXCL2	0.25	-4.02	-2.01	3.10E-19	-7.21E+09	7.21E+09
ENSG00000061938	TNK2	2.06	2.06	1.04	8.50E-20	7.07E+09	7.07E+09
ENSG00000112039	FANCE	0.46	-2.16	-1.11	9.46E-20	-7.01E+09	7.01E+09
ENSG00000130584	ZBTB46	2.40	2.40	1.26	1.25E-19	6.79E+09	6.79E+09

ENSG00000247679	RP11-1277A3.1	3.39	3.39	1.76	2.79E-19	6.41E+09	6.41E+09
ENSG00000263753	LINC00667	1.99	1.99	0.99	9.96E-20	6.31E+09	6.31E+09
ENSG00000184500	PROS1	0.47	-2.14	-1.10	1.20E-19	-6.19E+09	6.19E+09
ENSG00000186603	HPDL	0.34	-2.91	-1.54	2.21E-19	-6.18E+09	6.18E+09
ENSG00000134109	EDEM1	2.19	2.19	1.13	1.32E-19	6.03E+09	6.03E+09
ENSG00000055813	CCDC85A	0.50	-1.98	-0.99	1.19E-19	-5.74E+09	5.74E+09
ENSG00000181218	HIST3H2A	0.39	-2.57	-1.36	2.03E-19	-5.70E+09	5.70E+09
ENSG00000056972	TRAF3IP2	1.99	1.99	0.99	1.22E-19	5.68E+09	5.68E+09
ENSG00000092470	WDR76	0.50	-2.01	-1.00	1.27E-19	-5.62E+09	5.62E+09
ENSG00000111339	ART4	0.34	-2.95	-1.56	3.18E-19	-5.23E+09	5.23E+09
ENSG00000110002	VWA5A	2.09	2.09	1.06	1.63E-19	5.17E+09	5.17E+09
ENSG00000148824	MTG1	0.48	-2.06	-1.04	1.62E-19	-5.12E+09	5.12E+09
ENSG00000189056	RELN	3.96	3.96	1.99	6.14E-19	5.06E+09	5.06E+09
ENSG00000180353	HCLS1	0.46	-2.18	-1.13	1.96E-19	-4.93E+09	4.93E+09
ENSG00000187720	THSD4	1.98	1.98	0.98	1.66E-19	4.86E+09	4.86E+09
ENSG00000139263	LRIG3	0.48	-2.07	-1.05	1.89E-19	-4.75E+09	4.75E+09
ENSG00000127838	PNKD	0.50	-1.98	-0.99	1.85E-19	-4.61E+09	4.61E+09
ENSG00000118985	ELL2	0.50	-1.98	-0.99	1.86E-19	-4.60E+09	4.60E+09
ENSG00000185504	C17orf70	0.51	-1.97	-0.98	1.90E-19	-4.53E+09	4.53E+09
ENSG00000176658	MYO1D	2.03	2.03	1.02	2.11E-19	4.42E+09	4.42E+09
	RP11-492E3.1	1.97	1.97	0.98	2.13E-19	4.28E+09	4.28E+09
ENSG00000099622	CIRBP	0.49	-2.02	-1.02	2.35E-19	-4.17E+09	4.17E+09
ENSG00000162139	NEU3	0.40	-2.53	-1.34	4.24E-19	-3.88E+09	3.88E+09
ENSG00000185838	GNB1L	2.40	2.40	1.27	3.92E-19	3.84E+09	3.84E+09
ENSG00000111445	RFC5	0.50	-1.98	-0.99	2.84E-19	-3.72E+09	3.72E+09
ENSG00000122641	INHBA	0.47	-2.13	-1.09	3.28E-19	-3.71E+09	3.71E+09
ENSG00000141424	SLC39A6	1.94	1.94	0.95	3.04E-19	3.51E+09	3.51E+09
ENSG00000124762	CDKN1A	2.03	2.03	1.02	3.47E-19	3.45E+09	3.45E+09
ENSG00000173918	C1QTNF1	3.01	3.01	1.59	9.89E-19	3.03E+09	3.03E+09
ENSG00000187123	LYPD6	0.41	-2.41	-1.27	6.64E-19	-2.96E+09	2.96E+09
ENSG00000112981	NME5	0.24	-4.23	-2.08	2.05E-18	-2.95E+09	2.95E+09
ENSG00000160404	TOR2A	0.42	-2.39	-1.26	6.60E-19	-2.94E+09	2.94E+09
ENSG00000117682	DHDDS	0.50	-2.00	-1.00	4.93E-19	-2.84E+09	2.84E+09
ENSG00000154258	ABCA9	2.38	2.38	1.25	7.04E-19	2.84E+09	2.84E+09
ENSG00000278535	DHRS11	0.35	-2.89	-1.53	1.07E-18	-2.80E+09	2.80E+09
ENSG00000160447	PKN3	0.51	-1.95	-0.96	5.01E-19	-2.76E+09	2.76E+09
ENSG00000249459	ZNF286B	2.89	2.89	1.53	1.14E-18	2.71E+09	2.71E+09
ENSG00000140941	MAP1LC3B	2.04	2.04	1.03	6.38E-19	2.55E+09	2.55E+09
ENSG00000248429	RP11-597D13.9	2.35	2.35	1.23	8.64E-19	2.53E+09	2.53E+09
ENSG00000082512	TRAF5	0.48	-2.07	-1.05	6.75E-19	-2.52E+09	2.52E+09
ENSG00000170365	SMAD1	2.71	2.71	1.44	1.19E-18	2.48E+09	2.48E+09
ENSG00000261716	RP11-196G18.22	2.40	2.40	1.26	9.62E-19	2.44E+09	2.44E+09
ENSG00000171148	TADA3	1.96	1.96	0.97	6.87E-19	2.37E+09	2.37E+09
ENSG00000162522	KIAA1522	2.43	2.43	1.28	1.18E-18	2.24E+09	2.24E+09
ENSG00000197291	RAMP2-AS1	2.61	2.61	1.38	1.37E-18	2.23E+09	2.23E+09
ENSG00000139531	SUOX	0.48	-2.07	-1.05	9.10E-19	-2.16E+09	2.16E+09
ENSG00000139289	PHLDA1	2.11	2.11	1.08	9.51E-19	2.16E+09	2.16E+09
ENSG00000162735	PEX19	0.52	-1.92	-0.94	8.35E-19	-2.10E+09	2.10E+09
ENSG00000135643	KCNMB4	0.31	-3.20	-1.68	2.49E-18	-2.03E+09	2.03E+09
ENSG00000163795	ZNF513	0.44	-2.26	-1.18	1.25E-18	-2.02E+09	2.02E+09
ENSG00000229855	CTC-546K23.1	0.25	-4.00	-2.00	4.53E-18	-1.88E+09	1.88E+09
ENSG00000164104	HMGB2	0.49	-2.02	-1.02	1.20E-18	-1.85E+09	1.85E+09
ENSG00000101194	SLC17A9	0.46	-2.16	-1.11	1.37E-18	-1.85E+09	1.85E+09
ENSG00000073737	DHRS9	0.30	-3.29	-1.72	3.60E-18	-1.73E+09	1.73E+09
ENSG00000095370	SH2D3C	1.97	1.97	0.98	1.33E-18	1.71E+09	1.71E+09
ENSG00000124615	MOCS1	0.45	-2.20	-1.14	1.75E-18	-1.67E+09	1.67E+09
ENSG00000064393	HIPK2	2.05	2.05	1.04	1.52E-18	1.67E+09	1.67E+09
ENSG00000013619	MAMLD1	3.43	3.43	1.78	4.54E-18	1.61E+09	1.61E+09
ENSG00000102897	LYRM1	1.97	1.97	0.98	1.52E-18	1.60E+09	1.60E+09
ENSG00000178922	HYI	0.51	-1.96	-0.97	1.53E-18	-1.59E+09	1.59E+09
ENSG00000197077	KIAA1671	2.06	2.06	1.04	1.74E-18	1.57E+09	1.57E+09
ENSG00000142733	MAP3K6	0.43	-2.32	-1.22	2.27E-18	-1.54E+09	1.54E+09
ENSG00000132530	XAF1	0.33	-3.06	-1.61	4.04E-18	-1.52E+09	1.52E+09
ENSG00000073734	ABCB11	3.87	3.87	1.95	6.63E-18	1.50E+09	1.50E+09
ENSG00000233452	STXBP5-AS1	2.77	2.77	1.47	3.66E-18	1.45E+09	1.45E+09
ENSG00000261654	RP11-96K19.4	3.62	3.62	1.85	6.62E-18	1.40E+09	1.40E+09
ENSG00000159714	ZDHHC1	2.32	2.32	1.21	2.73E-18	1.40E+09	1.40E+09
ENSG00000235505	RP11-693N9.2	0.43	-2.35	-1.23	2.85E-18	-1.39E+09	1.39E+09
ENSG00000126249	PDCD2L	0.42	-2.39	-1.26	2.98E-18	-1.38E+09	1.38E+09
ENSG00000091844	RGS17	2.43	2.43	1.28	3.21E-18	1.36E+09	1.36E+09
ENSG00000083544	TDRD3	2.10	2.10	1.07	2.49E-18	1.33E+09	1.33E+09
ENSG00000187051	RPS19BP1	0.51	-1.95	-0.96	2.20E-18	-1.31E+09	1.31E+09
ENSG00000134324	LPIN1	2.08	2.08	1.06	2.68E-18	1.27E+09	1.27E+09
ENSG00000250510	GPR162	0.40	-2.50	-1.32	3.89E-18	-1.27E+09	1.27E+09
ENSG00000174370	C11orf45	3.34	3.34	1.74	7.29E-18	1.24E+09	1.24E+09
ENSG00000114450	GNB4	2.01	2.01	1.01	2.64E-18	1.23E+09	1.23E+09
ENSG00000161980	POLR3K	0.43	-2.31	-1.20	3.57E-18	-1.22E+09	1.22E+09
ENSG00000243232	PCDHAC2	0.27	-3.67	-1.88	9.07E-18	-1.22E+09	1.22E+09
ENSG00000186111	PIP5K1C	2.02	2.02	1.02	2.94E-18	1.18E+09	1.18E+09

ENSG00000136378	ADAMTS7	1.91	1.91	0.94	2.67E-18	1.17E+09	1.17E+09
ENSG00000162413	KLHL21	1.94	1.94	0.96	2.77E-18	1.16E+09	1.16E+09
ENSG00000183048	SLC25A10	0.40	-2.47	-1.30	4.65E-18	-1.14E+09	1.14E+09
ENSG00000100441	KHNYN	2.00	2.00	1.00	3.05E-18	1.14E+09	1.14E+09
ENSG00000109466	KLHL2	2.00	2.00	1.00	3.26E-18	1.10E+09	1.10E+09
ENSG00000151388	ADAMTS12	0.42	-2.40	-1.26	5.09E-18	-1.06E+09	1.06E+09
ENSG00000106415	GLCCI1	2.44	2.44	1.28	5.56E-18	1.03E+09	1.03E+09
ENSG00000132016	C19orf57	3.68	3.68	1.88	1.30E-17	1.02E+09	1.02E+09
ENSG00000160867	FGFR4	3.88	3.88	1.96	1.57E-17	9.81E+08	9.81E+08
ENSG00000138311	ZNF365	3.01	3.01	1.59	1.02E-17	9.45E+08	9.45E+08
ENSG00000205562	RP11-497E19.1	2.53	2.53	1.34	7.35E-18	9.32E+08	9.32E+08
ENSG00000166402	TUB	0.46	-2.19	-1.13	5.57E-18	-9.28E+08	9.28E+08
ENSG00000115525	ST3GAL5	1.97	1.97	0.98	4.87E-18	8.92E+08	8.92E+08
ENSG00000116455	WDR77	0.49	-2.04	-1.03	5.22E-18	-8.92E+08	8.92E+08
ENSG00000175352	NRIP3	0.52	-1.92	-0.94	5.07E-18	-8.50E+08	8.50E+08
ENSG00000132561	MATN2	0.21	-4.71	-2.24	3.20E-17	-8.33E+08	8.33E+08
ENSG00000090339	ICAM1	3.60	3.60	1.85	2.07E-17	7.91E+08	7.91E+08
ENSG00000072786	STK10	1.97	1.97	0.97	6.18E-18	7.91E+08	7.91E+08
ENSG00000163703	CRELD1	0.49	-2.06	-1.04	6.79E-18	-7.89E+08	7.89E+08
ENSG00000105617	LENG1	0.42	-2.40	-1.26	9.99E-18	-7.59E+08	7.59E+08
ENSG00000099219	ERMP1	0.47	-2.11	-1.07	8.21E-18	-7.35E+08	7.35E+08
ENSG00000099998	GGT5	0.44	-2.28	-1.19	9.82E-18	-7.26E+08	7.26E+08
ENSG00000114026	OGG1	0.49	-2.06	-1.04	8.35E-18	-7.13E+08	7.13E+08
ENSG00000129596	CDO1	0.22	-4.45	-2.15	3.90E-17	-7.12E+08	7.12E+08
ENSG00000122644	ARL4A	0.47	-2.11	-1.08	9.05E-18	-7.02E+08	7.02E+08
ENSG00000100100	PIK3IP1	0.26	-3.83	-1.94	3.11E-17	-6.87E+08	6.87E+08
ENSG00000181513	ACBD4	0.34	-2.94	-1.56	1.92E-17	-6.71E+08	6.71E+08
ENSG00000175183	CSRP2	0.44	-2.28	-1.19	1.17E-17	-6.67E+08	6.67E+08
ENSG00000114054	PCCB	0.52	-1.91	-0.94	8.44E-18	-6.59E+08	6.59E+08
ENSG00000114021	NIT2	0.52	-1.92	-0.94	8.98E-18	-6.42E+08	6.42E+08
ENSG00000188938	FAM120AOS	0.53	-1.88	-0.91	8.73E-18	-6.38E+08	6.38E+08
ENSG00000164620	RELL2	0.27	-3.74	-1.90	3.50E-17	-6.32E+08	6.32E+08
ENSG00000254369	HOXA-AS3	3.54	3.54	1.82	3.19E-17	6.28E+08	6.28E+08
ENSG00000109107	ALDOC	0.26	-3.79	-1.92	3.90E-17	-6.06E+08	6.06E+08
ENSG00000134107	BHLHE40	2.35	2.35	1.23	1.52E-17	6.01E+08	6.01E+08
ENSG00000166265	CYYR1	0.52	-1.91	-0.93	1.03E-17	-5.96E+08	5.96E+08
ENSG00000168060	NAALADL1	0.27	-3.74	-1.90	4.10E-17	-5.84E+08	5.84E+08
ENSG00000105135	ILVBL	0.51	-1.97	-0.98	1.25E-17	-5.58E+08	5.58E+08
ENSG00000160949	TONSL	0.44	-2.28	-1.19	1.69E-17	-5.54E+08	5.54E+08
ENSG00000132623	ANKRD5	2.08	2.08	1.06	1.44E-17	5.48E+08	5.48E+08
ENSG00000148334	PTGES2	0.53	-1.87	-0.91	1.17E-17	-5.48E+08	5.48E+08
ENSG00000120913	PDLIM2	0.48	-2.08	-1.05	1.50E-17	-5.36E+08	5.36E+08
ENSG00000101220	C20orf27	0.49	-2.05	-1.03	1.50E-17	-5.29E+08	5.29E+08
ENSG00000164062	APEH	0.51	-1.96	-0.97	1.56E-17	-4.97E+08	4.97E+08
ENSG00000124496	TRERF1	0.36	-2.80	-1.49	3.37E-17	-4.83E+08	4.83E+08
ENSG00000079335	CDC14A	1.92	1.92	0.94	1.59E-17	4.82E+08	4.82E+08
ENSG00000174446	SNAPC5	0.47	-2.13	-1.09	2.02E-17	-4.75E+08	4.75E+08
ENSG00000164284	GRPEL2	2.09	2.09	1.06	1.97E-17	4.71E+08	4.71E+08
ENSG00000173264	GPR137	2.06	2.06	1.04	2.01E-17	4.59E+08	4.59E+08
ENSG00000137269	LRRC1	0.53	-1.90	-0.93	1.81E-17	-4.48E+08	4.48E+08
ENSG00000173085	COQ2	0.50	-2.01	-1.01	2.08E-17	-4.41E+08	4.41E+08
ENSG00000073464	CLCN4	0.51	-1.97	-0.98	2.03E-17	-4.36E+08	4.36E+08
ENSG00000162692	VCAM1	0.19	-5.19	-2.38	1.42E-16	-4.36E+08	4.36E+08
ENSG00000203943	SAMD13	2.37	2.37	1.25	3.17E-17	4.21E+08	4.21E+08
ENSG00000254473	RP11-522I20.3	3.05	3.05	1.61	5.33E-17	4.18E+08	4.18E+08
ENSG00000141012	GALNS	2.30	2.30	1.20	3.20E-17	4.07E+08	4.07E+08
ENSG00000213983	AP1G2	0.45	-2.21	-1.15	2.98E-17	-4.05E+08	4.05E+08
ENSG00000103253	HAGHL	0.32	-3.09	-1.63	6.03E-17	-3.98E+08	3.98E+08
ENSG00000185100	ADSSL1	4.14	4.14	2.05	1.10E-16	3.93E+08	3.93E+08
ENSG00000052802	MSMO1	0.49	-2.04	-1.03	2.72E-17	-3.91E+08	3.91E+08
ENSG00000189077	TMEM120A	0.45	-2.22	-1.15	3.25E-17	-3.90E+08	3.90E+08
ENSG00000115486	GGCX	0.54	-1.87	-0.90	2.33E-17	-3.87E+08	3.87E+08
ENSG00000105711	SCN1B	0.44	-2.26	-1.18	3.44E-17	-3.86E+08	3.86E+08
ENSG00000106086	PLEKHA8	1.89	1.89	0.91	2.41E-17	3.84E+08	3.84E+08
ENSG00000184792	OSBP2	1.95	1.95	0.96	2.59E-17	3.83E+08	3.83E+08
ENSG00000237753	AC079922.3	3.03	3.03	1.60	6.44E-17	3.77E+08	3.77E+08
ENSG00000196639	HRH1	1.95	1.95	0.97	2.75E-17	3.73E+08	3.73E+08
ENSG00000133687	TMTC1	0.32	-3.14	-1.65	7.23E-17	-3.69E+08	3.69E+08
ENSG00000119650	IFT43	1.94	1.94	0.95	2.76E-17	3.69E+08	3.69E+08
ENSG00000163257	DCAF16	0.53	-1.88	-0.91	2.67E-17	-3.64E+08	3.64E+08
ENSG00000205220	PSMB10	0.36	-2.74	-1.46	5.69E-17	-3.64E+08	3.64E+08
ENSG00000142405	NLRP12	3.30	3.30	1.72	8.28E-17	3.62E+08	3.62E+08
ENSG00000176170	SPHK1	2.00	2.00	1.00	3.51E-17	3.38E+08	3.38E+08
ENSG00000144152	FBLN7	3.27	3.27	1.71	9.50E-17	3.35E+08	3.35E+08
ENSG00000133805	AMPD3	2.61	2.61	1.39	6.90E-17	3.15E+08	3.15E+08
ENSG00000197061	HIST1H4C	0.50	-2.02	-1.01	4.36E-17	-3.06E+08	3.06E+08
ENSG00000269728	RP11-145M9.4	1.97	1.97	0.98	4.19E-17	3.04E+08	3.04E+08
ENSG00000269343	ZNF587B	0.49	-2.04	-1.03	4.87E-17	-2.92E+08	2.92E+08
ENSG00000128340	RAC2	1.89	1.89	0.92	4.45E-17	2.83E+08	2.83E+08

ENSG00000163171	CDC42EP3	1.95	1.95	0.96	5.08E-17	2.73E+08	2.73E+08
ENSG00000162407	PPAP2B	0.52	-1.93	-0.95	5.13E-17	-2.70E+08	2.70E+08
ENSG00000108932	SLC16A6	3.74	3.74	1.90	1.92E-16	2.70E+08	2.70E+08
ENSG00000127528	KLF2	2.15	2.15	1.10	6.55E-17	2.65E+08	2.65E+08
ENSG00000164405	UQCRQ	0.49	-2.04	-1.03	6.37E-17	-2.55E+08	2.55E+08
ENSG00000132330	SCLY	0.38	-2.63	-1.40	1.07E-16	-2.54E+08	2.54E+08
	RP11-398F12.1	2.54	2.54	1.34	1.04E-16	2.48E+08	2.48E+08
ENSG00000165434	PGM2L1	2.04	2.04	1.03	7.07E-17	2.43E+08	2.43E+08
ENSG00000100028	SNRPD3	0.52	-1.92	-0.94	6.24E-17	-2.43E+08	2.43E+08
ENSG00000095059	DHPS	0.54	-1.87	-0.90	7.07E-17	-2.22E+08	2.22E+08
ENSG00000172159	FRMD3	0.44	-2.25	-1.17	1.07E-16	-2.18E+08	2.18E+08
ENSG00000142046	TMEM91	0.34	-2.93	-1.55	1.87E-16	-2.14E+08	2.14E+08
ENSG00000130508	PXDN	2.32	2.32	1.21	1.19E-16	2.13E+08	2.13E+08
ENSG00000130733	YIPF2	0.53	-1.88	-0.91	7.86E-17	-2.12E+08	2.12E+08
ENSG00000214753	HNRNPUL2	0.49	-2.04	-1.03	9.40E-17	-2.11E+08	2.11E+08
ENSG00000177000	MTHFR	1.96	1.96	0.97	8.85E-17	2.09E+08	2.09E+08
ENSG00000104823	ECH1	0.48	-2.07	-1.05	1.01E-16	-2.06E+08	2.06E+08
ENSG00000012061	ERCC1	2.04	2.04	1.03	9.86E-17	2.05E+08	2.05E+08
ENSG00000161618	ALDH16A1	0.52	-1.93	-0.95	9.27E-17	-2.01E+08	2.01E+08
ENSG00000204308	RNF5	0.52	-1.93	-0.95	9.33E-17	-2.00E+08	2.00E+08
ENSG00000107796	ACTA2	2.11	2.11	1.08	1.12E-16	2.00E+08	2.00E+08
ENSG00000100075	SLC25A1	0.53	-1.89	-0.92	8.92E-17	-2.00E+08	2.00E+08
ENSG00000162944	RFTN2	0.47	-2.13	-1.09	1.15E-16	-1.99E+08	1.99E+08
ENSG00000049130	KITLG	2.78	2.78	1.48	2.03E-16	1.95E+08	1.95E+08
ENSG00000164733	CTSB	1.94	1.94	0.96	1.01E-16	1.93E+08	1.93E+08
ENSG00000116133	DHCR24	0.52	-1.91	-0.93	1.00E-16	-1.91E+08	1.91E+08
ENSG00000246582	RP11-1149O23.3	2.27	2.27	1.18	1.43E-16	1.90E+08	1.90E+08
	AC002055.4	2.11	2.11	1.08	1.27E-16	1.88E+08	1.88E+08
ENSG00000198830	HMGN2	0.52	-1.93	-0.95	1.07E-16	-1.87E+08	1.87E+08
ENSG00000143344	RGL1	1.94	1.94	0.96	1.13E-16	1.82E+08	1.82E+08
ENSG00000276368	HIST1H2AJ	0.49	-2.02	-1.02	1.24E-16	-1.82E+08	1.82E+08
ENSG00000176209	C8orf40	1.99	1.99	1.00	1.21E-16	1.81E+08	1.81E+08
ENSG00000099937	SERPIND1	1.88	1.88	0.91	1.24E-16	1.68E+08	1.68E+08
ENSG00000231389	HLA-DPA1	0.43	-2.33	-1.22	1.98E-16	-1.66E+08	1.66E+08
ENSG00000119686	FLVCR2	0.31	-3.21	-1.68	4.00E-16	-1.61E+08	1.61E+08
ENSG00000117298	ECE1	2.41	2.41	1.27	2.26E-16	1.60E+08	1.60E+08
ENSG00000133422	MORC2	2.07	2.07	1.05	1.68E-16	1.60E+08	1.60E+08
ENSG00000164305	CASP3	1.93	1.93	0.95	1.46E-16	1.60E+08	1.60E+08
ENSG00000278272	HIST1H3C	0.50	-2.01	-1.01	1.71E-16	-1.54E+08	1.54E+08
ENSG00000112312	GMNN	0.50	-1.99	-0.99	1.70E-16	-1.53E+08	1.53E+08
ENSG00000255624	RP11-564D11.3	2.88	2.88	1.52	3.56E-16	1.52E+08	1.52E+08
ENSG00000259494	MRPL46	0.49	-2.05	-1.04	1.82E-16	-1.52E+08	1.52E+08
ENSG00000107984	DKK1	0.52	-1.92	-0.94	1.71E-16	-1.46E+08	1.46E+08
ENSG00000175309	AGXT2L2	0.53	-1.89	-0.91	1.83E-16	-1.39E+08	1.39E+08
ENSG00000111859	NEDD9	1.91	1.91	0.93	1.93E-16	1.37E+08	1.37E+08
ENSG00000127423	AUNIP	0.45	-2.21	-1.14	2.59E-16	-1.37E+08	1.37E+08
ENSG00000037897	METTL1	0.42	-2.41	-1.27	3.21E-16	-1.34E+08	1.34E+08
ENSG00000103266	STUB1	0.55	-1.83	-0.88	1.88E-16	-1.34E+08	1.34E+08
ENSG00000260948	RP11-552M11.8	3.81	3.81	1.93	8.17E-16	1.33E+08	1.33E+08
ENSG00000136159	NUDT15	0.54	-1.86	-0.89	2.00E-16	-1.31E+08	1.31E+08
	RP11-371B4.2	0.52	-1.93	-0.95	2.34E-16	-1.26E+08	1.26E+08
ENSG00000177697	CD151	0.48	-2.10	-1.07	2.84E-16	-1.25E+08	1.25E+08
ENSG00000060642	PIGV	1.99	1.99	0.99	2.56E-16	1.24E+08	1.24E+08
ENSG00000123892	RAB38	0.48	-2.08	-1.06	2.89E-16	-1.22E+08	1.22E+08
ENSG00000203706	SERTAD4-AS1	2.30	2.30	1.20	3.56E-16	1.22E+08	1.22E+08
ENSG00000260027	HOXB7	1.85	1.85	0.89	2.48E-16	1.18E+08	1.18E+08
ENSG00000197496	SLC2A10	1.89	1.89	0.92	2.59E-16	1.17E+08	1.17E+08
ENSG00000198700	IPO9	0.52	-1.94	-0.95	2.77E-16	-1.16E+08	1.16E+08
ENSG00000171680	PLEKHG5	1.90	1.90	0.93	2.72E-16	1.15E+08	1.15E+08
ENSG00000246763	CTC-463N11.3	3.31	3.31	1.73	8.56E-16	1.13E+08	1.13E+08
ENSG00000187498	COL4A1	2.05	2.05	1.04	3.30E-16	1.13E+08	1.13E+08
ENSG00000136802	LRRC8A	2.05	2.05	1.03	3.30E-16	1.13E+08	1.13E+08
ENSG00000106479	ZNF862	2.12	2.12	1.09	3.64E-16	1.11E+08	1.11E+08
ENSG00000152689	RASGRP3	2.17	2.17	1.12	3.80E-16	1.11E+08	1.11E+08
ENSG00000011422	PLAUR	1.89	1.89	0.92	2.92E-16	1.11E+08	1.11E+08
ENSG00000256940	RP11-783K16.5	2.30	2.30	1.20	4.34E-16	1.11E+08	1.11E+08
ENSG00000239305	RNF103	1.86	1.86	0.89	2.88E-16	1.09E+08	1.09E+08
ENSG00000172578	KLHL6	1.89	1.89	0.92	3.10E-16	1.08E+08	1.08E+08
ENSG00000173801	JUP	2.25	2.25	1.17	4.53E-16	1.06E+08	1.06E+08
ENSG00000206395	DDAH2	0.47	-2.14	-1.10	4.14E-16	-1.05E+08	1.05E+08
ENSG00000167695	FAM57A	0.53	-1.88	-0.91	3.22E-16	-1.05E+08	1.05E+08
ENSG00000104983	CCDC61	0.39	-2.55	-1.35	5.95E-16	-1.05E+08	1.05E+08
ENSG00000118292	C1orf54	0.47	-2.11	-1.08	4.17E-16	-1.03E+08	1.03E+08
ENSG00000119969	HELLS	0.54	-1.85	-0.88	3.23E-16	-1.03E+08	1.03E+08
ENSG00000101986	ABCD1	2.27	2.27	1.18	4.90E-16	1.03E+08	1.03E+08
ENSG00000196747	HIST1H2AI	0.50	-2.00	-1.00	3.89E-16	-1.01E+08	1.01E+08
ENSG00000157657	ZNF618	1.89	1.89	0.92	3.59E-16	9.98E+07	9.98E+07
ENSG00000204922	C11orf83	0.50	-2.00	-1.00	4.05E-16	-9.96E+07	9.96E+07
ENSG00000180488	FAM73A	1.86	1.86	0.90	3.73E-16	9.63E+07	9.63E+07

ENSG00000243926	TIPARP-AS1	3.85	3.85	1.94	1.64E-15	9.51E+07	9.51E+07
ENSG00000111057	KRT18	1.96	1.96	0.97	4.77E-16	8.98E+07	8.98E+07
ENSG00000106003	LFNG	2.73	2.73	1.45	9.33E-16	8.94E+07	8.94E+07
ENSG00000131944	C19orf40	2.05	2.05	1.03	5.33E-16	8.87E+07	8.87E+07
ENSG00000006757	PNPLA4	2.00	2.00	1.00	5.16E-16	8.81E+07	8.81E+07
ENSG00000099377	HSD3B7	0.26	-3.83	-1.94	1.98E-15	-8.62E+07	8.62E+07
ENSG00000174175	SELP	0.30	-3.36	-1.75	1.57E-15	-8.49E+07	8.49E+07
ENSG00000149743	TRPT1	0.50	-2.02	-1.01	5.67E-16	-8.47E+07	8.47E+07
ENSG00000137965	IFI44	0.52	-1.93	-0.95	5.18E-16	-8.47E+07	8.47E+07
ENSG00000065328	MCM10	0.54	-1.84	-0.88	4.88E-16	-8.32E+07	8.32E+07
ENSG00000143643	TTC13	2.05	2.05	1.04	6.13E-16	8.30E+07	8.30E+07
ENSG00000145675	PIK3R1	1.95	1.95	0.96	5.67E-16	8.18E+07	8.18E+07
ENSG00000104998	IL27RA	0.36	-2.80	-1.49	1.20E-15	-8.09E+07	8.09E+07
ENSG00000277224	HIST1H2BF	0.51	-1.97	-0.98	6.01E-16	-8.04E+07	8.04E+07
ENSG00000130714	POMT1	0.48	-2.07	-1.05	6.71E-16	-8.00E+07	8.00E+07
ENSG00000243244	STON1	0.52	-1.93	-0.95	6.13E-16	-7.80E+07	7.80E+07
ENSG00000138030	KHK	0.33	-3.04	-1.61	1.54E-15	-7.76E+07	7.76E+07
ENSG00000124766	SOX4	1.96	1.96	0.97	6.72E-16	7.54E+07	7.54E+07
ENSG00000213337	ANKRD39	0.34	-2.95	-1.56	1.53E-15	-7.53E+07	7.53E+07
ENSG00000184307	ZDHHC23	0.42	-2.36	-1.24	1.02E-15	-7.39E+07	7.39E+07
ENSG00000171681	ATF7IP	1.88	1.88	0.91	7.08E-16	7.07E+07	7.07E+07
ENSG00000183751	TBL3	0.52	-1.92	-0.94	7.42E-16	-7.05E+07	7.05E+07
ENSG00000121281	ADCY7	0.38	-2.64	-1.40	1.41E-15	-7.02E+07	7.02E+07
ENSG00000109736	MFSD10	0.53	-1.90	-0.93	7.35E-16	-7.02E+07	7.02E+07
ENSG00000230479	AP000695.6	1.90	1.90	0.92	7.31E-16	7.01E+07	7.01E+07
ENSG00000166033	HTRA1	1.90	1.90	0.93	8.00E-16	6.73E+07	6.73E+07
ENSG00000160957	RECQL4	0.51	-1.98	-0.98	8.68E-16	-6.70E+07	6.70E+07
ENSG00000169972	PUSL1	0.49	-2.04	-1.03	9.64E-16	-6.58E+07	6.58E+07
ENSG00000085982	USP40	1.89	1.89	0.92	8.63E-16	6.43E+07	6.43E+07
ENSG00000112667	C6orf108	0.51	-1.97	-0.98	9.54E-16	-6.37E+07	6.37E+07
ENSG00000130962	PRRG1	1.91	1.91	0.94	9.44E-16	6.23E+07	6.23E+07
ENSG00000170190	SLC16A5	2.17	2.17	1.12	1.24E-15	6.15E+07	6.15E+07
ENSG00000176809	LRRC37A3	2.66	2.66	1.41	1.92E-15	6.06E+07	6.06E+07
ENSG00000154358	OBSCN	2.40	2.40	1.26	1.63E-15	5.94E+07	5.94E+07
ENSG00000130066	SAT1	0.53	-1.89	-0.92	1.03E-15	-5.91E+07	5.91E+07
ENSG00000167925	GHDC	0.45	-2.24	-1.16	1.48E-15	-5.81E+07	5.81E+07
ENSG00000138744	NAAA	0.48	-2.09	-1.07	1.31E-15	-5.79E+07	5.79E+07
ENSG00000005073	HOXA11	1.87	1.87	0.91	1.09E-15	5.67E+07	5.67E+07
ENSG00000198055	GRK6	0.54	-1.85	-0.89	1.12E-15	-5.52E+07	5.52E+07
ENSG00000102226	USP11	0.48	-2.07	-1.05	1.41E-15	-5.50E+07	5.50E+07
ENSG00000160055	TMEM234	0.46	-2.16	-1.11	1.54E-15	-5.49E+07	5.49E+07
ENSG00000090013	BLVRB	1.85	1.85	0.89	1.14E-15	5.47E+07	5.47E+07
ENSG00000185567	AHNAK2	2.02	2.02	1.01	1.36E-15	5.47E+07	5.47E+07
ENSG00000213694	S1PR3	0.54	-1.84	-0.88	1.17E-15	-5.37E+07	5.37E+07
ENSG00000134574	DDB2	1.88	1.88	0.91	1.27E-15	5.27E+07	5.27E+07
ENSG00000231607	DLEU2	0.53	-1.89	-0.92	1.33E-15	-5.20E+07	5.20E+07
ENSG00000143942	CHAC2	0.52	-1.93	-0.95	1.47E-15	-5.03E+07	5.03E+07
ENSG00000151491	EPS8	0.48	-2.09	-1.06	1.76E-15	-4.99E+07	4.99E+07
ENSG00000005187	ACSM3	2.78	2.78	1.48	3.17E-15	4.94E+07	4.94E+07
ENSG00000144036	EXOC6B	1.88	1.88	0.91	1.52E-15	4.81E+07	4.81E+07
ENSG00000122386	ZNF205	0.51	-1.98	-0.99	1.74E-15	-4.74E+07	4.74E+07
ENSG00000151151	IPMK	2.08	2.08	1.06	1.94E-15	4.72E+07	4.72E+07
ENSG00000254703	FLI1-AS1	2.07	2.07	1.05	1.97E-15	4.66E+07	4.66E+07
ENSG00000137216	TMEM63B	1.83	1.83	0.88	1.64E-15	4.53E+07	4.53E+07
	RP11-327L3.1	0.43	-2.32	-1.21	2.63E-15	-4.52E+07	4.52E+07
ENSG00000158195	WASF2	1.93	1.93	0.95	1.88E-15	4.44E+07	4.44E+07
ENSG00000165071	TMEM71	0.35	-2.89	-1.53	4.32E-15	-4.40E+07	4.40E+07
ENSG00000272849	AC084018.1	2.19	2.19	1.13	2.61E-15	4.30E+07	4.30E+07
ENSG00000255088	CCDC111	2.06	2.06	1.04	2.30E-15	4.30E+07	4.30E+07
ENSG00000175063	UBE2C	0.54	-1.86	-0.90	1.94E-15	-4.23E+07	4.23E+07
ENSG00000136295	TTYH3	1.88	1.88	0.91	2.02E-15	4.18E+07	4.18E+07
ENSG00000197928	ZNF677	1.96	1.96	0.97	2.20E-15	4.17E+07	4.17E+07
ENSG00000109472	CPE	0.41	-2.46	-1.30	3.52E-15	-4.15E+07	4.15E+07
ENSG00000167522	ANKRD11	1.88	1.88	0.91	2.06E-15	4.15E+07	4.15E+07
ENSG00000132170	PPARG	0.38	-2.67	-1.41	4.35E-15	-4.04E+07	4.04E+07
ENSG00000196498	NCOR2	1.80	1.80	0.85	2.02E-15	4.02E+07	4.02E+07
ENSG00000196843	ARID5A	1.86	1.86	0.89	2.18E-15	3.98E+07	3.98E+07
ENSG00000277656	GSTT1	0.50	-2.00	-1.00	2.65E-15	-3.89E+07	3.89E+07
ENSG00000174177	CTU2	0.52	-1.92	-0.94	2.64E-15	-3.74E+07	3.74E+07
ENSG00000162618	ELTD1	1.90	1.90	0.93	2.74E-15	3.64E+07	3.64E+07
ENSG00000241553	ARPC4	0.52	-1.93	-0.95	2.90E-15	-3.59E+07	3.59E+07
ENSG00000115271	GCA	0.49	-2.02	-1.02	3.29E-15	-3.53E+07	3.53E+07
ENSG00000132669	RIN2	1.82	1.82	0.86	2.68E-15	3.52E+07	3.52E+07
ENSG00000166398	KIAA0355	1.93	1.93	0.95	3.01E-15	3.51E+07	3.51E+07
ENSG00000167264	DUS2L	0.53	-1.87	-0.91	2.95E-15	-3.45E+07	3.45E+07
ENSG00000148450	MSRB2	1.91	1.91	0.93	3.11E-15	3.41E+07	3.41E+07
ENSG00000151729	SLC25A4	0.53	-1.89	-0.91	3.08E-15	-3.40E+07	3.40E+07
ENSG00000206903	SNORA24	0.38	-2.62	-1.39	6.42E-15	-3.27E+07	3.27E+07
ENSG00000132780	NASP	0.53	-1.89	-0.92	3.35E-15	-3.27E+07	3.27E+07

ENSG00000127586	CHTF18	0.51	-1.95	-0.96	3.58E-15	-3.26E+07	3.26E+07
ENSG00000163762	TM4SF18	2.06	2.06	1.05	4.08E-15	3.23E+07	3.23E+07
ENSG00000137501	SYTL2	3.38	3.38	1.76	1.10E-14	3.23E+07	3.23E+07
ENSG00000125148	MT2A	0.52	-1.92	-0.94	3.52E-15	-3.23E+07	3.23E+07
ENSG00000184205	TSPYL2	0.47	-2.13	-1.09	4.56E-15	-3.16E+07	3.16E+07
ENSG00000213563	C8orf82	0.49	-2.06	-1.04	4.56E-15	-3.05E+07	3.05E+07
ENSG00000133874	RNF122	2.03	2.03	1.02	4.51E-15	3.02E+07	3.02E+07
	CTD-3211M3.1	2.66	2.66	1.41	7.83E-15	3.00E+07	3.00E+07
ENSG00000196878	LAMB3	2.60	2.60	1.38	7.71E-15	2.96E+07	2.96E+07
ENSG00000187193	MT1X	0.34	-2.91	-1.54	9.93E-15	-2.92E+07	2.92E+07
ENSG00000181019	NQO1	0.51	-1.95	-0.97	4.51E-15	-2.91E+07	2.91E+07
ENSG00000228203	RNF144A-AS1	2.44	2.44	1.29	7.08E-15	2.90E+07	2.90E+07
ENSG00000127603	KIAA0754	1.91	1.91	0.94	4.38E-15	2.89E+07	2.89E+07
ENSG00000166197	NOLC1	0.54	-1.87	-0.90	4.21E-15	-2.88E+07	2.88E+07
ENSG00000187210	GCNT1	0.53	-1.88	-0.91	4.42E-15	-2.82E+07	2.82E+07
ENSG00000270281	AC020910.2	3.26	3.26	1.71	1.34E-14	2.82E+07	2.82E+07
ENSG00000260742	RP11-366L5.1	2.70	2.70	1.43	9.20E-15	2.82E+07	2.82E+07
ENSG00000162572	SCNN1D	3.58	3.58	1.84	1.65E-14	2.79E+07	2.79E+07
ENSG00000159788	RGS12	1.82	1.82	0.87	4.36E-15	2.76E+07	2.76E+07
ENSG00000184260	HIST2H2AC	0.55	-1.83	-0.87	4.45E-15	-2.75E+07	2.75E+07
ENSG00000166750	SLFN5	2.02	2.02	1.02	5.43E-15	2.74E+07	2.74E+07
ENSG00000178209	PLEC	2.00	2.00	1.00	5.36E-15	2.74E+07	2.74E+07
ENSG00000162241	SLC25A45	2.85	2.85	1.51	1.11E-14	2.70E+07	2.70E+07
ENSG00000106683	LIMK1	1.87	1.87	0.90	4.85E-15	2.68E+07	2.68E+07
ENSG00000079277	MKNK1	0.54	-1.85	-0.89	5.30E-15	-2.54E+07	2.54E+07
ENSG00000169760	NLGN1	0.52	-1.93	-0.95	5.87E-15	-2.52E+07	2.52E+07
ENSG00000213901	SLC23A3	3.10	3.10	1.63	1.55E-14	2.49E+07	2.49E+07
ENSG00000176678	FOXL1	2.37	2.37	1.25	9.50E-15	2.43E+07	2.43E+07
ENSG00000137959	IFI44L	0.33	-3.05	-1.61	1.69E-14	-2.35E+07	2.35E+07
ENSG00000163596	ICA1L	0.43	-2.33	-1.22	1.00E-14	-2.33E+07	2.33E+07
ENSG00000143553	SNAPIN	0.54	-1.86	-0.89	6.39E-15	-2.32E+07	2.32E+07
ENSG00000136859	ANGPTL2	1.79	1.79	0.84	6.03E-15	2.31E+07	2.31E+07
ENSG00000198753	PLXNB3	0.30	-3.39	-1.76	2.16E-14	-2.31E+07	2.31E+07
ENSG00000108784	NAGLU	1.85	1.85	0.89	6.50E-15	2.29E+07	2.29E+07
ENSG00000259070	LINC00639	2.93	2.93	1.55	1.69E-14	2.25E+07	2.25E+07
ENSG00000073711	PPP2R3A	1.92	1.92	0.94	7.45E-15	2.22E+07	2.22E+07
ENSG00000203644	AC083799.1	2.06	2.06	1.04	8.84E-15	2.19E+07	2.19E+07
ENSG00000184164	CRELD2	0.51	-1.98	-0.98	8.32E-15	-2.17E+07	2.17E+07
ENSG00000144120	TMEM177	0.52	-1.94	-0.95	8.04E-15	-2.16E+07	2.16E+07
ENSG00000166902	MRPL16	0.56	-1.79	-0.84	6.95E-15	-2.15E+07	2.15E+07
ENSG00000117318	ID3	0.54	-1.86	-0.89	7.63E-15	-2.13E+07	2.13E+07
ENSG00000143816	WNT9A	2.56	2.56	1.36	1.58E-14	2.04E+07	2.04E+07
ENSG00000177406	RP11-218M22.1	2.25	2.25	1.17	1.23E-14	2.03E+07	2.03E+07
ENSG00000163092	XIRP2	5.25	5.25	2.39	6.72E-14	2.02E+07	2.02E+07
ENSG00000089127	OAS1	0.52	-1.94	-0.95	9.32E-15	-2.01E+07	2.01E+07
ENSG00000169439	SDC2	0.44	-2.28	-1.19	1.30E-14	-2.00E+07	2.00E+07
ENSG00000133069	TMCC2	2.41	2.41	1.27	1.47E-14	1.99E+07	1.99E+07
ENSG00000167900	TK1	0.56	-1.79	-0.84	8.37E-15	-1.96E+07	1.96E+07
ENSG00000124587	PEX6	1.88	1.88	0.91	9.23E-15	1.95E+07	1.95E+07
ENSG00000105810	CDK6	1.80	1.80	0.85	9.08E-15	1.89E+07	1.89E+07
ENSG00000075618	FSCN1	1.86	1.86	0.90	9.87E-15	1.87E+07	1.87E+07
ENSG00000154146	NRGN	0.39	-2.57	-1.36	1.93E-14	-1.85E+07	1.85E+07
ENSG00000120693	SMAD9	0.55	-1.82	-0.87	9.75E-15	-1.85E+07	1.85E+07
ENSG00000253293	HOXA10	0.53	-1.88	-0.91	1.05E-14	-1.84E+07	1.84E+07
ENSG00000067798	NAV3	1.87	1.87	0.90	1.07E-14	1.81E+07	1.81E+07
ENSG00000064687	ABCA7	3.13	3.13	1.64	3.08E-14	1.78E+07	1.78E+07
ENSG00000146072	TNFRSF21	2.00	2.00	1.00	1.30E-14	1.76E+07	1.76E+07
ENSG00000170891	CYTL1	1.81	1.81	0.86	1.16E-14	1.68E+07	1.68E+07
ENSG00000199753	SNORD104	0.27	-3.74	-1.90	5.01E-14	-1.67E+07	1.67E+07
ENSG00000180592	SKIDA1	2.72	2.72	1.45	2.66E-14	1.67E+07	1.67E+07
ENSG00000125629	INSIG2	1.76	1.76	0.82	1.12E-14	1.66E+07	1.66E+07
ENSG00000204856	FAM216A	0.53	-1.90	-0.93	1.34E-14	-1.64E+07	1.64E+07
ENSG00000170873	MTSS1	1.81	1.81	0.85	1.22E-14	1.64E+07	1.64E+07
ENSG00000174371	EXO1	0.56	-1.78	-0.83	1.22E-14	-1.61E+07	1.61E+07
ENSG00000114796	KLHL24	1.91	1.91	0.93	1.45E-14	1.58E+07	1.58E+07
ENSG00000198276	UCKL1	0.55	-1.82	-0.86	1.37E-14	-1.55E+07	1.55E+07
ENSG00000064199	SPA17	1.87	1.87	0.90	1.46E-14	1.54E+07	1.54E+07
ENSG00000068305	MEF2A	1.80	1.80	0.85	1.38E-14	1.54E+07	1.54E+07
ENSG00000008513	ST3GAL1	1.86	1.86	0.90	1.51E-14	1.51E+07	1.51E+07
ENSG00000147459	DOCK5	1.84	1.84	0.88	1.50E-14	1.51E+07	1.51E+07
ENSG00000157593	SLC35B2	0.54	-1.85	-0.89	1.52E-14	-1.50E+07	1.50E+07
ENSG00000178026	FAM211B	0.38	-2.64	-1.40	3.11E-14	-1.50E+07	1.50E+07
	RP11-566E18.3	1.98	1.98	0.99	1.79E-14	1.48E+07	1.48E+07
ENSG00000122592	HOXA7	1.88	1.88	0.91	1.65E-14	1.47E+07	1.47E+07
ENSG00000128335	APOL2	0.50	-2.02	-1.01	1.94E-14	-1.45E+07	1.45E+07
ENSG00000103254	FAM173A	0.48	-2.07	-1.05	2.06E-14	-1.45E+07	1.45E+07
ENSG00000000971	CFH	0.49	-2.04	-1.03	2.01E-14	-1.44E+07	1.44E+07
ENSG00000184083	FAM120C	1.84	1.84	0.88	1.78E-14	1.38E+07	1.38E+07
ENSG00000100395	L3MBTL2	0.57	-1.77	-0.82	1.64E-14	-1.38E+07	1.38E+07

ENSG00000157168	NRG1	0.53	-1.90	-0.93	1.91E-14	-1.38E+07	1.38E+07
ENSG00000211445	GPX3	0.36	-2.79	-1.48	4.18E-14	-1.37E+07	1.37E+07
ENSG00000206535	LNP1	2.93	2.93	1.55	5.01E-14	1.31E+07	1.31E+07
ENSG00000113119	TMCO6	0.48	-2.08	-1.05	2.64E-14	-1.28E+07	1.28E+07
ENSG00000162694	EXTL2	0.53	-1.89	-0.92	2.21E-14	-1.27E+07	1.27E+07
ENSG00000171115	GIMAP8	0.54	-1.85	-0.88	2.11E-14	-1.27E+07	1.27E+07
ENSG00000171227	TMEM37	0.41	-2.47	-1.30	4.07E-14	-1.22E+07	1.22E+07
ENSG00000089723	OTUB2	2.50	2.50	1.32	4.28E-14	1.21E+07	1.21E+07
ENSG00000183722	LHFP	0.56	-1.77	-0.83	2.22E-14	-1.19E+07	1.19E+07
ENSG00000160271	RALGDS	1.83	1.83	0.87	2.36E-14	1.19E+07	1.19E+07
ENSG00000205336	GPR56	1.99	1.99	0.99	2.90E-14	1.17E+07	1.17E+07
ENSG00000197081	IGF2R	1.96	1.96	0.97	2.88E-14	1.16E+07	1.16E+07
ENSG00000110047	EHD1	2.08	2.08	1.06	3.25E-14	1.15E+07	1.15E+07
ENSG00000134884	ARGLU1	0.53	-1.90	-0.93	2.82E-14	-1.13E+07	1.13E+07
ENSG00000172086	KRCC1	1.83	1.83	0.88	2.67E-14	1.12E+07	1.12E+07
ENSG00000167216	KATNAL2	2.96	2.96	1.57	7.48E-14	1.08E+07	1.08E+07
ENSG00000101400	SNTA1	2.02	2.02	1.02	3.51E-14	1.08E+07	1.08E+07
ENSG00000213625	LEPROT	1.83	1.83	0.87	2.91E-14	1.07E+07	1.07E+07
ENSG00000143622	RIT1	0.53	-1.90	-0.92	3.14E-14	-1.07E+07	1.07E+07
ENSG00000119899	SLC17A5	1.84	1.84	0.88	3.04E-14	1.06E+07	1.06E+07
ENSG00000112139	MDGA1	3.34	3.34	1.74	1.01E-13	1.05E+07	1.05E+07
ENSG00000106733	NMRK1	1.77	1.77	0.83	2.86E-14	1.05E+07	1.05E+07
ENSG00000104967	NOVA2	1.93	1.93	0.95	3.57E-14	1.02E+07	1.02E+07
ENSG00000019991	HGF	0.28	-3.55	-1.83	1.22E-13	-1.02E+07	1.02E+07
ENSG00000176714	CCDC121	2.46	2.46	1.30	5.96E-14	1.01E+07	1.01E+07
ENSG00000103855	CD276	1.91	1.91	0.94	3.82E-14	9.80E+06	9.80E+06
ENSG00000065427	KARS	0.55	-1.82	-0.87	3.47E-14	-9.79E+06	9.79E+06
ENSG00000181396	OGFOD3	0.56	-1.79	-0.84	3.55E-14	-9.53E+06	9.53E+06
ENSG00000255198	SNHG9	0.39	-2.58	-1.37	7.52E-14	-9.42E+06	9.42E+06
ENSG00000178035	IMPDH2	0.55	-1.83	-0.87	3.80E-14	-9.41E+06	9.41E+06
ENSG00000068383	INPP5A	1.77	1.77	0.82	3.64E-14	9.26E+06	9.26E+06
ENSG00000101670	LIPG	0.54	-1.84	-0.88	3.96E-14	-9.23E+06	9.23E+06
ENSG00000085415	SEH1L	0.54	-1.84	-0.88	4.04E-14	-9.17E+06	9.17E+06
ENSG00000233822	HIST1H2BN	0.54	-1.84	-0.88	4.27E-14	-8.93E+06	8.93E+06
ENSG00000122952	ZWINT	0.56	-1.79	-0.84	4.22E-14	-8.71E+06	8.71E+06
ENSG00000218336	TENM3	1.99	1.99	0.99	5.60E-14	8.41E+06	8.41E+06
ENSG00000111335	OAS2	0.44	-2.25	-1.17	7.32E-14	-8.32E+06	8.32E+06
ENSG00000109854	HTATIP2	1.82	1.82	0.86	4.91E-14	8.21E+06	8.21E+06
ENSG00000103021	CCDC113	2.28	2.28	1.19	7.71E-14	8.20E+06	8.20E+06
ENSG00000008853	RHOBTB2	0.53	-1.90	-0.93	5.61E-14	-8.04E+06	8.04E+06
ENSG00000124787	RPP40	0.56	-1.79	-0.84	5.11E-14	-7.91E+06	7.91E+06
ENSG00000071073	MGAT4A	1.92	1.92	0.94	5.91E-14	7.88E+06	7.88E+06
ENSG00000023697	DERA	0.57	-1.74	-0.80	4.90E-14	-7.88E+06	7.88E+06
ENSG00000154016	GRAP	1.76	1.76	0.82	5.01E-14	7.86E+06	7.86E+06
ENSG00000107560	RAB11FIP2	1.79	1.79	0.84	5.34E-14	7.76E+06	7.76E+06
ENSG00000100297	MCM5	0.52	-1.91	-0.93	6.11E-14	-7.73E+06	7.73E+06
ENSG00000103037	SETD6	0.43	-2.30	-1.20	8.88E-14	-7.72E+06	7.72E+06
ENSG00000164039	BDH2	0.52	-1.91	-0.93	6.27E-14	-7.62E+06	7.62E+06
ENSG00000226053	RP5-1070A16.1	2.36	2.36	1.24	9.64E-14	7.60E+06	7.60E+06
ENSG00000161956	SENP3	0.57	-1.77	-0.82	5.47E-14	-7.57E+06	7.57E+06
ENSG00000114388	NPRL2	0.52	-1.92	-0.94	6.78E-14	-7.38E+06	7.38E+06
ENSG00000225190	PLEKHM1	1.87	1.87	0.90	6.42E-14	7.38E+06	7.38E+06
ENSG00000007866	TEAD3	3.31	3.31	1.72	2.06E-13	7.28E+06	7.28E+06
ENSG00000185262	FAM100B	1.93	1.93	0.95	7.45E-14	7.07E+06	7.07E+06
ENSG00000086189	DIMT1	0.57	-1.76	-0.81	6.45E-14	-6.92E+06	6.92E+06
ENSG00000139637	C12orf10	0.55	-1.81	-0.85	7.15E-14	-6.76E+06	6.76E+06
ENSG00000171307	ZDHHC16	0.57	-1.76	-0.81	7.21E-14	-6.54E+06	6.54E+06
ENSG00000136935	GOLGA1	1.75	1.75	0.81	7.45E-14	6.41E+06	6.41E+06
ENSG00000101888	NXT2	1.88	1.88	0.91	9.75E-14	6.02E+06	6.02E+06
ENSG00000008517	IL32	2.12	2.12	1.08	1.25E-13	5.99E+06	5.99E+06
ENSG00000012232	EXTL3	1.91	1.91	0.93	1.03E-13	5.95E+06	5.95E+06
ENSG00000104689	TNFRSF10A	1.75	1.75	0.80	8.67E-14	5.93E+06	5.93E+06
ENSG00000160813	PPP1R35	0.55	-1.81	-0.85	9.44E-14	-5.89E+06	5.89E+06
	RP11-124N14.4	2.88	2.88	1.53	2.47E-13	5.80E+06	5.80E+06
ENSG00000146376	ARHGAP18	2.10	2.10	1.07	1.31E-13	5.78E+06	5.78E+06
ENSG00000213977	TAX1BP3	0.47	-2.11	-1.07	1.33E-13	-5.77E+06	5.77E+06
ENSG00000125734	GPR108	1.77	1.77	0.82	9.48E-14	5.75E+06	5.75E+06
ENSG00000114378	HYAL1	2.94	2.94	1.56	2.75E-13	5.61E+06	5.61E+06
ENSG00000185379	RAD51D	0.48	-2.07	-1.05	1.47E-13	-5.41E+06	5.41E+06
ENSG00000253953	PCDHGB4	2.08	2.08	1.06	1.48E-13	5.40E+06	5.40E+06
	AC017048.4	3.32	3.32	1.73	3.84E-13	5.35E+06	5.35E+06
ENSG00000155115	GTF3C6	0.57	-1.74	-0.80	1.07E-13	-5.33E+06	5.33E+06
ENSG00000173846	PLK3	1.78	1.78	0.83	1.19E-13	5.15E+06	5.15E+06
ENSG00000230148	HOXB-AS1	2.93	2.93	1.55	3.22E-13	5.15E+06	5.15E+06
	RP11-318M2.2	2.79	2.79	1.48	2.97E-13	5.12E+06	5.12E+06
ENSG00000166446	CDYL2	1.77	1.77	0.82	1.25E-13	5.00E+06	5.00E+06
ENSG00000079156	OSBPL6	0.41	-2.44	-1.29	2.51E-13	-4.87E+06	4.87E+06
ENSG00000167552	TUBA1A	1.82	1.82	0.86	1.39E-13	4.87E+06	4.87E+06
ENSG00000124610	HIST1H1A	0.53	-1.90	-0.93	1.54E-13	-4.85E+06	4.85E+06

ENSG00000196290	NIF3L1	0.54	-1.86	-0.90	1.53E-13	-4.76E+06	4.76E+06
ENSG00000253368	TRNP1	1.82	1.82	0.86	1.46E-13	4.75E+06	4.75E+06
ENSG00000100345	MYH9	2.04	2.04	1.03	1.88E-13	4.71E+06	4.71E+06
ENSG00000251432	RP11-420A23.1	3.20	3.20	1.68	4.67E-13	4.69E+06	4.69E+06
ENSG00000186496	ZNF396	2.64	2.64	1.40	3.29E-13	4.61E+06	4.61E+06
ENSG00000186409	CCDC30	2.83	2.83	1.50	3.79E-13	4.60E+06	4.60E+06
ENSG00000174628	IQCK	1.84	1.84	0.88	1.60E-13	4.59E+06	4.59E+06
ENSG00000105327	BBC3	1.78	1.78	0.84	1.54E-13	4.55E+06	4.55E+06
ENSG00000153071	DAB2	1.85	1.85	0.88	1.65E-13	4.55E+06	4.55E+06
ENSG00000134242	PTPN22	0.43	-2.32	-1.21	2.71E-13	-4.45E+06	4.45E+06
ENSG00000010404	IDS	1.83	1.83	0.87	1.72E-13	4.40E+06	4.40E+06
ENSG00000123908	EIF2C2	1.84	1.84	0.88	1.80E-13	4.33E+06	4.33E+06
ENSG00000137752	CASP1	0.37	-2.72	-1.44	4.24E-13	-4.18E+06	4.18E+06
ENSG00000134668	SPOCD1	1.80	1.80	0.85	1.93E-13	4.10E+06	4.10E+06
ENSG00000181350	FAM211A	0.34	-2.93	-1.55	5.14E-13	-4.08E+06	4.08E+06
ENSG00000134215	VAV3	2.39	2.39	1.26	3.44E-13	4.07E+06	4.07E+06
ENSG00000227268	KLLN	3.03	3.03	1.60	5.55E-13	4.06E+06	4.06E+06
ENSG00000079308	TNS1	1.74	1.74	0.80	1.96E-13	3.93E+06	3.93E+06
ENSG00000135392	DNAJC14	0.56	-1.78	-0.83	2.13E-13	-3.85E+06	3.85E+06
ENSG00000131759	RARA	1.95	1.95	0.96	2.58E-13	3.84E+06	3.84E+06
ENSG00000143387	CTSK	0.51	-1.96	-0.97	2.76E-13	-3.73E+06	3.73E+06
ENSG00000105737	GRIK5	3.03	3.03	1.60	6.62E-13	3.72E+06	3.72E+06
ENSG00000141295	SCRN2	0.56	-1.78	-0.83	2.32E-13	-3.70E+06	3.70E+06
ENSG00000242485	MRPL20	0.58	-1.73	-0.79	2.26E-13	-3.65E+06	3.65E+06
ENSG00000181852	RNF41	0.57	-1.74	-0.80	2.34E-13	-3.60E+06	3.60E+06
ENSG00000138336	TET1	0.38	-2.61	-1.39	5.27E-13	-3.60E+06	3.60E+06
	CTD-2003C8.1	2.04	2.04	1.03	3.22E-13	3.59E+06	3.59E+06
ENSG00000130309	GLT25D1	1.80	1.80	0.85	2.51E-13	3.59E+06	3.59E+06
ENSG00000183155	RABIF	0.54	-1.84	-0.88	2.62E-13	-3.59E+06	3.59E+06
ENSG00000172716	SLFN11	0.53	-1.87	-0.90	2.74E-13	-3.57E+06	3.57E+06
ENSG00000033030	ZCCHC8	0.56	-1.78	-0.83	2.53E-13	-3.54E+06	3.54E+06
ENSG00000162545	CAMK2N1	2.02	2.02	1.01	3.29E-13	3.52E+06	3.52E+06
ENSG00000172888	ZNF621	1.76	1.76	0.81	2.52E-13	3.50E+06	3.50E+06
ENSG00000179528	LBX2	2.38	2.38	1.25	4.72E-13	3.46E+06	3.46E+06
ENSG00000185033	SEMA4B	2.30	2.30	1.20	4.66E-13	3.37E+06	3.37E+06
ENSG00000174516	PELI3	0.39	-2.59	-1.37	5.90E-13	-3.37E+06	3.37E+06
ENSG00000150456	N6AMT2	1.80	1.80	0.85	2.95E-13	3.33E+06	3.33E+06
ENSG00000083807	SLC27A5	0.44	-2.29	-1.19	4.75E-13	-3.32E+06	3.32E+06
ENSG00000112624	KIAA0240	1.88	1.88	0.91	3.32E-13	3.27E+06	3.27E+06
ENSG00000163297	ANTXR2	1.78	1.78	0.83	3.07E-13	3.21E+06	3.21E+06
ENSG00000174004	LRRC33	2.60	2.60	1.38	6.75E-13	3.16E+06	3.16E+06
ENSG00000014138	POLA2	0.56	-1.79	-0.84	3.20E-13	-3.16E+06	3.16E+06
ENSG00000213949	ITGA1	0.41	-2.46	-1.30	6.29E-13	-3.10E+06	3.10E+06
ENSG00000108262	GIT1	1.75	1.75	0.81	3.19E-13	3.10E+06	3.10E+06
ENSG00000152953	STK32B	1.81	1.81	0.85	3.40E-13	3.10E+06	3.10E+06
ENSG00000221968	FADS3	1.79	1.79	0.84	3.38E-13	3.07E+06	3.07E+06
ENSG00000068971	PPP2R5B	1.76	1.76	0.82	3.33E-13	3.05E+06	3.05E+06
ENSG00000124608	AARS2	0.57	-1.75	-0.81	3.34E-13	-3.03E+06	3.03E+06
ENSG00000169247	SH3TC2	1.93	1.93	0.95	4.33E-13	2.93E+06	2.93E+06
ENSG00000204673	AKT1S1	1.77	1.77	0.83	3.73E-13	2.91E+06	2.91E+06
ENSG00000119760	SUPT7L	0.58	-1.72	-0.78	3.55E-13	-2.89E+06	2.89E+06
ENSG00000163382	APOA1BP	0.58	-1.73	-0.79	3.63E-13	-2.88E+06	2.88E+06
ENSG00000117151	CTBS	1.79	1.79	0.84	4.08E-13	2.80E+06	2.80E+06
ENSG00000121775	TMEM39B	0.56	-1.78	-0.83	4.07E-13	-2.79E+06	2.79E+06
ENSG00000165072	MAMDC2	2.34	2.34	1.23	7.36E-13	2.73E+06	2.73E+06
ENSG00000230606	AC159540.1	2.35	2.35	1.23	7.52E-13	2.71E+06	2.71E+06
ENSG00000204514	ZNF814	0.42	-2.37	-1.24	8.08E-13	-2.63E+06	2.63E+06
ENSG00000110871	COQ5	0.56	-1.77	-0.83	4.58E-13	-2.62E+06	2.62E+06
ENSG00000124440	HIF3A	0.38	-2.65	-1.41	1.04E-12	-2.60E+06	2.60E+06
ENSG00000204439	C6orf47	0.56	-1.79	-0.84	5.07E-13	-2.51E+06	2.51E+06
ENSG00000137288	MNF1	0.53	-1.89	-0.92	5.79E-13	-2.49E+06	2.49E+06
ENSG00000110721	CHKA	1.78	1.78	0.83	5.13E-13	2.48E+06	2.48E+06
ENSG00000243943	ZNF512	0.56	-1.79	-0.84	5.20E-13	-2.48E+06	2.48E+06
ENSG00000272729	AC009336.1	3.48	3.48	1.80	2.09E-12	2.41E+06	2.41E+06
ENSG00000213551	DNAJC9	0.57	-1.76	-0.82	5.37E-13	-2.40E+06	2.40E+06
ENSG00000088340	FER1L4	3.97	3.97	1.99	2.82E-12	2.36E+06	2.36E+06
ENSG00000140511	HAPLN3	0.54	-1.86	-0.90	6.52E-13	-2.31E+06	2.31E+06
ENSG00000090376	IRAK3	0.58	-1.72	-0.78	5.55E-13	-2.30E+06	2.30E+06
ENSG00000169189	NSMCE1	1.75	1.75	0.81	5.92E-13	2.28E+06	2.28E+06
ENSG00000185278	ZBTB37	2.01	2.01	1.01	7.84E-13	2.27E+06	2.27E+06
ENSG00000122970	IFT81	1.84	1.84	0.88	6.65E-13	2.26E+06	2.26E+06
ENSG00000260549	MT1L	0.54	-1.85	-0.89	6.80E-13	-2.25E+06	2.25E+06
ENSG00000143314	MRPL24	0.58	-1.71	-0.78	5.94E-13	-2.22E+06	2.22E+06
ENSG00000145287	PLAC8	0.32	-3.16	-1.66	2.06E-12	-2.20E+06	2.20E+06
ENSG00000278463	HIST1H2AB	0.57	-1.74	-0.80	6.46E-13	-2.17E+06	2.17E+06
ENSG00000162616	DNAJB4	0.53	-1.89	-0.92	7.71E-13	-2.15E+06	2.15E+06
ENSG00000178149	DALRD3	0.55	-1.82	-0.87	7.35E-13	-2.13E+06	2.13E+06
ENSG00000196781	TLE1	1.79	1.79	0.84	7.43E-13	2.08E+06	2.08E+06
ENSG00000003249	DBNDD1	1.76	1.76	0.81	7.22E-13	2.07E+06	2.07E+06

ENSG00000122557	HERPUD2	1.74	1.74	0.80	7.16E-13	2.06E+06	2.06E+06
ENSG00000186665	C17orf58	0.57	-1.76	-0.82	7.32E-13	-2.06E+06	2.06E+06
ENSG00000156873	PHKG2	0.56	-1.80	-0.85	7.66E-13	-2.06E+06	2.06E+06
ENSG00000100292	HMOX1	1.86	1.86	0.89	8.18E-13	2.05E+06	2.05E+06
ENSG00000162591	MEGF6	1.80	1.80	0.85	7.74E-13	2.05E+06	2.05E+06
ENSG00000140265	ZSCAN29	1.71	1.71	0.77	7.13E-13	2.03E+06	2.03E+06
ENSG00000096060	FKBP5	0.57	-1.76	-0.81	7.56E-13	-2.02E+06	2.02E+06
ENSG00000072840	EVC	1.70	1.70	0.76	7.14E-13	2.01E+06	2.01E+06
ENSG00000171105	INSR	1.76	1.76	0.82	7.80E-13	1.99E+06	1.99E+06
ENSG00000141349	G6PC3	0.54	-1.86	-0.90	8.77E-13	-1.99E+06	1.99E+06
ENSG00000124225	PMEPA1	1.73	1.73	0.79	7.59E-13	1.98E+06	1.98E+06
ENSG00000139160	METTL20	1.92	1.92	0.94	9.59E-13	1.96E+06	1.96E+06
ENSG00000189184	PCDH18	0.45	-2.22	-1.15	1.29E-12	-1.95E+06	1.95E+06
ENSG00000110218	PANX1	1.72	1.72	0.78	7.78E-13	1.95E+06	1.95E+06
ENSG00000103260	METRN	0.50	-2.02	-1.01	1.10E-12	-1.92E+06	1.92E+06
ENSG00000128641	MYO1B	1.80	1.80	0.85	9.05E-13	1.90E+06	1.90E+06
ENSG00000179144	GIMAP7	0.58	-1.73	-0.79	8.48E-13	-1.88E+06	1.88E+06
ENSG00000106404	CLDN15	2.61	2.61	1.38	1.98E-12	1.85E+06	1.85E+06
ENSG00000183431	SF3A3	0.57	-1.74	-0.80	9.07E-13	-1.83E+06	1.83E+06
ENSG00000188522	FAM83G	1.72	1.72	0.78	8.83E-13	1.83E+06	1.83E+06
ENSG00000145912	NHP2	0.58	-1.73	-0.79	9.24E-13	-1.80E+06	1.80E+06
ENSG00000152904	GGPS1	1.77	1.77	0.83	1.01E-12	1.76E+06	1.76E+06
ENSG00000162804	SNED1	1.82	1.82	0.86	1.08E-12	1.75E+06	1.75E+06
ENSG00000172534	HCFC1	1.88	1.88	0.91	1.22E-12	1.70E+06	1.70E+06
ENSG00000081307	UBA5	0.56	-1.78	-0.84	1.10E-12	-1.70E+06	1.70E+06
ENSG00000276043	UHRF1	0.55	-1.82	-0.86	1.15E-12	-1.70E+06	1.70E+06
ENSG00000136490	LIMD2	0.56	-1.80	-0.85	1.13E-12	-1.69E+06	1.69E+06
ENSG00000084731	KIF3C	1.70	1.70	0.76	1.04E-12	1.66E+06	1.66E+06
ENSG00000161267	BDH1	0.49	-2.06	-1.04	1.55E-12	-1.65E+06	1.65E+06
ENSG00000065911	MTHFD2	0.57	-1.75	-0.81	1.13E-12	-1.65E+06	1.65E+06
ENSG00000129048	CCRL1	0.45	-2.20	-1.14	1.81E-12	-1.63E+06	1.63E+06
ENSG00000100288	CHKB	0.32	-3.16	-1.66	3.78E-12	-1.62E+06	1.62E+06
ENSG00000267855	NDUFA7	0.57	-1.76	-0.82	1.18E-12	-1.62E+06	1.62E+06
ENSG00000233765	AL591479.1	2.07	2.07	1.05	1.67E-12	1.60E+06	1.60E+06
ENSG00000104343	UBE2W	1.76	1.76	0.81	1.20E-12	1.60E+06	1.60E+06
ENSG00000092964	DPYSL2	1.89	1.89	0.92	1.44E-12	1.58E+06	1.58E+06
ENSG00000087074	PPP1R15A	1.80	1.80	0.85	1.32E-12	1.57E+06	1.57E+06
ENSG00000130449	ZSWIM6	1.72	1.72	0.78	1.24E-12	1.55E+06	1.55E+06
ENSG00000177606	JUN	1.87	1.87	0.90	1.48E-12	1.53E+06	1.53E+06
ENSG00000118515	SGK1	0.57	-1.76	-0.81	1.37E-12	-1.50E+06	1.50E+06
ENSG00000169105	CHST14	1.74	1.74	0.80	1.36E-12	1.50E+06	1.50E+06
ENSG00000134222	PSRC1	0.55	-1.82	-0.87	1.49E-12	-1.50E+06	1.50E+06
ENSG00000145439	CBR4	1.77	1.77	0.82	1.40E-12	1.49E+06	1.49E+06
ENSG00000049192	ADAMTS6	1.76	1.76	0.81	1.40E-12	1.49E+06	1.49E+06
ENSG00000137767	SQRDL	0.56	-1.77	-0.83	1.42E-12	-1.49E+06	1.49E+06
ENSG00000136560	TANK	1.96	1.96	0.97	1.76E-12	1.48E+06	1.48E+06
ENSG00000128159	TUBGCP6	0.58	-1.72	-0.78	1.42E-12	-1.44E+06	1.44E+06
ENSG00000171608	PIK3CD	2.14	2.14	1.10	2.20E-12	1.44E+06	1.44E+06
ENSG00000196576	PLXNB2	1.78	1.78	0.83	1.54E-12	1.44E+06	1.44E+06
ENSG00000138685	FGF2	0.55	-1.81	-0.86	1.62E-12	-1.42E+06	1.42E+06
ENSG00000159267	HLCS	1.71	1.71	0.78	1.55E-12	1.38E+06	1.38E+06
ENSG00000066427	ATXN3	0.59	-1.69	-0.76	1.59E-12	-1.34E+06	1.34E+06
ENSG00000197142	ACSL5	0.43	-2.33	-1.22	3.02E-12	-1.34E+06	1.34E+06
ENSG00000154920	EME1	0.51	-1.96	-0.97	2.16E-12	-1.34E+06	1.34E+06
ENSG00000163378	EOGT	0.58	-1.73	-0.79	1.73E-12	-1.32E+06	1.32E+06
ENSG00000134871	COL4A2	2.11	2.11	1.07	2.56E-12	1.32E+06	1.32E+06
ENSG00000167747	C19orf48	0.57	-1.77	-0.82	1.93E-12	-1.27E+06	1.27E+06
ENSG00000111801	BTN3A3	0.49	-2.06	-1.04	2.66E-12	-1.26E+06	1.26E+06
ENSG00000170113	NIPA1	1.92	1.92	0.94	2.40E-12	1.24E+06	1.24E+06
ENSG00000155085	AKD1	2.03	2.03	1.02	2.67E-12	1.24E+06	1.24E+06
ENSG00000128739	SNRPN	1.92	1.92	0.94	2.39E-12	1.24E+06	1.24E+06
ENSG00000119900	OGFRL1	1.69	1.69	0.76	1.88E-12	1.23E+06	1.23E+06
ENSG00000109743	BST1	0.53	-1.89	-0.92	2.36E-12	-1.23E+06	1.23E+06
ENSG00000171476	HOPX	1.92	1.92	0.94	2.43E-12	1.23E+06	1.23E+06
ENSG00000147852	VLDLR	1.97	1.97	0.98	2.57E-12	1.23E+06	1.23E+06
ENSG00000158106	RHPN1	0.54	-1.86	-0.89	2.31E-12	-1.22E+06	1.22E+06
ENSG00000259207	ITGB3	0.58	-1.72	-0.79	2.02E-12	-1.21E+06	1.21E+06
ENSG00000155657	TTN	2.40	2.40	1.27	4.02E-12	1.20E+06	1.20E+06
ENSG00000110900	TSPAN11	2.03	2.03	1.02	2.88E-12	1.20E+06	1.20E+06
ENSG00000108523	RNF167	0.59	-1.71	-0.77	2.06E-12	-1.19E+06	1.19E+06
ENSG00000232838	PET117	0.36	-2.76	-1.47	5.44E-12	-1.18E+06	1.18E+06
ENSG00000166886	NAB2	0.55	-1.81	-0.85	2.37E-12	-1.17E+06	1.17E+06
ENSG00000243364	EFNA4	0.51	-1.95	-0.97	2.82E-12	-1.16E+06	1.16E+06
ENSG00000143971	ETAA1	1.81	1.81	0.85	2.52E-12	1.14E+06	1.14E+06
ENSG00000135749	PCNXL2	1.84	1.84	0.88	2.65E-12	1.13E+06	1.13E+06
ENSG00000180257	ZNF816	2.02	2.02	1.01	3.23E-12	1.12E+06	1.12E+06
ENSG00000106012	IQCE	1.74	1.74	0.80	2.43E-12	1.12E+06	1.12E+06
ENSG00000144712	CAND2	0.52	-1.93	-0.95	3.04E-12	-1.11E+06	1.11E+06
ENSG00000169398	PTK2	1.79	1.79	0.84	2.64E-12	1.10E+06	1.10E+06

ENSG00000260552	RP11-49I11.1	3.26	3.26	1.70	8.85E-12	1.10E+06	1.10E+06
ENSG00000039650	PNKP	0.58	-1.73	-0.79	2.53E-12	-1.09E+06	1.09E+06
ENSG00000186787	SPIN2B	0.37	-2.70	-1.43	6.32E-12	-1.07E+06	1.07E+06
ENSG00000108788	MLX	0.57	-1.76	-0.82	2.72E-12	-1.07E+06	1.07E+06
ENSG00000126953	TIMM8A	0.48	-2.07	-1.05	3.97E-12	-1.04E+06	1.04E+06
ENSG00000005882	PDK2	0.55	-1.82	-0.86	3.13E-12	-1.03E+06	1.03E+06
ENSG00000159259	CHAF1B	0.55	-1.83	-0.87	3.21E-12	-1.02E+06	1.02E+06
ENSG00000100065	CARD10	1.68	1.68	0.75	2.92E-12	9.83E+05	9.83E+05
ENSG00000132932	ATP8A2	1.99	1.99	0.99	4.11E-12	9.80E+05	9.80E+05
ENSG00000131779	PEX11B	0.57	-1.76	-0.81	3.23E-12	-9.79E+05	9.79E+05
ENSG00000149554	CHEK1	0.59	-1.69	-0.76	3.00E-12	-9.75E+05	9.75E+05
ENSG00000184005	ST6GALNAC3	0.54	-1.85	-0.89	3.66E-12	-9.66E+05	9.66E+05
ENSG00000213213	KIAA1984	2.56	2.56	1.36	7.07E-12	9.63E+05	9.63E+05
ENSG00000131669	NINJ1	1.70	1.70	0.76	3.11E-12	9.62E+05	9.62E+05
ENSG00000106571	GLI3	1.89	1.89	0.92	3.90E-12	9.57E+05	9.57E+05
ENSG00000111897	SERINC1	0.57	-1.74	-0.80	3.38E-12	-9.45E+05	9.45E+05
ENSG00000186951	PPARA	1.86	1.86	0.90	3.90E-12	9.44E+05	9.44E+05
ENSG00000132481	TRIM47	1.73	1.73	0.79	3.39E-12	9.38E+05	9.38E+05
ENSG00000155324	GRAMD3	0.37	-2.70	-1.43	8.34E-12	-9.35E+05	9.35E+05
ENSG00000121152	NCAPH	0.58	-1.72	-0.78	3.44E-12	-9.27E+05	9.27E+05
ENSG00000179862	CITED4	2.23	2.23	1.16	5.98E-12	9.14E+05	9.14E+05
ENSG00000169991	IFFO2	1.72	1.72	0.78	3.56E-12	9.10E+05	9.10E+05
ENSG00000213339	QTRT1	0.57	-1.77	-0.82	3.78E-12	-9.09E+05	9.09E+05
ENSG00000196507	TCEAL3	0.52	-1.92	-0.94	4.47E-12	-9.08E+05	9.08E+05
ENSG00000174640	SLCO2A1	0.38	-2.64	-1.40	8.69E-12	-8.95E+05	8.95E+05
ENSG00000167083	GNGT2	2.21	2.21	1.14	6.17E-12	8.90E+05	8.90E+05
ENSG00000164045	CDC25A	0.59	-1.68	-0.75	3.58E-12	-8.89E+05	8.89E+05
ENSG00000050165	DKK3	1.82	1.82	0.86	4.18E-12	8.88E+05	8.88E+05
ENSG00000149150	SLC43A1	0.52	-1.91	-0.94	4.66E-12	-8.87E+05	8.87E+05
ENSG00000171729	TMEM51	1.78	1.78	0.83	4.07E-12	8.80E+05	8.80E+05
ENSG00000168591	TMUB2	0.60	-1.68	-0.75	3.83E-12	-8.58E+05	8.58E+05
ENSG00000118960	HS1BP3	1.73	1.73	0.79	4.09E-12	8.58E+05	8.58E+05
ENSG00000110536	PTPMT1	0.58	-1.73	-0.79	4.11E-12	-8.54E+05	8.54E+05
ENSG00000256269	HMBS	0.41	-2.42	-1.27	8.17E-12	-8.47E+05	8.47E+05
ENSG00000103241	FOXF1	1.86	1.86	0.90	5.18E-12	8.17E+05	8.17E+05
ENSG00000100767	PAPLN	0.29	-3.40	-1.77	1.74E-11	-8.15E+05	8.15E+05
ENSG00000162600	OMA1	1.81	1.81	0.86	5.01E-12	8.10E+05	8.10E+05
ENSG00000064652	SNX24	0.52	-1.91	-0.93	5.64E-12	-8.04E+05	8.04E+05
ENSG00000116663	FBXO6	0.38	-2.61	-1.38	1.08E-11	-7.93E+05	7.93E+05
ENSG00000122786	CALD1	1.86	1.86	0.90	5.62E-12	7.87E+05	7.87E+05
ENSG00000155592	ZKSCAN2	1.74	1.74	0.80	4.91E-12	7.86E+05	7.86E+05
ENSG00000089091	DZANK1	2.36	2.36	1.24	9.26E-12	7.76E+05	7.76E+05
ENSG00000130940	CASZ1	2.04	2.04	1.03	6.98E-12	7.72E+05	7.72E+05
ENSG00000095303	PTGS1	0.51	-1.96	-0.97	6.46E-12	-7.71E+05	7.71E+05
ENSG00000072210	ALDH3A2	0.59	-1.70	-0.77	4.92E-12	-7.67E+05	7.67E+05
ENSG00000100234	TIMP3	0.55	-1.82	-0.87	5.67E-12	-7.66E+05	7.66E+05
ENSG00000179456	ZNF238	0.60	-1.68	-0.75	4.88E-12	-7.61E+05	7.61E+05
ENSG00000144827	ABHD10	0.60	-1.68	-0.75	4.91E-12	-7.58E+05	7.58E+05
ENSG00000139546	TARBP2	0.60	-1.67	-0.74	4.94E-12	-7.51E+05	7.51E+05
ENSG00000161040	FBXL13	1.89	1.89	0.92	6.33E-12	7.50E+05	7.50E+05
ENSG00000169432	SCN9A	0.50	-2.00	-1.00	7.19E-12	-7.47E+05	7.47E+05
ENSG00000188486	H2AFX	0.58	-1.72	-0.78	5.39E-12	-7.40E+05	7.40E+05
ENSG00000177666	PNPLA2	0.53	-1.87	-0.91	6.44E-12	-7.39E+05	7.39E+05
ENSG00000141867	BRD4	1.81	1.81	0.85	6.06E-12	7.34E+05	7.34E+05
ENSG00000118523	CTGF	0.58	-1.73	-0.79	5.78E-12	-7.20E+05	7.20E+05
ENSG00000174206	C12orf66	2.03	2.03	1.02	7.95E-12	7.19E+05	7.19E+05
ENSG00000144655	CSRNP1	1.69	1.69	0.75	5.54E-12	7.16E+05	7.16E+05
ENSG00000148730	EIF4EBP2	1.75	1.75	0.81	6.04E-12	7.14E+05	7.14E+05
ENSG00000206530	WDR52	2.17	2.17	1.12	9.38E-12	7.10E+05	7.10E+05
ENSG00000133597	ADCK2	1.72	1.72	0.78	6.15E-12	6.94E+05	6.94E+05
ENSG00000185808	PIGP	1.79	1.79	0.84	6.72E-12	6.91E+05	6.91E+05
ENSG00000087152	ATXN7L3	1.70	1.70	0.77	6.52E-12	6.67E+05	6.67E+05
ENSG00000026103	FAS	1.78	1.78	0.83	7.16E-12	6.66E+05	6.66E+05
ENSG00000119280	C1orf198	1.69	1.69	0.76	7.05E-12	6.38E+05	6.38E+05
ENSG00000165912	PACSIN3	0.56	-1.77	-0.83	7.98E-12	-6.28E+05	6.28E+05
ENSG00000137070	IL11RA	2.01	2.01	1.01	1.03E-11	6.28E+05	6.28E+05
ENSG00000140564	FURIN	1.74	1.74	0.80	7.68E-12	6.27E+05	6.27E+05
ENSG00000183323	CCDC125	0.56	-1.78	-0.83	8.21E-12	-6.22E+05	6.22E+05
ENSG00000116199	FAM20B	0.58	-1.72	-0.78	7.67E-12	-6.22E+05	6.22E+05
ENSG00000132004	FBXW9	0.53	-1.88	-0.91	9.15E-12	-6.20E+05	6.20E+05
ENSG00000142634	EFHD2	1.72	1.72	0.79	7.73E-12	6.20E+05	6.20E+05
ENSG00000035499	DEPDC1B	0.59	-1.71	-0.77	7.58E-12	-6.20E+05	6.20E+05
ENSG00000256632	PXMP2	0.56	-1.79	-0.84	8.66E-12	-6.08E+05	6.08E+05
ENSG00000258738	RP11-73E17.2	2.05	2.05	1.04	1.15E-11	6.05E+05	6.05E+05
ENSG00000184292	TACSTD2	1.70	1.70	0.76	7.94E-12	6.02E+05	6.02E+05
ENSG00000249673	NOP14-AS1	0.35	-2.84	-1.51	2.25E-11	-5.99E+05	5.99E+05
ENSG00000115392	FANCL	0.58	-1.71	-0.77	8.21E-12	-5.97E+05	5.97E+05
ENSG00000106610	STAG3L4	0.48	-2.08	-1.05	1.22E-11	-5.95E+05	5.95E+05
ENSG00000158552	ZFAND2B	0.56	-1.79	-0.84	9.22E-12	-5.91E+05	5.91E+05

ENSG00000143977	SNRPG	0.52	-1.91	-0.93	1.05E-11	-5.89E+05	5.89E+05
ENSG00000160703	NLRX1	1.91	1.91	0.94	1.05E-11	5.89E+05	5.89E+05
ENSG00000176485	PLA2G16	0.60	-1.67	-0.74	8.03E-12	-5.88E+05	5.88E+05
ENSG00000141736	ERBB2	0.55	-1.81	-0.86	9.75E-12	-5.81E+05	5.81E+05
ENSG00000258376	RP4-647C14.2	0.39	-2.59	-1.37	2.07E-11	-5.69E+05	5.69E+05
ENSG00000172071	EIF2AK3	1.66	1.66	0.73	8.72E-12	5.62E+05	5.62E+05
ENSG00000119522	DENND1A	0.58	-1.72	-0.78	9.42E-12	-5.60E+05	5.60E+05
ENSG00000168685	IL7R	0.37	-2.71	-1.44	2.37E-11	-5.57E+05	5.57E+05
ENSG00000058668	ATP2B4	1.73	1.73	0.79	9.74E-12	5.54E+05	5.54E+05
ENSG00000161692	DBF4B	0.52	-1.93	-0.95	1.24E-11	-5.49E+05	5.49E+05
ENSG00000136463	TACO1	0.60	-1.66	-0.73	9.37E-12	-5.42E+05	5.42E+05
ENSG00000216937	C10orf68	2.71	2.71	1.44	2.55E-11	5.37E+05	5.37E+05
ENSG00000054793	ATP9A	1.71	1.71	0.77	1.04E-11	5.30E+05	5.30E+05
ENSG00000139613	SMARCC2	0.58	-1.73	-0.79	1.10E-11	-5.21E+05	5.21E+05
ENSG00000197885	NKIRAS1	0.51	-1.96	-0.97	1.43E-11	-5.18E+05	5.18E+05
ENSG00000127328	RAB3IP	0.61	-1.65	-0.72	1.07E-11	-5.03E+05	5.03E+05
ENSG00000183258	DDX41	0.60	-1.68	-0.75	1.11E-11	-5.03E+05	5.03E+05
ENSG00000183955	SETD8	1.72	1.72	0.78	1.19E-11	4.98E+05	4.98E+05
ENSG00000120314	WDR55	0.59	-1.68	-0.75	1.15E-11	-4.96E+05	4.96E+05
ENSG00000119718	EIF2B2	1.69	1.69	0.76	1.19E-11	4.90E+05	4.90E+05
ENSG00000140905	GCSH	0.58	-1.72	-0.78	1.25E-11	-4.86E+05	4.86E+05
ENSG00000135636	DYSF	1.74	1.74	0.80	1.31E-11	4.81E+05	4.81E+05
ENSG00000187513	GJA4	0.38	-2.61	-1.38	2.97E-11	-4.78E+05	4.78E+05
ENSG00000239900	ADSL	0.59	-1.69	-0.76	1.26E-11	-4.76E+05	4.76E+05
ENSG00000181924	COA4	0.60	-1.66	-0.73	1.25E-11	-4.70E+05	4.70E+05
ENSG00000078674	PCM1	0.37	-2.69	-1.43	3.30E-11	-4.68E+05	4.68E+05
ENSG00000265681	RPL17	0.50	-2.00	-1.00	1.82E-11	-4.68E+05	4.68E+05
ENSG00000120699	EXOSC8	0.59	-1.68	-0.75	1.35E-11	-4.59E+05	4.59E+05
ENSG00000168101	NUDT16L1	0.57	-1.76	-0.82	1.51E-11	-4.52E+05	4.52E+05
ENSG00000138617	PARP16	1.80	1.80	0.84	1.58E-11	4.52E+05	4.52E+05
ENSG00000137094	DNAJB5	0.55	-1.82	-0.87	1.64E-11	-4.50E+05	4.50E+05
ENSG00000100034	PPM1F	1.71	1.71	0.77	1.47E-11	4.46E+05	4.46E+05
ENSG00000197385	ZNF860	2.27	2.27	1.18	2.79E-11	4.29E+05	4.29E+05
ENSG00000108551	RASD1	2.56	2.56	1.36	3.57E-11	4.29E+05	4.29E+05
ENSG00000168542	COL3A1	0.44	-2.27	-1.18	2.82E-11	-4.28E+05	4.28E+05
	RP5-995J12.2	2.17	2.17	1.12	2.58E-11	4.27E+05	4.27E+05
ENSG00000100577	GSTZ1	1.81	1.81	0.86	1.81E-11	4.25E+05	4.25E+05
ENSG00000173786	CNP	0.58	-1.71	-0.77	1.64E-11	-4.22E+05	4.22E+05
ENSG00000135617	PRADC1	0.57	-1.75	-0.81	1.73E-11	-4.20E+05	4.20E+05
ENSG00000186591	UBE2H	1.70	1.70	0.77	1.65E-11	4.20E+05	4.20E+05
ENSG00000096433	ITPR3	1.95	1.95	0.97	2.20E-11	4.17E+05	4.17E+05
ENSG00000189007	ADAT2	0.54	-1.87	-0.90	2.02E-11	-4.15E+05	4.15E+05
ENSG00000215375	MYL5	1.80	1.80	0.85	1.89E-11	4.14E+05	4.14E+05
ENSG00000010818	HIVEP2	1.79	1.79	0.84	1.87E-11	4.14E+05	4.14E+05
ENSG00000253552	HOXA-AS2	1.87	1.87	0.90	2.08E-11	4.10E+05	4.10E+05
ENSG00000080845	DLGAP4	1.68	1.68	0.75	1.69E-11	4.09E+05	4.09E+05
ENSG00000128228	SDF2L1	0.53	-1.88	-0.91	2.13E-11	-4.07E+05	4.07E+05
ENSG00000235106	LINC00094	0.57	-1.75	-0.81	1.90E-11	-4.01E+05	4.01E+05
ENSG00000073331	ALPK1	0.48	-2.10	-1.07	2.80E-11	-3.98E+05	3.98E+05
ENSG00000026950	BTN3A1	0.57	-1.75	-0.81	1.96E-11	-3.96E+05	3.96E+05
ENSG00000176907	C8orf4	2.17	2.17	1.12	3.03E-11	3.93E+05	3.93E+05
ENSG00000148180	GSN	1.71	1.71	0.77	1.90E-11	3.91E+05	3.91E+05
ENSG00000204267	TAP2	0.60	-1.65	-0.73	1.79E-11	-3.91E+05	3.91E+05
	RP11-134G8.8	2.70	2.70	1.43	4.76E-11	3.91E+05	3.91E+05
ENSG00000189057	FAM111B	0.55	-1.83	-0.87	2.20E-11	-3.90E+05	3.90E+05
ENSG00000182134	TDRKH	0.52	-1.92	-0.94	2.44E-11	-3.89E+05	3.89E+05
ENSG00000185324	CDK10	0.60	-1.67	-0.74	1.92E-11	-3.81E+05	3.81E+05
ENSG00000010626	LRRC23	2.24	2.24	1.16	3.57E-11	3.75E+05	3.75E+05
ENSG00000074935	TUBE1	0.58	-1.73	-0.79	2.12E-11	-3.75E+05	3.75E+05
ENSG00000168528	SERINC2	2.66	2.66	1.41	5.06E-11	3.74E+05	3.74E+05
ENSG00000161013	MGAT4B	1.75	1.75	0.81	2.18E-11	3.74E+05	3.74E+05
ENSG00000205133	TRIQK	1.87	1.87	0.90	2.74E-11	3.57E+05	3.57E+05
ENSG00000197530	MIB2	1.71	1.71	0.78	2.33E-11	3.55E+05	3.55E+05
ENSG00000184900	SUMO3	0.59	-1.69	-0.76	2.32E-11	-3.51E+05	3.51E+05
ENSG00000164172	MOCS2	0.59	-1.69	-0.75	2.35E-11	-3.48E+05	3.48E+05
ENSG00000169738	DCXR	0.53	-1.89	-0.92	3.03E-11	-3.44E+05	3.44E+05
ENSG00000196678	ERI2	1.75	1.75	0.81	2.62E-11	3.41E+05	3.41E+05
ENSG00000255717	SNHG1	0.59	-1.69	-0.76	2.46E-11	-3.41E+05	3.41E+05
ENSG00000155729	KCTD18	1.70	1.70	0.76	2.49E-11	3.40E+05	3.40E+05
ENSG00000100023	PPIL2	0.61	-1.64	-0.71	2.34E-11	-3.39E+05	3.39E+05
ENSG00000158301	GPRASP2	0.56	-1.79	-0.84	2.89E-11	-3.33E+05	3.33E+05
ENSG00000113595	TRIM23	1.88	1.88	0.91	3.21E-11	3.32E+05	3.32E+05
ENSG00000109323	MANBA	0.54	-1.86	-0.90	3.17E-11	-3.31E+05	3.31E+05
ENSG00000134243	SORT1	0.52	-1.93	-0.94	3.39E-11	-3.31E+05	3.31E+05
	CTA-217C2.1	1.83	1.83	0.87	3.09E-11	3.29E+05	3.29E+05
ENSG00000111641	NOP2	0.61	-1.63	-0.70	2.58E-11	-3.21E+05	3.21E+05
ENSG00000140937	CDH11	0.49	-2.04	-1.03	4.09E-11	-3.18E+05	3.18E+05
ENSG00000136068	FLNB	2.01	2.01	1.01	4.03E-11	3.16E+05	3.16E+05
ENSG00000143575	HAX1	0.60	-1.66	-0.73	2.79E-11	-3.14E+05	3.14E+05

ENSG00000180573	HIST1H2AC	0.58	-1.71	-0.77	2.99E-11	-3.13E+05	3.13E+05
ENSG00000143793	C1orf35	0.57	-1.74	-0.80	3.13E-11	-3.11E+05	3.11E+05
ENSG00000137880	GCHFR	2.01	2.01	1.01	4.19E-11	3.11E+05	3.11E+05
ENSG00000116833	NR5A2	0.61	-1.64	-0.71	2.95E-11	-3.02E+05	3.02E+05
ENSG00000165757	KIAA1462	1.69	1.69	0.76	3.14E-11	3.01E+05	3.01E+05
ENSG00000162511	LAPTM5	1.63	1.63	0.70	2.92E-11	3.01E+05	3.01E+05
ENSG00000254912	RP11-632K20.2	2.54	2.54	1.35	7.25E-11	2.99E+05	2.99E+05
ENSG00000145908	ZNF300	0.59	-1.69	-0.76	3.29E-11	-2.95E+05	2.95E+05
ENSG00000153551	CMTM7	1.64	1.64	0.72	3.14E-11	2.93E+05	2.93E+05
ENSG00000259802	CTD-2256P15.2	2.54	2.54	1.35	7.72E-11	2.89E+05	2.89E+05
ENSG00000105556	MIER2	1.67	1.67	0.74	3.39E-11	2.87E+05	2.87E+05
ENSG00000144369	FAM171B	0.53	-1.88	-0.91	4.30E-11	-2.86E+05	2.86E+05
ENSG00000183873	SCN5A	1.78	1.78	0.83	3.88E-11	2.86E+05	2.86E+05
ENSG00000220785	MTMR9LP	0.49	-2.05	-1.03	5.16E-11	-2.85E+05	2.85E+05
ENSG00000136010	ALDH1L2	0.43	-2.34	-1.22	6.77E-11	-2.84E+05	2.84E+05
ENSG00000130270	ATP8B3	2.65	2.65	1.41	8.86E-11	2.82E+05	2.82E+05
ENSG00000149257	SERPINH1	0.58	-1.72	-0.79	3.76E-11	-2.81E+05	2.81E+05
ENSG00000226711	FAM66C	2.51	2.51	1.33	7.94E-11	2.81E+05	2.81E+05
ENSG00000081791	KIAA0141	0.61	-1.64	-0.72	3.42E-11	-2.81E+05	2.81E+05
ENSG00000215788	TNFRSF25	1.86	1.86	0.89	4.51E-11	2.77E+05	2.77E+05
ENSG00000196586	MYO6	1.69	1.69	0.76	3.75E-11	2.76E+05	2.76E+05
ENSG00000146374	RSPO3	0.31	-3.18	-1.67	1.35E-10	-2.74E+05	2.74E+05
ENSG00000176108	CHMP6	0.58	-1.73	-0.79	4.04E-11	-2.72E+05	2.72E+05
ENSG00000113504	SLC12A7	1.68	1.68	0.75	3.87E-11	2.71E+05	2.71E+05
ENSG00000178802	MPI	0.59	-1.69	-0.76	3.97E-11	-2.68E+05	2.68E+05
ENSG00000123159	GIPC1	0.60	-1.66	-0.73	3.94E-11	-2.64E+05	2.64E+05
ENSG00000141959	PFKL	0.57	-1.77	-0.82	4.50E-11	-2.63E+05	2.63E+05
ENSG00000120318	ARAP3	1.88	1.88	0.91	5.13E-11	2.63E+05	2.63E+05
ENSG00000213699	C2orf18	0.60	-1.66	-0.73	4.04E-11	-2.61E+05	2.61E+05
ENSG00000085871	MGST2	1.84	1.84	0.88	4.99E-11	2.60E+05	2.60E+05
ENSG00000141429	GALNT1	1.80	1.80	0.85	4.80E-11	2.60E+05	2.60E+05
ENSG00000133119	RFC3	0.61	-1.64	-0.71	4.00E-11	-2.59E+05	2.59E+05
ENSG00000099256	PRTFDC1	0.60	-1.66	-0.73	4.22E-11	-2.55E+05	2.55E+05
ENSG00000161940	BCL6B	1.68	1.68	0.75	4.34E-11	2.55E+05	2.55E+05
ENSG00000164251	F2RL1	1.70	1.70	0.77	4.52E-11	2.53E+05	2.53E+05
ENSG00000012822	CALCOCO1	1.65	1.65	0.72	4.27E-11	2.53E+05	2.53E+05
ENSG00000132423	COQ3	0.52	-1.94	-0.96	6.38E-11	-2.43E+05	2.43E+05
ENSG00000147403	RPL10	0.57	-1.76	-0.82	5.35E-11	-2.41E+05	2.41E+05
ENSG00000204227	RING1	0.54	-1.85	-0.89	6.00E-11	-2.39E+05	2.39E+05
ENSG00000109674	NEIL3	0.55	-1.82	-0.86	5.87E-11	-2.37E+05	2.37E+05
ENSG00000008394	MGST1	0.60	-1.66	-0.73	4.90E-11	-2.37E+05	2.37E+05
ENSG00000173914	RBM4B	0.57	-1.74	-0.80	5.46E-11	-2.36E+05	2.36E+05
ENSG00000154767	XPC	1.66	1.66	0.73	5.05E-11	2.34E+05	2.34E+05
ENSG00000168291	PDHB	0.61	-1.65	-0.72	5.10E-11	-2.31E+05	2.31E+05
ENSG00000119720	NRDE2	0.59	-1.69	-0.76	5.56E-11	-2.27E+05	2.27E+05
ENSG00000104147	OIP5	0.55	-1.80	-0.85	6.40E-11	-2.25E+05	2.25E+05
ENSG00000143341	HMCN1	0.53	-1.88	-0.91	7.07E-11	-2.24E+05	2.24E+05
ENSG00000115295	CLIP4	1.63	1.63	0.71	5.33E-11	2.23E+05	2.23E+05
ENSG00000033627	ATP6V0A1	1.71	1.71	0.77	5.89E-11	2.23E+05	2.23E+05
ENSG00000171388	APLN	1.69	1.69	0.75	5.77E-11	2.22E+05	2.22E+05
ENSG00000164970	FAM219A	1.64	1.64	0.71	5.53E-11	2.21E+05	2.21E+05
ENSG00000090020	SLC9A1	1.78	1.78	0.83	6.54E-11	2.21E+05	2.21E+05
ENSG00000164283	ESM1	1.73	1.73	0.79	6.23E-11	2.19E+05	2.19E+05
ENSG00000134480	CCNH	0.61	-1.65	-0.72	5.73E-11	-2.18E+05	2.18E+05
ENSG00000011478	QPCTL	0.54	-1.87	-0.90	7.40E-11	-2.17E+05	2.17E+05
ENSG00000144063	MALL	1.69	1.69	0.76	6.06E-11	2.17E+05	2.17E+05
ENSG00000085840	ORC1	0.56	-1.77	-0.82	6.68E-11	-2.17E+05	2.17E+05
ENSG00000112029	FBXO5	0.62	-1.63	-0.70	5.69E-11	-2.15E+05	2.15E+05
ENSG00000183011	NAA38	0.60	-1.66	-0.73	6.21E-11	-2.11E+05	2.11E+05
ENSG00000197557	TTC30A	1.73	1.73	0.79	6.82E-11	2.09E+05	2.09E+05
ENSG00000227354	RBM26-AS1	2.61	2.61	1.38	1.56E-10	2.09E+05	2.09E+05
	RP11-122A3.2	0.47	-2.13	-1.09	1.07E-10	-2.06E+05	2.06E+05
ENSG00000035664	DAPK2	2.37	2.37	1.24	1.32E-10	2.06E+05	2.06E+05
ENSG00000100479	POLE2	0.59	-1.70	-0.77	6.88E-11	-2.05E+05	2.05E+05
ENSG00000100285	NEFH	0.43	-2.33	-1.22	1.30E-10	-2.05E+05	2.05E+05
ENSG00000244005	NFS1	0.60	-1.68	-0.75	6.77E-11	-2.04E+05	2.04E+05
ENSG00000115159	GPD2	0.60	-1.65	-0.73	6.61E-11	-2.03E+05	2.03E+05
ENSG00000124006	OBSL1	1.72	1.72	0.78	7.15E-11	2.03E+05	2.03E+05
ENSG00000175115	PACS1	0.54	-1.84	-0.88	8.55E-11	-1.99E+05	1.99E+05
ENSG00000176871	WSB2	1.66	1.66	0.73	6.98E-11	1.98E+05	1.98E+05
ENSG00000119138	KLF9	0.56	-1.79	-0.84	8.26E-11	-1.97E+05	1.97E+05
ENSG00000162073	PAQR4	0.58	-1.72	-0.78	7.90E-11	-1.94E+05	1.94E+05
ENSG00000104213	PDGFRL	0.55	-1.82	-0.86	8.87E-11	-1.93E+05	1.93E+05
ENSG00000223745	RP4-717I23.3	2.13	2.13	1.09	1.24E-10	1.91E+05	1.91E+05
ENSG00000087086	FTL	0.59	-1.69	-0.76	7.89E-11	-1.91E+05	1.91E+05
ENSG00000185010	F8	2.27	2.27	1.19	1.43E-10	1.90E+05	1.90E+05
ENSG00000204569	PPP1R10	1.88	1.88	0.91	9.84E-11	1.89E+05	1.89E+05
ENSG00000276045	ORAI1	1.62	1.62	0.70	7.36E-11	1.89E+05	1.89E+05
ENSG00000116017	ARID3A	1.75	1.75	0.81	8.74E-11	1.88E+05	1.88E+05

ENSG00000003402	CFLAR	1.67	1.67	0.74	8.04E-11	1.86E+05	1.86E+05
ENSG00000125378	BMP4	0.60	-1.66	-0.73	7.99E-11	-1.85E+05	1.85E+05
ENSG00000154930	ACSS1	1.83	1.83	0.88	9.83E-11	1.85E+05	1.85E+05
ENSG00000170903	MSANTD4	1.66	1.66	0.73	8.51E-11	1.80E+05	1.80E+05
ENSG00000103415	HMOX2	1.68	1.68	0.75	9.26E-11	1.75E+05	1.75E+05
ENSG00000130005	GAMT	0.54	-1.85	-0.89	1.16E-10	-1.72E+05	1.72E+05
ENSG00000254837	AP001372.2	2.64	2.64	1.40	2.35E-10	1.72E+05	1.72E+05
ENSG00000163975	MFI2	2.65	2.65	1.41	2.39E-10	1.71E+05	1.71E+05
ENSG00000187609	EXD3	1.74	1.74	0.80	1.06E-10	1.69E+05	1.69E+05
ENSG00000167720	SRR	1.67	1.67	0.74	9.99E-11	1.67E+05	1.67E+05
ENSG00000116641	DOCK7	1.79	1.79	0.84	1.17E-10	1.65E+05	1.65E+05
ENSG00000161011	SQSTM1	1.67	1.67	0.74	1.03E-10	1.65E+05	1.65E+05
	RP11-169K16.9	1.98	1.98	0.98	1.46E-10	1.64E+05	1.64E+05
ENSG00000245552	RP11-712B9.2	3.16	3.16	1.66	3.73E-10	1.64E+05	1.64E+05
ENSG00000131773	KHDRBS3	1.69	1.69	0.75	1.07E-10	1.63E+05	1.63E+05
ENSG00000137720	C11orf1	1.82	1.82	0.87	1.25E-10	1.63E+05	1.63E+05
ENSG00000123485	HJURP	0.59	-1.68	-0.75	1.07E-10	-1.63E+05	1.63E+05
ENSG00000008324	SS18L2	0.60	-1.67	-0.74	1.06E-10	-1.62E+05	1.62E+05
ENSG00000234810	RP11-466L17.1	2.47	2.47	1.31	2.36E-10	1.61E+05	1.61E+05
ENSG00000092931	MFSD11	1.65	1.65	0.72	1.07E-10	1.60E+05	1.60E+05
ENSG00000270154	AC130352.1	1.93	1.93	0.95	1.48E-10	1.59E+05	1.59E+05
ENSG00000196151	WDSUB1	0.53	-1.90	-0.93	1.44E-10	-1.58E+05	1.58E+05
ENSG00000064225	ST3GAL6	1.68	1.68	0.75	1.13E-10	1.58E+05	1.58E+05
ENSG00000177628	GBA	1.63	1.63	0.70	1.10E-10	1.55E+05	1.55E+05
	RP4-668G5.1	2.37	2.37	1.25	2.34E-10	1.55E+05	1.55E+05
ENSG00000076356	PLXNA2	2.00	2.00	1.00	1.74E-10	1.52E+05	1.52E+05
ENSG00000079739	PGM1	0.58	-1.72	-0.78	1.29E-10	-1.51E+05	1.51E+05
ENSG00000142552	RCN3	0.57	-1.76	-0.82	1.36E-10	-1.51E+05	1.51E+05
ENSG00000268006	PTOV1-AS1	3.00	3.00	1.58	3.99E-10	1.50E+05	1.50E+05
ENSG00000231185	AC005592.2	2.63	2.63	1.39	3.07E-10	1.50E+05	1.50E+05
ENSG00000111247	RAD51AP1	0.59	-1.69	-0.76	1.28E-10	-1.49E+05	1.49E+05
ENSG00000085999	RAD54L	0.52	-1.93	-0.95	1.69E-10	-1.48E+05	1.48E+05
ENSG00000089154	GCN1L1	0.58	-1.74	-0.80	1.37E-10	-1.48E+05	1.48E+05
ENSG00000108828	VAT1	1.67	1.67	0.74	1.27E-10	1.48E+05	1.48E+05
ENSG00000178999	AURKB	0.56	-1.79	-0.84	1.47E-10	-1.47E+05	1.47E+05
ENSG00000128512	DOCK4	1.71	1.71	0.77	1.34E-10	1.47E+05	1.47E+05
ENSG00000256087	ZNF432	1.80	1.80	0.84	1.50E-10	1.46E+05	1.46E+05
ENSG00000168496	FEN1	0.61	-1.63	-0.71	1.25E-10	-1.46E+05	1.46E+05
ENSG00000101017	CD40	1.70	1.70	0.76	1.43E-10	1.42E+05	1.42E+05
ENSG00000125454	SLC25A19	0.57	-1.76	-0.81	1.54E-10	-1.41E+05	1.41E+05
ENSG00000231770	TMEM44-AS1	2.62	2.62	1.39	3.49E-10	1.40E+05	1.40E+05
ENSG00000188493	C19orf54	2.61	2.61	1.38	3.51E-10	1.39E+05	1.39E+05
ENSG00000136319	TTC5	0.58	-1.72	-0.78	1.54E-10	-1.38E+05	1.38E+05
ENSG00000140044	JDP2	0.36	-2.76	-1.46	4.01E-10	-1.38E+05	1.38E+05
ENSG00000110427	KIAA1549L	0.56	-1.79	-0.84	1.70E-10	-1.37E+05	1.37E+05
ENSG00000224870	RP4-758J18.2	0.52	-1.91	-0.93	1.93E-10	-1.37E+05	1.37E+05
ENSG00000184988	TMEM106A	0.52	-1.93	-0.95	1.99E-10	-1.37E+05	1.37E+05
ENSG00000130307	USHBP1	1.85	1.85	0.89	1.87E-10	1.35E+05	1.35E+05
ENSG00000125885	MCM8	0.61	-1.63	-0.71	1.50E-10	-1.33E+05	1.33E+05
ENSG00000225511	LINC00475	2.35	2.35	1.23	3.12E-10	1.33E+05	1.33E+05
ENSG00000157693	C9orf91	0.50	-2.01	-1.01	2.32E-10	-1.32E+05	1.32E+05
ENSG00000215421	ZNF407	1.62	1.62	0.70	1.50E-10	1.32E+05	1.32E+05
ENSG00000197417	SHPK	0.62	-1.60	-0.68	1.48E-10	-1.32E+05	1.32E+05
ENSG00000033867	SLC4A7	1.73	1.73	0.79	1.76E-10	1.31E+05	1.31E+05
ENSG00000132475	H3F3B	0.60	-1.67	-0.74	1.65E-10	-1.30E+05	1.30E+05
ENSG00000170464	DNAJC18	1.72	1.72	0.78	1.74E-10	1.30E+05	1.30E+05
ENSG00000171130	ATP6V0E2	0.61	-1.64	-0.72	1.67E-10	-1.27E+05	1.27E+05
ENSG00000124120	TTPAL	0.58	-1.72	-0.78	1.82E-10	-1.27E+05	1.27E+05
ENSG00000129657	SEC14L1	1.66	1.66	0.73	1.71E-10	1.27E+05	1.27E+05
ENSG00000145331	TRMT10A	1.86	1.86	0.90	2.17E-10	1.26E+05	1.26E+05
ENSG00000223501	VPS52	0.60	-1.67	-0.74	1.76E-10	-1.26E+05	1.26E+05
ENSG00000231205	ZNF826P	0.47	-2.15	-1.10	2.94E-10	-1.25E+05	1.25E+05
ENSG00000159363	ATP13A2	1.77	1.77	0.83	2.03E-10	1.25E+05	1.25E+05
ENSG00000109103	UNC119	0.62	-1.61	-0.69	1.70E-10	-1.24E+05	1.24E+05
ENSG00000122912	SLC25A16	0.57	-1.74	-0.80	2.01E-10	-1.23E+05	1.23E+05
ENSG00000157036	EXOG	0.57	-1.77	-0.82	2.06E-10	-1.23E+05	1.23E+05
ENSG00000131061	ZNF341	2.56	2.56	1.35	4.37E-10	1.22E+05	1.22E+05
ENSG00000093009	CDC45	0.60	-1.66	-0.73	1.86E-10	-1.22E+05	1.22E+05
ENSG00000164109	MAD2L1	0.62	-1.62	-0.70	1.83E-10	-1.20E+05	1.20E+05
ENSG00000158715	SLC45A3	1.66	1.66	0.73	1.95E-10	1.19E+05	1.19E+05
ENSG00000182095	TNRC18	1.63	1.63	0.70	1.88E-10	1.19E+05	1.19E+05
ENSG00000276170	AC124789.1	2.25	2.25	1.17	3.68E-10	1.17E+05	1.17E+05
ENSG00000129173	E2F8	0.60	-1.67	-0.74	2.03E-10	-1.17E+05	1.17E+05
ENSG00000170681	MURC	2.07	2.07	1.05	3.19E-10	1.16E+05	1.16E+05
ENSG00000131187	F12	2.40	2.40	1.26	4.29E-10	1.16E+05	1.16E+05
ENSG00000034677	RNF19A	1.77	1.77	0.82	2.37E-10	1.15E+05	1.15E+05
ENSG00000197713	RPE	0.62	-1.62	-0.69	1.99E-10	-1.15E+05	1.15E+05
ENSG00000162852	CNST	1.64	1.64	0.71	2.05E-10	1.14E+05	1.14E+05
ENSG00000123572	NRK	0.50	-1.98	-0.99	3.02E-10	-1.14E+05	1.14E+05

ENSG00000154188	ANGPT1	0.44	-2.29	-1.20	4.08E-10	-1.13E+05	1.13E+05
ENSG00000154127	UBASH3B	0.60	-1.68	-0.75	2.24E-10	-1.12E+05	1.12E+05
ENSG00000005302	MSL3	1.61	1.61	0.69	2.13E-10	1.11E+05	1.11E+05
ENSG00000174738	NR1D2	1.62	1.62	0.69	2.19E-10	1.09E+05	1.09E+05
ENSG00000234912	LINC00338	0.55	-1.82	-0.87	2.79E-10	-1.09E+05	1.09E+05
ENSG00000141378	PTRH2	0.62	-1.60	-0.68	2.18E-10	-1.09E+05	1.09E+05
ENSG00000160352	ZNF714	0.63	-1.59	-0.67	2.17E-10	-1.08E+05	1.08E+05
ENSG00000120071	KANSL1	1.60	1.60	0.68	2.22E-10	1.08E+05	1.08E+05
ENSG00000085721	RRN3	0.62	-1.61	-0.69	2.30E-10	-1.06E+05	1.06E+05
ENSG00000161395	PGAP3	0.46	-2.17	-1.12	4.19E-10	-1.06E+05	1.06E+05
ENSG00000250571	GLI4	2.03	2.03	1.02	3.68E-10	1.06E+05	1.06E+05
ENSG00000158042	MRPL17	0.60	-1.66	-0.73	2.66E-10	-1.01E+05	1.01E+05
ENSG00000108733	PEX12	1.72	1.72	0.78	3.00E-10	9.90E+04	9.90E+04
ENSG00000158710	TAGLN2	1.65	1.65	0.72	2.85E-10	9.76E+04	9.76E+04
ENSG00000135905	DOCK10	2.02	2.02	1.02	4.34E-10	9.71E+04	9.71E+04
ENSG00000090263	MRPS33	0.62	-1.63	-0.70	2.83E-10	-9.66E+04	9.66E+04
ENSG00000124733	MEA1	0.61	-1.64	-0.71	2.87E-10	-9.66E+04	9.66E+04
ENSG00000154917	RAB6B	0.49	-2.04	-1.03	4.49E-10	-9.62E+04	9.62E+04
ENSG00000126821	SGPP1	1.61	1.61	0.69	2.82E-10	9.59E+04	9.59E+04
ENSG00000108187	PBLD	1.83	1.83	0.87	3.69E-10	9.51E+04	9.51E+04
ENSG00000162654	GBP4	0.51	-1.97	-0.98	4.33E-10	-9.45E+04	9.45E+04
ENSG00000198853	RUSC2	1.61	1.61	0.69	2.94E-10	9.40E+04	9.40E+04
ENSG00000232098	CTD-2619J13.14	2.62	2.62	1.39	7.84E-10	9.34E+04	9.34E+04
ENSG00000166825	ANPEP	2.06	2.06	1.04	4.86E-10	9.34E+04	9.34E+04
ENSG00000179085	DPM3	0.52	-1.92	-0.94	4.22E-10	-9.33E+04	9.33E+04
ENSG00000260879	RP11-483I13.5	3.32	3.32	1.73	1.30E-09	9.22E+04	9.22E+04
ENSG00000165959	CLMN	1.74	1.74	0.80	3.57E-10	9.20E+04	9.20E+04
ENSG00000249992	TMEM158	2.02	2.02	1.01	4.81E-10	9.20E+04	9.20E+04
ENSG00000138835	RGS3	1.65	1.65	0.72	3.31E-10	9.06E+04	9.06E+04
ENSG00000104231	ZFAND1	0.60	-1.68	-0.74	3.48E-10	-8.98E+04	8.98E+04
ENSG00000242622	RP11-18H7.1	2.38	2.38	1.25	7.07E-10	8.97E+04	8.97E+04
	RP5-874C20.3	0.61	-1.64	-0.71	3.38E-10	-8.92E+04	8.92E+04
ENSG00000140575	IQGAP1	1.83	1.83	0.87	4.22E-10	8.89E+04	8.89E+04
ENSG00000178057	NDUFAF3	0.63	-1.60	-0.68	3.24E-10	-8.88E+04	8.88E+04
ENSG00000117399	CDC20	0.53	-1.87	-0.90	4.48E-10	-8.84E+04	8.84E+04
ENSG00000164649	CDCA7L	0.60	-1.65	-0.73	3.53E-10	-8.80E+04	8.80E+04
ENSG00000184162	NR2C2AP	0.57	-1.77	-0.82	4.12E-10	-8.69E+04	8.69E+04
ENSG00000228998	RP11-697E2.7	1.69	1.69	0.76	3.80E-10	8.67E+04	8.67E+04
ENSG00000106123	EPHB6	0.47	-2.13	-1.09	6.10E-10	-8.63E+04	8.63E+04
ENSG00000274267	HIST1H3B	0.60	-1.66	-0.74	3.73E-10	-8.62E+04	8.62E+04
ENSG00000148399	WDR85	0.62	-1.61	-0.69	3.50E-10	-8.60E+04	8.60E+04
ENSG00000225216	AC007362.1	0.57	-1.77	-0.82	4.25E-10	-8.57E+04	8.57E+04
ENSG00000100422	CERK	1.64	1.64	0.72	3.76E-10	8.49E+04	8.49E+04
ENSG00000108064	TFAM	0.61	-1.64	-0.71	3.74E-10	-8.48E+04	8.48E+04
ENSG00000076248	UNG	0.63	-1.60	-0.68	3.57E-10	-8.46E+04	8.46E+04
	AL033532.1	1.66	1.66	0.74	3.88E-10	8.45E+04	8.45E+04
ENSG00000135926	TMBIM1	1.72	1.72	0.79	4.19E-10	8.42E+04	8.42E+04
ENSG00000100726	TELO2	0.59	-1.70	-0.76	4.12E-10	-8.36E+04	8.36E+04
ENSG00000164414	SLC35A1	0.59	-1.69	-0.76	4.12E-10	-8.33E+04	8.33E+04
ENSG00000149591	TAGLN	1.85	1.85	0.89	5.00E-10	8.26E+04	8.26E+04
ENSG00000181938	GINS3	0.61	-1.65	-0.72	4.09E-10	-8.15E+04	8.15E+04
ENSG00000123154	WDR83	0.55	-1.83	-0.88	5.08E-10	-8.14E+04	8.14E+04
ENSG00000073910	FRY	1.65	1.65	0.72	4.15E-10	8.09E+04	8.09E+04
ENSG00000118971	CCND2	1.77	1.77	0.83	4.80E-10	8.09E+04	8.09E+04
ENSG00000245498	RP11-677M14.7	2.27	2.27	1.19	8.13E-10	7.98E+04	7.98E+04
ENSG00000105976	MET	1.67	1.67	0.74	4.40E-10	7.96E+04	7.96E+04
ENSG00000212769	HMGN2P8	0.48	-2.08	-1.05	6.86E-10	-7.93E+04	7.93E+04
ENSG00000147065	MSN	1.71	1.71	0.77	4.89E-10	7.71E+04	7.71E+04
ENSG00000080546	SESN1	1.64	1.64	0.72	4.57E-10	7.69E+04	7.69E+04
ENSG00000083290	ULK2	1.60	1.60	0.68	4.38E-10	7.63E+04	7.63E+04
ENSG00000197008	ZNF138	0.54	-1.85	-0.89	5.91E-10	-7.62E+04	7.62E+04
ENSG00000086200	IPO11	1.65	1.65	0.73	4.70E-10	7.62E+04	7.62E+04
ENSG00000121797	CCRL2	1.83	1.83	0.87	5.84E-10	7.56E+04	7.56E+04
ENSG00000132906	CASP9	1.86	1.86	0.89	6.14E-10	7.49E+04	7.49E+04
ENSG00000106305	AIMP2	0.63	-1.59	-0.67	4.66E-10	-7.35E+04	7.35E+04
ENSG00000142871	CYR61	0.56	-1.78	-0.83	5.86E-10	-7.35E+04	7.35E+04
ENSG00000182240	BACE2	1.62	1.62	0.69	4.92E-10	7.30E+04	7.30E+04
ENSG00000149054	ZNF215	0.54	-1.86	-0.90	6.56E-10	-7.27E+04	7.27E+04
ENSG00000125630	POLR1B	0.63	-1.60	-0.67	5.10E-10	-7.07E+04	7.07E+04
ENSG00000166833	NAV2	1.79	1.79	0.84	6.42E-10	7.06E+04	7.06E+04
ENSG00000176896	TCEANC	2.28	2.28	1.19	1.05E-09	7.02E+04	7.02E+04
ENSG00000077152	UBE2T	0.62	-1.62	-0.69	5.39E-10	-6.97E+04	6.97E+04
ENSG00000102030	NAA10	0.60	-1.67	-0.74	5.77E-10	-6.97E+04	6.97E+04
ENSG00000152229	PSTPIP2	0.54	-1.86	-0.89	7.24E-10	-6.90E+04	6.90E+04
ENSG00000162434	JAK1	1.64	1.64	0.71	5.70E-10	6.86E+04	6.86E+04
ENSG00000140443	IGF1R	1.83	1.83	0.87	7.30E-10	6.78E+04	6.78E+04
ENSG00000101846	STS	1.66	1.66	0.73	6.01E-10	6.77E+04	6.77E+04
ENSG00000128284	APOL3	0.62	-1.60	-0.68	5.62E-10	-6.76E+04	6.76E+04
ENSG00000198252	STYX	0.61	-1.63	-0.71	5.97E-10	-6.69E+04	6.69E+04

ENSG00000067113	PPAP2A	0.59	-1.69	-0.76	6.52E-10	-6.62E+04	6.62E+04
ENSG00000247077	PGAM5	0.63	-1.58	-0.66	5.75E-10	-6.58E+04	6.58E+04
ENSG00000143543	JTB	0.61	-1.65	-0.72	6.29E-10	-6.57E+04	6.57E+04
ENSG00000134690	CDCA8	0.60	-1.67	-0.74	6.65E-10	-6.47E+04	6.47E+04
ENSG00000085831	TTC39A	0.37	-2.70	-1.43	1.75E-09	-6.47E+04	6.47E+04
ENSG00000167617	CDC42EP5	1.63	1.63	0.70	6.42E-10	6.42E+04	6.42E+04
ENSG00000145604	SKP2	0.61	-1.64	-0.71	6.59E-10	-6.39E+04	6.39E+04
ENSG00000203760	CENPW	0.62	-1.62	-0.69	6.51E-10	-6.33E+04	6.33E+04
ENSG00000159348	CYB5R1	0.63	-1.60	-0.68	6.42E-10	-6.31E+04	6.31E+04
ENSG00000173705	SUSD5	1.72	1.72	0.78	7.51E-10	6.26E+04	6.26E+04
ENSG00000135740	SLC9A5	2.02	2.02	1.01	1.08E-09	6.16E+04	6.16E+04
ENSG00000173535	TNFRSF10C	1.59	1.59	0.67	6.64E-10	6.15E+04	6.15E+04
ENSG00000149218	ENDOD1	0.63	-1.60	-0.68	6.79E-10	-6.14E+04	6.14E+04
ENSG00000108061	SHOC2	1.74	1.74	0.80	8.33E-10	6.04E+04	6.04E+04
ENSG00000197646	PDCD1LG2	0.62	-1.63	-0.70	7.29E-10	-6.02E+04	6.02E+04
ENSG00000137474	MYO7A	0.38	-2.63	-1.39	1.94E-09	-5.96E+04	5.96E+04
ENSG00000117461	PIK3R3	1.58	1.58	0.66	7.02E-10	5.96E+04	5.96E+04
ENSG00000161920	MED11	0.60	-1.66	-0.73	7.79E-10	-5.95E+04	5.95E+04
ENSG00000137124	ALDH1B1	0.64	-1.57	-0.65	6.96E-10	-5.95E+04	5.95E+04
ENSG00000147804	SLC39A4	0.39	-2.57	-1.36	1.91E-09	-5.87E+04	5.87E+04
ENSG00000087088	BAX	1.63	1.63	0.70	7.78E-10	5.84E+04	5.84E+04
ENSG00000152818	UTRN	2.01	2.01	1.01	1.19E-09	5.83E+04	5.83E+04
ENSG00000111725	PRKAB1	1.59	1.59	0.67	7.49E-10	5.81E+04	5.81E+04
ENSG00000173482	PTPRM	1.59	1.59	0.67	7.53E-10	5.80E+04	5.80E+04
ENSG00000103227	LMF1	1.94	1.94	0.96	1.13E-09	5.78E+04	5.78E+04
ENSG00000071127	WDR1	0.61	-1.63	-0.71	8.34E-10	-5.65E+04	5.65E+04
ENSG00000095713	CRTAC1	1.95	1.95	0.96	1.20E-09	5.64E+04	5.64E+04
ENSG00000172340	SUCLG2	0.63	-1.60	-0.67	8.02E-10	-5.63E+04	5.63E+04
ENSG00000162384	C1orf123	0.64	-1.57	-0.65	7.84E-10	-5.62E+04	5.62E+04
ENSG00000186105	LRRC70	0.51	-1.96	-0.97	1.25E-09	-5.56E+04	5.56E+04
ENSG00000235770	LINC00607	0.62	-1.62	-0.70	8.56E-10	-5.54E+04	5.54E+04
ENSG00000168297	PXK	0.62	-1.61	-0.69	8.55E-10	-5.52E+04	5.52E+04
ENSG00000156011	PSD3	1.58	1.58	0.66	8.21E-10	5.51E+04	5.51E+04
ENSG00000249859	PVT1	1.61	1.61	0.68	8.61E-10	5.48E+04	5.48E+04
ENSG00000105607	GCDH	0.61	-1.64	-0.72	9.01E-10	-5.47E+04	5.47E+04
ENSG00000127666	TICAM1	1.81	1.81	0.85	1.11E-09	5.41E+04	5.41E+04
ENSG00000148459	PDSS1	0.60	-1.67	-0.74	9.58E-10	-5.39E+04	5.39E+04
ENSG00000130881	LRP3	1.58	1.58	0.66	9.38E-10	5.16E+04	5.16E+04
ENSG00000099864	PALM	1.70	1.70	0.77	1.09E-09	5.15E+04	5.15E+04
ENSG00000196782	MAML3	1.64	1.64	0.72	1.02E-09	5.14E+04	5.14E+04
ENSG00000104881	PPP1R13L	1.93	1.93	0.95	1.41E-09	5.14E+04	5.14E+04
ENSG00000072954	TMEM38A	2.07	2.07	1.05	1.66E-09	5.08E+04	5.08E+04
ENSG00000166508	MCM7	0.61	-1.65	-0.72	1.06E-09	-5.07E+04	5.07E+04
ENSG00000102125	TAZ	1.66	1.66	0.73	1.07E-09	5.06E+04	5.06E+04
ENSG00000116062	MSH6	0.60	-1.67	-0.74	1.09E-09	-5.05E+04	5.05E+04
ENSG00000126005	MMP24-AS1	0.56	-1.80	-0.84	1.28E-09	-5.03E+04	5.03E+04
ENSG00000101361	NOP56	0.61	-1.63	-0.71	1.06E-09	-5.01E+04	5.01E+04
ENSG00000164038	SLC9B2	0.63	-1.59	-0.67	1.01E-09	-5.01E+04	5.01E+04
ENSG00000164171	ITGA2	1.63	1.63	0.70	1.06E-09	4.99E+04	4.99E+04
ENSG00000110031	LPXN	1.60	1.60	0.68	1.03E-09	4.99E+04	4.99E+04
ENSG00000187049	TMEM216	0.57	-1.74	-0.80	1.22E-09	-4.97E+04	4.97E+04
ENSG00000101353	C20orf132	2.41	2.41	1.27	2.36E-09	4.97E+04	4.97E+04
ENSG00000151276	MAGI1	1.62	1.62	0.70	1.08E-09	4.93E+04	4.93E+04
ENSG00000119446	RBM18	1.58	1.58	0.66	1.03E-09	4.91E+04	4.91E+04
ENSG00000162688	AGL	1.87	1.87	0.90	1.45E-09	4.90E+04	4.90E+04
ENSG00000115363	EVA1A	1.65	1.65	0.73	1.14E-09	4.89E+04	4.89E+04
ENSG00000133101	CCNA1	0.38	-2.60	-1.38	2.86E-09	-4.86E+04	4.86E+04
ENSG00000039523	FAM65A	1.60	1.60	0.68	1.09E-09	4.85E+04	4.85E+04
ENSG00000165097	KDM1B	0.53	-1.88	-0.91	1.53E-09	-4.80E+04	4.80E+04
ENSG00000063660	GPC1	1.73	1.73	0.79	1.30E-09	4.80E+04	4.80E+04
ENSG00000124104	SNX21	0.61	-1.63	-0.71	1.18E-09	-4.76E+04	4.76E+04
ENSG00000096093	EFHC1	1.61	1.61	0.69	1.16E-09	4.73E+04	4.73E+04
ENSG00000164985	PSIP1	0.62	-1.61	-0.69	1.17E-09	-4.72E+04	4.72E+04
ENSG00000074416	MGLL	1.72	1.72	0.79	1.34E-09	4.71E+04	4.71E+04
ENSG00000241058	NSUN6	1.68	1.68	0.75	1.28E-09	4.69E+04	4.69E+04
ENSG00000144182	LIPT1	0.52	-1.93	-0.95	1.70E-09	-4.69E+04	4.69E+04
ENSG00000219626	FAM228B	1.87	1.87	0.90	1.60E-09	4.68E+04	4.68E+04
ENSG00000119801	YPEL5	1.65	1.65	0.72	1.24E-09	4.67E+04	4.67E+04
ENSG00000251136	RP11-37B2.1	1.80	1.80	0.85	1.52E-09	4.61E+04	4.61E+04
ENSG00000196118	C16orf93	0.41	-2.45	-1.29	2.83E-09	-4.61E+04	4.61E+04
ENSG00000197467	COL13A1	0.63	-1.59	-0.67	1.20E-09	-4.59E+04	4.59E+04
ENSG00000103811	CTSH	0.38	-2.60	-1.38	3.32E-09	-4.52E+04	4.52E+04
ENSG00000135679	MDM2	1.63	1.63	0.71	1.33E-09	4.47E+04	4.47E+04
ENSG00000145386	CCNA2	0.60	-1.67	-0.74	1.40E-09	-4.46E+04	4.46E+04
ENSG00000159167	STC1	2.45	2.45	1.29	3.04E-09	4.45E+04	4.45E+04
ENSG00000234741	GAS5	0.60	-1.67	-0.74	1.41E-09	-4.44E+04	4.44E+04
ENSG00000205544	C17orf61	0.61	-1.63	-0.71	1.37E-09	-4.41E+04	4.41E+04
ENSG00000120256	LRP11	1.58	1.58	0.66	1.30E-09	4.38E+04	4.38E+04
ENSG00000157985	AGAP1	1.61	1.61	0.69	1.36E-09	4.37E+04	4.37E+04

ENSG00000177106	EPS8L2	2.26	2.26	1.18	2.69E-09	4.37E+04	4.37E+04
ENSG00000176853	FAM91A1	1.78	1.78	0.84	1.68E-09	4.36E+04	4.36E+04
ENSG00000111602	TIMELESS	0.64	-1.56	-0.64	1.30E-09	-4.33E+04	4.33E+04
ENSG00000165118	C9orf64	1.59	1.59	0.67	1.34E-09	4.33E+04	4.33E+04
ENSG00000205664	RP11-706O15.1	1.62	1.62	0.70	1.43E-09	4.29E+04	4.29E+04
ENSG00000116885	OSCP1	0.53	-1.88	-0.91	1.97E-09	-4.23E+04	4.23E+04
ENSG00000181885	CLDN7	0.56	-1.77	-0.83	1.81E-09	-4.16E+04	4.16E+04
ENSG00000169857	AVEN	0.63	-1.59	-0.67	1.50E-09	-4.10E+04	4.10E+04
ENSG00000123975	CKS2	0.62	-1.61	-0.69	1.56E-09	-4.07E+04	4.07E+04
ENSG00000169241	SLC50A1	0.60	-1.67	-0.74	1.69E-09	-4.05E+04	4.05E+04
ENSG00000183010	PYCR1	0.63	-1.58	-0.66	1.52E-09	-4.05E+04	4.05E+04
ENSG00000214293	RSBN1L-AS1	0.43	-2.30	-1.20	3.25E-09	-4.04E+04	4.04E+04
ENSG00000138448	ITGAV	1.61	1.61	0.69	1.60E-09	4.03E+04	4.03E+04
ENSG00000152661	GJA1	1.65	1.65	0.73	1.69E-09	4.03E+04	4.03E+04
ENSG00000246523	RP11-736K20.6	2.64	2.64	1.40	4.30E-09	4.02E+04	4.02E+04
ENSG00000151692	RNF144A	1.56	1.56	0.64	1.50E-09	4.01E+04	4.01E+04
ENSG00000156313	RPGR	0.57	-1.76	-0.81	1.95E-09	-3.98E+04	3.98E+04
ENSG00000233016	SNHG7	0.64	-1.56	-0.64	1.59E-09	-3.92E+04	3.92E+04
ENSG00000156711	MAPK13	0.52	-1.92	-0.94	2.46E-09	-3.87E+04	3.87E+04
ENSG00000244165	P2RY11	2.38	2.38	1.25	3.80E-09	3.86E+04	3.86E+04
ENSG00000038382	TRIO	1.63	1.63	0.70	1.78E-09	3.85E+04	3.85E+04
ENSG00000159314	ARHGAP27	1.58	1.58	0.66	1.69E-09	3.84E+04	3.84E+04
ENSG00000182372	CLN8	1.58	1.58	0.66	1.71E-09	3.83E+04	3.83E+04
ENSG00000234456	MAGI2-AS3	0.62	-1.62	-0.69	1.81E-09	-3.80E+04	3.80E+04
ENSG00000124772	CPNE5	1.73	1.73	0.79	2.12E-09	3.76E+04	3.76E+04
ENSG00000003989	SLC7A2	0.63	-1.60	-0.68	1.85E-09	-3.71E+04	3.71E+04
	AL353791.1	0.41	-2.45	-1.29	4.34E-09	-3.71E+04	3.71E+04
ENSG00000112679	DUSP22	1.60	1.60	0.67	1.87E-09	3.69E+04	3.69E+04
ENSG00000143507	DUSP10	2.39	2.39	1.26	4.26E-09	3.67E+04	3.67E+04
ENSG00000105676	ARMC6	0.64	-1.56	-0.64	1.83E-09	-3.66E+04	3.66E+04
ENSG00000168827	GFM1	1.74	1.74	0.80	2.27E-09	3.65E+04	3.65E+04
ENSG00000245910	SNHG6	0.57	-1.76	-0.82	2.36E-09	-3.62E+04	3.62E+04
ENSG00000152495	CAMK4	0.43	-2.34	-1.23	4.33E-09	-3.56E+04	3.56E+04
ENSG00000058799	YIPF1	1.59	1.59	0.67	2.00E-09	3.55E+04	3.55E+04
ENSG00000183605	SFXN4	0.60	-1.67	-0.74	2.27E-09	-3.50E+04	3.50E+04
ENSG00000105552	BCAT2	0.62	-1.62	-0.69	2.15E-09	-3.49E+04	3.49E+04
ENSG00000122884	P4HA1	0.61	-1.64	-0.71	2.22E-09	-3.48E+04	3.48E+04
ENSG00000130517	PGPEP1	1.58	1.58	0.66	2.10E-09	3.45E+04	3.45E+04
ENSG00000135842	FAM129A	0.54	-1.86	-0.90	2.96E-09	-3.43E+04	3.43E+04
ENSG00000169248	CXCL11	2.20	2.20	1.14	4.16E-09	3.41E+04	3.41E+04
ENSG00000163686	ABHD6	0.54	-1.84	-0.88	2.91E-09	-3.41E+04	3.41E+04
	RP11-66N24.4	2.27	2.27	1.18	4.47E-09	3.40E+04	3.40E+04
ENSG00000174928	C3orf33	0.48	-2.08	-1.06	3.77E-09	-3.39E+04	3.39E+04
ENSG00000230479	AP000695.1	2.04	2.04	1.03	3.60E-09	3.39E+04	3.39E+04
ENSG00000059122	FLYWCH1	1.59	1.59	0.67	2.20E-09	3.39E+04	3.39E+04
ENSG00000146112	PPP1R18	1.65	1.65	0.72	2.38E-09	3.38E+04	3.38E+04
ENSG00000088448	ANKRD10	1.57	1.57	0.65	2.21E-09	3.34E+04	3.34E+04
ENSG00000087157	PGS1	1.57	1.57	0.65	2.27E-09	3.30E+04	3.30E+04
ENSG00000171055	FEZ2	1.62	1.62	0.70	2.43E-09	3.30E+04	3.30E+04
ENSG00000002586	CD99	1.61	1.61	0.68	2.38E-09	3.29E+04	3.29E+04
ENSG00000140263	SORD	0.62	-1.61	-0.69	2.43E-09	-3.28E+04	3.28E+04
ENSG00000000460	C1orf112	0.64	-1.57	-0.65	2.31E-09	-3.28E+04	3.28E+04
ENSG00000095485	CWF19L1	0.64	-1.56	-0.64	2.27E-09	-3.27E+04	3.27E+04
ENSG00000128944	C15orf23	0.61	-1.63	-0.70	2.49E-09	-3.27E+04	3.27E+04
ENSG00000096092	TMEM14A	0.62	-1.60	-0.68	2.43E-09	-3.25E+04	3.25E+04
ENSG00000269858	EGLN2	0.63	-1.58	-0.66	2.38E-09	-3.24E+04	3.24E+04
ENSG00000112242	E2F3	1.57	1.57	0.65	2.36E-09	3.23E+04	3.23E+04
ENSG00000107263	RAPGEF1	1.59	1.59	0.67	2.51E-09	3.18E+04	3.18E+04
ENSG00000168028	RPSA	0.62	-1.62	-0.69	2.61E-09	-3.17E+04	3.17E+04
ENSG00000072422	RHOBTB1	1.60	1.60	0.68	2.60E-09	3.14E+04	3.14E+04
ENSG00000108826	MRPL27	0.63	-1.57	-0.66	2.53E-09	-3.13E+04	3.13E+04
ENSG00000116717	GADD45A	1.57	1.57	0.65	2.54E-09	3.12E+04	3.12E+04
ENSG00000135828	RNASEL	1.81	1.81	0.86	3.52E-09	3.05E+04	3.05E+04
ENSG00000073605	GSDMB	1.80	1.80	0.85	3.50E-09	3.05E+04	3.05E+04
ENSG00000107566	ERLIN1	0.63	-1.60	-0.68	2.78E-09	-3.03E+04	3.03E+04
ENSG00000261840	RP11-146F11.1	2.52	2.52	1.33	6.94E-09	3.02E+04	3.02E+04
ENSG00000106004	HOXA5	0.64	-1.57	-0.65	2.75E-09	-2.99E+04	2.99E+04
ENSG00000176597	B3GNT5	1.89	1.89	0.92	4.01E-09	2.99E+04	2.99E+04
ENSG00000119004	CYP20A1	0.64	-1.57	-0.65	2.80E-09	-2.97E+04	2.97E+04
ENSG00000083799	CYLD	1.57	1.57	0.65	2.81E-09	2.97E+04	2.97E+04
ENSG00000174840	PDE12	0.63	-1.58	-0.66	2.83E-09	-2.96E+04	2.96E+04
ENSG00000088682	COQ9	0.64	-1.57	-0.65	2.84E-09	-2.95E+04	2.95E+04
ENSG00000136811	ODF2	0.60	-1.66	-0.73	3.17E-09	-2.95E+04	2.95E+04
ENSG00000260912	RP11-363E7.4	0.57	-1.75	-0.81	3.54E-09	-2.95E+04	2.95E+04
ENSG00000134910	STT3A	0.62	-1.60	-0.68	2.97E-09	-2.94E+04	2.94E+04
ENSG00000071054	MAP4K4	1.71	1.71	0.77	3.41E-09	2.92E+04	2.92E+04
ENSG00000126767	ELK1	0.62	-1.60	-0.68	3.05E-09	-2.90E+04	2.90E+04
ENSG00000184271	POU6F1	1.75	1.75	0.81	3.65E-09	2.89E+04	2.89E+04
ENSG00000260920	RP1-228H13.5	2.42	2.42	1.28	7.07E-09	2.88E+04	2.88E+04

ENSG00000261824	LINC00662	1.65	1.65	0.72	3.31E-09	2.87E+04	2.87E+04
ENSG00000048392	RRM2B	1.77	1.77	0.83	3.84E-09	2.87E+04	2.87E+04
ENSG00000198816	ZNF358	1.57	1.57	0.65	3.01E-09	2.86E+04	2.86E+04
ENSG00000087586	AURKA	0.62	-1.62	-0.69	3.23E-09	-2.85E+04	2.85E+04
ENSG00000163900	TMEM41A	0.64	-1.57	-0.65	3.03E-09	-2.85E+04	2.85E+04
ENSG00000133731	IMPA1	0.60	-1.67	-0.74	3.55E-09	-2.80E+04	2.80E+04
ENSG00000198369	SPRED2	1.74	1.74	0.80	3.91E-09	2.78E+04	2.78E+04
ENSG00000136231	IGF2BP3	1.68	1.68	0.75	3.69E-09	2.76E+04	2.76E+04
ENSG00000132394	EEFSEC	0.50	-2.01	-1.01	5.30E-09	-2.76E+04	2.76E+04
ENSG00000182704	TSKU	1.60	1.60	0.68	3.49E-09	2.71E+04	2.71E+04
	RP11-395P17.3	2.27	2.27	1.18	7.06E-09	2.70E+04	2.70E+04
ENSG00000163751	CPA3	1.91	1.91	0.93	5.02E-09	2.69E+04	2.69E+04
ENSG00000074582	BCS1L	0.62	-1.62	-0.69	3.61E-09	-2.69E+04	2.69E+04
ENSG00000041880	PARP3	1.67	1.67	0.74	3.87E-09	2.69E+04	2.69E+04
ENSG00000128059	PPAT	0.63	-1.59	-0.67	3.52E-09	-2.68E+04	2.68E+04
ENSG00000136717	BIN1	1.61	1.61	0.69	3.62E-09	2.68E+04	2.68E+04
ENSG00000182318	ZSCAN22	1.75	1.75	0.81	4.34E-09	2.66E+04	2.66E+04
ENSG00000111203	ITFG2	0.59	-1.68	-0.75	4.05E-09	-2.64E+04	2.64E+04
ENSG00000185619	PCGF3	1.58	1.58	0.66	3.62E-09	2.62E+04	2.62E+04
ENSG00000051128	HOMER3	1.57	1.57	0.65	3.58E-09	2.62E+04	2.62E+04
ENSG00000177045	SIX5	1.66	1.66	0.73	4.03E-09	2.62E+04	2.62E+04
ENSG00000162819	BROX	1.61	1.61	0.69	3.81E-09	2.61E+04	2.61E+04
ENSG00000182378	PLCXD1	0.57	-1.77	-0.82	4.66E-09	-2.59E+04	2.59E+04
ENSG00000197746	PSAP	1.62	1.62	0.69	3.90E-09	2.59E+04	2.59E+04
ENSG00000148154	UGCG	1.61	1.61	0.68	3.88E-09	2.58E+04	2.58E+04
ENSG00000154839	SKA1	0.64	-1.56	-0.64	3.71E-09	-2.56E+04	2.56E+04
ENSG00000173065	FAM222B	1.76	1.76	0.82	4.92E-09	2.51E+04	2.51E+04
ENSG00000067177	PHKA1	0.62	-1.62	-0.69	4.21E-09	-2.49E+04	2.49E+04
ENSG00000092421	SEMA6A	0.39	-2.59	-1.37	1.09E-08	-2.49E+04	2.49E+04
ENSG00000180730	SHISA2	1.82	1.82	0.86	5.38E-09	2.48E+04	2.48E+04
ENSG00000087303	NID2	2.46	2.46	1.30	9.81E-09	2.48E+04	2.48E+04
ENSG00000075239	ACAT1	0.64	-1.57	-0.65	4.08E-09	-2.46E+04	2.46E+04
ENSG00000148187	MRRF	1.55	1.55	0.63	3.97E-09	2.45E+04	2.45E+04
ENSG00000153774	CFDP1	0.63	-1.58	-0.66	4.13E-09	-2.45E+04	2.45E+04
ENSG00000137337	MDC1	1.74	1.74	0.80	5.06E-09	2.45E+04	2.45E+04
ENSG00000120279	MYCT1	1.61	1.61	0.68	4.30E-09	2.45E+04	2.45E+04
ENSG00000146918	NCAPG2	0.62	-1.60	-0.68	4.42E-09	-2.41E+04	2.41E+04
ENSG00000148803	FUOM	0.56	-1.79	-0.84	5.63E-09	-2.39E+04	2.39E+04
ENSG00000196754	S100A2	2.30	2.30	1.20	9.33E-09	2.38E+04	2.38E+04
ENSG00000168309	FAM107A	0.60	-1.67	-0.74	4.89E-09	-2.38E+04	2.38E+04
ENSG00000124019	FAM124B	1.58	1.58	0.66	4.42E-09	2.38E+04	2.38E+04
ENSG00000169093	ASMTL	0.62	-1.62	-0.70	4.77E-09	-2.35E+04	2.35E+04
ENSG00000169083	AR	0.62	-1.61	-0.68	4.76E-09	-2.33E+04	2.33E+04
ENSG00000130147	SH3BP4	1.64	1.64	0.71	4.95E-09	2.33E+04	2.33E+04
ENSG00000147475	ERLIN2	1.58	1.58	0.66	4.63E-09	2.33E+04	2.33E+04
ENSG00000196597	ZNF782	1.75	1.75	0.81	5.69E-09	2.33E+04	2.33E+04
ENSG00000034533	ASTE1	0.59	-1.68	-0.75	5.30E-09	-2.31E+04	2.31E+04
ENSG00000124406	ATP8A1	0.54	-1.85	-0.89	6.54E-09	-2.29E+04	2.29E+04
	AC106722.1	2.14	2.14	1.10	8.83E-09	2.28E+04	2.28E+04
ENSG00000172586	CHCHD1	0.62	-1.61	-0.69	5.08E-09	-2.26E+04	2.26E+04
ENSG00000073111	MCM2	0.59	-1.69	-0.76	5.70E-09	-2.24E+04	2.24E+04
ENSG00000003096	KLHL13	0.65	-1.54	-0.62	4.82E-09	-2.22E+04	2.22E+04
ENSG00000166548	TK2	1.59	1.59	0.67	5.14E-09	2.21E+04	2.21E+04
ENSG00000106829	TLE4	1.56	1.56	0.64	4.96E-09	2.21E+04	2.21E+04
ENSG00000100027	YPEL1	0.48	-2.09	-1.07	8.97E-09	-2.21E+04	2.21E+04
ENSG00000198825	INPP5F	1.61	1.61	0.68	5.67E-09	2.13E+04	2.13E+04
	AC058791.2	1.82	1.82	0.86	7.36E-09	2.12E+04	2.12E+04
ENSG00000168724	DNAJC21	0.64	-1.56	-0.64	5.43E-09	-2.11E+04	2.11E+04
ENSG00000272412	Metazoa_SRP	0.48	-2.10	-1.07	9.91E-09	-2.11E+04	2.11E+04
ENSG00000185483	ROR1	1.63	1.63	0.71	6.03E-09	2.10E+04	2.10E+04
ENSG00000198589	LRBA	1.64	1.64	0.71	6.12E-09	2.09E+04	2.09E+04
ENSG00000046651	OFD1	1.56	1.56	0.64	5.68E-09	2.07E+04	2.07E+04
ENSG00000143842	SOX13	1.60	1.60	0.68	6.13E-09	2.05E+04	2.05E+04
ENSG00000130717	UCK1	0.63	-1.58	-0.66	6.02E-09	-2.04E+04	2.04E+04
ENSG00000163006	CCDC138	0.59	-1.69	-0.76	6.94E-09	-2.03E+04	2.03E+04
ENSG00000135929	CYP27A1	0.57	-1.76	-0.81	7.59E-09	-2.02E+04	2.02E+04
ENSG00000134698	EIF2C4	1.54	1.54	0.62	5.81E-09	2.01E+04	2.01E+04
ENSG00000108590	MED31	0.63	-1.59	-0.67	6.29E-09	-2.01E+04	2.01E+04
ENSG00000071242	RPS6KA2	1.58	1.58	0.66	6.18E-09	2.01E+04	2.01E+04
ENSG00000158467	AHCYL2	0.64	-1.55	-0.64	6.00E-09	-2.01E+04	2.01E+04
ENSG00000183161	FANCF	1.56	1.56	0.64	6.14E-09	1.99E+04	1.99E+04
ENSG00000157456	CCNB2	0.63	-1.58	-0.66	6.54E-09	-1.95E+04	1.95E+04
ENSG00000137547	MRPL15	1.94	1.94	0.96	1.00E-08	1.94E+04	1.94E+04
	RP11-10O17.2	1.90	1.90	0.93	9.71E-09	1.93E+04	1.93E+04
ENSG00000119938	PPP1R3C	0.49	-2.03	-1.02	1.13E-08	-1.90E+04	1.90E+04
ENSG00000170385	SLC30A1	1.62	1.62	0.70	7.30E-09	1.90E+04	1.90E+04
ENSG00000107882	SUFU	1.62	1.62	0.70	7.44E-09	1.88E+04	1.88E+04
ENSG00000257219	RP11-54A9.1	1.58	1.58	0.66	7.07E-09	1.88E+04	1.88E+04
ENSG00000157214	STEAP2	0.51	-1.95	-0.97	1.09E-08	-1.87E+04	1.87E+04

ENSG00000112406	HECA	1.56	1.56	0.65	7.16E-09	1.85E+04	1.85E+04
ENSG00000013297	CLDN11	0.61	-1.65	-0.72	7.97E-09	-1.84E+04	1.84E+04
ENSG00000054282	SDCCAG8	1.54	1.54	0.62	7.11E-09	1.82E+04	1.82E+04
ENSG00000106443	PHF14	1.58	1.58	0.66	7.50E-09	1.82E+04	1.82E+04
ENSG00000164136	IL15	0.50	-1.98	-0.99	1.26E-08	-1.77E+04	1.77E+04
ENSG00000151208	DLG5	1.61	1.61	0.69	8.44E-09	1.75E+04	1.75E+04
ENSG00000088305	DNMT3B	0.62	-1.61	-0.69	8.49E-09	-1.74E+04	1.74E+04
ENSG00000116353	MECR	0.61	-1.63	-0.71	8.85E-09	-1.73E+04	1.73E+04
ENSG00000170469	SPATA24	0.44	-2.30	-1.20	1.75E-08	-1.73E+04	1.73E+04
ENSG00000168405	CMAHP	0.43	-2.31	-1.21	1.79E-08	-1.72E+04	1.72E+04
ENSG00000102755	FLT1	1.57	1.57	0.65	8.45E-09	1.71E+04	1.71E+04
ENSG00000107554	DNMBP	1.68	1.68	0.75	9.83E-09	1.70E+04	1.70E+04
ENSG00000185737	NRG3	0.37	-2.73	-1.45	2.63E-08	-1.69E+04	1.69E+04
ENSG00000115946	PNO1	0.64	-1.57	-0.65	8.66E-09	-1.68E+04	1.68E+04
ENSG00000240771	ARHGEF25	1.78	1.78	0.83	1.12E-08	1.68E+04	1.68E+04
ENSG00000117676	RPS6KA1	0.60	-1.68	-0.75	9.95E-09	-1.68E+04	1.68E+04
ENSG00000015532	XYLT2	0.62	-1.61	-0.69	9.22E-09	-1.68E+04	1.68E+04
ENSG00000161204	ABCF3	0.64	-1.55	-0.63	8.59E-09	-1.67E+04	1.67E+04
ENSG00000162441	LZIC	1.54	1.54	0.62	8.56E-09	1.66E+04	1.66E+04
ENSG00000263327	TAPT1-AS1	2.22	2.22	1.15	1.80E-08	1.66E+04	1.66E+04
ENSG00000101842	VSIG1	1.75	1.75	0.80	1.14E-08	1.64E+04	1.64E+04
ENSG00000120889	TNFRSF10B	1.59	1.59	0.66	9.51E-09	1.63E+04	1.63E+04
ENSG00000213923	CSNK1E	1.57	1.57	0.65	9.33E-09	1.62E+04	1.62E+04
ENSG00000115415	STAT1	0.64	-1.57	-0.65	9.41E-09	-1.62E+04	1.62E+04
ENSG00000188153	COL4A5	0.62	-1.60	-0.68	9.88E-09	-1.61E+04	1.61E+04
ENSG00000254901	MEF2BNB	0.60	-1.65	-0.73	1.05E-08	-1.61E+04	1.61E+04
ENSG00000164167	LSM6	0.64	-1.56	-0.64	9.48E-09	-1.61E+04	1.61E+04
ENSG00000117394	SLC2A1	1.69	1.69	0.76	1.13E-08	1.59E+04	1.59E+04
ENSG00000126368	NR1D1	2.33	2.33	1.22	2.18E-08	1.57E+04	1.57E+04
ENSG00000048162	NOP16	0.65	-1.54	-0.62	9.73E-09	-1.56E+04	1.56E+04
ENSG00000167578	RAB4B	0.54	-1.86	-0.90	1.43E-08	-1.56E+04	1.56E+04
ENSG00000120860	CCDC53	1.54	1.54	0.62	1.01E-08	1.54E+04	1.54E+04
ENSG00000198689	SLC9A6	1.53	1.53	0.61	1.00E-08	1.53E+04	1.53E+04
ENSG00000100221	JOSD1	1.57	1.57	0.65	1.06E-08	1.53E+04	1.53E+04
ENSG00000186480	INSIG1	0.62	-1.62	-0.69	1.13E-08	-1.52E+04	1.52E+04
ENSG00000156603	MED19	0.63	-1.59	-0.67	1.10E-08	-1.51E+04	1.51E+04
ENSG00000121900	TMEM54	0.55	-1.82	-0.86	1.46E-08	-1.51E+04	1.51E+04
ENSG00000171791	BCL2	1.98	1.98	0.98	1.74E-08	1.50E+04	1.50E+04
ENSG00000130402	ACTN4	1.64	1.64	0.72	1.23E-08	1.48E+04	1.48E+04
ENSG00000074071	MRPS34	0.65	-1.53	-0.61	1.07E-08	-1.48E+04	1.48E+04
ENSG00000107872	FBXL15	0.58	-1.72	-0.78	1.36E-08	-1.47E+04	1.47E+04
ENSG00000274641	HIST1H2BO	0.62	-1.61	-0.69	1.20E-08	-1.47E+04	1.47E+04
ENSG00000094975	SUCO	1.64	1.64	0.71	1.25E-08	1.46E+04	1.46E+04
ENSG00000078246	TULP3	1.69	1.69	0.76	1.35E-08	1.46E+04	1.46E+04
ENSG00000105559	PLEKHA4	0.44	-2.26	-1.18	2.40E-08	-1.46E+04	1.46E+04
ENSG00000105767	CADM4	1.76	1.76	0.82	1.47E-08	1.45E+04	1.45E+04
ENSG00000075826	SEC31B	2.06	2.06	1.04	2.01E-08	1.45E+04	1.45E+04
ENSG00000142945	KIF2C	0.63	-1.59	-0.67	1.22E-08	-1.44E+04	1.44E+04
ENSG00000188641	DPYD	0.65	-1.53	-0.61	1.13E-08	-1.44E+04	1.44E+04
ENSG00000164442	CITED2	0.60	-1.68	-0.75	1.36E-08	-1.44E+04	1.44E+04
ENSG00000124191	TOX2	1.80	1.80	0.85	1.57E-08	1.43E+04	1.43E+04
ENSG00000186470	BTN3A2	0.65	-1.55	-0.63	1.16E-08	-1.43E+04	1.43E+04
ENSG00000142867	BCL10	1.57	1.57	0.65	1.20E-08	1.43E+04	1.43E+04
ENSG00000204351	SKIV2L	0.61	-1.64	-0.71	1.34E-08	-1.41E+04	1.41E+04
ENSG00000170542	SERPINB9	0.64	-1.55	-0.63	1.23E-08	-1.40E+04	1.40E+04
ENSG00000214263	RPSAP53	0.61	-1.64	-0.72	1.42E-08	-1.38E+04	1.38E+04
ENSG00000230630	DNM3OS	0.51	-1.95	-0.96	2.00E-08	-1.38E+04	1.38E+04
ENSG00000168310	IRF2	1.65	1.65	0.72	1.43E-08	1.38E+04	1.38E+04
ENSG00000164938	TP53INP1	1.60	1.60	0.68	1.37E-08	1.37E+04	1.37E+04
ENSG00000226137	BAIAP2-AS1	0.63	-1.60	-0.68	1.37E-08	-1.37E+04	1.37E+04
ENSG00000257167	TMPO-AS1	1.77	1.77	0.83	1.72E-08	1.35E+04	1.35E+04
ENSG00000176102	CSTF3	1.67	1.67	0.74	1.54E-08	1.35E+04	1.35E+04
ENSG00000135317	SNX14	0.64	-1.56	-0.64	1.34E-08	-1.34E+04	1.34E+04
ENSG00000182154	MRPL41	0.66	-1.52	-0.61	1.29E-08	-1.34E+04	1.34E+04
ENSG00000196083	IL1RAP	1.55	1.55	0.63	1.34E-08	1.34E+04	1.34E+04
ENSG00000047346	FAM214A	1.57	1.57	0.65	1.38E-08	1.34E+04	1.34E+04
ENSG00000112144	ICK	1.63	1.63	0.70	1.48E-08	1.34E+04	1.34E+04
ENSG00000101608	MYL12A	1.58	1.58	0.66	1.40E-08	1.33E+04	1.33E+04
ENSG00000018189	RUFY3	0.66	-1.52	-0.61	1.31E-08	-1.33E+04	1.33E+04
ENSG00000026508	CD44	1.61	1.61	0.69	1.47E-08	1.33E+04	1.33E+04
ENSG00000117448	AKR1A1	0.64	-1.55	-0.63	1.38E-08	-1.32E+04	1.32E+04
ENSG00000104964	AES	1.59	1.59	0.67	1.45E-08	1.32E+04	1.32E+04
ENSG00000164307	ERAP1	0.65	-1.54	-0.62	1.37E-08	-1.32E+04	1.32E+04
ENSG00000118946	PCDH17	0.44	-2.26	-1.17	2.97E-08	-1.31E+04	1.31E+04
ENSG00000102024	PLS3	1.75	1.75	0.81	1.79E-08	1.31E+04	1.31E+04
ENSG00000066697	MSANTD3	1.58	1.58	0.66	1.46E-08	1.31E+04	1.31E+04
ENSG00000167555	ZNF528	1.70	1.70	0.77	1.72E-08	1.30E+04	1.30E+04
ENSG00000151474	FRMD4A	1.57	1.57	0.65	1.48E-08	1.29E+04	1.29E+04
ENSG00000107140	TESK1	1.61	1.61	0.69	1.56E-08	1.29E+04	1.29E+04

ENSG00000176641	RNF152	0.63	-1.59	-0.67	1.55E-08	-1.28E+04	1.28E+04
ENSG00000260006	RP11-469M7.1	0.61	-1.64	-0.71	1.67E-08	-1.27E+04	1.27E+04
ENSG00000230513	THAP7-AS1	2.15	2.15	1.10	2.87E-08	1.27E+04	1.27E+04
ENSG00000196943	NOP9	0.65	-1.53	-0.61	1.45E-08	-1.27E+04	1.27E+04
ENSG00000179965	ZNF771	0.55	-1.83	-0.87	2.10E-08	-1.26E+04	1.26E+04
ENSG00000166451	CENPN	0.65	-1.53	-0.62	1.49E-08	-1.26E+04	1.26E+04
ENSG00000141519	CCDC40	1.86	1.86	0.90	2.21E-08	1.25E+04	1.25E+04
ENSG00000227124	ZNF717	1.67	1.67	0.74	1.81E-08	1.24E+04	1.24E+04
ENSG00000108561	C1QBP	0.63	-1.58	-0.66	1.65E-08	-1.23E+04	1.23E+04
ENSG00000267583	RP11-322E11.5	1.56	1.56	0.64	1.61E-08	1.23E+04	1.23E+04
ENSG00000072201	LNX1	1.75	1.75	0.81	2.07E-08	1.22E+04	1.22E+04
ENSG00000197415	VEPH1	1.57	1.57	0.66	1.68E-08	1.21E+04	1.21E+04
ENSG00000025770	NCAPH2	0.66	-1.51	-0.60	1.58E-08	-1.20E+04	1.20E+04
ENSG00000135932	CAB39	1.55	1.55	0.63	1.66E-08	1.20E+04	1.20E+04
ENSG00000160818	GPATCH4	0.62	-1.62	-0.69	1.81E-08	-1.20E+04	1.20E+04
ENSG00000080839	RBL1	0.65	-1.53	-0.61	1.61E-08	-1.20E+04	1.20E+04
ENSG00000088387	DOCK9	1.56	1.56	0.64	1.72E-08	1.19E+04	1.19E+04
	RP11-18F14.2	1.55	1.55	0.63	1.72E-08	1.18E+04	1.18E+04
ENSG00000259380	RP11-346D14.1	1.88	1.88	0.91	2.59E-08	1.17E+04	1.17E+04
ENSG00000070882	OSBPL3	1.56	1.56	0.64	1.77E-08	1.17E+04	1.17E+04
ENSG00000173451	THAP2	1.62	1.62	0.69	1.91E-08	1.17E+04	1.17E+04
ENSG00000223973	AC068491.1	1.52	1.52	0.60	1.70E-08	1.16E+04	1.16E+04
ENSG00000167378	IRGQ	1.54	1.54	0.62	1.75E-08	1.16E+04	1.16E+04
ENSG00000011566	MAP4K3	1.53	1.53	0.62	1.77E-08	1.15E+04	1.15E+04
ENSG00000200169	RNU5D-1	0.39	-2.55	-1.35	4.99E-08	-1.14E+04	1.14E+04
ENSG00000165886	UBTD1	1.52	1.52	0.60	1.77E-08	1.14E+04	1.14E+04
ENSG00000117481	NSUN4	0.64	-1.57	-0.66	1.93E-08	-1.13E+04	1.13E+04
ENSG00000151552	QDPR	0.47	-2.13	-1.09	3.57E-08	-1.13E+04	1.13E+04
ENSG00000102547	CAB39L	1.57	1.57	0.65	1.94E-08	1.13E+04	1.13E+04
ENSG00000136603	SKIL	1.71	1.71	0.77	2.29E-08	1.13E+04	1.13E+04
ENSG00000123179	EBPL	0.59	-1.69	-0.76	2.27E-08	-1.12E+04	1.12E+04
ENSG00000169379	ARL13B	0.65	-1.55	-0.63	1.90E-08	-1.12E+04	1.12E+04
ENSG00000075407	ZNF37A	1.68	1.68	0.75	2.24E-08	1.12E+04	1.12E+04
ENSG00000113161	HMGCR	0.63	-1.58	-0.66	1.98E-08	-1.12E+04	1.12E+04
ENSG00000240891	PLCXD2	1.93	1.93	0.95	3.00E-08	1.12E+04	1.12E+04
ENSG00000158435	C2orf29	0.64	-1.57	-0.65	2.01E-08	-1.11E+04	1.11E+04
ENSG00000148814	LRRC27	1.67	1.67	0.74	2.30E-08	1.10E+04	1.10E+04
ENSG00000182606	TRAK1	1.57	1.57	0.65	2.03E-08	1.10E+04	1.10E+04
ENSG00000132376	INPP5K	0.66	-1.52	-0.60	1.90E-08	-1.10E+04	1.10E+04
ENSG00000125871	C20orf72	1.54	1.54	0.62	1.95E-08	1.10E+04	1.10E+04
ENSG00000187097	ENTPD5	1.53	1.53	0.61	1.95E-08	1.09E+04	1.09E+04
ENSG00000100003	SEC14L2	0.56	-1.79	-0.84	2.70E-08	-1.09E+04	1.09E+04
ENSG00000087077	TRIP6	0.66	-1.52	-0.60	1.96E-08	-1.08E+04	1.08E+04
ENSG00000278540	ACACA	0.64	-1.55	-0.63	2.05E-08	-1.08E+04	1.08E+04
ENSG00000100911	PSME2	0.65	-1.54	-0.62	2.08E-08	-1.07E+04	1.07E+04
ENSG00000206538	VGLL3	0.60	-1.67	-0.74	2.47E-08	-1.06E+04	1.06E+04
ENSG00000184602	SNN	1.57	1.57	0.65	2.24E-08	1.05E+04	1.05E+04
ENSG00000129636	ITFG1	1.54	1.54	0.63	2.16E-08	1.05E+04	1.05E+04
ENSG00000053372	MRTO4	0.65	-1.53	-0.61	2.16E-08	-1.04E+04	1.04E+04
ENSG00000143322	ABL2	1.57	1.57	0.65	2.29E-08	1.04E+04	1.04E+04
ENSG00000137225	CAPN11	2.33	2.33	1.22	5.07E-08	1.03E+04	1.03E+04
ENSG00000188186	LAMTOR4	0.62	-1.60	-0.68	2.45E-08	-1.02E+04	1.02E+04
ENSG00000100979	PLTP	0.59	-1.69	-0.76	2.74E-08	-1.02E+04	1.02E+04
ENSG00000078269	SYNJ2	1.65	1.65	0.72	2.62E-08	1.02E+04	1.02E+04
ENSG00000157557	ETS2	1.62	1.62	0.70	2.60E-08	1.01E+04	1.01E+04
ENSG00000118418	HMGN3	0.65	-1.54	-0.62	2.34E-08	-1.01E+04	1.01E+04
ENSG00000170145	SIK2	1.65	1.65	0.72	2.70E-08	1.01E+04	1.01E+04
ENSG00000183401	CCDC159	0.53	-1.87	-0.91	3.49E-08	-1.00E+04	1.00E+04
ENSG00000119929	CUTC	0.66	-1.52	-0.60	2.32E-08	-9.98E+03	9.98E+03
ENSG00000116852	KIF21B	0.56	-1.78	-0.83	3.18E-08	-9.98E+03	9.98E+03
ENSG00000221963	APOL6	0.60	-1.67	-0.74	2.82E-08	-9.96E+03	9.96E+03
ENSG00000138587	MNS1	0.46	-2.18	-1.13	4.81E-08	-9.95E+03	9.95E+03
ENSG00000178033	FAM26E	1.51	1.51	0.59	2.34E-08	9.85E+03	9.85E+03
ENSG00000130313	PGLS	1.58	1.58	0.66	2.58E-08	9.84E+03	9.84E+03
ENSG00000213341	CHUK	0.64	-1.56	-0.64	2.51E-08	-9.83E+03	9.83E+03
ENSG00000204161	C10orf128	0.52	-1.93	-0.95	3.86E-08	-9.82E+03	9.82E+03
ENSG00000068489	PRR11	0.65	-1.55	-0.63	2.51E-08	-9.77E+03	9.77E+03
ENSG00000184588	PDE4B	1.55	1.55	0.63	2.56E-08	9.69E+03	9.69E+03
ENSG00000187118	CMC1	0.60	-1.66	-0.73	2.96E-08	-9.65E+03	9.65E+03
ENSG00000129055	ANAPC13	1.59	1.59	0.67	2.75E-08	9.60E+03	9.60E+03
ENSG00000130158	DOCK6	1.52	1.52	0.61	2.52E-08	9.59E+03	9.59E+03
ENSG00000123191	ATP7B	1.63	1.63	0.70	3.01E-08	9.37E+03	9.37E+03
ENSG00000260398	RP11-594N15.3	2.39	2.39	1.25	6.61E-08	9.28E+03	9.28E+03
ENSG00000157193	LRP8	0.66	-1.52	-0.60	2.69E-08	-9.25E+03	9.25E+03
ENSG00000171298	GAA	1.75	1.75	0.81	3.61E-08	9.23E+03	9.23E+03
ENSG00000141560	FN3KRP	0.62	-1.62	-0.70	3.08E-08	-9.23E+03	9.23E+03
	RP11-159D12.9	1.72	1.72	0.78	3.50E-08	9.17E+03	9.17E+03
ENSG00000149489	ROM1	0.51	-1.94	-0.96	4.58E-08	-9.09E+03	9.09E+03
ENSG00000071205	ARHGAP10	1.52	1.52	0.61	2.84E-08	9.04E+03	9.04E+03

ENSG00000163132	MSX1	2.15	2.15	1.10	5.77E-08	8.95E+03	8.95E+03
ENSG00000112186	CAP2	0.65	-1.55	-0.63	3.05E-08	-8.86E+03	8.86E+03
ENSG00000153214	TMEM87B	1.52	1.52	0.61	3.04E-08	8.73E+03	8.73E+03
ENSG00000137274	BPHL	1.55	1.55	0.64	3.21E-08	8.68E+03	8.68E+03
ENSG00000198805	PNP	1.57	1.57	0.65	3.28E-08	8.67E+03	8.67E+03
ENSG00000144445	KANSL1L	1.57	1.57	0.65	3.30E-08	8.64E+03	8.64E+03
ENSG00000277075	HIST1H2AE	0.62	-1.60	-0.68	3.48E-08	-8.58E+03	8.58E+03
ENSG00000167100	SAMD14	0.47	-2.15	-1.10	6.32E-08	-8.55E+03	8.55E+03
ENSG00000106688	SLC1A1	0.65	-1.53	-0.61	3.23E-08	-8.51E+03	8.51E+03
ENSG00000196352	CD55	1.57	1.57	0.65	3.40E-08	8.51E+03	8.51E+03
ENSG00000204084	INPP5B	0.66	-1.52	-0.60	3.21E-08	-8.47E+03	8.47E+03
	CTA-204B4.6	1.60	1.60	0.68	3.62E-08	8.39E+03	8.39E+03
ENSG00000136732	GYPC	1.66	1.66	0.74	3.98E-08	8.34E+03	8.34E+03
ENSG00000278705	HIST1H4B	0.60	-1.68	-0.75	4.06E-08	-8.33E+03	8.33E+03
ENSG00000186575	NF2	1.59	1.59	0.67	3.66E-08	8.31E+03	8.31E+03
ENSG00000196976	LAGE3	0.63	-1.60	-0.68	3.73E-08	-8.28E+03	8.28E+03
ENSG00000170776	AKAP13	1.55	1.55	0.63	3.49E-08	8.27E+03	8.27E+03
ENSG00000172336	POP7	0.66	-1.51	-0.59	3.36E-08	-8.24E+03	8.24E+03
ENSG00000140474	ULK3	0.66	-1.51	-0.60	3.39E-08	-8.21E+03	8.21E+03
ENSG00000151640	DPYSL4	2.09	2.09	1.07	6.50E-08	8.21E+03	8.21E+03
ENSG00000145779	TNFAIP8	0.62	-1.60	-0.68	3.82E-08	-8.19E+03	8.19E+03
ENSG00000171451	DSEL	0.65	-1.54	-0.62	3.53E-08	-8.19E+03	8.19E+03
ENSG00000186594	MIR22HG	1.50	1.50	0.58	3.38E-08	8.14E+03	8.14E+03
ENSG00000146648	EGFR	1.76	1.76	0.82	4.71E-08	8.13E+03	8.13E+03
ENSG00000188365	AC092171.1	2.25	2.25	1.17	7.86E-08	8.04E+03	8.04E+03
ENSG00000164509	IL31RA	0.51	-1.97	-0.98	6.00E-08	-8.04E+03	8.04E+03
ENSG00000182489	XKRX	1.99	1.99	0.99	6.12E-08	8.03E+03	8.03E+03
ENSG00000171097	CCBL1	0.59	-1.70	-0.77	4.51E-08	-8.02E+03	8.02E+03
ENSG00000181284	TMEM102	1.66	1.66	0.73	4.46E-08	7.87E+03	7.87E+03
ENSG00000274471	HERC2P2	0.64	-1.57	-0.65	3.96E-08	-7.87E+03	7.87E+03
ENSG00000136051	KIAA1033	1.69	1.69	0.76	4.64E-08	7.87E+03	7.87E+03
ENSG00000103404	USP31	1.54	1.54	0.62	3.83E-08	7.85E+03	7.85E+03
ENSG00000197386	HTT	1.58	1.58	0.66	4.05E-08	7.84E+03	7.84E+03
ENSG00000120833	SOCS2	1.59	1.59	0.67	4.11E-08	7.83E+03	7.83E+03
ENSG00000161904	LEMD2	1.54	1.54	0.63	3.88E-08	7.83E+03	7.83E+03
ENSG00000132294	EFR3A	1.54	1.54	0.63	3.99E-08	7.73E+03	7.73E+03
ENSG00000196458	ZNF605	1.74	1.74	0.80	5.11E-08	7.69E+03	7.69E+03
ENSG00000100106	TRIOBP	1.52	1.52	0.60	3.91E-08	7.68E+03	7.68E+03
ENSG00000165698	C9orf9	1.92	1.92	0.94	6.28E-08	7.65E+03	7.65E+03
ENSG00000152894	PTPRK	1.54	1.54	0.63	4.09E-08	7.63E+03	7.63E+03
ENSG00000180448	HMHA1	2.45	2.45	1.29	1.03E-07	7.61E+03	7.61E+03
ENSG00000159216	RUNX1	1.58	1.58	0.66	4.30E-08	7.60E+03	7.60E+03
	RP4-811H24.6	2.17	2.17	1.12	8.24E-08	7.55E+03	7.55E+03
ENSG00000105227	PRX	1.83	1.83	0.87	5.88E-08	7.53E+03	7.53E+03
ENSG00000198399	ITSN2	1.59	1.59	0.67	4.52E-08	7.50E+03	7.50E+03
ENSG00000131844	MCCC2	0.67	-1.49	-0.58	3.98E-08	-7.48E+03	7.48E+03
ENSG00000211772	TRBC2	0.65	-1.55	-0.63	4.28E-08	-7.48E+03	7.48E+03
ENSG00000100162	CENPM	0.62	-1.61	-0.69	4.69E-08	-7.45E+03	7.45E+03
ENSG00000100889	PCK2	0.65	-1.55	-0.63	4.38E-08	-7.40E+03	7.40E+03
ENSG00000196418	ZNF124	1.53	1.53	0.61	4.27E-08	7.39E+03	7.39E+03
ENSG00000178075	GRAMD1C	1.73	1.73	0.79	5.56E-08	7.35E+03	7.35E+03
ENSG00000044446	PHKA2	0.65	-1.54	-0.62	4.53E-08	-7.22E+03	7.22E+03
ENSG00000015133	CCDC88C	1.54	1.54	0.62	4.57E-08	7.20E+03	7.20E+03
ENSG00000127837	AAMP	0.66	-1.51	-0.60	4.48E-08	-7.15E+03	7.15E+03
ENSG00000179776	CDH5	1.59	1.59	0.67	5.02E-08	7.11E+03	7.11E+03
ENSG00000140836	ZFHX3	1.54	1.54	0.62	4.75E-08	7.06E+03	7.06E+03
ENSG00000204574	ABCF1	0.62	-1.61	-0.69	5.24E-08	-7.03E+03	7.03E+03
ENSG00000163239	TDRD10	1.84	1.84	0.88	6.86E-08	7.02E+03	7.02E+03
ENSG00000078804	TP53INP2	1.64	1.64	0.71	5.48E-08	7.01E+03	7.01E+03
ENSG00000153363	LINC00467	1.79	1.79	0.84	6.52E-08	7.01E+03	7.01E+03
ENSG00000132603	NIP7	0.66	-1.52	-0.60	4.68E-08	-7.01E+03	7.01E+03
ENSG00000020129	NCDN	1.58	1.58	0.66	5.10E-08	7.00E+03	7.00E+03
ENSG00000119681	LTBP2	1.86	1.86	0.90	7.14E-08	6.98E+03	6.98E+03
ENSG00000150593	PDCD4	1.51	1.51	0.59	4.68E-08	6.97E+03	6.97E+03
ENSG00000170892	TSEN34	0.66	-1.51	-0.59	4.76E-08	-6.90E+03	6.90E+03
ENSG00000143819	EPHX1	0.56	-1.78	-0.83	6.77E-08	-6.85E+03	6.85E+03
ENSG00000153391	INO80C	1.55	1.55	0.63	5.13E-08	6.85E+03	6.85E+03
ENSG00000101019	UQCC	1.63	1.63	0.70	5.67E-08	6.85E+03	6.85E+03
ENSG00000147697	GSDMC	1.92	1.92	0.94	7.89E-08	6.82E+03	6.82E+03
ENSG00000197632	SERPINB2	0.45	-2.21	-1.15	1.06E-07	-6.81E+03	6.81E+03
ENSG00000137135	ARHGEF39	0.52	-1.94	-0.96	8.14E-08	-6.79E+03	6.79E+03
ENSG00000214944	ARHGEF28	1.57	1.57	0.65	5.32E-08	6.79E+03	6.79E+03
ENSG00000159915	ZNF233	2.38	2.38	1.25	1.23E-07	6.79E+03	6.79E+03
ENSG00000213465	ARL2	1.55	1.55	0.63	5.37E-08	6.68E+03	6.68E+03
ENSG00000067057	PFKP	1.58	1.58	0.66	5.57E-08	6.68E+03	6.68E+03
ENSG00000147027	TMEM47	1.58	1.58	0.66	5.65E-08	6.67E+03	6.67E+03
ENSG00000106089	STX1A	1.60	1.60	0.68	5.83E-08	6.63E+03	6.63E+03
ENSG00000135372	NAT10	0.61	-1.63	-0.70	6.05E-08	-6.62E+03	6.62E+03
ENSG00000150756	FAM173B	0.65	-1.53	-0.61	5.36E-08	-6.61E+03	6.61E+03

ENSG00000188070	C11orf95	1.50	1.50	0.59	5.21E-08	6.58E+03	6.58E+03
ENSG00000184357	HIST1H1B	0.64	-1.55	-0.64	5.59E-08	-6.57E+03	6.57E+03
ENSG00000158406	HIST1H4H	0.59	-1.68	-0.75	6.62E-08	-6.54E+03	6.54E+03
ENSG00000243251	PGBD3	0.60	-1.66	-0.73	6.55E-08	-6.49E+03	6.49E+03
ENSG00000160345	C9orf116	1.94	1.94	0.96	8.98E-08	6.47E+03	6.47E+03
ENSG00000111271	ACAD10	1.65	1.65	0.72	6.51E-08	6.46E+03	6.46E+03
ENSG00000167394	ZNF668	1.56	1.56	0.64	5.86E-08	6.45E+03	6.45E+03
ENSG00000260630	SNAI3-AS1	1.73	1.73	0.79	7.24E-08	6.42E+03	6.42E+03
ENSG00000163617	KIAA1407	2.01	2.01	1.01	9.80E-08	6.42E+03	6.42E+03
ENSG00000100216	TOMM22	0.65	-1.54	-0.62	5.72E-08	-6.42E+03	6.42E+03
ENSG00000110422	HIPK3	1.59	1.59	0.67	6.19E-08	6.38E+03	6.38E+03
ENSG00000198478	SH3BGRL2	1.49	1.49	0.58	5.52E-08	6.35E+03	6.35E+03
ENSG00000142408	CACNG8	2.06	2.06	1.04	1.05E-07	6.35E+03	6.35E+03
ENSG00000160058	BSDC1	1.53	1.53	0.62	5.83E-08	6.34E+03	6.34E+03
ENSG00000198498	TMA16	0.67	-1.50	-0.59	5.79E-08	-6.24E+03	6.24E+03
ENSG00000260641	RP11-1299A16.3	1.81	1.81	0.86	8.52E-08	6.22E+03	6.22E+03
ENSG00000035687	ADSS	0.65	-1.53	-0.62	6.11E-08	-6.20E+03	6.20E+03
ENSG00000176749	CDK5R1	1.78	1.78	0.83	8.33E-08	6.18E+03	6.18E+03
ENSG00000258311	BLOC1S1	0.63	-1.59	-0.67	6.60E-08	-6.17E+03	6.17E+03
ENSG00000113240	CLK4	0.63	-1.59	-0.67	6.62E-08	-6.17E+03	6.17E+03
ENSG00000175854	SWI5	1.51	1.51	0.59	5.99E-08	6.16E+03	6.16E+03
ENSG00000186354	C9orf47	0.48	-2.10	-1.07	1.17E-07	-6.14E+03	6.14E+03
ENSG00000108433	GOSR2	0.67	-1.49	-0.58	5.97E-08	-6.11E+03	6.11E+03
ENSG00000065457	ADAT1	1.49	1.49	0.57	5.97E-08	6.09E+03	6.09E+03
ENSG00000243279	PRAF2	0.50	-2.00	-1.00	1.08E-07	-6.08E+03	6.08E+03
ENSG00000006015	C19orf60	0.66	-1.51	-0.59	6.16E-08	-6.07E+03	6.07E+03
ENSG00000259623	RP11-156E6.1	0.63	-1.58	-0.66	6.87E-08	-6.05E+03	6.05E+03
ENSG00000172260	NEGR1	0.58	-1.74	-0.80	8.30E-08	-6.03E+03	6.03E+03
ENSG00000112972	HMGCS1	0.64	-1.56	-0.64	6.73E-08	-6.02E+03	6.02E+03
ENSG00000159055	MIS18A	0.66	-1.51	-0.60	6.40E-08	-5.97E+03	5.97E+03
ENSG00000119922	IFIT2	0.46	-2.16	-1.11	1.32E-07	-5.95E+03	5.95E+03
ENSG00000135446	CDK4	0.65	-1.53	-0.61	6.73E-08	-5.89E+03	5.89E+03
ENSG00000213380	COG8	0.65	-1.54	-0.62	6.83E-08	-5.89E+03	5.89E+03
ENSG00000011028	MRC2	0.49	-2.03	-1.02	1.22E-07	-5.83E+03	5.83E+03
ENSG00000124067	SLC12A4	1.51	1.51	0.60	6.96E-08	5.74E+03	5.74E+03
ENSG00000083838	ZNF446	1.58	1.58	0.66	7.66E-08	5.73E+03	5.73E+03
ENSG00000270722	U1	0.52	-1.93	-0.95	1.15E-07	-5.69E+03	5.69E+03
ENSG00000143493	INTS7	0.66	-1.51	-0.60	7.13E-08	-5.66E+03	5.66E+03
ENSG00000170396	ZNF804A	0.46	-2.19	-1.13	1.50E-07	-5.65E+03	5.65E+03
ENSG00000168298	HIST1H1E	0.65	-1.55	-0.63	7.62E-08	-5.61E+03	5.61E+03
ENSG00000160796	NBEAL2	1.51	1.51	0.59	7.26E-08	5.59E+03	5.59E+03
ENSG00000082497	SERTAD4	1.74	1.74	0.80	9.69E-08	5.58E+03	5.58E+03
ENSG00000197102	DYNC1H1	1.64	1.64	0.71	8.62E-08	5.58E+03	5.58E+03
ENSG00000172936	MYD88	1.84	1.84	0.88	1.09E-07	5.57E+03	5.57E+03
ENSG00000089818	NECAP1	0.67	-1.49	-0.57	7.22E-08	-5.54E+03	5.54E+03
ENSG00000136830	FAM129B	1.57	1.57	0.65	8.15E-08	5.49E+03	5.49E+03
ENSG00000188130	MAPK12	0.67	-1.50	-0.59	7.49E-08	-5.49E+03	5.49E+03
ENSG00000137310	TCF19	0.65	-1.54	-0.62	7.94E-08	-5.46E+03	5.46E+03
ENSG00000143353	LYPLAL1	1.52	1.52	0.60	7.77E-08	5.46E+03	5.46E+03
ENSG00000159231	CBR3	0.64	-1.56	-0.64	8.23E-08	-5.44E+03	5.44E+03
ENSG00000006704	GTF2IRD1	1.57	1.57	0.65	8.38E-08	5.43E+03	5.43E+03
ENSG00000144455	SUMF1	1.50	1.50	0.58	7.64E-08	5.42E+03	5.42E+03
ENSG00000164124	TMEM144	1.65	1.65	0.72	9.39E-08	5.39E+03	5.39E+03
ENSG00000105671	DDX49	0.66	-1.51	-0.60	8.06E-08	-5.34E+03	5.34E+03
ENSG00000166432	ZMAT1	2.05	2.05	1.03	1.49E-07	5.29E+03	5.29E+03
ENSG00000135912	TTLL4	0.66	-1.52	-0.60	8.27E-08	-5.29E+03	5.29E+03
ENSG00000075420	FNDC3B	1.53	1.53	0.61	8.36E-08	5.28E+03	5.28E+03
ENSG00000133641	C12orf29	0.65	-1.55	-0.63	8.64E-08	-5.27E+03	5.27E+03
ENSG00000102384	CENPI	0.65	-1.54	-0.62	8.60E-08	-5.25E+03	5.25E+03
ENSG00000114738	MAPKAPK3	0.65	-1.55	-0.63	8.69E-08	-5.24E+03	5.24E+03
ENSG00000206053	HN1L	0.66	-1.52	-0.61	8.47E-08	-5.24E+03	5.24E+03
ENSG00000185347	C14orf80	0.58	-1.71	-0.77	1.07E-07	-5.23E+03	5.23E+03
ENSG00000215447	BX322557.10	1.64	1.64	0.71	9.80E-08	5.22E+03	5.22E+03
ENSG00000078687	TNRC6C	1.65	1.65	0.72	1.01E-07	5.20E+03	5.20E+03
ENSG00000132646	PCNA	0.65	-1.53	-0.61	8.66E-08	-5.20E+03	5.20E+03
ENSG00000169855	ROBO1	0.66	-1.52	-0.61	8.70E-08	-5.17E+03	5.17E+03
ENSG00000205356	TECPR1	1.54	1.54	0.62	8.92E-08	5.15E+03	5.15E+03
ENSG00000095951	HIVEP1	1.62	1.62	0.70	9.95E-08	5.15E+03	5.15E+03
	RP11-454H13.5	0.46	-2.19	-1.13	1.83E-07	-5.11E+03	5.11E+03
ENSG00000010704	HFE	0.63	-1.59	-0.67	9.77E-08	-5.08E+03	5.08E+03
ENSG00000064726	BTBD1	0.65	-1.53	-0.61	9.11E-08	-5.06E+03	5.06E+03
ENSG00000274997	HIST1H2AH	0.64	-1.55	-0.63	9.52E-08	-5.03E+03	5.03E+03
ENSG00000144231	POLR2D	0.66	-1.51	-0.59	8.98E-08	-5.03E+03	5.03E+03
ENSG00000079150	FKBP7	1.53	1.53	0.62	9.50E-08	4.97E+03	4.97E+03
ENSG00000234745	HLA-B	1.53	1.53	0.62	9.54E-08	4.97E+03	4.97E+03
ENSG00000145354	CISD2	0.62	-1.62	-0.70	1.06E-07	-4.96E+03	4.96E+03
ENSG00000175048	ZDHHC14	0.64	-1.56	-0.64	9.88E-08	-4.96E+03	4.96E+03
ENSG00000177425	PAWR	1.52	1.52	0.60	9.38E-08	4.96E+03	4.96E+03
ENSG00000168894	RNF181	0.66	-1.51	-0.60	9.33E-08	-4.95E+03	4.95E+03

	AC011526.1	1.51	1.51	0.60	9.39E-08	4.94E+03	4.94E+03
ENSG00000196950	SLC39A10	1.61	1.61	0.69	1.07E-07	4.92E+03	4.92E+03
ENSG00000196365	LONP1	0.66	-1.51	-0.59	9.49E-08	-4.89E+03	4.89E+03
ENSG00000033100	CHPF2	1.57	1.57	0.65	1.05E-07	4.86E+03	4.86E+03
ENSG00000174327	SLC16A13	2.00	2.00	1.00	1.69E-07	4.86E+03	4.86E+03
ENSG00000154240	CEP112	1.78	1.78	0.83	1.35E-07	4.84E+03	4.84E+03
ENSG00000155366	RHOC	1.53	1.53	0.62	1.01E-07	4.81E+03	4.81E+03
ENSG00000130749	ZC3H4	1.61	1.61	0.69	1.13E-07	4.81E+03	4.81E+03
ENSG00000204130	RUFY2	1.56	1.56	0.64	1.06E-07	4.79E+03	4.79E+03
ENSG00000084693	AGBL5	0.64	-1.56	-0.64	1.07E-07	-4.77E+03	4.77E+03
ENSG00000178498	DTX3	0.63	-1.59	-0.67	1.12E-07	-4.77E+03	4.77E+03
ENSG00000140403	DNAJA4	0.63	-1.58	-0.66	1.10E-07	-4.75E+03	4.75E+03
ENSG00000163565	IFI16	1.62	1.62	0.70	1.18E-07	4.74E+03	4.74E+03
ENSG00000143401	ANP32E	0.65	-1.54	-0.62	1.07E-07	-4.70E+03	4.70E+03
ENSG00000133401	PDZD2	1.97	1.97	0.98	1.76E-07	4.69E+03	4.69E+03
ENSG00000164331	ANKRA2	1.51	1.51	0.59	1.04E-07	4.69E+03	4.69E+03
ENSG00000183978	COA3	0.67	-1.50	-0.58	1.03E-07	-4.68E+03	4.68E+03
ENSG00000051180	RAD51	0.65	-1.55	-0.63	1.11E-07	-4.66E+03	4.66E+03
ENSG00000197183	C20orf112	1.64	1.64	0.72	1.24E-07	4.66E+03	4.66E+03
ENSG00000221914	PPP2R2A	1.53	1.53	0.61	1.09E-07	4.64E+03	4.64E+03
ENSG00000158856	EPB49	1.67	1.67	0.74	1.30E-07	4.64E+03	4.64E+03
ENSG00000177548	RABEP2	0.67	-1.50	-0.58	1.05E-07	-4.62E+03	4.62E+03
ENSG00000125901	MRPS26	0.68	-1.48	-0.57	1.03E-07	-4.61E+03	4.61E+03
ENSG00000135451	TROAP	0.65	-1.53	-0.62	1.11E-07	-4.60E+03	4.60E+03
ENSG00000134686	PHC2	1.53	1.53	0.61	1.12E-07	4.56E+03	4.56E+03
ENSG00000111671	SPSB2	0.46	-2.20	-1.14	2.38E-07	-4.50E+03	4.50E+03
ENSG00000069275	NUCKS1	0.66	-1.52	-0.60	1.14E-07	-4.50E+03	4.50E+03
ENSG00000117305	HMGCL	1.51	1.51	0.59	1.13E-07	4.48E+03	4.48E+03
ENSG00000183386	FHL3	1.70	1.70	0.77	1.45E-07	4.47E+03	4.47E+03
ENSG00000177189	RPS6KA3	1.60	1.60	0.68	1.28E-07	4.46E+03	4.46E+03
ENSG00000204392	LSM2	0.68	-1.48	-0.57	1.11E-07	-4.44E+03	4.44E+03
ENSG00000079435	LIPE	0.48	-2.07	-1.05	2.20E-07	-4.42E+03	4.42E+03
ENSG00000170166	HOXD4	0.62	-1.62	-0.70	1.35E-07	-4.42E+03	4.42E+03
ENSG00000196646	ZNF136	1.68	1.68	0.75	1.46E-07	4.39E+03	4.39E+03
ENSG00000101096	NFATC2	1.57	1.57	0.65	1.29E-07	4.36E+03	4.36E+03
ENSG00000168701	TMEM208	0.64	-1.57	-0.65	1.33E-07	-4.30E+03	4.30E+03
ENSG00000111231	GPN3	0.68	-1.47	-0.56	1.18E-07	-4.29E+03	4.29E+03
	RP11-329L6.1	0.40	-2.50	-1.32	3.45E-07	-4.26E+03	4.26E+03
ENSG00000221869	CEBPD	0.56	-1.79	-0.84	1.77E-07	-4.25E+03	4.25E+03
ENSG00000122515	ZMIZ2	1.61	1.61	0.69	1.45E-07	4.23E+03	4.23E+03
ENSG00000130766	SESN2	1.51	1.51	0.59	1.29E-07	4.20E+03	4.20E+03
ENSG00000198740	ZNF652	1.56	1.56	0.64	1.39E-07	4.19E+03	4.19E+03
ENSG00000180596	HIST1H2BC	0.64	-1.57	-0.65	1.42E-07	-4.18E+03	4.18E+03
ENSG00000092847	EIF2C1	0.65	-1.54	-0.62	1.35E-07	-4.18E+03	4.18E+03
ENSG00000181222	POLR2A	1.53	1.53	0.62	1.36E-07	4.16E+03	4.16E+03
ENSG00000197785	ATAD3A	0.67	-1.50	-0.58	1.31E-07	-4.15E+03	4.15E+03
ENSG00000151929	BAG3	1.71	1.71	0.77	1.71E-07	4.13E+03	4.13E+03
ENSG00000173611	SCAI	0.59	-1.70	-0.77	1.70E-07	-4.12E+03	4.12E+03
ENSG00000143476	DTL	0.67	-1.49	-0.58	1.32E-07	-4.11E+03	4.11E+03
ENSG00000239836	PSMB9	0.54	-1.86	-0.90	2.08E-07	-4.08E+03	4.08E+03
ENSG00000130559	CAMSAP1	1.53	1.53	0.61	1.41E-07	4.07E+03	4.07E+03
ENSG00000066027	PPP2R5A	1.52	1.52	0.60	1.40E-07	4.05E+03	4.05E+03
ENSG00000109084	TMEM97	0.67	-1.49	-0.57	1.35E-07	-4.05E+03	4.05E+03
ENSG00000179988	PSTK	0.48	-2.08	-1.05	2.64E-07	-4.05E+03	4.05E+03
ENSG00000169504	CLIC4	1.54	1.54	0.63	1.46E-07	4.04E+03	4.04E+03
ENSG00000162458	FBLIM1	1.54	1.54	0.63	1.46E-07	4.04E+03	4.04E+03
ENSG00000070778	PTPN21	1.52	1.52	0.61	1.43E-07	4.03E+03	4.03E+03
ENSG00000079616	KIF22	0.65	-1.54	-0.62	1.46E-07	-4.02E+03	4.02E+03
ENSG00000109775	UFSP2	0.67	-1.49	-0.57	1.37E-07	-4.02E+03	4.02E+03
ENSG00000180543	TSPYL5	1.50	1.50	0.59	1.39E-07	4.02E+03	4.02E+03
ENSG00000082269	FAM135A	1.67	1.67	0.74	1.74E-07	4.01E+03	4.01E+03
ENSG00000149930	TAOK2	1.51	1.51	0.60	1.45E-07	3.98E+03	3.98E+03
ENSG00000135736	CCDC102A	1.81	1.81	0.86	2.08E-07	3.97E+03	3.97E+03
ENSG00000129103	SUMF2	0.67	-1.50	-0.58	1.42E-07	-3.96E+03	3.96E+03
ENSG00000168661	ZNF30	0.58	-1.72	-0.78	1.90E-07	-3.95E+03	3.95E+03
ENSG00000197535	MYO5A	1.52	1.52	0.61	1.50E-07	3.94E+03	3.94E+03
ENSG00000134470	IL15RA	1.65	1.65	0.73	1.77E-07	3.93E+03	3.93E+03
ENSG00000159199	ATP5G1	0.66	-1.52	-0.60	1.49E-07	-3.93E+03	3.93E+03
ENSG00000123415	SMUG1	0.64	-1.56	-0.64	1.58E-07	-3.92E+03	3.92E+03
ENSG00000140450	ARRDC4	0.48	-2.08	-1.05	2.84E-07	-3.90E+03	3.90E+03
ENSG00000125510	OPRL1	1.86	1.86	0.90	2.28E-07	3.90E+03	3.90E+03
ENSG00000204301	NOTCH4	0.67	-1.48	-0.57	1.46E-07	-3.88E+03	3.88E+03
ENSG00000197930	ERO1L	1.52	1.52	0.61	1.56E-07	3.85E+03	3.85E+03
ENSG00000178502	KLHL11	0.65	-1.53	-0.61	1.57E-07	-3.85E+03	3.85E+03
ENSG00000130204	TOMM40	0.67	-1.49	-0.57	1.50E-07	-3.85E+03	3.85E+03
ENSG00000018699	TTC27	0.67	-1.48	-0.57	1.50E-07	-3.83E+03	3.83E+03
ENSG00000130695	CEP85	0.68	-1.48	-0.56	1.49E-07	-3.82E+03	3.82E+03
ENSG00000169231	THBS3	1.58	1.58	0.66	1.73E-07	3.81E+03	3.81E+03
ENSG00000196922	ZNF252P	1.48	1.48	0.56	1.53E-07	3.78E+03	3.78E+03

ENSG00000136205	TNS3	1.78	1.78	0.83	2.22E-07	3.77E+03	3.77E+03
ENSG00000129187	DCTD	1.50	1.50	0.59	1.61E-07	3.74E+03	3.74E+03
ENSG00000132383	RPA1	0.61	-1.65	-0.72	1.95E-07	-3.74E+03	3.74E+03
ENSG00000262580	RP11-334C17.5	2.14	2.14	1.10	3.32E-07	3.72E+03	3.72E+03
ENSG00000182916	TCEAL7	0.61	-1.63	-0.71	1.95E-07	-3.70E+03	3.70E+03
ENSG00000166250	CLMP	0.54	-1.86	-0.90	2.55E-07	-3.69E+03	3.69E+03
ENSG00000140416	TPM1	1.52	1.52	0.60	1.71E-07	3.67E+03	3.67E+03
ENSG00000176105	YES1	1.63	1.63	0.70	1.97E-07	3.66E+03	3.66E+03
ENSG00000214367	HAUS3	0.66	-1.51	-0.60	1.71E-07	-3.66E+03	3.66E+03
ENSG00000137822	TUBGCP4	0.66	-1.51	-0.60	1.71E-07	-3.66E+03	3.66E+03
ENSG00000035862	TIMP2	1.60	1.60	0.68	1.94E-07	3.64E+03	3.64E+03
ENSG00000146083	RNF44	1.62	1.62	0.70	1.99E-07	3.64E+03	3.64E+03
ENSG00000134369	NAV1	1.62	1.62	0.70	2.00E-07	3.62E+03	3.62E+03
ENSG00000043093	DCUN1D1	1.55	1.55	0.63	1.82E-07	3.62E+03	3.62E+03
ENSG00000138085	ATRAID	1.52	1.52	0.60	1.75E-07	3.62E+03	3.62E+03
ENSG00000111846	GCNT2	0.67	-1.49	-0.57	1.71E-07	-3.60E+03	3.60E+03
ENSG00000106714	CNTNAP3	0.66	-1.51	-0.59	1.78E-07	-3.58E+03	3.58E+03
ENSG00000100558	PLEK2	1.60	1.60	0.68	2.01E-07	3.58E+03	3.58E+03
ENSG00000121858	TNFSF10	0.61	-1.64	-0.72	2.11E-07	-3.57E+03	3.57E+03
ENSG00000150787	PTS	0.65	-1.54	-0.63	1.87E-07	-3.57E+03	3.57E+03
ENSG00000087338	GMCL1	0.67	-1.48	-0.57	1.74E-07	-3.56E+03	3.56E+03
ENSG00000134375	TIMM17A	0.64	-1.57	-0.65	1.98E-07	-3.53E+03	3.53E+03
ENSG00000106789	CORO2A	0.52	-1.92	-0.94	2.99E-07	-3.51E+03	3.51E+03
ENSG00000137601	NEK1	1.67	1.67	0.74	2.27E-07	3.50E+03	3.50E+03
ENSG00000110315	RNF141	1.53	1.53	0.61	1.91E-07	3.49E+03	3.49E+03
ENSG00000168256	NKIRAS2	1.50	1.50	0.58	1.84E-07	3.49E+03	3.49E+03
ENSG00000179454	KLHL28	1.67	1.67	0.74	2.30E-07	3.48E+03	3.48E+03
	RP11-231E4.4	1.58	1.58	0.66	2.07E-07	3.48E+03	3.48E+03
ENSG00000099800	TIMM13	0.65	-1.53	-0.62	1.97E-07	-3.45E+03	3.45E+03
ENSG00000151012	SLC7A11	1.69	1.69	0.76	2.41E-07	3.44E+03	3.44E+03
ENSG00000157110	RBPMS	1.57	1.57	0.65	2.08E-07	3.44E+03	3.44E+03
ENSG00000006468	ETV1	1.48	1.48	0.56	1.85E-07	3.43E+03	3.43E+03
ENSG00000173083	HPSE	0.64	-1.56	-0.64	2.07E-07	-3.43E+03	3.43E+03
ENSG00000272551	AC017048.3	0.66	-1.52	-0.61	1.98E-07	-3.42E+03	3.42E+03
ENSG00000160685	ZBTB7B	1.50	1.50	0.58	1.93E-07	3.42E+03	3.42E+03
ENSG00000143228	NUF2	0.64	-1.56	-0.64	2.09E-07	-3.41E+03	3.41E+03
ENSG00000106049	HIBADH	0.68	-1.47	-0.55	1.88E-07	-3.39E+03	3.39E+03
ENSG00000134057	CCNB1	0.65	-1.55	-0.63	2.09E-07	-3.39E+03	3.39E+03
ENSG00000168078	PBK	0.64	-1.55	-0.63	2.11E-07	-3.38E+03	3.38E+03
ENSG00000171311	EXOSC1	0.67	-1.48	-0.57	1.93E-07	-3.38E+03	3.38E+03
ENSG00000102119	EMD	0.66	-1.52	-0.60	2.03E-07	-3.37E+03	3.37E+03
ENSG00000165806	CASP7	1.51	1.51	0.60	2.05E-07	3.35E+03	3.35E+03
ENSG00000143158	MPC2	0.68	-1.47	-0.56	1.97E-07	-3.32E+03	3.32E+03
ENSG00000181544	FANCB	0.59	-1.68	-0.75	2.60E-07	-3.30E+03	3.30E+03
ENSG00000164327	RICTOR	1.99	1.99	0.99	3.63E-07	3.29E+03	3.29E+03
ENSG00000078399	HOXA9	0.67	-1.49	-0.58	2.06E-07	-3.29E+03	3.29E+03
ENSG00000173276	ZNF295	1.51	1.51	0.60	2.13E-07	3.27E+03	3.27E+03
ENSG00000101417	PXMP4	0.58	-1.73	-0.79	2.82E-07	-3.25E+03	3.25E+03
ENSG00000060656	PTPRU	1.90	1.90	0.93	3.43E-07	3.25E+03	3.25E+03
ENSG00000153993	SEMA3D	0.53	-1.89	-0.92	3.42E-07	-3.23E+03	3.23E+03
ENSG00000197016	ZNF470	1.70	1.70	0.77	2.79E-07	3.23E+03	3.23E+03
ENSG00000099992	TBC1D10A	0.55	-1.81	-0.86	3.18E-07	-3.21E+03	3.21E+03
ENSG00000224877	C17orf89	0.68	-1.48	-0.57	2.12E-07	-3.21E+03	3.21E+03
ENSG00000235863	B3GALT4	0.48	-2.08	-1.05	4.19E-07	-3.21E+03	3.21E+03
ENSG00000248890	HHIP-AS1	0.68	-1.46	-0.55	2.09E-07	-3.20E+03	3.20E+03
ENSG00000004799	PDK4	0.56	-1.78	-0.83	3.11E-07	-3.19E+03	3.19E+03
ENSG00000186522	SEPT10	1.54	1.54	0.62	2.33E-07	3.19E+03	3.19E+03
ENSG00000125505	MBOAT7	1.54	1.54	0.62	2.33E-07	3.19E+03	3.19E+03
ENSG00000164164	OTUD4	1.50	1.50	0.59	2.25E-07	3.17E+03	3.17E+03
ENSG00000177873	ZNF619	0.58	-1.72	-0.78	2.98E-07	-3.14E+03	3.14E+03
ENSG00000180354	C7orf41	0.66	-1.52	-0.61	2.36E-07	-3.13E+03	3.13E+03
ENSG00000083844	ZNF264	1.54	1.54	0.62	2.44E-07	3.11E+03	3.11E+03
ENSG00000235823	LINC00263	2.06	2.06	1.04	4.39E-07	3.11E+03	3.11E+03
ENSG00000186994	KANK3	1.49	1.49	0.57	2.31E-07	3.10E+03	3.10E+03
ENSG00000176912	C18orf56	1.89	1.89	0.92	3.74E-07	3.09E+03	3.09E+03
ENSG00000140931	CMTM3	1.51	1.51	0.60	2.41E-07	3.08E+03	3.08E+03
ENSG00000108592	FTSJ3	0.67	-1.50	-0.59	2.40E-07	-3.07E+03	3.07E+03
	KIAA1107	1.97	1.97	0.98	4.13E-07	3.06E+03	3.06E+03
ENSG00000145012	LPP	1.51	1.51	0.59	2.43E-07	3.06E+03	3.06E+03
ENSG00000054148	PHPT1	0.68	-1.48	-0.56	2.34E-07	-3.05E+03	3.05E+03
ENSG00000078967	UBE2D4	0.60	-1.65	-0.73	2.94E-07	-3.05E+03	3.05E+03
ENSG00000058056	USP13	0.66	-1.51	-0.59	2.46E-07	-3.04E+03	3.04E+03
ENSG00000054392	HHAT	1.82	1.82	0.87	3.60E-07	3.04E+03	3.04E+03
ENSG00000124370	MCEE	0.59	-1.70	-0.77	3.17E-07	-3.03E+03	3.03E+03
ENSG00000116667	C1orf21	0.67	-1.48	-0.57	2.40E-07	-3.02E+03	3.02E+03
ENSG00000187801	ZNF643	1.88	1.88	0.91	3.91E-07	3.00E+03	3.00E+03
ENSG00000086544	ITPKC	1.47	1.47	0.55	2.40E-07	2.99E+03	2.99E+03
ENSG00000139190	VAMP1	0.52	-1.92	-0.94	4.19E-07	-2.96E+03	2.96E+03
ENSG00000131470	PSMC3IP	0.60	-1.66	-0.73	3.15E-07	-2.95E+03	2.95E+03

ENSG00000149948	HMGA2	0.66	-1.51	-0.59	2.65E-07	-2.93E+03	2.93E+03
ENSG00000115310	RTN4	1.54	1.54	0.62	2.78E-07	2.92E+03	2.92E+03
ENSG00000162910	MRPL55	0.68	-1.47	-0.56	2.55E-07	-2.92E+03	2.92E+03
ENSG00000126461	SCAF1	1.52	1.52	0.61	2.81E-07	2.87E+03	2.87E+03
ENSG00000164010	ERMAP	1.59	1.59	0.67	3.13E-07	2.84E+03	2.84E+03
ENSG00000128928	IVD	0.66	-1.50	-0.59	2.80E-07	-2.84E+03	2.84E+03
ENSG00000131732	ZCCHC9	0.67	-1.50	-0.59	2.81E-07	-2.83E+03	2.83E+03
ENSG00000124802	EEF1E1	0.66	-1.50	-0.59	2.84E-07	-2.82E+03	2.82E+03
ENSG00000077684	PHF17	0.67	-1.49	-0.57	2.79E-07	-2.82E+03	2.82E+03
ENSG00000254470	AP5B1	1.66	1.66	0.73	3.47E-07	2.82E+03	2.82E+03
ENSG00000078177	N4BP2	2.10	2.10	1.07	5.68E-07	2.79E+03	2.79E+03
ENSG00000165060	FXN	0.63	-1.58	-0.66	3.21E-07	-2.78E+03	2.78E+03
ENSG00000162227	TAF6L	0.65	-1.54	-0.63	3.08E-07	-2.78E+03	2.78E+03
ENSG00000132274	TRIM22	0.67	-1.50	-0.59	2.93E-07	-2.78E+03	2.78E+03
ENSG00000028310	BRD9	0.68	-1.46	-0.55	2.78E-07	-2.77E+03	2.77E+03
ENSG00000162772	ATF3	1.93	1.93	0.95	4.85E-07	2.77E+03	2.77E+03
ENSG00000173898	SPTBN2	1.49	1.49	0.57	2.91E-07	2.76E+03	2.76E+03
ENSG00000277969	CTB-58E17.1	2.26	2.26	1.17	6.70E-07	2.76E+03	2.76E+03
ENSG00000119636	CCDC176	1.99	1.99	0.99	5.25E-07	2.75E+03	2.75E+03
ENSG00000157107	FCHO2	1.55	1.55	0.63	3.18E-07	2.75E+03	2.75E+03
ENSG00000142798	HSPG2	1.53	1.53	0.61	3.11E-07	2.74E+03	2.74E+03
ENSG00000165244	ZNF367	0.67	-1.49	-0.58	3.01E-07	-2.72E+03	2.72E+03
ENSG00000176454	LPCAT4	0.65	-1.53	-0.61	3.16E-07	-2.72E+03	2.72E+03
ENSG00000013016	EHD3	0.61	-1.64	-0.71	3.62E-07	-2.72E+03	2.72E+03
ENSG00000167700	MFSD3	0.61	-1.63	-0.70	3.61E-07	-2.71E+03	2.71E+03
ENSG00000178882	FAM101A	0.65	-1.55	-0.63	3.26E-07	-2.71E+03	2.71E+03
ENSG00000174799	CEP135	1.78	1.78	0.83	4.33E-07	2.71E+03	2.71E+03
ENSG00000218510	LINC00339	1.55	1.55	0.63	3.34E-07	2.68E+03	2.68E+03
ENSG00000166012	TAF1D	0.65	-1.54	-0.62	3.30E-07	-2.68E+03	2.68E+03
ENSG00000181026	AEN	1.49	1.49	0.58	3.12E-07	2.67E+03	2.67E+03
ENSG00000213186	TRIM59	0.66	-1.51	-0.60	3.20E-07	-2.67E+03	2.67E+03
ENSG00000203805	PPAPDC1A	1.50	1.50	0.59	3.17E-07	2.67E+03	2.67E+03
ENSG00000125650	PSPN	1.88	1.88	0.91	5.03E-07	2.65E+03	2.65E+03
ENSG00000255112	CHMP1B	1.49	1.49	0.57	3.15E-07	2.65E+03	2.65E+03
ENSG00000127603	MACF1	1.53	1.53	0.61	3.39E-07	2.63E+03	2.63E+03
ENSG00000147526	TACC1	1.51	1.51	0.59	3.42E-07	2.58E+03	2.58E+03
ENSG00000198556	ZNF789	0.63	-1.60	-0.68	3.87E-07	-2.57E+03	2.57E+03
ENSG00000182224	CYB5D1	0.68	-1.47	-0.55	3.31E-07	-2.55E+03	2.55E+03
ENSG00000124257	NEURL2	2.03	2.03	1.02	6.33E-07	2.55E+03	2.55E+03
ENSG00000177337	DLGAP1-AS1	1.55	1.55	0.64	3.76E-07	2.53E+03	2.53E+03
ENSG00000198873	GRK5	1.51	1.51	0.60	3.58E-07	2.53E+03	2.53E+03
ENSG00000167543	TP53I13	0.66	-1.52	-0.60	3.61E-07	-2.52E+03	2.52E+03
ENSG00000086065	CHMP5	1.52	1.52	0.60	3.66E-07	2.51E+03	2.51E+03
ENSG00000142494	SLC47A1	0.60	-1.68	-0.75	4.54E-07	-2.50E+03	2.50E+03
ENSG00000049541	RFC2	0.69	-1.45	-0.54	3.43E-07	-2.48E+03	2.48E+03
ENSG00000132356	PRKAA1	0.67	-1.50	-0.59	3.67E-07	-2.48E+03	2.48E+03
ENSG00000181804	SLC9A9	0.44	-2.27	-1.18	8.45E-07	-2.47E+03	2.47E+03
ENSG00000088826	SMOX	1.49	1.49	0.58	3.66E-07	2.47E+03	2.47E+03
ENSG00000174917	C19orf70	0.68	-1.47	-0.55	3.54E-07	-2.47E+03	2.47E+03
ENSG00000113456	RAD1	0.65	-1.54	-0.62	3.93E-07	-2.46E+03	2.46E+03
ENSG00000240682	ISY1	0.65	-1.54	-0.62	3.94E-07	-2.45E+03	2.45E+03
ENSG00000144935	TRPC1	1.51	1.51	0.59	3.78E-07	2.45E+03	2.45E+03
ENSG00000168758	SEMA4C	1.48	1.48	0.56	3.63E-07	2.45E+03	2.45E+03
ENSG00000170525	PFKFB3	1.48	1.48	0.57	3.75E-07	2.42E+03	2.42E+03
ENSG00000205476	CCDC85C	1.55	1.55	0.63	4.11E-07	2.42E+03	2.42E+03
ENSG00000179958	DCTPP1	0.66	-1.52	-0.60	3.95E-07	-2.42E+03	2.42E+03
ENSG00000198382	UVRAG	1.49	1.49	0.58	3.87E-07	2.40E+03	2.40E+03
ENSG00000115306	SPTBN1	1.53	1.53	0.62	4.10E-07	2.39E+03	2.39E+03
ENSG00000124216	SNAI1	1.88	1.88	0.91	6.25E-07	2.38E+03	2.38E+03
ENSG00000171320	ESCO2	0.62	-1.62	-0.69	4.62E-07	-2.38E+03	2.38E+03
ENSG00000100151	PICK1	0.65	-1.55	-0.63	4.26E-07	-2.38E+03	2.38E+03
ENSG00000159596	TMEM69	0.69	-1.45	-0.54	3.79E-07	-2.36E+03	2.36E+03
ENSG00000137364	TPMT	0.68	-1.47	-0.55	3.87E-07	-2.36E+03	2.36E+03
ENSG00000099977	DDT	0.67	-1.49	-0.58	4.05E-07	-2.35E+03	2.35E+03
ENSG00000100320	RBFOX2	1.50	1.50	0.58	4.08E-07	2.35E+03	2.35E+03
ENSG00000130382	CTC-503J8.6	0.67	-1.50	-0.59	4.12E-07	-2.34E+03	2.34E+03
ENSG00000167173	C15orf39	1.62	1.62	0.70	4.83E-07	2.33E+03	2.33E+03
ENSG00000174405	LIG4	1.67	1.67	0.74	5.10E-07	2.33E+03	2.33E+03
ENSG00000121486	TRMT1L	1.51	1.51	0.59	4.22E-07	2.32E+03	2.32E+03
ENSG00000166289	PLEKHF1	1.61	1.61	0.69	4.82E-07	2.32E+03	2.32E+03
ENSG00000099381	SETD1A	1.58	1.58	0.66	4.62E-07	2.32E+03	2.32E+03
ENSG00000172830	SSH3	0.58	-1.72	-0.78	5.53E-07	-2.31E+03	2.31E+03
ENSG00000119514	GALNT12	1.79	1.79	0.84	6.03E-07	2.31E+03	2.31E+03
ENSG00000174307	PHLDA3	1.47	1.47	0.56	4.09E-07	2.30E+03	2.30E+03
ENSG00000136861	CDK5RAP2	0.68	-1.47	-0.55	4.08E-07	-2.30E+03	2.30E+03
ENSG00000198001	IRAK4	1.46	1.46	0.55	4.16E-07	2.27E+03	2.27E+03
ENSG00000126243	LRFN3	1.70	1.70	0.77	5.66E-07	2.26E+03	2.26E+03
ENSG00000088280	ASAP3	0.67	-1.50	-0.58	4.40E-07	-2.25E+03	2.25E+03
ENSG00000137955	RABGGTB	0.66	-1.51	-0.60	4.54E-07	-2.25E+03	2.25E+03

ENSG00000169718	DUS1L	0.69	-1.45	-0.54	4.19E-07	-2.24E+03	2.24E+03
ENSG00000197860	SGTB	1.45	1.45	0.54	4.26E-07	2.23E+03	2.23E+03
ENSG00000148341	SH3GLB2	0.67	-1.50	-0.59	4.55E-07	-2.23E+03	2.23E+03
ENSG00000164463	CREBRF	1.72	1.72	0.78	5.99E-07	2.22E+03	2.22E+03
ENSG00000173486	FKBP2	0.67	-1.48	-0.57	4.46E-07	-2.22E+03	2.22E+03
ENSG00000152137	HSPB8	1.51	1.51	0.59	4.72E-07	2.19E+03	2.19E+03
ENSG00000062822	POLD1	0.67	-1.49	-0.58	4.64E-07	-2.19E+03	2.19E+03
ENSG00000073536	NLE1	0.66	-1.51	-0.60	4.77E-07	-2.19E+03	2.19E+03
ENSG00000137831	UACA	1.73	1.73	0.79	6.30E-07	2.19E+03	2.19E+03
ENSG00000135801	TAF5L	0.69	-1.45	-0.54	4.43E-07	-2.18E+03	2.18E+03
ENSG00000067208	EVI5	1.47	1.47	0.56	4.54E-07	2.18E+03	2.18E+03
ENSG00000109906	ZBTB16	0.59	-1.71	-0.77	6.15E-07	-2.18E+03	2.18E+03
ENSG00000127334	DYRK2	1.45	1.45	0.53	4.42E-07	2.17E+03	2.17E+03
ENSG00000055609	MLL3	1.52	1.52	0.61	4.91E-07	2.17E+03	2.17E+03
ENSG00000130702	LAMA5	1.45	1.45	0.54	4.47E-07	2.17E+03	2.17E+03
ENSG00000152939	MARVELD2	0.62	-1.62	-0.70	5.58E-07	-2.17E+03	2.17E+03
	RP4-564F22.2	0.65	-1.55	-0.63	5.13E-07	-2.16E+03	2.16E+03
ENSG00000166401	SERPINB8	0.69	-1.45	-0.54	4.54E-07	-2.16E+03	2.16E+03
ENSG00000008441	NFIX	0.62	-1.62	-0.70	5.72E-07	-2.14E+03	2.14E+03
ENSG00000198056	PRIM1	0.67	-1.48	-0.57	4.78E-07	-2.14E+03	2.14E+03
ENSG00000179091	CYC1	0.67	-1.50	-0.59	4.95E-07	-2.13E+03	2.13E+03
ENSG00000133131	MORC4	0.66	-1.51	-0.60	5.07E-07	-2.13E+03	2.13E+03
ENSG00000146063	TRIM41	1.50	1.50	0.59	5.02E-07	2.12E+03	2.12E+03
ENSG00000090266	NDUFB2	0.63	-1.58	-0.66	5.54E-07	-2.12E+03	2.12E+03
ENSG00000167716	WDR81	1.51	1.51	0.59	5.11E-07	2.11E+03	2.11E+03
ENSG00000095383	TBC1D2	1.66	1.66	0.73	6.26E-07	2.10E+03	2.10E+03
ENSG00000146828	SLC12A9	0.68	-1.46	-0.55	4.88E-07	-2.09E+03	2.09E+03
ENSG00000146223	RPL7L1	0.67	-1.49	-0.57	5.07E-07	-2.09E+03	2.09E+03
ENSG00000111110	PPM1H	1.87	1.87	0.90	8.04E-07	2.08E+03	2.08E+03
ENSG00000099385	BCL7C	0.69	-1.46	-0.54	5.01E-07	-2.06E+03	2.06E+03
ENSG00000113369	ARRDC3	1.51	1.51	0.59	5.38E-07	2.06E+03	2.06E+03
ENSG00000184208	C22orf46	2.03	2.03	1.02	9.83E-07	2.05E+03	2.05E+03
ENSG00000100368	CSF2RB	0.69	-1.45	-0.53	5.00E-07	-2.05E+03	2.05E+03
ENSG00000267194	RP1-193H18.2	0.54	-1.86	-0.90	8.28E-07	-2.04E+03	2.04E+03
ENSG00000179820	MYADM	1.45	1.45	0.53	5.02E-07	2.04E+03	2.04E+03
ENSG00000151693	ASAP2	1.49	1.49	0.58	5.40E-07	2.03E+03	2.03E+03
ENSG00000165046	LETM2	1.57	1.57	0.65	6.00E-07	2.03E+03	2.03E+03
ENSG00000255517	CTD-3074O7.5	1.93	1.93	0.95	9.19E-07	2.02E+03	2.02E+03
ENSG00000070759	TESK2	0.48	-2.06	-1.05	1.08E-06	-1.99E+03	1.99E+03
ENSG00000107968	MAP3K8	0.53	-1.89	-0.92	9.10E-07	-1.98E+03	1.98E+03
ENSG00000257261	RP11-96H19.1	2.52	2.52	1.33	1.70E-06	1.93E+03	1.93E+03
ENSG00000006625	GGCT	0.69	-1.44	-0.53	5.58E-07	-1.93E+03	1.93E+03
ENSG00000228716	DHFR	0.66	-1.51	-0.59	6.16E-07	-1.92E+03	1.92E+03
ENSG00000162086	ZNF75A	1.51	1.51	0.60	6.21E-07	1.92E+03	1.92E+03
ENSG00000249867	RP11-115J23.1	0.53	-1.89	-0.92	9.77E-07	-1.92E+03	1.92E+03
ENSG00000160214	RRP1	0.69	-1.44	-0.53	5.68E-07	-1.91E+03	1.91E+03
ENSG00000133863	TEX15	0.50	-1.98	-0.99	1.09E-06	-1.90E+03	1.90E+03
ENSG00000106244	PDAP1	0.65	-1.53	-0.61	6.55E-07	-1.89E+03	1.89E+03
ENSG00000167721	TSR1	0.67	-1.50	-0.59	6.33E-07	-1.89E+03	1.89E+03
ENSG00000135048	TMEM2	1.51	1.51	0.59	6.41E-07	1.88E+03	1.88E+03
ENSG00000162430	SEPN1	1.49	1.49	0.58	6.33E-07	1.88E+03	1.88E+03
ENSG00000115425	PECR	0.62	-1.61	-0.68	7.45E-07	-1.86E+03	1.86E+03
ENSG00000178425	NT5DC1	0.69	-1.45	-0.54	6.09E-07	-1.86E+03	1.86E+03
ENSG00000107438	PDLIM1	0.66	-1.51	-0.60	6.61E-07	-1.86E+03	1.86E+03
ENSG00000161243	FBXO27	0.49	-2.05	-1.04	1.22E-06	-1.86E+03	1.86E+03
ENSG00000188295	ZNF669	1.56	1.56	0.65	7.12E-07	1.86E+03	1.86E+03
ENSG00000152782	PANK1	0.62	-1.62	-0.69	7.64E-07	-1.85E+03	1.85E+03
ENSG00000126804	ZBTB1	1.47	1.47	0.56	6.39E-07	1.84E+03	1.84E+03
ENSG00000175768	TOMM5	0.57	-1.75	-0.81	9.07E-07	-1.84E+03	1.84E+03
ENSG00000175104	TRAF6	1.50	1.50	0.59	6.74E-07	1.83E+03	1.83E+03
ENSG00000102984	ZNF821	1.63	1.63	0.70	7.91E-07	1.83E+03	1.83E+03
ENSG00000187605	TET3	1.69	1.69	0.76	8.59E-07	1.83E+03	1.83E+03
ENSG00000114268	PFKFB4	0.55	-1.81	-0.86	9.92E-07	-1.82E+03	1.82E+03
ENSG00000164967	RPP25L	0.63	-1.60	-0.67	7.71E-07	-1.82E+03	1.82E+03
ENSG00000145244	CORIN	0.31	-3.23	-1.69	3.17E-06	-1.82E+03	1.82E+03
ENSG00000128591	FLNC	1.62	1.62	0.70	7.97E-07	1.81E+03	1.81E+03
ENSG00000143776	CDC42BPA	1.50	1.50	0.58	6.83E-07	1.81E+03	1.81E+03
ENSG00000133704	IPO8	0.68	-1.47	-0.55	6.58E-07	-1.81E+03	1.81E+03
ENSG00000043143	PHF15	1.44	1.44	0.53	6.38E-07	1.80E+03	1.80E+03
ENSG00000131788	PIAS3	0.66	-1.51	-0.59	7.01E-07	-1.80E+03	1.80E+03
ENSG00000172985	SH3RF3	1.59	1.59	0.67	7.83E-07	1.79E+03	1.79E+03
ENSG00000143409	FAM63A	0.63	-1.59	-0.67	7.87E-07	-1.79E+03	1.79E+03
ENSG00000168172	HOOK3	1.46	1.46	0.54	6.69E-07	1.78E+03	1.78E+03
ENSG00000184207	PGP	0.68	-1.47	-0.55	6.83E-07	-1.78E+03	1.78E+03
ENSG00000197024	ZNF398	1.48	1.48	0.57	6.94E-07	1.78E+03	1.78E+03
ENSG00000182054	IDH2	0.68	-1.47	-0.55	6.84E-07	-1.77E+03	1.77E+03
ENSG00000177192	PUS1	0.67	-1.50	-0.59	7.23E-07	-1.76E+03	1.76E+03
ENSG00000108474	PIGL	0.65	-1.53	-0.62	7.66E-07	-1.75E+03	1.75E+03
ENSG00000089157	RPLP0	0.67	-1.48	-0.57	7.17E-07	-1.75E+03	1.75E+03

ENSG00000152253	SPC25	0.66	-1.52	-0.60	7.52E-07	-1.75E+03	1.75E+03
ENSG00000166341	DCHS1	1.67	1.67	0.74	9.13E-07	1.75E+03	1.75E+03
ENSG00000081923	ATP8B1	1.47	1.47	0.56	7.19E-07	1.74E+03	1.74E+03
ENSG00000237649	KIFC1	0.67	-1.50	-0.59	7.45E-07	-1.74E+03	1.74E+03
ENSG00000241685	ARPC1A	1.48	1.48	0.57	7.30E-07	1.73E+03	1.73E+03
ENSG00000070614	NDST1	1.47	1.47	0.56	7.31E-07	1.72E+03	1.72E+03
ENSG00000125898	FAM110A	1.90	1.90	0.93	1.23E-06	1.71E+03	1.71E+03
ENSG00000006282	SPATA20	1.48	1.48	0.57	7.62E-07	1.70E+03	1.70E+03
ENSG00000247950	SEC24B-AS1	1.89	1.89	0.92	1.23E-06	1.70E+03	1.70E+03
ENSG00000100393	EP300	1.60	1.60	0.68	8.88E-07	1.70E+03	1.70E+03
ENSG00000102103	PQBP1	0.67	-1.50	-0.59	7.87E-07	-1.69E+03	1.69E+03
ENSG00000165724	ZMYND19	0.68	-1.47	-0.56	7.58E-07	-1.69E+03	1.69E+03
ENSG00000156113	KCNMA1	2.06	2.06	1.04	1.49E-06	1.69E+03	1.69E+03
ENSG00000128590	DNAJB9	0.67	-1.49	-0.57	7.75E-07	-1.69E+03	1.69E+03
ENSG00000138434	SSFA2	1.48	1.48	0.57	7.77E-07	1.68E+03	1.68E+03
ENSG00000267002	RP11-242D8.1	2.11	2.11	1.07	1.60E-06	1.66E+03	1.66E+03
ENSG00000168303	MPLKIP	0.66	-1.51	-0.60	8.33E-07	-1.66E+03	1.66E+03
ENSG00000198075	SULT1C4	0.56	-1.77	-0.83	1.15E-06	-1.65E+03	1.65E+03
ENSG00000180346	TIGD2	0.61	-1.65	-0.72	1.01E-06	-1.65E+03	1.65E+03
ENSG00000148229	POLE3	0.68	-1.46	-0.55	7.97E-07	-1.64E+03	1.64E+03
ENSG00000111669	TPI1	0.67	-1.48	-0.57	8.26E-07	-1.63E+03	1.63E+03
ENSG00000167799	NUDT8	0.55	-1.82	-0.86	1.25E-06	-1.63E+03	1.63E+03
ENSG00000115109	EPB41L5	0.68	-1.48	-0.56	8.24E-07	-1.63E+03	1.63E+03
ENSG00000126456	IRF3	0.68	-1.46	-0.55	8.06E-07	-1.63E+03	1.63E+03
ENSG00000266173	STRADA	0.54	-1.84	-0.88	1.28E-06	-1.63E+03	1.63E+03
ENSG00000161647	MPP3	0.60	-1.65	-0.73	1.04E-06	-1.63E+03	1.63E+03
ENSG00000231074	HCG18	0.68	-1.47	-0.56	8.35E-07	-1.61E+03	1.61E+03
ENSG00000114686	MRPL3	0.68	-1.46	-0.55	8.20E-07	-1.61E+03	1.61E+03
	AC108488.3	1.57	1.57	0.65	9.50E-07	1.61E+03	1.61E+03
ENSG00000187244	BCAM	0.51	-1.97	-0.98	1.50E-06	-1.61E+03	1.61E+03
ENSG00000173166	RAPH1	0.68	-1.46	-0.55	8.26E-07	-1.61E+03	1.61E+03
ENSG00000145687	SSBP2	0.66	-1.51	-0.59	8.84E-07	-1.61E+03	1.61E+03
ENSG00000147155	EBP	0.68	-1.47	-0.55	8.38E-07	-1.60E+03	1.60E+03
ENSG00000102871	TRADD	0.64	-1.55	-0.64	9.41E-07	-1.60E+03	1.60E+03
ENSG00000066322	ELOVL1	1.48	1.48	0.56	8.61E-07	1.59E+03	1.59E+03
ENSG00000198833	UBE2J1	1.52	1.52	0.61	9.22E-07	1.59E+03	1.59E+03
ENSG00000234546	RP3-510D11.2	2.18	2.18	1.12	1.89E-06	1.58E+03	1.58E+03
ENSG00000198826	ARHGAP11A	0.61	-1.64	-0.72	1.08E-06	-1.58E+03	1.58E+03
ENSG00000168159	RNF187	1.49	1.49	0.57	8.81E-07	1.58E+03	1.58E+03
ENSG00000223496	EXOSC6	0.69	-1.45	-0.53	8.36E-07	-1.58E+03	1.58E+03
ENSG00000162337	LRP5	1.47	1.47	0.55	8.62E-07	1.58E+03	1.58E+03
ENSG00000139428	MMAB	0.69	-1.46	-0.54	8.58E-07	-1.57E+03	1.57E+03
ENSG00000276293	PIP4K2B	0.68	-1.46	-0.55	8.67E-07	-1.57E+03	1.57E+03
ENSG00000153956	CACNA2D1	1.88	1.88	0.91	1.44E-06	1.57E+03	1.57E+03
ENSG00000149182	ARFGAP2	0.69	-1.46	-0.54	8.66E-07	-1.56E+03	1.56E+03
ENSG00000135473	PAN2	1.55	1.55	0.63	9.93E-07	1.56E+03	1.56E+03
ENSG00000232164	AC092669.3	1.76	1.76	0.81	1.28E-06	1.55E+03	1.55E+03
ENSG00000196628	TCF4	1.57	1.57	0.65	1.04E-06	1.54E+03	1.54E+03
ENSG00000070950	RAD18	0.67	-1.48	-0.57	9.26E-07	-1.54E+03	1.54E+03
	RP11-382A18.1	2.15	2.15	1.10	1.96E-06	1.53E+03	1.53E+03
ENSG00000177374	HIC1	1.48	1.48	0.57	9.39E-07	1.53E+03	1.53E+03
ENSG00000197312	DDI2	1.54	1.54	0.62	1.01E-06	1.53E+03	1.53E+03
ENSG00000174775	HRAS	1.45	1.45	0.53	8.94E-07	1.53E+03	1.53E+03
ENSG00000157426	AASDH	0.67	-1.49	-0.57	9.48E-07	-1.53E+03	1.53E+03
ENSG00000204262	COL5A2	0.67	-1.48	-0.57	9.44E-07	-1.53E+03	1.53E+03
ENSG00000254726	MEX3A	1.62	1.62	0.69	1.12E-06	1.53E+03	1.53E+03
ENSG00000166900	STX3	1.45	1.45	0.54	9.10E-07	1.52E+03	1.52E+03
ENSG00000168843	FSTL5	0.58	-1.74	-0.80	1.32E-06	-1.51E+03	1.51E+03
ENSG00000176444	CLK2	0.69	-1.45	-0.54	9.25E-07	-1.51E+03	1.51E+03
ENSG00000186866	POFUT2	1.44	1.44	0.53	9.13E-07	1.51E+03	1.51E+03
ENSG00000181038	METTL23	0.64	-1.57	-0.65	1.09E-06	-1.50E+03	1.50E+03
ENSG00000148835	TAF5	0.68	-1.46	-0.55	9.48E-07	-1.50E+03	1.50E+03
ENSG00000158286	RNF207	0.50	-2.01	-1.01	1.80E-06	-1.50E+03	1.50E+03
ENSG00000111817	DSE	0.68	-1.46	-0.55	9.54E-07	-1.50E+03	1.50E+03
ENSG00000101246	ARFRP1	0.70	-1.44	-0.52	9.45E-07	-1.48E+03	1.48E+03
ENSG00000227051	C14orf132	0.60	-1.67	-0.74	1.28E-06	-1.48E+03	1.48E+03
ENSG00000213123	TCTEX1D2	1.59	1.59	0.67	1.17E-06	1.47E+03	1.47E+03
ENSG00000150457	LATS2	1.46	1.46	0.55	9.91E-07	1.47E+03	1.47E+03
ENSG00000166311	SMPD1	1.60	1.60	0.68	1.19E-06	1.47E+03	1.47E+03
ENSG00000158163	DZIP1L	0.53	-1.90	-0.93	1.68E-06	-1.46E+03	1.46E+03
ENSG00000161558	TMEM143	2.00	2.00	1.00	1.86E-06	1.46E+03	1.46E+03
ENSG00000125520	SLC2A4RG	0.69	-1.45	-0.54	9.95E-07	-1.46E+03	1.46E+03
ENSG00000142627	EPHA2	1.65	1.65	0.72	1.28E-06	1.45E+03	1.45E+03
	RP11-322M19.2	2.00	2.00	1.00	1.92E-06	1.44E+03	1.44E+03
ENSG00000134815	DHX34	1.47	1.47	0.56	1.05E-06	1.44E+03	1.44E+03
ENSG00000110711	AIP	0.70	-1.43	-0.52	9.97E-07	-1.43E+03	1.43E+03
ENSG00000170312	CDK1	0.66	-1.50	-0.59	1.10E-06	-1.43E+03	1.43E+03
ENSG00000089876	DHX32	1.46	1.46	0.55	1.06E-06	1.42E+03	1.42E+03
ENSG00000100311	PDGFB	1.51	1.51	0.59	1.13E-06	1.42E+03	1.42E+03

ENSG00000120925	RNF170	1.46	1.46	0.54	1.06E-06	1.42E+03	1.42E+03
ENSG00000100485	SOS2	1.45	1.45	0.53	1.04E-06	1.42E+03	1.42E+03
ENSG00000107485	GATA3	1.78	1.78	0.83	1.60E-06	1.41E+03	1.41E+03
ENSG00000090581	GNPTG	0.67	-1.48	-0.57	1.12E-06	-1.40E+03	1.40E+03
ENSG00000165494	PCF11	1.50	1.50	0.59	1.15E-06	1.40E+03	1.40E+03
ENSG00000124243	BCAS4	0.51	-1.96	-0.97	1.96E-06	-1.40E+03	1.40E+03
ENSG00000140382	HMG20A	1.47	1.47	0.56	1.11E-06	1.40E+03	1.40E+03
ENSG00000188343	FAM92A1	0.70	-1.44	-0.52	1.06E-06	-1.40E+03	1.40E+03
ENSG00000101079	NDRG3	0.69	-1.44	-0.53	1.07E-06	-1.40E+03	1.40E+03
ENSG00000198858	R3HDM4	1.44	1.44	0.53	1.08E-06	1.39E+03	1.39E+03
ENSG00000180530	NRIP1	1.96	1.96	0.97	1.98E-06	1.39E+03	1.39E+03
ENSG00000108798	ABI3	0.66	-1.51	-0.60	1.19E-06	-1.38E+03	1.38E+03
ENSG00000226950	DANCR	0.69	-1.44	-0.53	1.09E-06	-1.38E+03	1.38E+03
ENSG00000107625	DDX50	0.70	-1.44	-0.52	1.08E-06	-1.38E+03	1.38E+03
ENSG00000112137	PHACTR1	1.69	1.69	0.76	1.51E-06	1.38E+03	1.38E+03
ENSG00000185728	YTHDF3	1.50	1.50	0.59	1.19E-06	1.38E+03	1.38E+03
ENSG00000130856	ZNF236	1.43	1.43	0.51	1.07E-06	1.38E+03	1.38E+03
ENSG00000112245	PTP4A1	1.50	1.50	0.58	1.19E-06	1.37E+03	1.37E+03
ENSG00000163947	ARHGEF3	1.44	1.44	0.53	1.11E-06	1.37E+03	1.37E+03
ENSG00000150779	TIMM8B	0.68	-1.46	-0.55	1.15E-06	-1.36E+03	1.36E+03
ENSG00000251379	RP11-484O2.1	2.26	2.26	1.18	2.78E-06	1.36E+03	1.36E+03
ENSG00000131069	ACSS2	0.70	-1.43	-0.52	1.14E-06	-1.34E+03	1.34E+03
ENSG00000171435	KSR2	1.67	1.67	0.74	1.56E-06	1.34E+03	1.34E+03
ENSG00000133256	PDE6B	1.72	1.72	0.78	1.65E-06	1.34E+03	1.34E+03
ENSG00000142687	KIAA0319L	1.45	1.45	0.54	1.18E-06	1.34E+03	1.34E+03
ENSG00000137133	HINT2	1.50	1.50	0.59	1.26E-06	1.34E+03	1.34E+03
ENSG00000198912	C1orf174	0.70	-1.44	-0.52	1.16E-06	-1.33E+03	1.33E+03
ENSG00000146147	MLIP	0.64	-1.57	-0.65	1.38E-06	-1.33E+03	1.33E+03
ENSG00000115556	PLCD4	0.54	-1.86	-0.90	1.96E-06	-1.33E+03	1.33E+03
ENSG00000126705	AHDC1	1.66	1.66	0.73	1.55E-06	1.33E+03	1.33E+03
ENSG00000244701	RP5-894A10.2	1.89	1.89	0.92	2.03E-06	1.33E+03	1.33E+03
ENSG00000179598	PLD6	0.50	-2.01	-1.01	2.32E-06	-1.32E+03	1.32E+03
ENSG00000176845	METRNL	1.50	1.50	0.59	1.31E-06	1.31E+03	1.31E+03
ENSG00000120253	NUP43	0.68	-1.47	-0.55	1.25E-06	-1.31E+03	1.31E+03
ENSG00000162889	MAPKAPK2	1.48	1.48	0.56	1.27E-06	1.31E+03	1.31E+03
ENSG00000165716	FAM69B	0.68	-1.47	-0.55	1.25E-06	-1.31E+03	1.31E+03
ENSG00000197714	ZNF460	1.45	1.45	0.54	1.25E-06	1.30E+03	1.30E+03
ENSG00000143815	LBR	0.68	-1.47	-0.56	1.28E-06	-1.30E+03	1.30E+03
ENSG00000137171	KLC4	1.49	1.49	0.58	1.33E-06	1.29E+03	1.29E+03
ENSG00000178980	SEPW1	0.67	-1.49	-0.57	1.32E-06	-1.29E+03	1.29E+03
ENSG00000101452	DHX35	0.68	-1.47	-0.56	1.31E-06	-1.29E+03	1.29E+03
ENSG00000197724	PHF2	0.65	-1.53	-0.61	1.41E-06	-1.28E+03	1.28E+03
ENSG00000120333	MRPS14	0.70	-1.43	-0.51	1.26E-06	-1.27E+03	1.27E+03
ENSG00000204220	PFDN6	0.67	-1.49	-0.58	1.37E-06	-1.27E+03	1.27E+03
ENSG00000116604	MEF2D	1.54	1.54	0.62	1.47E-06	1.27E+03	1.27E+03
ENSG00000007968	E2F2	0.59	-1.71	-0.77	1.80E-06	-1.27E+03	1.27E+03
ENSG00000178038	ALS2CL	1.59	1.59	0.67	1.60E-06	1.26E+03	1.26E+03
ENSG00000235501	RP4-639F20.1	1.91	1.91	0.93	2.31E-06	1.26E+03	1.26E+03
ENSG00000167107	ACSF2	0.67	-1.49	-0.57	1.41E-06	-1.25E+03	1.25E+03
ENSG00000173548	SNX33	1.43	1.43	0.52	1.32E-06	1.25E+03	1.25E+03
ENSG00000142599	RERE	1.47	1.47	0.55	1.38E-06	1.25E+03	1.25E+03
ENSG00000118689	FOXO3	1.45	1.45	0.53	1.36E-06	1.24E+03	1.24E+03
ENSG00000101311	FERMT1	0.52	-1.94	-0.95	2.44E-06	-1.24E+03	1.24E+03
ENSG00000071894	CPSF1	0.69	-1.45	-0.54	1.39E-06	-1.23E+03	1.23E+03
ENSG00000165644	COMTD1	0.56	-1.78	-0.83	2.09E-06	-1.23E+03	1.23E+03
ENSG00000182220	ATP6AP2	1.48	1.48	0.56	1.45E-06	1.23E+03	1.23E+03
ENSG00000073921	PICALM	1.50	1.50	0.59	1.51E-06	1.22E+03	1.22E+03
ENSG00000137054	POLR1E	0.69	-1.44	-0.53	1.41E-06	-1.22E+03	1.22E+03
ENSG00000159445	THEM4	0.69	-1.45	-0.53	1.41E-06	-1.22E+03	1.22E+03
ENSG00000087245	MMP2	1.58	1.58	0.66	1.70E-06	1.21E+03	1.21E+03
ENSG00000144959	NCEH1	0.65	-1.54	-0.62	1.62E-06	-1.21E+03	1.21E+03
ENSG00000117758	STX12	0.68	-1.47	-0.56	1.49E-06	-1.21E+03	1.21E+03
ENSG00000214189	ZNF788	0.66	-1.50	-0.59	1.56E-06	-1.21E+03	1.21E+03
ENSG00000074266	EED	0.69	-1.45	-0.54	1.45E-06	-1.21E+03	1.21E+03
ENSG00000135845	PIGC	0.69	-1.44	-0.53	1.43E-06	-1.21E+03	1.21E+03
ENSG00000124508	BTN2A2	0.68	-1.47	-0.56	1.50E-06	-1.20E+03	1.20E+03
ENSG00000269486	CTC-360G5.9	1.68	1.68	0.75	1.95E-06	1.20E+03	1.20E+03
ENSG00000156030	ELMSAN1	1.46	1.46	0.55	1.49E-06	1.20E+03	1.20E+03
ENSG00000147408	CSGALNACT1	1.45	1.45	0.53	1.48E-06	1.19E+03	1.19E+03
ENSG00000251669	FAM86EP	2.07	2.07	1.05	3.06E-06	1.18E+03	1.18E+03
ENSG00000006025	OSBPL7	0.64	-1.57	-0.65	1.77E-06	-1.18E+03	1.18E+03
ENSG00000245213	RP11-10K16.1	1.88	1.88	0.91	2.52E-06	1.18E+03	1.18E+03
ENSG00000198042	MAK16	0.70	-1.43	-0.52	1.48E-06	-1.18E+03	1.18E+03
ENSG00000120708	TGFBI	1.53	1.53	0.62	1.70E-06	1.18E+03	1.18E+03
ENSG00000175727	MLXIP	1.43	1.43	0.51	1.48E-06	1.18E+03	1.18E+03
ENSG00000152377	SPOCK1	1.55	1.55	0.63	1.73E-06	1.17E+03	1.17E+03
ENSG00000130748	TMEM160	0.60	-1.68	-0.75	2.05E-06	-1.17E+03	1.17E+03
ENSG00000006607	FARP2	0.66	-1.52	-0.61	1.68E-06	-1.17E+03	1.17E+03
ENSG00000164603	C7orf60	1.46	1.46	0.55	1.57E-06	1.17E+03	1.17E+03

ENSG00000116668	SWT1	1.61	1.61	0.68	1.90E-06	1.17E+03	1.17E+03
ENSG00000112559	MDFI	1.46	1.46	0.55	1.58E-06	1.17E+03	1.17E+03
ENSG00000113300	CNOT6	1.43	1.43	0.52	1.51E-06	1.17E+03	1.17E+03
ENSG00000186329	TMEM212	1.78	1.78	0.83	2.35E-06	1.16E+03	1.16E+03
ENSG00000105204	DYRK1B	1.69	1.69	0.75	2.12E-06	1.16E+03	1.16E+03
ENSG00000115053	NCL	0.68	-1.47	-0.55	1.63E-06	-1.15E+03	1.15E+03
ENSG00000198932	GPRASP1	0.50	-1.99	-0.99	3.00E-06	-1.15E+03	1.15E+03
ENSG00000132313	MRPL35	0.70	-1.43	-0.52	1.57E-06	-1.15E+03	1.15E+03
ENSG00000101265	RASSF2	1.48	1.48	0.56	1.67E-06	1.14E+03	1.14E+03
ENSG00000172765	TMCC1	1.44	1.44	0.53	1.59E-06	1.14E+03	1.14E+03
ENSG00000173281	PPP1R3B	1.45	1.45	0.54	1.63E-06	1.14E+03	1.14E+03
	CTD-3116E22.4	1.89	1.89	0.92	2.75E-06	1.14E+03	1.14E+03
ENSG00000135968	GCC2	1.69	1.69	0.75	2.20E-06	1.14E+03	1.14E+03
ENSG00000185215	TNFAIP2	1.47	1.47	0.56	1.68E-06	1.14E+03	1.14E+03
ENSG00000198954	KIAA1279	0.69	-1.46	-0.54	1.65E-06	-1.13E+03	1.13E+03
ENSG00000109436	TBC1D9	1.47	1.47	0.55	1.68E-06	1.13E+03	1.13E+03
ENSG00000132661	NXT1	0.69	-1.46	-0.54	1.65E-06	-1.13E+03	1.13E+03
ENSG00000162236	STX5	0.70	-1.43	-0.52	1.61E-06	-1.13E+03	1.13E+03
ENSG00000172922	RNASEH2C	0.68	-1.48	-0.56	1.74E-06	-1.12E+03	1.12E+03
ENSG00000235954	TTC28-AS1	1.45	1.45	0.53	1.67E-06	1.12E+03	1.12E+03
ENSG00000186666	BCDIN3D	1.63	1.63	0.71	2.16E-06	1.11E+03	1.11E+03
ENSG00000141425	RPRD1A	0.69	-1.46	-0.55	1.72E-06	-1.11E+03	1.11E+03
ENSG00000183763	TRAIP	0.61	-1.63	-0.71	2.18E-06	-1.11E+03	1.11E+03
ENSG00000152409	JMY	1.53	1.53	0.61	1.90E-06	1.11E+03	1.11E+03
ENSG00000113272	THG1L	0.64	-1.56	-0.64	2.00E-06	-1.10E+03	1.10E+03
ENSG00000117643	MAN1C1	0.56	-1.78	-0.83	2.65E-06	-1.10E+03	1.10E+03
ENSG00000138780	GSTCD	0.70	-1.44	-0.52	1.73E-06	-1.09E+03	1.09E+03
ENSG00000166145	SPINT1	1.85	1.85	0.89	2.87E-06	1.09E+03	1.09E+03
ENSG00000118200	CAMSAP2	1.61	1.61	0.69	2.18E-06	1.09E+03	1.09E+03
ENSG00000196584	XRCC2	0.69	-1.45	-0.54	1.79E-06	-1.08E+03	1.08E+03
ENSG00000151348	EXT2	1.46	1.46	0.55	1.83E-06	1.08E+03	1.08E+03
ENSG00000225921	NOL7	0.69	-1.44	-0.53	1.78E-06	-1.08E+03	1.08E+03
ENSG00000127564	PKMYT1	0.61	-1.64	-0.72	2.32E-06	-1.08E+03	1.08E+03
ENSG00000267242	AC069278.4	1.90	1.90	0.93	3.11E-06	1.08E+03	1.08E+03
ENSG00000065308	TRAM2	1.54	1.54	0.62	2.06E-06	1.07E+03	1.07E+03
ENSG00000158321	AUTS2	1.79	1.79	0.84	2.81E-06	1.07E+03	1.07E+03
ENSG00000142694	EVA1B	0.71	-1.42	-0.50	1.76E-06	-1.07E+03	1.07E+03
ENSG00000071462	WBSCR22	0.69	-1.45	-0.54	1.86E-06	-1.07E+03	1.07E+03
ENSG00000159842	ABR	1.55	1.55	0.63	2.13E-06	1.06E+03	1.06E+03
ENSG00000077097	TOP2B	1.57	1.57	0.65	2.18E-06	1.06E+03	1.06E+03
ENSG00000018408	WWTR1	1.46	1.46	0.55	1.89E-06	1.06E+03	1.06E+03
ENSG00000249348	UGDH-AS1	1.75	1.75	0.81	2.71E-06	1.06E+03	1.06E+03
ENSG00000137411	VARS2	1.43	1.43	0.51	1.81E-06	1.06E+03	1.06E+03
ENSG00000132541	HRSP12	1.45	1.45	0.54	1.88E-06	1.06E+03	1.06E+03
ENSG00000137496	IL18BP	0.57	-1.77	-0.82	2.81E-06	-1.06E+03	1.06E+03
ENSG00000100926	TM9SF1	0.70	-1.44	-0.52	1.86E-06	-1.05E+03	1.05E+03
ENSG00000160190	SLC37A1	1.54	1.54	0.62	2.15E-06	1.05E+03	1.05E+03
ENSG00000176974	SHMT1	0.70	-1.44	-0.52	1.88E-06	-1.05E+03	1.05E+03
ENSG00000164916	FOXK1	1.46	1.46	0.55	1.96E-06	1.05E+03	1.05E+03
ENSG00000173418	NAA20	0.68	-1.47	-0.55	1.97E-06	-1.04E+03	1.04E+03
ENSG00000165355	FBXO33	1.43	1.43	0.52	1.90E-06	1.04E+03	1.04E+03
ENSG00000126709	IFI6	0.53	-1.88	-0.91	3.27E-06	-1.04E+03	1.04E+03
ENSG00000008952	SEC62	1.54	1.54	0.62	2.21E-06	1.04E+03	1.04E+03
ENSG00000091409	ITGA6	1.47	1.47	0.55	2.00E-06	1.04E+03	1.04E+03
ENSG00000167460	TPM4	1.46	1.46	0.55	2.00E-06	1.04E+03	1.04E+03
ENSG00000153317	ASAP1	1.52	1.52	0.60	2.17E-06	1.03E+03	1.03E+03
ENSG00000091129	NRCAM	0.69	-1.46	-0.54	2.03E-06	-1.02E+03	1.02E+03
ENSG00000138166	DUSP5	1.48	1.48	0.57	2.11E-06	1.02E+03	1.02E+03
ENSG00000204217	BMPR2	1.59	1.59	0.67	2.44E-06	1.02E+03	1.02E+03
ENSG00000100036	SLC35E4	1.47	1.47	0.56	2.12E-06	1.01E+03	1.01E+03
ENSG00000155666	KDM8	0.57	-1.75	-0.81	3.00E-06	-1.01E+03	1.01E+03
ENSG00000171867	PRNP	1.46	1.46	0.54	2.08E-06	1.01E+03	1.01E+03
ENSG00000105497	ZNF175	1.43	1.43	0.51	2.00E-06	1.01E+03	1.01E+03
	AC004967.8	0.55	-1.83	-0.87	3.31E-06	-1.01E+03	1.01E+03
ENSG00000136940	PDCL	1.56	1.56	0.65	2.43E-06	1.00E+03	1.00E+03
ENSG00000115661	STK16	1.50	1.50	0.59	2.27E-06	9.96E+02	9.96E+02
ENSG00000173638	SLC19A1	0.67	-1.50	-0.59	2.28E-06	-9.95E+02	9.95E+02
ENSG00000254087	LYN	1.48	1.48	0.57	2.22E-06	9.94E+02	9.94E+02
ENSG00000150551	LYPD1	0.67	-1.49	-0.58	2.25E-06	-9.93E+02	9.93E+02
ENSG00000167088	SNRPD1	0.68	-1.48	-0.57	2.22E-06	-9.93E+02	9.93E+02
ENSG00000174574	AKIRIN1	1.46	1.46	0.55	2.17E-06	9.92E+02	9.92E+02
ENSG00000234614	AL450992.2	2.02	2.02	1.01	4.15E-06	9.89E+02	9.89E+02
ENSG00000159388	BTG2	1.47	1.47	0.56	2.22E-06	9.89E+02	9.89E+02
ENSG00000167984	NLRC3	1.91	1.91	0.93	3.75E-06	9.86E+02	9.86E+02
ENSG00000110852	CLEC2B	1.45	1.45	0.54	2.17E-06	9.86E+02	9.86E+02
ENSG00000182923	CEP63	1.48	1.48	0.56	2.26E-06	9.83E+02	9.83E+02
ENSG00000102763	VWA8	0.70	-1.44	-0.52	2.14E-06	-9.83E+02	9.83E+02
ENSG00000111364	DDX55	0.70	-1.44	-0.52	2.15E-06	-9.82E+02	9.82E+02
ENSG00000184497	FAM70B	1.49	1.49	0.57	2.30E-06	9.81E+02	9.81E+02

ENSG00000188735	TMEM120B	0.67	-1.50	-0.59	2.36E-06	-9.79E+02	9.79E+02
ENSG00000144843	ADPRH	0.60	-1.66	-0.73	2.91E-06	-9.73E+02	9.73E+02
ENSG00000198142	SOWAHC	1.60	1.60	0.68	2.72E-06	9.71E+02	9.71E+02
ENSG00000184056	VPS33B	0.70	-1.43	-0.52	2.18E-06	-9.70E+02	9.70E+02
ENSG00000179909	ZNF154	0.53	-1.88	-0.91	3.80E-06	-9.66E+02	9.66E+02
ENSG00000160783	PMF1	0.69	-1.46	-0.54	2.29E-06	-9.64E+02	9.64E+02
ENSG00000167779	IGFBP6	1.49	1.49	0.58	2.41E-06	9.63E+02	9.63E+02
ENSG00000212916	KIAA1383	1.57	1.57	0.65	2.64E-06	9.63E+02	9.63E+02
ENSG00000261236	BOP1	0.65	-1.53	-0.62	2.54E-06	-9.61E+02	9.61E+02
ENSG00000198324	FAM109A	1.50	1.50	0.58	2.46E-06	9.57E+02	9.57E+02
ENSG00000163633	C4orf36	1.58	1.58	0.66	2.73E-06	9.55E+02	9.55E+02
ENSG00000197943	PLCG2	0.65	-1.53	-0.61	2.56E-06	-9.55E+02	9.55E+02
ENSG00000179314	WSCD1	1.44	1.44	0.52	2.26E-06	9.55E+02	9.55E+02
ENSG00000165568	AKR1E2	1.59	1.59	0.67	2.77E-06	9.55E+02	9.55E+02
ENSG00000127511	SIN3B	1.46	1.46	0.54	2.33E-06	9.54E+02	9.54E+02
ENSG00000197451	HNRNPAB	0.68	-1.46	-0.55	2.35E-06	-9.54E+02	9.54E+02
ENSG00000130921	C12orf65	0.68	-1.47	-0.55	2.37E-06	-9.53E+02	9.53E+02
ENSG00000162377	SELRC1	0.68	-1.46	-0.55	2.37E-06	-9.50E+02	9.50E+02
ENSG00000070366	SMG6	1.54	1.54	0.62	2.65E-06	9.45E+02	9.45E+02
ENSG00000260267	RP11-452L6.5	1.50	1.50	0.58	2.53E-06	9.43E+02	9.43E+02
ENSG00000135269	TES	1.44	1.44	0.52	2.34E-06	9.39E+02	9.39E+02
ENSG00000092208	GEMIN2	0.68	-1.48	-0.57	2.49E-06	-9.38E+02	9.38E+02
ENSG00000198625	MDM4	0.69	-1.44	-0.53	2.37E-06	-9.38E+02	9.38E+02
ENSG00000172613	RAD9A	0.59	-1.70	-0.76	3.29E-06	-9.36E+02	9.36E+02
ENSG00000171206	TRIM8	1.44	1.44	0.53	2.37E-06	9.35E+02	9.35E+02
ENSG00000108312	UBTF	0.70	-1.43	-0.52	2.35E-06	-9.35E+02	9.35E+02
ENSG00000132846	ZBED3	0.58	-1.72	-0.78	3.43E-06	-9.29E+02	9.29E+02
ENSG00000044090	CUL7	1.44	1.44	0.53	2.41E-06	9.29E+02	9.29E+02
ENSG00000053770	AP5M1	1.50	1.50	0.59	2.64E-06	9.24E+02	9.24E+02
ENSG00000087299	L2HGDH	0.59	-1.69	-0.76	3.37E-06	-9.22E+02	9.22E+02
ENSG00000196872	KIAA1211L	1.55	1.55	0.63	2.86E-06	9.17E+02	9.17E+02
ENSG00000164758	MED30	0.68	-1.48	-0.56	2.60E-06	-9.16E+02	9.16E+02
ENSG00000261295	RP11-524D16__A.3	1.78	1.78	0.83	3.80E-06	9.14E+02	9.14E+02
ENSG00000162924	REL	1.57	1.57	0.65	2.94E-06	9.13E+02	9.13E+02
ENSG00000165458	INPPL1	1.45	1.45	0.53	2.52E-06	9.11E+02	9.11E+02
ENSG00000073584	SMARCE1	1.43	1.43	0.52	2.49E-06	9.09E+02	9.09E+02
ENSG00000130713	EXOSC2	0.70	-1.43	-0.52	2.53E-06	-9.02E+02	9.02E+02
ENSG00000118900	UBN1	1.46	1.46	0.55	2.65E-06	8.97E+02	8.97E+02
ENSG00000179364	PACS2	1.48	1.48	0.56	2.72E-06	8.96E+02	8.96E+02
ENSG00000158019	BRE	1.46	1.46	0.55	2.66E-06	8.96E+02	8.96E+02
ENSG00000011451	WIZ	1.45	1.45	0.54	2.62E-06	8.96E+02	8.96E+02
ENSG00000100299	ARSA	1.52	1.52	0.61	2.90E-06	8.94E+02	8.94E+02
ENSG00000081803	CADPS2	0.70	-1.43	-0.52	2.58E-06	-8.90E+02	8.90E+02
ENSG00000092201	SUPT16H	0.69	-1.45	-0.53	2.70E-06	-8.82E+02	8.82E+02
ENSG00000157111	TMEM171	1.56	1.56	0.65	3.17E-06	8.79E+02	8.79E+02
ENSG00000168938	PPIC	1.43	1.43	0.51	2.66E-06	8.76E+02	8.76E+02
ENSG00000162144	CYBASC3	1.44	1.44	0.53	2.72E-06	8.73E+02	8.73E+02
ENSG00000117616	C1orf63	0.70	-1.43	-0.52	2.69E-06	-8.73E+02	8.73E+02
ENSG00000172731	LRRC20	1.48	1.48	0.57	2.90E-06	8.72E+02	8.72E+02
ENSG00000198814	GK	0.66	-1.52	-0.61	3.05E-06	-8.72E+02	8.72E+02
ENSG00000111696	NT5DC3	0.68	-1.48	-0.57	2.90E-06	-8.69E+02	8.69E+02
ENSG00000156535	CD109	1.47	1.47	0.56	2.88E-06	8.68E+02	8.68E+02
ENSG00000132849	INADL	1.48	1.48	0.56	2.94E-06	8.62E+02	8.62E+02
ENSG00000185947	ZNF267	1.59	1.59	0.67	3.43E-06	8.58E+02	8.58E+02
ENSG00000104763	ASAH1	1.50	1.50	0.59	3.07E-06	8.56E+02	8.56E+02
ENSG00000169136	ATF5	0.67	-1.49	-0.57	3.07E-06	-8.50E+02	8.50E+02
ENSG00000148400	NOTCH1	1.55	1.55	0.64	3.35E-06	8.49E+02	8.49E+02
ENSG00000129195	FAM64A	0.69	-1.46	-0.54	2.95E-06	-8.48E+02	8.48E+02
ENSG00000125089	SH3TC1	1.44	1.44	0.53	2.95E-06	8.39E+02	8.39E+02
ENSG00000128973	CLN6	0.70	-1.42	-0.51	2.86E-06	-8.39E+02	8.39E+02
ENSG00000240849	TMEM189	1.46	1.46	0.55	3.06E-06	8.36E+02	8.36E+02
ENSG00000204954	C12orf73	1.65	1.65	0.72	3.91E-06	8.35E+02	8.35E+02
ENSG00000174705	SH3PXD2B	1.47	1.47	0.56	3.12E-06	8.34E+02	8.34E+02
ENSG00000089335	ZNF302	1.53	1.53	0.61	3.36E-06	8.33E+02	8.33E+02
ENSG00000107372	ZFAND5	1.45	1.45	0.54	3.05E-06	8.32E+02	8.32E+02
ENSG00000259673	IQCH-AS1	1.92	1.92	0.94	5.38E-06	8.30E+02	8.30E+02
ENSG00000151773	CCDC122	1.62	1.62	0.70	3.82E-06	8.29E+02	8.29E+02
ENSG00000139197	PEX5	1.43	1.43	0.51	2.98E-06	8.26E+02	8.26E+02
ENSG00000160799	CCDC12	1.41	1.41	0.50	2.93E-06	8.25E+02	8.25E+02
ENSG00000168765	GSTM4	0.49	-2.05	-1.04	6.19E-06	-8.25E+02	8.25E+02
ENSG00000179271	GADD45GIP1	0.71	-1.42	-0.50	3.00E-06	-8.18E+02	8.18E+02
ENSG00000121350	PYROXD1	1.52	1.52	0.60	3.51E-06	8.11E+02	8.11E+02
ENSG00000123219	CENPK	0.68	-1.48	-0.56	3.32E-06	-8.10E+02	8.10E+02
ENSG00000273899	NOL12	0.57	-1.77	-0.82	4.79E-06	-8.07E+02	8.07E+02
ENSG00000068724	TTC7A	1.44	1.44	0.53	3.19E-06	8.05E+02	8.05E+02
ENSG00000116198	CEP104	1.57	1.57	0.65	3.80E-06	8.05E+02	8.05E+02
ENSG00000263272	CTC-524C5.2	0.50	-1.99	-1.00	6.15E-06	-8.04E+02	8.04E+02
ENSG00000105287	PRKD2	1.42	1.42	0.51	3.16E-06	8.00E+02	8.00E+02
ENSG00000069020	MAST4	1.46	1.46	0.54	3.33E-06	7.98E+02	7.98E+02

ENSG00000233178	RP11-88I18.2	1.67	1.67	0.74	4.39E-06	7.97E+02	7.97E+02
ENSG00000164611	PTTG1	0.69	-1.45	-0.54	3.36E-06	-7.92E+02	7.92E+02
ENSG00000141994	DUS3L	0.68	-1.46	-0.55	3.41E-06	-7.91E+02	7.91E+02
ENSG00000034152	MAP2K3	1.45	1.45	0.54	3.40E-06	7.87E+02	7.87E+02
ENSG00000077092	RARB	0.68	-1.47	-0.56	3.50E-06	-7.86E+02	7.86E+02
ENSG00000173905	GOLIM4	1.46	1.46	0.55	3.46E-06	7.84E+02	7.84E+02
ENSG00000178921	PFAS	0.66	-1.52	-0.60	3.76E-06	-7.83E+02	7.83E+02
ENSG00000167220	HDHD2	0.68	-1.46	-0.55	3.50E-06	-7.82E+02	7.82E+02
ENSG00000108256	NUFIP2	1.46	1.46	0.54	3.48E-06	7.80E+02	7.80E+02
ENSG00000113522	RAD50	1.78	1.78	0.83	5.22E-06	7.80E+02	7.80E+02
ENSG00000248360	LINC00504	1.74	1.74	0.80	5.03E-06	7.76E+02	7.76E+02
ENSG00000168612	ZSWIM1	0.63	-1.60	-0.68	4.26E-06	-7.76E+02	7.76E+02
ENSG00000114491	UMPS	0.71	-1.42	-0.50	3.33E-06	-7.75E+02	7.75E+02
ENSG00000167601	AXL	0.65	-1.53	-0.61	3.88E-06	-7.75E+02	7.75E+02
ENSG00000148411	NACC2	1.43	1.43	0.52	3.43E-06	7.75E+02	7.75E+02
ENSG00000166340	TPP1	1.42	1.42	0.50	3.36E-06	7.74E+02	7.74E+02
ENSG00000161960	EIF4A1	0.62	-1.61	-0.69	4.35E-06	-7.73E+02	7.73E+02
ENSG00000161542	PRPSAP1	1.42	1.42	0.50	3.36E-06	7.72E+02	7.72E+02
ENSG00000242028	HYPK	1.65	1.65	0.72	4.56E-06	7.72E+02	7.72E+02
ENSG00000258199	RP11-977G19.5	0.55	-1.83	-0.87	5.66E-06	-7.71E+02	7.71E+02
ENSG00000184378	ACTRT3	0.55	-1.81	-0.85	5.51E-06	-7.70E+02	7.70E+02
ENSG00000152944	MED21	0.71	-1.42	-0.50	3.45E-06	-7.62E+02	7.62E+02
ENSG00000260855	RP11-439E19.10	2.16	2.16	1.11	8.11E-06	7.59E+02	7.59E+02
	C16orf85	1.80	1.80	0.85	5.65E-06	7.59E+02	7.59E+02
ENSG00000126247	CAPNS1	1.46	1.46	0.55	3.77E-06	7.51E+02	7.51E+02
ENSG00000116675	DNAJC6	0.63	-1.59	-0.67	4.52E-06	-7.48E+02	7.48E+02
ENSG00000000419	DPM1	0.70	-1.42	-0.51	3.65E-06	-7.45E+02	7.45E+02
ENSG00000143669	LYST	1.73	1.73	0.79	5.39E-06	7.45E+02	7.45E+02
ENSG00000102174	PHEX	1.54	1.54	0.62	4.31E-06	7.41E+02	7.41E+02
ENSG00000102606	ARHGEF7	1.44	1.44	0.53	3.89E-06	7.32E+02	7.32E+02
ENSG00000198707	CEP290	1.55	1.55	0.63	4.46E-06	7.32E+02	7.32E+02
ENSG00000198846	TOX	0.62	-1.61	-0.69	4.83E-06	-7.32E+02	7.32E+02
ENSG00000143127	ITGA10	0.70	-1.43	-0.52	3.83E-06	-7.31E+02	7.31E+02
ENSG00000120875	DUSP4	1.53	1.53	0.62	4.44E-06	7.27E+02	7.27E+02
ENSG00000006459	JHDM1D	1.42	1.42	0.51	3.82E-06	7.26E+02	7.26E+02
ENSG00000186010	NDUFA13	0.65	-1.53	-0.61	4.43E-06	-7.26E+02	7.26E+02
ENSG00000091428	RAPGEF4	0.63	-1.59	-0.67	4.90E-06	-7.17E+02	7.17E+02
ENSG00000154529	CNTNAP3B	0.69	-1.45	-0.54	4.11E-06	-7.16E+02	7.16E+02
ENSG00000184719	RNLS	0.55	-1.81	-0.86	6.39E-06	-7.16E+02	7.16E+02
ENSG00000188542	DUSP28	1.70	1.70	0.77	5.66E-06	7.15E+02	7.15E+02
ENSG00000170581	STAT2	0.71	-1.42	-0.50	3.93E-06	-7.14E+02	7.14E+02
ENSG00000112343	TRIM38	1.41	1.41	0.49	3.90E-06	7.13E+02	7.13E+02
ENSG00000147174	ACRC	1.74	1.74	0.80	5.96E-06	7.13E+02	7.13E+02
ENSG00000108479	GALK1	0.71	-1.40	-0.49	3.90E-06	-7.11E+02	7.11E+02
ENSG00000113657	DPYSL3	1.53	1.53	0.62	4.71E-06	7.07E+02	7.07E+02
ENSG00000141699	FAM134C	1.43	1.43	0.51	4.14E-06	7.02E+02	7.02E+02
ENSG00000128050	PAICS	0.67	-1.49	-0.57	4.50E-06	-7.00E+02	7.00E+02
ENSG00000164885	CDK5	1.41	1.41	0.50	4.07E-06	6.99E+02	6.99E+02
ENSG00000069943	PIGB	0.69	-1.45	-0.54	4.32E-06	-6.99E+02	6.99E+02
ENSG00000149761	NUDT22	0.70	-1.43	-0.52	4.21E-06	-6.97E+02	6.97E+02
ENSG00000078902	TOLLIP	1.40	1.40	0.49	4.07E-06	6.97E+02	6.97E+02
ENSG00000159479	MED8	0.71	-1.42	-0.50	4.17E-06	-6.93E+02	6.93E+02
ENSG00000179922	ZNF784	0.56	-1.79	-0.84	6.70E-06	-6.91E+02	6.91E+02
ENSG00000198865	CCDC152	0.61	-1.65	-0.72	5.69E-06	-6.91E+02	6.91E+02
ENSG00000148158	SNX30	1.43	1.43	0.51	4.31E-06	6.88E+02	6.88E+02
ENSG00000101236	RNF24	0.62	-1.60	-0.68	5.42E-06	-6.88E+02	6.88E+02
ENSG00000114988	LMAN2L	1.40	1.40	0.49	4.17E-06	6.87E+02	6.87E+02
ENSG00000164032	H2AFZ	0.68	-1.46	-0.55	4.53E-06	-6.86E+02	6.86E+02
ENSG00000183018	SPNS2	0.66	-1.52	-0.61	4.94E-06	-6.86E+02	6.86E+02
ENSG00000014123	UFL1	1.72	1.72	0.78	6.35E-06	6.81E+02	6.81E+02
ENSG00000275342	SGK223	1.59	1.59	0.67	5.52E-06	6.78E+02	6.78E+02
ENSG00000065534	MYLK	1.50	1.50	0.58	4.91E-06	6.76E+02	6.76E+02
ENSG00000198844	ARHGEF15	1.45	1.45	0.54	4.62E-06	6.76E+02	6.76E+02
ENSG00000104870	FCGRT	0.71	-1.41	-0.50	4.36E-06	-6.76E+02	6.76E+02
ENSG00000204946	ZNF783	0.64	-1.57	-0.65	5.37E-06	-6.76E+02	6.76E+02
ENSG00000102316	MAGED2	0.69	-1.45	-0.53	4.63E-06	-6.73E+02	6.73E+02
ENSG00000107099	DOCK8	1.45	1.45	0.54	4.68E-06	6.71E+02	6.71E+02
ENSG00000059728	MXD1	0.65	-1.54	-0.62	5.27E-06	-6.70E+02	6.70E+02
ENSG00000131778	CHD1L	0.70	-1.42	-0.51	4.54E-06	-6.68E+02	6.68E+02
ENSG00000177119	ANO6	1.45	1.45	0.54	4.76E-06	6.66E+02	6.66E+02
ENSG00000173198	CYSLTR1	1.92	1.92	0.94	8.31E-06	6.65E+02	6.65E+02
ENSG00000126945	HNRNPH2	0.68	-1.47	-0.56	4.92E-06	-6.64E+02	6.64E+02
ENSG00000166741	NNMT	0.69	-1.44	-0.53	4.71E-06	-6.64E+02	6.64E+02
ENSG00000182021	RP11-381O7.3	1.90	1.90	0.92	8.16E-06	6.64E+02	6.64E+02
ENSG00000127824	TUBA4A	0.52	-1.92	-0.94	8.35E-06	-6.63E+02	6.63E+02
ENSG00000110074	FOXRED1	0.68	-1.46	-0.55	4.94E-06	-6.58E+02	6.58E+02
ENSG00000105355	PLIN3	1.43	1.43	0.52	4.75E-06	6.56E+02	6.56E+02
ENSG00000251314	CTD-2337A12.1	1.86	1.86	0.90	8.04E-06	6.56E+02	6.56E+02
ENSG00000149798	CDC42EP2	1.57	1.57	0.65	5.71E-06	6.56E+02	6.56E+02

ENSG00000103121	CMC2	0.71	-1.40	-0.49	4.59E-06	-6.55E+02	6.55E+02
ENSG00000079432	CIC	1.54	1.54	0.63	5.59E-06	6.53E+02	6.53E+02
ENSG00000197863	ZNF790	1.62	1.62	0.70	6.21E-06	6.51E+02	6.51E+02
ENSG00000075914	EXOSC7	0.71	-1.40	-0.49	4.66E-06	-6.51E+02	6.51E+02
ENSG00000136807	CDK9	0.69	-1.46	-0.54	5.03E-06	-6.50E+02	6.50E+02
ENSG00000106477	CEP41	1.39	1.39	0.48	4.61E-06	6.50E+02	6.50E+02
ENSG00000196810	CTBP1-AS1	1.50	1.50	0.59	5.34E-06	6.50E+02	6.50E+02
ENSG00000139645	ANKRD52	1.41	1.41	0.50	4.74E-06	6.49E+02	6.49E+02
ENSG00000141002	TCF25	0.71	-1.42	-0.50	4.77E-06	-6.49E+02	6.49E+02
ENSG00000248275	CTC-338M12.3	0.61	-1.63	-0.71	6.35E-06	-6.47E+02	6.47E+02
ENSG00000108439	PNPO	0.71	-1.41	-0.50	4.85E-06	-6.41E+02	6.41E+02
ENSG00000126215	XRCC3	0.69	-1.46	-0.54	5.18E-06	-6.41E+02	6.41E+02
ENSG00000165704	HPRT1	0.59	-1.69	-0.76	7.08E-06	-6.36E+02	6.36E+02
ENSG00000137693	YAP1	0.61	-1.63	-0.71	6.64E-06	-6.34E+02	6.34E+02
ENSG00000077463	SIRT6	1.46	1.46	0.54	5.27E-06	6.34E+02	6.34E+02
ENSG00000129235	TXNDC17	0.67	-1.50	-0.58	5.58E-06	-6.33E+02	6.33E+02
ENSG00000169570	DTWD2	0.58	-1.71	-0.77	7.32E-06	-6.32E+02	6.32E+02
ENSG00000173327	MAP3K11	1.45	1.45	0.53	5.25E-06	6.31E+02	6.31E+02
ENSG00000225733	FGD5-AS1	0.70	-1.43	-0.52	5.18E-06	-6.28E+02	6.28E+02
ENSG00000132773	TOE1	0.71	-1.41	-0.50	5.10E-06	-6.25E+02	6.25E+02
ENSG00000163312	HELQ	1.43	1.43	0.52	5.24E-06	6.24E+02	6.24E+02
ENSG00000069966	GNB5	0.71	-1.42	-0.50	5.20E-06	-6.22E+02	6.22E+02
ENSG00000167861	HID1	1.44	1.44	0.53	5.40E-06	6.21E+02	6.21E+02
ENSG00000169139	UBE2V2	1.45	1.45	0.53	5.45E-06	6.19E+02	6.19E+02
ENSG00000186523	FAM86B1	2.03	2.03	1.02	1.07E-05	6.19E+02	6.19E+02
ENSG00000100575	TIMM9	0.70	-1.44	-0.52	5.41E-06	-6.18E+02	6.18E+02
ENSG00000112576	CCND3	0.71	-1.41	-0.50	5.23E-06	-6.17E+02	6.17E+02
ENSG00000059691	PET112	1.40	1.40	0.49	5.21E-06	6.15E+02	6.15E+02
ENSG00000140961	OSGIN1	1.57	1.57	0.65	6.62E-06	6.08E+02	6.08E+02
ENSG00000171827	ZNF570	1.56	1.56	0.64	6.56E-06	6.08E+02	6.08E+02
ENSG00000214530	STARD10	1.53	1.53	0.62	6.36E-06	6.07E+02	6.07E+02
ENSG00000083817	ZNF416	1.52	1.52	0.61	6.31E-06	6.07E+02	6.07E+02
ENSG00000166780	C16orf45	0.70	-1.42	-0.51	5.50E-06	-6.07E+02	6.07E+02
ENSG00000112851	ERBB2IP	1.60	1.60	0.68	6.97E-06	6.06E+02	6.06E+02
ENSG00000026025	VIM	1.53	1.53	0.61	6.51E-06	6.00E+02	6.00E+02
ENSG00000162614	NEXN	0.65	-1.54	-0.62	6.60E-06	-5.99E+02	5.99E+02
ENSG00000169359	SLC33A1	1.43	1.43	0.52	5.72E-06	5.99E+02	5.99E+02
ENSG00000135587	SMPD2	0.66	-1.51	-0.60	6.38E-06	-5.98E+02	5.98E+02
ENSG00000154262	ABCA6	0.65	-1.55	-0.63	6.70E-06	-5.97E+02	5.97E+02
ENSG00000125648	SLC25A23	1.44	1.44	0.53	5.86E-06	5.96E+02	5.96E+02
ENSG00000183255	PTTG1IP	1.43	1.43	0.52	5.81E-06	5.95E+02	5.95E+02
ENSG00000240356	RPL23AP7	1.56	1.56	0.64	6.98E-06	5.89E+02	5.89E+02
ENSG00000138738	PRDM5	0.64	-1.57	-0.65	7.10E-06	-5.88E+02	5.88E+02
ENSG00000153147	SMARCA5	1.47	1.47	0.56	6.26E-06	5.88E+02	5.88E+02
ENSG00000130734	ATG4D	1.47	1.47	0.56	6.30E-06	5.87E+02	5.87E+02
ENSG00000173465	SSSCA1	0.70	-1.43	-0.52	5.97E-06	-5.86E+02	5.86E+02
ENSG00000211455	STK38L	1.45	1.45	0.54	6.17E-06	5.84E+02	5.84E+02
ENSG00000198331	HYLS1	0.71	-1.41	-0.49	5.83E-06	-5.83E+02	5.83E+02
ENSG00000108622	ICAM2	0.69	-1.44	-0.53	6.15E-06	-5.82E+02	5.82E+02
ENSG00000135002	RFK	0.70	-1.44	-0.52	6.11E-06	-5.82E+02	5.82E+02
ENSG00000159131	GART	0.68	-1.48	-0.56	6.45E-06	-5.81E+02	5.81E+02
ENSG00000129473	BCL2L2	0.70	-1.42	-0.51	6.00E-06	-5.81E+02	5.81E+02
ENSG00000087191	PSMC5	0.67	-1.49	-0.58	6.61E-06	-5.80E+02	5.80E+02
ENSG00000037757	MRI1	0.71	-1.41	-0.50	5.98E-06	-5.77E+02	5.77E+02
ENSG00000181634	TNFSF15	0.71	-1.41	-0.50	6.01E-06	-5.76E+02	5.76E+02
ENSG00000092094	OSGEP	0.71	-1.41	-0.50	6.01E-06	-5.76E+02	5.76E+02
ENSG00000164403	SHROOM1	0.66	-1.52	-0.60	6.98E-06	-5.75E+02	5.75E+02
ENSG00000124831	LRRFIP1	1.43	1.43	0.52	6.24E-06	5.73E+02	5.73E+02
ENSG00000151422	FER	1.74	1.74	0.80	9.24E-06	5.73E+02	5.73E+02
ENSG00000132688	NES	1.44	1.44	0.53	6.36E-06	5.73E+02	5.73E+02
ENSG00000151413	NUBPL	1.43	1.43	0.51	6.26E-06	5.71E+02	5.71E+02
ENSG00000171067	C11orf24	1.43	1.43	0.51	6.29E-06	5.70E+02	5.70E+02
ENSG00000131080	EDA2R	1.46	1.46	0.55	6.62E-06	5.68E+02	5.68E+02
ENSG00000101546	RBFA	0.70	-1.43	-0.52	6.39E-06	-5.66E+02	5.66E+02
ENSG00000187164	KIAA1598	1.40	1.40	0.48	6.11E-06	5.66E+02	5.66E+02
ENSG00000145555	MYO10	1.55	1.55	0.63	7.63E-06	5.60E+02	5.60E+02
ENSG00000171988	JMJD1C	1.77	1.77	0.82	1.00E-05	5.58E+02	5.58E+02
ENSG00000121691	CAT	0.71	-1.41	-0.49	6.39E-06	-5.56E+02	5.56E+02
ENSG00000197275	RAD54B	0.60	-1.67	-0.74	9.03E-06	-5.55E+02	5.55E+02
ENSG00000196305	IARS	0.69	-1.44	-0.53	6.76E-06	-5.54E+02	5.54E+02
ENSG00000106355	LSM5	0.70	-1.42	-0.51	6.63E-06	-5.53E+02	5.53E+02
ENSG00000158402	CDC25C	0.69	-1.45	-0.54	6.93E-06	-5.52E+02	5.52E+02
ENSG00000162385	MAGOH	0.67	-1.50	-0.58	7.36E-06	-5.52E+02	5.52E+02
ENSG00000131238	PPT1	0.71	-1.42	-0.50	6.61E-06	-5.52E+02	5.52E+02
ENSG00000261468	RP11-1024P17.1	1.55	1.55	0.63	7.91E-06	5.51E+02	5.51E+02
ENSG00000163291	PAQR3	0.72	-1.39	-0.48	6.37E-06	-5.51E+02	5.51E+02
ENSG00000249087	C1orf213	1.64	1.64	0.71	8.82E-06	5.51E+02	5.51E+02
ENSG00000175110	MRPS22	0.72	-1.40	-0.48	6.50E-06	-5.48E+02	5.48E+02
ENSG00000146242	TPBG	1.49	1.49	0.58	7.46E-06	5.46E+02	5.46E+02

ENSG00000151612	ZNF827	1.53	1.53	0.61	7.83E-06	5.46E+02	5.46E+02
ENSG00000169891	REPS2	1.50	1.50	0.58	7.58E-06	5.45E+02	5.45E+02
ENSG00000166173	LARP6	1.42	1.42	0.51	6.85E-06	5.44E+02	5.44E+02
ENSG00000079462	PAFAH1B3	0.69	-1.45	-0.54	7.17E-06	-5.43E+02	5.43E+02
ENSG00000180998	GPR137C	1.80	1.80	0.85	1.11E-05	5.41E+02	5.41E+02
ENSG00000154102	C16orf74	1.42	1.42	0.51	6.93E-06	5.41E+02	5.41E+02
ENSG00000214941	ZSWIM7	1.46	1.46	0.54	7.26E-06	5.41E+02	5.41E+02
ENSG00000138686	BBS7	1.44	1.44	0.52	7.08E-06	5.39E+02	5.39E+02
ENSG00000120458	MSANTD2	0.71	-1.41	-0.50	6.84E-06	-5.39E+02	5.39E+02
ENSG00000124275	MTRR	0.70	-1.43	-0.52	7.10E-06	-5.37E+02	5.37E+02
ENSG00000188511	C22orf34	0.61	-1.64	-0.72	9.36E-06	-5.37E+02	5.37E+02
ENSG00000187239	FNBP1	0.70	-1.44	-0.52	7.20E-06	-5.36E+02	5.36E+02
ENSG00000196998	WDR45	1.43	1.43	0.52	7.13E-06	5.36E+02	5.36E+02
ENSG00000198682	PAPSS2	0.68	-1.46	-0.55	7.43E-06	-5.36E+02	5.36E+02
ENSG00000102034	ELF4	1.43	1.43	0.51	7.11E-06	5.35E+02	5.35E+02
ENSG00000147400	CETN2	0.68	-1.48	-0.56	7.67E-06	-5.34E+02	5.34E+02
ENSG00000198917	C9orf114	0.71	-1.41	-0.49	6.96E-06	-5.33E+02	5.33E+02
ENSG00000144554	FANCD2	0.71	-1.41	-0.50	7.04E-06	-5.32E+02	5.32E+02
ENSG00000174718	KIAA1551	1.54	1.54	0.62	8.38E-06	5.32E+02	5.32E+02
ENSG00000253873	PCDHGA11	1.75	1.75	0.81	1.09E-05	5.30E+02	5.30E+02
ENSG00000213085	CCDC19	1.92	1.92	0.94	1.32E-05	5.30E+02	5.30E+02
ENSG00000110435	PDHX	1.40	1.40	0.49	7.04E-06	5.30E+02	5.30E+02
ENSG00000198440	ZNF583	1.57	1.57	0.65	8.76E-06	5.30E+02	5.30E+02
ENSG00000118276	B4GALT6	1.41	1.41	0.50	7.09E-06	5.29E+02	5.29E+02
ENSG00000180304	OAZ2	0.61	-1.63	-0.70	9.50E-06	-5.28E+02	5.28E+02
ENSG00000167325	RRM1	0.70	-1.42	-0.51	7.27E-06	-5.28E+02	5.28E+02
ENSG00000217128	FNIP1	1.44	1.44	0.53	7.52E-06	5.26E+02	5.26E+02
ENSG00000109089	CDR2L	1.41	1.41	0.49	7.18E-06	5.25E+02	5.25E+02
ENSG00000123094	RASSF8	1.47	1.47	0.56	7.86E-06	5.25E+02	5.25E+02
ENSG00000125810	CD93	1.43	1.43	0.52	7.44E-06	5.25E+02	5.25E+02
ENSG00000167393	PPP2R3B	0.65	-1.53	-0.61	8.50E-06	-5.24E+02	5.24E+02
ENSG00000224470	ATXN1L	1.41	1.41	0.49	7.29E-06	5.22E+02	5.22E+02
ENSG00000184661	CDCA2	0.70	-1.44	-0.52	7.59E-06	-5.21E+02	5.21E+02
ENSG00000150510	FAM124A	0.65	-1.55	-0.63	8.82E-06	-5.21E+02	5.21E+02
ENSG00000105472	CLEC11A	0.64	-1.56	-0.64	9.01E-06	-5.18E+02	5.18E+02
ENSG00000156990	RPUSD3	0.70	-1.43	-0.52	7.80E-06	-5.12E+02	5.12E+02
ENSG00000077454	LRCH4	0.69	-1.44	-0.53	7.97E-06	-5.12E+02	5.12E+02
ENSG00000198695	MT-ND6	0.37	-2.69	-1.43	2.80E-05	-5.09E+02	5.09E+02
ENSG00000104332	SFRP1	0.66	-1.51	-0.59	8.81E-06	-5.07E+02	5.07E+02
ENSG00000123472	ATPAF1	0.72	-1.39	-0.48	7.61E-06	-5.06E+02	5.06E+02
ENSG00000197694	SPTAN1	1.43	1.43	0.52	8.14E-06	5.01E+02	5.01E+02
ENSG00000125875	TBC1D20	1.38	1.38	0.47	7.66E-06	5.00E+02	5.00E+02
ENSG00000171604	CXXC5	0.53	-1.87	-0.90	1.42E-05	-4.98E+02	4.98E+02
ENSG00000166347	CYB5A	0.72	-1.40	-0.48	7.87E-06	-4.98E+02	4.98E+02
ENSG00000225830	ERCC6	0.72	-1.39	-0.47	7.77E-06	-4.97E+02	4.97E+02
ENSG00000100744	GSKIP	1.51	1.51	0.60	9.24E-06	4.97E+02	4.97E+02
ENSG00000138778	CENPE	0.66	-1.52	-0.61	9.41E-06	-4.96E+02	4.96E+02
ENSG00000004139	SARM1	1.63	1.63	0.71	1.10E-05	4.91E+02	4.91E+02
ENSG00000135763	URB2	0.71	-1.41	-0.49	8.23E-06	-4.90E+02	4.90E+02
ENSG00000215424	MCM3AP-AS1	0.56	-1.80	-0.84	1.35E-05	-4.89E+02	4.89E+02
ENSG00000173041	ZNF680	0.70	-1.42	-0.51	8.55E-06	-4.87E+02	4.87E+02
ENSG00000138074	SLC5A6	0.72	-1.40	-0.48	8.29E-06	-4.85E+02	4.85E+02
ENSG00000183137	CEP57L1	0.69	-1.44	-0.53	8.88E-06	-4.84E+02	4.84E+02
ENSG00000119227	PIGZ	0.52	-1.92	-0.94	1.60E-05	-4.80E+02	4.80E+02
ENSG00000106799	TGFBR1	1.40	1.40	0.48	8.55E-06	4.79E+02	4.79E+02
ENSG00000182742	HOXB4	1.41	1.41	0.50	8.82E-06	4.76E+02	4.76E+02
ENSG00000149289	ZC3H12C	1.42	1.42	0.50	8.93E-06	4.74E+02	4.74E+02
ENSG00000089006	SNX5	0.71	-1.41	-0.50	8.99E-06	-4.71E+02	4.71E+02
ENSG00000107679	PLEKHA1	1.42	1.42	0.51	9.12E-06	4.71E+02	4.71E+02
ENSG00000011105	TSPAN9	1.44	1.44	0.52	9.30E-06	4.71E+02	4.71E+02
ENSG00000183172	C22orf32	0.55	-1.82	-0.86	1.50E-05	-4.70E+02	4.70E+02
ENSG00000115163	CENPA	0.71	-1.40	-0.49	8.95E-06	-4.69E+02	4.69E+02
ENSG00000213619	NDUFS3	0.72	-1.39	-0.48	8.90E-06	-4.67E+02	4.67E+02
ENSG00000119139	TJP2	1.47	1.47	0.56	9.97E-06	4.65E+02	4.65E+02
ENSG00000112773	FAM46A	1.68	1.68	0.75	1.31E-05	4.63E+02	4.63E+02
ENSG00000162909	CAPN2	1.43	1.43	0.52	9.59E-06	4.62E+02	4.62E+02
ENSG00000198855	FICD	0.65	-1.54	-0.63	1.12E-05	-4.61E+02	4.61E+02
ENSG00000165338	HECTD2	1.65	1.65	0.73	1.29E-05	4.60E+02	4.60E+02
ENSG00000184903	IMMP2L	0.62	-1.61	-0.69	1.23E-05	-4.59E+02	4.59E+02
ENSG00000196511	TPK1	0.70	-1.42	-0.51	9.58E-06	-4.59E+02	4.59E+02
ENSG00000146373	RNF217	1.53	1.53	0.61	1.12E-05	4.57E+02	4.57E+02
ENSG00000104872	PIH1D1	0.72	-1.39	-0.48	9.32E-06	-4.56E+02	4.56E+02
ENSG00000185621	LMLN	0.58	-1.74	-0.80	1.45E-05	-4.56E+02	4.56E+02
ENSG00000146729	GBAS	0.71	-1.40	-0.48	9.45E-06	-4.55E+02	4.55E+02
ENSG00000158113	LRRC43	1.85	1.85	0.88	1.66E-05	4.53E+02	4.53E+02
ENSG00000103249	CLCN7	1.48	1.48	0.57	1.08E-05	4.52E+02	4.52E+02
ENSG00000184557	SOCS3	0.71	-1.41	-0.50	9.81E-06	-4.50E+02	4.50E+02
ENSG00000270141	TERC	0.70	-1.43	-0.52	1.01E-05	-4.50E+02	4.50E+02
ENSG00000103502	CDIPT	0.72	-1.39	-0.48	9.56E-06	-4.50E+02	4.50E+02

ENSG00000163607	GTPBP8	0.71	-1.41	-0.49	9.94E-06	-4.46E+02	4.46E+02
ENSG00000170558	CDH2	1.43	1.43	0.52	1.03E-05	4.46E+02	4.46E+02
ENSG00000167315	ACAA2	1.40	1.40	0.48	9.80E-06	4.46E+02	4.46E+02
ENSG00000155957	TMBIM4	0.71	-1.42	-0.50	1.01E-05	-4.45E+02	4.45E+02
ENSG00000087008	ACOX3	0.71	-1.42	-0.50	1.02E-05	-4.43E+02	4.43E+02
ENSG00000158691	ZSCAN12	0.67	-1.48	-0.57	1.13E-05	-4.43E+02	4.43E+02
ENSG00000114374	USP9Y	1.77	1.77	0.83	1.62E-05	4.41E+02	4.41E+02
ENSG00000213903	LTB4R	1.66	1.66	0.73	1.42E-05	4.40E+02	4.40E+02
ENSG00000186638	KIF24	0.67	-1.49	-0.58	1.14E-05	-4.40E+02	4.40E+02
ENSG00000119862	LGALSL	1.46	1.46	0.55	1.11E-05	4.40E+02	4.40E+02
ENSG00000088836	SLC4A11	1.55	1.55	0.63	1.24E-05	4.40E+02	4.40E+02
ENSG00000141642	ELAC1	1.46	1.46	0.55	1.10E-05	4.40E+02	4.40E+02
ENSG00000130038	EFCAB4B	0.68	-1.46	-0.55	1.11E-05	-4.39E+02	4.39E+02
ENSG00000146670	CDCA5	0.71	-1.41	-0.50	1.04E-05	-4.38E+02	4.38E+02
ENSG00000102870	ZNF629	0.68	-1.47	-0.55	1.15E-05	-4.34E+02	4.34E+02
ENSG00000103111	MON1B	1.47	1.47	0.56	1.15E-05	4.34E+02	4.34E+02
ENSG00000213347	MXD3	0.67	-1.48	-0.57	1.17E-05	-4.34E+02	4.34E+02
ENSG00000130772	MED18	1.46	1.46	0.55	1.14E-05	4.33E+02	4.33E+02
ENSG00000168010	ATG16L2	0.64	-1.56	-0.64	1.30E-05	-4.33E+02	4.33E+02
ENSG00000182957	SPATA13	0.69	-1.45	-0.54	1.13E-05	-4.32E+02	4.32E+02
ENSG00000162437	RAVER2	0.72	-1.39	-0.47	1.03E-05	-4.32E+02	4.32E+02
ENSG00000101974	ATP11C	1.55	1.55	0.63	1.29E-05	4.32E+02	4.32E+02
ENSG00000131389	SLC6A6	1.42	1.42	0.51	1.10E-05	4.30E+02	4.30E+02
ENSG00000273319	AC058791.1	1.65	1.65	0.72	1.48E-05	4.28E+02	4.28E+02
ENSG00000113742	CPEB4	1.41	1.41	0.49	1.08E-05	4.28E+02	4.28E+02
ENSG00000138162	TACC2	1.71	1.71	0.77	1.60E-05	4.27E+02	4.27E+02
ENSG00000121542	SEC22A	0.70	-1.43	-0.51	1.12E-05	-4.27E+02	4.27E+02
ENSG00000057019	DCBLD2	1.41	1.41	0.50	1.10E-05	4.27E+02	4.27E+02
ENSG00000003509	NDUFAF7	0.72	-1.39	-0.47	1.07E-05	-4.24E+02	4.24E+02
ENSG00000245680	ZNF585B	1.47	1.47	0.55	1.20E-05	4.24E+02	4.24E+02
ENSG00000116237	ICMT	0.71	-1.42	-0.50	1.13E-05	-4.21E+02	4.21E+02
ENSG00000154175	ABI3BP	0.67	-1.48	-0.57	1.25E-05	-4.20E+02	4.20E+02
ENSG00000075539	FRYL	1.70	1.70	0.76	1.64E-05	4.19E+02	4.19E+02
ENSG00000161048	NAPEPLD	0.64	-1.56	-0.64	1.39E-05	-4.19E+02	4.19E+02
ENSG00000103051	COG4	1.43	1.43	0.51	1.16E-05	4.19E+02	4.19E+02
ENSG00000134056	MRPS36	0.70	-1.42	-0.51	1.16E-05	-4.19E+02	4.19E+02
ENSG00000198585	NUDT16	1.43	1.43	0.51	1.16E-05	4.19E+02	4.19E+02
ENSG00000101868	POLA1	0.71	-1.40	-0.49	1.13E-05	-4.18E+02	4.18E+02
ENSG00000156218	ADAMTSL3	1.69	1.69	0.75	1.64E-05	4.16E+02	4.16E+02
ENSG00000140859	KIFC3	1.39	1.39	0.47	1.13E-05	4.14E+02	4.14E+02
ENSG00000261490	RP11-448G15.3	1.59	1.59	0.67	1.48E-05	4.13E+02	4.13E+02
ENSG00000112769	LAMA4	1.42	1.42	0.50	1.17E-05	4.13E+02	4.13E+02
ENSG00000123992	DNPEP	0.73	-1.38	-0.46	1.11E-05	-4.13E+02	4.13E+02
ENSG00000171960	PPIH	0.73	-1.38	-0.46	1.12E-05	-4.12E+02	4.12E+02
ENSG00000107331	ABCA2	0.64	-1.57	-0.65	1.45E-05	-4.12E+02	4.12E+02
ENSG00000145743	FBXL17	1.41	1.41	0.50	1.18E-05	4.12E+02	4.12E+02
ENSG00000162390	ACOT11	0.63	-1.59	-0.67	1.51E-05	-4.11E+02	4.11E+02
ENSG00000134748	PRPF38A	0.73	-1.38	-0.46	1.13E-05	-4.09E+02	4.09E+02
ENSG00000109534	GAR1	0.65	-1.54	-0.62	1.42E-05	-4.09E+02	4.09E+02
ENSG00000167972	ABCA3	1.52	1.52	0.61	1.39E-05	4.08E+02	4.08E+02
ENSG00000064042	LIMCH1	0.72	-1.39	-0.48	1.21E-05	-4.00E+02	4.00E+02
ENSG00000149636	DSN1	0.72	-1.39	-0.48	1.22E-05	-4.00E+02	4.00E+02
ENSG00000104835	SARS2	0.61	-1.63	-0.71	1.68E-05	-3.99E+02	3.99E+02
ENSG00000149564	ESAM	1.43	1.43	0.52	1.31E-05	3.96E+02	3.96E+02
ENSG00000053438	NNAT	1.62	1.62	0.69	1.68E-05	3.94E+02	3.94E+02
ENSG00000269386	RAB11B-AS1	1.50	1.50	0.59	1.46E-05	3.93E+02	3.93E+02
ENSG00000118579	MED28	0.68	-1.47	-0.56	1.41E-05	-3.92E+02	3.92E+02
ENSG00000182307	C8orf33	0.72	-1.39	-0.47	1.26E-05	-3.91E+02	3.91E+02
ENSG00000119403	PHF19	0.70	-1.42	-0.51	1.33E-05	-3.90E+02	3.90E+02
ENSG00000198947	DMD	0.68	-1.48	-0.57	1.45E-05	-3.88E+02	3.88E+02
ENSG00000101751	POLI	1.45	1.45	0.53	1.40E-05	3.88E+02	3.88E+02
ENSG00000083223	ZCCHC6	1.58	1.58	0.66	1.66E-05	3.87E+02	3.87E+02
ENSG00000147536	GINS4	0.72	-1.38	-0.47	1.28E-05	-3.86E+02	3.86E+02
ENSG00000141447	OSBPL1A	0.70	-1.43	-0.51	1.39E-05	-3.83E+02	3.83E+02
ENSG00000101040	ZMYND8	1.42	1.42	0.51	1.38E-05	3.82E+02	3.82E+02
ENSG00000257122	RRN3P3	0.64	-1.55	-0.63	1.66E-05	-3.81E+02	3.81E+02
ENSG00000157191	NECAP2	1.40	1.40	0.49	1.36E-05	3.80E+02	3.80E+02
ENSG00000067992	PDK3	0.66	-1.52	-0.61	1.62E-05	-3.79E+02	3.79E+02
ENSG00000082781	ITGB5	1.41	1.41	0.49	1.38E-05	3.79E+02	3.79E+02
ENSG00000169131	ZNF354A	1.41	1.41	0.49	1.40E-05	3.76E+02	3.76E+02
ENSG00000161714	PLCD3	1.46	1.46	0.54	1.50E-05	3.76E+02	3.76E+02
ENSG00000164609	SLU7	1.39	1.39	0.48	1.38E-05	3.75E+02	3.75E+02
ENSG00000173040	EVC2	1.52	1.52	0.60	1.65E-05	3.75E+02	3.75E+02
ENSG00000196150	ZNF250	1.42	1.42	0.51	1.45E-05	3.75E+02	3.75E+02
ENSG00000141644	MBD1	0.72	-1.38	-0.46	1.36E-05	-3.74E+02	3.74E+02
ENSG00000121940	CLCC1	0.70	-1.42	-0.51	1.45E-05	-3.74E+02	3.74E+02
	RP11-325F22.3	1.89	1.89	0.92	2.56E-05	3.74E+02	3.74E+02
ENSG00000136147	PHF11	0.66	-1.51	-0.59	1.64E-05	-3.73E+02	3.73E+02
ENSG00000011258	MBTD1	1.40	1.40	0.49	1.45E-05	3.69E+02	3.69E+02

ENSG00000107672	NSMCE4A	0.72	-1.39	-0.47	1.41E-05	-3.69E+02	3.69E+02
ENSG00000157881	PANK4	0.72	-1.39	-0.47	1.43E-05	-3.67E+02	3.67E+02
ENSG00000183718	TRIM52	1.60	1.60	0.68	1.91E-05	3.66E+02	3.66E+02
ENSG00000133195	SLC39A11	1.50	1.50	0.58	1.69E-05	3.65E+02	3.65E+02
ENSG00000066135	KDM4A	0.71	-1.40	-0.48	1.47E-05	-3.65E+02	3.65E+02
ENSG00000101000	PROCR	0.70	-1.42	-0.51	1.53E-05	-3.64E+02	3.64E+02
ENSG00000169967	MAP3K2	1.44	1.44	0.53	1.58E-05	3.63E+02	3.63E+02
ENSG00000134152	KATNBL1	0.72	-1.39	-0.48	1.47E-05	-3.63E+02	3.63E+02
ENSG00000130827	PLXNA3	0.67	-1.49	-0.57	1.69E-05	-3.62E+02	3.62E+02
ENSG00000172270	BSG	1.56	1.56	0.64	1.85E-05	3.62E+02	3.62E+02
ENSG00000120334	CENPL	0.73	-1.37	-0.46	1.45E-05	-3.61E+02	3.61E+02
ENSG00000101333	PLCB4	0.48	-2.07	-1.05	3.28E-05	-3.61E+02	3.61E+02
ENSG00000179409	GEMIN4	0.66	-1.52	-0.60	1.77E-05	-3.61E+02	3.61E+02
ENSG00000213085	CCDC75	0.72	-1.40	-0.48	1.51E-05	-3.59E+02	3.59E+02
ENSG00000188211	NCR3LG1	0.70	-1.42	-0.51	1.57E-05	-3.59E+02	3.59E+02
ENSG00000175606	TMEM70	0.70	-1.43	-0.51	1.58E-05	-3.59E+02	3.59E+02
ENSG00000165389	SPTSSA	0.64	-1.56	-0.64	1.91E-05	-3.57E+02	3.57E+02
ENSG00000108797	CNTNAP1	0.64	-1.56	-0.64	1.93E-05	-3.56E+02	3.56E+02
ENSG00000146426	TIAM2	0.66	-1.51	-0.60	1.82E-05	-3.54E+02	3.54E+02
ENSG00000137312	FLOT1	0.72	-1.38	-0.47	1.53E-05	-3.53E+02	3.53E+02
ENSG00000119777	TMEM214	0.72	-1.40	-0.48	1.57E-05	-3.53E+02	3.53E+02
ENSG00000183850	ZNF730	1.72	1.72	0.78	2.40E-05	3.50E+02	3.50E+02
ENSG00000182141	ZNF708	1.53	1.53	0.61	1.90E-05	3.50E+02	3.50E+02
ENSG00000108448	TRIM16L	1.53	1.53	0.61	1.91E-05	3.49E+02	3.49E+02
ENSG00000188026	RILPL1	1.43	1.43	0.51	1.67E-05	3.49E+02	3.49E+02
ENSG00000163867	ZMYM6	1.41	1.41	0.50	1.65E-05	3.48E+02	3.48E+02
ENSG00000140307	GTF2A2	0.71	-1.40	-0.49	1.64E-05	-3.46E+02	3.46E+02
ENSG00000102935	ZNF423	0.58	-1.73	-0.79	2.50E-05	-3.45E+02	3.45E+02
ENSG00000229056	AC020571.3	2.20	2.20	1.14	4.06E-05	3.45E+02	3.45E+02
ENSG00000160199	PKNOX1	1.40	1.40	0.49	1.65E-05	3.45E+02	3.45E+02
ENSG00000197070	ARRDC1	0.66	-1.51	-0.59	1.92E-05	-3.44E+02	3.44E+02
ENSG00000109445	ZNF330	0.71	-1.40	-0.49	1.68E-05	-3.43E+02	3.43E+02
ENSG00000101160	CTSZ	1.40	1.40	0.48	1.70E-05	3.39E+02	3.39E+02
ENSG00000071967	CYBRD1	1.41	1.41	0.49	1.72E-05	3.38E+02	3.38E+02
ENSG00000166086	JAM3	1.43	1.43	0.51	1.79E-05	3.37E+02	3.37E+02
ENSG00000127663	KDM4B	1.38	1.38	0.47	1.68E-05	3.36E+02	3.36E+02
ENSG00000244968	LIFR-AS1	1.76	1.76	0.81	2.73E-05	3.36E+02	3.36E+02
ENSG00000176624	MEX3C	1.41	1.41	0.49	1.75E-05	3.36E+02	3.36E+02
ENSG00000145337	PYURF	0.69	-1.44	-0.53	1.87E-05	-3.34E+02	3.34E+02
ENSG00000165171	WBSCR27	1.76	1.76	0.81	2.79E-05	3.33E+02	3.33E+02
ENSG00000167548	MLL2	1.42	1.42	0.50	1.82E-05	3.32E+02	3.32E+02
ENSG00000163322	FAM175A	0.62	-1.61	-0.68	2.35E-05	-3.32E+02	3.32E+02
ENSG00000143641	GALNT2	1.41	1.41	0.50	1.82E-05	3.31E+02	3.31E+02
ENSG00000159921	GNE	0.73	-1.37	-0.45	1.72E-05	-3.30E+02	3.30E+02
ENSG00000163611	SPICE1	0.71	-1.40	-0.49	1.82E-05	-3.28E+02	3.28E+02
ENSG00000165861	ZFYVE1	1.37	1.37	0.45	1.75E-05	3.28E+02	3.28E+02
ENSG00000198720	ANKRD13B	1.49	1.49	0.58	2.07E-05	3.28E+02	3.28E+02
ENSG00000178904	DPY19L3	0.71	-1.40	-0.49	1.83E-05	-3.28E+02	3.28E+02
ENSG00000110497	AMBRA1	1.42	1.42	0.51	1.89E-05	3.27E+02	3.27E+02
ENSG00000164031	DNAJB14	0.66	-1.52	-0.61	2.17E-05	-3.27E+02	3.27E+02
ENSG00000135622	SEMA4F	0.66	-1.52	-0.61	2.19E-05	-3.26E+02	3.26E+02
ENSG00000111196	MAGOHB	0.70	-1.43	-0.52	1.94E-05	-3.26E+02	3.26E+02
ENSG00000131634	TMEM204	1.51	1.51	0.60	2.16E-05	3.25E+02	3.25E+02
ENSG00000049656	CLPTM1L	0.71	-1.40	-0.49	1.87E-05	-3.24E+02	3.24E+02
ENSG00000261801	LOXL1-AS1	0.56	-1.79	-0.84	3.04E-05	-3.24E+02	3.24E+02
ENSG00000138092	CENPO	0.71	-1.42	-0.50	1.91E-05	-3.24E+02	3.24E+02
ENSG00000132718	SYT11	0.72	-1.39	-0.47	1.85E-05	-3.22E+02	3.22E+02
ENSG00000124749	COL21A1	0.59	-1.70	-0.76	2.77E-05	-3.22E+02	3.22E+02
ENSG00000129353	SLC44A2	1.59	1.59	0.67	2.45E-05	3.21E+02	3.21E+02
ENSG00000269821	KCNQ1OT1	1.39	1.39	0.48	1.88E-05	3.21E+02	3.21E+02
ENSG00000187535	IFT140	1.43	1.43	0.51	1.98E-05	3.21E+02	3.21E+02
ENSG00000089289	IGBP1	1.38	1.38	0.46	1.84E-05	3.20E+02	3.20E+02
ENSG00000136636	KCTD3	0.70	-1.43	-0.52	2.02E-05	-3.18E+02	3.18E+02
ENSG00000005893	LAMP2	1.41	1.41	0.50	1.96E-05	3.18E+02	3.18E+02
ENSG00000165996	PTPLA	0.70	-1.42	-0.51	2.00E-05	-3.18E+02	3.18E+02
ENSG00000159873	CCDC117	0.72	-1.39	-0.47	1.91E-05	-3.18E+02	3.18E+02
ENSG00000148677	ANKRD1	0.71	-1.40	-0.49	1.96E-05	-3.17E+02	3.17E+02
ENSG00000092036	HAUS4	0.71	-1.41	-0.50	1.98E-05	-3.17E+02	3.17E+02
ENSG00000185485	SDHAP1	0.60	-1.67	-0.74	2.78E-05	-3.16E+02	3.16E+02
ENSG00000102543	CDADC1	1.49	1.49	0.58	2.24E-05	3.16E+02	3.16E+02
ENSG00000213445	SIPA1	1.38	1.38	0.46	1.90E-05	3.16E+02	3.16E+02
ENSG00000177700	POLR2L	0.71	-1.41	-0.49	1.99E-05	-3.15E+02	3.15E+02
ENSG00000168874	ATOH8	1.41	1.41	0.50	2.02E-05	3.15E+02	3.15E+02
ENSG00000019144	PHLDB1	1.40	1.40	0.49	2.01E-05	3.12E+02	3.12E+02
ENSG00000165891	E2F7	1.39	1.39	0.48	1.99E-05	3.12E+02	3.12E+02
ENSG00000119723	COQ6	0.71	-1.41	-0.49	2.03E-05	-3.12E+02	3.12E+02
ENSG00000126602	TRAP1	0.68	-1.48	-0.56	2.24E-05	-3.12E+02	3.12E+02
ENSG00000134363	FST	0.56	-1.77	-0.83	3.26E-05	-3.11E+02	3.11E+02
ENSG00000177125	ZBTB34	1.38	1.38	0.46	1.97E-05	3.10E+02	3.10E+02

ENSG00000164048	ZNF589	0.71	-1.40	-0.49	2.05E-05	-3.10E+02	3.10E+02
ENSG00000164086	DUSP7	1.39	1.39	0.48	2.02E-05	3.10E+02	3.10E+02
ENSG00000254635	WAC-AS1	0.71	-1.41	-0.50	2.09E-05	-3.09E+02	3.09E+02
ENSG00000101447	FAM83D	0.70	-1.43	-0.52	2.18E-05	-3.07E+02	3.07E+02
ENSG00000188313	PLSCR1	0.73	-1.37	-0.46	2.00E-05	-3.07E+02	3.07E+02
ENSG00000140326	CDAN1	0.71	-1.40	-0.49	2.09E-05	-3.07E+02	3.07E+02
ENSG00000205413	SAMD9	1.43	1.43	0.51	2.17E-05	3.07E+02	3.07E+02
ENSG00000125746	EML2	0.68	-1.47	-0.56	2.30E-05	-3.07E+02	3.07E+02
ENSG00000105329	TGFB1	1.42	1.42	0.51	2.16E-05	3.06E+02	3.06E+02
ENSG00000166225	FRS2	1.40	1.40	0.48	2.08E-05	3.06E+02	3.06E+02
ENSG00000159784	FAM131B	1.81	1.81	0.86	3.52E-05	3.05E+02	3.05E+02
ENSG00000164949	GEM	1.74	1.74	0.80	3.24E-05	3.05E+02	3.05E+02
ENSG00000176022	B3GALT6	1.38	1.38	0.47	2.07E-05	3.04E+02	3.04E+02
ENSG00000143624	INTS3	1.44	1.44	0.53	2.25E-05	3.04E+02	3.04E+02
ENSG00000186205	MARC1	0.54	-1.87	-0.90	3.77E-05	-3.04E+02	3.04E+02
ENSG00000150403	TMCO3	1.39	1.39	0.48	2.10E-05	3.04E+02	3.04E+02
ENSG00000115904	SOS1	1.38	1.38	0.46	2.05E-05	3.04E+02	3.04E+02
ENSG00000173706	HEG1	1.50	1.50	0.58	2.44E-05	3.03E+02	3.03E+02
ENSG00000187626	ZKSCAN4	0.66	-1.51	-0.60	2.50E-05	-3.03E+02	3.03E+02
ENSG00000023287	RB1CC1	1.71	1.71	0.77	3.22E-05	3.02E+02	3.02E+02
ENSG00000131370	SH3BP5	1.40	1.40	0.49	2.16E-05	3.01E+02	3.01E+02
ENSG00000090432	MUL1	1.38	1.38	0.46	2.09E-05	3.01E+02	3.01E+02
ENSG00000163918	RFC4	0.72	-1.38	-0.47	2.11E-05	-3.01E+02	3.01E+02
ENSG00000171155	C1GALT1C1	0.72	-1.38	-0.47	2.13E-05	-3.00E+02	3.00E+02
ENSG00000177076	ACER2	0.67	-1.50	-0.59	2.52E-05	-2.99E+02	2.99E+02
ENSG00000171056	SOX7	1.41	1.41	0.50	2.24E-05	2.98E+02	2.98E+02
ENSG00000204131	NHSL2	1.41	1.41	0.50	2.25E-05	2.98E+02	2.98E+02
ENSG00000068878	PSME4	1.42	1.42	0.50	2.27E-05	2.98E+02	2.98E+02
ENSG00000196422	PPP1R26	1.39	1.39	0.47	2.19E-05	2.96E+02	2.96E+02
ENSG00000182504	CEP97	1.48	1.48	0.57	2.51E-05	2.96E+02	2.96E+02
ENSG00000243156	MICAL3	1.45	1.45	0.54	2.41E-05	2.96E+02	2.96E+02
ENSG00000228439	TSTD3	0.58	-1.71	-0.78	3.36E-05	-2.96E+02	2.96E+02
ENSG00000136888	ATP6V1G1	1.39	1.39	0.48	2.22E-05	2.95E+02	2.95E+02
ENSG00000138375	SMARCAL1	1.38	1.38	0.46	2.21E-05	2.94E+02	2.94E+02
ENSG00000164736	SOX17	1.39	1.39	0.48	2.25E-05	2.93E+02	2.93E+02
ENSG00000185219	ZNF445	0.69	-1.44	-0.53	2.43E-05	-2.93E+02	2.93E+02
ENSG00000120696	KBTBD7	1.43	1.43	0.51	2.38E-05	2.93E+02	2.93E+02
ENSG00000186281	GPAT2	0.60	-1.65	-0.73	3.19E-05	-2.93E+02	2.93E+02
ENSG00000130193	THEM6	0.72	-1.39	-0.48	2.28E-05	-2.92E+02	2.92E+02
ENSG00000075292	ZNF638	1.56	1.56	0.64	2.86E-05	2.92E+02	2.92E+02
ENSG00000145335	SNCA	0.70	-1.42	-0.51	2.38E-05	-2.91E+02	2.91E+02
ENSG00000101255	TRIB3	1.38	1.38	0.47	2.26E-05	2.91E+02	2.91E+02
ENSG00000034693	PEX3	0.71	-1.40	-0.49	2.33E-05	-2.90E+02	2.90E+02
ENSG00000171735	CAMTA1	0.70	-1.42	-0.51	2.43E-05	-2.89E+02	2.89E+02
ENSG00000169908	TM4SF1	1.40	1.40	0.48	2.36E-05	2.88E+02	2.88E+02
ENSG00000085117	CD82	1.65	1.65	0.72	3.28E-05	2.88E+02	2.88E+02
ENSG00000152104	PTPN14	1.39	1.39	0.48	2.34E-05	2.88E+02	2.88E+02
ENSG00000228742	RP5-884M6.1	1.68	1.68	0.75	3.43E-05	2.87E+02	2.87E+02
ENSG00000154217	PITPNC1	1.44	1.44	0.52	2.52E-05	2.86E+02	2.86E+02
ENSG00000178104	PDE4DIP	1.38	1.38	0.46	2.35E-05	2.85E+02	2.85E+02
ENSG00000130332	LSM7	0.68	-1.46	-0.55	2.64E-05	-2.84E+02	2.84E+02
ENSG00000120802	TMPO	0.71	-1.41	-0.49	2.46E-05	-2.84E+02	2.84E+02
ENSG00000149292	TTC12	1.50	1.50	0.58	2.82E-05	2.82E+02	2.82E+02
ENSG00000119979	FAM45A	1.43	1.43	0.51	2.57E-05	2.82E+02	2.82E+02
ENSG00000130520	LSM4	1.38	1.38	0.47	2.42E-05	2.81E+02	2.81E+02
ENSG00000102699	PARP4	1.39	1.39	0.47	2.44E-05	2.81E+02	2.81E+02
ENSG00000132535	DLG4	0.64	-1.57	-0.65	3.14E-05	-2.81E+02	2.81E+02
ENSG00000254004	ZNF260	1.56	1.56	0.64	3.08E-05	2.80E+02	2.80E+02
ENSG00000230734	RPL10P3	0.69	-1.44	-0.53	2.65E-05	-2.80E+02	2.80E+02
ENSG00000136960	ENPP2	0.53	-1.90	-0.92	4.61E-05	-2.80E+02	2.80E+02
ENSG00000005007	UPF1	1.47	1.47	0.55	2.77E-05	2.79E+02	2.79E+02
ENSG00000130810	PPAN	0.59	-1.69	-0.76	3.67E-05	-2.79E+02	2.79E+02
ENSG00000069998	CECR5	0.73	-1.37	-0.46	2.42E-05	-2.79E+02	2.79E+02
ENSG00000266903	CTB-171A8.1	1.53	1.53	0.62	3.03E-05	2.78E+02	2.78E+02
ENSG00000168615	ADAM9	1.43	1.43	0.52	2.66E-05	2.78E+02	2.78E+02
ENSG00000168872	DDX19A	0.73	-1.37	-0.45	2.44E-05	-2.78E+02	2.78E+02
ENSG00000160193	WDR4	0.59	-1.68	-0.75	3.70E-05	-2.76E+02	2.76E+02
ENSG00000106665	CLIP2	1.37	1.37	0.46	2.46E-05	2.76E+02	2.76E+02
ENSG00000107854	TNKS2	1.55	1.55	0.63	3.18E-05	2.74E+02	2.74E+02
ENSG00000136161	RCBTB2	0.71	-1.40	-0.48	2.64E-05	-2.72E+02	2.72E+02
ENSG00000197776	KLHDC1	1.86	1.86	0.89	4.68E-05	2.71E+02	2.71E+02
ENSG00000121039	RDH10	1.38	1.38	0.46	2.58E-05	2.71E+02	2.71E+02
ENSG00000133216	EPHB2	1.56	1.56	0.65	3.36E-05	2.70E+02	2.70E+02
ENSG00000116406	EDEM3	1.41	1.41	0.50	2.78E-05	2.67E+02	2.67E+02
ENSG00000168216	LMBRD1	1.38	1.38	0.46	2.66E-05	2.67E+02	2.67E+02
ENSG00000163884	KLF15	0.61	-1.63	-0.70	3.73E-05	-2.67E+02	2.67E+02
ENSG00000164687	FABP5	0.62	-1.62	-0.70	3.72E-05	-2.66E+02	2.66E+02
ENSG00000178449	COX14	0.68	-1.47	-0.55	3.04E-05	-2.66E+02	2.66E+02
ENSG00000125772	GPCPD1	0.72	-1.38	-0.47	2.72E-05	-2.65E+02	2.65E+02

ENSG00000066583	ISOC1	0.71	-1.40	-0.49	2.83E-05	-2.64E+02	2.64E+02
ENSG00000250241	RP11-9G1.3	1.79	1.79	0.84	4.59E-05	2.64E+02	2.64E+02
ENSG00000147889	CDKN2A	1.79	1.79	0.84	4.67E-05	2.62E+02	2.62E+02
ENSG00000005486	RHBDD2	1.44	1.44	0.52	3.01E-05	2.62E+02	2.62E+02
ENSG00000175471	MCTP1	0.69	-1.45	-0.53	3.06E-05	-2.62E+02	2.62E+02
ENSG00000102780	DGKH	1.44	1.44	0.53	3.05E-05	2.62E+02	2.62E+02
ENSG00000130826	DKC1	0.72	-1.39	-0.48	2.83E-05	-2.61E+02	2.61E+02
ENSG00000221994	ZNF630	1.61	1.61	0.69	3.85E-05	2.60E+02	2.60E+02
ENSG00000119242	CCDC92	1.43	1.43	0.52	3.04E-05	2.60E+02	2.60E+02
ENSG00000095321	CRAT	1.47	1.47	0.56	3.21E-05	2.59E+02	2.59E+02
ENSG00000108175	ZMIZ1	1.43	1.43	0.52	3.06E-05	2.59E+02	2.59E+02
ENSG00000125450	NUP85	0.73	-1.38	-0.46	2.82E-05	-2.59E+02	2.59E+02
ENSG00000136878	USP20	1.41	1.41	0.49	2.97E-05	2.59E+02	2.59E+02
ENSG00000119699	TGFB3	1.56	1.56	0.64	3.63E-05	2.59E+02	2.59E+02
ENSG00000198089	SFI1	0.69	-1.44	-0.53	3.12E-05	-2.58E+02	2.58E+02
ENSG00000198742	SMURF1	1.39	1.39	0.47	2.92E-05	2.57E+02	2.57E+02
ENSG00000140526	ABHD2	1.44	1.44	0.52	3.13E-05	2.57E+02	2.57E+02
ENSG00000139629	GALNT6	1.46	1.46	0.55	3.26E-05	2.56E+02	2.56E+02
ENSG00000116815	CD58	0.69	-1.44	-0.53	3.17E-05	-2.56E+02	2.56E+02
ENSG00000184489	PTP4A3	1.62	1.62	0.70	4.03E-05	2.55E+02	2.55E+02
ENSG00000106780	MEGF9	1.38	1.38	0.47	2.94E-05	2.55E+02	2.55E+02
ENSG00000171159	C9orf16	0.72	-1.40	-0.48	3.01E-05	-2.55E+02	2.55E+02
ENSG00000112893	MAN2A1	1.43	1.43	0.51	3.15E-05	2.55E+02	2.55E+02
ENSG00000172432	GTPBP2	0.73	-1.38	-0.46	2.94E-05	-2.54E+02	2.54E+02
ENSG00000125149	C16orf70	0.72	-1.39	-0.47	2.99E-05	-2.54E+02	2.54E+02
ENSG00000166333	ILK	1.40	1.40	0.48	3.05E-05	2.53E+02	2.53E+02
ENSG00000177469	PTRF	1.39	1.39	0.48	3.04E-05	2.53E+02	2.53E+02
ENSG00000225138	CTD-2228K2.7	1.48	1.48	0.56	3.41E-05	2.53E+02	2.53E+02
ENSG00000105971	CAV2	1.40	1.40	0.48	3.08E-05	2.52E+02	2.52E+02
ENSG00000172469	MANEA	0.67	-1.49	-0.58	3.49E-05	-2.52E+02	2.52E+02
ENSG00000113318	MSH3	1.36	1.36	0.44	2.95E-05	2.51E+02	2.51E+02
ENSG00000124201	ZNFX1	1.38	1.38	0.47	3.04E-05	2.51E+02	2.51E+02
ENSG00000196924	FLNA	1.55	1.55	0.64	3.85E-05	2.50E+02	2.50E+02
ENSG00000105612	DNASE2	1.38	1.38	0.47	3.04E-05	2.50E+02	2.50E+02
ENSG00000171310	CHST11	1.37	1.37	0.46	3.01E-05	2.50E+02	2.50E+02
ENSG00000166046	TCP11L2	1.41	1.41	0.49	3.17E-05	2.50E+02	2.50E+02
ENSG00000171811	TTC40	0.54	-1.85	-0.89	5.53E-05	-2.49E+02	2.49E+02
ENSG00000198856	OSTC	0.69	-1.45	-0.54	3.39E-05	-2.49E+02	2.49E+02
ENSG00000104936	DMPK	1.51	1.51	0.59	3.73E-05	2.47E+02	2.47E+02
ENSG00000141480	ARRB2	0.71	-1.40	-0.49	3.26E-05	-2.46E+02	2.46E+02
ENSG00000113845	TIMMDC1	1.38	1.38	0.46	3.16E-05	2.45E+02	2.45E+02
ENSG00000044115	CTNNA1	1.40	1.40	0.49	3.27E-05	2.45E+02	2.45E+02
ENSG00000107371	EXOSC3	0.73	-1.37	-0.45	3.14E-05	-2.44E+02	2.44E+02
ENSG00000105738	SIPA1L3	1.48	1.48	0.57	3.67E-05	2.44E+02	2.44E+02
ENSG00000261770	CTC-459F4.1	1.79	1.79	0.84	5.40E-05	2.43E+02	2.43E+02
ENSG00000134030	CTIF	1.37	1.37	0.46	3.19E-05	2.43E+02	2.43E+02
ENSG00000166188	ZNF319	1.45	1.45	0.54	3.60E-05	2.42E+02	2.42E+02
ENSG00000140545	MFGE8	1.52	1.52	0.60	3.91E-05	2.42E+02	2.42E+02
ENSG00000204564	C6orf136	0.62	-1.60	-0.68	4.39E-05	-2.42E+02	2.42E+02
ENSG00000142327	RNPEPL1	1.36	1.36	0.44	3.17E-05	2.42E+02	2.42E+02
	RP11-325F22.4	1.81	1.81	0.86	5.63E-05	2.42E+02	2.42E+02
ENSG00000125459	MSTO1	0.68	-1.48	-0.56	3.77E-05	-2.40E+02	2.40E+02
ENSG00000110092	CCND1	1.46	1.46	0.55	3.69E-05	2.40E+02	2.40E+02
ENSG00000092621	PHGDH	0.70	-1.43	-0.51	3.53E-05	-2.40E+02	2.40E+02
ENSG00000184110	EIF3C	0.61	-1.63	-0.71	4.64E-05	-2.39E+02	2.39E+02
ENSG00000146950	SHROOM2	1.54	1.54	0.63	4.16E-05	2.39E+02	2.39E+02
ENSG00000147416	ATP6V1B2	1.38	1.38	0.47	3.35E-05	2.39E+02	2.39E+02
ENSG00000100209	HSCB	1.41	1.41	0.49	3.46E-05	2.39E+02	2.39E+02
ENSG00000164659	KIAA1324L	1.36	1.36	0.45	3.28E-05	2.38E+02	2.38E+02
ENSG00000104946	TBC1D17	1.44	1.44	0.52	3.66E-05	2.38E+02	2.38E+02
ENSG00000168273	SMIM4	0.59	-1.69	-0.75	5.06E-05	-2.37E+02	2.37E+02
ENSG00000144034	TPRKB	0.72	-1.39	-0.47	3.47E-05	-2.35E+02	2.35E+02
ENSG00000152127	MGAT5	1.39	1.39	0.47	3.52E-05	2.34E+02	2.34E+02
ENSG00000188554	NBR1	1.39	1.39	0.47	3.51E-05	2.34E+02	2.34E+02
ENSG00000140534	TICRR	0.69	-1.45	-0.53	3.85E-05	-2.33E+02	2.33E+02
ENSG00000004975	DVL2	0.71	-1.40	-0.49	3.61E-05	-2.33E+02	2.33E+02
ENSG00000140451	PIF1	0.68	-1.46	-0.55	3.94E-05	-2.33E+02	2.33E+02
ENSG00000101871	MID1	1.39	1.39	0.47	3.54E-05	2.33E+02	2.33E+02
ENSG00000151876	FBXO4	1.43	1.43	0.51	3.78E-05	2.32E+02	2.32E+02
ENSG00000236901	MIR600HG	0.57	-1.74	-0.80	5.65E-05	-2.31E+02	2.31E+02
ENSG00000063176	SPHK2	1.49	1.49	0.58	4.15E-05	2.31E+02	2.31E+02
ENSG00000143147	GPR161	1.36	1.36	0.44	3.47E-05	2.31E+02	2.31E+02
ENSG00000267473	AC005789.11	1.67	1.67	0.74	5.23E-05	2.31E+02	2.31E+02
ENSG00000177082	WDR73	0.73	-1.36	-0.45	3.50E-05	-2.31E+02	2.31E+02
ENSG00000269937	RP11-20I23.8	1.58	1.58	0.66	4.71E-05	2.31E+02	2.31E+02
ENSG00000152749	GPR180	1.41	1.41	0.49	3.72E-05	2.31E+02	2.31E+02
ENSG00000155755	TMEM237	0.73	-1.36	-0.45	3.50E-05	-2.30E+02	2.30E+02
ENSG00000105397	TYK2	0.73	-1.36	-0.45	3.52E-05	-2.30E+02	2.30E+02
ENSG00000145365	TIFA	0.73	-1.37	-0.45	3.57E-05	-2.29E+02	2.29E+02

ENSG00000204540	PSORS1C1	1.58	1.58	0.66	4.80E-05	2.28E+02	2.28E+02
ENSG00000201098	RNY1	1.88	1.88	0.91	6.76E-05	2.28E+02	2.28E+02
	RP11-31E23.1	1.72	1.72	0.78	5.74E-05	2.27E+02	2.27E+02
ENSG00000165512	ZNF22	0.71	-1.40	-0.49	3.86E-05	-2.26E+02	2.26E+02
ENSG00000198081	ZFP161	1.36	1.36	0.45	3.64E-05	2.26E+02	2.26E+02
ENSG00000130382	MLLT1	0.70	-1.43	-0.52	4.01E-05	-2.26E+02	2.26E+02
ENSG00000167785	ZNF558	1.47	1.47	0.56	4.26E-05	2.25E+02	2.25E+02
ENSG00000198885	ITPRIPL1	0.60	-1.66	-0.73	5.46E-05	-2.25E+02	2.25E+02
ENSG00000167323	STIM1	1.47	1.47	0.55	4.26E-05	2.25E+02	2.25E+02
ENSG00000179832	HEATR7A	1.45	1.45	0.53	4.16E-05	2.24E+02	2.24E+02
ENSG00000173064	HECTD4	1.47	1.47	0.56	4.32E-05	2.23E+02	2.23E+02
ENSG00000107874	CUEDC2	1.36	1.36	0.44	3.72E-05	2.23E+02	2.23E+02
ENSG00000142534	RPS11	0.71	-1.41	-0.49	4.00E-05	-2.22E+02	2.22E+02
ENSG00000160087	UBE2J2	1.36	1.36	0.44	3.77E-05	2.21E+02	2.21E+02
ENSG00000089248	ERP29	0.71	-1.42	-0.50	4.14E-05	-2.21E+02	2.21E+02
ENSG00000229043	AC091729.9	1.69	1.69	0.76	5.90E-05	2.21E+02	2.21E+02
ENSG00000130023	C6orf70	1.37	1.37	0.46	3.94E-05	2.19E+02	2.19E+02
ENSG00000261526	CTB-31O20.2	0.57	-1.75	-0.81	6.42E-05	-2.18E+02	2.18E+02
ENSG00000105518	TMEM205	1.38	1.38	0.46	3.99E-05	2.18E+02	2.18E+02
ENSG00000136021	SCYL2	1.51	1.51	0.60	4.87E-05	2.17E+02	2.17E+02
ENSG00000115128	SF3B14	0.72	-1.39	-0.48	4.15E-05	-2.16E+02	2.16E+02
ENSG00000162999	DUSP19	1.52	1.52	0.60	4.93E-05	2.16E+02	2.16E+02
ENSG00000065615	CYB5R4	1.47	1.47	0.55	4.63E-05	2.15E+02	2.15E+02
ENSG00000087111	PIGS	0.71	-1.42	-0.50	4.36E-05	-2.15E+02	2.15E+02
ENSG00000115758	ODC1	0.72	-1.40	-0.48	4.27E-05	-2.14E+02	2.14E+02
ENSG00000104447	TRPS1	1.46	1.46	0.54	4.65E-05	2.14E+02	2.14E+02
ENSG00000119689	DLST	0.69	-1.45	-0.53	4.60E-05	-2.13E+02	2.13E+02
ENSG00000123700	KCNJ2	0.72	-1.38	-0.47	4.20E-05	-2.13E+02	2.13E+02
ENSG00000132434	LANCL2	0.74	-1.36	-0.44	4.05E-05	-2.13E+02	2.13E+02
ENSG00000227825	SLC9A7P1	0.61	-1.64	-0.72	5.97E-05	-2.13E+02	2.13E+02
ENSG00000274151	AC018737.1	0.63	-1.58	-0.66	5.55E-05	-2.13E+02	2.13E+02
ENSG00000141741	MIEN1	0.73	-1.36	-0.45	4.12E-05	-2.12E+02	2.12E+02
ENSG00000158528	PPP1R9A	0.64	-1.56	-0.64	5.38E-05	-2.12E+02	2.12E+02
ENSG00000178028	DMAP1	0.69	-1.45	-0.54	4.71E-05	-2.11E+02	2.11E+02
ENSG00000172831	CES2	1.37	1.37	0.45	4.24E-05	2.10E+02	2.10E+02
ENSG00000177311	ZBTB38	1.46	1.46	0.54	4.86E-05	2.09E+02	2.09E+02
ENSG00000143751	SDE2	1.38	1.38	0.46	4.34E-05	2.09E+02	2.09E+02
ENSG00000149483	TMEM138	0.71	-1.41	-0.50	4.56E-05	-2.09E+02	2.09E+02
ENSG00000138119	MYOF	1.38	1.38	0.47	4.39E-05	2.09E+02	2.09E+02
ENSG00000169744	LDB2	0.73	-1.38	-0.46	4.35E-05	-2.09E+02	2.09E+02
ENSG00000108094	CUL2	1.42	1.42	0.50	4.61E-05	2.09E+02	2.09E+02
ENSG00000198453	ZNF568	1.41	1.41	0.50	4.59E-05	2.09E+02	2.09E+02
ENSG00000168434	COG7	0.72	-1.39	-0.47	4.42E-05	-2.08E+02	2.08E+02
ENSG00000138642	HERC6	0.67	-1.50	-0.59	5.23E-05	-2.08E+02	2.08E+02
ENSG00000167513	CDT1	0.70	-1.42	-0.50	4.68E-05	-2.07E+02	2.07E+02
ENSG00000116157	GPX7	0.71	-1.40	-0.49	4.56E-05	-2.07E+02	2.07E+02
ENSG00000174282	ZBTB4	1.36	1.36	0.45	4.32E-05	2.07E+02	2.07E+02
ENSG00000100815	TRIP11	1.60	1.60	0.68	6.01E-05	2.07E+02	2.07E+02
ENSG00000174796	THAP6	1.45	1.45	0.53	4.91E-05	2.07E+02	2.07E+02
ENSG00000167291	TBC1D16	0.73	-1.37	-0.46	4.41E-05	-2.06E+02	2.06E+02
ENSG00000161016	RPL8	0.71	-1.42	-0.50	4.75E-05	-2.06E+02	2.06E+02
ENSG00000185551	NR2F2	0.71	-1.41	-0.49	4.70E-05	-2.05E+02	2.05E+02
ENSG00000164543	STK17A	0.72	-1.38	-0.47	4.53E-05	-2.05E+02	2.05E+02
ENSG00000116711	PLA2G4A	1.41	1.41	0.50	4.76E-05	2.05E+02	2.05E+02
ENSG00000232388	LINC00493	0.72	-1.39	-0.47	4.60E-05	-2.04E+02	2.04E+02
ENSG00000144283	PKP4	0.72	-1.39	-0.47	4.62E-05	-2.04E+02	2.04E+02
ENSG00000247626	MARS2	0.69	-1.45	-0.54	5.09E-05	-2.04E+02	2.04E+02
ENSG00000151967	SCHIP1	1.46	1.46	0.55	5.18E-05	2.04E+02	2.04E+02
ENSG00000183077	AFMID	0.71	-1.40	-0.49	4.77E-05	-2.03E+02	2.03E+02
ENSG00000154263	ABCA10	1.69	1.69	0.76	6.99E-05	2.02E+02	2.02E+02
ENSG00000204271	SPIN3	0.55	-1.83	-0.88	8.27E-05	-2.02E+02	2.02E+02
ENSG00000160221	C21orf33	1.35	1.35	0.43	4.49E-05	2.01E+02	2.01E+02
ENSG00000198093	ZNF649	0.70	-1.43	-0.51	5.03E-05	-2.01E+02	2.01E+02
ENSG00000144426	NBEAL1	1.73	1.73	0.79	7.45E-05	2.01E+02	2.01E+02
ENSG00000115282	TTC31	0.70	-1.44	-0.52	5.14E-05	-2.01E+02	2.01E+02
ENSG00000075213	SEMA3A	0.56	-1.80	-0.85	8.05E-05	-2.00E+02	2.00E+02
ENSG00000214706	IFRD2	0.71	-1.42	-0.50	5.07E-05	-1.99E+02	1.99E+02
ENSG00000186193	SAPCD2	0.70	-1.43	-0.51	5.16E-05	-1.99E+02	1.99E+02
ENSG00000143947	RPS27A	0.68	-1.46	-0.55	5.42E-05	-1.98E+02	1.98E+02
ENSG00000116871	MAP7D1	1.42	1.42	0.51	5.16E-05	1.98E+02	1.98E+02
ENSG00000114353	GNAI2	1.38	1.38	0.47	4.88E-05	1.98E+02	1.98E+02
ENSG00000167005	NUDT21	0.72	-1.38	-0.47	4.94E-05	-1.97E+02	1.97E+02
ENSG00000154743	TSEN2	0.69	-1.44	-0.53	5.39E-05	-1.96E+02	1.96E+02
ENSG00000076944	STXBP2	1.43	1.43	0.52	5.36E-05	1.96E+02	1.96E+02
ENSG00000148335	NTMT1	0.73	-1.38	-0.46	4.97E-05	-1.95E+02	1.95E+02
ENSG00000124145	SDC4	1.38	1.38	0.46	5.00E-05	1.94E+02	1.94E+02
ENSG00000180011	ZADH2	1.36	1.36	0.45	4.92E-05	1.94E+02	1.94E+02
ENSG00000077348	EXOSC5	0.68	-1.46	-0.55	5.72E-05	-1.94E+02	1.94E+02
ENSG00000105948	TTC26	0.73	-1.37	-0.46	5.06E-05	-1.93E+02	1.93E+02

ENSG00000139826	ABHD13	1.38	1.38	0.46	5.11E-05	1.93E+02	1.93E+02
ENSG00000152056	AP1S3	0.63	-1.60	-0.68	6.89E-05	-1.93E+02	1.93E+02
ENSG00000104953	TLE6	1.92	1.92	0.94	1.01E-04	1.91E+02	1.91E+02
ENSG00000143164	DCAF6	1.37	1.37	0.45	5.12E-05	1.91E+02	1.91E+02
ENSG00000168575	SLC20A2	1.43	1.43	0.52	5.63E-05	1.91E+02	1.91E+02
ENSG00000127955	GNAI1	0.74	-1.35	-0.43	5.01E-05	-1.91E+02	1.91E+02
ENSG00000104915	STX10	0.74	-1.36	-0.44	5.07E-05	-1.90E+02	1.90E+02
ENSG00000122778	KIAA1549	1.46	1.46	0.55	5.96E-05	1.90E+02	1.90E+02
ENSG00000182768	NGRN	1.36	1.36	0.44	5.19E-05	1.89E+02	1.89E+02
ENSG00000269900	RMRP	0.67	-1.49	-0.57	6.21E-05	-1.89E+02	1.89E+02
ENSG00000166685	COG1	0.74	-1.36	-0.44	5.23E-05	-1.87E+02	1.87E+02
ENSG00000155393	HEATR3	0.74	-1.35	-0.43	5.17E-05	-1.87E+02	1.87E+02
ENSG00000203875	SNHG5	0.70	-1.43	-0.52	5.89E-05	-1.86E+02	1.86E+02
ENSG00000134285	FKBP11	0.71	-1.41	-0.50	5.75E-05	-1.86E+02	1.86E+02
	RP11-323F24.1	1.65	1.65	0.72	7.99E-05	1.84E+02	1.84E+02
ENSG00000176018	LYSMD3	1.43	1.43	0.52	6.02E-05	1.84E+02	1.84E+02
ENSG00000159082	SYNJ1	1.49	1.49	0.57	6.52E-05	1.84E+02	1.84E+02
ENSG00000164081	TEX264	1.35	1.35	0.44	5.40E-05	1.84E+02	1.84E+02
ENSG00000129474	AJUBA	1.34	1.34	0.42	5.31E-05	1.84E+02	1.84E+02
ENSG00000255389	TRAF3IP2-AS1	0.61	-1.64	-0.72	8.03E-05	-1.83E+02	1.83E+02
ENSG00000167306	MYO5B	1.79	1.79	0.84	9.50E-05	1.83E+02	1.83E+02
ENSG00000186185	KIF18B	0.73	-1.38	-0.46	5.70E-05	-1.82E+02	1.82E+02
ENSG00000082701	GSK3B	1.39	1.39	0.47	5.79E-05	1.82E+02	1.82E+02
ENSG00000136247	ZDHHC4	0.74	-1.36	-0.44	5.58E-05	-1.82E+02	1.82E+02
ENSG00000197702	PARVA	0.72	-1.38	-0.47	5.78E-05	-1.82E+02	1.82E+02
ENSG00000005022	SLC25A5	0.73	-1.38	-0.46	5.78E-05	-1.81E+02	1.81E+02
ENSG00000138035	PNPT1	0.73	-1.37	-0.45	5.67E-05	-1.81E+02	1.81E+02
	CTD-2314B22.3	1.47	1.47	0.55	6.56E-05	1.81E+02	1.81E+02
ENSG00000196954	CASP4	0.73	-1.37	-0.46	5.76E-05	-1.81E+02	1.81E+02
ENSG00000127419	TMEM175	0.58	-1.71	-0.78	9.00E-05	-1.80E+02	1.80E+02
ENSG00000074590	NUAK1	1.39	1.39	0.47	5.91E-05	1.80E+02	1.80E+02
ENSG00000005100	DHX33	0.72	-1.40	-0.48	6.02E-05	-1.80E+02	1.80E+02
ENSG00000188290	HES4	1.72	1.72	0.78	9.15E-05	1.79E+02	1.79E+02
ENSG00000164253	WDR41	1.36	1.36	0.45	5.76E-05	1.79E+02	1.79E+02
ENSG00000135245	HILPDA	0.70	-1.42	-0.51	6.35E-05	-1.79E+02	1.79E+02
ENSG00000114019	AMOTL2	1.41	1.41	0.49	6.26E-05	1.78E+02	1.78E+02
ENSG00000137449	CPEB2	1.40	1.40	0.49	6.21E-05	1.78E+02	1.78E+02
ENSG00000125266	EFNB2	0.74	-1.36	-0.44	5.81E-05	-1.78E+02	1.78E+02
ENSG00000185201	IFITM2	0.73	-1.37	-0.46	6.03E-05	-1.77E+02	1.77E+02
ENSG00000104450	SPAG1	0.70	-1.43	-0.52	6.66E-05	-1.75E+02	1.75E+02
ENSG00000111726	CMAS	0.73	-1.37	-0.46	6.17E-05	-1.75E+02	1.75E+02
ENSG00000271601	LIX1L	0.74	-1.36	-0.44	6.01E-05	-1.75E+02	1.75E+02
ENSG00000174996	KLC2	1.37	1.37	0.45	6.12E-05	1.75E+02	1.75E+02
ENSG00000100461	RBM23	0.74	-1.35	-0.44	6.03E-05	-1.74E+02	1.74E+02
ENSG00000183474	GTF2H2C	0.69	-1.45	-0.54	7.01E-05	-1.73E+02	1.73E+02
ENSG00000167565	SERTAD3	1.35	1.35	0.43	6.09E-05	1.73E+02	1.73E+02
ENSG00000163605	PPP4R2	1.40	1.40	0.49	6.62E-05	1.72E+02	1.72E+02
ENSG00000158769	F11R	1.37	1.37	0.45	6.34E-05	1.72E+02	1.72E+02
ENSG00000114115	RBP1	0.60	-1.67	-0.74	9.48E-05	-1.72E+02	1.72E+02
ENSG00000189362	TMEM194B	0.68	-1.46	-0.55	7.26E-05	-1.71E+02	1.71E+02
ENSG00000163428	LRRC58	1.36	1.36	0.44	6.27E-05	1.71E+02	1.71E+02
ENSG00000187778	MCRS1	0.74	-1.36	-0.44	6.38E-05	-1.70E+02	1.70E+02
ENSG00000188811	NHLRC3	1.36	1.36	0.44	6.38E-05	1.70E+02	1.70E+02
ENSG00000157827	FMNL2	1.36	1.36	0.44	6.38E-05	1.70E+02	1.70E+02
ENSG00000173889	PHC3	1.39	1.39	0.48	6.71E-05	1.70E+02	1.70E+02
ENSG00000137878	GCOM1	1.65	1.65	0.72	9.38E-05	1.70E+02	1.70E+02
ENSG00000198692	EIF1AY	0.56	-1.79	-0.84	1.12E-04	-1.69E+02	1.69E+02
ENSG00000177981	ASB8	0.74	-1.34	-0.43	6.30E-05	-1.69E+02	1.69E+02
ENSG00000222041	LINC00152	1.34	1.34	0.43	6.38E-05	1.68E+02	1.68E+02
ENSG00000149639	SOGA1	1.38	1.38	0.47	6.75E-05	1.68E+02	1.68E+02
ENSG00000171033	PKIA	1.36	1.36	0.45	6.60E-05	1.68E+02	1.68E+02
ENSG00000082996	RNF13	1.36	1.36	0.44	6.57E-05	1.68E+02	1.68E+02
ENSG00000256061	DYX1C1	1.73	1.73	0.79	1.07E-04	1.67E+02	1.67E+02
ENSG00000188610	FAM72B	1.66	1.66	0.73	9.84E-05	1.67E+02	1.67E+02
ENSG00000130340	SNX9	1.36	1.36	0.45	6.66E-05	1.67E+02	1.67E+02
ENSG00000006327	TNFRSF12A	0.73	-1.37	-0.46	6.77E-05	-1.67E+02	1.67E+02
ENSG00000133858	ZFC3H1	1.47	1.47	0.55	7.74E-05	1.67E+02	1.67E+02
ENSG00000113441	LNPEP	1.37	1.37	0.45	6.73E-05	1.67E+02	1.67E+02
ENSG00000091140	DLD	0.73	-1.37	-0.45	6.80E-05	-1.66E+02	1.66E+02
ENSG00000128965	CHAC1	1.76	1.76	0.82	1.13E-04	1.66E+02	1.66E+02
ENSG00000113593	PPWD1	0.72	-1.38	-0.47	7.02E-05	-1.65E+02	1.65E+02
ENSG00000070669	ASNS	0.71	-1.40	-0.49	7.24E-05	-1.65E+02	1.65E+02
ENSG00000152455	SUV39H2	0.74	-1.36	-0.44	6.79E-05	-1.65E+02	1.65E+02
ENSG00000146278	PNRC1	1.38	1.38	0.47	7.06E-05	1.64E+02	1.64E+02
ENSG00000112378	PERP	1.37	1.37	0.45	6.94E-05	1.64E+02	1.64E+02
ENSG00000188677	PARVB	1.38	1.38	0.46	7.03E-05	1.64E+02	1.64E+02
ENSG00000229809	ZNF688	1.50	1.50	0.59	8.37E-05	1.64E+02	1.64E+02
ENSG00000163513	TGFBR2	1.38	1.38	0.46	7.02E-05	1.64E+02	1.64E+02
ENSG00000170917	NUDT6	1.41	1.41	0.49	7.37E-05	1.64E+02	1.64E+02

ENSG00000152332	UHMK1	1.39	1.39	0.47	7.22E-05	1.63E+02	1.63E+02
ENSG00000123384	LRP1	1.58	1.58	0.66	9.43E-05	1.63E+02	1.63E+02
ENSG00000118640	VAMP8	0.72	-1.39	-0.48	7.36E-05	-1.63E+02	1.63E+02
ENSG00000108883	EFTUD2	0.73	-1.37	-0.46	7.14E-05	-1.62E+02	1.62E+02
ENSG00000116649	SRM	0.74	-1.34	-0.43	7.00E-05	-1.61E+02	1.61E+02
ENSG00000105538	RASIP1	1.42	1.42	0.51	7.86E-05	1.60E+02	1.60E+02
ENSG00000204394	VARS	1.40	1.40	0.49	7.71E-05	1.60E+02	1.60E+02
ENSG00000178605	GTPBP6	0.73	-1.37	-0.45	7.36E-05	-1.59E+02	1.59E+02
ENSG00000131435	PDLIM4	1.36	1.36	0.44	7.29E-05	1.59E+02	1.59E+02
ENSG00000164654	MIOS	0.75	-1.34	-0.42	7.20E-05	-1.58E+02	1.58E+02
ENSG00000109787	KLF3	1.37	1.37	0.45	7.47E-05	1.58E+02	1.58E+02
ENSG00000132950	ZMYM5	1.35	1.35	0.43	7.33E-05	1.58E+02	1.58E+02
ENSG00000006530	AGK	0.75	-1.34	-0.42	7.32E-05	-1.56E+02	1.56E+02
ENSG00000181894	ZNF329	1.36	1.36	0.44	7.56E-05	1.56E+02	1.56E+02
ENSG00000178971	CTC1	0.65	-1.53	-0.61	9.64E-05	-1.56E+02	1.56E+02
ENSG00000077514	POLD3	0.75	-1.34	-0.42	7.52E-05	-1.55E+02	1.55E+02
ENSG00000089820	ARHGAP4	1.35	1.35	0.43	7.61E-05	1.54E+02	1.54E+02
ENSG00000141101	NOB1	0.68	-1.47	-0.56	9.10E-05	-1.54E+02	1.54E+02
ENSG00000005189	AC004381.6	0.68	-1.48	-0.56	9.16E-05	-1.54E+02	1.54E+02
ENSG00000185896	LAMP1	1.39	1.39	0.48	8.17E-05	1.54E+02	1.54E+02
ENSG00000145979	TBC1D7	1.36	1.36	0.44	7.84E-05	1.53E+02	1.53E+02
ENSG00000244627	RP3-449O17.1	1.74	1.74	0.80	1.30E-04	1.53E+02	1.53E+02
ENSG00000167074	TEF	1.52	1.52	0.61	9.93E-05	1.53E+02	1.53E+02
ENSG00000165501	LRR1	0.74	-1.35	-0.43	7.80E-05	-1.53E+02	1.53E+02
ENSG00000111371	SLC38A1	1.36	1.36	0.45	7.95E-05	1.53E+02	1.53E+02
ENSG00000087448	KLHDC5	1.36	1.36	0.44	7.97E-05	1.52E+02	1.52E+02
ENSG00000001167	NFYA	1.53	1.53	0.61	1.01E-04	1.52E+02	1.52E+02
ENSG00000005339	CREBBP	1.40	1.40	0.49	8.47E-05	1.52E+02	1.52E+02
ENSG00000168234	TTC39C	0.67	-1.49	-0.58	9.64E-05	-1.52E+02	1.52E+02
ENSG00000129071	MBD4	0.75	-1.34	-0.42	7.78E-05	-1.52E+02	1.52E+02
ENSG00000126226	PCID2	0.74	-1.34	-0.43	7.92E-05	-1.51E+02	1.51E+02
ENSG00000158427	TMSB15B	1.37	1.37	0.45	8.21E-05	1.51E+02	1.51E+02
ENSG00000149177	PTPRJ	1.35	1.35	0.43	8.04E-05	1.50E+02	1.50E+02
ENSG00000175643	RMI2	0.68	-1.46	-0.55	9.53E-05	-1.50E+02	1.50E+02
ENSG00000089159	PXN	1.47	1.47	0.56	9.66E-05	1.50E+02	1.50E+02
ENSG00000253059	SNORA31	0.53	-1.87	-0.90	1.56E-04	-1.50E+02	1.50E+02
ENSG00000132823	C20orf111	1.34	1.34	0.42	8.01E-05	1.49E+02	1.49E+02
ENSG00000109220	CHIC2	1.33	1.33	0.42	8.09E-05	1.48E+02	1.48E+02
ENSG00000154957	ZNF18	1.42	1.42	0.51	9.21E-05	1.48E+02	1.48E+02
ENSG00000100142	POLR2F	0.73	-1.36	-0.45	8.49E-05	-1.48E+02	1.48E+02
ENSG00000182628	SKA2	0.73	-1.36	-0.45	8.55E-05	-1.47E+02	1.47E+02
ENSG00000181754	AMIGO1	0.58	-1.73	-0.79	1.37E-04	-1.47E+02	1.47E+02
ENSG00000100478	AP4S1	0.68	-1.48	-0.57	1.01E-04	-1.47E+02	1.47E+02
ENSG00000106772	PRUNE2	0.72	-1.39	-0.48	8.97E-05	-1.47E+02	1.47E+02
ENSG00000123473	STIL	0.75	-1.34	-0.42	8.34E-05	-1.47E+02	1.47E+02
ENSG00000130413	STK33	0.54	-1.84	-0.88	1.60E-04	-1.46E+02	1.46E+02
ENSG00000172465	TCEAL1	0.73	-1.38	-0.46	8.92E-05	-1.46E+02	1.46E+02
ENSG00000267532	MIR497HG	1.53	1.53	0.61	1.09E-04	1.46E+02	1.46E+02
ENSG00000235194	PPP1R3E	0.64	-1.57	-0.65	1.15E-04	-1.46E+02	1.46E+02
ENSG00000177842	ZNF620	0.68	-1.48	-0.56	1.03E-04	-1.46E+02	1.46E+02
ENSG00000121236	TRIM6	0.62	-1.60	-0.68	1.22E-04	-1.45E+02	1.45E+02
ENSG00000090889	KIF4A	0.73	-1.37	-0.46	8.95E-05	-1.45E+02	1.45E+02
ENSG00000196263	ZNF471	1.67	1.67	0.74	1.34E-04	1.44E+02	1.44E+02
ENSG00000106991	ENG	1.43	1.43	0.52	9.86E-05	1.44E+02	1.44E+02
ENSG00000147535	PPAPDC1B	0.74	-1.34	-0.43	8.69E-05	-1.44E+02	1.44E+02
ENSG00000101911	PRPS2	0.75	-1.33	-0.42	8.57E-05	-1.44E+02	1.44E+02
ENSG00000248487	ABHD14A	0.62	-1.61	-0.69	1.25E-04	-1.44E+02	1.44E+02
ENSG00000177946	CENPBD1	1.49	1.49	0.58	1.08E-04	1.44E+02	1.44E+02
ENSG00000157741	UBN2	1.37	1.37	0.45	9.02E-05	1.44E+02	1.44E+02
ENSG00000107816	LZTS2	1.34	1.34	0.42	8.72E-05	1.44E+02	1.44E+02
ENSG00000104679	R3HCC1	0.74	-1.34	-0.42	8.76E-05	-1.43E+02	1.43E+02
ENSG00000176715	ACSF3	0.75	-1.34	-0.42	8.84E-05	-1.43E+02	1.43E+02
ENSG00000105639	JAK3	0.54	-1.85	-0.88	1.67E-04	-1.43E+02	1.43E+02
ENSG00000096080	MRPS18A	0.74	-1.35	-0.43	8.96E-05	-1.42E+02	1.42E+02
ENSG00000167508	MVD	0.75	-1.34	-0.42	8.96E-05	-1.42E+02	1.42E+02
ENSG00000233429	HOTAIRM1	0.72	-1.38	-0.47	9.55E-05	-1.41E+02	1.41E+02
	RP11-446H18.3	0.70	-1.43	-0.52	1.03E-04	-1.41E+02	1.41E+02
ENSG00000213859	KCTD11	1.50	1.50	0.58	1.12E-04	1.41E+02	1.41E+02
ENSG00000109576	AADAT	0.55	-1.81	-0.86	1.67E-04	-1.40E+02	1.40E+02
ENSG00000182405	PGBD4	0.59	-1.70	-0.77	1.47E-04	-1.40E+02	1.40E+02
ENSG00000157216	SSBP3	1.34	1.34	0.42	9.23E-05	1.40E+02	1.40E+02
ENSG00000070476	ZXDC	1.35	1.35	0.44	9.47E-05	1.39E+02	1.39E+02
ENSG00000254122	PCDHGB7	1.75	1.75	0.81	1.58E-04	1.39E+02	1.39E+02
ENSG00000176390	CRLF3	0.75	-1.34	-0.42	9.29E-05	-1.39E+02	1.39E+02
ENSG00000215183	MSMP	1.35	1.35	0.44	9.50E-05	1.39E+02	1.39E+02
ENSG00000168517	HEXIM2	1.59	1.59	0.67	1.33E-04	1.38E+02	1.38E+02
ENSG00000130024	PHF10	1.35	1.35	0.43	9.60E-05	1.37E+02	1.37E+02
ENSG00000163528	CHCHD4	0.74	-1.36	-0.44	9.74E-05	-1.37E+02	1.37E+02
ENSG00000131446	MGAT1	0.73	-1.36	-0.45	9.89E-05	-1.37E+02	1.37E+02

ENSG00000123124	WWP1	1.41	1.41	0.49	1.06E-04	1.37E+02	1.37E+02
ENSG00000160570	DEDD2	1.37	1.37	0.45	1.00E-04	1.37E+02	1.37E+02
ENSG00000066056	TIE1	1.37	1.37	0.45	1.00E-04	1.37E+02	1.37E+02
ENSG00000168228	ZCCHC4	0.71	-1.40	-0.49	1.06E-04	-1.36E+02	1.36E+02
ENSG00000163002	NUP35	0.69	-1.44	-0.53	1.12E-04	-1.36E+02	1.36E+02
ENSG00000151117	TMEM86A	1.58	1.58	0.66	1.34E-04	1.36E+02	1.36E+02
ENSG00000161888	SPC24	0.73	-1.36	-0.45	1.00E-04	-1.36E+02	1.36E+02
ENSG00000156414	TDRD9	0.60	-1.68	-0.75	1.52E-04	-1.36E+02	1.36E+02
ENSG00000160172	FAM86C2P	1.65	1.65	0.72	1.48E-04	1.36E+02	1.36E+02
ENSG00000130363	RSPH3	1.53	1.53	0.61	1.27E-04	1.36E+02	1.36E+02
ENSG00000173614	NMNAT1	1.38	1.38	0.46	1.03E-04	1.36E+02	1.36E+02
ENSG00000049860	HEXB	1.36	1.36	0.45	1.01E-04	1.35E+02	1.35E+02
ENSG00000110811	LEPREL2	0.75	-1.34	-0.42	9.80E-05	-1.35E+02	1.35E+02
ENSG00000130755	GMFG	1.44	1.44	0.53	1.14E-04	1.35E+02	1.35E+02
ENSG00000170537	TMC7	1.47	1.47	0.56	1.19E-04	1.35E+02	1.35E+02
ENSG00000248334	WHAMMP2	1.42	1.42	0.51	1.11E-04	1.35E+02	1.35E+02
ENSG00000197948	FCHSD1	0.73	-1.38	-0.46	1.05E-04	-1.34E+02	1.34E+02
ENSG00000138678	AGPAT9	1.39	1.39	0.48	1.08E-04	1.34E+02	1.34E+02
	RP11-645N11.2	1.53	1.53	0.61	1.29E-04	1.34E+02	1.34E+02
ENSG00000071051	NCK2	1.34	1.34	0.42	9.96E-05	1.34E+02	1.34E+02
ENSG00000072062	PRKACA	0.73	-1.37	-0.45	1.05E-04	-1.34E+02	1.34E+02
ENSG00000112118	MCM3	0.74	-1.36	-0.44	1.03E-04	-1.34E+02	1.34E+02
ENSG00000182272	B4GALNT4	1.41	1.41	0.49	1.11E-04	1.34E+02	1.34E+02
ENSG00000228506	RP11-98I9.4	1.53	1.53	0.61	1.30E-04	1.34E+02	1.34E+02
ENSG00000176593	CTD-2368P22.1	1.54	1.54	0.63	1.33E-04	1.34E+02	1.34E+02
ENSG00000099797	TECR	0.74	-1.35	-0.43	1.03E-04	-1.33E+02	1.33E+02
ENSG00000147324	MFHAS1	1.34	1.34	0.43	1.04E-04	1.32E+02	1.32E+02
ENSG00000204176	SYT15	1.76	1.76	0.82	1.80E-04	1.31E+02	1.31E+02
ENSG00000113368	LMNB1	0.72	-1.38	-0.47	1.11E-04	-1.31E+02	1.31E+02
ENSG00000005884	ITGA3	1.46	1.46	0.54	1.24E-04	1.31E+02	1.31E+02
ENSG00000125744	RTN2	1.59	1.59	0.67	1.47E-04	1.31E+02	1.31E+02
ENSG00000187608	ISG15	0.71	-1.41	-0.50	1.17E-04	-1.31E+02	1.31E+02
ENSG00000165661	QSOX2	1.40	1.40	0.49	1.15E-04	1.31E+02	1.31E+02
ENSG00000242265	PEG10	0.75	-1.34	-0.42	1.05E-04	-1.31E+02	1.31E+02
ENSG00000163191	S100A11	1.35	1.35	0.43	1.08E-04	1.30E+02	1.30E+02
ENSG00000170775	GPR37	0.65	-1.54	-0.62	1.41E-04	-1.30E+02	1.30E+02
ENSG00000163625	WDFY3	1.43	1.43	0.52	1.22E-04	1.29E+02	1.29E+02
ENSG00000158023	WDR66	1.56	1.56	0.64	1.46E-04	1.29E+02	1.29E+02
ENSG00000259943	RP1-39G22.7	0.66	-1.51	-0.59	1.37E-04	-1.29E+02	1.29E+02
ENSG00000143319	ISG20L2	0.75	-1.33	-0.41	1.06E-04	-1.29E+02	1.29E+02
ENSG00000183020	AP2A2	1.35	1.35	0.44	1.11E-04	1.29E+02	1.29E+02
ENSG00000168116	KIAA1586	0.71	-1.42	-0.50	1.22E-04	-1.28E+02	1.28E+02
ENSG00000087253	LPCAT2	0.74	-1.35	-0.43	1.11E-04	-1.28E+02	1.28E+02
ENSG00000168090	COPS6	0.74	-1.35	-0.43	1.11E-04	-1.28E+02	1.28E+02
ENSG00000270022	RNU12	0.57	-1.75	-0.81	1.87E-04	-1.28E+02	1.28E+02
ENSG00000197635	DPP4	1.33	1.33	0.41	1.08E-04	1.27E+02	1.27E+02
ENSG00000142731	PLK4	0.71	-1.41	-0.50	1.24E-04	-1.27E+02	1.27E+02
ENSG00000135272	MDFIC	0.74	-1.35	-0.43	1.13E-04	-1.27E+02	1.27E+02
ENSG00000165914	TTC7B	1.44	1.44	0.52	1.28E-04	1.27E+02	1.27E+02
ENSG00000169925	BRD3	1.36	1.36	0.44	1.14E-04	1.27E+02	1.27E+02
ENSG00000232119	MCTS1	1.37	1.37	0.45	1.17E-04	1.26E+02	1.26E+02
ENSG00000138002	IFT172	0.73	-1.37	-0.46	1.19E-04	-1.26E+02	1.26E+02
ENSG00000172661	FAM21C	1.38	1.38	0.46	1.20E-04	1.26E+02	1.26E+02
ENSG00000164023	SGMS2	0.72	-1.38	-0.47	1.21E-04	-1.26E+02	1.26E+02
ENSG00000178761	FAM219B	0.75	-1.33	-0.42	1.13E-04	-1.25E+02	1.25E+02
ENSG00000151503	NCAPD3	0.74	-1.34	-0.43	1.15E-04	-1.25E+02	1.25E+02
ENSG00000182934	SRPR	0.70	-1.43	-0.51	1.30E-04	-1.25E+02	1.25E+02
ENSG00000156136	DCK	0.75	-1.33	-0.41	1.14E-04	-1.25E+02	1.25E+02
ENSG00000170653	ATF7	1.36	1.36	0.44	1.20E-04	1.24E+02	1.24E+02
ENSG00000157617	C2CD2	1.35	1.35	0.43	1.19E-04	1.24E+02	1.24E+02
ENSG00000146281	PM20D2	1.33	1.33	0.41	1.16E-04	1.24E+02	1.24E+02
ENSG00000102362	SYTL4	0.75	-1.33	-0.41	1.17E-04	-1.23E+02	1.23E+02
ENSG00000237292	RP11-540K16.1	1.62	1.62	0.70	1.74E-04	1.23E+02	1.23E+02
ENSG00000171992	SYNPO	1.43	1.43	0.52	1.36E-04	1.23E+02	1.23E+02
ENSG00000111665	CDCA3	0.71	-1.40	-0.49	1.31E-04	-1.23E+02	1.23E+02
ENSG00000175906	ARL4D	0.61	-1.63	-0.70	1.76E-04	-1.23E+02	1.23E+02
ENSG00000169826	CSGALNACT2	1.34	1.34	0.42	1.21E-04	1.22E+02	1.22E+02
ENSG00000169627	BOLA2B	0.55	-1.80	-0.85	2.20E-04	-1.22E+02	1.22E+02
ENSG00000270647	TAF15	0.72	-1.38	-0.47	1.32E-04	-1.20E+02	1.20E+02
ENSG00000054965	FAM168A	1.34	1.34	0.42	1.25E-04	1.20E+02	1.20E+02
ENSG00000181472	ZBTB2	0.76	-1.32	-0.41	1.22E-04	-1.20E+02	1.20E+02
ENSG00000144815	NXPE3	0.67	-1.50	-0.59	1.57E-04	-1.20E+02	1.20E+02
ENSG00000179981	TSHZ1	1.35	1.35	0.43	1.27E-04	1.20E+02	1.20E+02
ENSG00000184979	USP18	0.74	-1.35	-0.43	1.28E-04	-1.19E+02	1.19E+02
ENSG00000119632	IFI27L2	0.71	-1.41	-0.50	1.41E-04	-1.19E+02	1.19E+02
ENSG00000129003	VPS13C	1.76	1.76	0.82	2.20E-04	1.19E+02	1.19E+02
ENSG00000197879	MYO1C	0.74	-1.35	-0.44	1.30E-04	-1.18E+02	1.18E+02
ENSG00000171241	SHCBP1	0.74	-1.35	-0.44	1.31E-04	-1.18E+02	1.18E+02
ENSG00000204370	SDHD	0.73	-1.36	-0.45	1.33E-04	-1.18E+02	1.18E+02

ENSG00000246273	SBF2-AS1	1.68	1.68	0.75	2.02E-04	1.18E+02	1.18E+02
ENSG00000102218	RP2	1.35	1.35	0.44	1.32E-04	1.18E+02	1.18E+02
ENSG00000123600	METTL8	0.75	-1.33	-0.42	1.28E-04	-1.18E+02	1.18E+02
ENSG00000124786	SLC35B3	1.34	1.34	0.42	1.30E-04	1.18E+02	1.18E+02
ENSG00000153786	ZDHHC7	1.36	1.36	0.44	1.34E-04	1.17E+02	1.17E+02
ENSG00000156697	UTP14A	0.75	-1.33	-0.41	1.28E-04	-1.17E+02	1.17E+02
ENSG00000075275	CELSR1	1.60	1.60	0.68	1.87E-04	1.17E+02	1.17E+02
ENSG00000059377	TBXAS1	0.61	-1.63	-0.70	1.94E-04	-1.17E+02	1.17E+02
ENSG00000123870	ZNF137P	1.81	1.81	0.85	2.40E-04	1.17E+02	1.17E+02
ENSG00000089063	TMEM230	0.75	-1.34	-0.42	1.32E-04	-1.17E+02	1.17E+02
ENSG00000117748	RPA2	0.75	-1.33	-0.41	1.30E-04	-1.17E+02	1.17E+02
ENSG00000159720	ATP6V0D1	1.47	1.47	0.55	1.59E-04	1.16E+02	1.16E+02
ENSG00000146842	TMEM209	1.33	1.33	0.41	1.31E-04	1.16E+02	1.16E+02
ENSG00000055163	CYFIP2	1.36	1.36	0.44	1.37E-04	1.16E+02	1.16E+02
ENSG00000163453	IGFBP7	1.41	1.41	0.50	1.50E-04	1.16E+02	1.16E+02
ENSG00000185480	PARPBP	0.67	-1.49	-0.58	1.67E-04	-1.15E+02	1.15E+02
ENSG00000178719	GRINA	1.36	1.36	0.44	1.40E-04	1.15E+02	1.15E+02
ENSG00000197045	GMFB	1.46	1.46	0.54	1.62E-04	1.15E+02	1.15E+02
ENSG00000163517	HDAC11	1.42	1.42	0.51	1.54E-04	1.15E+02	1.15E+02
ENSG00000177352	CCDC71	0.70	-1.44	-0.52	1.58E-04	-1.15E+02	1.15E+02
ENSG00000102858	MGRN1	1.42	1.42	0.51	1.55E-04	1.14E+02	1.14E+02
ENSG00000185420	SMYD3	0.72	-1.39	-0.48	1.48E-04	-1.14E+02	1.14E+02
ENSG00000166582	CENPV	0.75	-1.33	-0.41	1.35E-04	-1.14E+02	1.14E+02
ENSG00000100425	BRD1	1.37	1.37	0.45	1.43E-04	1.14E+02	1.14E+02
ENSG00000099246	RAB18	1.37	1.37	0.45	1.44E-04	1.14E+02	1.14E+02
ENSG00000131477	RAMP2	1.45	1.45	0.54	1.62E-04	1.14E+02	1.14E+02
ENSG00000137331	IER3	0.74	-1.35	-0.43	1.41E-04	-1.14E+02	1.14E+02
ENSG00000123143	PKN1	1.41	1.41	0.49	1.54E-04	1.13E+02	1.13E+02
ENSG00000160789	LMNA	1.38	1.38	0.46	1.48E-04	1.13E+02	1.13E+02
ENSG00000109686	SH3D19	1.36	1.36	0.44	1.45E-04	1.12E+02	1.12E+02
ENSG00000175595	ERCC4	1.33	1.33	0.41	1.41E-04	1.12E+02	1.12E+02
ENSG00000225938	RP4-575N6.4	1.52	1.52	0.61	1.86E-04	1.12E+02	1.12E+02
ENSG00000222076	RNU2-3P	0.46	-2.19	-1.13	3.82E-04	-1.12E+02	1.12E+02
ENSG00000119383	PPP2R4	1.35	1.35	0.44	1.47E-04	1.12E+02	1.12E+02
ENSG00000166439	RNF169	1.34	1.34	0.43	1.45E-04	1.12E+02	1.12E+02
ENSG00000033327	GAB2	1.34	1.34	0.42	1.44E-04	1.12E+02	1.12E+02
ENSG00000013573	DDX11	0.73	-1.38	-0.46	1.53E-04	-1.11E+02	1.11E+02
ENSG00000104774	MAN2B1	0.66	-1.52	-0.60	1.87E-04	-1.11E+02	1.11E+02
ENSG00000160445	ZER1	1.36	1.36	0.44	1.49E-04	1.11E+02	1.11E+02
ENSG00000214087	ARL16	0.73	-1.37	-0.45	1.51E-04	-1.11E+02	1.11E+02
ENSG00000067221	STOML1	1.38	1.38	0.47	1.55E-04	1.11E+02	1.11E+02
ENSG00000136404	TM6SF1	1.33	1.33	0.41	1.44E-04	1.11E+02	1.11E+02
ENSG00000118162	KPTN	0.68	-1.48	-0.56	1.79E-04	-1.11E+02	1.11E+02
ENSG00000115942	ORC2	0.75	-1.33	-0.41	1.44E-04	-1.11E+02	1.11E+02
ENSG00000186088	PION	1.48	1.48	0.56	1.79E-04	1.10E+02	1.10E+02
ENSG00000234284	ZNF879	1.39	1.39	0.48	1.60E-04	1.10E+02	1.10E+02
ENSG00000076706	MCAM	1.36	1.36	0.44	1.52E-04	1.10E+02	1.10E+02
ENSG00000016864	GLT8D1	0.75	-1.33	-0.41	1.46E-04	-1.10E+02	1.10E+02
ENSG00000214114	MYCBP	0.75	-1.33	-0.41	1.47E-04	-1.10E+02	1.10E+02
ENSG00000164933	SLC25A32	0.75	-1.34	-0.42	1.49E-04	-1.10E+02	1.10E+02
ENSG00000128709	HOXD9	1.55	1.55	0.63	2.01E-04	1.09E+02	1.09E+02
ENSG00000141985	SH3GL1	1.36	1.36	0.44	1.55E-04	1.09E+02	1.09E+02
ENSG00000204934	ATP6V0E2-AS1	1.51	1.51	0.59	1.95E-04	1.08E+02	1.08E+02
ENSG00000170043	TRAPPC1	1.59	1.59	0.67	2.17E-04	1.08E+02	1.08E+02
ENSG00000121766	ZCCHC17	0.75	-1.33	-0.41	1.52E-04	-1.08E+02	1.08E+02
ENSG00000130731	C16orf13	0.75	-1.33	-0.41	1.52E-04	-1.08E+02	1.08E+02
ENSG00000115541	HSPE1	0.72	-1.38	-0.47	1.65E-04	-1.08E+02	1.08E+02
ENSG00000083312	TNPO1	1.43	1.43	0.52	1.77E-04	1.08E+02	1.08E+02
ENSG00000182541	LIMK2	1.34	1.34	0.43	1.59E-04	1.07E+02	1.07E+02
ENSG00000151287	TEX30	0.76	-1.32	-0.40	1.55E-04	-1.07E+02	1.07E+02
ENSG00000125843	AP5S1	0.73	-1.37	-0.46	1.67E-04	-1.06E+02	1.06E+02
ENSG00000179912	R3HDM2	1.39	1.39	0.48	1.72E-04	1.06E+02	1.06E+02
ENSG00000127481	UBR4	1.45	1.45	0.54	1.87E-04	1.06E+02	1.06E+02
ENSG00000126822	PLEKHG3	0.64	-1.56	-0.64	2.17E-04	-1.06E+02	1.06E+02
ENSG00000108604	SMARCD2	0.75	-1.34	-0.42	1.59E-04	-1.06E+02	1.06E+02
ENSG00000164713	BRI3	1.37	1.37	0.46	1.69E-04	1.05E+02	1.05E+02
ENSG00000103044	HAS3	0.37	-2.70	-1.43	6.61E-04	-1.05E+02	1.05E+02
ENSG00000139926	FRMD6	1.35	1.35	0.43	1.64E-04	1.05E+02	1.05E+02
ENSG00000206838	SNORA5A	1.83	1.83	0.87	3.07E-04	1.04E+02	1.04E+02
ENSG00000198598	MMP17	0.65	-1.53	-0.62	2.17E-04	-1.04E+02	1.04E+02
ENSG00000064666	CNN2	1.43	1.43	0.52	1.89E-04	1.04E+02	1.04E+02
ENSG00000148082	SHC3	1.91	1.91	0.94	3.37E-04	1.04E+02	1.04E+02
ENSG00000168394	TAP1	1.32	1.32	0.40	1.60E-04	1.04E+02	1.04E+02
ENSG00000104880	ARHGEF18	1.35	1.35	0.43	1.69E-04	1.04E+02	1.04E+02
ENSG00000162585	C1orf86	1.38	1.38	0.47	1.78E-04	1.04E+02	1.04E+02
ENSG00000233251	AC007743.1	1.37	1.37	0.45	1.75E-04	1.04E+02	1.04E+02
ENSG00000129968	FAM108A1	1.32	1.32	0.40	1.62E-04	1.03E+02	1.03E+02
ENSG00000099331	MYO9B	1.37	1.37	0.46	1.77E-04	1.03E+02	1.03E+02
ENSG00000197381	ADARB1	1.33	1.33	0.41	1.67E-04	1.03E+02	1.03E+02

ENSG00000025434	NR1H3	0.64	-1.57	-0.65	2.36E-04	-1.02E+02	1.02E+02
ENSG00000186395	KRT10	0.62	-1.62	-0.69	2.50E-04	-1.02E+02	1.02E+02
ENSG00000160336	ZNF761	1.41	1.41	0.50	1.90E-04	1.02E+02	1.02E+02
ENSG00000162396	PARS2	1.45	1.45	0.53	2.00E-04	1.02E+02	1.02E+02
ENSG00000071246	VASH1	1.34	1.34	0.42	1.73E-04	1.02E+02	1.02E+02
ENSG00000070087	PFN2	0.74	-1.35	-0.43	1.74E-04	-1.02E+02	1.02E+02
ENSG00000250588	IQCJ-SCHIP1	1.47	1.47	0.56	2.09E-04	1.02E+02	1.02E+02
ENSG00000132600	PRMT7	0.74	-1.35	-0.43	1.75E-04	-1.02E+02	1.02E+02
ENSG00000167535	CACNB3	0.75	-1.33	-0.41	1.71E-04	-1.02E+02	1.02E+02
ENSG00000157450	RNF111	1.34	1.34	0.42	1.74E-04	1.02E+02	1.02E+02
ENSG00000260336	RP11-395B7.7	1.47	1.47	0.55	2.11E-04	1.01E+02	1.01E+02
ENSG00000131495	NDUFA2	0.74	-1.35	-0.44	1.79E-04	-1.01E+02	1.01E+02
ENSG00000106603	COA1	1.32	1.32	0.40	1.71E-04	1.01E+02	1.01E+02
ENSG00000165102	HGSNAT	1.32	1.32	0.40	1.72E-04	1.01E+02	1.01E+02
ENSG00000123080	CDKN2C	0.73	-1.37	-0.46	1.86E-04	-1.01E+02	1.01E+02
ENSG00000180901	KCTD2	1.35	1.35	0.43	1.83E-04	9.99E+01	9.99E+01
ENSG00000132256	TRIM5	0.75	-1.34	-0.42	1.80E-04	-9.97E+01	9.97E+01
ENSG00000178913	TAF7	1.35	1.35	0.43	1.84E-04	9.93E+01	9.93E+01
ENSG00000174951	FUT1	0.73	-1.38	-0.46	1.94E-04	-9.89E+01	9.89E+01
ENSG00000069424	KCNAB2	1.65	1.65	0.72	2.80E-04	9.85E+01	9.85E+01
ENSG00000104549	SQLE	0.74	-1.35	-0.44	1.89E-04	-9.84E+01	9.84E+01
ENSG00000142178	SIK1	1.45	1.45	0.54	2.20E-04	9.80E+01	9.80E+01
ENSG00000149474	CSRP2BP	1.37	1.37	0.45	1.96E-04	9.78E+01	9.78E+01
ENSG00000048471	SNX29	1.37	1.37	0.45	1.97E-04	9.73E+01	9.73E+01
ENSG00000066379	ZNRD1	0.75	-1.33	-0.42	1.89E-04	-9.71E+01	9.71E+01
ENSG00000166002	C11orf75	0.65	-1.55	-0.63	2.57E-04	-9.66E+01	9.66E+01
ENSG00000200534	SNORA33	0.59	-1.69	-0.76	3.06E-04	-9.66E+01	9.66E+01
ENSG00000115317	HTRA2	1.32	1.32	0.40	1.87E-04	9.66E+01	9.66E+01
ENSG00000185973	TMLHE	0.73	-1.38	-0.46	2.04E-04	-9.64E+01	9.64E+01
ENSG00000139187	KLRG1	0.73	-1.37	-0.45	2.03E-04	-9.61E+01	9.61E+01
ENSG00000186130	ZBTB6	1.44	1.44	0.52	2.25E-04	9.59E+01	9.59E+01
ENSG00000246089	RP11-115C21.2	1.57	1.57	0.65	2.71E-04	9.56E+01	9.56E+01
ENSG00000151229	SLC2A13	1.44	1.44	0.52	2.27E-04	9.55E+01	9.55E+01
ENSG00000100104	SRRD	0.76	-1.32	-0.40	1.91E-04	-9.54E+01	9.54E+01
ENSG00000089050	RBBP9	0.76	-1.32	-0.40	1.93E-04	-9.53E+01	9.53E+01
ENSG00000137942	FNBP1L	1.37	1.37	0.46	2.09E-04	9.50E+01	9.50E+01
ENSG00000065000	AP3D1	1.37	1.37	0.45	2.07E-04	9.50E+01	9.50E+01
ENSG00000143845	ETNK2	0.73	-1.38	-0.46	2.12E-04	-9.47E+01	9.47E+01
ENSG00000099814	KIAA0284	1.35	1.35	0.43	2.05E-04	9.43E+01	9.43E+01
ENSG00000107738	C10orf54	1.60	1.60	0.68	2.89E-04	9.43E+01	9.43E+01
ENSG00000091317	CMTM6	0.73	-1.36	-0.45	2.09E-04	-9.43E+01	9.43E+01
ENSG00000175832	ETV4	1.33	1.33	0.41	2.01E-04	9.40E+01	9.40E+01
ENSG00000164187	LMBRD2	1.51	1.51	0.59	2.57E-04	9.40E+01	9.40E+01
ENSG00000116898	MRPS15	0.75	-1.34	-0.42	2.03E-04	-9.40E+01	9.40E+01
ENSG00000151611	MMAA	1.43	1.43	0.51	2.31E-04	9.40E+01	9.40E+01
ENSG00000115875	SRSF7	0.74	-1.35	-0.43	2.06E-04	-9.39E+01	9.39E+01
ENSG00000147202	DIAPH2	1.37	1.37	0.45	2.12E-04	9.39E+01	9.39E+01
ENSG00000006453	BAIAP2L1	1.50	1.50	0.58	2.55E-04	9.37E+01	9.37E+01
	RP11-1407O15.2	0.63	-1.57	-0.66	2.84E-04	-9.35E+01	9.35E+01
ENSG00000146263	MMS22L	0.75	-1.34	-0.42	2.04E-04	-9.35E+01	9.35E+01
ENSG00000153406	NMRAL1	0.76	-1.32	-0.40	1.98E-04	-9.34E+01	9.34E+01
ENSG00000110911	SLC11A2	0.75	-1.33	-0.41	2.01E-04	-9.34E+01	9.34E+01
ENSG00000135976	ANKRD36	1.37	1.37	0.45	2.14E-04	9.33E+01	9.33E+01
ENSG00000225792	AC004540.4	1.49	1.49	0.58	2.57E-04	9.31E+01	9.31E+01
ENSG00000264538	SUZ12P	0.69	-1.46	-0.54	2.46E-04	-9.30E+01	9.30E+01
ENSG00000116138	DNAJC16	1.32	1.32	0.40	2.04E-04	9.27E+01	9.27E+01
ENSG00000100941	PNN	0.76	-1.32	-0.41	2.05E-04	-9.26E+01	9.26E+01
ENSG00000164576	SAP30L	1.35	1.35	0.43	2.12E-04	9.25E+01	9.25E+01
ENSG00000104756	KCTD9	1.35	1.35	0.43	2.12E-04	9.24E+01	9.24E+01
ENSG00000182325	FBXL6	0.71	-1.41	-0.49	2.32E-04	-9.23E+01	9.23E+01
ENSG00000141499	WRAP53	0.73	-1.36	-0.45	2.21E-04	-9.17E+01	9.17E+01
ENSG00000153037	SRP19	0.73	-1.37	-0.46	2.25E-04	-9.16E+01	9.16E+01
ENSG00000070495	JMJD6	1.32	1.32	0.40	2.07E-04	9.15E+01	9.15E+01
ENSG00000100908	EMC9	0.73	-1.38	-0.46	2.27E-04	-9.14E+01	9.14E+01
ENSG00000176024	ZNF613	1.40	1.40	0.48	2.34E-04	9.12E+01	9.12E+01
ENSG00000184990	SIVA1	0.75	-1.33	-0.41	2.13E-04	-9.12E+01	9.12E+01
ENSG00000142279	WTIP	0.74	-1.35	-0.43	2.19E-04	-9.10E+01	9.10E+01
ENSG00000088256	GNA11	1.34	1.34	0.43	2.19E-04	9.09E+01	9.09E+01
ENSG00000169679	BUB1	0.75	-1.34	-0.42	2.18E-04	-9.09E+01	9.09E+01
ENSG00000148339	SLC25A25	0.76	-1.32	-0.40	2.12E-04	-9.07E+01	9.07E+01
ENSG00000139722	VPS37B	1.32	1.32	0.40	2.12E-04	9.06E+01	9.06E+01
ENSG00000089685	BIRC5	0.74	-1.34	-0.43	2.20E-04	-9.06E+01	9.06E+01
ENSG00000133083	DCLK1	0.63	-1.59	-0.67	3.08E-04	-9.06E+01	9.06E+01
ENSG00000137575	SDCBP	1.42	1.42	0.51	2.46E-04	9.06E+01	9.06E+01
ENSG00000110700	RPS13	0.73	-1.37	-0.45	2.28E-04	-9.04E+01	9.04E+01
ENSG00000112249	ASCC3	1.48	1.48	0.57	2.68E-04	9.04E+01	9.04E+01
ENSG00000172733	PURG	1.55	1.55	0.63	2.94E-04	9.04E+01	9.04E+01
ENSG00000068903	SIRT2	1.33	1.33	0.42	2.18E-04	9.03E+01	9.03E+01
ENSG00000188229	TUBB4B	0.74	-1.35	-0.43	2.24E-04	-9.02E+01	9.02E+01

ENSG00000118263	KLF7	1.36	1.36	0.44	2.26E-04	9.02E+01	9.02E+01
ENSG00000149311	ATM	1.60	1.60	0.68	3.17E-04	9.02E+01	9.02E+01
ENSG00000083720	OXCT1	0.75	-1.33	-0.41	2.18E-04	-8.99E+01	8.99E+01
ENSG00000111961	SASH1	1.35	1.35	0.43	2.26E-04	8.97E+01	8.97E+01
ENSG00000171488	LRRC8C	1.34	1.34	0.42	2.23E-04	8.96E+01	8.96E+01
ENSG00000170989	S1PR1	1.34	1.34	0.42	2.24E-04	8.94E+01	8.94E+01
ENSG00000203326	ZNF525	1.47	1.47	0.56	2.72E-04	8.93E+01	8.93E+01
ENSG00000182158	CREB3L2	1.35	1.35	0.43	2.29E-04	8.93E+01	8.93E+01
ENSG00000148700	ADD3	1.36	1.36	0.44	2.30E-04	8.93E+01	8.93E+01
ENSG00000169871	TRIM56	1.38	1.38	0.46	2.38E-04	8.92E+01	8.92E+01
ENSG00000119471	HSDL2	0.76	-1.32	-0.40	2.20E-04	-8.91E+01	8.91E+01
ENSG00000185596	WASH3P	1.40	1.40	0.49	2.48E-04	8.91E+01	8.91E+01
ENSG00000110011	DNAJC4	0.72	-1.39	-0.48	2.44E-04	-8.91E+01	8.91E+01
ENSG00000092969	TGFB2	1.38	1.38	0.46	2.39E-04	8.91E+01	8.91E+01
ENSG00000169255	B3GALNT1	0.74	-1.35	-0.43	2.29E-04	-8.90E+01	8.90E+01
ENSG00000247498	RP11-392P7.6	0.65	-1.53	-0.62	2.98E-04	-8.88E+01	8.88E+01
ENSG00000135316	SYNCRIP	0.74	-1.35	-0.43	2.32E-04	-8.86E+01	8.86E+01
ENSG00000053501	USE1	0.69	-1.46	-0.54	2.71E-04	-8.85E+01	8.85E+01
ENSG00000214253	FIS1	0.74	-1.35	-0.43	2.32E-04	-8.84E+01	8.84E+01
ENSG00000183814	LIN9	0.71	-1.40	-0.49	2.52E-04	-8.84E+01	8.84E+01
ENSG00000164692	COL1A2	0.56	-1.77	-0.83	4.03E-04	-8.82E+01	8.82E+01
ENSG00000150048	CLEC1A	0.75	-1.33	-0.41	2.29E-04	-8.78E+01	8.78E+01
ENSG00000073146	MOV10L1	0.75	-1.34	-0.42	2.33E-04	-8.77E+01	8.77E+01
ENSG00000076382	SPAG5	0.73	-1.37	-0.45	2.43E-04	-8.76E+01	8.76E+01
ENSG00000101191	DIDO1	1.36	1.36	0.44	2.41E-04	8.75E+01	8.75E+01
ENSG00000007944	MYLIP	0.71	-1.41	-0.49	2.59E-04	-8.74E+01	8.74E+01
ENSG00000234465	PINLYP	0.58	-1.73	-0.79	3.90E-04	-8.74E+01	8.74E+01
ENSG00000099194	SCD	0.74	-1.36	-0.44	2.41E-04	-8.74E+01	8.74E+01
ENSG00000099917	MED15	1.32	1.32	0.40	2.28E-04	8.73E+01	8.73E+01
ENSG00000160613	PCSK7	0.76	-1.31	-0.39	2.25E-04	-8.72E+01	8.72E+01
ENSG00000166822	TMEM170A	0.75	-1.34	-0.42	2.37E-04	-8.70E+01	8.70E+01
ENSG00000197122	SRC	1.32	1.32	0.40	2.31E-04	8.68E+01	8.68E+01
ENSG00000171453	POLR1C	0.76	-1.32	-0.40	2.33E-04	-8.62E+01	8.62E+01
ENSG00000183250	C21orf67	1.62	1.62	0.70	3.54E-04	8.62E+01	8.62E+01
ENSG00000197958	RPL12	0.75	-1.34	-0.42	2.42E-04	-8.62E+01	8.62E+01
ENSG00000197019	SERTAD1	0.73	-1.37	-0.45	2.53E-04	-8.62E+01	8.62E+01
ENSG00000171812	COL8A2	0.64	-1.55	-0.64	3.25E-04	-8.61E+01	8.61E+01
ENSG00000116747	TROVE2	1.59	1.59	0.67	3.40E-04	8.61E+01	8.61E+01
ENSG00000155252	PI4K2A	1.31	1.31	0.39	2.32E-04	8.59E+01	8.59E+01
ENSG00000151500	THYN1	0.75	-1.33	-0.41	2.39E-04	-8.58E+01	8.58E+01
ENSG00000139832	RAB20	0.58	-1.72	-0.79	4.04E-04	-8.58E+01	8.58E+01
ENSG00000166171	DPCD	0.75	-1.33	-0.41	2.40E-04	-8.58E+01	8.58E+01
ENSG00000005243	COPZ2	0.69	-1.44	-0.53	2.84E-04	-8.56E+01	8.56E+01
ENSG00000137776	SLTM	1.37	1.37	0.45	2.56E-04	8.56E+01	8.56E+01
ENSG00000198053	SIRPA	1.35	1.35	0.43	2.49E-04	8.55E+01	8.55E+01
ENSG00000183853	KIRREL	1.45	1.45	0.54	2.89E-04	8.53E+01	8.53E+01
ENSG00000099810	MTAP	0.76	-1.32	-0.40	2.40E-04	-8.51E+01	8.51E+01
ENSG00000161654	LSM12	0.75	-1.33	-0.41	2.46E-04	-8.47E+01	8.47E+01
ENSG00000199161	MIR126	0.46	-2.17	-1.12	6.55E-04	-8.47E+01	8.47E+01
ENSG00000104518	GSDMD	0.75	-1.33	-0.41	2.49E-04	-8.44E+01	8.44E+01
ENSG00000145029	NICN1	0.68	-1.46	-0.55	3.01E-04	-8.42E+01	8.42E+01
ENSG00000105373	GLTSCR2	0.75	-1.34	-0.42	2.53E-04	-8.42E+01	8.42E+01
ENSG00000166435	XRRA1	0.68	-1.46	-0.55	3.02E-04	-8.40E+01	8.40E+01
ENSG00000184178	SCFD2	0.76	-1.32	-0.40	2.50E-04	-8.35E+01	8.35E+01
ENSG00000196159	FAT4	1.34	1.34	0.42	2.57E-04	8.34E+01	8.34E+01
ENSG00000109861	CTSC	0.74	-1.34	-0.43	2.61E-04	-8.32E+01	8.32E+01
ENSG00000183688	FAM101B	1.33	1.33	0.41	2.55E-04	8.31E+01	8.31E+01
ENSG00000055950	MRPL43	0.76	-1.31	-0.39	2.51E-04	-8.28E+01	8.28E+01
ENSG00000152465	NMT2	1.35	1.35	0.43	2.66E-04	8.28E+01	8.28E+01
ENSG00000143862	ARL8A	1.33	1.33	0.41	2.61E-04	8.26E+01	8.26E+01
ENSG00000115365	LANCL1	0.73	-1.36	-0.45	2.73E-04	-8.26E+01	8.26E+01
ENSG00000159399	HK2	0.72	-1.40	-0.48	2.88E-04	-8.23E+01	8.23E+01
ENSG00000138069	RAB1A	1.35	1.35	0.43	2.70E-04	8.22E+01	8.22E+01
ENSG00000216937	CCDC7	1.64	1.64	0.72	4.02E-04	8.19E+01	8.19E+01
ENSG00000196636	ACN9	0.68	-1.47	-0.55	3.24E-04	-8.16E+01	8.16E+01
ENSG00000127445	PIN1	0.75	-1.34	-0.42	2.70E-04	-8.15E+01	8.15E+01
ENSG00000273983	HIST1H3G	0.73	-1.36	-0.45	2.81E-04	-8.14E+01	8.14E+01
ENSG00000140750	ARHGAP17	1.49	1.49	0.58	3.38E-04	8.13E+01	8.13E+01
ENSG00000156026	MCU	0.74	-1.34	-0.43	2.74E-04	-8.13E+01	8.13E+01
ENSG00000215256	DHRS4-AS1	0.70	-1.42	-0.51	3.06E-04	-8.12E+01	8.12E+01
ENSG00000069122	GPR116	1.35	1.35	0.44	2.79E-04	8.11E+01	8.11E+01
ENSG00000167977	KCTD5	1.31	1.31	0.39	2.61E-04	8.10E+01	8.10E+01
ENSG00000144118	RALB	0.75	-1.34	-0.42	2.74E-04	-8.10E+01	8.10E+01
ENSG00000198198	SZT2	1.33	1.33	0.41	2.71E-04	8.07E+01	8.07E+01
ENSG00000177426	TGIF1	1.32	1.32	0.40	2.69E-04	8.03E+01	8.03E+01
ENSG00000167670	CHAF1A	0.74	-1.34	-0.43	2.80E-04	-8.02E+01	8.02E+01
ENSG00000155640	C10orf12	1.34	1.34	0.42	2.79E-04	8.00E+01	8.00E+01
ENSG00000027075	PRKCH	1.34	1.34	0.42	2.79E-04	8.00E+01	8.00E+01
ENSG00000131368	MRPS25	0.76	-1.31	-0.39	2.69E-04	-8.00E+01	8.00E+01

ENSG00000001630	CYP51A1	0.66	-1.53	-0.61	3.64E-04	-7.99E+01	7.99E+01
ENSG00000131100	ATP6V1E1	1.33	1.33	0.41	2.76E-04	7.99E+01	7.99E+01
ENSG00000001497	LAS1L	0.76	-1.31	-0.39	2.71E-04	-7.99E+01	7.99E+01
ENSG00000184916	JAG2	1.32	1.32	0.40	2.73E-04	7.98E+01	7.98E+01
ENSG00000117868	ESYT2	1.34	1.34	0.42	2.82E-04	7.96E+01	7.96E+01
ENSG00000130956	HABP4	1.35	1.35	0.43	2.87E-04	7.95E+01	7.95E+01
ENSG00000175455	CCDC14	0.71	-1.40	-0.49	3.12E-04	-7.93E+01	7.93E+01
ENSG00000100284	TOM1	1.33	1.33	0.41	2.82E-04	7.92E+01	7.92E+01
ENSG00000159202	UBE2Z	0.75	-1.33	-0.41	2.81E-04	-7.91E+01	7.91E+01
ENSG00000137500	CCDC90B	1.33	1.33	0.41	2.81E-04	7.91E+01	7.91E+01
ENSG00000154760	SLFN13	0.63	-1.57	-0.66	3.98E-04	-7.89E+01	7.89E+01
ENSG00000182983	ZNF662	0.60	-1.67	-0.74	4.48E-04	-7.87E+01	7.87E+01
ENSG00000112306	RPS12	0.72	-1.38	-0.47	3.09E-04	-7.86E+01	7.86E+01
ENSG00000150477	KIAA1328	1.40	1.40	0.48	3.17E-04	7.85E+01	7.85E+01
ENSG00000070367	EXOC5	1.52	1.52	0.60	3.75E-04	7.84E+01	7.84E+01
ENSG00000106537	TSPAN13	1.39	1.39	0.47	3.14E-04	7.84E+01	7.84E+01
ENSG00000205659	LIN52	0.74	-1.35	-0.43	2.98E-04	-7.83E+01	7.83E+01
ENSG00000007255	TRAPPC6A	1.41	1.41	0.50	3.27E-04	7.82E+01	7.82E+01
ENSG00000264469	RP11-173M1.8	0.67	-1.50	-0.59	3.69E-04	-7.81E+01	7.81E+01
ENSG00000113273	ARSB	1.31	1.31	0.39	2.83E-04	7.79E+01	7.79E+01
ENSG00000167702	KIFC2	1.43	1.43	0.51	3.36E-04	7.78E+01	7.78E+01
ENSG00000065057	NTHL1	0.76	-1.32	-0.40	2.88E-04	-7.78E+01	7.78E+01
ENSG00000166949	SMAD3	1.39	1.39	0.48	3.22E-04	7.76E+01	7.76E+01
ENSG00000179361	ARID3B	1.42	1.42	0.51	3.37E-04	7.74E+01	7.74E+01
ENSG00000133657	ATP13A3	1.32	1.32	0.41	2.93E-04	7.74E+01	7.74E+01
ENSG00000164114	MAP9	1.85	1.85	0.89	5.74E-04	7.72E+01	7.72E+01
ENSG00000110987	BCL7A	1.35	1.35	0.44	3.08E-04	7.71E+01	7.71E+01
ENSG00000073969	NSF	0.76	-1.31	-0.39	2.91E-04	-7.68E+01	7.68E+01
ENSG00000162836	ACP6	1.41	1.41	0.49	3.39E-04	7.66E+01	7.66E+01
ENSG00000187951	ARHGAP11B	0.67	-1.48	-0.57	3.78E-04	-7.63E+01	7.63E+01
ENSG00000166454	ATMIN	1.33	1.33	0.41	3.03E-04	7.61E+01	7.61E+01
ENSG00000166946	CCNDBP1	0.76	-1.31	-0.39	2.97E-04	-7.60E+01	7.60E+01
ENSG00000257727	CNPY2	0.75	-1.34	-0.42	3.10E-04	-7.59E+01	7.59E+01
ENSG00000075568	TMEM131	1.36	1.36	0.44	3.21E-04	7.59E+01	7.59E+01
ENSG00000120868	APAF1	1.33	1.33	0.41	3.08E-04	7.58E+01	7.58E+01
ENSG00000139324	TMTC3	1.61	1.61	0.69	4.52E-04	7.58E+01	7.58E+01
ENSG00000267383	CTC-260E6.6	1.54	1.54	0.62	4.12E-04	7.57E+01	7.57E+01
ENSG00000143179	UCK2	0.75	-1.33	-0.42	3.10E-04	-7.57E+01	7.57E+01
ENSG00000158352	SHROOM4	1.33	1.33	0.41	3.07E-04	7.57E+01	7.57E+01
ENSG00000160360	GPSM1	1.32	1.32	0.40	3.04E-04	7.56E+01	7.56E+01
ENSG00000124486	USP9X	1.34	1.34	0.43	3.15E-04	7.56E+01	7.56E+01
ENSG00000139278	GLIPR1	1.41	1.41	0.50	3.48E-04	7.56E+01	7.56E+01
ENSG00000116151	MORN1	1.56	1.56	0.64	4.27E-04	7.55E+01	7.55E+01
ENSG00000176371	ZSCAN2	1.33	1.33	0.41	3.11E-04	7.55E+01	7.55E+01
ENSG00000150967	ABCB9	1.34	1.34	0.42	3.16E-04	7.54E+01	7.54E+01
ENSG00000139977	NAA30	1.32	1.32	0.40	3.08E-04	7.54E+01	7.54E+01
ENSG00000204580	DDR1	0.72	-1.40	-0.48	3.46E-04	-7.52E+01	7.52E+01
ENSG00000089060	SLC24A6	1.31	1.31	0.39	3.04E-04	7.51E+01	7.51E+01
ENSG00000114867	EIF4G1	1.45	1.45	0.54	3.75E-04	7.50E+01	7.50E+01
ENSG00000146410	FAM54A	0.69	-1.45	-0.54	3.77E-04	-7.49E+01	7.49E+01
ENSG00000172671	ZFAND4	0.71	-1.40	-0.49	3.50E-04	-7.49E+01	7.49E+01
ENSG00000100647	KIAA0247	1.32	1.32	0.40	3.10E-04	7.48E+01	7.48E+01
ENSG00000158828	PINK1	1.35	1.35	0.44	3.28E-04	7.48E+01	7.48E+01
	ABC7-42389800N19.1	1.44	1.44	0.53	3.72E-04	7.47E+01	7.47E+01
ENSG00000269825	CTD-3099C6.9	0.73	-1.38	-0.46	3.40E-04	-7.47E+01	7.47E+01
ENSG00000176225	RTTN	0.75	-1.34	-0.42	3.20E-04	-7.46E+01	7.46E+01
ENSG00000139372	TDG	0.76	-1.31	-0.39	3.09E-04	-7.46E+01	7.46E+01
ENSG00000136014	USP44	0.61	-1.63	-0.70	4.77E-04	-7.45E+01	7.45E+01
ENSG00000127418	FGFRL1	1.50	1.50	0.58	4.05E-04	7.44E+01	7.44E+01
ENSG00000101782	RIOK3	1.33	1.33	0.41	3.19E-04	7.44E+01	7.44E+01
ENSG00000168564	CDKN2AIP	1.31	1.31	0.39	3.10E-04	7.44E+01	7.44E+01
ENSG00000203705	TATDN3	1.33	1.33	0.41	3.18E-04	7.43E+01	7.43E+01
	RP6-109B7.3	0.69	-1.45	-0.53	3.80E-04	-7.42E+01	7.42E+01
ENSG00000284038	hsa-mir-10a	1.59	1.59	0.67	4.62E-04	7.40E+01	7.40E+01
ENSG00000095261	PSMD5	0.75	-1.33	-0.42	3.25E-04	-7.40E+01	7.40E+01
ENSG00000213096	ZNF254	1.33	1.33	0.41	3.23E-04	7.40E+01	7.40E+01
ENSG00000174125	TLR1	0.65	-1.53	-0.61	4.32E-04	-7.37E+01	7.37E+01
ENSG00000158793	NIT1	0.74	-1.35	-0.43	3.35E-04	-7.36E+01	7.36E+01
ENSG00000237441	RGL2	0.77	-1.30	-0.38	3.13E-04	-7.35E+01	7.35E+01
ENSG00000122550	KLHL7	1.31	1.31	0.39	3.20E-04	7.34E+01	7.34E+01
ENSG00000101596	SMCHD1	1.47	1.47	0.56	4.04E-04	7.34E+01	7.34E+01
ENSG00000149499	EML3	0.76	-1.31	-0.39	3.21E-04	-7.32E+01	7.32E+01
ENSG00000160551	TAOK1	1.37	1.37	0.46	3.54E-04	7.30E+01	7.30E+01
ENSG00000177685	EFCAB4A	0.59	-1.69	-0.76	5.37E-04	-7.30E+01	7.30E+01
ENSG00000006125	AP2B1	1.37	1.37	0.46	3.53E-04	7.30E+01	7.30E+01
ENSG00000273802	HIST1H2BG	0.74	-1.35	-0.43	3.43E-04	-7.29E+01	7.29E+01
ENSG00000010327	STAB1	1.34	1.34	0.42	3.37E-04	7.28E+01	7.28E+01
ENSG00000119787	ATL2	0.76	-1.32	-0.40	3.27E-04	-7.28E+01	7.28E+01
ENSG00000146592	CREB5	1.40	1.40	0.49	3.72E-04	7.27E+01	7.27E+01

ENSG00000168495	POLR3D	1.30	1.30	0.38	3.23E-04	7.26E+01	7.26E+01
ENSG00000040199	PHLPP2	0.74	-1.35	-0.43	3.46E-04	-7.26E+01	7.26E+01
ENSG00000206503	HLA-A	1.34	1.34	0.42	3.42E-04	7.24E+01	7.24E+01
ENSG00000155016	CYP2U1	0.73	-1.36	-0.45	3.55E-04	-7.23E+01	7.23E+01
ENSG00000079102	RUNX1T1	0.75	-1.33	-0.41	3.38E-04	-7.22E+01	7.22E+01
ENSG00000138134	STAMBPL1	0.61	-1.64	-0.72	5.19E-04	-7.21E+01	7.21E+01
ENSG00000072364	AFF4	1.33	1.33	0.42	3.42E-04	7.21E+01	7.21E+01
ENSG00000067225	PKM	1.37	1.37	0.46	3.64E-04	7.20E+01	7.20E+01
ENSG00000197362	ZNF786	0.72	-1.40	-0.48	3.77E-04	-7.19E+01	7.19E+01
ENSG00000198824	CHAMP1	0.72	-1.39	-0.47	3.74E-04	-7.17E+01	7.17E+01
ENSG00000100462	PRMT5	0.76	-1.31	-0.39	3.37E-04	-7.17E+01	7.17E+01
ENSG00000268516	CTD-3138B18.5	0.60	-1.67	-0.74	5.46E-04	-7.16E+01	7.16E+01
ENSG00000182718	ANXA2	1.33	1.33	0.41	3.44E-04	7.16E+01	7.16E+01
ENSG00000118965	WDR35	1.34	1.34	0.42	3.50E-04	7.15E+01	7.15E+01
ENSG00000172954	LCLAT1	1.34	1.34	0.43	3.54E-04	7.13E+01	7.13E+01
ENSG00000198040	ZNF84	1.36	1.36	0.45	3.66E-04	7.13E+01	7.13E+01
ENSG00000204619	PPP1R11	0.76	-1.31	-0.39	3.37E-04	-7.12E+01	7.12E+01
ENSG00000135211	TMEM60	0.74	-1.35	-0.43	3.61E-04	-7.11E+01	7.11E+01
ENSG00000268912	CTD-2619J13.17	1.66	1.66	0.73	5.48E-04	7.10E+01	7.10E+01
ENSG00000197647	ZNF433	0.63	-1.59	-0.67	5.05E-04	-7.09E+01	7.09E+01
ENSG00000121310	ECHDC2	0.75	-1.33	-0.41	3.54E-04	-7.05E+01	7.05E+01
ENSG00000196267	ZNF836	1.42	1.42	0.50	4.04E-04	7.05E+01	7.05E+01
ENSG00000102452	NALCN	0.73	-1.36	-0.45	3.77E-04	-7.03E+01	7.03E+01
ENSG00000186314	PRELID2	0.74	-1.35	-0.43	3.69E-04	-7.03E+01	7.03E+01
ENSG00000101003	GINS1	0.74	-1.35	-0.43	3.68E-04	-7.02E+01	7.02E+01
ENSG00000139343	SNRPF	0.75	-1.34	-0.42	3.65E-04	-7.01E+01	7.01E+01
ENSG00000253352	TUG1	1.34	1.34	0.42	3.63E-04	7.01E+01	7.01E+01
	RP11-473M20.14	1.44	1.44	0.53	4.23E-04	7.01E+01	7.01E+01
ENSG00000102796	DHRS12	1.48	1.48	0.56	4.45E-04	7.01E+01	7.01E+01
ENSG00000128805	ARHGAP22	1.31	1.31	0.39	3.51E-04	6.99E+01	6.99E+01
ENSG00000177889	UBE2N	0.76	-1.32	-0.40	3.58E-04	-6.98E+01	6.98E+01
ENSG00000140521	POLG	0.75	-1.33	-0.41	3.64E-04	-6.96E+01	6.96E+01
ENSG00000167733	HSD11B1L	0.61	-1.63	-0.70	5.47E-04	-6.95E+01	6.95E+01
ENSG00000254685	FPGT	1.39	1.39	0.48	4.04E-04	6.94E+01	6.94E+01
ENSG00000197324	LRP10	1.39	1.39	0.48	4.05E-04	6.93E+01	6.93E+01
ENSG00000115211	EIF2B4	0.77	-1.30	-0.38	3.54E-04	-6.92E+01	6.92E+01
ENSG00000247287	RP11-902B17.1	1.45	1.45	0.54	4.44E-04	6.90E+01	6.90E+01
ENSG00000147050	KDM6A	1.31	1.31	0.39	3.58E-04	6.90E+01	6.90E+01
ENSG00000103145	HCFC1R1	0.74	-1.36	-0.44	3.90E-04	-6.89E+01	6.89E+01
ENSG00000100319	ZMAT5	1.35	1.35	0.43	3.82E-04	6.89E+01	6.89E+01
ENSG00000100307	CBX7	0.59	-1.70	-0.76	6.08E-04	-6.88E+01	6.88E+01
ENSG00000187741	FANCA	0.74	-1.35	-0.44	3.90E-04	-6.86E+01	6.86E+01
ENSG00000154803	FLCN	1.30	1.30	0.38	3.60E-04	6.85E+01	6.85E+01
ENSG00000172366	FAM195A	0.74	-1.35	-0.43	3.87E-04	-6.85E+01	6.85E+01
ENSG00000090612	ZNF268	1.63	1.63	0.70	5.64E-04	6.84E+01	6.84E+01
ENSG00000179526	SHARPIN	0.75	-1.33	-0.41	3.78E-04	-6.84E+01	6.84E+01
ENSG00000127720	METTL25	0.73	-1.38	-0.46	4.06E-04	-6.83E+01	6.83E+01
ENSG00000059588	TARBP1	0.77	-1.31	-0.38	3.66E-04	-6.83E+01	6.83E+01
ENSG00000040487	PQLC2	1.38	1.38	0.46	4.08E-04	6.82E+01	6.82E+01
ENSG00000129911	KLF16	1.33	1.33	0.41	3.83E-04	6.81E+01	6.81E+01
ENSG00000114904	NEK4	1.33	1.33	0.41	3.83E-04	6.79E+01	6.79E+01
ENSG00000153046	CDYL	1.32	1.32	0.41	3.83E-04	6.77E+01	6.77E+01
ENSG00000156515	HK1	1.39	1.39	0.48	4.24E-04	6.75E+01	6.75E+01
ENSG00000143891	GALM	0.74	-1.36	-0.44	4.05E-04	-6.74E+01	6.74E+01
ENSG00000122034	GTF3A	1.32	1.32	0.40	3.86E-04	6.74E+01	6.74E+01
ENSG00000145293	ENOPH1	0.76	-1.32	-0.40	3.82E-04	-6.73E+01	6.73E+01
ENSG00000160679	CHTOP	0.75	-1.34	-0.42	3.96E-04	-6.71E+01	6.71E+01
ENSG00000215305	VPS16	0.77	-1.30	-0.38	3.77E-04	-6.71E+01	6.71E+01
ENSG00000197363	ZNF517	1.52	1.52	0.60	5.16E-04	6.69E+01	6.69E+01
ENSG00000162433	AK4	0.74	-1.35	-0.43	4.05E-04	-6.68E+01	6.68E+01
ENSG00000138614	VWA9	0.76	-1.31	-0.39	3.84E-04	-6.68E+01	6.68E+01
ENSG00000196655	TRAPPC4	0.76	-1.32	-0.40	3.92E-04	-6.68E+01	6.68E+01
ENSG00000157483	MYO1E	1.32	1.32	0.40	3.92E-04	6.68E+01	6.68E+01
ENSG00000123240	OPTN	1.32	1.32	0.40	3.92E-04	6.67E+01	6.67E+01
ENSG00000211450	C11orf31	0.75	-1.33	-0.41	3.98E-04	-6.67E+01	6.67E+01
ENSG00000181031	RPH3AL	0.69	-1.46	-0.54	4.78E-04	-6.66E+01	6.66E+01
ENSG00000100147	CCDC134	0.70	-1.44	-0.52	4.67E-04	-6.65E+01	6.65E+01
ENSG00000118257	NRP2	1.38	1.38	0.47	4.33E-04	6.64E+01	6.64E+01
ENSG00000165233	C9orf89	1.32	1.32	0.40	3.95E-04	6.64E+01	6.64E+01
ENSG00000234072	AC074117.10	1.49	1.49	0.57	5.01E-04	6.63E+01	6.63E+01
ENSG00000153140	CETN3	1.34	1.34	0.42	4.10E-04	6.61E+01	6.61E+01
ENSG00000213853	EMP2	0.77	-1.30	-0.38	3.91E-04	-6.60E+01	6.60E+01
ENSG00000135535	CD164	1.37	1.37	0.45	4.33E-04	6.59E+01	6.59E+01
ENSG00000069956	MAPK6	1.49	1.49	0.58	5.14E-04	6.58E+01	6.58E+01
ENSG00000154122	ANKH	1.32	1.32	0.40	4.02E-04	6.57E+01	6.57E+01
ENSG00000143702	CEP170	1.51	1.51	0.60	5.35E-04	6.55E+01	6.55E+01
ENSG00000198538	ZNF28	1.42	1.42	0.51	4.75E-04	6.53E+01	6.53E+01
ENSG00000111981	ULBP1	1.44	1.44	0.52	4.86E-04	6.53E+01	6.53E+01
ENSG00000087269	NOP14	0.75	-1.33	-0.41	4.16E-04	-6.50E+01	6.50E+01

ENSG00000088832	FKBP1A	1.34	1.34	0.42	4.23E-04	6.49E+01	6.49E+01
ENSG00000100364	KIAA0930	1.31	1.31	0.39	4.09E-04	6.47E+01	6.47E+01
ENSG00000197461	PDGFA	1.30	1.30	0.38	4.08E-04	6.46E+01	6.46E+01
ENSG00000121895	TMEM156	1.47	1.47	0.56	5.21E-04	6.45E+01	6.45E+01
ENSG00000139083	ETV6	1.31	1.31	0.39	4.13E-04	6.45E+01	6.45E+01
ENSG00000128294	TPST2	1.30	1.30	0.38	4.10E-04	6.44E+01	6.44E+01
ENSG00000119705	SLIRP	0.74	-1.35	-0.43	4.39E-04	-6.44E+01	6.44E+01
ENSG00000165240	ATP7A	0.77	-1.31	-0.38	4.12E-04	-6.44E+01	6.44E+01
ENSG00000223768	LINC00205	1.36	1.36	0.44	4.47E-04	6.43E+01	6.43E+01
ENSG00000122863	CHST3	1.31	1.31	0.39	4.14E-04	6.42E+01	6.42E+01
ENSG00000171425	ZNF581	0.73	-1.37	-0.45	4.55E-04	-6.42E+01	6.42E+01
ENSG00000084734	GCKR	0.65	-1.54	-0.62	5.76E-04	-6.40E+01	6.40E+01
ENSG00000214013	GANC	1.32	1.32	0.40	4.25E-04	6.40E+01	6.40E+01
ENSG00000196549	MME	1.40	1.40	0.49	4.82E-04	6.39E+01	6.39E+01
ENSG00000145494	NDUFS6	0.75	-1.33	-0.41	4.37E-04	-6.38E+01	6.38E+01
ENSG00000105983	LMBR1	1.34	1.34	0.42	4.41E-04	6.37E+01	6.37E+01
ENSG00000167470	MIDN	1.37	1.37	0.46	4.63E-04	6.37E+01	6.37E+01
ENSG00000198429	ZNF69	1.52	1.52	0.60	5.68E-04	6.36E+01	6.36E+01
ENSG00000249614	RP11-703G6.1	1.90	1.90	0.93	8.94E-04	6.35E+01	6.35E+01
ENSG00000168411	RFWD3	0.76	-1.31	-0.39	4.28E-04	-6.34E+01	6.34E+01
ENSG00000160049	DFFA	0.76	-1.32	-0.40	4.33E-04	-6.32E+01	6.32E+01
ENSG00000150433	TMEM218	0.76	-1.31	-0.39	4.33E-04	-6.32E+01	6.32E+01
ENSG00000198464	ZNF480	1.34	1.34	0.43	4.54E-04	6.30E+01	6.30E+01
ENSG00000168389	MFSD2A	0.68	-1.47	-0.55	5.44E-04	-6.30E+01	6.30E+01
ENSG00000196437	ZNF569	1.45	1.45	0.54	5.31E-04	6.29E+01	6.29E+01
ENSG00000166275	C10orf32	0.74	-1.34	-0.43	4.57E-04	-6.28E+01	6.28E+01
ENSG00000107819	SFXN3	1.31	1.31	0.39	4.33E-04	6.28E+01	6.28E+01
ENSG00000162599	NFIA	0.77	-1.30	-0.38	4.30E-04	-6.28E+01	6.28E+01
ENSG00000149557	FEZ1	1.31	1.31	0.39	4.37E-04	6.26E+01	6.26E+01
ENSG00000205084	TMEM231	0.73	-1.38	-0.46	4.87E-04	-6.25E+01	6.25E+01
ENSG00000164182	NDUFAF2	0.77	-1.30	-0.38	4.35E-04	-6.24E+01	6.24E+01
ENSG00000156787	WDR67	0.74	-1.36	-0.44	4.73E-04	-6.24E+01	6.24E+01
ENSG00000157349	DDX19B	0.77	-1.31	-0.38	4.40E-04	-6.22E+01	6.22E+01
ENSG00000124541	RRP36	0.76	-1.32	-0.40	4.49E-04	-6.21E+01	6.21E+01
ENSG00000100084	HIRA	1.36	1.36	0.44	4.79E-04	6.20E+01	6.20E+01
ENSG00000111906	HDDC2	1.33	1.33	0.41	4.63E-04	6.19E+01	6.19E+01
ENSG00000160094	ZNF362	1.41	1.41	0.49	5.19E-04	6.19E+01	6.19E+01
ENSG00000100568	VTI1B	0.77	-1.31	-0.38	4.46E-04	-6.18E+01	6.18E+01
ENSG00000186104	CYP2R1	1.31	1.31	0.39	4.50E-04	6.16E+01	6.16E+01
ENSG00000162923	WDR26	1.32	1.32	0.40	4.58E-04	6.16E+01	6.16E+01
	XXyac-YRM2039.2	1.61	1.61	0.69	6.83E-04	6.16E+01	6.16E+01
ENSG00000006712	PAF1	0.76	-1.31	-0.39	4.55E-04	-6.15E+01	6.15E+01
ENSG00000267350	RP1-178F10.3	1.30	1.30	0.38	4.48E-04	6.14E+01	6.14E+01
ENSG00000153310	FAM49B	1.31	1.31	0.39	4.53E-04	6.14E+01	6.14E+01
ENSG00000149923	PPP4C	0.76	-1.32	-0.40	4.64E-04	-6.12E+01	6.12E+01
ENSG00000105063	PPP6R1	1.33	1.33	0.41	4.69E-04	6.12E+01	6.12E+01
ENSG00000115526	CHST10	0.77	-1.31	-0.38	4.57E-04	-6.11E+01	6.11E+01
ENSG00000123200	ZC3H13	1.43	1.43	0.52	5.50E-04	6.11E+01	6.11E+01
ENSG00000173960	UBXN2A	1.30	1.30	0.38	4.58E-04	6.09E+01	6.09E+01
ENSG00000058804	TMEM48	0.76	-1.31	-0.39	4.67E-04	-6.08E+01	6.08E+01
ENSG00000204177	BMS1P1	0.71	-1.41	-0.50	5.37E-04	-6.08E+01	6.08E+01
ENSG00000165417	GTF2A1	1.32	1.32	0.40	4.74E-04	6.08E+01	6.08E+01
ENSG00000127252	HRASLS	0.63	-1.60	-0.68	6.93E-04	-6.07E+01	6.07E+01
ENSG00000167110	GOLGA2	1.30	1.30	0.38	4.58E-04	6.07E+01	6.07E+01
ENSG00000180611	MB21D2	1.31	1.31	0.39	4.65E-04	6.07E+01	6.07E+01
ENSG00000141655	TNFRSF11A	0.71	-1.40	-0.49	5.35E-04	-6.06E+01	6.06E+01
ENSG00000135457	TFCP2	0.75	-1.34	-0.42	4.89E-04	-6.05E+01	6.05E+01
ENSG00000235109	ZNF323	1.31	1.31	0.39	4.71E-04	6.05E+01	6.05E+01
ENSG00000151470	C4orf33	1.42	1.42	0.50	5.48E-04	6.05E+01	6.05E+01
ENSG00000064932	SBNO2	1.35	1.35	0.43	4.99E-04	6.03E+01	6.03E+01
ENSG00000100348	TXN2	0.71	-1.41	-0.49	5.44E-04	-6.03E+01	6.03E+01
ENSG00000163249	CCNYL1	0.68	-1.47	-0.56	5.95E-04	-6.03E+01	6.03E+01
ENSG00000102359	SRPX2	1.32	1.32	0.40	4.80E-04	6.02E+01	6.02E+01
ENSG00000215417	MIR17HG	0.73	-1.37	-0.45	5.15E-04	-6.02E+01	6.02E+01
ENSG00000248866	USP46-AS1	0.60	-1.66	-0.73	7.66E-04	-6.01E+01	6.01E+01
ENSG00000170906	NDUFA3	0.76	-1.32	-0.40	4.85E-04	-6.00E+01	6.00E+01
ENSG00000171940	ZNF217	1.30	1.30	0.38	4.69E-04	6.00E+01	6.00E+01
ENSG00000085563	ABCB1	0.65	-1.53	-0.62	6.52E-04	-6.00E+01	6.00E+01
ENSG00000038532	CLEC16A	1.30	1.30	0.37	4.69E-04	5.99E+01	5.99E+01
ENSG00000102978	POLR2C	0.76	-1.32	-0.40	4.84E-04	-5.98E+01	5.98E+01
ENSG00000214960	ISPD	0.61	-1.65	-0.72	7.67E-04	-5.96E+01	5.96E+01
ENSG00000141564	RPTOR	1.41	1.41	0.50	5.62E-04	5.96E+01	5.96E+01
ENSG00000068024	HDAC4	1.33	1.33	0.42	5.02E-04	5.96E+01	5.96E+01
	RP11-27I1.2	0.75	-1.33	-0.41	4.99E-04	-5.95E+01	5.95E+01
ENSG00000189319	FAM53B	1.29	1.29	0.37	4.69E-04	5.95E+01	5.95E+01
ENSG00000106608	URGCP	1.30	1.30	0.38	4.79E-04	5.94E+01	5.94E+01
ENSG00000169851	PCDH7	0.76	-1.31	-0.39	4.86E-04	-5.94E+01	5.94E+01
ENSG00000185252	ZNF74	1.30	1.30	0.38	4.81E-04	5.94E+01	5.94E+01
ENSG00000132003	ZSWIM4	1.37	1.37	0.46	5.38E-04	5.92E+01	5.92E+01

ENSG00000148655	C10orf11	0.70	-1.43	-0.52	5.93E-04	-5.89E+01	5.89E+01
ENSG00000069667	RORA	1.45	1.45	0.54	6.08E-04	5.89E+01	5.89E+01
ENSG00000145220	LYAR	0.77	-1.30	-0.38	4.89E-04	-5.89E+01	5.89E+01
ENSG00000182247	UBE2E2	0.76	-1.32	-0.40	5.05E-04	-5.86E+01	5.86E+01
ENSG00000107643	MAPK8	0.77	-1.30	-0.38	4.96E-04	-5.86E+01	5.86E+01
ENSG00000182871	COL18A1	1.44	1.44	0.52	6.03E-04	5.85E+01	5.85E+01
ENSG00000168653	NDUFS5	0.74	-1.35	-0.44	5.35E-04	-5.84E+01	5.84E+01
ENSG00000145725	PPIP5K2	1.46	1.46	0.54	6.23E-04	5.84E+01	5.84E+01
ENSG00000185989	RASA3	1.32	1.32	0.40	5.10E-04	5.83E+01	5.83E+01
ENSG00000175220	ARHGAP1	1.35	1.35	0.43	5.37E-04	5.81E+01	5.81E+01
ENSG00000187266	EPOR	1.42	1.42	0.51	6.01E-04	5.80E+01	5.80E+01
	RP11-117P22.1	1.31	1.31	0.39	5.15E-04	5.77E+01	5.77E+01
ENSG00000103599	IQCH	1.60	1.60	0.68	7.69E-04	5.76E+01	5.76E+01
ENSG00000085276	MECOM	1.37	1.37	0.45	5.66E-04	5.76E+01	5.76E+01
ENSG00000213967	ZNF726	0.68	-1.46	-0.55	6.48E-04	-5.75E+01	5.75E+01
ENSG00000067082	KLF6	1.37	1.37	0.46	5.71E-04	5.75E+01	5.75E+01
ENSG00000115183	TANC1	1.37	1.37	0.45	5.68E-04	5.75E+01	5.75E+01
ENSG00000006118	TMEM132A	1.31	1.31	0.39	5.18E-04	5.74E+01	5.74E+01
ENSG00000008256	CYTH3	1.33	1.33	0.41	5.38E-04	5.73E+01	5.73E+01
ENSG00000173207	CKS1B	0.77	-1.30	-0.38	5.18E-04	-5.71E+01	5.71E+01
ENSG00000071553	ATP6AP1	1.31	1.31	0.38	5.22E-04	5.71E+01	5.71E+01
ENSG00000185920	PTCH1	0.68	-1.48	-0.57	6.75E-04	-5.69E+01	5.69E+01
ENSG00000171792	RHNO1	0.76	-1.31	-0.39	5.33E-04	-5.68E+01	5.68E+01
ENSG00000126457	PRMT1	0.76	-1.32	-0.40	5.46E-04	-5.66E+01	5.66E+01
ENSG00000160131	VMA21	0.76	-1.32	-0.40	5.45E-04	-5.65E+01	5.65E+01
ENSG00000118181	RPS25	0.72	-1.38	-0.47	6.00E-04	-5.64E+01	5.64E+01
ENSG00000134255	CEPT1	0.77	-1.31	-0.38	5.36E-04	-5.64E+01	5.64E+01
ENSG00000166377	ATP9B	0.77	-1.30	-0.38	5.31E-04	-5.63E+01	5.63E+01
ENSG00000214654	RP11-27I1.4	1.61	1.61	0.69	8.16E-04	5.63E+01	5.63E+01
ENSG00000180884	ZNF792	1.30	1.30	0.37	5.30E-04	5.63E+01	5.63E+01
ENSG00000135338	LCA5	1.46	1.46	0.55	6.78E-04	5.62E+01	5.62E+01
ENSG00000222365	SNORD12B	0.58	-1.72	-0.78	9.38E-04	-5.60E+01	5.60E+01
ENSG00000134758	RNF138	0.77	-1.29	-0.37	5.36E-04	-5.59E+01	5.59E+01
ENSG00000177954	RPS27	0.73	-1.36	-0.45	5.99E-04	-5.57E+01	5.57E+01
ENSG00000154814	OXNAD1	0.76	-1.31	-0.39	5.55E-04	-5.57E+01	5.57E+01
ENSG00000172244	C5orf34	0.72	-1.39	-0.47	6.21E-04	-5.56E+01	5.56E+01
ENSG00000177888	ZBTB41	1.54	1.54	0.62	7.69E-04	5.56E+01	5.56E+01
ENSG00000189266	PNRC2	0.76	-1.31	-0.39	5.58E-04	-5.54E+01	5.54E+01
ENSG00000175061	C17orf76-AS1	0.76	-1.32	-0.40	5.66E-04	-5.53E+01	5.53E+01
ENSG00000243811	APOBEC3D	1.67	1.67	0.74	9.15E-04	5.53E+01	5.53E+01
ENSG00000112992	NNT	0.76	-1.31	-0.39	5.59E-04	-5.53E+01	5.53E+01
ENSG00000233757	AC092835.2	1.46	1.46	0.55	7.00E-04	5.52E+01	5.52E+01
ENSG00000066651	TRMT11	0.73	-1.37	-0.46	6.18E-04	-5.52E+01	5.52E+01
ENSG00000108465	CDK5RAP3	0.78	-1.29	-0.36	5.48E-04	-5.50E+01	5.50E+01
ENSG00000112514	CUTA	0.77	-1.30	-0.37	5.57E-04	-5.49E+01	5.49E+01
ENSG00000165115	KIF27	1.52	1.52	0.60	7.63E-04	5.49E+01	5.49E+01
ENSG00000163138	PACRGL	0.77	-1.31	-0.39	5.68E-04	-5.48E+01	5.48E+01
ENSG00000151292	CSNK1G3	1.31	1.31	0.39	5.73E-04	5.48E+01	5.48E+01
ENSG00000075240	GRAMD4	1.34	1.34	0.42	5.96E-04	5.48E+01	5.48E+01
ENSG00000131475	VPS25	1.32	1.32	0.40	5.82E-04	5.45E+01	5.45E+01
ENSG00000023909	GCLM	0.75	-1.33	-0.41	5.97E-04	-5.45E+01	5.45E+01
ENSG00000167081	PBX3	1.30	1.30	0.38	5.72E-04	5.43E+01	5.43E+01
ENSG00000140396	NCOA2	1.29	1.29	0.36	5.61E-04	5.43E+01	5.43E+01
ENSG00000213693	SEC14L1P1	1.61	1.61	0.68	8.81E-04	5.42E+01	5.42E+01
ENSG00000160959	LRRC14	0.77	-1.30	-0.38	5.79E-04	-5.41E+01	5.41E+01
ENSG00000123643	SLC36A1	1.35	1.35	0.44	6.25E-04	5.41E+01	5.41E+01
ENSG00000177463	NR2C2	1.34	1.34	0.42	6.09E-04	5.41E+01	5.41E+01
ENSG00000143420	ENSA	0.76	-1.31	-0.39	5.86E-04	-5.41E+01	5.41E+01
ENSG00000104131	EIF3J	0.75	-1.34	-0.42	6.15E-04	-5.40E+01	5.40E+01
ENSG00000126464	PRR12	1.40	1.40	0.49	6.79E-04	5.37E+01	5.37E+01
ENSG00000103047	TMCO7	0.75	-1.33	-0.41	6.11E-04	-5.37E+01	5.37E+01
ENSG00000166532	RIMKLB	0.76	-1.31	-0.39	6.01E-04	-5.35E+01	5.35E+01
ENSG00000172878	METAP1D	0.72	-1.39	-0.47	6.72E-04	-5.34E+01	5.34E+01
ENSG00000105011	ASF1B	0.75	-1.34	-0.42	6.27E-04	-5.34E+01	5.34E+01
ENSG00000151148	UBE3B	1.33	1.33	0.41	6.23E-04	5.33E+01	5.33E+01
ENSG00000141956	PRDM15	1.34	1.34	0.42	6.34E-04	5.33E+01	5.33E+01
ENSG00000198924	DCLRE1A	1.38	1.38	0.47	6.72E-04	5.33E+01	5.33E+01
ENSG00000132326	PER2	0.65	-1.53	-0.61	8.26E-04	-5.32E+01	5.32E+01
ENSG00000121864	ZNF639	0.78	-1.29	-0.37	5.86E-04	-5.32E+01	5.32E+01
ENSG00000139436	GIT2	1.30	1.30	0.38	5.94E-04	5.32E+01	5.32E+01
ENSG00000257103	LSM14A	0.76	-1.32	-0.40	6.17E-04	-5.32E+01	5.32E+01
ENSG00000152642	GPD1L	0.78	-1.29	-0.37	5.89E-04	-5.31E+01	5.31E+01
ENSG00000136478	TEX2	0.76	-1.31	-0.39	6.10E-04	-5.31E+01	5.31E+01
ENSG00000141458	NPC1	1.31	1.31	0.39	6.09E-04	5.30E+01	5.30E+01
ENSG00000123728	RAP2C	0.76	-1.32	-0.40	6.22E-04	-5.29E+01	5.29E+01
ENSG00000050130	JKAMP	1.31	1.31	0.39	6.14E-04	5.29E+01	5.29E+01
ENSG00000134899	ERCC5	1.30	1.30	0.38	6.09E-04	5.29E+01	5.29E+01
ENSG00000144674	GOLGA4	1.39	1.39	0.47	6.94E-04	5.28E+01	5.28E+01
ENSG00000205765	C5orf51	1.30	1.30	0.38	6.07E-04	5.27E+01	5.27E+01

ENSG00000160124	CCDC58	0.76	-1.32	-0.40	6.27E-04	-5.27E+01	5.27E+01
ENSG00000275126	HIST1H4L	0.74	-1.34	-0.43	6.50E-04	-5.27E+01	5.27E+01
ENSG00000163684	RPP14	0.78	-1.29	-0.36	5.97E-04	-5.27E+01	5.27E+01
ENSG00000167965	MLST8	0.65	-1.54	-0.62	8.60E-04	-5.25E+01	5.25E+01
ENSG00000143499	SMYD2	0.78	-1.29	-0.36	6.00E-04	-5.25E+01	5.25E+01
ENSG00000196141	SPATS2L	1.32	1.32	0.40	6.30E-04	5.25E+01	5.25E+01
ENSG00000107829	FBXW4	1.35	1.35	0.43	6.64E-04	5.24E+01	5.24E+01
ENSG00000171861	RNMTL1	0.77	-1.30	-0.38	6.16E-04	-5.23E+01	5.23E+01
ENSG00000092098	RNF31	0.76	-1.32	-0.40	6.34E-04	-5.23E+01	5.23E+01
ENSG00000120942	UBIAD1	1.31	1.31	0.39	6.32E-04	5.22E+01	5.22E+01
ENSG00000121957	GPSM2	0.76	-1.31	-0.39	6.29E-04	-5.22E+01	5.22E+01
ENSG00000140945	CDH13	1.32	1.32	0.40	6.45E-04	5.21E+01	5.21E+01
ENSG00000181004	BBS12	1.39	1.39	0.47	7.11E-04	5.21E+01	5.21E+01
	AC004383.4	0.71	-1.40	-0.49	7.25E-04	-5.21E+01	5.21E+01
ENSG00000163681	SLMAP	1.30	1.30	0.38	6.28E-04	5.19E+01	5.19E+01
ENSG00000170222	ADPRM	0.73	-1.37	-0.46	7.04E-04	-5.18E+01	5.18E+01
ENSG00000196550	FAM72A	1.63	1.63	0.71	9.97E-04	5.17E+01	5.17E+01
ENSG00000079691	LRRC16A	1.30	1.30	0.38	6.30E-04	5.17E+01	5.17E+01
ENSG00000183527	PSMG1	0.77	-1.31	-0.39	6.42E-04	-5.16E+01	5.16E+01
ENSG00000138442	WDR12	0.78	-1.28	-0.36	6.23E-04	-5.14E+01	5.14E+01
ENSG00000100266	PACSIN2	0.76	-1.32	-0.40	6.64E-04	-5.14E+01	5.14E+01
ENSG00000197622	CDC42SE1	1.31	1.31	0.39	6.47E-04	5.13E+01	5.13E+01
ENSG00000233404	FLJ20373	1.30	1.30	0.38	6.46E-04	5.12E+01	5.12E+01
ENSG00000042088	TDP1	0.77	-1.30	-0.38	6.48E-04	-5.11E+01	5.11E+01
ENSG00000102390	CXorf26	0.73	-1.37	-0.45	7.21E-04	-5.10E+01	5.10E+01
ENSG00000169919	GUSB	0.78	-1.28	-0.36	6.35E-04	-5.09E+01	5.09E+01
ENSG00000227057	WDR46	0.77	-1.29	-0.37	6.44E-04	-5.08E+01	5.08E+01
ENSG00000141524	TMC6	1.28	1.28	0.36	6.40E-04	5.07E+01	5.07E+01
ENSG00000115738	ID2	0.63	-1.60	-0.68	9.92E-04	-5.07E+01	5.07E+01
ENSG00000106366	SERPINE1	1.35	1.35	0.43	7.04E-04	5.07E+01	5.07E+01
ENSG00000068097	HEATR6	0.75	-1.33	-0.41	6.84E-04	-5.07E+01	5.07E+01
ENSG00000175334	BANF1	0.71	-1.42	-0.50	7.85E-04	-5.06E+01	5.06E+01
ENSG00000181458	TMEM45A	1.34	1.34	0.43	7.05E-04	5.06E+01	5.06E+01
ENSG00000231500	RPS18	0.74	-1.35	-0.43	7.11E-04	-5.05E+01	5.05E+01
ENSG00000099940	SNAP29	1.41	1.41	0.50	7.80E-04	5.05E+01	5.05E+01
ENSG00000241404	EGFL8	0.65	-1.54	-0.63	9.40E-04	-5.04E+01	5.04E+01
ENSG00000049618	ARID1B	1.29	1.29	0.37	6.62E-04	5.03E+01	5.03E+01
ENSG00000108861	DUSP3	1.30	1.30	0.37	6.66E-04	5.02E+01	5.02E+01
ENSG00000139636	LMBR1L	1.32	1.32	0.40	6.93E-04	5.02E+01	5.02E+01
ENSG00000137700	SLC37A4	0.77	-1.29	-0.37	6.68E-04	-5.01E+01	5.01E+01
ENSG00000105968	H2AFV	0.77	-1.31	-0.39	6.82E-04	-5.00E+01	5.00E+01
ENSG00000182993	C12orf60	1.56	1.56	0.64	9.78E-04	5.00E+01	5.00E+01
ENSG00000113070	HBEGF	1.30	1.30	0.38	6.84E-04	4.99E+01	4.99E+01
ENSG00000154133	ROBO4	1.37	1.37	0.45	7.52E-04	4.99E+01	4.99E+01
ENSG00000230551	CTB-89H12.4	0.77	-1.30	-0.38	6.83E-04	-4.98E+01	4.98E+01
ENSG00000146833	TRIM4	1.29	1.29	0.36	6.69E-04	4.97E+01	4.97E+01
ENSG00000087494	PTHLH	0.64	-1.56	-0.65	9.89E-04	-4.97E+01	4.97E+01
ENSG00000138190	EXOC6	1.36	1.36	0.45	7.50E-04	4.97E+01	4.97E+01
ENSG00000137404	NRM	0.74	-1.36	-0.44	7.49E-04	-4.97E+01	4.97E+01
ENSG00000105404	RABAC1	1.31	1.31	0.39	7.01E-04	4.96E+01	4.96E+01
ENSG00000172216	CEBPB	0.73	-1.37	-0.46	7.66E-04	-4.96E+01	4.96E+01
ENSG00000130635	COL5A1	1.39	1.39	0.47	7.85E-04	4.95E+01	4.95E+01
ENSG00000155846	PPARGC1B	0.65	-1.53	-0.61	9.57E-04	-4.94E+01	4.94E+01
ENSG00000159840	ZYX	1.44	1.44	0.53	8.48E-04	4.94E+01	4.94E+01
ENSG00000067191	CACNB1	0.67	-1.50	-0.59	9.27E-04	-4.94E+01	4.94E+01
ENSG00000106628	POLD2	0.76	-1.32	-0.40	7.20E-04	-4.93E+01	4.93E+01
ENSG00000146414	SHPRH	1.32	1.32	0.40	7.17E-04	4.93E+01	4.93E+01
ENSG00000100554	ATP6V1D	1.30	1.30	0.38	6.98E-04	4.91E+01	4.91E+01
ENSG00000124784	RIOK1	0.78	-1.28	-0.36	6.85E-04	-4.90E+01	4.90E+01
ENSG00000159403	C1R	1.61	1.61	0.69	1.08E-03	4.90E+01	4.90E+01
ENSG00000081181	ARG2	1.29	1.29	0.37	6.94E-04	4.89E+01	4.89E+01
ENSG00000171634	BPTF	1.36	1.36	0.45	7.74E-04	4.89E+01	4.89E+01
ENSG00000182199	SHMT2	0.76	-1.31	-0.39	7.22E-04	-4.88E+01	4.88E+01
ENSG00000180228	PRKRA	0.78	-1.29	-0.36	6.96E-04	-4.88E+01	4.88E+01
ENSG00000182700	IGIP	1.86	1.86	0.90	1.46E-03	4.87E+01	4.87E+01
ENSG00000131711	MAP1B	1.33	1.33	0.41	7.51E-04	4.86E+01	4.86E+01
ENSG00000253159	PCDHGA12	1.56	1.56	0.64	1.03E-03	4.85E+01	4.85E+01
ENSG00000182180	MRPS16	0.77	-1.30	-0.38	7.17E-04	-4.84E+01	4.84E+01
ENSG00000130164	LDLR	1.36	1.36	0.45	7.93E-04	4.84E+01	4.84E+01
ENSG00000104853	CLPTM1	1.46	1.46	0.55	9.14E-04	4.84E+01	4.84E+01
ENSG00000198937	CCDC167	0.75	-1.34	-0.42	7.69E-04	-4.83E+01	4.83E+01
ENSG00000120685	PROSER1	1.30	1.30	0.38	7.28E-04	4.82E+01	4.82E+01
ENSG00000186187	ZNRF1	1.29	1.29	0.37	7.17E-04	4.82E+01	4.82E+01
ENSG00000149577	SIDT2	0.71	-1.41	-0.50	8.60E-04	-4.82E+01	4.82E+01
ENSG00000070371	CLTCL1	0.75	-1.34	-0.42	7.78E-04	-4.81E+01	4.81E+01
ENSG00000197747	S100A10	1.32	1.32	0.40	7.58E-04	4.80E+01	4.80E+01
ENSG00000204622	HLA-J	1.49	1.49	0.58	9.82E-04	4.77E+01	4.77E+01
ENSG00000129480	DTD2	0.77	-1.29	-0.37	7.35E-04	-4.76E+01	4.76E+01
ENSG00000245571	AP001258.4	0.66	-1.52	-0.60	1.02E-03	-4.76E+01	4.76E+01

ENSG00000141556	TBCD	1.29	1.29	0.37	7.32E-04	4.76E+01	4.76E+01
ENSG00000168209	DDIT4	1.29	1.29	0.37	7.34E-04	4.75E+01	4.75E+01
ENSG00000187824	TMEM220	0.68	-1.48	-0.56	9.68E-04	-4.75E+01	4.75E+01
ENSG00000116514	RNF19B	1.30	1.30	0.37	7.43E-04	4.75E+01	4.75E+01
ENSG00000133789	SWAP70	1.32	1.32	0.40	7.68E-04	4.75E+01	4.75E+01
ENSG00000073050	XRCC1	0.77	-1.30	-0.38	7.52E-04	-4.75E+01	4.75E+01
ENSG00000132824	SERINC3	1.31	1.31	0.39	7.62E-04	4.74E+01	4.74E+01
ENSG00000151914	DST	1.42	1.42	0.51	9.01E-04	4.74E+01	4.74E+01
ENSG00000088298	EDEM2	1.29	1.29	0.37	7.41E-04	4.73E+01	4.73E+01
ENSG00000189079	ARID2	1.33	1.33	0.41	7.90E-04	4.73E+01	4.73E+01
ENSG00000184436	THAP7	0.76	-1.31	-0.39	7.77E-04	-4.72E+01	4.72E+01
ENSG00000099290	FAM21A	1.37	1.37	0.45	8.45E-04	4.70E+01	4.70E+01
ENSG00000126216	TUBGCP3	0.78	-1.28	-0.36	7.47E-04	-4.70E+01	4.70E+01
ENSG00000187607	ZNF286A	1.28	1.28	0.36	7.46E-04	4.70E+01	4.70E+01
ENSG00000176731	C8orf59	0.75	-1.34	-0.42	8.21E-04	-4.66E+01	4.66E+01
ENSG00000166908	PIP4K2C	1.33	1.33	0.42	8.20E-04	4.66E+01	4.66E+01
	AC018633.4	1.67	1.67	0.74	1.28E-03	4.66E+01	4.66E+01
ENSG00000122966	CIT	0.77	-1.30	-0.38	7.84E-04	-4.65E+01	4.65E+01
ENSG00000100387	RBX1	0.73	-1.38	-0.46	8.78E-04	-4.65E+01	4.65E+01
ENSG00000185163	DDX51	0.77	-1.30	-0.38	7.89E-04	-4.64E+01	4.64E+01
ENSG00000166349	RAG1	1.55	1.55	0.64	1.12E-03	4.64E+01	4.64E+01
ENSG00000144504	ANKMY1	1.37	1.37	0.45	8.69E-04	4.64E+01	4.64E+01
ENSG00000133895	MEN1	1.36	1.36	0.45	8.65E-04	4.64E+01	4.64E+01
ENSG00000139684	ESD	0.76	-1.31	-0.39	8.04E-04	-4.62E+01	4.62E+01
ENSG00000165359	DDX26B	0.73	-1.37	-0.45	8.76E-04	-4.62E+01	4.62E+01
ENSG00000096654	ZNF184	1.36	1.36	0.44	8.68E-04	4.61E+01	4.61E+01
ENSG00000165029	ABCA1	0.77	-1.30	-0.37	7.96E-04	-4.60E+01	4.60E+01
ENSG00000165943	MOAP1	0.77	-1.30	-0.38	8.01E-04	-4.59E+01	4.59E+01
	RP11-222A11.1	0.60	-1.65	-0.73	1.30E-03	-4.58E+01	4.58E+01
ENSG00000130707	ASS1	1.58	1.58	0.66	1.19E-03	4.58E+01	4.58E+01
ENSG00000144134	RABL2A	1.50	1.50	0.58	1.08E-03	4.56E+01	4.56E+01
ENSG00000205208	C4orf46	0.77	-1.29	-0.37	8.04E-04	-4.55E+01	4.55E+01
ENSG00000204264	PSMB8	0.78	-1.28	-0.36	7.93E-04	-4.55E+01	4.55E+01
ENSG00000213689	TREX1	0.72	-1.40	-0.48	9.45E-04	-4.55E+01	4.55E+01
ENSG00000163104	SMARCAD1	1.40	1.40	0.49	9.56E-04	4.54E+01	4.54E+01
ENSG00000205683	DPF3	1.33	1.33	0.41	8.58E-04	4.52E+01	4.52E+01
ENSG00000071082	RPL31	0.74	-1.36	-0.44	9.05E-04	-4.52E+01	4.52E+01
ENSG00000169193	CCDC126	1.36	1.36	0.45	9.12E-04	4.52E+01	4.52E+01
ENSG00000132768	DPH2	0.78	-1.29	-0.36	8.11E-04	-4.52E+01	4.52E+01
ENSG00000177191	B3GNT8	0.60	-1.66	-0.73	1.35E-03	-4.52E+01	4.52E+01
ENSG00000126950	TMEM35	1.33	1.33	0.41	8.71E-04	4.51E+01	4.51E+01
ENSG00000151687	ANKAR	0.66	-1.50	-0.59	1.11E-03	-4.51E+01	4.51E+01
ENSG00000113761	ZNF346	0.77	-1.29	-0.37	8.21E-04	-4.51E+01	4.51E+01
ENSG00000204381	LAYN	1.67	1.67	0.74	1.39E-03	4.50E+01	4.50E+01
ENSG00000119402	FBXW2	0.78	-1.28	-0.35	8.06E-04	-4.50E+01	4.50E+01
ENSG00000006062	MAP3K14	0.73	-1.38	-0.46	9.41E-04	-4.50E+01	4.50E+01
ENSG00000177917	ARL6IP6	0.77	-1.30	-0.38	8.41E-04	-4.50E+01	4.50E+01
ENSG00000196172	ZNF681	0.63	-1.59	-0.67	1.25E-03	-4.49E+01	4.49E+01
ENSG00000145332	KLHL8	0.78	-1.28	-0.36	8.22E-04	-4.48E+01	4.48E+01
ENSG00000165516	KLHDC2	0.78	-1.28	-0.36	8.19E-04	-4.47E+01	4.47E+01
ENSG00000156802	ATAD2	0.72	-1.39	-0.48	9.73E-04	-4.47E+01	4.47E+01
ENSG00000132321	IQCA1	0.71	-1.41	-0.49	9.92E-04	-4.46E+01	4.46E+01
	AD000090.2	1.37	1.37	0.45	9.38E-04	4.46E+01	4.46E+01
ENSG00000173145	NOC3L	0.73	-1.36	-0.45	9.36E-04	-4.45E+01	4.45E+01
ENSG00000102393	GLA	0.78	-1.29	-0.37	8.41E-04	-4.44E+01	4.44E+01
ENSG00000174227	PIGG	1.29	1.29	0.37	8.45E-04	4.44E+01	4.44E+01
ENSG00000178252	WDR6	0.77	-1.30	-0.37	8.57E-04	-4.43E+01	4.43E+01
ENSG00000168214	RBPJ	1.31	1.31	0.39	8.76E-04	4.43E+01	4.43E+01
ENSG00000077238	IL4R	1.28	1.28	0.36	8.45E-04	4.40E+01	4.40E+01
ENSG00000174010	KLHL15	0.75	-1.33	-0.41	9.17E-04	-4.40E+01	4.40E+01
ENSG00000112983	BRD8	0.76	-1.32	-0.40	9.05E-04	-4.40E+01	4.40E+01
ENSG00000168566	SNRNP48	0.78	-1.28	-0.35	8.44E-04	-4.39E+01	4.39E+01
ENSG00000181045	SLC26A11	1.33	1.33	0.41	9.16E-04	4.39E+01	4.39E+01
ENSG00000134508	CABLES1	1.34	1.34	0.42	9.33E-04	4.38E+01	4.38E+01
ENSG00000111144	LTA4H	0.77	-1.30	-0.38	8.79E-04	-4.38E+01	4.38E+01
ENSG00000259953	RP11-4O1.2	0.64	-1.55	-0.63	1.26E-03	-4.38E+01	4.38E+01
ENSG00000092439	TRPM7	1.31	1.31	0.39	9.01E-04	4.38E+01	4.38E+01
ENSG00000169684	CHRNA5	0.74	-1.35	-0.43	9.53E-04	-4.37E+01	4.37E+01
ENSG00000140691	ARMC5	1.31	1.31	0.39	9.01E-04	4.37E+01	4.37E+01
ENSG00000134954	ETS1	1.34	1.34	0.43	9.49E-04	4.36E+01	4.36E+01
ENSG00000171467	ZNF318	1.53	1.53	0.62	1.24E-03	4.36E+01	4.36E+01
ENSG00000109654	TRIM2	1.30	1.30	0.38	8.95E-04	4.35E+01	4.35E+01
ENSG00000104081	BMF	1.42	1.42	0.50	1.06E-03	4.35E+01	4.35E+01
ENSG00000196110	ZNF699	1.39	1.39	0.47	1.02E-03	4.35E+01	4.35E+01
ENSG00000164258	NDUFS4	0.76	-1.31	-0.39	9.15E-04	-4.33E+01	4.33E+01
ENSG00000170871	KIAA0232	1.29	1.29	0.36	8.86E-04	4.32E+01	4.32E+01
ENSG00000180263	FGD6	1.28	1.28	0.36	8.82E-04	4.32E+01	4.32E+01
ENSG00000174007	CEP19	1.45	1.45	0.54	1.13E-03	4.32E+01	4.32E+01
ENSG00000171793	CTPS1	0.77	-1.30	-0.38	9.04E-04	-4.32E+01	4.32E+01

ENSG00000168792	ABHD15	1.35	1.35	0.43	9.80E-04	4.31E+01	4.31E+01
ENSG00000251095	RP11-115D19.1	0.64	-1.57	-0.65	1.33E-03	-4.31E+01	4.31E+01
ENSG00000214401	KANSL1-AS1	1.54	1.54	0.62	1.27E-03	4.31E+01	4.31E+01
ENSG00000087095	NLK	1.28	1.28	0.36	8.87E-04	4.31E+01	4.31E+01
ENSG00000105202	FBL	0.67	-1.50	-0.59	1.21E-03	-4.31E+01	4.31E+01
ENSG00000177239	MAN1B1	1.32	1.32	0.40	9.33E-04	4.31E+01	4.31E+01
ENSG00000041988	THAP3	0.74	-1.35	-0.43	9.79E-04	-4.30E+01	4.30E+01
ENSG00000177646	ACAD9	0.77	-1.30	-0.38	9.11E-04	-4.30E+01	4.30E+01
ENSG00000182621	PLCB1	1.34	1.34	0.43	9.77E-04	4.30E+01	4.30E+01
ENSG00000184986	TMEM121	0.61	-1.65	-0.72	1.48E-03	-4.29E+01	4.29E+01
ENSG00000114520	SNX4	1.31	1.31	0.39	9.33E-04	4.28E+01	4.28E+01
ENSG00000076351	SLC46A1	1.38	1.38	0.47	1.04E-03	4.28E+01	4.28E+01
ENSG00000273749	CYFIP1	1.30	1.30	0.38	9.23E-04	4.28E+01	4.28E+01
ENSG00000239407	LL0XNC01-237H1.2	1.54	1.54	0.62	1.29E-03	4.28E+01	4.28E+01
ENSG00000012817	KDM5D	0.68	-1.47	-0.56	1.19E-03	-4.27E+01	4.27E+01
ENSG00000106025	TSPAN12	0.78	-1.28	-0.36	9.08E-04	-4.25E+01	4.25E+01
ENSG00000102595	UGGT2	0.74	-1.35	-0.43	1.00E-03	-4.25E+01	4.25E+01
ENSG00000236618	PITPNA-AS1	0.71	-1.41	-0.49	1.10E-03	-4.25E+01	4.25E+01
ENSG00000164751	PEX2	0.78	-1.28	-0.35	9.09E-04	-4.24E+01	4.24E+01
ENSG00000173517	PEAK1	1.30	1.30	0.38	9.44E-04	4.23E+01	4.23E+01
ENSG00000169641	LUZP1	1.29	1.29	0.37	9.36E-04	4.23E+01	4.23E+01
ENSG00000108179	PPIF	0.77	-1.30	-0.38	9.45E-04	-4.23E+01	4.23E+01
ENSG00000109118	PHF12	1.31	1.31	0.39	9.61E-04	4.23E+01	4.23E+01
ENSG00000140682	TGFB1I1	1.27	1.27	0.35	9.10E-04	4.22E+01	4.22E+01
ENSG00000105829	BET1	0.78	-1.28	-0.36	9.25E-04	-4.22E+01	4.22E+01
ENSG00000104866	PPP1R37	0.78	-1.29	-0.36	9.28E-04	-4.22E+01	4.22E+01
ENSG00000206149	HERC2P9	0.73	-1.37	-0.46	1.06E-03	-4.22E+01	4.22E+01
ENSG00000171109	MFN1	0.77	-1.30	-0.38	9.54E-04	-4.22E+01	4.22E+01
ENSG00000170088	TMEM192	1.28	1.28	0.35	9.19E-04	4.21E+01	4.21E+01
ENSG00000101935	AMMECR1	0.77	-1.30	-0.38	9.55E-04	-4.20E+01	4.20E+01
ENSG00000104731	KLHDC4	0.78	-1.29	-0.36	9.39E-04	-4.20E+01	4.20E+01
ENSG00000275004	ZNF280B	0.71	-1.41	-0.49	1.12E-03	-4.20E+01	4.20E+01
ENSG00000168036	CTNNB1	1.30	1.30	0.38	9.64E-04	4.20E+01	4.20E+01
ENSG00000267160	RP11-1072C15.4	1.65	1.65	0.72	1.55E-03	4.20E+01	4.20E+01
ENSG00000068650	ATP11A	1.41	1.41	0.50	1.14E-03	4.19E+01	4.19E+01
ENSG00000140948	ZCCHC14	1.32	1.32	0.40	9.86E-04	4.19E+01	4.19E+01
ENSG00000179041	RRS1	0.75	-1.34	-0.42	1.02E-03	-4.19E+01	4.19E+01
ENSG00000128606	LRRC17	0.77	-1.31	-0.38	9.73E-04	-4.19E+01	4.19E+01
ENSG00000132286	FXC1	0.78	-1.29	-0.37	9.52E-04	-4.18E+01	4.18E+01
ENSG00000169291	SHE	1.30	1.30	0.38	9.72E-04	4.17E+01	4.17E+01
ENSG00000103365	GGA2	0.78	-1.28	-0.35	9.37E-04	-4.17E+01	4.17E+01
ENSG00000154305	MIA3	1.29	1.29	0.37	9.64E-04	4.17E+01	4.17E+01
ENSG00000164073	MFSD8	1.31	1.31	0.39	9.88E-04	4.16E+01	4.16E+01
ENSG00000167182	SP2	1.62	1.62	0.69	1.51E-03	4.16E+01	4.16E+01
ENSG00000108107	RPL28	0.69	-1.44	-0.53	1.21E-03	-4.15E+01	4.15E+01
ENSG00000178950	GAK	1.30	1.30	0.38	9.83E-04	4.15E+01	4.15E+01
ENSG00000143149	ALDH9A1	0.78	-1.28	-0.36	9.58E-04	-4.14E+01	4.14E+01
ENSG00000116584	ARHGEF2	1.32	1.32	0.40	1.02E-03	4.14E+01	4.14E+01
ENSG00000231007	CDC20P1	0.57	-1.74	-0.80	1.77E-03	-4.13E+01	4.13E+01
ENSG00000147439	BIN3	0.78	-1.29	-0.37	9.72E-04	-4.13E+01	4.13E+01
ENSG00000179240	RP11-111M22.2	1.58	1.58	0.66	1.46E-03	4.13E+01	4.13E+01
ENSG00000110697	PITPNM1	0.77	-1.30	-0.38	9.94E-04	-4.12E+01	4.12E+01
ENSG00000068001	HYAL2	1.30	1.30	0.37	9.93E-04	4.11E+01	4.11E+01
ENSG00000120519	SLC10A7	0.76	-1.32	-0.40	1.04E-03	-4.11E+01	4.11E+01
ENSG00000234431	AC007283.5	1.37	1.37	0.45	1.11E-03	4.11E+01	4.11E+01
ENSG00000117984	CTSD	1.33	1.33	0.41	1.05E-03	4.11E+01	4.11E+01
ENSG00000067715	SYT1	0.76	-1.32	-0.40	1.04E-03	-4.11E+01	4.11E+01
ENSG00000169594	BNC1	1.29	1.29	0.37	9.90E-04	4.10E+01	4.10E+01
ENSG00000213762	ZNF134	1.32	1.32	0.40	1.03E-03	4.10E+01	4.10E+01
ENSG00000213988	ZNF90	0.64	-1.57	-0.65	1.46E-03	-4.10E+01	4.10E+01
ENSG00000176994	SMCR8	1.29	1.29	0.37	9.99E-04	4.09E+01	4.09E+01
ENSG00000183032	SLC25A21	0.65	-1.55	-0.63	1.43E-03	-4.09E+01	4.09E+01
ENSG00000173933	RBM4	0.73	-1.37	-0.46	1.13E-03	-4.08E+01	4.08E+01
ENSG00000124549	BTN2A3P	0.73	-1.36	-0.45	1.11E-03	-4.08E+01	4.08E+01
ENSG00000109618	SEPSECS	1.30	1.30	0.38	1.02E-03	4.07E+01	4.07E+01
ENSG00000152582	SPEF2	0.60	-1.66	-0.73	1.66E-03	-4.07E+01	4.07E+01
ENSG00000103351	CLUAP1	0.77	-1.29	-0.37	1.01E-03	-4.07E+01	4.07E+01
ENSG00000138303	ASCC1	1.28	1.28	0.35	9.88E-04	4.06E+01	4.06E+01
ENSG00000105514	RAB3D	0.76	-1.31	-0.39	1.04E-03	-4.06E+01	4.06E+01
ENSG00000007202	KIAA0100	0.77	-1.30	-0.38	1.02E-03	-4.06E+01	4.06E+01
ENSG00000173917	HOXB2	0.75	-1.34	-0.42	1.09E-03	-4.06E+01	4.06E+01
ENSG00000273542	HIST1H4K	0.70	-1.43	-0.51	1.24E-03	-4.06E+01	4.06E+01
ENSG00000057252	SOAT1	0.76	-1.31	-0.39	1.04E-03	-4.06E+01	4.06E+01
ENSG00000106344	RBM28	0.78	-1.29	-0.36	1.01E-03	-4.05E+01	4.05E+01
ENSG00000253103	RP11-946L20.4	1.51	1.51	0.59	1.39E-03	4.05E+01	4.05E+01
ENSG00000130560	UBAC1	0.78	-1.28	-0.36	1.00E-03	-4.04E+01	4.04E+01
ENSG00000138758	SEPT11	1.30	1.30	0.38	1.04E-03	4.04E+01	4.04E+01
ENSG00000255284	AP006621.5	1.50	1.50	0.59	1.38E-03	4.04E+01	4.04E+01
ENSG00000136052	SLC41A2	1.31	1.31	0.39	1.05E-03	4.04E+01	4.04E+01

ENSG00000084090	STARD7	0.77	-1.30	-0.37	1.03E-03	-4.04E+01	4.04E+01
ENSG00000024048	UBR2	1.29	1.29	0.37	1.02E-03	4.03E+01	4.03E+01
ENSG00000103591	AAGAB	0.77	-1.30	-0.38	1.05E-03	-4.03E+01	4.03E+01
ENSG00000179588	ZFPM1	1.76	1.76	0.81	1.90E-03	4.03E+01	4.03E+01
ENSG00000154114	TBCEL	1.29	1.29	0.37	1.03E-03	4.03E+01	4.03E+01
ENSG00000160075	SSU72	1.29	1.29	0.37	1.03E-03	4.02E+01	4.02E+01
ENSG00000139697	SBNO1	1.30	1.30	0.38	1.05E-03	4.01E+01	4.01E+01
ENSG00000169902	TPST1	0.78	-1.29	-0.36	1.03E-03	-4.01E+01	4.01E+01
ENSG00000134330	IAH1	0.78	-1.28	-0.36	1.02E-03	-4.00E+01	4.00E+01
ENSG00000188827	SLX4	1.39	1.39	0.47	1.20E-03	4.00E+01	4.00E+01
ENSG00000140199	SLC12A6	1.28	1.28	0.35	1.02E-03	4.00E+01	4.00E+01
ENSG00000187994	RINL	0.75	-1.33	-0.41	1.11E-03	-3.99E+01	3.99E+01
ENSG00000184113	CLDN5	1.30	1.30	0.38	1.06E-03	3.99E+01	3.99E+01
ENSG00000168067	MAP4K2	0.78	-1.28	-0.36	1.03E-03	-3.99E+01	3.99E+01
ENSG00000101654	RNMT	1.28	1.28	0.35	1.03E-03	3.98E+01	3.98E+01
ENSG00000170515	PA2G4	0.77	-1.30	-0.38	1.07E-03	-3.98E+01	3.98E+01
ENSG00000083635	NUFIP1	0.76	-1.31	-0.39	1.08E-03	-3.98E+01	3.98E+01
ENSG00000138613	APH1B	1.29	1.29	0.36	1.05E-03	3.98E+01	3.98E+01
ENSG00000085433	WDR47	1.27	1.27	0.35	1.03E-03	3.97E+01	3.97E+01
ENSG00000163832	ELP6	0.79	-1.27	-0.35	1.03E-03	-3.97E+01	3.97E+01
ENSG00000066926	FECH	0.78	-1.28	-0.35	1.04E-03	-3.97E+01	3.97E+01
ENSG00000171204	TMEM126B	0.78	-1.28	-0.36	1.05E-03	-3.96E+01	3.96E+01
ENSG00000123297	TSFM	0.78	-1.28	-0.35	1.04E-03	-3.96E+01	3.96E+01
ENSG00000180891	CUEDC1	1.30	1.30	0.38	1.08E-03	3.96E+01	3.96E+01
ENSG00000163050	ADCK3	0.75	-1.34	-0.42	1.15E-03	-3.96E+01	3.96E+01
ENSG00000108469	RECQL5	1.33	1.33	0.41	1.13E-03	3.96E+01	3.96E+01
ENSG00000134253	TRIM45	0.68	-1.47	-0.55	1.38E-03	-3.95E+01	3.95E+01
ENSG00000257923	CUX1	1.29	1.29	0.37	1.07E-03	3.95E+01	3.95E+01
ENSG00000164342	TLR3	0.71	-1.41	-0.50	1.28E-03	-3.95E+01	3.95E+01
ENSG00000215190	LINC00680	0.71	-1.40	-0.49	1.26E-03	-3.94E+01	3.94E+01
ENSG00000151694	ADAM17	1.30	1.30	0.37	1.09E-03	3.92E+01	3.92E+01
ENSG00000155229	MMS19	0.77	-1.30	-0.37	1.09E-03	-3.92E+01	3.92E+01
ENSG00000151923	TIAL1	0.79	-1.27	-0.35	1.06E-03	-3.92E+01	3.92E+01
	RP11-296I10.6	1.38	1.38	0.46	1.25E-03	3.90E+01	3.90E+01
ENSG00000256043	CTSO	1.35	1.35	0.44	1.21E-03	3.90E+01	3.90E+01
ENSG00000182903	ZNF721	1.37	1.37	0.46	1.24E-03	3.89E+01	3.89E+01
ENSG00000224531	C6orf228	1.28	1.28	0.36	1.09E-03	3.89E+01	3.89E+01
ENSG00000007392	LUC7L	0.78	-1.29	-0.37	1.11E-03	-3.88E+01	3.88E+01
ENSG00000123739	PLA2G12A	0.78	-1.28	-0.36	1.10E-03	-3.87E+01	3.87E+01
ENSG00000168386	FILIP1L	1.33	1.33	0.41	1.19E-03	3.86E+01	3.86E+01
ENSG00000135083	CCNJL	1.30	1.30	0.38	1.14E-03	3.85E+01	3.85E+01
ENSG00000169217	CD2BP2	0.78	-1.29	-0.37	1.12E-03	-3.85E+01	3.85E+01
ENSG00000112183	RBM24	1.42	1.42	0.51	1.37E-03	3.84E+01	3.84E+01
ENSG00000158526	TSR2	0.78	-1.29	-0.36	1.12E-03	-3.84E+01	3.84E+01
ENSG00000167815	PRDX2	1.29	1.29	0.37	1.14E-03	3.84E+01	3.84E+01
ENSG00000205362	MT1A	0.63	-1.60	-0.67	1.73E-03	-3.84E+01	3.84E+01
ENSG00000030582	GRN	1.56	1.56	0.64	1.67E-03	3.83E+01	3.83E+01
ENSG00000051620	HEBP2	0.77	-1.30	-0.38	1.16E-03	-3.82E+01	3.82E+01
ENSG00000189308	LIN54	0.78	-1.28	-0.35	1.12E-03	-3.82E+01	3.82E+01
ENSG00000134153	EMC7	0.77	-1.30	-0.38	1.15E-03	-3.82E+01	3.82E+01
ENSG00000107443	CCNJ	1.31	1.31	0.39	1.18E-03	3.81E+01	3.81E+01
ENSG00000091136	LAMB1	1.29	1.29	0.37	1.16E-03	3.80E+01	3.80E+01
ENSG00000126088	UROD	1.29	1.29	0.36	1.15E-03	3.80E+01	3.80E+01
ENSG00000160113	NR2F6	1.27	1.27	0.35	1.13E-03	3.79E+01	3.79E+01
ENSG00000123609	NMI	0.78	-1.28	-0.36	1.15E-03	-3.78E+01	3.78E+01
ENSG00000165312	OTUD1	1.36	1.36	0.44	1.28E-03	3.78E+01	3.78E+01
ENSG00000188636	LDOC1L	1.28	1.28	0.35	1.14E-03	3.78E+01	3.78E+01
ENSG00000166813	KIF7	0.76	-1.31	-0.39	1.21E-03	-3.78E+01	3.78E+01
ENSG00000142657	PGD	0.77	-1.29	-0.37	1.17E-03	-3.77E+01	3.77E+01
ENSG00000168487	BMP1	1.28	1.28	0.36	1.16E-03	3.77E+01	3.77E+01
ENSG00000164930	FZD6	1.30	1.30	0.38	1.19E-03	3.77E+01	3.77E+01
ENSG00000189046	ALKBH2	0.74	-1.36	-0.44	1.30E-03	-3.76E+01	3.76E+01
ENSG00000214765	SEPT7P2	0.72	-1.39	-0.48	1.38E-03	-3.76E+01	3.76E+01
ENSG00000243335	KCTD7	0.75	-1.34	-0.42	1.28E-03	-3.76E+01	3.76E+01
ENSG00000102802	MEDAG	1.28	1.28	0.36	1.17E-03	3.75E+01	3.75E+01
ENSG00000115685	PPP1R7	1.28	1.28	0.35	1.16E-03	3.74E+01	3.74E+01
ENSG00000139725	RHOF	0.62	-1.60	-0.68	1.85E-03	-3.73E+01	3.73E+01
ENSG00000154642	C21orf91	1.44	1.44	0.53	1.50E-03	3.72E+01	3.72E+01
ENSG00000100949	RABGGTA	1.39	1.39	0.47	1.40E-03	3.71E+01	3.71E+01
ENSG00000186448	ZNF197	1.30	1.30	0.37	1.22E-03	3.71E+01	3.71E+01
ENSG00000213390	ARHGAP19	0.78	-1.28	-0.35	1.19E-03	-3.71E+01	3.71E+01
ENSG00000269335	IKBKG	1.30	1.30	0.38	1.23E-03	3.70E+01	3.70E+01
ENSG00000137962	ARHGAP29	1.30	1.30	0.38	1.24E-03	3.70E+01	3.70E+01
ENSG00000128309	MPST	1.29	1.29	0.37	1.22E-03	3.69E+01	3.69E+01
ENSG00000175166	PSMD2	1.30	1.30	0.38	1.25E-03	3.69E+01	3.69E+01
ENSG00000120217	CD274	1.30	1.30	0.38	1.25E-03	3.69E+01	3.69E+01
ENSG00000164631	ZNF12	1.37	1.37	0.45	1.38E-03	3.68E+01	3.68E+01
ENSG00000230124	ACBD6	0.78	-1.28	-0.35	1.21E-03	-3.68E+01	3.68E+01
ENSG00000128626	MRPS12	0.76	-1.31	-0.39	1.27E-03	-3.68E+01	3.68E+01

ENSG00000127804	METTL16	0.78	-1.28	-0.36	1.22E-03	-3.68E+01	3.68E+01
ENSG00000234327	AC012146.7	1.45	1.45	0.53	1.55E-03	3.67E+01	3.67E+01
ENSG00000169955	ZNF747	1.32	1.32	0.40	1.29E-03	3.67E+01	3.67E+01
ENSG00000103005	USB1	0.76	-1.32	-0.40	1.30E-03	-3.66E+01	3.66E+01
ENSG00000230849	GOT2P2	1.68	1.68	0.75	2.11E-03	3.66E+01	3.66E+01
ENSG00000170540	ARL6IP1	0.78	-1.29	-0.37	1.24E-03	-3.66E+01	3.66E+01
ENSG00000108219	TSPAN14	1.29	1.29	0.37	1.24E-03	3.65E+01	3.65E+01
ENSG00000075975	MKRN2	0.77	-1.29	-0.37	1.25E-03	-3.65E+01	3.65E+01
ENSG00000163637	PRICKLE2	0.74	-1.35	-0.44	1.37E-03	-3.65E+01	3.65E+01
ENSG00000084234	APLP2	1.29	1.29	0.37	1.25E-03	3.65E+01	3.65E+01
ENSG00000172301	C17orf79	0.79	-1.27	-0.34	1.21E-03	-3.65E+01	3.65E+01
ENSG00000160310	PRMT2	0.78	-1.28	-0.36	1.24E-03	-3.64E+01	3.64E+01
ENSG00000164808	KIAA0146	0.79	-1.27	-0.35	1.22E-03	-3.64E+01	3.64E+01
ENSG00000128487	SPECC1	1.31	1.31	0.39	1.29E-03	3.64E+01	3.64E+01
ENSG00000266208	CTD-2267D19.3	0.67	-1.49	-0.57	1.68E-03	-3.63E+01	3.63E+01
ENSG00000100410	PHF5A	0.78	-1.28	-0.36	1.25E-03	-3.63E+01	3.63E+01
ENSG00000157625	TAB3	1.28	1.28	0.35	1.24E-03	3.62E+01	3.62E+01
ENSG00000054267	ARID4B	1.38	1.38	0.47	1.46E-03	3.62E+01	3.62E+01
ENSG00000200087	SNORA73B	0.63	-1.58	-0.66	1.92E-03	-3.62E+01	3.62E+01
ENSG00000267519	CTD-3252C9.4	1.37	1.37	0.46	1.44E-03	3.62E+01	3.62E+01
ENSG00000176155	CCDC57	1.33	1.33	0.41	1.36E-03	3.62E+01	3.62E+01
ENSG00000136280	CCM2	0.79	-1.26	-0.34	1.22E-03	-3.61E+01	3.61E+01
ENSG00000213918	DNASE1	1.34	1.34	0.42	1.37E-03	3.61E+01	3.61E+01
ENSG00000170915	PAQR8	1.33	1.33	0.41	1.36E-03	3.61E+01	3.61E+01
ENSG00000123737	EXOSC9	0.78	-1.28	-0.36	1.26E-03	-3.60E+01	3.60E+01
ENSG00000053900	ANAPC4	0.77	-1.29	-0.37	1.29E-03	-3.60E+01	3.60E+01
ENSG00000198931	APRT	0.78	-1.29	-0.36	1.29E-03	-3.58E+01	3.58E+01
ENSG00000188312	CENPP	0.75	-1.34	-0.42	1.41E-03	-3.57E+01	3.57E+01
ENSG00000133678	TMEM254	1.36	1.36	0.44	1.46E-03	3.56E+01	3.56E+01
ENSG00000159214	CCDC24	0.67	-1.49	-0.57	1.74E-03	-3.56E+01	3.56E+01
ENSG00000109062	SLC9A3R1	0.77	-1.30	-0.38	1.33E-03	-3.56E+01	3.56E+01
ENSG00000177200	CHD9	1.49	1.49	0.57	1.76E-03	3.54E+01	3.54E+01
ENSG00000107815	C10orf2	0.74	-1.36	-0.44	1.47E-03	-3.53E+01	3.53E+01
ENSG00000214174	AMZ2P1	1.39	1.39	0.48	1.56E-03	3.53E+01	3.53E+01
ENSG00000144026	ZNF514	1.29	1.29	0.37	1.34E-03	3.53E+01	3.53E+01
ENSG00000229334	AC046143.3	0.64	-1.56	-0.64	1.95E-03	-3.53E+01	3.53E+01
ENSG00000117475	BLZF1	1.35	1.35	0.43	1.46E-03	3.52E+01	3.52E+01
ENSG00000177613	CSTF2T	0.79	-1.27	-0.34	1.30E-03	-3.52E+01	3.52E+01
ENSG00000235609	AF127936.7	0.65	-1.55	-0.63	1.94E-03	-3.52E+01	3.52E+01
ENSG00000171492	LRRC8D	0.78	-1.27	-0.35	1.31E-03	-3.52E+01	3.52E+01
ENSG00000278845	MRPL45	1.27	1.27	0.34	1.30E-03	3.51E+01	3.51E+01
ENSG00000161677	JOSD2	1.35	1.35	0.43	1.47E-03	3.51E+01	3.51E+01
ENSG00000143222	UFC1	0.77	-1.29	-0.37	1.36E-03	-3.51E+01	3.51E+01
ENSG00000023516	AKAP11	1.47	1.47	0.56	1.76E-03	3.51E+01	3.51E+01
ENSG00000133935	C14orf1	0.79	-1.27	-0.34	1.31E-03	-3.50E+01	3.50E+01
ENSG00000145431	PDGFC	1.32	1.32	0.40	1.41E-03	3.50E+01	3.50E+01
ENSG00000141627	DYM	0.78	-1.28	-0.35	1.34E-03	-3.49E+01	3.49E+01
ENSG00000198774	RASSF9	0.62	-1.61	-0.69	2.12E-03	-3.49E+01	3.49E+01
ENSG00000198920	KIAA0753	1.27	1.27	0.34	1.32E-03	3.49E+01	3.49E+01
ENSG00000150455	TIRAP	0.71	-1.42	-0.50	1.65E-03	-3.49E+01	3.49E+01
ENSG00000060982	BCAT1	1.38	1.38	0.46	1.57E-03	3.49E+01	3.49E+01
ENSG00000186222	BLOC1S4	1.32	1.32	0.40	1.43E-03	3.48E+01	3.48E+01
ENSG00000141522	ARHGDIA	1.38	1.38	0.46	1.56E-03	3.48E+01	3.48E+01
ENSG00000129757	CDKN1C	1.50	1.50	0.58	1.85E-03	3.48E+01	3.48E+01
ENSG00000086991	NOX4	0.79	-1.27	-0.35	1.34E-03	-3.47E+01	3.47E+01
ENSG00000184232	OAF	1.29	1.29	0.37	1.40E-03	3.46E+01	3.46E+01
ENSG00000185361	TNFAIP8L1	1.28	1.28	0.36	1.37E-03	3.46E+01	3.46E+01
ENSG00000178202	KDELC2	1.28	1.28	0.36	1.38E-03	3.46E+01	3.46E+01
ENSG00000152433	ZNF547	1.50	1.50	0.59	1.91E-03	3.43E+01	3.43E+01
ENSG00000167778	SPRYD3	0.79	-1.27	-0.35	1.38E-03	-3.42E+01	3.42E+01
ENSG00000106853	PTGR1	0.75	-1.33	-0.41	1.51E-03	-3.42E+01	3.42E+01
ENSG00000184209	SNRNP35	0.77	-1.30	-0.38	1.45E-03	-3.41E+01	3.41E+01
ENSG00000134779	TPGS2	0.78	-1.28	-0.36	1.42E-03	-3.41E+01	3.41E+01
ENSG00000254615	RP11-395G23.3	0.66	-1.51	-0.60	1.97E-03	-3.41E+01	3.41E+01
ENSG00000131018	SYNE1	1.32	1.32	0.40	1.49E-03	3.41E+01	3.41E+01
ENSG00000231025	RP11-175O19.4	1.39	1.39	0.47	1.67E-03	3.40E+01	3.40E+01
ENSG00000145016	KIAA0226	1.30	1.30	0.38	1.46E-03	3.40E+01	3.40E+01
ENSG00000128915	NARG2	0.76	-1.32	-0.40	1.50E-03	-3.40E+01	3.40E+01
ENSG00000134852	CLOCK	1.39	1.39	0.48	1.68E-03	3.40E+01	3.40E+01
ENSG00000198815	FOXJ3	1.36	1.36	0.45	1.61E-03	3.39E+01	3.39E+01
ENSG00000168283	BMI1	1.29	1.29	0.36	1.44E-03	3.39E+01	3.39E+01
ENSG00000057757	PITHD1	0.78	-1.28	-0.36	1.43E-03	-3.39E+01	3.39E+01
ENSG00000105698	USF2	1.28	1.28	0.36	1.43E-03	3.38E+01	3.38E+01
ENSG00000174373	RALGAPA1	1.29	1.29	0.36	1.45E-03	3.38E+01	3.38E+01
ENSG00000030066	NUP160	0.79	-1.27	-0.35	1.42E-03	-3.38E+01	3.38E+01
ENSG00000091127	PUS7	0.78	-1.29	-0.37	1.46E-03	-3.37E+01	3.37E+01
ENSG00000179218	CALR	0.77	-1.30	-0.37	1.48E-03	-3.37E+01	3.37E+01
ENSG00000141452	C18orf8	1.26	1.26	0.34	1.41E-03	3.36E+01	3.36E+01
ENSG00000128245	YWHAH	1.28	1.28	0.36	1.46E-03	3.36E+01	3.36E+01

ENSG00000108578	BLMH	0.78	-1.28	-0.35	1.45E-03	-3.36E+01	3.36E+01
ENSG00000163320	CGGBP1	1.31	1.31	0.39	1.53E-03	3.35E+01	3.35E+01
ENSG00000211584	SLC48A1	0.77	-1.31	-0.39	1.52E-03	-3.35E+01	3.35E+01
ENSG00000173894	CBX2	1.27	1.27	0.34	1.44E-03	3.35E+01	3.35E+01
ENSG00000126106	TMEM53	1.43	1.43	0.52	1.84E-03	3.35E+01	3.35E+01
ENSG00000170296	GABARAP	0.63	-1.58	-0.66	2.24E-03	-3.33E+01	3.33E+01
ENSG00000106397	PLOD3	1.41	1.41	0.50	1.80E-03	3.33E+01	3.33E+01
ENSG00000136243	NUPL2	0.79	-1.26	-0.34	1.45E-03	-3.32E+01	3.32E+01
ENSG00000146858	ZC3HAV1L	1.35	1.35	0.43	1.65E-03	3.32E+01	3.32E+01
ENSG00000213638	ADAT3	1.81	1.81	0.85	2.97E-03	3.32E+01	3.32E+01
ENSG00000144840	RABL3	0.79	-1.26	-0.33	1.45E-03	-3.31E+01	3.31E+01
ENSG00000136631	VPS45	0.79	-1.26	-0.34	1.46E-03	-3.30E+01	3.30E+01
ENSG00000237036	ZEB1-AS1	1.48	1.48	0.56	2.00E-03	3.30E+01	3.30E+01
ENSG00000145194	ECE2	0.71	-1.40	-0.49	1.80E-03	-3.30E+01	3.30E+01
ENSG00000160145	KALRN	0.74	-1.35	-0.43	1.68E-03	-3.29E+01	3.29E+01
ENSG00000101188	NTSR1	1.56	1.56	0.64	2.25E-03	3.28E+01	3.28E+01
ENSG00000120129	DUSP1	1.27	1.27	0.35	1.50E-03	3.28E+01	3.28E+01
	RP11-151N17.2	0.71	-1.41	-0.50	1.86E-03	-3.28E+01	3.28E+01
ENSG00000260035	CTD-2651B20.6	0.59	-1.69	-0.76	2.68E-03	-3.27E+01	3.27E+01
ENSG00000164209	SLC25A46	1.31	1.31	0.39	1.60E-03	3.27E+01	3.27E+01
ENSG00000163964	PIGX	0.76	-1.31	-0.39	1.62E-03	-3.27E+01	3.27E+01
ENSG00000163697	APBB2	1.29	1.29	0.37	1.56E-03	3.26E+01	3.26E+01
ENSG00000196632	WNK3	0.72	-1.39	-0.48	1.83E-03	-3.26E+01	3.26E+01
ENSG00000140525	FANCI	0.78	-1.28	-0.36	1.54E-03	-3.26E+01	3.26E+01
ENSG00000101421	CHMP4B	1.36	1.36	0.44	1.74E-03	3.26E+01	3.26E+01
ENSG00000002549	LAP3	0.78	-1.28	-0.36	1.56E-03	-3.25E+01	3.25E+01
ENSG00000188483	IER5L	0.74	-1.36	-0.44	1.75E-03	-3.25E+01	3.25E+01
ENSG00000085733	CTTN	1.28	1.28	0.36	1.56E-03	3.25E+01	3.25E+01
ENSG00000141076	CIRH1A	0.74	-1.34	-0.43	1.72E-03	-3.24E+01	3.24E+01
ENSG00000125834	STK35	1.28	1.28	0.36	1.57E-03	3.24E+01	3.24E+01
ENSG00000012983	MAP4K5	1.30	1.30	0.38	1.61E-03	3.24E+01	3.24E+01
ENSG00000112759	SLC29A1	0.78	-1.28	-0.36	1.56E-03	-3.24E+01	3.24E+01
ENSG00000119559	C19orf25	0.77	-1.31	-0.38	1.63E-03	-3.23E+01	3.23E+01
ENSG00000118058	MLL	1.30	1.30	0.38	1.63E-03	3.23E+01	3.23E+01
ENSG00000171858	RPS21	0.70	-1.44	-0.52	1.98E-03	-3.23E+01	3.23E+01
ENSG00000113460	BRIX1	0.79	-1.27	-0.34	1.54E-03	-3.22E+01	3.22E+01
ENSG00000135862	LAMC1	1.42	1.42	0.51	1.94E-03	3.22E+01	3.22E+01
ENSG00000164741	DLC1	1.35	1.35	0.43	1.76E-03	3.22E+01	3.22E+01
ENSG00000197594	ENPP1	0.77	-1.29	-0.37	1.62E-03	-3.22E+01	3.22E+01
ENSG00000014216	CAPN1	1.30	1.30	0.38	1.63E-03	3.22E+01	3.22E+01
ENSG00000160688	FLAD1	0.79	-1.26	-0.33	1.54E-03	-3.21E+01	3.21E+01
ENSG00000125845	BMP2	1.29	1.29	0.37	1.64E-03	3.20E+01	3.20E+01
ENSG00000143093	STRIP1	0.72	-1.38	-0.47	1.88E-03	-3.19E+01	3.19E+01
ENSG00000165675	ENOX2	0.77	-1.30	-0.38	1.65E-03	-3.19E+01	3.19E+01
ENSG00000035115	SH3YL1	1.28	1.28	0.36	1.62E-03	3.18E+01	3.18E+01
ENSG00000145632	PLK2	1.29	1.29	0.36	1.65E-03	3.17E+01	3.17E+01
ENSG00000175582	RAB6A	1.28	1.28	0.36	1.64E-03	3.17E+01	3.17E+01
ENSG00000065526	SPEN	1.31	1.31	0.39	1.70E-03	3.17E+01	3.17E+01
ENSG00000198736	MSRB1	0.74	-1.35	-0.43	1.82E-03	-3.17E+01	3.17E+01
ENSG00000105877	DNAH11	0.72	-1.39	-0.47	1.92E-03	-3.17E+01	3.17E+01
ENSG00000008988	RPS20	0.75	-1.33	-0.41	1.76E-03	-3.16E+01	3.16E+01
ENSG00000115762	PLEKHB2	1.32	1.32	0.40	1.75E-03	3.15E+01	3.15E+01
ENSG00000106771	TMEM245	1.28	1.28	0.36	1.66E-03	3.15E+01	3.15E+01
ENSG00000242372	EIF6	0.77	-1.29	-0.37	1.68E-03	-3.15E+01	3.15E+01
ENSG00000076108	BAZ2A	1.32	1.32	0.40	1.75E-03	3.14E+01	3.14E+01
ENSG00000108963	DPH1	1.34	1.34	0.42	1.81E-03	3.14E+01	3.14E+01
ENSG00000013503	POLR3B	0.77	-1.30	-0.38	1.72E-03	-3.14E+01	3.14E+01
ENSG00000065833	ME1	0.78	-1.28	-0.35	1.66E-03	-3.13E+01	3.13E+01
ENSG00000198612	COPS8	0.78	-1.28	-0.36	1.67E-03	-3.13E+01	3.13E+01
ENSG00000122986	HVCN1	0.72	-1.39	-0.48	1.98E-03	-3.13E+01	3.13E+01
ENSG00000176438	LINC00341	1.43	1.43	0.51	2.08E-03	3.12E+01	3.12E+01
ENSG00000237380	HOXD-AS2	0.65	-1.53	-0.62	2.41E-03	-3.12E+01	3.12E+01
ENSG00000140688	C16orf58	1.28	1.28	0.35	1.68E-03	3.12E+01	3.12E+01
ENSG00000013441	CLK1	0.77	-1.31	-0.38	1.75E-03	-3.12E+01	3.12E+01
ENSG00000090661	CERS4	1.48	1.48	0.57	2.26E-03	3.11E+01	3.11E+01
ENSG00000204852	TCTN1	1.26	1.26	0.33	1.64E-03	3.11E+01	3.11E+01
ENSG00000107338	SHB	1.34	1.34	0.42	1.85E-03	3.11E+01	3.11E+01
ENSG00000122591	FAM126A	1.31	1.31	0.39	1.79E-03	3.11E+01	3.11E+01
ENSG00000022556	NLRP2	0.69	-1.45	-0.54	2.18E-03	-3.11E+01	3.11E+01
ENSG00000204525	HLA-C	1.28	1.28	0.36	1.71E-03	3.10E+01	3.10E+01
ENSG00000121104	FAM117A	0.74	-1.35	-0.43	1.89E-03	-3.10E+01	3.10E+01
ENSG00000067829	IDH3G	0.79	-1.27	-0.34	1.68E-03	-3.09E+01	3.09E+01
ENSG00000151553	FAM160B1	1.28	1.28	0.35	1.70E-03	3.09E+01	3.09E+01
ENSG00000213397	HAUS7	0.63	-1.60	-0.68	2.68E-03	-3.09E+01	3.09E+01
ENSG00000129562	DAD1	1.29	1.29	0.36	1.74E-03	3.09E+01	3.09E+01
ENSG00000165282	PIGO	0.77	-1.31	-0.38	1.79E-03	-3.09E+01	3.09E+01
ENSG00000110013	SIAE	1.27	1.27	0.34	1.69E-03	3.09E+01	3.09E+01
ENSG00000100714	MTHFD1	0.76	-1.31	-0.39	1.80E-03	-3.09E+01	3.09E+01
ENSG00000020577	SAMD4A	1.27	1.27	0.35	1.71E-03	3.08E+01	3.08E+01

ENSG00000177432	NAP1L5	0.76	-1.31	-0.39	1.80E-03	-3.08E+01	3.08E+01
ENSG00000109390	NDUFC1	0.77	-1.29	-0.37	1.77E-03	-3.08E+01	3.08E+01
ENSG00000047932	GOPC	1.27	1.27	0.34	1.71E-03	3.07E+01	3.07E+01
	RP11-701H24.2	1.43	1.43	0.51	2.16E-03	3.07E+01	3.07E+01
ENSG00000138182	KIF20B	0.76	-1.32	-0.40	1.85E-03	-3.06E+01	3.06E+01
ENSG00000152291	TGOLN2	1.29	1.29	0.36	1.76E-03	3.06E+01	3.06E+01
ENSG00000100612	DHRS7	0.79	-1.26	-0.34	1.71E-03	-3.06E+01	3.06E+01
ENSG00000100105	PATZ1	0.78	-1.28	-0.35	1.75E-03	-3.05E+01	3.05E+01
ENSG00000182004	SNRPE	0.76	-1.31	-0.39	1.85E-03	-3.05E+01	3.05E+01
ENSG00000146587	RBAK	1.40	1.40	0.48	2.10E-03	3.05E+01	3.05E+01
ENSG00000133142	TCEAL4	0.77	-1.29	-0.37	1.81E-03	-3.04E+01	3.04E+01
ENSG00000198231	DDX42	0.78	-1.28	-0.36	1.78E-03	-3.04E+01	3.04E+01
ENSG00000160917	CPSF4	0.79	-1.26	-0.34	1.73E-03	-3.03E+01	3.03E+01
ENSG00000117385	LEPRE1	1.29	1.29	0.36	1.81E-03	3.03E+01	3.03E+01
ENSG00000022840	RNF10	1.29	1.29	0.37	1.82E-03	3.03E+01	3.03E+01
ENSG00000173926	MARCH3	0.79	-1.27	-0.34	1.76E-03	-3.03E+01	3.03E+01
ENSG00000176619	LMNB2	0.78	-1.29	-0.36	1.81E-03	-3.02E+01	3.02E+01
ENSG00000183665	TRMT12	0.77	-1.30	-0.38	1.85E-03	-3.02E+01	3.02E+01
ENSG00000166925	TSC22D4	1.27	1.27	0.34	1.77E-03	3.01E+01	3.01E+01
ENSG00000189339	SLC35E2B	1.26	1.26	0.34	1.77E-03	3.01E+01	3.01E+01
ENSG00000001561	ENPP4	0.72	-1.39	-0.48	2.15E-03	-3.01E+01	3.01E+01
ENSG00000035403	VCL	1.29	1.29	0.37	1.85E-03	3.00E+01	3.00E+01
ENSG00000166436	TRIM66	1.43	1.43	0.52	2.27E-03	3.00E+01	3.00E+01
ENSG00000239713	APOBEC3G	1.39	1.39	0.47	2.13E-03	3.00E+01	3.00E+01
ENSG00000130816	DNMT1	0.73	-1.38	-0.46	2.11E-03	-3.00E+01	3.00E+01
ENSG00000237765	FAM200B	1.27	1.27	0.34	1.80E-03	2.99E+01	2.99E+01
ENSG00000264885	RP11-815I9.4	1.58	1.58	0.66	2.80E-03	2.99E+01	2.99E+01
ENSG00000175806	MSRA	1.29	1.29	0.36	1.86E-03	2.99E+01	2.99E+01
ENSG00000165169	DYNLT3	1.32	1.32	0.41	1.97E-03	2.99E+01	2.99E+01
ENSG00000105708	ZNF14	1.42	1.42	0.51	2.27E-03	2.98E+01	2.98E+01
ENSG00000089916	GPATCH2L	1.26	1.26	0.34	1.82E-03	2.97E+01	2.97E+01
ENSG00000148908	RGS10	1.26	1.26	0.33	1.80E-03	2.96E+01	2.96E+01
ENSG00000154553	PDLIM3	0.79	-1.27	-0.35	1.85E-03	-2.96E+01	2.96E+01
ENSG00000100354	TNRC6B	1.27	1.27	0.35	1.86E-03	2.96E+01	2.96E+01
ENSG00000165819	METTL3	0.79	-1.27	-0.34	1.84E-03	-2.95E+01	2.95E+01
ENSG00000155962	CLIC2	0.60	-1.66	-0.73	3.18E-03	-2.95E+01	2.95E+01
ENSG00000204209	DAXX	1.29	1.29	0.37	1.92E-03	2.94E+01	2.94E+01
ENSG00000155380	SLC16A1	0.78	-1.28	-0.36	1.91E-03	-2.94E+01	2.94E+01
ENSG00000234449	RP11-706O15.3	1.41	1.41	0.49	2.31E-03	2.93E+01	2.93E+01
ENSG00000160211	G6PD	0.79	-1.26	-0.34	1.86E-03	-2.93E+01	2.93E+01
	RP11-304L19.5	0.67	-1.48	-0.57	2.57E-03	-2.93E+01	2.93E+01
ENSG00000022976	ZNF839	1.34	1.34	0.43	2.11E-03	2.93E+01	2.93E+01
ENSG00000090273	NUDC	1.28	1.28	0.36	1.92E-03	2.92E+01	2.92E+01
ENSG00000136527	TRA2B	0.78	-1.28	-0.36	1.92E-03	-2.92E+01	2.92E+01
ENSG00000128699	ORMDL1	0.78	-1.28	-0.35	1.91E-03	-2.92E+01	2.92E+01
ENSG00000138750	NUP54	0.78	-1.28	-0.36	1.93E-03	-2.92E+01	2.92E+01
ENSG00000185825	BCAP31	1.27	1.27	0.35	1.91E-03	2.91E+01	2.91E+01
ENSG00000104714	ERICH1	1.36	1.36	0.45	2.19E-03	2.91E+01	2.91E+01
ENSG00000106546	AHR	1.28	1.28	0.35	1.93E-03	2.91E+01	2.91E+01
ENSG00000171421	MRPL36	0.79	-1.26	-0.34	1.89E-03	-2.90E+01	2.90E+01
ENSG00000155100	OTUD6B	0.79	-1.27	-0.35	1.93E-03	-2.90E+01	2.90E+01
ENSG00000205885	C1RL-AS1	1.46	1.46	0.54	2.53E-03	2.90E+01	2.90E+01
ENSG00000130640	TUBGCP2	1.29	1.29	0.36	1.98E-03	2.89E+01	2.89E+01
ENSG00000160972	PPP1R16A	0.78	-1.28	-0.36	1.97E-03	-2.89E+01	2.89E+01
ENSG00000105726	ATP13A1	1.47	1.47	0.56	2.58E-03	2.89E+01	2.89E+01
ENSG00000075131	TIPIN	0.79	-1.27	-0.35	1.94E-03	-2.89E+01	2.89E+01
ENSG00000106080	FKBP14	0.78	-1.29	-0.36	1.99E-03	-2.89E+01	2.89E+01
ENSG00000109113	RAB34	0.78	-1.27	-0.35	1.96E-03	-2.88E+01	2.88E+01
ENSG00000078401	EDN1	0.77	-1.29	-0.37	2.03E-03	-2.87E+01	2.87E+01
ENSG00000177853	ZNF518A	1.55	1.55	0.63	2.91E-03	2.86E+01	2.86E+01
ENSG00000154727	GABPA	1.45	1.45	0.53	2.56E-03	2.86E+01	2.86E+01
ENSG00000162645	GBP2	0.79	-1.27	-0.34	1.97E-03	-2.86E+01	2.86E+01
ENSG00000140299	BNIP2	1.32	1.32	0.40	2.13E-03	2.86E+01	2.86E+01
ENSG00000128266	GNAZ	1.59	1.59	0.67	3.11E-03	2.85E+01	2.85E+01
ENSG00000077782	FGFR1	1.28	1.28	0.36	2.02E-03	2.85E+01	2.85E+01
ENSG00000135924	DNAJB2	1.27	1.27	0.34	1.97E-03	2.85E+01	2.85E+01
ENSG00000117751	PPP1R8	0.79	-1.27	-0.34	1.98E-03	-2.85E+01	2.85E+01
ENSG00000169188	APEX2	0.78	-1.29	-0.36	2.06E-03	-2.84E+01	2.84E+01
ENSG00000134313	KIDINS220	1.28	1.28	0.36	2.04E-03	2.84E+01	2.84E+01
ENSG00000238961	SNORA47	0.60	-1.68	-0.75	3.51E-03	-2.83E+01	2.83E+01
ENSG00000168884	TNIP2	0.80	-1.26	-0.33	1.97E-03	-2.83E+01	2.83E+01
ENSG00000171163	ZNF692	0.77	-1.30	-0.38	2.12E-03	-2.83E+01	2.83E+01
ENSG00000166166	TRMT61A	0.76	-1.31	-0.39	2.17E-03	-2.82E+01	2.82E+01
ENSG00000019485	PRDM11	1.36	1.36	0.45	2.33E-03	2.82E+01	2.82E+01
ENSG00000088876	ZNF343	0.78	-1.29	-0.36	2.08E-03	-2.82E+01	2.82E+01
ENSG00000141562	NARF	1.26	1.26	0.33	2.00E-03	2.82E+01	2.82E+01
ENSG00000114744	COMMD2	0.79	-1.26	-0.33	2.01E-03	-2.81E+01	2.81E+01
ENSG00000137207	YIPF3	1.29	1.29	0.37	2.12E-03	2.81E+01	2.81E+01
ENSG00000186889	TMEM17	0.64	-1.55	-0.63	3.05E-03	-2.81E+01	2.81E+01

ENSG00000069974	RAB27A	0.77	-1.30	-0.38	2.15E-03	-2.80E+01	2.80E+01
ENSG00000142864	SERBP1	0.79	-1.27	-0.35	2.06E-03	-2.80E+01	2.80E+01
ENSG00000186716	BCR	1.32	1.32	0.40	2.21E-03	2.80E+01	2.80E+01
ENSG00000022267	FHL1	1.27	1.27	0.34	2.05E-03	2.80E+01	2.80E+01
ENSG00000106554	CHCHD3	0.79	-1.27	-0.34	2.05E-03	-2.80E+01	2.80E+01
ENSG00000109180	OCIAD1	1.29	1.29	0.36	2.12E-03	2.79E+01	2.79E+01
ENSG00000142039	CCDC97	0.76	-1.32	-0.40	2.22E-03	-2.79E+01	2.79E+01
ENSG00000091651	ORC6	0.80	-1.26	-0.33	2.03E-03	-2.79E+01	2.79E+01
ENSG00000224167	RP3-522D1.1	0.74	-1.35	-0.44	2.36E-03	-2.79E+01	2.79E+01
ENSG00000260196	RP1-239B22.5	0.71	-1.41	-0.50	2.57E-03	-2.79E+01	2.79E+01
ENSG00000196417	ZNF765	1.33	1.33	0.41	2.28E-03	2.78E+01	2.78E+01
ENSG00000057294	PKP2	1.36	1.36	0.44	2.37E-03	2.78E+01	2.78E+01
ENSG00000120159	CAAP1	0.78	-1.28	-0.36	2.12E-03	-2.78E+01	2.78E+01
ENSG00000116497	S100PBP	1.25	1.25	0.33	2.04E-03	2.78E+01	2.78E+01
ENSG00000180357	ZNF609	1.29	1.29	0.37	2.16E-03	2.78E+01	2.78E+01
ENSG00000093217	XYLB	0.69	-1.44	-0.53	2.71E-03	-2.77E+01	2.77E+01
ENSG00000143514	TP53BP2	1.26	1.26	0.34	2.09E-03	2.77E+01	2.77E+01
ENSG00000099901	RANBP1	0.79	-1.27	-0.35	2.12E-03	-2.76E+01	2.76E+01
ENSG00000111845	PAK1IP1	0.80	-1.26	-0.33	2.07E-03	-2.76E+01	2.76E+01
ENSG00000101193	GID8	1.25	1.25	0.33	2.06E-03	2.76E+01	2.76E+01
ENSG00000108819	PPP1R9B	1.26	1.26	0.33	2.08E-03	2.75E+01	2.75E+01
ENSG00000042445	RETSAT	0.80	-1.25	-0.33	2.07E-03	-2.75E+01	2.75E+01
ENSG00000200959	SNORA74A	0.70	-1.42	-0.51	2.68E-03	-2.75E+01	2.75E+01
ENSG00000068366	ACSL4	1.39	1.39	0.48	2.57E-03	2.75E+01	2.75E+01
ENSG00000120156	TEK	0.78	-1.28	-0.35	2.15E-03	-2.75E+01	2.75E+01
ENSG00000169242	EFNA1	1.27	1.27	0.35	2.15E-03	2.75E+01	2.75E+01
ENSG00000185875	THNSL1	1.34	1.34	0.43	2.39E-03	2.75E+01	2.75E+01
ENSG00000249242	TMEM150C	0.69	-1.45	-0.54	2.78E-03	-2.75E+01	2.75E+01
ENSG00000103264	FBXO31	1.28	1.28	0.36	2.18E-03	2.74E+01	2.74E+01
ENSG00000165055	METTL2B	0.79	-1.26	-0.33	2.12E-03	-2.74E+01	2.74E+01
ENSG00000113140	SPARC	1.28	1.28	0.35	2.17E-03	2.74E+01	2.74E+01
ENSG00000113068	PFDN1	1.27	1.27	0.34	2.15E-03	2.74E+01	2.74E+01
ENSG00000184840	TMED9	0.76	-1.31	-0.39	2.31E-03	-2.73E+01	2.73E+01
ENSG00000166965	RCCD1	0.75	-1.32	-0.41	2.35E-03	-2.73E+01	2.73E+01
ENSG00000156140	ADAMTS3	1.45	1.45	0.53	2.83E-03	2.72E+01	2.72E+01
ENSG00000146350	C6orf170	1.42	1.42	0.50	2.72E-03	2.72E+01	2.72E+01
ENSG00000140006	WDR89	0.76	-1.32	-0.40	2.37E-03	-2.72E+01	2.72E+01
ENSG00000160953	MUM1	1.26	1.26	0.33	2.14E-03	2.71E+01	2.71E+01
ENSG00000155542	SETD9	0.77	-1.30	-0.37	2.29E-03	-2.71E+01	2.71E+01
ENSG00000175105	ZNF654	1.42	1.42	0.51	2.75E-03	2.71E+01	2.71E+01
ENSG00000101928	MOSPD1	1.27	1.27	0.34	2.21E-03	2.70E+01	2.70E+01
ENSG00000168275	COA6	0.77	-1.30	-0.38	2.32E-03	-2.70E+01	2.70E+01
ENSG00000151240	DIP2C	1.30	1.30	0.38	2.32E-03	2.70E+01	2.70E+01
ENSG00000105223	PLD3	1.32	1.32	0.40	2.41E-03	2.68E+01	2.68E+01
ENSG00000178031	ADAMTSL1	0.77	-1.30	-0.38	2.35E-03	-2.68E+01	2.68E+01
ENSG00000115970	THADA	0.80	-1.25	-0.33	2.19E-03	-2.67E+01	2.67E+01
ENSG00000137710	RDX	1.34	1.34	0.43	2.54E-03	2.67E+01	2.67E+01
ENSG00000067900	ROCK1	1.42	1.42	0.51	2.83E-03	2.67E+01	2.67E+01
ENSG00000123416	TUBA1B	0.78	-1.28	-0.35	2.30E-03	-2.66E+01	2.66E+01
ENSG00000103319	EEF2K	0.76	-1.32	-0.40	2.47E-03	-2.66E+01	2.66E+01
ENSG00000138496	PARP9	0.79	-1.27	-0.34	2.28E-03	-2.66E+01	2.66E+01
ENSG00000112984	KIF20A	0.77	-1.30	-0.38	2.39E-03	-2.66E+01	2.66E+01
ENSG00000117139	KDM5B	1.28	1.28	0.36	2.33E-03	2.65E+01	2.65E+01
ENSG00000197083	ZNF300P1	0.62	-1.61	-0.69	3.69E-03	-2.65E+01	2.65E+01
ENSG00000181481	RNF135	1.29	1.29	0.36	2.36E-03	2.65E+01	2.65E+01
ENSG00000147996	CBWD5	0.68	-1.47	-0.55	3.06E-03	-2.65E+01	2.65E+01
ENSG00000166441	RPL27A	0.76	-1.31	-0.39	2.44E-03	-2.65E+01	2.65E+01
ENSG00000176142	TMEM39A	0.79	-1.27	-0.34	2.29E-03	-2.65E+01	2.65E+01
ENSG00000164941	INTS8	1.29	1.29	0.36	2.36E-03	2.65E+01	2.65E+01
ENSG00000164252	AGGF1	1.29	1.29	0.36	2.36E-03	2.65E+01	2.65E+01
ENSG00000121964	GTDC1	0.79	-1.26	-0.33	2.28E-03	-2.64E+01	2.64E+01
ENSG00000125945	ZNF436	1.25	1.25	0.32	2.25E-03	2.64E+01	2.64E+01
ENSG00000204219	TCEA3	0.65	-1.55	-0.63	3.45E-03	-2.63E+01	2.63E+01
ENSG00000177732	SOX12	1.32	1.32	0.40	2.53E-03	2.62E+01	2.62E+01
ENSG00000185088	RPS27L	1.32	1.32	0.41	2.56E-03	2.62E+01	2.62E+01
ENSG00000108984	MAP2K6	0.79	-1.27	-0.34	2.35E-03	-2.62E+01	2.62E+01
ENSG00000119950	MXI1	1.31	1.31	0.39	2.52E-03	2.62E+01	2.62E+01
ENSG00000164466	SFXN1	0.79	-1.27	-0.34	2.34E-03	-2.61E+01	2.61E+01
ENSG00000136521	NDUFB5	0.78	-1.27	-0.35	2.38E-03	-2.61E+01	2.61E+01
ENSG00000089682	RBM41	1.32	1.32	0.40	2.54E-03	2.61E+01	2.61E+01
ENSG00000103343	ZNF174	1.35	1.35	0.43	2.68E-03	2.61E+01	2.61E+01
ENSG00000139793	MBNL2	1.36	1.36	0.45	2.73E-03	2.60E+01	2.60E+01
ENSG00000138640	FAM13A	0.78	-1.28	-0.36	2.41E-03	-2.60E+01	2.60E+01
ENSG00000221944	TIGD1	1.32	1.32	0.40	2.58E-03	2.60E+01	2.60E+01
ENSG00000196793	ZNF239	0.66	-1.51	-0.59	3.38E-03	-2.59E+01	2.59E+01
ENSG00000169714	CNBP	0.78	-1.28	-0.35	2.43E-03	-2.59E+01	2.59E+01
ENSG00000175426	PCSK1	0.74	-1.35	-0.44	2.75E-03	-2.58E+01	2.58E+01
ENSG00000130723	PRRC2B	1.32	1.32	0.40	2.61E-03	2.58E+01	2.58E+01
ENSG00000153201	RANBP2	1.54	1.54	0.63	3.58E-03	2.58E+01	2.58E+01

ENSG00000258297	RP11-658F2.8	0.76	-1.31	-0.39	2.60E-03	-2.57E+01	2.57E+01
ENSG00000177464	GPR4	1.32	1.32	0.40	2.64E-03	2.57E+01	2.57E+01
ENSG00000076685	NT5C2	1.29	1.29	0.37	2.52E-03	2.57E+01	2.57E+01
ENSG00000275714	HIST1H3A	0.78	-1.28	-0.36	2.48E-03	-2.57E+01	2.57E+01
ENSG00000197971	MBP	1.28	1.28	0.35	2.48E-03	2.56E+01	2.56E+01
ENSG00000163950	SLBP	0.78	-1.28	-0.35	2.49E-03	-2.56E+01	2.56E+01
ENSG00000138629	UBL7	1.25	1.25	0.32	2.38E-03	2.55E+01	2.55E+01
ENSG00000112977	DAP	1.27	1.27	0.34	2.47E-03	2.55E+01	2.55E+01
ENSG00000227077	AC107983.4	0.75	-1.33	-0.41	2.73E-03	-2.54E+01	2.54E+01
ENSG00000100206	DMC1	1.54	1.54	0.62	3.66E-03	2.54E+01	2.54E+01
ENSG00000178685	PARP10	0.77	-1.30	-0.38	2.61E-03	-2.54E+01	2.54E+01
ENSG00000269044	CTC-429P9.3	0.66	-1.52	-0.60	3.59E-03	-2.53E+01	2.53E+01
ENSG00000152422	XRCC4	0.74	-1.36	-0.44	2.87E-03	-2.53E+01	2.53E+01
ENSG00000160201	U2AF1	0.77	-1.30	-0.38	2.65E-03	-2.53E+01	2.53E+01
ENSG00000204590	GNL1	0.79	-1.27	-0.35	2.54E-03	-2.52E+01	2.52E+01
ENSG00000198889	DCAF12L1	0.71	-1.41	-0.50	3.15E-03	-2.52E+01	2.52E+01
ENSG00000054598	FOXC1	0.78	-1.29	-0.36	2.63E-03	-2.51E+01	2.51E+01
ENSG00000162613	FUBP1	0.79	-1.27	-0.35	2.57E-03	-2.51E+01	2.51E+01
ENSG00000168994	PXDC1	1.26	1.26	0.33	2.53E-03	2.51E+01	2.51E+01
	AC009336.24	1.36	1.36	0.44	2.94E-03	2.51E+01	2.51E+01
ENSG00000152284	TCF7L1	1.54	1.54	0.62	3.76E-03	2.51E+01	2.51E+01
ENSG00000117155	SSX2IP	0.77	-1.30	-0.38	2.69E-03	-2.50E+01	2.50E+01
ENSG00000023572	GLRX2	0.75	-1.34	-0.42	2.87E-03	-2.50E+01	2.50E+01
ENSG00000276963	RP11-318K12.1	1.42	1.42	0.51	3.22E-03	2.50E+01	2.50E+01
ENSG00000178397	FAM220A	1.27	1.27	0.34	2.57E-03	2.50E+01	2.50E+01
ENSG00000147894	C9orf72	1.28	1.28	0.35	2.62E-03	2.50E+01	2.50E+01
ENSG00000135940	COX5B	0.78	-1.29	-0.36	2.65E-03	-2.50E+01	2.50E+01
ENSG00000108557	RAI1	1.27	1.27	0.35	2.60E-03	2.49E+01	2.49E+01
ENSG00000175073	VCPIP1	1.34	1.34	0.42	2.88E-03	2.49E+01	2.49E+01
ENSG00000114541	FRMD4B	1.28	1.28	0.35	2.62E-03	2.49E+01	2.49E+01
ENSG00000141034	GID4	1.26	1.26	0.34	2.58E-03	2.49E+01	2.49E+01
ENSG00000142459	EVI5L	1.28	1.28	0.36	2.67E-03	2.48E+01	2.48E+01
ENSG00000058453	CROCC	0.78	-1.28	-0.35	2.64E-03	-2.48E+01	2.48E+01
ENSG00000102921	N4BP1	1.26	1.26	0.34	2.58E-03	2.48E+01	2.48E+01
ENSG00000138658	C4orf21	0.73	-1.36	-0.45	3.03E-03	-2.48E+01	2.48E+01
ENSG00000104341	LAPTM4B	0.79	-1.27	-0.34	2.61E-03	-2.48E+01	2.48E+01
ENSG00000160410	SHKBP1	1.25	1.25	0.32	2.55E-03	2.47E+01	2.47E+01
ENSG00000198901	PRC1	0.78	-1.28	-0.36	2.71E-03	-2.47E+01	2.47E+01
ENSG00000100852	ARHGAP5	1.45	1.45	0.53	3.44E-03	2.47E+01	2.47E+01
ENSG00000197779	ZNF81	1.28	1.28	0.36	2.71E-03	2.47E+01	2.47E+01
ENSG00000197121	PGAP1	0.74	-1.35	-0.43	3.00E-03	-2.46E+01	2.46E+01
ENSG00000134901	KDELC1	0.78	-1.29	-0.36	2.74E-03	-2.46E+01	2.46E+01
ENSG00000138411	HECW2	1.27	1.27	0.34	2.66E-03	2.45E+01	2.45E+01
ENSG00000140471	LINS	0.79	-1.27	-0.34	2.67E-03	-2.45E+01	2.45E+01
ENSG00000123178	SPRYD7	0.76	-1.32	-0.40	2.88E-03	-2.45E+01	2.45E+01
ENSG00000187653	TMSB4XP8	0.74	-1.35	-0.43	3.03E-03	-2.44E+01	2.44E+01
ENSG00000130764	LRRC47	1.25	1.25	0.32	2.62E-03	2.44E+01	2.44E+01
ENSG00000119669	IRF2BPL	1.26	1.26	0.33	2.66E-03	2.44E+01	2.44E+01
ENSG00000121579	NAA50	0.79	-1.27	-0.34	2.70E-03	-2.44E+01	2.44E+01
ENSG00000183935	HTR7P1	1.26	1.26	0.34	2.69E-03	2.44E+01	2.44E+01
ENSG00000169062	UPF3A	0.77	-1.31	-0.39	2.88E-03	-2.43E+01	2.43E+01
ENSG00000233276	GPX1	0.77	-1.29	-0.37	2.83E-03	-2.43E+01	2.43E+01
ENSG00000092853	CLSPN	0.80	-1.25	-0.32	2.64E-03	-2.43E+01	2.43E+01
ENSG00000114850	SSR3	1.29	1.29	0.36	2.80E-03	2.43E+01	2.43E+01
ENSG00000010017	RANBP9	1.25	1.25	0.32	2.65E-03	2.43E+01	2.43E+01
ENSG00000136371	MTHFS	0.62	-1.63	-0.70	4.47E-03	-2.43E+01	2.43E+01
ENSG00000158716	DUSP23	0.80	-1.25	-0.32	2.62E-03	-2.43E+01	2.43E+01
ENSG00000073849	ST6GAL1	0.79	-1.27	-0.35	2.75E-03	-2.43E+01	2.43E+01
	RP11-159F24.1	1.42	1.42	0.51	3.45E-03	2.42E+01	2.42E+01
ENSG00000166387	PPFIBP2	0.66	-1.51	-0.59	3.88E-03	-2.42E+01	2.42E+01
ENSG00000095139	ARCN1	1.27	1.27	0.35	2.75E-03	2.42E+01	2.42E+01
ENSG00000148516	ZEB1	1.40	1.40	0.49	3.36E-03	2.42E+01	2.42E+01
ENSG00000204438	GPANK1	1.28	1.28	0.35	2.79E-03	2.42E+01	2.42E+01
ENSG00000005059	CCDC109B	0.80	-1.26	-0.33	2.71E-03	-2.41E+01	2.41E+01
ENSG00000131236	CAP1	1.27	1.27	0.34	2.77E-03	2.40E+01	2.40E+01
ENSG00000200913	SNORD46	0.62	-1.62	-0.70	4.54E-03	-2.40E+01	2.40E+01
ENSG00000167645	YIF1B	0.80	-1.25	-0.32	2.70E-03	-2.40E+01	2.40E+01
ENSG00000168038	ULK4	1.28	1.28	0.36	2.85E-03	2.40E+01	2.40E+01
ENSG00000166529	ZSCAN21	0.77	-1.29	-0.37	2.91E-03	-2.40E+01	2.40E+01
ENSG00000177683	THAP5	1.29	1.29	0.37	2.90E-03	2.39E+01	2.39E+01
ENSG00000143952	VPS54	0.79	-1.26	-0.33	2.78E-03	-2.39E+01	2.39E+01
ENSG00000189337	KAZN	0.65	-1.53	-0.61	4.10E-03	-2.39E+01	2.39E+01
ENSG00000061676	NCKAP1	1.34	1.34	0.43	3.16E-03	2.39E+01	2.39E+01
ENSG00000159111	MRPL10	1.34	1.34	0.42	3.14E-03	2.38E+01	2.38E+01
ENSG00000100906	NFKBIA	0.78	-1.28	-0.36	2.89E-03	-2.38E+01	2.38E+01
ENSG00000239096	snoU13	1.40	1.40	0.49	3.48E-03	2.38E+01	2.38E+01
ENSG00000197050	ZNF420	1.40	1.40	0.49	3.46E-03	2.38E+01	2.38E+01
ENSG00000170248	PDCD6IP	1.27	1.27	0.35	2.86E-03	2.38E+01	2.38E+01
ENSG00000132471	WBP2	1.38	1.38	0.46	3.36E-03	2.38E+01	2.38E+01

ENSG00000053747	LAMA3	1.32	1.32	0.40	3.07E-03	2.38E+01	2.38E+01
ENSG00000116761	CTH	0.63	-1.60	-0.68	4.54E-03	-2.37E+01	2.37E+01
ENSG00000101347	SAMHD1	0.80	-1.25	-0.33	2.79E-03	-2.37E+01	2.37E+01
ENSG00000182749	PAQR7	0.76	-1.31	-0.39	3.07E-03	-2.37E+01	2.37E+01
ENSG00000119640	ACYP1	0.77	-1.30	-0.37	3.00E-03	-2.37E+01	2.37E+01
ENSG00000133574	GIMAP4	0.78	-1.27	-0.35	2.90E-03	-2.37E+01	2.37E+01
ENSG00000114735	HEMK1	1.26	1.26	0.33	2.83E-03	2.36E+01	2.36E+01
ENSG00000108510	MED13	1.32	1.32	0.40	3.10E-03	2.36E+01	2.36E+01
ENSG00000196455	PIK3R4	1.25	1.25	0.32	2.80E-03	2.36E+01	2.36E+01
ENSG00000162729	IGSF8	1.31	1.31	0.39	3.09E-03	2.36E+01	2.36E+01
ENSG00000135363	LMO2	1.26	1.26	0.33	2.86E-03	2.36E+01	2.36E+01
ENSG00000197951	ZNF71	1.27	1.27	0.35	2.93E-03	2.36E+01	2.36E+01
ENSG00000164151	KIAA0947	1.27	1.27	0.35	2.92E-03	2.35E+01	2.35E+01
ENSG00000008086	CDKL5	1.45	1.45	0.53	3.79E-03	2.35E+01	2.35E+01
	RP11-206L10.11	1.36	1.36	0.44	3.33E-03	2.35E+01	2.35E+01
ENSG00000104129	DNAJC17	0.78	-1.28	-0.36	2.97E-03	-2.35E+01	2.35E+01
ENSG00000239779	WBP1	1.43	1.43	0.51	3.71E-03	2.34E+01	2.34E+01
ENSG00000138443	ABI2	0.79	-1.26	-0.34	2.91E-03	-2.34E+01	2.34E+01
ENSG00000183828	NUDT14	0.78	-1.28	-0.35	2.97E-03	-2.34E+01	2.34E+01
ENSG00000145391	SETD7	1.29	1.29	0.37	3.03E-03	2.34E+01	2.34E+01
ENSG00000198961	PJA2	1.36	1.36	0.45	3.39E-03	2.34E+01	2.34E+01
ENSG00000120533	ENY2	0.78	-1.28	-0.35	2.98E-03	-2.34E+01	2.34E+01
ENSG00000225470	JPX	0.79	-1.26	-0.34	2.91E-03	-2.34E+01	2.34E+01
ENSG00000107551	RASSF4	0.75	-1.34	-0.42	3.26E-03	-2.34E+01	2.34E+01
ENSG00000145623	OSMR	0.79	-1.27	-0.35	2.95E-03	-2.34E+01	2.34E+01
ENSG00000164934	DCAF13	0.78	-1.28	-0.36	3.00E-03	-2.34E+01	2.34E+01
ENSG00000095906	NUBP2	0.79	-1.26	-0.33	2.90E-03	-2.34E+01	2.34E+01
ENSG00000224032	EPB41L4A-AS1	0.76	-1.31	-0.39	3.13E-03	-2.34E+01	2.34E+01
ENSG00000116044	NFE2L2	1.38	1.38	0.47	3.51E-03	2.33E+01	2.33E+01
ENSG00000146143	PRIM2	0.80	-1.24	-0.32	2.84E-03	-2.33E+01	2.33E+01
ENSG00000204632	HLA-G	1.40	1.40	0.48	3.58E-03	2.33E+01	2.33E+01
ENSG00000204261	XXbac-BPG246D15.8	0.68	-1.47	-0.56	4.01E-03	-2.33E+01	2.33E+01
ENSG00000067248	DHX29	1.37	1.37	0.45	3.46E-03	2.33E+01	2.33E+01
ENSG00000163319	MRPS18C	0.78	-1.28	-0.36	3.06E-03	-2.32E+01	2.32E+01
ENSG00000170089	RP11-423H2.1	0.74	-1.35	-0.44	3.40E-03	-2.32E+01	2.32E+01
ENSG00000116260	QSOX1	1.36	1.36	0.44	3.42E-03	2.32E+01	2.32E+01
ENSG00000215386	LINC00478	0.76	-1.32	-0.40	3.22E-03	-2.32E+01	2.32E+01
ENSG00000115207	GTF3C2	0.80	-1.25	-0.32	2.90E-03	-2.32E+01	2.32E+01
ENSG00000156469	MTERFD1	0.80	-1.26	-0.33	2.95E-03	-2.32E+01	2.32E+01
ENSG00000112655	PTK7	0.78	-1.28	-0.35	3.04E-03	-2.31E+01	2.31E+01
ENSG00000169155	ZBTB43	1.27	1.27	0.34	3.00E-03	2.31E+01	2.31E+01
ENSG00000121210	KIAA0922	1.27	1.27	0.35	3.04E-03	2.31E+01	2.31E+01
ENSG00000137497	NUMA1	1.30	1.30	0.38	3.17E-03	2.31E+01	2.31E+01
ENSG00000161638	ITGA5	1.34	1.34	0.43	3.40E-03	2.30E+01	2.30E+01
ENSG00000120656	TAF12	1.25	1.25	0.32	2.93E-03	2.30E+01	2.30E+01
ENSG00000162063	CCNF	0.77	-1.30	-0.38	3.20E-03	-2.30E+01	2.30E+01
ENSG00000173349	SFT2D3	0.77	-1.30	-0.38	3.20E-03	-2.30E+01	2.30E+01
ENSG00000196912	ANKRD36B	1.44	1.44	0.52	3.90E-03	2.30E+01	2.30E+01
	RP11-475N22.4	1.30	1.30	0.38	3.21E-03	2.30E+01	2.30E+01
ENSG00000005436	GCFC2	0.80	-1.25	-0.32	2.96E-03	-2.29E+01	2.29E+01
ENSG00000173221	GLRX	0.78	-1.29	-0.36	3.14E-03	-2.29E+01	2.29E+01
ENSG00000111581	NUP107	0.78	-1.28	-0.36	3.12E-03	-2.29E+01	2.29E+01
ENSG00000175893	ZDHHC21	1.34	1.34	0.43	3.44E-03	2.29E+01	2.29E+01
ENSG00000065183	WDR3	0.79	-1.26	-0.33	3.04E-03	-2.28E+01	2.28E+01
ENSG00000105865	DUS4L	1.31	1.31	0.39	3.30E-03	2.28E+01	2.28E+01
ENSG00000114127	XRN1	1.39	1.39	0.48	3.71E-03	2.28E+01	2.28E+01
ENSG00000241839	PLEKHO2	1.25	1.25	0.32	2.99E-03	2.28E+01	2.28E+01
ENSG00000196526	AFAP1	1.33	1.33	0.42	3.43E-03	2.28E+01	2.28E+01
ENSG00000166477	LEO1	0.79	-1.26	-0.33	3.06E-03	-2.28E+01	2.28E+01
ENSG00000137806	NDUFAF1	0.80	-1.25	-0.33	3.03E-03	-2.28E+01	2.28E+01
ENSG00000125691	RPL23	0.79	-1.27	-0.35	3.12E-03	-2.28E+01	2.28E+01
ENSG00000137845	ADAM10	1.35	1.35	0.44	3.53E-03	2.28E+01	2.28E+01
ENSG00000166704	ZNF606	1.29	1.29	0.37	3.22E-03	2.27E+01	2.27E+01
ENSG00000240429	LRRFIP1P1	1.51	1.51	0.59	4.42E-03	2.27E+01	2.27E+01
ENSG00000166226	CCT2	0.79	-1.26	-0.34	3.12E-03	-2.26E+01	2.26E+01
ENSG00000232063	RP11-307E17.8	1.48	1.48	0.57	4.30E-03	2.26E+01	2.26E+01
ENSG00000188997	KCTD21	1.29	1.29	0.37	3.27E-03	2.26E+01	2.26E+01
ENSG00000137656	BUD13	0.80	-1.24	-0.31	3.04E-03	-2.25E+01	2.25E+01
ENSG00000204463	BAG6	1.32	1.32	0.40	3.41E-03	2.25E+01	2.25E+01
ENSG00000116396	KCNC4	1.26	1.26	0.33	3.12E-03	2.25E+01	2.25E+01
ENSG00000197093	GAL3ST4	1.56	1.56	0.64	4.78E-03	2.25E+01	2.25E+01
ENSG00000108582	CPD	1.26	1.26	0.34	3.18E-03	2.24E+01	2.24E+01
ENSG00000136425	CIB2	0.72	-1.39	-0.48	3.87E-03	-2.24E+01	2.24E+01
ENSG00000171475	WIPF2	0.78	-1.28	-0.35	3.25E-03	-2.24E+01	2.24E+01
ENSG00000148429	USP6NL	1.25	1.25	0.32	3.13E-03	2.24E+01	2.24E+01
ENSG00000212747	FAM127C	1.29	1.29	0.37	3.34E-03	2.23E+01	2.23E+01
ENSG00000140025	EFCAB11	1.30	1.30	0.38	3.42E-03	2.23E+01	2.23E+01
ENSG00000179627	ZBTB42	0.70	-1.43	-0.52	4.13E-03	-2.23E+01	2.23E+01
ENSG00000147383	NSDHL	0.78	-1.29	-0.36	3.35E-03	-2.23E+01	2.23E+01

ENSG00000103932	RPAP1	1.32	1.32	0.40	3.55E-03	2.22E+01	2.22E+01
ENSG00000140950	KIAA1609	1.24	1.24	0.31	3.12E-03	2.22E+01	2.22E+01
ENSG00000162961	DPY30	0.80	-1.25	-0.32	3.17E-03	-2.22E+01	2.22E+01
ENSG00000186073	C15orf41	0.77	-1.30	-0.38	3.44E-03	-2.22E+01	2.22E+01
ENSG00000165672	PRDX3	0.79	-1.26	-0.33	3.24E-03	-2.22E+01	2.22E+01
ENSG00000150347	ARID5B	0.77	-1.29	-0.37	3.43E-03	-2.21E+01	2.21E+01
ENSG00000078124	ACER3	1.26	1.26	0.33	3.25E-03	2.21E+01	2.21E+01
ENSG00000140259	MFAP1	0.81	-1.24	-0.31	3.16E-03	-2.21E+01	2.21E+01
ENSG00000169727	GPS1	0.80	-1.26	-0.33	3.26E-03	-2.20E+01	2.20E+01
ENSG00000100099	HPS4	1.26	1.26	0.33	3.27E-03	2.20E+01	2.20E+01
ENSG00000041802	LSG1	1.27	1.27	0.34	3.31E-03	2.20E+01	2.20E+01
ENSG00000129933	MAU2	1.25	1.25	0.33	3.25E-03	2.20E+01	2.20E+01
ENSG00000116750	UCHL5	0.79	-1.26	-0.34	3.30E-03	-2.20E+01	2.20E+01
ENSG00000175602	CCDC85B	0.77	-1.30	-0.37	3.48E-03	-2.20E+01	2.20E+01
ENSG00000106484	MEST	0.76	-1.31	-0.39	3.56E-03	-2.20E+01	2.20E+01
ENSG00000181610	MRPS23	0.80	-1.24	-0.32	3.22E-03	-2.19E+01	2.19E+01
ENSG00000071537	SEL1L	1.26	1.26	0.33	3.31E-03	2.19E+01	2.19E+01
ENSG00000104728	ARHGEF10	1.25	1.25	0.32	3.25E-03	2.19E+01	2.19E+01
ENSG00000229729	RP11-159G9.5	1.44	1.44	0.52	4.31E-03	2.19E+01	2.19E+01
ENSG00000121289	CEP89	1.27	1.27	0.35	3.38E-03	2.19E+01	2.19E+01
ENSG00000100504	PYGL	1.31	1.31	0.39	3.62E-03	2.18E+01	2.18E+01
ENSG00000118707	TGIF2	1.26	1.26	0.33	3.34E-03	2.18E+01	2.18E+01
ENSG00000113532	ST8SIA4	1.30	1.30	0.38	3.59E-03	2.18E+01	2.18E+01
ENSG00000164512	ANKRD55	0.69	-1.44	-0.53	4.40E-03	-2.18E+01	2.18E+01
ENSG00000068323	TFE3	1.25	1.25	0.33	3.31E-03	2.18E+01	2.18E+01
ENSG00000129680	MAP7D3	1.26	1.26	0.33	3.34E-03	2.18E+01	2.18E+01
ENSG00000163349	HIPK1	1.27	1.27	0.34	3.40E-03	2.17E+01	2.17E+01
ENSG00000198792	TMEM184B	1.26	1.26	0.33	3.36E-03	2.17E+01	2.17E+01
ENSG00000060237	WNK1	1.28	1.28	0.36	3.49E-03	2.17E+01	2.17E+01
ENSG00000187953	PMS2CL	1.29	1.29	0.37	3.56E-03	2.17E+01	2.17E+01
ENSG00000006634	DBF4	0.79	-1.26	-0.34	3.41E-03	-2.16E+01	2.16E+01
ENSG00000116991	SIPA1L2	1.27	1.27	0.34	3.44E-03	2.16E+01	2.16E+01
ENSG00000196712	NF1	1.25	1.25	0.32	3.34E-03	2.16E+01	2.16E+01
ENSG00000261188	CTA-445C9.14	0.63	-1.59	-0.67	5.40E-03	-2.16E+01	2.16E+01
ENSG00000119326	CTNNAL1	0.74	-1.36	-0.44	3.95E-03	-2.16E+01	2.16E+01
ENSG00000109079	TNFAIP1	1.29	1.29	0.36	3.57E-03	2.16E+01	2.16E+01
ENSG00000124380	SNRNP27	0.79	-1.26	-0.33	3.42E-03	-2.16E+01	2.16E+01
ENSG00000006831	ADIPOR2	0.80	-1.25	-0.32	3.40E-03	-2.15E+01	2.15E+01
ENSG00000253982	CTD-2336O2.1	1.28	1.28	0.35	3.52E-03	2.15E+01	2.15E+01
ENSG00000155097	ATP6V1C1	1.26	1.26	0.34	3.45E-03	2.15E+01	2.15E+01
ENSG00000173273	TNKS	1.25	1.25	0.32	3.38E-03	2.15E+01	2.15E+01
ENSG00000103423	DNAJA3	0.80	-1.24	-0.31	3.35E-03	-2.15E+01	2.15E+01
ENSG00000168939	SPRY3	0.68	-1.48	-0.57	4.76E-03	-2.15E+01	2.15E+01
ENSG00000177706	FAM20C	1.27	1.27	0.34	3.50E-03	2.14E+01	2.14E+01
ENSG00000102317	RBM3	1.27	1.27	0.35	3.53E-03	2.14E+01	2.14E+01
	RP11-139H15.1	0.73	-1.37	-0.45	4.09E-03	-2.14E+01	2.14E+01
ENSG00000187257	RSBN1L	0.79	-1.27	-0.34	3.52E-03	-2.14E+01	2.14E+01
	AC007390.5	0.80	-1.26	-0.33	3.47E-03	-2.13E+01	2.13E+01
ENSG00000087076	HSD17B14	0.75	-1.34	-0.42	3.95E-03	-2.13E+01	2.13E+01
ENSG00000147180	ZNF711	0.75	-1.33	-0.42	3.96E-03	-2.12E+01	2.12E+01
ENSG00000166189	HPS6	1.25	1.25	0.32	3.47E-03	2.12E+01	2.12E+01
ENSG00000114779	ABHD14B	0.78	-1.29	-0.36	3.70E-03	-2.12E+01	2.12E+01
ENSG00000225614	ZNF469	1.27	1.27	0.34	3.58E-03	2.11E+01	2.11E+01
ENSG00000143756	FBXO28	1.26	1.26	0.33	3.53E-03	2.11E+01	2.11E+01
ENSG00000181929	PRKAG1	0.80	-1.25	-0.32	3.50E-03	-2.11E+01	2.11E+01
ENSG00000119285	HEATR1	0.79	-1.26	-0.33	3.57E-03	-2.11E+01	2.11E+01
ENSG00000151806	GUF1	1.24	1.24	0.31	3.47E-03	2.11E+01	2.11E+01
ENSG00000106868	SUSD1	0.79	-1.26	-0.33	3.60E-03	-2.10E+01	2.10E+01
ENSG00000175634	RPS6KB2	0.81	-1.24	-0.31	3.51E-03	-2.09E+01	2.09E+01
ENSG00000123104	ITPR2	1.35	1.35	0.43	4.17E-03	2.09E+01	2.09E+01
ENSG00000099804	CDC34	1.25	1.25	0.32	3.56E-03	2.09E+01	2.09E+01
ENSG00000100490	CDKL1	0.74	-1.35	-0.44	4.20E-03	-2.09E+01	2.09E+01
ENSG00000168769	TET2	1.30	1.30	0.37	3.86E-03	2.09E+01	2.09E+01
	RP1-80B9.2	1.48	1.48	0.57	5.05E-03	2.09E+01	2.09E+01
ENSG00000197536	C5orf56	0.70	-1.42	-0.51	4.67E-03	-2.08E+01	2.08E+01
ENSG00000032219	ARID4A	1.32	1.32	0.41	4.05E-03	2.08E+01	2.08E+01
ENSG00000262246	CORO7	1.30	1.30	0.38	3.92E-03	2.08E+01	2.08E+01
ENSG00000159917	ZNF235	1.41	1.41	0.49	4.58E-03	2.08E+01	2.08E+01
ENSG00000207844	AC061975.1	0.63	-1.58	-0.66	5.80E-03	-2.08E+01	2.08E+01
ENSG00000183111	ARHGEF37	1.31	1.31	0.39	3.96E-03	2.07E+01	2.07E+01
ENSG00000083857	FAT1	0.74	-1.35	-0.43	4.24E-03	-2.07E+01	2.07E+01
ENSG00000131899	LLGL1	0.81	-1.24	-0.31	3.58E-03	-2.07E+01	2.07E+01
ENSG00000083937	CHMP2B	1.25	1.25	0.32	3.62E-03	2.07E+01	2.07E+01
ENSG00000168395	ING5	0.80	-1.26	-0.33	3.69E-03	-2.07E+01	2.07E+01
ENSG00000072756	TRNT1	0.80	-1.25	-0.32	3.65E-03	-2.07E+01	2.07E+01
ENSG00000225914	XXbac-BPG154L12.4	1.55	1.55	0.63	5.63E-03	2.07E+01	2.07E+01
ENSG00000251257	CTD-2263F21.1	1.53	1.53	0.61	5.48E-03	2.06E+01	2.06E+01
ENSG00000147813	NAPRT1	0.81	-1.24	-0.31	3.60E-03	-2.06E+01	2.06E+01
ENSG00000169410	PTPN9	1.25	1.25	0.32	3.68E-03	2.06E+01	2.06E+01

ENSG00000095209	TMEM38B	0.78	-1.28	-0.36	3.86E-03	-2.06E+01	2.06E+01
ENSG00000130202	PVRL2	1.29	1.29	0.37	3.94E-03	2.06E+01	2.06E+01
ENSG00000114126	TFDP2	0.81	-1.24	-0.31	3.61E-03	-2.06E+01	2.06E+01
ENSG00000149380	P4HA3	1.25	1.25	0.33	3.73E-03	2.05E+01	2.05E+01
ENSG00000177169	ULK1	1.29	1.29	0.37	3.94E-03	2.05E+01	2.05E+01
ENSG00000175106	FAM18B2	0.71	-1.40	-0.49	4.66E-03	-2.05E+01	2.05E+01
ENSG00000164105	SAP30	0.81	-1.24	-0.31	3.66E-03	-2.05E+01	2.05E+01
ENSG00000001084	GCLC	0.81	-1.24	-0.31	3.64E-03	-2.05E+01	2.05E+01
	FLJ14082	1.44	1.44	0.53	4.94E-03	2.05E+01	2.05E+01
ENSG00000099341	PSMD8	0.80	-1.25	-0.33	3.74E-03	-2.05E+01	2.05E+01
ENSG00000181638	ZFP41	1.35	1.35	0.43	4.34E-03	2.05E+01	2.05E+01
ENSG00000164989	CCDC171	1.39	1.39	0.48	4.62E-03	2.05E+01	2.05E+01
ENSG00000189298	ZKSCAN3	1.33	1.33	0.41	4.21E-03	2.05E+01	2.05E+01
ENSG00000102900	NUP93	0.79	-1.26	-0.33	3.79E-03	-2.05E+01	2.05E+01
ENSG00000111490	TBC1D30	0.79	-1.27	-0.34	3.82E-03	-2.05E+01	2.05E+01
ENSG00000178105	DDX10	0.80	-1.25	-0.32	3.74E-03	-2.05E+01	2.05E+01
ENSG00000166788	SAAL1	0.81	-1.24	-0.31	3.65E-03	-2.05E+01	2.05E+01
ENSG00000090470	PDCD7	0.79	-1.26	-0.33	3.78E-03	-2.05E+01	2.05E+01
ENSG00000176101	SSNA1	0.79	-1.26	-0.33	3.79E-03	-2.05E+01	2.05E+01
ENSG00000170340	B3GNT2	0.80	-1.24	-0.31	3.70E-03	-2.05E+01	2.05E+01
ENSG00000159792	PSKH1	0.78	-1.28	-0.36	3.95E-03	-2.04E+01	2.04E+01
ENSG00000172053	QARS	0.79	-1.26	-0.33	3.80E-03	-2.04E+01	2.04E+01
ENSG00000115084	SLC35F5	1.25	1.25	0.32	3.75E-03	2.04E+01	2.04E+01
ENSG00000118482	PHF3	1.45	1.45	0.54	5.10E-03	2.04E+01	2.04E+01
ENSG00000132613	MTSS1L	1.26	1.26	0.33	3.80E-03	2.04E+01	2.04E+01
ENSG00000078070	MCCC1	0.80	-1.24	-0.32	3.74E-03	-2.04E+01	2.04E+01
ENSG00000253190	AC084082.3	0.62	-1.61	-0.69	6.25E-03	-2.03E+01	2.03E+01
ENSG00000167912	RP11-25K19.1	0.74	-1.36	-0.44	4.48E-03	-2.03E+01	2.03E+01
ENSG00000109066	TMEM104	1.27	1.27	0.34	3.89E-03	2.03E+01	2.03E+01
ENSG00000067064	IDI1	0.79	-1.26	-0.34	3.89E-03	-2.03E+01	2.03E+01
ENSG00000176826	FKBP9L	1.45	1.45	0.54	5.13E-03	2.03E+01	2.03E+01
ENSG00000198363	ASPH	1.26	1.26	0.33	3.85E-03	2.03E+01	2.03E+01
ENSG00000109265	KIAA1211	1.34	1.34	0.43	4.41E-03	2.02E+01	2.02E+01
ENSG00000146701	MDH2	1.28	1.28	0.35	3.99E-03	2.02E+01	2.02E+01
ENSG00000131469	RPL27	0.77	-1.30	-0.38	4.18E-03	-2.02E+01	2.02E+01
ENSG00000125375	ATP5S	0.75	-1.33	-0.41	4.31E-03	-2.02E+01	2.02E+01
ENSG00000275835	TUBGCP5	0.81	-1.24	-0.31	3.78E-03	-2.02E+01	2.02E+01
ENSG00000083750	RRAGB	1.27	1.27	0.34	3.96E-03	2.01E+01	2.01E+01
ENSG00000213995	CARKD	0.80	-1.25	-0.32	3.84E-03	-2.01E+01	2.01E+01
ENSG00000147010	SH3KBP1	0.81	-1.24	-0.31	3.78E-03	-2.01E+01	2.01E+01
ENSG00000146802	TMEM168	0.78	-1.28	-0.35	4.03E-03	-2.01E+01	2.01E+01
ENSG00000165802	NELF	1.23	1.23	0.30	3.78E-03	2.01E+01	2.01E+01
ENSG00000108639	SYNGR2	1.25	1.25	0.32	3.86E-03	2.01E+01	2.01E+01
ENSG00000158796	DEDD	0.81	-1.24	-0.31	3.80E-03	-2.01E+01	2.01E+01
ENSG00000102977	ACD	0.79	-1.26	-0.33	3.95E-03	-2.01E+01	2.01E+01
ENSG00000074201	CLNS1A	0.80	-1.25	-0.33	3.92E-03	-2.00E+01	2.00E+01
ENSG00000131504	DIAPH1	0.79	-1.27	-0.34	4.01E-03	-2.00E+01	2.00E+01
ENSG00000141858	SAMD1	0.79	-1.26	-0.33	3.96E-03	-2.00E+01	2.00E+01
ENSG00000172123	SLFN12	0.80	-1.25	-0.32	3.90E-03	-2.00E+01	2.00E+01
ENSG00000149809	TM7SF2	0.63	-1.60	-0.68	6.41E-03	-2.00E+01	2.00E+01
ENSG00000162076	FLYWCH2	0.80	-1.25	-0.33	3.96E-03	-1.99E+01	1.99E+01
ENSG00000066117	SMARCD1	1.28	1.28	0.36	4.15E-03	1.99E+01	1.99E+01
ENSG00000104529	EEF1D	0.80	-1.25	-0.33	3.98E-03	-1.99E+01	1.99E+01
ENSG00000196209	SIRPB2	0.67	-1.48	-0.57	5.56E-03	-1.99E+01	1.99E+01
ENSG00000156970	BUB1B	0.78	-1.28	-0.36	4.17E-03	-1.98E+01	1.98E+01
ENSG00000169519	METTL15	0.80	-1.26	-0.33	4.05E-03	-1.97E+01	1.97E+01
ENSG00000144401	METTL21A	0.78	-1.28	-0.35	4.19E-03	-1.97E+01	1.97E+01
ENSG00000184481	FOXO4	0.75	-1.33	-0.41	4.60E-03	-1.96E+01	1.96E+01
ENSG00000197345	MRPL21	0.80	-1.25	-0.32	4.09E-03	-1.96E+01	1.96E+01
ENSG00000265666	CTD-2267D19.2	0.70	-1.44	-0.52	5.43E-03	-1.95E+01	1.95E+01
ENSG00000226871	AC135178.1	1.39	1.39	0.47	5.05E-03	1.95E+01	1.95E+01
ENSG00000129007	CALML4	0.75	-1.34	-0.42	4.73E-03	-1.95E+01	1.95E+01
ENSG00000140395	WDR61	0.80	-1.24	-0.31	4.07E-03	-1.95E+01	1.95E+01
ENSG00000149089	APIP	0.81	-1.24	-0.31	4.07E-03	-1.95E+01	1.95E+01
ENSG00000163938	GNL3	0.80	-1.26	-0.33	4.19E-03	-1.94E+01	1.94E+01
ENSG00000115841	FAM82A1	1.37	1.37	0.46	4.98E-03	1.94E+01	1.94E+01
ENSG00000178951	ZBTB7A	1.34	1.34	0.42	4.75E-03	1.94E+01	1.94E+01
ENSG00000065970	FOXJ2	1.36	1.36	0.44	4.92E-03	1.94E+01	1.94E+01
ENSG00000169239	CA5B	0.77	-1.30	-0.38	4.53E-03	-1.93E+01	1.93E+01
ENSG00000175746	C15orf54	1.40	1.40	0.49	5.27E-03	1.93E+01	1.93E+01
ENSG00000075336	TIMM21	0.81	-1.24	-0.31	4.10E-03	-1.93E+01	1.93E+01
ENSG00000162512	SDC3	1.26	1.26	0.33	4.26E-03	1.93E+01	1.93E+01
ENSG00000076650	GPATCH1	0.78	-1.28	-0.35	4.40E-03	-1.93E+01	1.93E+01
ENSG00000164815	ORC5	1.24	1.24	0.31	4.14E-03	1.93E+01	1.93E+01
ENSG00000197808	ZNF461	1.31	1.31	0.39	4.67E-03	1.92E+01	1.92E+01
ENSG00000137804	NUSAP1	0.77	-1.29	-0.37	4.53E-03	-1.92E+01	1.92E+01
ENSG00000273611	ZNHIT3	0.80	-1.25	-0.33	4.25E-03	-1.92E+01	1.92E+01
ENSG00000147457	CHMP7	0.81	-1.24	-0.31	4.16E-03	-1.92E+01	1.92E+01
ENSG00000254986	DPP3	1.23	1.23	0.30	4.12E-03	1.92E+01	1.92E+01

ENSG00000110921	MVK	0.81	-1.24	-0.31	4.18E-03	-1.92E+01	1.92E+01
ENSG00000154380	ENAH	0.80	-1.25	-0.32	4.26E-03	-1.92E+01	1.92E+01
ENSG00000104472	CHRAC1	0.79	-1.26	-0.34	4.34E-03	-1.92E+01	1.92E+01
ENSG00000213588	ZBTB9	0.78	-1.27	-0.35	4.45E-03	-1.91E+01	1.91E+01
ENSG00000178585	CTNNBIP1	1.24	1.24	0.31	4.19E-03	1.91E+01	1.91E+01
ENSG00000138834	MAPK8IP3	1.23	1.23	0.30	4.17E-03	1.91E+01	1.91E+01
ENSG00000198542	ITGBL1	1.31	1.31	0.39	4.75E-03	1.91E+01	1.91E+01
ENSG00000147854	UHRF2	0.80	-1.26	-0.33	4.34E-03	-1.91E+01	1.91E+01
ENSG00000158560	DYNC1I1	0.67	-1.49	-0.58	6.11E-03	-1.91E+01	1.91E+01
ENSG00000170946	DNAJC24	0.79	-1.26	-0.33	4.36E-03	-1.90E+01	1.90E+01
ENSG00000105339	DENND3	1.24	1.24	0.31	4.24E-03	1.90E+01	1.90E+01
ENSG00000168003	SLC3A2	1.26	1.26	0.33	4.35E-03	1.90E+01	1.90E+01
ENSG00000132612	VPS4A	0.80	-1.24	-0.31	4.28E-03	-1.90E+01	1.90E+01
ENSG00000167693	NXN	0.81	-1.24	-0.31	4.25E-03	-1.90E+01	1.90E+01
ENSG00000162650	ATXN7L2	0.68	-1.47	-0.55	5.97E-03	-1.90E+01	1.90E+01
ENSG00000102098	SCML2	0.72	-1.40	-0.48	5.42E-03	-1.90E+01	1.90E+01
ENSG00000155561	NUP205	0.79	-1.26	-0.33	4.42E-03	-1.89E+01	1.89E+01
ENSG00000142156	COL6A1	1.36	1.36	0.45	5.19E-03	1.89E+01	1.89E+01
ENSG00000204186	ZDBF2	1.39	1.39	0.47	5.38E-03	1.89E+01	1.89E+01
ENSG00000229117	RPL41	0.79	-1.27	-0.35	4.55E-03	-1.89E+01	1.89E+01
ENSG00000114166	KAT2B	1.24	1.24	0.31	4.33E-03	1.89E+01	1.89E+01
ENSG00000213741	RPS29	0.73	-1.36	-0.45	5.22E-03	-1.89E+01	1.89E+01
ENSG00000196510	ANAPC7	0.80	-1.24	-0.32	4.35E-03	-1.89E+01	1.89E+01
ENSG00000140563	MCTP2	0.68	-1.47	-0.56	6.12E-03	-1.88E+01	1.88E+01
ENSG00000099139	PCSK5	1.29	1.29	0.37	4.70E-03	1.88E+01	1.88E+01
ENSG00000120837	NFYB	1.25	1.25	0.33	4.46E-03	1.88E+01	1.88E+01
ENSG00000225091	SNORA71A	0.65	-1.54	-0.63	6.76E-03	-1.88E+01	1.88E+01
ENSG00000071539	TRIP13	0.80	-1.25	-0.33	4.47E-03	-1.88E+01	1.88E+01
ENSG00000127329	PTPRB	1.27	1.27	0.34	4.59E-03	1.87E+01	1.87E+01
ENSG00000204344	STK19	1.30	1.30	0.37	4.79E-03	1.87E+01	1.87E+01
ENSG00000198642	KLHL9	1.26	1.26	0.33	4.54E-03	1.87E+01	1.87E+01
ENSG00000178718	RPP25	1.24	1.24	0.31	4.42E-03	1.87E+01	1.87E+01
ENSG00000196505	GDAP2	1.24	1.24	0.31	4.38E-03	1.87E+01	1.87E+01
ENSG00000174099	MSRB3	0.80	-1.25	-0.32	4.45E-03	-1.87E+01	1.87E+01
ENSG00000185477	GPRIN3	1.37	1.37	0.46	5.42E-03	1.87E+01	1.87E+01
ENSG00000198431	TXNRD1	1.26	1.26	0.33	4.54E-03	1.87E+01	1.87E+01
ENSG00000174606	ANGEL2	1.24	1.24	0.31	4.40E-03	1.87E+01	1.87E+01
ENSG00000121851	POLR3GL	0.80	-1.26	-0.33	4.53E-03	-1.86E+01	1.86E+01
ENSG00000233927	RPS28	0.76	-1.32	-0.40	5.01E-03	-1.86E+01	1.86E+01
ENSG00000166847	DCTN5	1.25	1.25	0.32	4.47E-03	1.86E+01	1.86E+01
ENSG00000067560	RHOA	1.25	1.25	0.32	4.53E-03	1.86E+01	1.86E+01
ENSG00000163659	TIPARP	1.26	1.26	0.33	4.56E-03	1.86E+01	1.86E+01
ENSG00000066777	ARFGEF1	1.33	1.33	0.41	5.13E-03	1.86E+01	1.86E+01
ENSG00000112701	SENP6	1.27	1.27	0.35	4.71E-03	1.86E+01	1.86E+01
ENSG00000072042	RDH11	0.80	-1.25	-0.32	4.56E-03	-1.85E+01	1.85E+01
ENSG00000082258	CCNT2	1.29	1.29	0.36	4.83E-03	1.85E+01	1.85E+01
ENSG00000188629	ZNF177	1.49	1.49	0.57	6.47E-03	1.85E+01	1.85E+01
ENSG00000175782	SLC35E3	1.24	1.24	0.31	4.50E-03	1.85E+01	1.85E+01
ENSG00000125449	ARMC7	1.29	1.29	0.36	4.86E-03	1.84E+01	1.84E+01
ENSG00000113013	HSPA9	0.80	-1.25	-0.32	4.60E-03	-1.84E+01	1.84E+01
ENSG00000010810	FYN	1.25	1.25	0.32	4.60E-03	1.84E+01	1.84E+01
ENSG00000040633	PHF23	0.81	-1.24	-0.31	4.51E-03	-1.84E+01	1.84E+01
ENSG00000274211	SOCS7	1.32	1.32	0.40	5.17E-03	1.84E+01	1.84E+01
ENSG00000180822	PSMG4	0.81	-1.24	-0.31	4.55E-03	-1.84E+01	1.84E+01
ENSG00000084072	PPIE	0.79	-1.27	-0.35	4.84E-03	-1.83E+01	1.83E+01
ENSG00000139620	KANSL2	0.81	-1.23	-0.30	4.52E-03	-1.83E+01	1.83E+01
ENSG00000198728	LDB1	1.24	1.24	0.31	4.59E-03	1.83E+01	1.83E+01
ENSG00000161996	WDR90	0.78	-1.28	-0.36	4.92E-03	-1.83E+01	1.83E+01
ENSG00000002822	MAD1L1	0.80	-1.26	-0.33	4.75E-03	-1.83E+01	1.83E+01
ENSG00000258399	MEG8	1.32	1.32	0.40	5.28E-03	1.82E+01	1.82E+01
ENSG00000198793	MTOR	1.28	1.28	0.36	4.95E-03	1.82E+01	1.82E+01
ENSG00000180423	HARBI1	1.38	1.38	0.46	5.75E-03	1.82E+01	1.82E+01
ENSG00000151718	WWC2	1.26	1.26	0.34	4.84E-03	1.82E+01	1.82E+01
ENSG00000143363	PRUNE	1.28	1.28	0.35	4.95E-03	1.81E+01	1.81E+01
ENSG00000131149	GSE1	1.38	1.38	0.47	5.82E-03	1.81E+01	1.81E+01
ENSG00000115760	BIRC6	1.32	1.32	0.40	5.28E-03	1.81E+01	1.81E+01
ENSG00000137040	RANBP6	1.35	1.35	0.44	5.60E-03	1.81E+01	1.81E+01
ENSG00000196268	ZNF493	1.29	1.29	0.36	5.08E-03	1.80E+01	1.80E+01
ENSG00000168672	FAM84B	0.81	-1.24	-0.31	4.74E-03	-1.80E+01	1.80E+01
ENSG00000035141	FAM136A	0.80	-1.24	-0.31	4.75E-03	-1.80E+01	1.80E+01
ENSG00000037280	FLT4	0.79	-1.26	-0.34	4.90E-03	-1.80E+01	1.80E+01
ENSG00000185658	BRWD1	1.36	1.36	0.44	5.68E-03	1.80E+01	1.80E+01
ENSG00000144395	CCDC150	0.75	-1.33	-0.41	5.44E-03	-1.80E+01	1.80E+01
ENSG00000144320	KIAA1715	1.25	1.25	0.33	4.86E-03	1.80E+01	1.80E+01
ENSG00000196177	ACADSB	1.26	1.26	0.33	4.89E-03	1.80E+01	1.80E+01
	RP11-429G19.2	0.75	-1.33	-0.41	5.47E-03	-1.79E+01	1.79E+01
ENSG00000068796	KIF2A	1.34	1.34	0.42	5.54E-03	1.79E+01	1.79E+01
ENSG00000215717	TMEM167B	0.80	-1.24	-0.32	4.81E-03	-1.79E+01	1.79E+01
ENSG00000105447	GRWD1	0.81	-1.23	-0.30	4.74E-03	-1.79E+01	1.79E+01

ENSG00000196741	CXorf24	1.46	1.46	0.54	6.61E-03	1.79E+01	1.79E+01
ENSG00000108349	CASC3	0.80	-1.25	-0.32	4.88E-03	-1.79E+01	1.79E+01
ENSG00000166140	ZFYVE19	1.25	1.25	0.32	4.88E-03	1.79E+01	1.79E+01
ENSG00000103852	TTC23	0.81	-1.23	-0.30	4.74E-03	-1.78E+01	1.78E+01
ENSG00000087884	AAMDC	0.80	-1.25	-0.32	4.88E-03	-1.78E+01	1.78E+01
ENSG00000221978	CCNL2	0.80	-1.26	-0.33	4.98E-03	-1.78E+01	1.78E+01
ENSG00000138760	SCARB2	1.26	1.26	0.33	4.99E-03	1.78E+01	1.78E+01
ENSG00000213066	FGFR1OP	0.81	-1.24	-0.31	4.86E-03	-1.77E+01	1.77E+01
ENSG00000142089	IFITM3	0.80	-1.25	-0.32	4.96E-03	-1.77E+01	1.77E+01
ENSG00000059573	ALDH18A1	0.80	-1.25	-0.32	4.94E-03	-1.77E+01	1.77E+01
ENSG00000198718	FAM179B	1.27	1.27	0.35	5.15E-03	1.77E+01	1.77E+01
ENSG00000100359	SGSM3	0.81	-1.23	-0.30	4.87E-03	-1.77E+01	1.77E+01
ENSG00000166024	R3HCC1L	1.28	1.28	0.35	5.21E-03	1.77E+01	1.77E+01
ENSG00000151532	VTI1A	1.23	1.23	0.30	4.87E-03	1.77E+01	1.77E+01
ENSG00000113194	FAF2	1.24	1.24	0.31	4.95E-03	1.77E+01	1.77E+01
ENSG00000150687	PRSS23	1.27	1.27	0.34	5.18E-03	1.76E+01	1.76E+01
ENSG00000226479	TMEM185B	1.41	1.41	0.49	6.42E-03	1.76E+01	1.76E+01
ENSG00000178917	ZNF852	0.73	-1.37	-0.45	6.11E-03	-1.75E+01	1.75E+01
ENSG00000105993	DNAJB6	1.25	1.25	0.32	5.05E-03	1.75E+01	1.75E+01
ENSG00000110442	COMMD9	1.23	1.23	0.30	4.94E-03	1.75E+01	1.75E+01
ENSG00000256667	KLRAP1	1.47	1.47	0.56	7.08E-03	1.75E+01	1.75E+01
ENSG00000128274	A4GALT	1.31	1.31	0.39	5.63E-03	1.75E+01	1.75E+01
ENSG00000155868	MED7	0.80	-1.25	-0.32	5.14E-03	-1.75E+01	1.75E+01
ENSG00000080824	HSP90AA1	1.30	1.30	0.38	5.57E-03	1.75E+01	1.75E+01
ENSG00000061273	HDAC7	1.25	1.25	0.32	5.14E-03	1.75E+01	1.75E+01
ENSG00000158411	MITD1	0.81	-1.23	-0.30	4.99E-03	-1.74E+01	1.74E+01
ENSG00000182858	ALG12	1.26	1.26	0.34	5.25E-03	1.74E+01	1.74E+01
ENSG00000099260	PALMD	1.26	1.26	0.33	5.20E-03	1.74E+01	1.74E+01
ENSG00000156795	WDYHV1	0.78	-1.28	-0.36	5.43E-03	-1.74E+01	1.74E+01
ENSG00000117602	RCAN3	0.66	-1.51	-0.60	7.58E-03	-1.74E+01	1.74E+01
ENSG00000119682	KIAA0317	1.26	1.26	0.33	5.25E-03	1.74E+01	1.74E+01
ENSG00000035681	NSMAF	0.81	-1.24	-0.31	5.12E-03	-1.74E+01	1.74E+01
ENSG00000103148	NPRL3	0.75	-1.33	-0.41	5.83E-03	-1.74E+01	1.74E+01
ENSG00000112096	SOD2	1.25	1.25	0.32	5.18E-03	1.73E+01	1.73E+01
ENSG00000048707	VPS13D	1.31	1.31	0.39	5.74E-03	1.73E+01	1.73E+01
ENSG00000139644	TMBIM6	1.26	1.26	0.33	5.31E-03	1.73E+01	1.73E+01
ENSG00000174483	BBS1	1.27	1.27	0.34	5.38E-03	1.73E+01	1.73E+01
ENSG00000105618	PRPF31	0.80	-1.24	-0.32	5.19E-03	-1.73E+01	1.73E+01
ENSG00000146540	C7orf50	0.81	-1.23	-0.30	5.07E-03	-1.73E+01	1.73E+01
ENSG00000213398	LCAT	1.42	1.42	0.50	6.75E-03	1.72E+01	1.72E+01
ENSG00000179950	PUF60	0.80	-1.25	-0.32	5.28E-03	-1.72E+01	1.72E+01
ENSG00000105576	TNPO2	0.79	-1.27	-0.35	5.50E-03	-1.72E+01	1.72E+01
ENSG00000144306	SCRN3	1.24	1.24	0.31	5.23E-03	1.72E+01	1.72E+01
ENSG00000061794	MRPS35	0.80	-1.25	-0.32	5.27E-03	-1.72E+01	1.72E+01
ENSG00000268573	RP11-158H5.7	0.70	-1.42	-0.51	6.89E-03	-1.71E+01	1.71E+01
ENSG00000143374	TARS2	0.79	-1.27	-0.35	5.53E-03	-1.71E+01	1.71E+01
ENSG00000127452	FBXL12	1.26	1.26	0.33	5.41E-03	1.71E+01	1.71E+01
ENSG00000176386	CDC26	0.79	-1.26	-0.33	5.44E-03	-1.71E+01	1.71E+01
ENSG00000178234	GALNT11	0.81	-1.23	-0.30	5.20E-03	-1.71E+01	1.71E+01
ENSG00000108848	LUC7L3	0.79	-1.27	-0.35	5.56E-03	-1.71E+01	1.71E+01
ENSG00000069329	VPS35	1.25	1.25	0.32	5.33E-03	1.71E+01	1.71E+01
ENSG00000151689	INPP1	1.24	1.24	0.31	5.26E-03	1.71E+01	1.71E+01
ENSG00000117408	IPO13	1.27	1.27	0.35	5.56E-03	1.71E+01	1.71E+01
ENSG00000196670	ZFP62	1.24	1.24	0.31	5.29E-03	1.70E+01	1.70E+01
ENSG00000133706	LARS	0.80	-1.24	-0.32	5.35E-03	-1.70E+01	1.70E+01
ENSG00000109586	GALNT7	0.79	-1.26	-0.34	5.54E-03	-1.70E+01	1.70E+01
ENSG00000108799	EZH1	1.25	1.25	0.32	5.41E-03	1.69E+01	1.69E+01
ENSG00000175505	CLCF1	1.42	1.42	0.51	7.03E-03	1.69E+01	1.69E+01
ENSG00000162065	TBC1D24	0.81	-1.24	-0.31	5.37E-03	-1.69E+01	1.69E+01
ENSG00000168077	SCARA3	0.80	-1.26	-0.33	5.53E-03	-1.69E+01	1.69E+01
ENSG00000101473	ACOT8	0.79	-1.27	-0.34	5.64E-03	-1.68E+01	1.68E+01
ENSG00000138468	SENP7	1.31	1.31	0.39	6.04E-03	1.68E+01	1.68E+01
ENSG00000213906	LTB4R2	1.46	1.46	0.55	7.54E-03	1.68E+01	1.68E+01
ENSG00000139405	C12orf52	0.80	-1.25	-0.32	5.54E-03	-1.68E+01	1.68E+01
ENSG00000110104	CCDC86	1.38	1.38	0.46	6.71E-03	1.68E+01	1.68E+01
ENSG00000100599	RIN3	1.46	1.46	0.54	7.54E-03	1.68E+01	1.68E+01
ENSG00000172167	MTBP	0.78	-1.28	-0.36	5.82E-03	-1.68E+01	1.68E+01
ENSG00000116990	MYCL1	0.70	-1.43	-0.51	7.23E-03	-1.68E+01	1.68E+01
ENSG00000170006	TMEM154	0.80	-1.26	-0.33	5.61E-03	-1.68E+01	1.68E+01
ENSG00000005175	RPAP3	0.78	-1.28	-0.36	5.88E-03	-1.67E+01	1.67E+01
ENSG00000108961	RANGRF	0.80	-1.26	-0.33	5.65E-03	-1.67E+01	1.67E+01
ENSG00000133316	WDR74	1.23	1.23	0.29	5.38E-03	1.67E+01	1.67E+01
ENSG00000110395	CBL	1.27	1.27	0.35	5.81E-03	1.67E+01	1.67E+01
ENSG00000160888	IER2	1.26	1.26	0.33	5.66E-03	1.67E+01	1.67E+01
ENSG00000197256	KANK2	1.25	1.25	0.32	5.63E-03	1.67E+01	1.67E+01
ENSG00000203995	ZYG11A	0.75	-1.33	-0.41	6.39E-03	-1.67E+01	1.67E+01
ENSG00000134955	SLC37A2	0.68	-1.48	-0.57	7.94E-03	-1.66E+01	1.66E+01
ENSG00000127124	HIVEP3	1.35	1.35	0.43	6.63E-03	1.66E+01	1.66E+01
ENSG00000155158	TTC39B	0.75	-1.34	-0.42	6.49E-03	-1.66E+01	1.66E+01

ENSG00000115977	AAK1	1.27	1.27	0.35	5.87E-03	1.66E+01	1.66E+01
ENSG00000104142	VPS18	1.23	1.23	0.30	5.53E-03	1.65E+01	1.65E+01
ENSG00000158882	TOMM40L	0.81	-1.23	-0.30	5.55E-03	-1.65E+01	1.65E+01
ENSG00000013306	SLC25A39	0.81	-1.24	-0.31	5.66E-03	-1.65E+01	1.65E+01
ENSG00000185115	NDNL2	1.23	1.23	0.30	5.55E-03	1.65E+01	1.65E+01
ENSG00000173421	CCDC36	0.75	-1.34	-0.42	6.62E-03	-1.65E+01	1.65E+01
ENSG00000049246	PER3	0.69	-1.46	-0.54	7.83E-03	-1.65E+01	1.65E+01
ENSG00000133030	MPRIP	1.25	1.25	0.32	5.81E-03	1.64E+01	1.64E+01
ENSG00000176058	TPRN	0.79	-1.27	-0.34	5.99E-03	-1.64E+01	1.64E+01
ENSG00000135540	NHSL1	1.25	1.25	0.33	5.86E-03	1.64E+01	1.64E+01
ENSG00000150054	MPP7	1.62	1.62	0.70	9.82E-03	1.64E+01	1.64E+01
ENSG00000267520	RP11-373L24.1	1.57	1.57	0.65	9.28E-03	1.63E+01	1.63E+01
ENSG00000103222	ABCC1	1.25	1.25	0.33	5.95E-03	1.63E+01	1.63E+01
ENSG00000151702	FLI1	0.81	-1.24	-0.31	5.84E-03	-1.63E+01	1.63E+01
ENSG00000259583	RP11-66B24.4	1.33	1.33	0.42	6.73E-03	1.63E+01	1.63E+01
	AC016683.6	1.37	1.37	0.45	7.11E-03	1.62E+01	1.62E+01
ENSG00000167302	ENTHD2	1.29	1.29	0.36	6.29E-03	1.62E+01	1.62E+01
ENSG00000013364	MVP	1.30	1.30	0.38	6.43E-03	1.62E+01	1.62E+01
ENSG00000015479	MATR3	1.31	1.31	0.38	6.50E-03	1.62E+01	1.62E+01
ENSG00000137198	GMPR	1.24	1.24	0.31	5.83E-03	1.62E+01	1.62E+01
ENSG00000137166	FOXP4	1.37	1.37	0.45	7.16E-03	1.62E+01	1.62E+01
ENSG00000106330	MOSPD3	1.32	1.32	0.40	6.74E-03	1.61E+01	1.61E+01
ENSG00000164111	ANXA5	1.24	1.24	0.32	5.98E-03	1.61E+01	1.61E+01
ENSG00000184584	TMEM173	1.26	1.26	0.33	6.11E-03	1.61E+01	1.61E+01
ENSG00000133740	E2F5	0.79	-1.27	-0.35	6.28E-03	-1.61E+01	1.61E+01
ENSG00000124942	AHNAK	1.28	1.28	0.35	6.33E-03	1.61E+01	1.61E+01
ENSG00000065135	GNAI3	0.80	-1.25	-0.32	6.02E-03	-1.60E+01	1.60E+01
ENSG00000092445	TYRO3	0.81	-1.24	-0.31	5.99E-03	-1.60E+01	1.60E+01
ENSG00000065029	ZNF76	1.26	1.26	0.34	6.21E-03	1.60E+01	1.60E+01
ENSG00000182287	AP1S2	0.80	-1.25	-0.32	6.09E-03	-1.60E+01	1.60E+01
ENSG00000197043	ANXA6	0.80	-1.25	-0.32	6.09E-03	-1.60E+01	1.60E+01
ENSG00000135390	ATP5G2	0.80	-1.26	-0.33	6.20E-03	-1.60E+01	1.60E+01
ENSG00000160563	MED27	1.27	1.27	0.35	6.37E-03	1.60E+01	1.60E+01
ENSG00000078596	ITM2A	1.53	1.53	0.61	9.14E-03	1.60E+01	1.60E+01
ENSG00000185716	C16orf52	0.81	-1.23	-0.30	5.99E-03	-1.60E+01	1.60E+01
ENSG00000148481	FAM188A	0.82	-1.22	-0.29	5.90E-03	-1.59E+01	1.59E+01
ENSG00000160877	NACC1	1.27	1.27	0.34	6.33E-03	1.59E+01	1.59E+01
ENSG00000132819	RBM38	1.25	1.25	0.32	6.13E-03	1.59E+01	1.59E+01
ENSG00000173599	PC	0.77	-1.30	-0.38	6.72E-03	-1.59E+01	1.59E+01
ENSG00000198730	CTR9	1.23	1.23	0.30	5.98E-03	1.59E+01	1.59E+01
ENSG00000215251	FASTKD5	1.23	1.23	0.30	6.01E-03	1.59E+01	1.59E+01
ENSG00000151835	SACS	1.49	1.49	0.58	8.86E-03	1.59E+01	1.59E+01
ENSG00000160326	SLC2A6	0.81	-1.23	-0.30	6.03E-03	-1.59E+01	1.59E+01
ENSG00000143878	RHOB	1.27	1.27	0.35	6.43E-03	1.58E+01	1.58E+01
ENSG00000124074	ENKD1	0.79	-1.26	-0.34	6.40E-03	-1.58E+01	1.58E+01
ENSG00000111860	CEP85L	1.31	1.31	0.38	6.83E-03	1.58E+01	1.58E+01
ENSG00000100417	PMM1	0.75	-1.34	-0.42	7.25E-03	-1.58E+01	1.58E+01
ENSG00000197780	TAF13	0.76	-1.31	-0.39	6.96E-03	-1.57E+01	1.57E+01
ENSG00000115641	FHL2	1.24	1.24	0.32	6.26E-03	1.57E+01	1.57E+01
ENSG00000186654	PRR5	0.70	-1.44	-0.52	8.35E-03	-1.57E+01	1.57E+01
ENSG00000074657	ZNF532	0.81	-1.23	-0.30	6.13E-03	-1.57E+01	1.57E+01
ENSG00000166348	USP54	1.26	1.26	0.34	6.51E-03	1.57E+01	1.57E+01
ENSG00000135677	GNS	1.24	1.24	0.32	6.31E-03	1.57E+01	1.57E+01
ENSG00000085998	POMGNT1	1.23	1.23	0.30	6.20E-03	1.56E+01	1.56E+01
ENSG00000185070	FLRT2	0.80	-1.24	-0.32	6.33E-03	-1.56E+01	1.56E+01
ENSG00000267575	CTC-459F4.3	0.76	-1.32	-0.40	7.10E-03	-1.56E+01	1.56E+01
ENSG00000143578	CREB3L4	1.27	1.27	0.35	6.64E-03	1.56E+01	1.56E+01
ENSG00000151576	QTRTD1	0.81	-1.24	-0.31	6.33E-03	-1.56E+01	1.56E+01
ENSG00000158941	KIAA1967	0.79	-1.26	-0.33	6.53E-03	-1.56E+01	1.56E+01
ENSG00000142409	ZNF787	0.81	-1.23	-0.30	6.25E-03	-1.56E+01	1.56E+01
ENSG00000151690	MFSD6	1.43	1.43	0.52	8.50E-03	1.56E+01	1.56E+01
ENSG00000135837	CEP350	1.54	1.54	0.62	9.76E-03	1.56E+01	1.56E+01
ENSG00000013375	PGM3	0.78	-1.28	-0.36	6.82E-03	-1.55E+01	1.55E+01
ENSG00000170325	PRDM10	1.23	1.23	0.30	6.35E-03	1.55E+01	1.55E+01
ENSG00000123636	BAZ2B	1.47	1.47	0.55	8.95E-03	1.55E+01	1.55E+01
ENSG00000161653	NAGS	0.79	-1.26	-0.33	6.62E-03	-1.55E+01	1.55E+01
ENSG00000116005	PCYOX1	1.26	1.26	0.33	6.59E-03	1.55E+01	1.55E+01
ENSG00000172037	LAMB2	1.34	1.34	0.42	7.45E-03	1.55E+01	1.55E+01
ENSG00000145715	RASA1	1.27	1.27	0.34	6.70E-03	1.55E+01	1.55E+01
	RP11-290F20.1	0.69	-1.45	-0.53	8.73E-03	-1.55E+01	1.55E+01
ENSG00000169180	XPO6	0.80	-1.25	-0.32	6.52E-03	-1.55E+01	1.55E+01
ENSG00000124172	ATP5E	0.78	-1.28	-0.35	6.86E-03	-1.54E+01	1.54E+01
ENSG00000174306	ZHX3	0.81	-1.23	-0.30	6.35E-03	-1.54E+01	1.54E+01
ENSG00000198087	CD2AP	1.28	1.28	0.36	6.89E-03	1.54E+01	1.54E+01
ENSG00000140876	NUDT7	1.34	1.34	0.42	7.52E-03	1.54E+01	1.54E+01
ENSG00000160439	RDH13	0.76	-1.31	-0.39	7.22E-03	-1.54E+01	1.54E+01
ENSG00000101161	PRPF6	0.81	-1.23	-0.30	6.45E-03	-1.54E+01	1.54E+01
ENSG00000115840	SLC25A12	0.82	-1.23	-0.29	6.36E-03	-1.54E+01	1.54E+01
ENSG00000155621	C9orf85	0.76	-1.31	-0.39	7.29E-03	-1.54E+01	1.54E+01

ENSG00000131848	ZSCAN5A	1.37	1.37	0.46	7.97E-03	1.54E+01	1.54E+01
ENSG00000169599	NFU1	0.82	-1.23	-0.29	6.38E-03	-1.54E+01	1.54E+01
ENSG00000124160	NCOA5	1.26	1.26	0.33	6.72E-03	1.54E+01	1.54E+01
ENSG00000115652	UXS1	1.42	1.42	0.50	8.51E-03	1.53E+01	1.53E+01
ENSG00000182108	DEXI	1.22	1.22	0.29	6.37E-03	1.53E+01	1.53E+01
ENSG00000197548	ATG7	0.82	-1.23	-0.29	6.42E-03	-1.53E+01	1.53E+01
ENSG00000196652	ZKSCAN5	1.22	1.22	0.29	6.42E-03	1.53E+01	1.53E+01
ENSG00000168268	NT5DC2	1.24	1.24	0.31	6.62E-03	1.53E+01	1.53E+01
ENSG00000142856	ITGB3BP	0.81	-1.23	-0.30	6.49E-03	-1.53E+01	1.53E+01
ENSG00000173163	COMMD1	0.81	-1.23	-0.30	6.53E-03	-1.52E+01	1.52E+01
ENSG00000138399	FASTKD1	1.22	1.22	0.29	6.44E-03	1.52E+01	1.52E+01
ENSG00000158859	ADAMTS4	1.26	1.26	0.33	6.85E-03	1.52E+01	1.52E+01
ENSG00000133313	CNDP2	1.29	1.29	0.37	7.16E-03	1.52E+01	1.52E+01
ENSG00000183615	FAM167B	1.46	1.46	0.54	9.15E-03	1.52E+01	1.52E+01
ENSG00000140854	KATNB1	0.81	-1.24	-0.31	6.63E-03	-1.52E+01	1.52E+01
ENSG00000104067	TJP1	1.24	1.24	0.31	6.64E-03	1.52E+01	1.52E+01
ENSG00000273559	CWC25	0.80	-1.25	-0.32	6.79E-03	-1.52E+01	1.52E+01
ENSG00000117222	RBBP5	0.82	-1.23	-0.29	6.55E-03	-1.51E+01	1.51E+01
ENSG00000120093	HOXB3	1.24	1.24	0.31	6.73E-03	1.51E+01	1.51E+01
ENSG00000076067	RBMS2	1.25	1.25	0.32	6.80E-03	1.51E+01	1.51E+01
ENSG00000131351	HAUS8	0.80	-1.24	-0.32	6.80E-03	-1.51E+01	1.51E+01
ENSG00000186871	ERCC6L	0.74	-1.35	-0.43	7.99E-03	-1.51E+01	1.51E+01
ENSG00000100413	POLR3H	0.82	-1.22	-0.29	6.58E-03	-1.50E+01	1.50E+01
ENSG00000086666	ZFAND6	1.23	1.23	0.30	6.70E-03	1.50E+01	1.50E+01
ENSG00000152443	ZNF776	1.23	1.23	0.30	6.67E-03	1.50E+01	1.50E+01
	RP3-402G11.5	0.79	-1.26	-0.33	7.03E-03	-1.50E+01	1.50E+01
ENSG00000136141	LRCH1	1.29	1.29	0.37	7.42E-03	1.50E+01	1.50E+01
ENSG00000198755	RPL10A	0.80	-1.25	-0.32	6.92E-03	-1.50E+01	1.50E+01
ENSG00000168152	THAP9	1.33	1.33	0.41	7.85E-03	1.50E+01	1.50E+01
ENSG00000013288	MAN2B2	1.31	1.31	0.39	7.66E-03	1.50E+01	1.50E+01
ENSG00000111880	RNGTT	0.81	-1.23	-0.30	6.72E-03	-1.50E+01	1.50E+01
ENSG00000157014	TATDN2	1.26	1.26	0.33	7.12E-03	1.49E+01	1.49E+01
ENSG00000070756	PABPC1	1.25	1.25	0.32	6.97E-03	1.49E+01	1.49E+01
ENSG00000111911	HINT3	1.22	1.22	0.29	6.70E-03	1.49E+01	1.49E+01
ENSG00000086696	HSD17B2	1.47	1.47	0.55	9.73E-03	1.49E+01	1.49E+01
ENSG00000147099	HDAC8	0.81	-1.23	-0.30	6.86E-03	-1.48E+01	1.48E+01
ENSG00000014919	COX15	1.27	1.27	0.34	7.33E-03	1.48E+01	1.48E+01
ENSG00000085365	SCAMP1	1.29	1.29	0.37	7.63E-03	1.48E+01	1.48E+01
ENSG00000116954	RRAGC	1.27	1.27	0.35	7.45E-03	1.47E+01	1.47E+01
ENSG00000156976	EIF4A2	0.81	-1.24	-0.31	7.09E-03	-1.47E+01	1.47E+01
ENSG00000164237	CMBL	0.80	-1.25	-0.33	7.24E-03	-1.47E+01	1.47E+01
ENSG00000204634	TBC1D8	0.80	-1.25	-0.32	7.18E-03	-1.47E+01	1.47E+01
ENSG00000110925	CSRNP2	1.23	1.23	0.30	7.02E-03	1.47E+01	1.47E+01
ENSG00000133138	TBC1D8B	1.28	1.28	0.35	7.58E-03	1.46E+01	1.46E+01
ENSG00000132196	HSD17B7	1.29	1.29	0.36	7.71E-03	1.46E+01	1.46E+01
ENSG00000197063	MAFG	1.23	1.23	0.29	7.05E-03	1.46E+01	1.46E+01
ENSG00000118705	RPN2	1.24	1.24	0.31	7.17E-03	1.46E+01	1.46E+01
ENSG00000215301	DDX3X	1.28	1.28	0.36	7.75E-03	1.46E+01	1.46E+01
ENSG00000107937	GTPBP4	0.81	-1.23	-0.30	7.17E-03	-1.46E+01	1.46E+01
ENSG00000197299	BLM	0.81	-1.24	-0.31	7.25E-03	-1.46E+01	1.46E+01
ENSG00000173214	KIAA1919	1.26	1.26	0.33	7.45E-03	1.46E+01	1.46E+01
ENSG00000184226	PCDH9	0.78	-1.29	-0.36	7.81E-03	-1.45E+01	1.45E+01
ENSG00000141577	AZI1	0.79	-1.27	-0.34	7.58E-03	-1.45E+01	1.45E+01
ENSG00000153982	GDPD1	0.75	-1.34	-0.42	8.44E-03	-1.45E+01	1.45E+01
ENSG00000143537	ADAM15	1.24	1.24	0.31	7.24E-03	1.45E+01	1.45E+01
ENSG00000009830	POMT2	1.26	1.26	0.33	7.49E-03	1.45E+01	1.45E+01
ENSG00000186812	ZNF397	1.30	1.30	0.38	8.08E-03	1.45E+01	1.45E+01
ENSG00000179941	BBS10	0.81	-1.24	-0.31	7.27E-03	-1.45E+01	1.45E+01
ENSG00000159063	ALG8	1.22	1.22	0.29	7.10E-03	1.45E+01	1.45E+01
ENSG00000181827	RFX7	1.23	1.23	0.30	7.23E-03	1.45E+01	1.45E+01
ENSG00000182667	NTM	1.40	1.40	0.48	9.33E-03	1.45E+01	1.45E+01
ENSG00000169946	ZFPM2	0.80	-1.25	-0.33	7.51E-03	-1.45E+01	1.45E+01
ENSG00000158805	ZNF276	1.22	1.22	0.29	7.15E-03	1.44E+01	1.44E+01
ENSG00000186364	NUDT17	1.37	1.37	0.45	9.02E-03	1.44E+01	1.44E+01
ENSG00000072571	HMMR	1.44	1.44	0.53	1.00E-02	1.44E+01	1.44E+01
ENSG00000100227	POLDIP3	1.23	1.23	0.30	7.26E-03	1.44E+01	1.44E+01
ENSG00000125812	GZF1	0.80	-1.25	-0.33	7.56E-03	-1.44E+01	1.44E+01
ENSG00000198521	ZNF43	1.27	1.27	0.34	7.74E-03	1.44E+01	1.44E+01
ENSG00000111348	ARHGDIB	1.24	1.24	0.31	7.37E-03	1.44E+01	1.44E+01
ENSG00000218347	HNRNPA1P1	1.53	1.53	0.61	1.12E-02	1.44E+01	1.44E+01
ENSG00000197498	RPF2	0.79	-1.26	-0.33	7.74E-03	-1.43E+01	1.43E+01
ENSG00000205707	LYRM5	0.80	-1.24	-0.31	7.56E-03	-1.43E+01	1.43E+01
ENSG00000013523	ANGEL1	1.25	1.25	0.32	7.59E-03	1.43E+01	1.43E+01
ENSG00000268471	DKFZP434I0714	0.73	-1.36	-0.45	9.08E-03	-1.43E+01	1.43E+01
ENSG00000101945	SUV39H1	0.77	-1.29	-0.37	8.17E-03	-1.43E+01	1.43E+01
ENSG00000164053	ATRIP	0.78	-1.28	-0.36	8.07E-03	-1.43E+01	1.43E+01
ENSG00000198945	L3MBTL3	1.26	1.26	0.33	7.73E-03	1.43E+01	1.43E+01
ENSG00000174444	RPL4	0.81	-1.24	-0.31	7.49E-03	-1.43E+01	1.43E+01
ENSG00000262099	AC004148.1	0.70	-1.42	-0.51	9.89E-03	-1.43E+01	1.43E+01

ENSG00000145819	ARHGAP26	0.81	-1.23	-0.30	7.49E-03	-1.43E+01	1.43E+01
ENSG00000113734	BNIP1	0.80	-1.25	-0.33	7.73E-03	-1.42E+01	1.42E+01
ENSG00000198298	ZNF485	0.71	-1.41	-0.50	9.87E-03	-1.42E+01	1.42E+01
ENSG00000240694	PNMA2	0.81	-1.24	-0.31	7.61E-03	-1.42E+01	1.42E+01
ENSG00000240498	CDKN2B-AS1	1.41	1.41	0.49	9.81E-03	1.42E+01	1.42E+01
ENSG00000030110	BAK1	1.24	1.24	0.31	7.65E-03	1.42E+01	1.42E+01
ENSG00000131725	WDR44	1.22	1.22	0.29	7.39E-03	1.42E+01	1.42E+01
ENSG00000135698	MPHOSPH6	0.82	-1.22	-0.28	7.33E-03	-1.42E+01	1.42E+01
ENSG00000112290	WASF1	1.25	1.25	0.32	7.76E-03	1.42E+01	1.42E+01
ENSG00000186352	ANKRD37	1.42	1.42	0.50	9.95E-03	1.42E+01	1.42E+01
ENSG00000072163	LIMS2	0.80	-1.26	-0.33	7.84E-03	-1.42E+01	1.42E+01
ENSG00000141367	CLTC	1.24	1.24	0.31	7.61E-03	1.42E+01	1.42E+01
ENSG00000152684	PELO	1.22	1.22	0.28	7.40E-03	1.41E+01	1.41E+01
ENSG00000151498	ACAD8	0.82	-1.22	-0.29	7.44E-03	-1.41E+01	1.41E+01
ENSG00000158863	FAM160B2	1.26	1.26	0.33	7.91E-03	1.41E+01	1.41E+01
ENSG00000148143	ZNF462	1.25	1.25	0.32	7.85E-03	1.41E+01	1.41E+01
ENSG00000134970	TMED7	0.80	-1.25	-0.32	7.88E-03	-1.41E+01	1.41E+01
ENSG00000179044	EXOC3L1	1.43	1.43	0.52	1.03E-02	1.41E+01	1.41E+01
ENSG00000114302	PRKAR2A	0.81	-1.24	-0.31	7.72E-03	-1.41E+01	1.41E+01
ENSG00000204842	ATXN2	1.26	1.26	0.33	8.02E-03	1.40E+01	1.40E+01
ENSG00000155903	RASA2	1.24	1.24	0.31	7.81E-03	1.40E+01	1.40E+01
ENSG00000103174	NAGPA	1.24	1.24	0.32	7.89E-03	1.40E+01	1.40E+01
ENSG00000136936	XPA	0.81	-1.23	-0.30	7.76E-03	-1.40E+01	1.40E+01
ENSG00000166986	MARS	0.80	-1.24	-0.31	7.88E-03	-1.40E+01	1.40E+01
ENSG00000082516	GEMIN5	0.82	-1.23	-0.29	7.73E-03	-1.39E+01	1.39E+01
ENSG00000179428	AC073072.5	0.72	-1.38	-0.47	9.86E-03	-1.39E+01	1.39E+01
ENSG00000231365	RP11-418J17.1	1.29	1.29	0.37	8.58E-03	1.39E+01	1.39E+01
ENSG00000147454	SLC25A37	1.22	1.22	0.28	7.63E-03	1.39E+01	1.39E+01
ENSG00000227500	SCAMP4	0.78	-1.28	-0.35	8.42E-03	-1.39E+01	1.39E+01
ENSG00000115457	IGFBP2	0.81	-1.24	-0.31	7.94E-03	-1.39E+01	1.39E+01
ENSG00000164684	ZNF704	1.23	1.23	0.30	7.87E-03	1.38E+01	1.38E+01
ENSG00000232956	SNHG15	0.80	-1.25	-0.33	8.20E-03	-1.38E+01	1.38E+01
ENSG00000090006	LTBP4	1.33	1.33	0.41	9.29E-03	1.38E+01	1.38E+01
ENSG00000170889	RPS9	0.79	-1.27	-0.34	8.41E-03	-1.38E+01	1.38E+01
ENSG00000132205	EMILIN2	0.78	-1.29	-0.37	8.69E-03	-1.38E+01	1.38E+01
ENSG00000160695	VPS11	1.22	1.22	0.28	7.75E-03	1.38E+01	1.38E+01
ENSG00000105254	TBCB	0.81	-1.23	-0.30	7.94E-03	-1.38E+01	1.38E+01
ENSG00000258890	CEP95	0.81	-1.23	-0.30	8.02E-03	-1.38E+01	1.38E+01
ENSG00000010438	PRSS3	1.25	1.25	0.32	8.22E-03	1.37E+01	1.37E+01
ENSG00000196367	TRRAP	1.33	1.33	0.41	9.37E-03	1.37E+01	1.37E+01
ENSG00000272333	WBP7	1.26	1.26	0.33	8.40E-03	1.37E+01	1.37E+01
ENSG00000196371	FUT4	0.80	-1.25	-0.32	8.32E-03	-1.37E+01	1.37E+01
	RP11-742N3.1	0.69	-1.45	-0.54	1.13E-02	-1.37E+01	1.37E+01
ENSG00000136059	VILL	0.75	-1.33	-0.41	9.50E-03	-1.37E+01	1.37E+01
ENSG00000088812	ATRN	1.24	1.24	0.31	8.23E-03	1.37E+01	1.37E+01
ENSG00000163166	IWS1	0.82	-1.22	-0.29	8.04E-03	-1.36E+01	1.36E+01
ENSG00000145860	RNF145	1.31	1.31	0.39	9.25E-03	1.36E+01	1.36E+01
ENSG00000122882	ECD	0.82	-1.22	-0.29	7.99E-03	-1.36E+01	1.36E+01
ENSG00000079459	FDFT1	0.81	-1.23	-0.30	8.17E-03	-1.36E+01	1.36E+01
ENSG00000100916	BRMS1L	0.77	-1.30	-0.38	9.15E-03	-1.36E+01	1.36E+01
ENSG00000131116	ZNF428	0.81	-1.23	-0.30	8.13E-03	-1.36E+01	1.36E+01
ENSG00000065802	ASB1	1.22	1.22	0.29	8.06E-03	1.36E+01	1.36E+01
ENSG00000130349	C6orf203	0.81	-1.23	-0.30	8.18E-03	-1.36E+01	1.36E+01
ENSG00000008838	MED24	1.24	1.24	0.31	8.28E-03	1.36E+01	1.36E+01
ENSG00000038219	BOD1L1	1.46	1.46	0.54	1.15E-02	1.36E+01	1.36E+01
ENSG00000104375	STK3	0.80	-1.25	-0.32	8.40E-03	-1.36E+01	1.36E+01
ENSG00000127314	RAP1B	1.25	1.25	0.32	8.51E-03	1.36E+01	1.36E+01
ENSG00000156052	GNAQ	1.23	1.23	0.30	8.21E-03	1.36E+01	1.36E+01
ENSG00000115239	ASB3	1.21	1.21	0.28	8.06E-03	1.35E+01	1.35E+01
ENSG00000068745	IP6K2	1.22	1.22	0.29	8.17E-03	1.35E+01	1.35E+01
ENSG00000008311	AASS	1.23	1.23	0.30	8.25E-03	1.35E+01	1.35E+01
ENSG00000144136	SLC20A1	1.25	1.25	0.32	8.56E-03	1.35E+01	1.35E+01
ENSG00000101773	RBBP8	1.25	1.25	0.32	8.61E-03	1.35E+01	1.35E+01
ENSG00000123595	RAB9A	1.22	1.22	0.28	8.15E-03	1.35E+01	1.35E+01
ENSG00000196981	WDR5B	0.79	-1.26	-0.34	8.79E-03	-1.35E+01	1.35E+01
ENSG00000095066	HOOK2	1.29	1.29	0.37	9.22E-03	1.35E+01	1.35E+01
ENSG00000114790	ARHGEF26	0.64	-1.57	-0.65	1.36E-02	-1.35E+01	1.35E+01
ENSG00000068400	GRIPAP1	0.82	-1.22	-0.29	8.21E-03	-1.35E+01	1.35E+01
ENSG00000114767	RRP9	0.80	-1.24	-0.32	8.56E-03	-1.35E+01	1.35E+01
ENSG00000143515	ATP8B2	1.23	1.23	0.30	8.43E-03	1.35E+01	1.35E+01
ENSG00000119537	KDSR	1.22	1.22	0.29	8.22E-03	1.34E+01	1.34E+01
ENSG00000114978	MOB1A	0.81	-1.23	-0.30	8.40E-03	-1.34E+01	1.34E+01
ENSG00000148362	C9orf142	0.80	-1.25	-0.32	8.70E-03	-1.34E+01	1.34E+01
ENSG00000120616	EPC1	0.82	-1.22	-0.29	8.28E-03	-1.34E+01	1.34E+01
ENSG00000173457	PPP1R14B	1.23	1.23	0.30	8.42E-03	1.34E+01	1.34E+01
ENSG00000138593	SECISBP2L	1.24	1.24	0.31	8.49E-03	1.34E+01	1.34E+01
ENSG00000248175	CTC-428G20.3	0.63	-1.60	-0.68	1.42E-02	-1.34E+01	1.34E+01
ENSG00000181555	SETD2	1.23	1.23	0.30	8.39E-03	1.34E+01	1.34E+01
ENSG00000171316	CHD7	1.22	1.22	0.29	8.30E-03	1.34E+01	1.34E+01

ENSG00000105185	PDCD5	0.81	-1.23	-0.30	8.45E-03	-1.34E+01	1.34E+01
ENSG00000177409	SAMD9L	1.36	1.36	0.45	1.04E-02	1.34E+01	1.34E+01
ENSG00000112511	PHF1	0.80	-1.24	-0.32	8.66E-03	-1.34E+01	1.34E+01
ENSG00000074660	SCARF1	1.24	1.24	0.31	8.59E-03	1.34E+01	1.34E+01
ENSG00000165699	TSC1	1.22	1.22	0.29	8.39E-03	1.34E+01	1.34E+01
ENSG00000122376	FAM35A	1.24	1.24	0.31	8.67E-03	1.33E+01	1.33E+01
ENSG00000099942	CRKL	0.81	-1.24	-0.31	8.60E-03	-1.33E+01	1.33E+01
ENSG00000177096	FAM109B	0.81	-1.24	-0.31	8.67E-03	-1.33E+01	1.33E+01
ENSG00000120784	ZFP30	1.25	1.25	0.32	8.77E-03	1.33E+01	1.33E+01
ENSG00000264207	RP11-196G18.23	0.70	-1.43	-0.51	1.15E-02	-1.33E+01	1.33E+01
ENSG00000032444	PNPLA6	1.26	1.26	0.34	9.05E-03	1.33E+01	1.33E+01
ENSG00000186687	LYRM7	0.81	-1.24	-0.31	8.73E-03	-1.33E+01	1.33E+01
ENSG00000170017	ALCAM	1.27	1.27	0.34	9.17E-03	1.32E+01	1.32E+01
ENSG00000157837	SPPL3	1.24	1.24	0.31	8.81E-03	1.32E+01	1.32E+01
ENSG00000110108	TMEM109	1.26	1.26	0.33	9.12E-03	1.32E+01	1.32E+01
	RP11-453E17.1	0.78	-1.28	-0.35	9.35E-03	-1.32E+01	1.32E+01
ENSG00000177383	MAGEF1	1.23	1.23	0.30	8.76E-03	1.32E+01	1.32E+01
ENSG00000110321	EIF4G2	1.23	1.23	0.30	8.76E-03	1.32E+01	1.32E+01
ENSG00000167550	RHEBL1	0.69	-1.44	-0.53	1.20E-02	-1.32E+01	1.32E+01
ENSG00000015676	NUDCD3	1.22	1.22	0.29	8.66E-03	1.31E+01	1.31E+01
ENSG00000103544	C16orf62	0.81	-1.23	-0.30	8.77E-03	-1.31E+01	1.31E+01
ENSG00000144746	ARL6IP5	1.30	1.30	0.38	9.78E-03	1.31E+01	1.31E+01
ENSG00000169689	STRA13	0.80	-1.25	-0.32	9.04E-03	-1.31E+01	1.31E+01
ENSG00000137338	PGBD1	0.81	-1.23	-0.30	8.82E-03	-1.31E+01	1.31E+01
ENSG00000196693	ZNF33B	1.33	1.33	0.41	1.03E-02	1.31E+01	1.31E+01
ENSG00000137944	CCBL2	0.81	-1.23	-0.30	8.88E-03	-1.31E+01	1.31E+01
ENSG00000176473	WDR25	0.80	-1.25	-0.32	9.14E-03	-1.31E+01	1.31E+01
ENSG00000100379	KCTD17	0.82	-1.22	-0.28	8.67E-03	-1.31E+01	1.31E+01
ENSG00000100426	ZBED4	1.28	1.28	0.35	9.54E-03	1.31E+01	1.31E+01
ENSG00000115274	INO80B	0.81	-1.23	-0.30	8.92E-03	-1.30E+01	1.30E+01
ENSG00000063180	CA11	1.42	1.42	0.51	1.20E-02	1.30E+01	1.30E+01
ENSG00000139163	ETNK1	1.21	1.21	0.28	8.64E-03	1.30E+01	1.30E+01
ENSG00000124181	PLCG1	1.22	1.22	0.29	8.85E-03	1.30E+01	1.30E+01
ENSG00000118922	KLF12	1.29	1.29	0.37	9.87E-03	1.30E+01	1.30E+01
ENSG00000197903	HIST1H2BK	0.80	-1.25	-0.32	9.27E-03	-1.30E+01	1.30E+01
ENSG00000131408	NR1H2	0.82	-1.22	-0.29	8.92E-03	-1.30E+01	1.30E+01
ENSG00000160007	ARHGAP35	1.23	1.23	0.30	9.02E-03	1.29E+01	1.29E+01
ENSG00000213799	ZNF845	1.32	1.32	0.40	1.03E-02	1.29E+01	1.29E+01
ENSG00000151893	CACUL1	1.23	1.23	0.30	9.05E-03	1.29E+01	1.29E+01
ENSG00000213047	DENND1B	1.36	1.36	0.44	1.10E-02	1.29E+01	1.29E+01
ENSG00000091640	SPAG7	1.22	1.22	0.29	8.95E-03	1.29E+01	1.29E+01
ENSG00000003756	RBM5	0.81	-1.24	-0.31	9.18E-03	-1.29E+01	1.29E+01
ENSG00000162817	C1orf115	1.23	1.23	0.30	9.03E-03	1.29E+01	1.29E+01
ENSG00000114391	RPL24	0.81	-1.24	-0.31	9.21E-03	-1.29E+01	1.29E+01
ENSG00000126603	GLIS2	1.45	1.45	0.53	1.26E-02	1.29E+01	1.29E+01
ENSG00000101850	GPR143	1.22	1.22	0.29	8.97E-03	1.29E+01	1.29E+01
ENSG00000118454	ANKRD13C	1.21	1.21	0.28	8.82E-03	1.29E+01	1.29E+01
ENSG00000174353	STAG3L3	0.68	-1.46	-0.55	1.28E-02	-1.29E+01	1.29E+01
ENSG00000154429	CCSAP	0.82	-1.22	-0.28	8.93E-03	-1.29E+01	1.29E+01
ENSG00000148841	ITPRIP	1.22	1.22	0.29	9.03E-03	1.29E+01	1.29E+01
ENSG00000159433	STARD9	0.80	-1.25	-0.32	9.41E-03	-1.29E+01	1.29E+01
ENSG00000095794	CREM	1.23	1.23	0.30	9.12E-03	1.29E+01	1.29E+01
ENSG00000164542	KIAA0895	1.27	1.27	0.35	9.75E-03	1.29E+01	1.29E+01
ENSG00000187650	VMAC	0.72	-1.39	-0.48	1.17E-02	-1.29E+01	1.29E+01
ENSG00000196182	STK40	1.22	1.22	0.29	9.06E-03	1.29E+01	1.29E+01
ENSG00000172757	CFL1	1.24	1.24	0.31	9.26E-03	1.28E+01	1.28E+01
ENSG00000127311	HELB	0.75	-1.34	-0.42	1.09E-02	-1.28E+01	1.28E+01
ENSG00000132357	CARD6	1.22	1.22	0.29	9.10E-03	1.28E+01	1.28E+01
ENSG00000269881	ITFG3	1.26	1.26	0.34	9.73E-03	1.28E+01	1.28E+01
ENSG00000054118	THRAP3	0.82	-1.23	-0.29	9.16E-03	-1.28E+01	1.28E+01
ENSG00000119509	INVS	1.21	1.21	0.28	8.97E-03	1.28E+01	1.28E+01
ENSG00000154978	VOPP1	1.23	1.23	0.30	9.28E-03	1.28E+01	1.28E+01
ENSG00000167232	ZNF91	1.33	1.33	0.41	1.08E-02	1.28E+01	1.28E+01
ENSG00000103199	ZNF500	1.25	1.25	0.32	9.54E-03	1.28E+01	1.28E+01
ENSG00000171295	ZNF440	1.23	1.23	0.30	9.27E-03	1.28E+01	1.28E+01
ENSG00000121621	KIF18A	0.79	-1.27	-0.34	9.84E-03	-1.28E+01	1.28E+01
ENSG00000124571	XPO5	0.82	-1.23	-0.29	9.25E-03	-1.27E+01	1.27E+01
ENSG00000084652	TXLNA	0.81	-1.24	-0.31	9.47E-03	-1.27E+01	1.27E+01
ENSG00000117632	STMN1	1.39	1.39	0.48	1.20E-02	1.27E+01	1.27E+01
ENSG00000214413	BBIP1	1.22	1.22	0.29	9.22E-03	1.27E+01	1.27E+01
ENSG00000011638	TMEM159	0.82	-1.21	-0.28	9.16E-03	-1.27E+01	1.27E+01
ENSG00000257093	KIAA1147	1.23	1.23	0.29	9.33E-03	1.27E+01	1.27E+01
ENSG00000178177	LCORL	0.79	-1.27	-0.34	9.99E-03	-1.27E+01	1.27E+01
ENSG00000213366	GSTM2	0.74	-1.36	-0.44	1.14E-02	-1.27E+01	1.27E+01
ENSG00000153107	ANAPC1	0.82	-1.22	-0.29	9.28E-03	-1.27E+01	1.27E+01
ENSG00000003436	TFPI	1.31	1.31	0.39	1.07E-02	1.27E+01	1.27E+01
ENSG00000214655	ZSWIM8	1.22	1.22	0.28	9.23E-03	1.27E+01	1.27E+01
ENSG00000110888	CAPRIN2	0.82	-1.22	-0.28	9.26E-03	-1.26E+01	1.26E+01
ENSG00000161800	RACGAP1	0.81	-1.23	-0.30	9.43E-03	-1.26E+01	1.26E+01

ENSG00000101544	ADNP2	0.80	-1.25	-0.32	9.80E-03	-1.26E+01	1.26E+01
ENSG00000099625	C19orf26	1.35	1.35	0.44	1.15E-02	1.26E+01	1.26E+01
ENSG00000196961	AP2A1	1.26	1.26	0.33	9.93E-03	1.26E+01	1.26E+01
ENSG00000186767	SPIN4	0.80	-1.26	-0.33	9.96E-03	-1.26E+01	1.26E+01
ENSG00000224959	AC017002.2	1.36	1.36	0.45	1.17E-02	1.26E+01	1.26E+01
ENSG00000112159	MDN1	0.81	-1.24	-0.31	9.70E-03	-1.26E+01	1.26E+01
ENSG00000102934	PLLP	1.33	1.33	0.41	1.11E-02	1.26E+01	1.26E+01
ENSG00000162607	USP1	0.79	-1.26	-0.34	1.01E-02	-1.26E+01	1.26E+01
ENSG00000140743	CDR2	1.22	1.22	0.28	9.37E-03	1.26E+01	1.26E+01
ENSG00000164897	TMUB1	0.79	-1.27	-0.34	1.02E-02	-1.25E+01	1.25E+01
ENSG00000178381	ZFAND2A	1.24	1.24	0.31	9.75E-03	1.25E+01	1.25E+01
ENSG00000189306	RRP7A	0.82	-1.22	-0.29	9.53E-03	-1.25E+01	1.25E+01
ENSG00000112578	BYSL	0.79	-1.26	-0.34	1.02E-02	-1.25E+01	1.25E+01
ENSG00000131724	IL13RA1	1.23	1.23	0.30	9.63E-03	1.25E+01	1.25E+01
ENSG00000117595	IRF6	1.31	1.31	0.39	1.10E-02	1.25E+01	1.25E+01
ENSG00000175203	DCTN2	1.23	1.23	0.30	9.68E-03	1.25E+01	1.25E+01
ENSG00000155099	TMEM55A	0.80	-1.25	-0.32	9.99E-03	-1.25E+01	1.25E+01
ENSG00000040531	CTNS	0.81	-1.23	-0.30	9.75E-03	-1.25E+01	1.25E+01
ENSG00000117586	TNFSF4	1.26	1.26	0.33	1.02E-02	1.25E+01	1.25E+01
ENSG00000083123	BCKDHB	0.82	-1.22	-0.29	9.65E-03	-1.25E+01	1.25E+01
ENSG00000088247	KHSRP	1.23	1.23	0.30	9.69E-03	1.25E+01	1.25E+01
ENSG00000131791	PRKAB2	1.21	1.21	0.28	9.49E-03	1.25E+01	1.25E+01
ENSG00000180318	ALX1	0.72	-1.39	-0.47	1.24E-02	-1.24E+01	1.24E+01
ENSG00000103042	SLC38A7	1.22	1.22	0.28	9.57E-03	1.24E+01	1.24E+01
ENSG00000175265	GOLGA8A	0.75	-1.32	-0.41	1.14E-02	-1.24E+01	1.24E+01
ENSG00000133739	LRRCC1	1.30	1.30	0.38	1.10E-02	1.24E+01	1.24E+01
ENSG00000105700	KXD1	1.25	1.25	0.32	1.02E-02	1.24E+01	1.24E+01
ENSG00000197619	ZNF615	1.33	1.33	0.41	1.15E-02	1.24E+01	1.24E+01
ENSG00000224536	AC096677.1	1.43	1.43	0.52	1.34E-02	1.24E+01	1.24E+01
ENSG00000201882	snoU2-30	1.41	1.41	0.50	1.30E-02	1.24E+01	1.24E+01
ENSG00000162032	SPSB3	0.81	-1.24	-0.31	9.98E-03	-1.24E+01	1.24E+01
ENSG00000137038	C9orf123	1.21	1.21	0.28	9.60E-03	1.24E+01	1.24E+01
ENSG00000113758	DBN1	1.24	1.24	0.31	1.00E-02	1.24E+01	1.24E+01
ENSG00000163110	PDLIM5	1.23	1.23	0.30	9.93E-03	1.24E+01	1.24E+01
ENSG00000204536	CCHCR1	0.80	-1.25	-0.33	1.03E-02	-1.23E+01	1.23E+01
ENSG00000157106	SMG1	1.23	1.23	0.30	9.88E-03	1.23E+01	1.23E+01
ENSG00000122026	RPL21	0.81	-1.24	-0.31	1.00E-02	-1.23E+01	1.23E+01
ENSG00000144366	GULP1	1.25	1.25	0.32	1.03E-02	1.23E+01	1.23E+01
ENSG00000066923	STAG3	0.71	-1.41	-0.50	1.31E-02	-1.23E+01	1.23E+01
ENSG00000112210	RAB23	1.22	1.22	0.28	9.75E-03	1.23E+01	1.23E+01
ENSG00000177728	KIAA0195	1.28	1.28	0.35	1.08E-02	1.23E+01	1.23E+01
ENSG00000010322	NISCH	1.23	1.23	0.30	1.00E-02	1.23E+01	1.23E+01
ENSG00000145041	VPRBP	0.81	-1.23	-0.30	1.00E-02	-1.23E+01	1.23E+01
ENSG00000144228	SPOPL	1.26	1.26	0.34	1.06E-02	1.23E+01	1.23E+01
ENSG00000134278	SPIRE1	1.23	1.23	0.30	1.01E-02	1.23E+01	1.23E+01
ENSG00000103150	MLYCD	1.28	1.28	0.35	1.09E-02	1.23E+01	1.23E+01
ENSG00000139998	RAB15	1.21	1.21	0.28	9.81E-03	1.22E+01	1.22E+01
ENSG00000168118	RAB4A	0.82	-1.21	-0.28	9.85E-03	-1.22E+01	1.22E+01
ENSG00000180104	EXOC3	0.83	-1.21	-0.27	9.73E-03	-1.22E+01	1.22E+01
ENSG00000113448	PDE4D	1.24	1.24	0.31	1.02E-02	1.22E+01	1.22E+01
ENSG00000011021	CLCN6	1.25	1.25	0.32	1.04E-02	1.22E+01	1.22E+01
ENSG00000054967	RELT	0.80	-1.25	-0.32	1.04E-02	-1.22E+01	1.22E+01
ENSG00000143294	PRCC	1.25	1.25	0.32	1.05E-02	1.22E+01	1.22E+01
ENSG00000119333	WDR34	0.82	-1.22	-0.29	9.99E-03	-1.22E+01	1.22E+01
ENSG00000154359	LONRF1	0.81	-1.23	-0.30	1.02E-02	-1.22E+01	1.22E+01
ENSG00000132300	PTCD3	0.82	-1.22	-0.29	1.01E-02	-1.22E+01	1.22E+01
ENSG00000179943	FIZ1	1.31	1.31	0.39	1.16E-02	1.22E+01	1.22E+01
ENSG00000166681	NGFRAP1	0.81	-1.23	-0.30	1.03E-02	-1.22E+01	1.22E+01
ENSG00000147133	TAF1	1.22	1.22	0.28	1.00E-02	1.21E+01	1.21E+01
ENSG00000143924	EML4	0.82	-1.22	-0.29	1.01E-02	-1.21E+01	1.21E+01
ENSG00000260565	ERVK13-1	1.26	1.26	0.33	1.08E-02	1.21E+01	1.21E+01
ENSG00000171943	SRGAP2C	1.33	1.33	0.41	1.21E-02	1.21E+01	1.21E+01
	RP11-15H20.5	1.40	1.40	0.49	1.34E-02	1.21E+01	1.21E+01
ENSG00000083535	PIBF1	1.24	1.24	0.31	1.04E-02	1.21E+01	1.21E+01
ENSG00000119185	ITGB1BP1	0.82	-1.22	-0.29	1.02E-02	-1.21E+01	1.21E+01
ENSG00000185344	ATP6V0A2	1.22	1.22	0.28	1.01E-02	1.21E+01	1.21E+01
ENSG00000170949	ZNF160	1.21	1.21	0.28	1.01E-02	1.21E+01	1.21E+01
ENSG00000214160	ALG3	1.24	1.24	0.31	1.06E-02	1.21E+01	1.21E+01
ENSG00000168397	ATG4B	1.21	1.21	0.27	1.00E-02	1.21E+01	1.21E+01
ENSG00000173275	ZNF449	1.25	1.25	0.32	1.08E-02	1.21E+01	1.21E+01
ENSG00000130544	ZNF557	1.23	1.23	0.30	1.05E-02	1.20E+01	1.20E+01
ENSG00000147224	PRPS1	0.83	-1.21	-0.28	1.02E-02	-1.20E+01	1.20E+01
ENSG00000168883	USP39	0.82	-1.22	-0.28	1.03E-02	-1.20E+01	1.20E+01
ENSG00000140350	ANP32A	0.81	-1.23	-0.30	1.05E-02	-1.20E+01	1.20E+01
ENSG00000129691	ASH2L	0.77	-1.30	-0.38	1.18E-02	-1.20E+01	1.20E+01
ENSG00000015171	ZMYND11	1.22	1.22	0.29	1.04E-02	1.20E+01	1.20E+01
ENSG00000111731	KIAA0528	0.79	-1.27	-0.34	1.13E-02	-1.19E+01	1.19E+01
ENSG00000267365	KCNJ2-AS1	1.34	1.34	0.42	1.25E-02	1.19E+01	1.19E+01
ENSG00000139180	NDUFA9	0.80	-1.24	-0.31	1.08E-02	-1.19E+01	1.19E+01

ENSG00000112110	MRPL18	0.82	-1.22	-0.28	1.04E-02	-1.19E+01	1.19E+01
ENSG00000157224	CLDN12	1.25	1.25	0.32	1.10E-02	1.19E+01	1.19E+01
ENSG00000165527	ARF6	0.82	-1.22	-0.29	1.05E-02	-1.19E+01	1.19E+01
ENSG00000008710	PKD1	1.22	1.22	0.29	1.05E-02	1.19E+01	1.19E+01
ENSG00000163738	MTHFD2L	1.21	1.21	0.27	1.03E-02	1.19E+01	1.19E+01
ENSG00000132109	TRIM21	1.21	1.21	0.27	1.04E-02	1.19E+01	1.19E+01
ENSG00000133884	DPF2	1.21	1.21	0.28	1.04E-02	1.19E+01	1.19E+01
ENSG00000188994	ZNF292	1.36	1.36	0.44	1.30E-02	1.19E+01	1.19E+01
ENSG00000100711	ZFYVE21	0.82	-1.21	-0.28	1.04E-02	-1.19E+01	1.19E+01
ENSG00000104884	ERCC2	0.82	-1.22	-0.29	1.06E-02	-1.19E+01	1.19E+01
ENSG00000130779	CLIP1	1.26	1.26	0.34	1.14E-02	1.18E+01	1.18E+01
ENSG00000133114	KIAA1704	0.83	-1.21	-0.27	1.04E-02	-1.18E+01	1.18E+01
ENSG00000152763	WDR78	1.34	1.34	0.42	1.30E-02	1.18E+01	1.18E+01
ENSG00000241343	RPL36A	0.80	-1.26	-0.33	1.14E-02	-1.18E+01	1.18E+01
ENSG00000175198	PCCA	1.21	1.21	0.28	1.07E-02	1.17E+01	1.17E+01
ENSG00000153815	CMIP	1.29	1.29	0.37	1.21E-02	1.17E+01	1.17E+01
ENSG00000150630	VEGFC	0.83	-1.21	-0.28	1.07E-02	-1.17E+01	1.17E+01
ENSG00000102554	KLF5	0.69	-1.44	-0.53	1.52E-02	-1.17E+01	1.17E+01
ENSG00000170379	FAM115C	0.79	-1.27	-0.34	1.18E-02	-1.17E+01	1.17E+01
ENSG00000106299	WASL	1.24	1.24	0.30	1.12E-02	1.17E+01	1.17E+01
ENSG00000087263	OGFOD1	0.82	-1.22	-0.29	1.09E-02	-1.17E+01	1.17E+01
ENSG00000009307	CSDE1	1.22	1.22	0.29	1.10E-02	1.17E+01	1.17E+01
ENSG00000115524	SF3B1	1.24	1.24	0.31	1.13E-02	1.17E+01	1.17E+01
ENSG00000136122	BORA	0.81	-1.23	-0.30	1.12E-02	-1.17E+01	1.17E+01
ENSG00000164535	DAGLB	0.82	-1.22	-0.29	1.11E-02	-1.16E+01	1.16E+01
ENSG00000153574	RPIA	0.81	-1.24	-0.31	1.14E-02	-1.16E+01	1.16E+01
ENSG00000196155	PLEKHG4	1.21	1.21	0.28	1.09E-02	1.16E+01	1.16E+01
ENSG00000197329	PELI1	1.21	1.21	0.27	1.08E-02	1.16E+01	1.16E+01
ENSG00000004455	AK2	0.82	-1.22	-0.28	1.10E-02	-1.16E+01	1.16E+01
ENSG00000224660	SH3BP5-AS1	1.33	1.33	0.41	1.32E-02	1.16E+01	1.16E+01
ENSG00000133627	ACTR3B	0.75	-1.33	-0.41	1.31E-02	-1.16E+01	1.16E+01
ENSG00000136908	DPM2	0.81	-1.24	-0.31	1.14E-02	-1.16E+01	1.16E+01
ENSG00000164054	SHISA5	1.23	1.23	0.30	1.13E-02	1.16E+01	1.16E+01
ENSG00000164877	MICALL2	1.24	1.24	0.31	1.16E-02	1.15E+01	1.15E+01
ENSG00000106617	PRKAG2	1.22	1.22	0.29	1.12E-02	1.15E+01	1.15E+01
ENSG00000144792	ZNF660	0.73	-1.37	-0.45	1.41E-02	-1.15E+01	1.15E+01
ENSG00000112320	SOBP	1.28	1.28	0.35	1.23E-02	1.15E+01	1.15E+01
ENSG00000138081	FBXO11	1.21	1.21	0.28	1.11E-02	1.15E+01	1.15E+01
ENSG00000162231	NXF1	1.30	1.30	0.38	1.28E-02	1.15E+01	1.15E+01
ENSG00000186468	RPS23	0.80	-1.25	-0.32	1.18E-02	-1.15E+01	1.15E+01
ENSG00000196935	SRGAP1	1.25	1.25	0.32	1.19E-02	1.15E+01	1.15E+01
ENSG00000181220	ZNF746	1.24	1.24	0.31	1.17E-02	1.15E+01	1.15E+01
ENSG00000129946	SHC2	1.32	1.32	0.40	1.33E-02	1.14E+01	1.14E+01
ENSG00000126107	HECTD3	1.28	1.28	0.36	1.26E-02	1.14E+01	1.14E+01
ENSG00000189332	RP11-113D6.10	0.80	-1.24	-0.31	1.19E-02	-1.14E+01	1.14E+01
ENSG00000154719	MRPL39	0.83	-1.21	-0.27	1.12E-02	-1.14E+01	1.14E+01
ENSG00000084093	REST	1.22	1.22	0.29	1.15E-02	1.14E+01	1.14E+01
ENSG00000051009	FAM160A2	1.22	1.22	0.29	1.15E-02	1.14E+01	1.14E+01
ENSG00000099337	KCNK6	0.76	-1.31	-0.39	1.32E-02	-1.14E+01	1.14E+01
ENSG00000215193	PEX26	0.83	-1.21	-0.27	1.13E-02	-1.14E+01	1.14E+01
ENSG00000163626	COX18	1.20	1.20	0.27	1.12E-02	1.14E+01	1.14E+01
ENSG00000140983	RHOT2	0.83	-1.21	-0.28	1.14E-02	-1.14E+01	1.14E+01
ENSG00000163811	WDR43	0.82	-1.22	-0.29	1.15E-02	-1.14E+01	1.14E+01
ENSG00000180329	CCDC43	0.83	-1.21	-0.27	1.14E-02	-1.13E+01	1.13E+01
ENSG00000135250	SRPK2	0.82	-1.21	-0.28	1.14E-02	-1.13E+01	1.13E+01
ENSG00000115307	AUP1	1.22	1.22	0.28	1.15E-02	1.13E+01	1.13E+01
ENSG00000115993	TRAK2	1.22	1.22	0.28	1.15E-02	1.13E+01	1.13E+01
ENSG00000002834	LASP1	1.22	1.22	0.29	1.16E-02	1.13E+01	1.13E+01
ENSG00000070010	UFD1L	1.22	1.22	0.28	1.16E-02	1.13E+01	1.13E+01
ENSG00000131019	ULBP3	0.72	-1.39	-0.47	1.51E-02	-1.13E+01	1.13E+01
	RP11-5N23.2	0.72	-1.40	-0.48	1.53E-02	-1.13E+01	1.13E+01
ENSG00000259001	RPPH1	0.79	-1.27	-0.34	1.27E-02	-1.13E+01	1.13E+01
ENSG00000130052	STARD8	1.23	1.23	0.30	1.20E-02	1.12E+01	1.12E+01
ENSG00000170633	RNF34	1.21	1.21	0.28	1.16E-02	1.12E+01	1.12E+01
ENSG00000121716	PILRB	0.80	-1.25	-0.32	1.24E-02	-1.12E+01	1.12E+01
ENSG00000143970	ASXL2	1.24	1.24	0.31	1.23E-02	1.12E+01	1.12E+01
ENSG00000090674	MCOLN1	0.78	-1.28	-0.36	1.31E-02	-1.12E+01	1.12E+01
ENSG00000196914	ARHGEF12	1.22	1.22	0.29	1.19E-02	1.12E+01	1.12E+01
ENSG00000103018	CYB5B	0.82	-1.22	-0.29	1.20E-02	-1.12E+01	1.12E+01
ENSG00000170836	PPM1D	1.21	1.21	0.27	1.17E-02	1.12E+01	1.12E+01
ENSG00000065717	TLE2	1.24	1.24	0.31	1.24E-02	1.12E+01	1.12E+01
ENSG00000215068	AC025171.1	0.74	-1.35	-0.43	1.47E-02	-1.11E+01	1.11E+01
ENSG00000020426	MNAT1	0.83	-1.20	-0.26	1.16E-02	-1.11E+01	1.11E+01
ENSG00000125755	SYMPK	1.21	1.21	0.28	1.19E-02	1.11E+01	1.11E+01
ENSG00000132024	CC2D1A	1.31	1.31	0.39	1.38E-02	1.11E+01	1.11E+01
ENSG00000163655	GMPS	0.82	-1.22	-0.29	1.21E-02	-1.11E+01	1.11E+01
ENSG00000116285	ERRFI1	0.82	-1.22	-0.29	1.21E-02	-1.11E+01	1.11E+01
ENSG00000133393	FOPNL	0.83	-1.21	-0.27	1.19E-02	-1.11E+01	1.11E+01
ENSG00000111554	MDM1	0.80	-1.24	-0.31	1.26E-02	-1.11E+01	1.11E+01

ENSG00000111252	SH2B3	1.23	1.23	0.30	1.24E-02	1.11E+01	1.11E+01
ENSG00000130703	OSBPL2	1.22	1.22	0.28	1.21E-02	1.10E+01	1.10E+01
ENSG00000074964	ARHGEF10L	1.28	1.28	0.35	1.34E-02	1.10E+01	1.10E+01
ENSG00000196739	COL27A1	1.20	1.20	0.26	1.19E-02	1.10E+01	1.10E+01
ENSG00000236104	ZBTB22	0.81	-1.23	-0.30	1.25E-02	-1.10E+01	1.10E+01
ENSG00000162222	TTC9C	0.83	-1.21	-0.27	1.20E-02	-1.10E+01	1.10E+01
ENSG00000074219	TEAD2	0.83	-1.21	-0.28	1.21E-02	-1.10E+01	1.10E+01
ENSG00000204178	TMEM57	1.21	1.21	0.27	1.21E-02	1.10E+01	1.10E+01
ENSG00000127184	COX7C	0.80	-1.24	-0.31	1.29E-02	-1.10E+01	1.10E+01
	RP11-65F13.2	0.80	-1.25	-0.32	1.30E-02	-1.10E+01	1.10E+01
ENSG00000152683	SLC30A6	0.83	-1.20	-0.27	1.20E-02	-1.10E+01	1.10E+01
ENSG00000102189	EEA1	1.39	1.39	0.48	1.61E-02	1.09E+01	1.09E+01
ENSG00000198373	WWP2	1.26	1.26	0.33	1.33E-02	1.09E+01	1.09E+01
ENSG00000162129	CLPB	0.82	-1.22	-0.29	1.25E-02	-1.09E+01	1.09E+01
ENSG00000140464	PML	1.24	1.24	0.31	1.29E-02	1.09E+01	1.09E+01
ENSG00000180979	LRRC57	0.83	-1.21	-0.27	1.22E-02	-1.09E+01	1.09E+01
ENSG00000128578	STRIP2	0.83	-1.21	-0.27	1.22E-02	-1.09E+01	1.09E+01
ENSG00000091073	DTX2	1.31	1.31	0.38	1.44E-02	1.09E+01	1.09E+01
ENSG00000115649	CNPPD1	1.21	1.21	0.27	1.22E-02	1.09E+01	1.09E+01
ENSG00000123106	CCDC91	1.24	1.24	0.31	1.30E-02	1.09E+01	1.09E+01
ENSG00000135974	C2orf49	0.83	-1.20	-0.27	1.22E-02	-1.09E+01	1.09E+01
ENSG00000117592	PRDX6	0.82	-1.22	-0.29	1.26E-02	-1.09E+01	1.09E+01
ENSG00000172339	ALG14	0.80	-1.25	-0.32	1.32E-02	-1.09E+01	1.09E+01
ENSG00000108840	HDAC5	1.23	1.23	0.29	1.27E-02	1.09E+01	1.09E+01
ENSG00000228106	RP11-452F19.3	1.32	1.32	0.40	1.47E-02	1.09E+01	1.09E+01
ENSG00000112787	FBRSL1	1.24	1.24	0.31	1.30E-02	1.09E+01	1.09E+01
ENSG00000176406	RIMS2	0.73	-1.37	-0.45	1.58E-02	-1.09E+01	1.09E+01
ENSG00000115234	SNX17	0.83	-1.21	-0.28	1.25E-02	-1.08E+01	1.08E+01
ENSG00000138688	KIAA1109	1.47	1.47	0.55	1.84E-02	1.08E+01	1.08E+01
ENSG00000138382	METTL5	0.83	-1.20	-0.27	1.24E-02	-1.08E+01	1.08E+01
ENSG00000122958	VPS26A	1.24	1.24	0.31	1.32E-02	1.08E+01	1.08E+01
ENSG00000211456	SACM1L	1.20	1.20	0.26	1.24E-02	1.08E+01	1.08E+01
ENSG00000135164	DMTF1	1.26	1.26	0.34	1.37E-02	1.08E+01	1.08E+01
ENSG00000171448	ZBTB26	0.75	-1.34	-0.42	1.54E-02	-1.08E+01	1.08E+01
ENSG00000107897	ACBD5	1.21	1.21	0.27	1.25E-02	1.08E+01	1.08E+01
ENSG00000179163	FUCA1	1.25	1.25	0.32	1.34E-02	1.08E+01	1.08E+01
ENSG00000163882	POLR2H	0.82	-1.22	-0.28	1.28E-02	-1.08E+01	1.08E+01
ENSG00000135046	ANXA1	1.29	1.29	0.36	1.43E-02	1.08E+01	1.08E+01
ENSG00000185614	FAM212A	1.31	1.31	0.39	1.48E-02	1.08E+01	1.08E+01
ENSG00000126070	EIF2C3	0.82	-1.21	-0.28	1.27E-02	-1.08E+01	1.08E+01
ENSG00000140943	MBTPS1	1.24	1.24	0.31	1.34E-02	1.08E+01	1.08E+01
ENSG00000007520	TSR3	0.83	-1.20	-0.26	1.25E-02	-1.07E+01	1.07E+01
ENSG00000167136	ENDOG	0.79	-1.26	-0.34	1.39E-02	-1.07E+01	1.07E+01
ENSG00000140262	TCF12	1.22	1.22	0.29	1.31E-02	1.07E+01	1.07E+01
ENSG00000032742	IFT88	1.22	1.22	0.29	1.30E-02	1.07E+01	1.07E+01
ENSG00000047578	KIAA0556	1.23	1.23	0.30	1.32E-02	1.07E+01	1.07E+01
ENSG00000110063	DCPS	0.80	-1.25	-0.33	1.37E-02	-1.07E+01	1.07E+01
ENSG00000134146	ATPBD4	1.27	1.27	0.34	1.41E-02	1.07E+01	1.07E+01
ENSG00000180008	SOCS4	1.28	1.28	0.36	1.44E-02	1.07E+01	1.07E+01
ENSG00000173230	GOLGB1	1.33	1.33	0.42	1.57E-02	1.07E+01	1.07E+01
ENSG00000004399	PLXND1	1.22	1.22	0.28	1.30E-02	1.07E+01	1.07E+01
ENSG00000109270	LAMTOR3	1.21	1.21	0.27	1.29E-02	1.06E+01	1.06E+01
ENSG00000167118	URM1	1.21	1.21	0.28	1.30E-02	1.06E+01	1.06E+01
ENSG00000140092	FBLN5	1.31	1.31	0.39	1.52E-02	1.06E+01	1.06E+01
ENSG00000244509	APOBEC3C	0.66	-1.52	-0.60	2.04E-02	-1.06E+01	1.06E+01
ENSG00000179094	PER1	0.76	-1.31	-0.39	1.51E-02	-1.06E+01	1.06E+01
ENSG00000140553	UNC45A	0.82	-1.22	-0.28	1.31E-02	-1.06E+01	1.06E+01
ENSG00000119703	ZC2HC1C	1.42	1.42	0.51	1.80E-02	1.06E+01	1.06E+01
ENSG00000135931	ARMC9	1.21	1.21	0.27	1.30E-02	1.06E+01	1.06E+01
ENSG00000267673	FDX1L	0.81	-1.23	-0.30	1.35E-02	-1.06E+01	1.06E+01
ENSG00000231925	TAPBP	0.80	-1.25	-0.33	1.41E-02	-1.06E+01	1.06E+01
ENSG00000166471	TMEM41B	0.81	-1.23	-0.30	1.35E-02	-1.06E+01	1.06E+01
ENSG00000105856	HBP1	1.22	1.22	0.29	1.33E-02	1.06E+01	1.06E+01
ENSG00000169223	LMAN2	1.21	1.21	0.28	1.32E-02	1.06E+01	1.06E+01
ENSG00000176788	BASP1	1.21	1.21	0.28	1.33E-02	1.05E+01	1.05E+01
ENSG00000013810	TACC3	0.82	-1.22	-0.29	1.34E-02	-1.05E+01	1.05E+01
ENSG00000128283	CDC42EP1	1.22	1.22	0.29	1.35E-02	1.05E+01	1.05E+01
ENSG00000145907	G3BP1	0.82	-1.22	-0.29	1.35E-02	-1.05E+01	1.05E+01
ENSG00000124222	STX16	0.70	-1.43	-0.52	1.86E-02	-1.05E+01	1.05E+01
ENSG00000255090	RP11-820L6.1	0.70	-1.43	-0.51	1.85E-02	-1.05E+01	1.05E+01
ENSG00000198034	RPS4X	0.82	-1.22	-0.29	1.35E-02	-1.05E+01	1.05E+01
ENSG00000165490	C11orf82	0.79	-1.27	-0.35	1.47E-02	-1.05E+01	1.05E+01
ENSG00000134202	GSTM3	0.83	-1.20	-0.27	1.31E-02	-1.05E+01	1.05E+01
ENSG00000148296	SURF6	0.83	-1.21	-0.27	1.33E-02	-1.05E+01	1.05E+01
ENSG00000172239	PAIP1	1.22	1.22	0.28	1.36E-02	1.04E+01	1.04E+01
ENSG00000091436	MLTK	0.82	-1.21	-0.28	1.35E-02	-1.04E+01	1.04E+01
ENSG00000224078	SNHG14	1.21	1.21	0.28	1.35E-02	1.04E+01	1.04E+01
ENSG00000067533	RRP15	0.83	-1.21	-0.27	1.34E-02	-1.04E+01	1.04E+01
ENSG00000109814	UGDH	0.81	-1.23	-0.30	1.38E-02	-1.04E+01	1.04E+01

ENSG00000097046	CDC7	0.79	-1.27	-0.35	1.48E-02	-1.04E+01	1.04E+01
ENSG00000110801	PSMD9	0.83	-1.21	-0.27	1.35E-02	-1.04E+01	1.04E+01
ENSG00000105323	HNRNPUL1	0.80	-1.25	-0.33	1.44E-02	-1.04E+01	1.04E+01
ENSG00000146856	AGBL3	0.76	-1.32	-0.40	1.61E-02	-1.04E+01	1.04E+01
ENSG00000127022	CANX	1.22	1.22	0.29	1.38E-02	1.04E+01	1.04E+01
ENSG00000101187	SLCO4A1	0.77	-1.31	-0.39	1.58E-02	-1.04E+01	1.04E+01
ENSG00000260534	RP11-1006G14.4	1.51	1.51	0.59	2.11E-02	1.04E+01	1.04E+01
ENSG00000105426	PTPRS	1.29	1.29	0.37	1.55E-02	1.04E+01	1.04E+01
ENSG00000164077	MON1A	0.82	-1.22	-0.29	1.39E-02	-1.04E+01	1.04E+01
ENSG00000214176	PLEKHM1P	1.24	1.24	0.31	1.43E-02	1.04E+01	1.04E+01
ENSG00000116962	NID1	1.33	1.33	0.41	1.64E-02	1.04E+01	1.04E+01
ENSG00000163956	LRPAP1	1.21	1.21	0.28	1.37E-02	1.03E+01	1.03E+01
ENSG00000130338	TULP4	1.21	1.21	0.27	1.36E-02	1.03E+01	1.03E+01
ENSG00000011304	PTBP1	1.24	1.24	0.31	1.45E-02	1.03E+01	1.03E+01
ENSG00000186141	POLR3C	0.83	-1.20	-0.26	1.36E-02	-1.03E+01	1.03E+01
ENSG00000121879	PIK3CA	1.30	1.30	0.38	1.59E-02	1.03E+01	1.03E+01
ENSG00000101811	CSTF2	0.83	-1.20	-0.26	1.35E-02	-1.03E+01	1.03E+01
ENSG00000124177	CHD6	1.23	1.23	0.29	1.41E-02	1.03E+01	1.03E+01
ENSG00000011143	MKS1	1.23	1.23	0.30	1.42E-02	1.03E+01	1.03E+01
ENSG00000204767	FAM196B	0.79	-1.27	-0.34	1.51E-02	-1.03E+01	1.03E+01
ENSG00000164050	PLXNB1	1.22	1.22	0.28	1.40E-02	1.03E+01	1.03E+01
ENSG00000104381	GDAP1	0.75	-1.33	-0.41	1.67E-02	-1.03E+01	1.03E+01
ENSG00000156482	RPL30	0.81	-1.23	-0.30	1.43E-02	-1.03E+01	1.03E+01
ENSG00000148444	COMMD3	1.20	1.20	0.26	1.36E-02	1.03E+01	1.03E+01
ENSG00000110619	CARS	1.22	1.22	0.28	1.41E-02	1.03E+01	1.03E+01
ENSG00000239665	RP11-295P9.3	0.75	-1.34	-0.42	1.71E-02	-1.03E+01	1.03E+01
ENSG00000126254	RBM42	1.21	1.21	0.27	1.39E-02	1.03E+01	1.03E+01
ENSG00000145916	RMND5B	0.82	-1.21	-0.28	1.41E-02	-1.02E+01	1.02E+01
ENSG00000147669	POLR2K	0.83	-1.21	-0.27	1.40E-02	-1.02E+01	1.02E+01
	RP11-89C21.2	1.32	1.32	0.40	1.68E-02	1.02E+01	1.02E+01
ENSG00000138326	RPS24	0.81	-1.24	-0.31	1.47E-02	-1.02E+01	1.02E+01
ENSG00000184634	MED12	0.77	-1.30	-0.38	1.63E-02	-1.02E+01	1.02E+01
ENSG00000172007	RAB33B	1.21	1.21	0.27	1.41E-02	1.02E+01	1.02E+01
ENSG00000197892	KIF13B	1.24	1.24	0.31	1.49E-02	1.01E+01	1.01E+01
ENSG00000158092	NCK1	1.22	1.22	0.29	1.45E-02	1.01E+01	1.01E+01
ENSG00000075142	SRI	0.82	-1.21	-0.28	1.43E-02	-1.01E+01	1.01E+01
ENSG00000164056	SPRY1	0.81	-1.24	-0.31	1.49E-02	-1.01E+01	1.01E+01
ENSG00000173875	ZNF791	1.20	1.20	0.27	1.41E-02	1.01E+01	1.01E+01
ENSG00000166452	AKIP1	0.83	-1.20	-0.27	1.41E-02	-1.01E+01	1.01E+01
ENSG00000172775	FAM192A	1.21	1.21	0.28	1.44E-02	1.01E+01	1.01E+01
ENSG00000105281	SLC1A5	0.81	-1.23	-0.30	1.48E-02	-1.01E+01	1.01E+01
ENSG00000197355	UAP1L1	1.20	1.20	0.26	1.42E-02	1.01E+01	1.01E+01
ENSG00000011198	ABHD5	0.83	-1.20	-0.27	1.42E-02	-1.01E+01	1.01E+01
ENSG00000120675	DNAJC15	0.82	-1.22	-0.28	1.46E-02	-1.01E+01	1.01E+01
ENSG00000018610	CXorf56	0.83	-1.20	-0.27	1.43E-02	-1.01E+01	1.01E+01
ENSG00000155111	CDK19	1.21	1.21	0.28	1.46E-02	1.00E+01	1.00E+01
ENSG00000122484	RPAP2	1.21	1.21	0.28	1.47E-02	1.00E+01	1.00E+01
ENSG00000079337	RAPGEF3	1.20	1.20	0.27	1.44E-02	1.00E+01	1.00E+01
ENSG00000180370	PAK2	0.81	-1.23	-0.30	1.50E-02	-1.00E+01	1.00E+01
ENSG00000005075	POLR2J	0.83	-1.21	-0.27	1.46E-02	-1.00E+01	1.00E+01
ENSG00000107290	SETX	1.34	1.34	0.42	1.79E-02	9.99E+00	9.99E+00
ENSG00000037241	RPL26L1	1.22	1.22	0.28	1.49E-02	9.99E+00	9.99E+00
ENSG00000171530	TBCA	0.82	-1.23	-0.29	1.51E-02	-9.99E+00	9.99E+00
ENSG00000178229	ZNF543	1.23	1.23	0.30	1.52E-02	9.98E+00	9.98E+00
ENSG00000197782	ZNF780A	0.80	-1.24	-0.32	1.56E-02	-9.98E+00	9.98E+00
ENSG00000128923	FAM63B	1.27	1.27	0.35	1.63E-02	9.96E+00	9.96E+00
ENSG00000130255	RPL36	0.81	-1.23	-0.30	1.52E-02	-9.96E+00	9.96E+00
ENSG00000145945	FAM50B	1.24	1.24	0.32	1.56E-02	9.96E+00	9.96E+00
ENSG00000060069	CTDP1	1.20	1.20	0.27	1.46E-02	9.95E+00	9.95E+00
ENSG00000168255	POLR2J3	0.80	-1.25	-0.33	1.59E-02	-9.94E+00	9.94E+00
ENSG00000204316	MRPL38	0.69	-1.44	-0.53	2.11E-02	-9.94E+00	9.94E+00
ENSG00000126777	KTN1	1.27	1.27	0.34	1.63E-02	9.93E+00	9.93E+00
ENSG00000163923	RPL39L	0.83	-1.20	-0.27	1.47E-02	-9.92E+00	9.92E+00
ENSG00000172354	GNB2	1.21	1.21	0.28	1.49E-02	9.92E+00	9.92E+00
ENSG00000198563	DDX39B	0.84	-1.20	-0.26	1.46E-02	-9.91E+00	9.91E+00
ENSG00000152219	ARL14EP	0.83	-1.21	-0.27	1.49E-02	-9.91E+00	9.91E+00
ENSG00000187678	SPRY4	1.24	1.24	0.31	1.56E-02	9.89E+00	9.89E+00
ENSG00000135506	OS9	1.22	1.22	0.28	1.52E-02	9.89E+00	9.89E+00
ENSG00000170759	KIF5B	1.32	1.32	0.40	1.79E-02	9.89E+00	9.89E+00
ENSG00000164961	KIAA0196	1.23	1.23	0.30	1.54E-02	9.88E+00	9.88E+00
ENSG00000148688	RPP30	0.84	-1.19	-0.26	1.46E-02	-9.88E+00	9.88E+00
ENSG00000138463	DIRC2	1.20	1.20	0.26	1.48E-02	9.86E+00	9.86E+00
ENSG00000145147	SLIT2	0.83	-1.21	-0.28	1.51E-02	-9.86E+00	9.86E+00
ENSG00000156521	TYSND1	0.80	-1.26	-0.33	1.63E-02	-9.85E+00	9.85E+00
ENSG00000270103	RNU11	0.76	-1.31	-0.39	1.76E-02	-9.85E+00	9.85E+00
ENSG00000081692	JMJD4	0.83	-1.20	-0.26	1.49E-02	-9.85E+00	9.85E+00
ENSG00000250479	CHCHD10	0.81	-1.23	-0.30	1.56E-02	-9.84E+00	9.84E+00
ENSG00000088179	PTPN4	1.25	1.25	0.32	1.61E-02	9.83E+00	9.83E+00
ENSG00000100220	C22orf28	0.83	-1.21	-0.27	1.51E-02	-9.82E+00	9.82E+00

ENSG00000197044	ZNF441	1.30	1.30	0.38	1.75E-02	9.81E+00	9.81E+00
ENSG00000159733	ZFYVE28	1.23	1.23	0.30	1.59E-02	9.80E+00	9.80E+00
ENSG00000101474	APMAP	1.21	1.21	0.27	1.52E-02	9.80E+00	9.80E+00
ENSG00000273048	MIR4720	0.82	-1.22	-0.29	1.56E-02	-9.78E+00	9.78E+00
ENSG00000106524	ANKMY2	0.82	-1.22	-0.29	1.56E-02	-9.78E+00	9.78E+00
ENSG00000198039	ZNF273	0.79	-1.27	-0.35	1.70E-02	-9.75E+00	9.75E+00
ENSG00000157978	LDLRAP1	0.83	-1.20	-0.27	1.52E-02	-9.74E+00	9.74E+00
ENSG00000148219	ASTN2	1.23	1.23	0.29	1.59E-02	9.73E+00	9.73E+00
ENSG00000072135	PTPN18	0.82	-1.22	-0.29	1.57E-02	-9.73E+00	9.73E+00
ENSG00000116906	GNPAT	1.21	1.21	0.27	1.55E-02	9.73E+00	9.73E+00
ENSG00000267248	CTD-2319I12.4	1.43	1.43	0.52	2.17E-02	9.72E+00	9.72E+00
ENSG00000155008	APOOL	1.29	1.29	0.36	1.75E-02	9.71E+00	9.71E+00
ENSG00000171016	PYGO1	0.81	-1.24	-0.31	1.63E-02	-9.70E+00	9.70E+00
ENSG00000175040	CHST2	0.80	-1.25	-0.32	1.66E-02	-9.70E+00	9.70E+00
ENSG00000160298	C21orf58	1.24	1.24	0.31	1.63E-02	9.70E+00	9.70E+00
ENSG00000064651	SLC12A2	1.24	1.24	0.31	1.64E-02	9.69E+00	9.69E+00
ENSG00000234616	JRK	0.83	-1.21	-0.28	1.56E-02	-9.69E+00	9.69E+00
ENSG00000129810	SGOL1	0.81	-1.23	-0.30	1.62E-02	-9.69E+00	9.69E+00
ENSG00000136158	SPRY2	1.21	1.21	0.27	1.55E-02	9.69E+00	9.69E+00
ENSG00000145730	PAM	1.22	1.22	0.29	1.59E-02	9.68E+00	9.68E+00
ENSG00000145685	LHFPL2	1.21	1.21	0.28	1.57E-02	9.66E+00	9.66E+00
ENSG00000100644	HIF1A	1.28	1.28	0.35	1.75E-02	9.66E+00	9.66E+00
ENSG00000089053	ANAPC5	0.83	-1.21	-0.27	1.57E-02	-9.66E+00	9.66E+00
ENSG00000093167	LRRFIP2	0.83	-1.21	-0.27	1.56E-02	-9.66E+00	9.66E+00
ENSG00000145375	SPATA5	0.83	-1.20	-0.26	1.55E-02	-9.64E+00	9.64E+00
ENSG00000175395	ZNF25	1.22	1.22	0.29	1.61E-02	9.63E+00	9.63E+00
ENSG00000178175	ZNF366	0.76	-1.31	-0.39	1.85E-02	-9.62E+00	9.62E+00
ENSG00000127337	YEATS4	0.84	-1.20	-0.26	1.55E-02	-9.61E+00	9.61E+00
ENSG00000141526	SLC16A3	0.83	-1.21	-0.27	1.57E-02	-9.61E+00	9.61E+00
ENSG00000004534	RBM6	0.83	-1.20	-0.26	1.56E-02	-9.61E+00	9.61E+00
ENSG00000107223	EDF1	0.82	-1.22	-0.28	1.60E-02	-9.61E+00	9.61E+00
ENSG00000155287	SLC25A28	0.82	-1.22	-0.29	1.61E-02	-9.60E+00	9.60E+00
ENSG00000173715	C11orf80	1.20	1.20	0.26	1.56E-02	9.60E+00	9.60E+00
ENSG00000014914	MTMR11	0.75	-1.33	-0.41	1.93E-02	-9.60E+00	9.60E+00
ENSG00000187098	MITF	1.27	1.27	0.34	1.75E-02	9.59E+00	9.59E+00
ENSG00000105656	ELL	1.27	1.27	0.34	1.75E-02	9.59E+00	9.59E+00
ENSG00000198522	GPN1	0.83	-1.20	-0.27	1.58E-02	-9.59E+00	9.59E+00
	CTD-2086O20.3	1.39	1.39	0.48	2.11E-02	9.59E+00	9.59E+00
ENSG00000239672	NME1	0.82	-1.21	-0.28	1.60E-02	-9.58E+00	9.58E+00
ENSG00000060138	CSDA	1.22	1.22	0.28	1.61E-02	9.58E+00	9.58E+00
ENSG00000224189	HOXD-AS1	1.21	1.21	0.27	1.59E-02	9.57E+00	9.57E+00
ENSG00000058729	RIOK2	0.82	-1.22	-0.29	1.62E-02	-9.57E+00	9.57E+00
ENSG00000224152	AC009506.1	1.31	1.31	0.39	1.88E-02	9.55E+00	9.55E+00
ENSG00000127870	RNF6	1.26	1.26	0.34	1.75E-02	9.55E+00	9.55E+00
ENSG00000100526	CDKN3	0.83	-1.21	-0.27	1.60E-02	-9.54E+00	9.54E+00
ENSG00000256407	AL357673.1	0.72	-1.38	-0.47	2.10E-02	-9.54E+00	9.54E+00
ENSG00000100242	SUN2	1.22	1.22	0.28	1.63E-02	9.53E+00	9.53E+00
ENSG00000133226	SRRM1	0.83	-1.20	-0.27	1.60E-02	-9.52E+00	9.52E+00
ENSG00000135723	FHOD1	1.25	1.25	0.32	1.71E-02	9.52E+00	9.52E+00
ENSG00000175130	MARCKSL1	0.83	-1.21	-0.27	1.61E-02	-9.52E+00	9.52E+00
ENSG00000213593	TMX2	0.83	-1.20	-0.26	1.59E-02	-9.52E+00	9.52E+00
ENSG00000198113	TOR4A	1.21	1.21	0.28	1.63E-02	9.51E+00	9.51E+00
ENSG00000106560	GIMAP2	0.81	-1.23	-0.30	1.67E-02	-9.50E+00	9.50E+00
ENSG00000125166	GOT2	1.30	1.30	0.38	1.87E-02	9.50E+00	9.50E+00
ENSG00000181666	HKR1	1.20	1.20	0.26	1.59E-02	9.49E+00	9.49E+00
ENSG00000182827	ACBD3	1.20	1.20	0.27	1.61E-02	9.49E+00	9.49E+00
ENSG00000188021	UBQLN2	1.22	1.22	0.28	1.64E-02	9.49E+00	9.49E+00
ENSG00000120662	MTRF1	1.22	1.22	0.29	1.66E-02	9.49E+00	9.49E+00
ENSG00000130304	SLC27A1	0.80	-1.25	-0.32	1.74E-02	-9.48E+00	9.48E+00
ENSG00000152484	USP12	1.22	1.22	0.29	1.67E-02	9.47E+00	9.47E+00
ENSG00000145545	SRD5A1	1.23	1.23	0.30	1.68E-02	9.46E+00	9.46E+00
ENSG00000111786	SRSF9	0.81	-1.23	-0.30	1.68E-02	-9.46E+00	9.46E+00
ENSG00000102967	DHODH	0.81	-1.23	-0.30	1.69E-02	-9.43E+00	9.43E+00
ENSG00000169914	OTUD3	0.82	-1.21	-0.28	1.66E-02	-9.42E+00	9.42E+00
ENSG00000104825	NFKBIB	1.22	1.22	0.29	1.68E-02	9.42E+00	9.42E+00
ENSG00000196449	YRDC	1.21	1.21	0.27	1.65E-02	9.42E+00	9.42E+00
ENSG00000267680	ZNF224	1.21	1.21	0.27	1.65E-02	9.41E+00	9.41E+00
ENSG00000043514	TRIT1	0.83	-1.20	-0.27	1.64E-02	-9.41E+00	9.41E+00
ENSG00000183309	ZNF623	1.20	1.20	0.26	1.62E-02	9.40E+00	9.40E+00
ENSG00000112941	PAPD7	1.21	1.21	0.28	1.67E-02	9.40E+00	9.40E+00
ENSG00000161914	ZNF653	1.37	1.37	0.46	2.13E-02	9.39E+00	9.39E+00
ENSG00000083845	RPS5	0.82	-1.21	-0.28	1.67E-02	-9.39E+00	9.39E+00
ENSG00000271303	SRXN1	1.21	1.21	0.27	1.65E-02	9.38E+00	9.38E+00
ENSG00000233183	RP3-468B3.2	1.40	1.40	0.48	2.22E-02	9.38E+00	9.38E+00
ENSG00000083896	YTHDC1	1.20	1.20	0.26	1.64E-02	9.38E+00	9.38E+00
ENSG00000075413	MARK3	1.20	1.20	0.27	1.65E-02	9.36E+00	9.36E+00
ENSG00000121274	PAPD5	1.19	1.19	0.26	1.63E-02	9.35E+00	9.35E+00
ENSG00000172409	CLP1	0.82	-1.22	-0.28	1.69E-02	-9.35E+00	9.35E+00
ENSG00000161999	JMJD8	0.82	-1.21	-0.28	1.69E-02	-9.34E+00	9.34E+00

ENSG00000101745	ANKRD12	0.82	-1.23	-0.29	1.73E-02	-9.33E+00	9.33E+00
ENSG00000093000	NUP50	0.83	-1.21	-0.27	1.67E-02	-9.33E+00	9.33E+00
ENSG00000108352	RAPGEFL1	0.71	-1.40	-0.49	2.27E-02	-9.32E+00	9.32E+00
ENSG00000028528	SNX1	0.83	-1.21	-0.28	1.69E-02	-9.30E+00	9.30E+00
ENSG00000075711	DLG1	0.83	-1.20	-0.27	1.68E-02	-9.29E+00	9.29E+00
ENSG00000004660	CAMKK1	0.78	-1.28	-0.35	1.90E-02	-9.26E+00	9.26E+00
ENSG00000136114	THSD1	1.19	1.19	0.25	1.66E-02	9.25E+00	9.25E+00
ENSG00000134531	EMP1	1.22	1.22	0.29	1.74E-02	9.25E+00	9.25E+00
ENSG00000178996	SNX18	1.21	1.21	0.28	1.71E-02	9.24E+00	9.24E+00
ENSG00000198176	TFDP1	0.82	-1.22	-0.29	1.74E-02	-9.24E+00	9.24E+00
ENSG00000196236	XPNPEP3	1.19	1.19	0.25	1.67E-02	9.24E+00	9.24E+00
ENSG00000099326	MZF1	1.24	1.24	0.31	1.81E-02	9.24E+00	9.24E+00
	RP11-571M6.6	0.82	-1.22	-0.29	1.75E-02	-9.23E+00	9.23E+00
ENSG00000143811	PYCR2	0.84	-1.19	-0.25	1.67E-02	-9.23E+00	9.23E+00
ENSG00000052723	SIKE1	0.83	-1.21	-0.27	1.71E-02	-9.22E+00	9.22E+00
ENSG00000149541	B3GAT3	0.83	-1.21	-0.28	1.73E-02	-9.22E+00	9.22E+00
ENSG00000182197	EXT1	1.21	1.21	0.27	1.71E-02	9.21E+00	9.21E+00
ENSG00000211460	TSN	0.83	-1.21	-0.28	1.73E-02	-9.21E+00	9.21E+00
ENSG00000117523	PRRC2C	1.22	1.22	0.28	1.74E-02	9.21E+00	9.21E+00
ENSG00000258920	FOXN3-AS1	1.38	1.38	0.47	2.26E-02	9.20E+00	9.20E+00
	RP11-322M19.1	0.83	-1.20	-0.26	1.70E-02	-9.19E+00	9.19E+00
ENSG00000072310	SREBF1	1.20	1.20	0.27	1.72E-02	9.18E+00	9.18E+00
ENSG00000100280	AP1B1	1.23	1.23	0.30	1.80E-02	9.18E+00	9.18E+00
ENSG00000170390	DCLK2	1.31	1.31	0.39	2.03E-02	9.17E+00	9.17E+00
ENSG00000181090	EHMT1	1.21	1.21	0.27	1.74E-02	9.17E+00	9.17E+00
ENSG00000167528	ZNF641	0.78	-1.28	-0.35	1.94E-02	-9.17E+00	9.17E+00
ENSG00000010803	SCMH1	0.83	-1.20	-0.26	1.71E-02	-9.16E+00	9.16E+00
ENSG00000089280	FUS	0.82	-1.22	-0.29	1.78E-02	-9.15E+00	9.15E+00
ENSG00000171466	ZNF562	0.84	-1.19	-0.25	1.70E-02	-9.14E+00	9.14E+00
ENSG00000106009	BRAT1	1.21	1.21	0.27	1.75E-02	9.13E+00	9.13E+00
ENSG00000115935	WIPF1	1.22	1.22	0.29	1.79E-02	9.12E+00	9.12E+00
ENSG00000125170	DOK4	0.83	-1.20	-0.27	1.74E-02	-9.12E+00	9.12E+00
ENSG00000203727	SAMD5	0.83	-1.20	-0.26	1.74E-02	-9.11E+00	9.11E+00
ENSG00000234444	ZNF736	0.76	-1.31	-0.39	2.07E-02	-9.09E+00	9.09E+00
ENSG00000187566	NHLRC1	1.34	1.34	0.42	2.17E-02	9.08E+00	9.08E+00
ENSG00000007047	MARK4	1.21	1.21	0.28	1.78E-02	9.07E+00	9.07E+00
ENSG00000130489	SCO2	0.82	-1.22	-0.28	1.80E-02	-9.07E+00	9.07E+00
ENSG00000134014	ELP3	0.84	-1.19	-0.25	1.73E-02	-9.07E+00	9.07E+00
ENSG00000213516	RBMXL1	0.83	-1.20	-0.26	1.76E-02	-9.06E+00	9.06E+00
ENSG00000090447	TFAP4	0.80	-1.26	-0.33	1.92E-02	-9.06E+00	9.06E+00
ENSG00000188171	ZNF626	1.25	1.25	0.32	1.89E-02	9.06E+00	9.06E+00
ENSG00000118503	TNFAIP3	1.33	1.33	0.41	2.16E-02	9.04E+00	9.04E+00
ENSG00000167553	TUBA1C	0.83	-1.21	-0.28	1.79E-02	-9.04E+00	9.04E+00
ENSG00000173113	TRMT112	0.83	-1.21	-0.27	1.79E-02	-9.03E+00	9.03E+00
ENSG00000066084	DIP2B	1.21	1.21	0.28	1.80E-02	9.03E+00	9.03E+00
ENSG00000229152	ANKRD10-IT1	0.76	-1.32	-0.40	2.14E-02	-9.03E+00	9.03E+00
ENSG00000217555	CKLF	0.82	-1.22	-0.28	1.82E-02	-9.02E+00	9.02E+00
ENSG00000084774	CAD	0.79	-1.26	-0.34	1.97E-02	-8.99E+00	8.99E+00
ENSG00000131845	ZNF304	1.20	1.20	0.27	1.79E-02	8.99E+00	8.99E+00
ENSG00000160993	ALKBH4	0.81	-1.23	-0.30	1.89E-02	-8.98E+00	8.98E+00
ENSG00000137193	PIM1	0.81	-1.23	-0.30	1.88E-02	-8.97E+00	8.97E+00
ENSG00000158161	EYA3	0.84	-1.19	-0.25	1.77E-02	-8.97E+00	8.97E+00
ENSG00000135720	DYNC1LI2	1.21	1.21	0.27	1.81E-02	8.96E+00	8.96E+00
ENSG00000119711	ALDH6A1	0.84	-1.19	-0.26	1.78E-02	-8.95E+00	8.95E+00
ENSG00000164574	GALNT10	1.21	1.21	0.27	1.83E-02	8.93E+00	8.93E+00
ENSG00000125841	NRSN2	0.83	-1.20	-0.27	1.82E-02	-8.92E+00	8.92E+00
ENSG00000203485	INF2	1.20	1.20	0.26	1.80E-02	8.92E+00	8.92E+00
ENSG00000256223	ZNF10	1.32	1.32	0.40	2.19E-02	8.92E+00	8.92E+00
ENSG00000157510	AFAP1L1	1.21	1.21	0.28	1.84E-02	8.92E+00	8.92E+00
ENSG00000144736	SHQ1	0.84	-1.20	-0.26	1.80E-02	-8.91E+00	8.91E+00
ENSG00000049759	NEDD4L	1.21	1.21	0.28	1.85E-02	8.90E+00	8.90E+00
ENSG00000139549	DHH	1.32	1.32	0.41	2.22E-02	8.89E+00	8.89E+00
ENSG00000113716	HMGXB3	1.20	1.20	0.26	1.82E-02	8.89E+00	8.89E+00
ENSG00000141337	ARSG	0.78	-1.28	-0.36	2.07E-02	-8.89E+00	8.89E+00
ENSG00000074695	LMAN1	1.25	1.25	0.33	2.00E-02	8.86E+00	8.86E+00
ENSG00000110090	CPT1A	0.82	-1.22	-0.29	1.91E-02	-8.86E+00	8.86E+00
ENSG00000140391	TSPAN3	1.21	1.21	0.27	1.86E-02	8.85E+00	8.85E+00
	CTD-2339L15.1	1.33	1.33	0.41	2.25E-02	8.85E+00	8.85E+00
ENSG00000178567	EPM2AIP1	1.20	1.20	0.27	1.86E-02	8.84E+00	8.84E+00
ENSG00000159256	MORC3	1.23	1.23	0.30	1.95E-02	8.82E+00	8.82E+00
ENSG00000117153	KLHL12	0.84	-1.19	-0.25	1.83E-02	-8.80E+00	8.80E+00
ENSG00000146067	FAM193B	0.84	-1.20	-0.26	1.85E-02	-8.79E+00	8.79E+00
ENSG00000090565	RAB11FIP3	0.84	-1.19	-0.25	1.83E-02	-8.79E+00	8.79E+00
ENSG00000144867	SRPRB	0.83	-1.20	-0.26	1.87E-02	-8.79E+00	8.79E+00
ENSG00000116957	TBCE	0.84	-1.20	-0.26	1.86E-02	-8.79E+00	8.79E+00
ENSG00000198301	SDAD1	1.20	1.20	0.27	1.87E-02	8.79E+00	8.79E+00
ENSG00000059145	UNKL	1.22	1.22	0.28	1.91E-02	8.78E+00	8.78E+00
ENSG00000125826	RBCK1	1.19	1.19	0.25	1.85E-02	8.76E+00	8.76E+00
ENSG00000116525	TRIM62	1.35	1.35	0.43	2.38E-02	8.75E+00	8.75E+00

ENSG00000105245	NUMBL	1.23	1.23	0.30	2.00E-02	8.74E+00	8.74E+00
ENSG00000105429	MEGF8	1.21	1.21	0.27	1.91E-02	8.74E+00	8.74E+00
ENSG00000178338	ZNF354B	1.21	1.21	0.27	1.90E-02	8.74E+00	8.74E+00
ENSG00000242802	AP5Z1	1.19	1.19	0.25	1.87E-02	8.73E+00	8.73E+00
ENSG00000108010	GLRX3	1.20	1.20	0.26	1.89E-02	8.73E+00	8.73E+00
ENSG00000080986	NDC80	0.82	-1.22	-0.29	1.95E-02	-8.73E+00	8.73E+00
ENSG00000165121	RP11-213G2.3	0.71	-1.40	-0.48	2.58E-02	-8.72E+00	8.72E+00
ENSG00000112531	QKI	1.20	1.20	0.27	1.91E-02	8.71E+00	8.71E+00
ENSG00000062716	VMP1	1.22	1.22	0.28	1.95E-02	8.71E+00	8.71E+00
ENSG00000118507	AKAP7	0.79	-1.27	-0.34	2.11E-02	-8.71E+00	8.71E+00
ENSG00000258301	RP11-488C13.5	1.35	1.35	0.44	2.42E-02	8.71E+00	8.71E+00
ENSG00000180626	ZNF594	1.25	1.25	0.33	2.07E-02	8.71E+00	8.71E+00
ENSG00000134809	TIMM10	0.83	-1.20	-0.27	1.91E-02	-8.71E+00	8.71E+00
ENSG00000100271	TTLL1	1.23	1.23	0.29	1.99E-02	8.69E+00	8.69E+00
ENSG00000228801	RP11-110G21.1	1.46	1.46	0.54	2.82E-02	8.68E+00	8.68E+00
ENSG00000126858	RHOT1	0.84	-1.19	-0.25	1.88E-02	-8.68E+00	8.68E+00
ENSG00000126775	ATG14	1.19	1.19	0.25	1.88E-02	8.68E+00	8.68E+00
ENSG00000171723	GPHN	0.81	-1.24	-0.31	2.03E-02	-8.68E+00	8.68E+00
ENSG00000112079	STK38	1.19	1.19	0.25	1.90E-02	8.66E+00	8.66E+00
ENSG00000152475	ZNF837	1.38	1.38	0.46	2.52E-02	8.66E+00	8.66E+00
ENSG00000168314	MOBP	1.41	1.41	0.49	2.65E-02	8.65E+00	8.65E+00
ENSG00000117650	NEK2	0.81	-1.24	-0.31	2.05E-02	-8.64E+00	8.64E+00
ENSG00000215630	GUSBP9	1.40	1.40	0.49	2.63E-02	8.63E+00	8.63E+00
ENSG00000086758	HUWE1	1.25	1.25	0.32	2.09E-02	8.62E+00	8.62E+00
ENSG00000176890	TYMS	0.81	-1.24	-0.31	2.07E-02	-8.61E+00	8.61E+00
ENSG00000185189	NRBP2	0.80	-1.24	-0.32	2.09E-02	-8.61E+00	8.61E+00
ENSG00000072682	P4HA2	1.20	1.20	0.26	1.94E-02	8.61E+00	8.61E+00
ENSG00000197124	ZNF682	0.77	-1.29	-0.37	2.26E-02	-8.60E+00	8.60E+00
ENSG00000122203	KIAA1191	1.20	1.20	0.26	1.94E-02	8.60E+00	8.60E+00
ENSG00000107021	TBC1D13	0.83	-1.20	-0.26	1.95E-02	-8.58E+00	8.58E+00
ENSG00000152382	TADA1	0.83	-1.21	-0.27	1.99E-02	-8.57E+00	8.57E+00
ENSG00000214870	AC004540.5	0.74	-1.36	-0.44	2.51E-02	-8.56E+00	8.56E+00
ENSG00000127952	STYXL1	0.84	-1.19	-0.26	1.94E-02	-8.56E+00	8.56E+00
ENSG00000138829	FBN2	1.26	1.26	0.33	2.17E-02	8.53E+00	8.53E+00
ENSG00000100802	C14orf93	1.24	1.24	0.30	2.10E-02	8.52E+00	8.52E+00
ENSG00000134802	SLC43A3	1.20	1.20	0.26	1.97E-02	8.52E+00	8.52E+00
ENSG00000088766	CRLS1	0.84	-1.19	-0.25	1.95E-02	-8.51E+00	8.51E+00
ENSG00000100239	PPP6R2	0.84	-1.19	-0.25	1.97E-02	-8.50E+00	8.50E+00
ENSG00000152439	ZNF773	1.26	1.26	0.33	2.20E-02	8.50E+00	8.50E+00
ENSG00000258441	LINC00641	0.82	-1.21	-0.28	2.04E-02	-8.48E+00	8.48E+00
ENSG00000162298	SYVN1	1.25	1.25	0.32	2.17E-02	8.47E+00	8.47E+00
ENSG00000178385	PLEKHM3	1.25	1.25	0.32	2.19E-02	8.46E+00	8.46E+00
ENSG00000204520	MICA	0.82	-1.22	-0.28	2.07E-02	-8.46E+00	8.46E+00
	RP11-383J24.5	1.34	1.34	0.42	2.51E-02	8.45E+00	8.45E+00
ENSG00000102738	MRPS31	0.84	-1.19	-0.25	2.00E-02	-8.44E+00	8.44E+00
ENSG00000114861	FOXP1	0.83	-1.21	-0.27	2.05E-02	-8.43E+00	8.43E+00
ENSG00000090621	PABPC4	1.21	1.21	0.27	2.05E-02	8.42E+00	8.42E+00
ENSG00000130175	PRKCSH	1.33	1.33	0.42	2.52E-02	8.41E+00	8.41E+00
ENSG00000129347	KRI1	0.82	-1.22	-0.29	2.10E-02	-8.41E+00	8.41E+00
ENSG00000111667	USP5	0.83	-1.20	-0.26	2.04E-02	-8.41E+00	8.41E+00
ENSG00000132840	BHMT2	0.68	-1.46	-0.55	3.03E-02	-8.40E+00	8.40E+00
ENSG00000085978	ATG16L1	1.19	1.19	0.25	2.01E-02	8.40E+00	8.40E+00
ENSG00000089693	MLF2	0.84	-1.19	-0.25	2.01E-02	-8.39E+00	8.39E+00
ENSG00000171603	CLSTN1	1.22	1.22	0.29	2.12E-02	8.39E+00	8.39E+00
ENSG00000111206	FOXM1	1.27	1.27	0.34	2.28E-02	8.38E+00	8.38E+00
ENSG00000123684	LPGAT1	1.20	1.20	0.26	2.05E-02	8.38E+00	8.38E+00
ENSG00000153885	KCTD15	0.84	-1.18	-0.24	1.99E-02	-8.38E+00	8.38E+00
ENSG00000100813	ACIN1	0.83	-1.20	-0.26	2.06E-02	-8.38E+00	8.38E+00
ENSG00000157764	BRAF	1.20	1.20	0.26	2.04E-02	8.38E+00	8.38E+00
ENSG00000100281	HMGXB4	0.83	-1.20	-0.26	2.06E-02	-8.37E+00	8.37E+00
ENSG00000105173	CCNE1	0.83	-1.20	-0.26	2.06E-02	-8.36E+00	8.36E+00
ENSG00000164506	STXBP5	1.19	1.19	0.25	2.02E-02	8.36E+00	8.36E+00
ENSG00000187109	NAP1L1	0.83	-1.20	-0.27	2.08E-02	-8.36E+00	8.36E+00
ENSG00000157379	DHRS1	0.80	-1.25	-0.32	2.23E-02	-8.35E+00	8.35E+00
ENSG00000011243	AKAP8L	1.19	1.19	0.26	2.05E-02	8.34E+00	8.34E+00
ENSG00000204256	BRD2	1.20	1.20	0.26	2.06E-02	8.34E+00	8.34E+00
ENSG00000087842	PIR	1.20	1.20	0.27	2.09E-02	8.33E+00	8.33E+00
ENSG00000168675	LDLRAD4	1.33	1.33	0.41	2.56E-02	8.33E+00	8.33E+00
ENSG00000109320	NFKB1	0.84	-1.20	-0.26	2.07E-02	-8.33E+00	8.33E+00
ENSG00000158483	FAM86C1	1.29	1.29	0.37	2.40E-02	8.31E+00	8.31E+00
ENSG00000086598	TMED2	0.82	-1.21	-0.28	2.14E-02	-8.31E+00	8.31E+00
ENSG00000224597	RP11-534G20.3	1.19	1.19	0.25	2.04E-02	8.31E+00	8.31E+00
ENSG00000184058	TBX1	1.31	1.31	0.39	2.47E-02	8.31E+00	8.31E+00
ENSG00000175701	LINC00116	0.83	-1.21	-0.27	2.12E-02	-8.30E+00	8.30E+00
ENSG00000151176	PLBD2	1.24	1.24	0.30	2.22E-02	8.30E+00	8.30E+00
ENSG00000122642	FKBP9	1.19	1.19	0.25	2.07E-02	8.29E+00	8.29E+00
ENSG00000092929	UNC13D	1.40	1.40	0.48	2.85E-02	8.28E+00	8.28E+00
ENSG00000082641	NFE2L1	1.20	1.20	0.26	2.10E-02	8.27E+00	8.27E+00
ENSG00000140332	TLE3	1.21	1.21	0.27	2.14E-02	8.26E+00	8.26E+00

ENSG00000101407	TTI1	0.83	-1.20	-0.26	2.11E-02	-8.26E+00	8.26E+00
ENSG00000169621	APLF	1.25	1.25	0.32	2.28E-02	8.25E+00	8.25E+00
ENSG00000115540	MOB4	1.20	1.20	0.27	2.13E-02	8.25E+00	8.25E+00
ENSG00000128652	HOXD3	0.81	-1.23	-0.30	2.23E-02	-8.22E+00	8.22E+00
ENSG00000115020	PIKFYVE	1.21	1.21	0.28	2.17E-02	8.21E+00	8.21E+00
ENSG00000124299	PEPD	1.20	1.20	0.26	2.12E-02	8.21E+00	8.21E+00
ENSG00000143256	PFDN2	0.84	-1.18	-0.24	2.08E-02	-8.21E+00	8.21E+00
ENSG00000197818	SLC9A8	1.21	1.21	0.28	2.19E-02	8.20E+00	8.20E+00
ENSG00000132432	SEC61G	0.78	-1.28	-0.35	2.43E-02	-8.20E+00	8.20E+00
ENSG00000189042	ZNF567	1.22	1.22	0.29	2.23E-02	8.20E+00	8.20E+00
ENSG00000134186	PRPF38B	0.83	-1.20	-0.27	2.16E-02	-8.19E+00	8.19E+00
ENSG00000164292	RHOBTB3	1.20	1.20	0.26	2.14E-02	8.19E+00	8.19E+00
ENSG00000109475	RPL34	0.82	-1.22	-0.29	2.23E-02	-8.19E+00	8.19E+00
ENSG00000204498	NFKBIL1	1.21	1.21	0.27	2.17E-02	8.19E+00	8.19E+00
ENSG00000100567	PSMA3	0.84	-1.20	-0.26	2.14E-02	-8.17E+00	8.17E+00
ENSG00000129084	PSMA1	0.83	-1.20	-0.27	2.18E-02	-8.15E+00	8.15E+00
ENSG00000187815	ZNF642	0.80	-1.26	-0.33	2.37E-02	-8.15E+00	8.15E+00
ENSG00000115204	MPV17	1.19	1.19	0.25	2.13E-02	8.15E+00	8.15E+00
ENSG00000233565	AC007690.1	0.74	-1.36	-0.44	2.78E-02	-8.14E+00	8.14E+00
ENSG00000124523	SIRT5	1.20	1.20	0.26	2.16E-02	8.14E+00	8.14E+00
ENSG00000059804	SLC2A3	1.19	1.19	0.25	2.14E-02	8.13E+00	8.13E+00
ENSG00000164134	NAA15	1.24	1.24	0.31	2.33E-02	8.13E+00	8.13E+00
ENSG00000164296	TIGD6	1.23	1.23	0.30	2.29E-02	8.12E+00	8.12E+00
ENSG00000115896	PLCL1	1.26	1.26	0.34	2.43E-02	8.11E+00	8.11E+00
ENSG00000075643	MOCOS	1.31	1.31	0.39	2.59E-02	8.11E+00	8.11E+00
ENSG00000237440	ZNF737	1.24	1.24	0.32	2.36E-02	8.10E+00	8.10E+00
ENSG00000156876	SASS6	0.80	-1.25	-0.32	2.37E-02	-8.09E+00	8.09E+00
ENSG00000171222	SCAND1	1.19	1.19	0.25	2.16E-02	8.08E+00	8.08E+00
ENSG00000142207	URB1	0.82	-1.23	-0.29	2.30E-02	-8.08E+00	8.08E+00
ENSG00000134452	FBXO18	1.21	1.21	0.27	2.23E-02	8.08E+00	8.08E+00
ENSG00000165821	SALL2	0.75	-1.33	-0.41	2.71E-02	-8.08E+00	8.08E+00
ENSG00000129250	KIF1C	0.83	-1.20	-0.26	2.20E-02	-8.08E+00	8.08E+00
	RP3-341D10.4	0.74	-1.36	-0.44	2.83E-02	-8.07E+00	8.07E+00
ENSG00000110400	PVRL1	1.24	1.24	0.31	2.37E-02	8.07E+00	8.07E+00
ENSG00000170027	YWHAG	1.20	1.20	0.27	2.23E-02	8.07E+00	8.07E+00
ENSG00000064995	TAF11	0.84	-1.19	-0.25	2.17E-02	-8.07E+00	8.07E+00
ENSG00000174109	C16orf91	0.82	-1.22	-0.29	2.29E-02	-8.07E+00	8.07E+00
ENSG00000163788	SNRK	1.20	1.20	0.27	2.22E-02	8.06E+00	8.06E+00
ENSG00000006576	PHTF2	1.20	1.20	0.27	2.23E-02	8.06E+00	8.06E+00
ENSG00000175470	PPP2R2D	1.19	1.19	0.25	2.19E-02	8.05E+00	8.05E+00
ENSG00000259985	RP11-549B18.1	1.36	1.36	0.44	2.85E-02	8.04E+00	8.04E+00
ENSG00000147853	AK3	0.83	-1.20	-0.26	2.23E-02	-8.04E+00	8.04E+00
ENSG00000100139	MICALL1	1.20	1.20	0.27	2.25E-02	8.03E+00	8.03E+00
ENSG00000105778	AVL9	1.19	1.19	0.26	2.21E-02	8.03E+00	8.03E+00
ENSG00000215548	RP11-764K9.4	0.73	-1.36	-0.44	2.88E-02	-8.02E+00	8.02E+00
ENSG00000127914	AKAP9	1.25	1.25	0.32	2.43E-02	8.02E+00	8.02E+00
ENSG00000044574	HSPA5	0.78	-1.28	-0.36	2.56E-02	-8.02E+00	8.02E+00
ENSG00000180787	ZFP3	0.80	-1.24	-0.31	2.40E-02	-8.02E+00	8.02E+00
ENSG00000198843	SELT	1.20	1.20	0.26	2.22E-02	8.01E+00	8.01E+00
ENSG00000103540	CCP110	1.26	1.26	0.33	2.47E-02	8.01E+00	8.01E+00
ENSG00000130119	GNL3L	0.85	-1.18	-0.24	2.18E-02	-8.00E+00	8.00E+00
ENSG00000106785	TRIM14	0.81	-1.23	-0.30	2.36E-02	-8.00E+00	8.00E+00
	RP11-122G18.5	1.30	1.30	0.37	2.64E-02	7.98E+00	7.98E+00
ENSG00000196235	SUPT5H	1.20	1.20	0.27	2.27E-02	7.98E+00	7.98E+00
ENSG00000152795	HNRPDL	0.83	-1.20	-0.26	2.28E-02	-7.95E+00	7.95E+00
ENSG00000131467	PSME3	0.84	-1.20	-0.26	2.26E-02	-7.95E+00	7.95E+00
ENSG00000173848	NET1	0.82	-1.22	-0.29	2.36E-02	-7.95E+00	7.95E+00
ENSG00000110660	SLC35F2	1.20	1.20	0.26	2.27E-02	7.95E+00	7.95E+00
ENSG00000113966	ARL6	0.79	-1.26	-0.33	2.51E-02	-7.95E+00	7.95E+00
ENSG00000204519	ZNF551	1.22	1.22	0.28	2.34E-02	7.94E+00	7.94E+00
ENSG00000188033	ZNF490	1.30	1.30	0.38	2.69E-02	7.94E+00	7.94E+00
ENSG00000129158	SERGEF	0.79	-1.27	-0.35	2.58E-02	-7.93E+00	7.93E+00
ENSG00000137504	CREBZF	0.84	-1.19	-0.26	2.27E-02	-7.92E+00	7.92E+00
ENSG00000109189	USP46	1.19	1.19	0.25	2.25E-02	7.92E+00	7.92E+00
ENSG00000183963	SMTN	1.24	1.24	0.31	2.45E-02	7.92E+00	7.92E+00
ENSG00000125652	ALKBH7	0.82	-1.22	-0.29	2.39E-02	-7.92E+00	7.92E+00
ENSG00000188227	ZNF793	1.34	1.34	0.42	2.85E-02	7.91E+00	7.91E+00
ENSG00000135049	AGTPBP1	0.84	-1.19	-0.25	2.26E-02	-7.91E+00	7.91E+00
ENSG00000137764	MAP2K5	1.25	1.25	0.32	2.48E-02	7.91E+00	7.91E+00
ENSG00000182774	RPS17	0.67	-1.50	-0.58	3.59E-02	-7.91E+00	7.91E+00
ENSG00000096746	HNRNPH3	1.19	1.19	0.25	2.27E-02	7.90E+00	7.90E+00
ENSG00000065613	SLK	1.32	1.32	0.40	2.77E-02	7.90E+00	7.90E+00
ENSG00000155438	MKI67IP	0.84	-1.19	-0.25	2.27E-02	-7.89E+00	7.89E+00
ENSG00000133561	GIMAP6	0.84	-1.20	-0.26	2.30E-02	-7.88E+00	7.88E+00
ENSG00000109572	CLCN3	1.20	1.20	0.26	2.32E-02	7.87E+00	7.87E+00
ENSG00000214274	ANG	0.75	-1.34	-0.42	2.91E-02	-7.87E+00	7.87E+00
ENSG00000196440	ARMCX4	0.81	-1.24	-0.31	2.48E-02	-7.87E+00	7.87E+00
ENSG00000223705	NSUN5P1	0.77	-1.30	-0.38	2.74E-02	-7.86E+00	7.86E+00
ENSG00000100731	PCNX	1.19	1.19	0.25	2.30E-02	7.86E+00	7.86E+00

ENSG00000166444	ST5	0.77	-1.29	-0.37	2.72E-02	-7.85E+00	7.85E+00
ENSG00000154229	PRKCA	1.18	1.18	0.24	2.26E-02	7.85E+00	7.85E+00
ENSG00000138413	IDH1	0.83	-1.20	-0.27	2.36E-02	-7.84E+00	7.84E+00
ENSG00000260822	GS1-358P8.4	0.79	-1.26	-0.34	2.60E-02	-7.84E+00	7.84E+00
ENSG00000147121	ZNF673	1.21	1.21	0.27	2.38E-02	7.84E+00	7.84E+00
ENSG00000139651	ZNF740	0.84	-1.19	-0.25	2.31E-02	-7.83E+00	7.83E+00
ENSG00000145990	GFOD1	0.84	-1.20	-0.26	2.34E-02	-7.82E+00	7.82E+00
ENSG00000091483	FH	0.84	-1.20	-0.26	2.34E-02	-7.81E+00	7.81E+00
ENSG00000133961	NUMB	1.23	1.23	0.30	2.48E-02	7.81E+00	7.81E+00
ENSG00000097007	ABL1	1.24	1.24	0.32	2.55E-02	7.80E+00	7.80E+00
ENSG00000205309	NT5M	1.38	1.38	0.46	3.13E-02	7.80E+00	7.80E+00
ENSG00000134138	MEIS2	1.19	1.19	0.25	2.34E-02	7.79E+00	7.79E+00
ENSG00000156110	ADK	0.84	-1.19	-0.26	2.35E-02	-7.79E+00	7.79E+00
ENSG00000185129	PURA	1.21	1.21	0.28	2.43E-02	7.78E+00	7.78E+00
ENSG00000196369	SRGAP2B	1.20	1.20	0.26	2.37E-02	7.78E+00	7.78E+00
	AC092295.7	1.31	1.31	0.39	2.82E-02	7.78E+00	7.78E+00
ENSG00000125352	RNF113A	1.20	1.20	0.26	2.38E-02	7.78E+00	7.78E+00
ENSG00000077254	USP33	1.20	1.20	0.26	2.38E-02	7.77E+00	7.77E+00
ENSG00000174886	NDUFA11	0.83	-1.21	-0.28	2.43E-02	-7.77E+00	7.77E+00
ENSG00000166526	ZNF3	1.18	1.18	0.24	2.32E-02	7.76E+00	7.76E+00
ENSG00000100532	CGRRF1	1.20	1.20	0.26	2.38E-02	7.76E+00	7.76E+00
ENSG00000124279	FASTKD3	0.83	-1.20	-0.27	2.40E-02	-7.76E+00	7.76E+00
ENSG00000185238	PRMT3	0.85	-1.18	-0.24	2.32E-02	-7.75E+00	7.75E+00
ENSG00000173597	SULT1B1	0.80	-1.24	-0.32	2.58E-02	-7.75E+00	7.75E+00
ENSG00000134453	RBM17	0.84	-1.19	-0.25	2.37E-02	-7.74E+00	7.74E+00
ENSG00000184402	SS18L1	1.22	1.22	0.28	2.47E-02	7.74E+00	7.74E+00
ENSG00000175899	A2M	0.70	-1.42	-0.51	3.37E-02	-7.73E+00	7.73E+00
ENSG00000178467	P4HTM	1.21	1.21	0.27	2.44E-02	7.73E+00	7.73E+00
ENSG00000134569	LRP4	0.73	-1.38	-0.46	3.18E-02	-7.71E+00	7.71E+00
ENSG00000188807	TMEM201	0.81	-1.23	-0.30	2.54E-02	-7.70E+00	7.70E+00
ENSG00000119772	DNMT3A	0.84	-1.18	-0.24	2.37E-02	-7.69E+00	7.69E+00
ENSG00000123353	ORMDL2	0.84	-1.18	-0.24	2.37E-02	-7.69E+00	7.69E+00
ENSG00000001631	KRIT1	1.20	1.20	0.27	2.46E-02	7.67E+00	7.67E+00
ENSG00000184428	TOP1MT	0.83	-1.21	-0.27	2.48E-02	-7.67E+00	7.67E+00
ENSG00000127948	POR	1.30	1.30	0.38	2.88E-02	7.65E+00	7.65E+00
ENSG00000128694	OSGEPL1	0.83	-1.21	-0.27	2.49E-02	-7.65E+00	7.65E+00
ENSG00000047315	POLR2B	1.20	1.20	0.26	2.44E-02	7.65E+00	7.65E+00
ENSG00000140740	UQCRC2	0.84	-1.19	-0.26	2.44E-02	-7.64E+00	7.64E+00
ENSG00000138606	SHF	0.76	-1.32	-0.40	2.99E-02	-7.64E+00	7.64E+00
	AC004463.6	0.74	-1.35	-0.43	3.12E-02	-7.64E+00	7.64E+00
ENSG00000184863	RBM33	1.19	1.19	0.25	2.41E-02	7.64E+00	7.64E+00
ENSG00000132275	RRP8	0.85	-1.18	-0.24	2.40E-02	-7.63E+00	7.63E+00
ENSG00000173218	VANGL1	1.19	1.19	0.25	2.43E-02	7.63E+00	7.63E+00
ENSG00000065665	SEC61A2	1.25	1.25	0.33	2.71E-02	7.62E+00	7.62E+00
ENSG00000106638	TBL2	1.18	1.18	0.24	2.42E-02	7.62E+00	7.62E+00
ENSG00000198189	HSD17B11	0.85	-1.18	-0.24	2.40E-02	-7.61E+00	7.61E+00
ENSG00000108854	SMURF2	1.20	1.20	0.26	2.47E-02	7.61E+00	7.61E+00
ENSG00000114626	ABTB1	0.74	-1.36	-0.44	3.18E-02	-7.61E+00	7.61E+00
ENSG00000172292	CERS6	0.84	-1.20	-0.26	2.48E-02	-7.59E+00	7.59E+00
ENSG00000188732	FAM221A	0.79	-1.26	-0.33	2.75E-02	-7.59E+00	7.59E+00
ENSG00000149212	SESN3	0.81	-1.24	-0.31	2.66E-02	-7.59E+00	7.59E+00
ENSG00000204713	TRIM27	1.19	1.19	0.25	2.47E-02	7.59E+00	7.59E+00
ENSG00000114942	EEF1B2	0.83	-1.21	-0.27	2.54E-02	-7.58E+00	7.58E+00
ENSG00000042317	SPATA7	1.22	1.22	0.29	2.61E-02	7.58E+00	7.58E+00
ENSG00000172456	FGGY	1.26	1.26	0.33	2.77E-02	7.55E+00	7.55E+00
ENSG00000188215	DCUN1D3	1.19	1.19	0.25	2.48E-02	7.55E+00	7.55E+00
ENSG00000099968	BCL2L13	1.19	1.19	0.25	2.48E-02	7.55E+00	7.55E+00
ENSG00000266962	RP11-400F19.6	0.83	-1.20	-0.27	2.54E-02	-7.54E+00	7.54E+00
ENSG00000182809	CRIP2	1.19	1.19	0.25	2.50E-02	7.54E+00	7.54E+00
ENSG00000119574	ZBTB45	1.28	1.28	0.35	2.87E-02	7.53E+00	7.53E+00
ENSG00000065559	MAP2K4	1.18	1.18	0.24	2.47E-02	7.52E+00	7.52E+00
ENSG00000072110	ACTN1	1.28	1.28	0.35	2.89E-02	7.51E+00	7.51E+00
ENSG00000165948	IFI27L1	0.83	-1.21	-0.27	2.59E-02	-7.51E+00	7.51E+00
ENSG00000115380	EFEMP1	0.83	-1.20	-0.26	2.56E-02	-7.51E+00	7.51E+00
ENSG00000168610	STAT3	1.20	1.20	0.26	2.54E-02	7.51E+00	7.51E+00
ENSG00000171160	MORN4	0.80	-1.26	-0.33	2.80E-02	-7.50E+00	7.50E+00
ENSG00000184635	ZNF93	0.81	-1.23	-0.30	2.72E-02	-7.49E+00	7.49E+00
ENSG00000131871	VIMP	0.83	-1.20	-0.27	2.58E-02	-7.49E+00	7.49E+00
ENSG00000121211	MND1	0.81	-1.24	-0.31	2.74E-02	-7.48E+00	7.48E+00
ENSG00000132122	SPATA6	0.78	-1.28	-0.35	2.92E-02	-7.48E+00	7.48E+00
ENSG00000128609	NDUFA5	0.83	-1.20	-0.26	2.58E-02	-7.48E+00	7.48E+00
ENSG00000181061	HIGD1A	1.20	1.20	0.26	2.57E-02	7.47E+00	7.47E+00
ENSG00000155906	RMND1	0.84	-1.19	-0.25	2.55E-02	-7.47E+00	7.47E+00
ENSG00000068137	PLEKHH3	1.25	1.25	0.32	2.81E-02	7.46E+00	7.46E+00
ENSG00000100083	GGA1	1.18	1.18	0.24	2.51E-02	7.45E+00	7.45E+00
ENSG00000183060	LYSMD4	0.79	-1.26	-0.33	2.87E-02	-7.45E+00	7.45E+00
ENSG00000196683	TOMM7	0.82	-1.23	-0.29	2.71E-02	-7.44E+00	7.44E+00
ENSG00000091986	CCDC80	1.21	1.21	0.28	2.66E-02	7.43E+00	7.43E+00
ENSG00000132128	LRRC41	0.84	-1.20	-0.26	2.59E-02	-7.43E+00	7.43E+00

ENSG00000197283	SYNGAP1	1.32	1.32	0.40	3.15E-02	7.42E+00	7.42E+00
ENSG00000205581	HMGN1	0.84	-1.20	-0.26	2.60E-02	-7.42E+00	7.42E+00
ENSG00000114331	ACAP2	1.23	1.23	0.29	2.73E-02	7.41E+00	7.41E+00
ENSG00000151445	VIPAS39	0.85	-1.18	-0.24	2.53E-02	-7.41E+00	7.41E+00
ENSG00000137807	KIF23	0.81	-1.23	-0.30	2.74E-02	-7.41E+00	7.41E+00
ENSG00000176903	PNMA1	0.84	-1.19	-0.25	2.58E-02	-7.41E+00	7.41E+00
ENSG00000185730	ZNF696	0.80	-1.25	-0.32	2.83E-02	-7.41E+00	7.41E+00
ENSG00000157259	GATAD1	1.18	1.18	0.24	2.53E-02	7.40E+00	7.40E+00
ENSG00000198265	HELZ	1.19	1.19	0.25	2.59E-02	7.39E+00	7.39E+00
ENSG00000175581	MRPL48	0.85	-1.18	-0.23	2.53E-02	-7.39E+00	7.39E+00
ENSG00000143450	OAZ3	1.36	1.36	0.45	3.41E-02	7.39E+00	7.39E+00
ENSG00000187764	SEMA4D	1.31	1.31	0.39	3.15E-02	7.38E+00	7.38E+00
ENSG00000170471	RALGAPB	1.19	1.19	0.26	2.62E-02	7.38E+00	7.38E+00
ENSG00000267321	RP11-1094M14.11	0.81	-1.23	-0.30	2.80E-02	-7.38E+00	7.38E+00
ENSG00000185650	ZFP36L1	1.19	1.19	0.26	2.62E-02	7.37E+00	7.37E+00
ENSG00000204576	PRR3	0.83	-1.21	-0.27	2.68E-02	-7.37E+00	7.37E+00
ENSG00000196396	PTPN1	1.20	1.20	0.27	2.67E-02	7.37E+00	7.37E+00
ENSG00000169118	CSNK1G1	1.18	1.18	0.24	2.59E-02	7.36E+00	7.36E+00
ENSG00000159377	PSMB4	0.84	-1.20	-0.26	2.66E-02	-7.34E+00	7.34E+00
ENSG00000186615	KTN1-AS1	0.75	-1.34	-0.42	3.35E-02	-7.34E+00	7.34E+00
ENSG00000268895	A1BG-AS1	0.78	-1.29	-0.37	3.09E-02	-7.34E+00	7.34E+00
ENSG00000163820	FYCO1	1.21	1.21	0.28	2.74E-02	7.33E+00	7.33E+00
	RP11-357N13.7	1.35	1.35	0.44	3.41E-02	7.33E+00	7.33E+00
ENSG00000130396	MLLT4	1.19	1.19	0.25	2.64E-02	7.32E+00	7.32E+00
ENSG00000104960	PTOV1	1.19	1.19	0.25	2.62E-02	7.32E+00	7.32E+00
ENSG00000180902	D2HGDH	1.22	1.22	0.29	2.81E-02	7.31E+00	7.31E+00
ENSG00000138107	ACTR1A	0.84	-1.19	-0.25	2.66E-02	-7.31E+00	7.31E+00
ENSG00000244405	ETV5	1.21	1.21	0.28	2.78E-02	7.28E+00	7.28E+00
ENSG00000154370	TRIM11	1.20	1.20	0.26	2.70E-02	7.28E+00	7.28E+00
	RP11-622K12.1	0.85	-1.18	-0.24	2.63E-02	-7.28E+00	7.28E+00
ENSG00000178343	SHISA3	0.82	-1.22	-0.28	2.80E-02	-7.27E+00	7.27E+00
ENSG00000133619	KRBA1	1.23	1.23	0.30	2.86E-02	7.27E+00	7.27E+00
ENSG00000141030	COPS3	0.84	-1.19	-0.25	2.67E-02	-7.26E+00	7.26E+00
ENSG00000256185	RP11-612B6.2	1.35	1.35	0.43	3.47E-02	7.25E+00	7.25E+00
ENSG00000129675	ARHGEF6	0.83	-1.20	-0.26	2.74E-02	-7.25E+00	7.25E+00
ENSG00000143761	ARF1	1.20	1.20	0.26	2.72E-02	7.25E+00	7.25E+00
ENSG00000115902	SLC1A4	0.82	-1.22	-0.29	2.83E-02	-7.24E+00	7.24E+00
ENSG00000092203	TOX4	1.18	1.18	0.24	2.67E-02	7.24E+00	7.24E+00
ENSG00000178078	STAP2	0.80	-1.25	-0.32	2.98E-02	-7.24E+00	7.24E+00
ENSG00000134262	AP4B1	0.85	-1.18	-0.24	2.65E-02	-7.23E+00	7.23E+00
ENSG00000161547	SRSF2	0.84	-1.19	-0.25	2.72E-02	-7.23E+00	7.23E+00
ENSG00000076662	ICAM3	1.25	1.25	0.32	2.97E-02	7.23E+00	7.23E+00
ENSG00000130348	QRSL1	0.85	-1.18	-0.23	2.65E-02	-7.22E+00	7.22E+00
ENSG00000170854	MINA	0.85	-1.18	-0.24	2.67E-02	-7.22E+00	7.22E+00
ENSG00000072274	TFRC	0.84	-1.19	-0.25	2.72E-02	-7.22E+00	7.22E+00
ENSG00000175592	FOSL1	1.26	1.26	0.34	3.07E-02	7.22E+00	7.22E+00
ENSG00000078304	PPP2R5C	1.19	1.19	0.25	2.72E-02	7.22E+00	7.22E+00
ENSG00000158792	SPATA2L	0.79	-1.27	-0.35	3.11E-02	-7.21E+00	7.21E+00
ENSG00000128408	RIBC2	1.33	1.33	0.41	3.40E-02	7.21E+00	7.21E+00
ENSG00000119523	ALG2	1.18	1.18	0.24	2.69E-02	7.21E+00	7.21E+00
ENSG00000204790	CBWD6	0.76	-1.31	-0.39	3.33E-02	-7.20E+00	7.20E+00
ENSG00000166922	SCG5	0.71	-1.41	-0.49	3.81E-02	-7.20E+00	7.20E+00
ENSG00000133612	AGAP3	1.18	1.18	0.24	2.68E-02	7.19E+00	7.19E+00
ENSG00000131196	NFATC1	1.22	1.22	0.28	2.86E-02	7.19E+00	7.19E+00
ENSG00000111321	LTBR	0.81	-1.23	-0.30	2.94E-02	-7.19E+00	7.19E+00
ENSG00000256683	ZNF350	1.24	1.24	0.31	2.98E-02	7.18E+00	7.18E+00
ENSG00000111652	COPS7A	0.85	-1.18	-0.24	2.71E-02	-7.18E+00	7.18E+00
ENSG00000183723	CMTM4	0.83	-1.21	-0.27	2.84E-02	-7.17E+00	7.17E+00
ENSG00000266074	BAHCC1	1.26	1.26	0.33	3.10E-02	7.16E+00	7.16E+00
ENSG00000176340	COX8A	0.84	-1.20	-0.26	2.80E-02	-7.15E+00	7.15E+00
ENSG00000100836	PABPN1	0.84	-1.19	-0.26	2.79E-02	-7.15E+00	7.15E+00
ENSG00000167461	RAB8A	0.84	-1.18	-0.24	2.74E-02	-7.15E+00	7.15E+00
ENSG00000144560	VGLL4	1.21	1.21	0.27	2.85E-02	7.15E+00	7.15E+00
ENSG00000186479	RGS7BP	0.79	-1.26	-0.34	3.12E-02	-7.15E+00	7.15E+00
ENSG00000207445	SNORD15B	0.73	-1.37	-0.45	3.66E-02	-7.14E+00	7.14E+00
ENSG00000144451	SPAG16	0.83	-1.21	-0.27	2.85E-02	-7.14E+00	7.14E+00
ENSG00000134982	APC	1.38	1.38	0.46	3.73E-02	7.12E+00	7.12E+00
ENSG00000109738	GLRB	0.81	-1.24	-0.31	3.04E-02	-7.11E+00	7.11E+00
ENSG00000112304	ACOT13	0.85	-1.18	-0.24	2.75E-02	-7.11E+00	7.11E+00
ENSG00000167526	RPL13	0.84	-1.20	-0.26	2.84E-02	-7.10E+00	7.10E+00
ENSG00000167562	ZNF701	1.21	1.21	0.28	2.92E-02	7.09E+00	7.09E+00
ENSG00000220370	RP3-399J4.2	0.73	-1.37	-0.45	3.74E-02	-7.09E+00	7.09E+00
ENSG00000176401	EID2B	0.78	-1.28	-0.35	3.25E-02	-7.08E+00	7.08E+00
ENSG00000130684	ZNF337	1.17	1.17	0.23	2.75E-02	7.08E+00	7.08E+00
ENSG00000042429	MED17	1.19	1.19	0.25	2.80E-02	7.08E+00	7.08E+00
ENSG00000166912	MTMR10	0.84	-1.19	-0.25	2.81E-02	-7.08E+00	7.08E+00
ENSG00000269955	LUC7L2	0.85	-1.18	-0.24	2.79E-02	-7.07E+00	7.07E+00
	RP11-1136G11.7	0.73	-1.36	-0.45	3.71E-02	-7.07E+00	7.07E+00
ENSG00000142784	WDTC1	1.20	1.20	0.26	2.88E-02	7.06E+00	7.06E+00

ENSG00000070961	ATP2B1	0.82	-1.23	-0.30	3.02E-02	-7.06E+00	7.06E+00
ENSG00000083168	KAT6A	1.18	1.18	0.24	2.81E-02	7.06E+00	7.06E+00
ENSG00000166619	BLCAP	0.85	-1.18	-0.24	2.80E-02	-7.05E+00	7.05E+00
ENSG00000139734	DIAPH3	0.84	-1.19	-0.25	2.86E-02	-7.04E+00	7.04E+00
ENSG00000101052	IFT52	0.84	-1.19	-0.25	2.84E-02	-7.04E+00	7.04E+00
ENSG00000144224	UBXN4	1.25	1.25	0.32	3.14E-02	7.03E+00	7.03E+00
ENSG00000145592	RPL37	0.82	-1.22	-0.29	3.04E-02	-7.02E+00	7.02E+00
ENSG00000147649	MTDH	1.19	1.19	0.25	2.87E-02	7.02E+00	7.02E+00
ENSG00000009413	REV3L	1.38	1.38	0.46	3.86E-02	7.01E+00	7.01E+00
ENSG00000180806	HOXC9	1.38	1.38	0.47	3.89E-02	7.00E+00	7.00E+00
ENSG00000107249	GLIS3	1.33	1.33	0.42	3.64E-02	6.99E+00	6.99E+00
ENSG00000196937	FAM3C	1.18	1.18	0.24	2.86E-02	6.99E+00	6.99E+00
ENSG00000142173	COL6A2	1.35	1.35	0.43	3.71E-02	6.98E+00	6.98E+00
ENSG00000268205	CTC-444N24.11	1.23	1.23	0.30	3.12E-02	6.98E+00	6.98E+00
ENSG00000137492	PRKRIR	0.85	-1.18	-0.24	2.87E-02	-6.97E+00	6.97E+00
ENSG00000136492	BRIP1	0.80	-1.25	-0.33	3.24E-02	-6.96E+00	6.96E+00
ENSG00000171522	PTGER4	1.20	1.20	0.26	2.96E-02	6.96E+00	6.96E+00
ENSG00000115042	FAHD2A	0.84	-1.19	-0.25	2.91E-02	-6.96E+00	6.96E+00
ENSG00000186298	PPP1CC	0.82	-1.21	-0.28	3.04E-02	-6.96E+00	6.96E+00
ENSG00000168040	FADD	1.18	1.18	0.24	2.90E-02	6.96E+00	6.96E+00
ENSG00000109111	SUPT6H	1.19	1.19	0.25	2.91E-02	6.96E+00	6.96E+00
ENSG00000119977	TCTN3	1.18	1.18	0.24	2.89E-02	6.96E+00	6.96E+00
ENSG00000051382	PIK3CB	1.18	1.18	0.24	2.87E-02	6.95E+00	6.95E+00
ENSG00000135362	PRR5L	1.22	1.22	0.28	3.06E-02	6.95E+00	6.95E+00
ENSG00000205485	AC004980.7	0.75	-1.34	-0.42	3.71E-02	-6.94E+00	6.94E+00
ENSG00000105325	FZR1	1.20	1.20	0.27	3.01E-02	6.94E+00	6.94E+00
ENSG00000149269	PAK1	0.83	-1.20	-0.26	2.98E-02	-6.94E+00	6.94E+00
ENSG00000213020	ZNF611	1.21	1.21	0.27	3.02E-02	6.94E+00	6.94E+00
ENSG00000119878	CRIPT	0.82	-1.22	-0.28	3.08E-02	-6.94E+00	6.94E+00
ENSG00000151239	TWF1	1.22	1.22	0.28	3.08E-02	6.93E+00	6.93E+00
ENSG00000136160	EDNRB	0.81	-1.23	-0.30	3.15E-02	-6.93E+00	6.93E+00
ENSG00000129295	LRRC6	1.24	1.24	0.31	3.18E-02	6.93E+00	6.93E+00
ENSG00000160285	LSS	0.83	-1.20	-0.26	3.00E-02	-6.92E+00	6.92E+00
ENSG00000109971	HSPA8	0.84	-1.19	-0.25	2.97E-02	-6.92E+00	6.92E+00
ENSG00000158169	FANCC	0.82	-1.21	-0.28	3.07E-02	-6.92E+00	6.92E+00
ENSG00000198752	CDC42BPB	1.21	1.21	0.27	3.06E-02	6.92E+00	6.92E+00
ENSG00000180694	TMEM64	1.19	1.19	0.25	2.96E-02	6.91E+00	6.91E+00
ENSG00000025800	KPNA6	0.84	-1.19	-0.26	2.99E-02	-6.91E+00	6.91E+00
ENSG00000142227	EMP3	0.84	-1.19	-0.25	2.97E-02	-6.91E+00	6.91E+00
ENSG00000110330	BIRC2	1.20	1.20	0.26	3.01E-02	6.90E+00	6.90E+00
ENSG00000166130	IKBIP	1.19	1.19	0.25	2.97E-02	6.89E+00	6.89E+00
ENSG00000163399	ATP1A1	1.30	1.30	0.38	3.58E-02	6.89E+00	6.89E+00
ENSG00000196642	RABL6	0.84	-1.18	-0.24	2.95E-02	-6.89E+00	6.89E+00
ENSG00000184857	TMEM186	0.80	-1.24	-0.32	3.26E-02	-6.89E+00	6.89E+00
ENSG00000145022	TCTA	0.83	-1.20	-0.27	3.06E-02	-6.88E+00	6.88E+00
	AC103965.1	0.83	-1.21	-0.27	3.09E-02	-6.88E+00	6.88E+00
ENSG00000105520	LPPR2	1.29	1.29	0.37	3.54E-02	6.87E+00	6.87E+00
ENSG00000144048	DUSP11	0.85	-1.18	-0.24	2.95E-02	-6.87E+00	6.87E+00
ENSG00000090905	TNRC6A	0.85	-1.18	-0.24	2.96E-02	-6.87E+00	6.87E+00
ENSG00000170955	PRKCDBP	1.19	1.19	0.25	3.02E-02	6.87E+00	6.87E+00
ENSG00000137075	RNF38	1.18	1.18	0.24	2.94E-02	6.87E+00	6.87E+00
ENSG00000172590	MRPL52	0.82	-1.21	-0.28	3.12E-02	-6.86E+00	6.86E+00
ENSG00000086619	ERO1LB	1.24	1.24	0.31	3.27E-02	6.86E+00	6.86E+00
ENSG00000125967	NECAB3	0.80	-1.25	-0.33	3.35E-02	-6.85E+00	6.85E+00
ENSG00000139168	ZCRB1	1.18	1.18	0.24	2.99E-02	6.84E+00	6.84E+00
ENSG00000124198	ARFGEF2	1.19	1.19	0.25	3.03E-02	6.84E+00	6.84E+00
	CTA-211A9.5	0.73	-1.37	-0.45	4.01E-02	-6.84E+00	6.84E+00
ENSG00000102010	BMX	1.20	1.20	0.26	3.09E-02	6.83E+00	6.83E+00
ENSG00000107077	KDM4C	1.18	1.18	0.24	2.98E-02	6.83E+00	6.83E+00
ENSG00000100316	RPL3	0.81	-1.24	-0.31	3.30E-02	-6.83E+00	6.83E+00
ENSG00000188242	CTD-2228K2.5	0.82	-1.23	-0.29	3.23E-02	-6.82E+00	6.82E+00
ENSG00000099282	TSPAN15	0.85	-1.17	-0.23	2.96E-02	-6.82E+00	6.82E+00
ENSG00000160223	ICOSLG	1.30	1.30	0.38	3.63E-02	6.81E+00	6.81E+00
ENSG00000111640	GAPDH	0.83	-1.20	-0.27	3.12E-02	-6.81E+00	6.81E+00
ENSG00000164024	METAP1	0.85	-1.18	-0.24	3.02E-02	-6.81E+00	6.81E+00
ENSG00000082438	COBLL1	1.21	1.21	0.28	3.16E-02	6.81E+00	6.81E+00
ENSG00000047849	MAP4	1.23	1.23	0.30	3.25E-02	6.81E+00	6.81E+00
ENSG00000114473	IQCG	1.26	1.26	0.34	3.45E-02	6.80E+00	6.80E+00
ENSG00000138768	USO1	1.25	1.25	0.32	3.40E-02	6.79E+00	6.79E+00
ENSG00000130830	MPP1	1.17	1.17	0.23	2.98E-02	6.79E+00	6.79E+00
ENSG00000205078	SYCE1L	0.75	-1.33	-0.41	3.82E-02	-6.79E+00	6.79E+00
ENSG00000164338	UTP15	0.85	-1.17	-0.23	3.00E-02	-6.78E+00	6.78E+00
ENSG00000119421	NDUFA8	1.19	1.19	0.25	3.08E-02	6.78E+00	6.78E+00
ENSG00000166123	GPT2	1.18	1.18	0.24	3.05E-02	6.76E+00	6.76E+00
ENSG00000130414	NDUFA10	0.85	-1.18	-0.24	3.06E-02	-6.75E+00	6.75E+00
ENSG00000125247	TMTC4	1.17	1.17	0.23	3.03E-02	6.75E+00	6.75E+00
ENSG00000062485	CS	0.84	-1.20	-0.26	3.14E-02	-6.74E+00	6.74E+00
ENSG00000198911	SREBF2	0.85	-1.18	-0.23	3.04E-02	-6.74E+00	6.74E+00
	AL132709.5	1.18	1.18	0.24	3.08E-02	6.74E+00	6.74E+00

ENSG00000184384	MAML2	1.20	1.20	0.27	3.20E-02	6.74E+00	6.74E+00
ENSG00000154556	SORBS2	0.83	-1.20	-0.27	3.20E-02	-6.74E+00	6.74E+00
ENSG00000173226	IQCB1	0.85	-1.18	-0.23	3.06E-02	-6.72E+00	6.72E+00
ENSG00000228989	AC133528.2	0.75	-1.33	-0.41	3.94E-02	-6.71E+00	6.71E+00
ENSG00000242588	RP11-274B21.1	1.28	1.28	0.36	3.65E-02	6.71E+00	6.71E+00
ENSG00000164035	EMCN	0.80	-1.25	-0.32	3.47E-02	-6.71E+00	6.71E+00
ENSG00000159079	C21orf59	0.77	-1.30	-0.38	3.78E-02	-6.71E+00	6.71E+00
ENSG00000168505	GBX2	1.34	1.34	0.43	4.02E-02	6.71E+00	6.71E+00
ENSG00000119684	MLH3	1.20	1.20	0.26	3.20E-02	6.70E+00	6.70E+00
ENSG00000167657	DAPK3	1.19	1.19	0.25	3.17E-02	6.69E+00	6.69E+00
	RP11-732M18.3	1.29	1.29	0.37	3.72E-02	6.69E+00	6.69E+00
ENSG00000196204	RNF216P1	0.84	-1.19	-0.25	3.17E-02	-6.68E+00	6.68E+00
ENSG00000137486	ARRB1	1.21	1.21	0.27	3.27E-02	6.67E+00	6.67E+00
ENSG00000122483	CCDC18	1.20	1.20	0.26	3.23E-02	6.67E+00	6.67E+00
ENSG00000243660	ZNF487P	1.30	1.30	0.38	3.78E-02	6.67E+00	6.67E+00
ENSG00000137414	FAM8A1	0.85	-1.18	-0.23	3.11E-02	-6.67E+00	6.67E+00
ENSG00000137509	PRCP	1.20	1.20	0.26	3.23E-02	6.66E+00	6.66E+00
ENSG00000214026	MRPL23	1.17	1.17	0.23	3.10E-02	6.65E+00	6.65E+00
ENSG00000237190	CDKN2AIPNL	0.85	-1.17	-0.23	3.10E-02	-6.65E+00	6.65E+00
ENSG00000138376	BARD1	0.85	-1.17	-0.23	3.10E-02	-6.65E+00	6.65E+00
ENSG00000095002	MSH2	0.84	-1.19	-0.26	3.22E-02	-6.65E+00	6.65E+00
ENSG00000188681	TEKT4P2	1.23	1.23	0.30	3.43E-02	6.65E+00	6.65E+00
ENSG00000102081	FMR1	1.18	1.18	0.24	3.16E-02	6.65E+00	6.65E+00
ENSG00000080947	CROCCP3	0.78	-1.28	-0.35	3.69E-02	-6.64E+00	6.64E+00
ENSG00000021762	OSBPL5	1.20	1.20	0.26	3.27E-02	6.64E+00	6.64E+00
ENSG00000180398	MCFD2	1.18	1.18	0.24	3.18E-02	6.63E+00	6.63E+00
ENSG00000185697	MYBL1	1.20	1.20	0.27	3.29E-02	6.63E+00	6.63E+00
ENSG00000104812	GYS1	0.84	-1.19	-0.25	3.23E-02	-6.62E+00	6.62E+00
ENSG00000135404	CD63	1.20	1.20	0.26	3.26E-02	6.62E+00	6.62E+00
ENSG00000060971	ACAA1	0.82	-1.23	-0.29	3.44E-02	-6.62E+00	6.62E+00
ENSG00000133247	SUV420H2	1.28	1.28	0.36	3.75E-02	6.61E+00	6.61E+00
ENSG00000179152	TCAIM	1.19	1.19	0.25	3.23E-02	6.61E+00	6.61E+00
ENSG00000253710	ALG11	0.82	-1.22	-0.29	3.43E-02	-6.61E+00	6.61E+00
ENSG00000012963	UBR7	0.85	-1.18	-0.24	3.19E-02	-6.61E+00	6.61E+00
ENSG00000135365	PHF21A	1.19	1.19	0.25	3.25E-02	6.61E+00	6.61E+00
ENSG00000140939	NOL3	0.79	-1.26	-0.34	3.65E-02	-6.60E+00	6.60E+00
ENSG00000106462	EZH2	0.84	-1.19	-0.25	3.27E-02	-6.60E+00	6.60E+00
ENSG00000166963	MAP1A	0.79	-1.27	-0.34	3.70E-02	-6.60E+00	6.60E+00
ENSG00000161298	ZNF382	1.24	1.24	0.31	3.52E-02	6.60E+00	6.60E+00
ENSG00000167566	NCKAP5L	1.18	1.18	0.24	3.20E-02	6.59E+00	6.59E+00
ENSG00000155959	VBP1	0.84	-1.19	-0.26	3.28E-02	-6.59E+00	6.59E+00
ENSG00000184076	UQCR10	0.83	-1.20	-0.26	3.31E-02	-6.59E+00	6.59E+00
ENSG00000022277	RTFDC1	0.85	-1.18	-0.24	3.21E-02	-6.59E+00	6.59E+00
ENSG00000120798	NR2C1	0.84	-1.19	-0.25	3.26E-02	-6.58E+00	6.58E+00
ENSG00000167113	COQ4	0.85	-1.18	-0.24	3.21E-02	-6.58E+00	6.58E+00
ENSG00000116161	CACYBP	0.85	-1.18	-0.24	3.23E-02	-6.57E+00	6.57E+00
ENSG00000133302	ANKRD32	0.81	-1.24	-0.31	3.56E-02	-6.57E+00	6.57E+00
ENSG00000110057	UNC93B1	1.24	1.24	0.31	3.57E-02	6.56E+00	6.56E+00
ENSG00000055070	SZRD1	1.26	1.26	0.33	3.69E-02	6.56E+00	6.56E+00
ENSG00000227345	PARG	1.18	1.18	0.24	3.26E-02	6.56E+00	6.56E+00
ENSG00000146386	ABRACL	1.21	1.21	0.28	3.42E-02	6.56E+00	6.56E+00
ENSG00000160785	SLC25A44	0.85	-1.17	-0.23	3.20E-02	-6.55E+00	6.55E+00
ENSG00000164162	ANAPC10	0.85	-1.18	-0.24	3.27E-02	-6.54E+00	6.54E+00
ENSG00000247572	CTC-281B15.1	0.75	-1.33	-0.41	4.11E-02	-6.54E+00	6.54E+00
ENSG00000276966	HIST1H4E	0.81	-1.24	-0.31	3.57E-02	-6.54E+00	6.54E+00
ENSG00000253797	UTP14C	1.18	1.18	0.23	3.23E-02	6.54E+00	6.54E+00
ENSG00000146872	TLK2	1.17	1.17	0.23	3.23E-02	6.53E+00	6.53E+00
ENSG00000129925	TMEM8A	0.85	-1.18	-0.24	3.27E-02	-6.53E+00	6.53E+00
ENSG00000225663	FAM195B	1.22	1.22	0.29	3.49E-02	6.53E+00	6.53E+00
ENSG00000060749	QSER1	1.22	1.22	0.28	3.48E-02	6.53E+00	6.53E+00
ENSG00000137145	DENND4C	1.22	1.22	0.29	3.50E-02	6.53E+00	6.53E+00
ENSG00000265688	MAFG-AS1	1.34	1.34	0.42	4.22E-02	6.52E+00	6.52E+00
ENSG00000198162	MAN1A2	1.19	1.19	0.25	3.34E-02	6.50E+00	6.50E+00
ENSG00000088970	PLK1S1	1.23	1.23	0.30	3.59E-02	6.49E+00	6.49E+00
ENSG00000188243	COMMD6	1.20	1.20	0.27	3.44E-02	6.49E+00	6.49E+00
ENSG00000105197	TIMM50	0.85	-1.17	-0.23	3.26E-02	-6.48E+00	6.48E+00
ENSG00000092020	PPP2R3C	0.83	-1.21	-0.27	3.47E-02	-6.48E+00	6.48E+00
ENSG00000167671	UBXN6	0.85	-1.17	-0.23	3.29E-02	-6.48E+00	6.48E+00
ENSG00000160908	ZNF394	1.17	1.17	0.23	3.29E-02	6.47E+00	6.47E+00
ENSG00000165185	KIAA1958	0.77	-1.29	-0.37	3.99E-02	-6.47E+00	6.47E+00
ENSG00000112651	MRPL2	0.84	-1.18	-0.24	3.36E-02	-6.46E+00	6.46E+00
ENSG00000232112	TMA7	0.82	-1.21	-0.28	3.53E-02	-6.46E+00	6.46E+00
ENSG00000256771	ZNF253	1.22	1.22	0.29	3.57E-02	6.46E+00	6.46E+00
ENSG00000101972	STAG2	1.27	1.27	0.34	3.86E-02	6.46E+00	6.46E+00
ENSG00000102781	KATNAL1	1.18	1.18	0.24	3.34E-02	6.45E+00	6.45E+00
ENSG00000183765	CHEK2	0.85	-1.17	-0.23	3.31E-02	-6.45E+00	6.45E+00
ENSG00000165915	SLC39A13	1.17	1.17	0.23	3.30E-02	6.45E+00	6.45E+00
ENSG00000025772	TOMM34	0.84	-1.19	-0.25	3.42E-02	-6.45E+00	6.45E+00
ENSG00000272047	GTF2H5	1.18	1.18	0.24	3.34E-02	6.45E+00	6.45E+00

ENSG00000141873	SLC39A3	0.83	-1.20	-0.27	3.48E-02	-6.44E+00	6.44E+00
ENSG00000008294	SPAG9	1.18	1.18	0.24	3.36E-02	6.44E+00	6.44E+00
ENSG00000155304	HSPA13	1.28	1.28	0.35	3.93E-02	6.44E+00	6.44E+00
ENSG00000173530	TNFRSF10D	1.18	1.18	0.24	3.37E-02	6.44E+00	6.44E+00
ENSG00000025293	PHF20	0.85	-1.18	-0.24	3.39E-02	-6.43E+00	6.43E+00
ENSG00000080603	SRCAP	1.18	1.18	0.23	3.36E-02	6.42E+00	6.42E+00
ENSG00000109911	ELP4	0.85	-1.18	-0.23	3.35E-02	-6.42E+00	6.42E+00
ENSG00000161021	MAML1	0.83	-1.21	-0.28	3.56E-02	-6.42E+00	6.42E+00
ENSG00000054983	GALC	1.17	1.17	0.23	3.33E-02	6.41E+00	6.41E+00
ENSG00000189159	HN1	1.18	1.18	0.24	3.40E-02	6.41E+00	6.41E+00
ENSG00000125741	OPA3	0.85	-1.18	-0.23	3.37E-02	-6.41E+00	6.41E+00
ENSG00000179119	SPTY2D1	1.17	1.17	0.23	3.35E-02	6.40E+00	6.40E+00
ENSG00000137409	MTCH1	1.18	1.18	0.24	3.41E-02	6.40E+00	6.40E+00
ENSG00000167257	RNF214	1.23	1.23	0.30	3.72E-02	6.39E+00	6.39E+00
ENSG00000167380	ZNF226	0.85	-1.18	-0.23	3.38E-02	-6.39E+00	6.39E+00
ENSG00000189180	ZNF33A	1.23	1.23	0.29	3.68E-02	6.39E+00	6.39E+00
ENSG00000124193	SRSF6	0.85	-1.18	-0.24	3.43E-02	-6.38E+00	6.38E+00
ENSG00000196428	TSC22D2	1.17	1.17	0.23	3.38E-02	6.38E+00	6.38E+00
ENSG00000141905	NFIC	1.19	1.19	0.25	3.46E-02	6.38E+00	6.38E+00
ENSG00000165792	METTL17	0.85	-1.17	-0.23	3.37E-02	-6.37E+00	6.37E+00
ENSG00000167202	TBC1D2B	1.18	1.18	0.24	3.41E-02	6.37E+00	6.37E+00
ENSG00000143436	MRPL9	1.18	1.18	0.24	3.41E-02	6.37E+00	6.37E+00
ENSG00000260916	CCPG1	0.83	-1.21	-0.28	3.61E-02	-6.37E+00	6.37E+00
ENSG00000170734	POLH	1.22	1.22	0.29	3.66E-02	6.37E+00	6.37E+00
ENSG00000163444	TMEM183A	0.85	-1.18	-0.24	3.44E-02	-6.37E+00	6.37E+00
ENSG00000221886	C5orf54	0.79	-1.27	-0.34	3.95E-02	-6.36E+00	6.36E+00
ENSG00000122566	HNRNPA2B1	0.80	-1.26	-0.33	3.90E-02	-6.36E+00	6.36E+00
ENSG00000270066	SCARNA2	0.79	-1.26	-0.34	3.93E-02	-6.36E+00	6.36E+00
ENSG00000145390	USP53	1.25	1.25	0.32	3.83E-02	6.36E+00	6.36E+00
ENSG00000011523	CEP68	0.86	-1.17	-0.23	3.38E-02	-6.36E+00	6.36E+00
ENSG00000277161	PIGW	0.82	-1.22	-0.29	3.67E-02	-6.36E+00	6.36E+00
ENSG00000134644	PUM1	1.18	1.18	0.24	3.44E-02	6.35E+00	6.35E+00
ENSG00000135835	KIAA1614	0.77	-1.30	-0.38	4.17E-02	-6.35E+00	6.35E+00
ENSG00000136754	ABI1	1.18	1.18	0.23	3.43E-02	6.35E+00	6.35E+00
ENSG00000077235	GTF3C1	1.21	1.21	0.28	3.66E-02	6.35E+00	6.35E+00
ENSG00000177600	RPLP2	0.83	-1.20	-0.27	3.60E-02	-6.34E+00	6.34E+00
ENSG00000173511	VEGFB	1.20	1.20	0.27	3.61E-02	6.34E+00	6.34E+00
	RP11-181C21.6	0.80	-1.25	-0.33	3.92E-02	-6.34E+00	6.34E+00
ENSG00000120337	TNFSF18	0.82	-1.22	-0.28	3.69E-02	-6.33E+00	6.33E+00
ENSG00000087460	GNAS	1.19	1.19	0.25	3.54E-02	6.33E+00	6.33E+00
ENSG00000143418	CERS2	1.21	1.21	0.27	3.65E-02	6.33E+00	6.33E+00
ENSG00000187391	MAGI2	1.29	1.29	0.37	4.16E-02	6.33E+00	6.33E+00
ENSG00000143429	AC027612.6	1.24	1.24	0.31	3.86E-02	6.32E+00	6.32E+00
ENSG00000117122	MFAP2	1.18	1.18	0.24	3.51E-02	6.31E+00	6.31E+00
ENSG00000124596	OARD1	1.18	1.18	0.24	3.50E-02	6.30E+00	6.30E+00
ENSG00000259345	RP11-624L4.1	1.35	1.35	0.43	4.57E-02	6.29E+00	6.29E+00
ENSG00000147862	NFIB	1.19	1.19	0.25	3.56E-02	6.29E+00	6.29E+00
ENSG00000163029	SMC6	1.21	1.21	0.27	3.67E-02	6.29E+00	6.29E+00
ENSG00000104221	BRF2	0.84	-1.19	-0.25	3.59E-02	-6.29E+00	6.29E+00
ENSG00000122435	TRMT13	1.21	1.21	0.28	3.73E-02	6.29E+00	6.29E+00
ENSG00000269051	CTD-2245F17.3	1.33	1.33	0.41	4.48E-02	6.29E+00	6.29E+00
ENSG00000148484	RSU1	1.18	1.18	0.24	3.52E-02	6.29E+00	6.29E+00
ENSG00000132604	TERF2	1.20	1.20	0.27	3.66E-02	6.29E+00	6.29E+00
ENSG00000074696	PTPLAD1	0.85	-1.18	-0.24	3.53E-02	-6.29E+00	6.29E+00
ENSG00000149929	HIRIP3	0.77	-1.29	-0.37	4.24E-02	-6.28E+00	6.28E+00
ENSG00000110719	TCIRG1	0.85	-1.17	-0.23	3.48E-02	-6.28E+00	6.28E+00
ENSG00000137947	GTF2B	1.17	1.17	0.23	3.49E-02	6.27E+00	6.27E+00
ENSG00000162244	RPL29	0.84	-1.19	-0.25	3.60E-02	-6.27E+00	6.27E+00
ENSG00000177565	TBL1XR1	1.19	1.19	0.25	3.59E-02	6.26E+00	6.26E+00
ENSG00000114739	ACVR2B	1.28	1.28	0.35	4.15E-02	6.26E+00	6.26E+00
ENSG00000177570	SAMD12	0.82	-1.22	-0.29	3.82E-02	-6.26E+00	6.26E+00
ENSG00000156304	SCAF4	1.19	1.19	0.25	3.61E-02	6.25E+00	6.25E+00
ENSG00000134419	RPS15A	0.83	-1.20	-0.26	3.69E-02	-6.24E+00	6.24E+00
ENSG00000136240	KDELR2	0.83	-1.20	-0.26	3.70E-02	-6.24E+00	6.24E+00
ENSG00000114544	SLC41A3	1.18	1.18	0.23	3.55E-02	6.24E+00	6.24E+00
ENSG00000141933	TPGS1	1.20	1.20	0.26	3.70E-02	6.24E+00	6.24E+00
ENSG00000227199	ST7-AS1	1.36	1.36	0.45	4.79E-02	6.24E+00	6.24E+00
ENSG00000186300	ZNF555	1.19	1.19	0.25	3.64E-02	6.23E+00	6.23E+00
ENSG00000075856	SART3	0.84	-1.18	-0.24	3.62E-02	-6.23E+00	6.23E+00
ENSG00000131115	ZNF227	1.19	1.19	0.25	3.66E-02	6.22E+00	6.22E+00
ENSG00000164169	PRMT10	1.18	1.18	0.24	3.58E-02	6.22E+00	6.22E+00
ENSG00000178163	ZNF518B	1.19	1.19	0.25	3.68E-02	6.22E+00	6.22E+00
ENSG00000221838	AP4M1	0.85	-1.17	-0.23	3.55E-02	-6.22E+00	6.22E+00
ENSG00000110583	NAA40	0.85	-1.17	-0.23	3.55E-02	-6.22E+00	6.22E+00
ENSG00000116001	TIA1	1.21	1.21	0.27	3.76E-02	6.22E+00	6.22E+00
ENSG00000184640	SEPT9	1.21	1.21	0.27	3.77E-02	6.22E+00	6.22E+00
ENSG00000138785	INTS12	1.19	1.19	0.25	3.64E-02	6.21E+00	6.21E+00
ENSG00000086504	MRPL28	0.85	-1.17	-0.23	3.55E-02	-6.21E+00	6.21E+00
ENSG00000136271	DDX56	0.85	-1.17	-0.23	3.58E-02	-6.21E+00	6.21E+00

ENSG00000147509	RGS20	1.33	1.33	0.42	4.61E-02	6.21E+00	6.21E+00
ENSG00000110931	CAMKK2	0.85	-1.18	-0.24	3.62E-02	-6.20E+00	6.20E+00
ENSG00000181704	YIPF6	0.85	-1.18	-0.24	3.64E-02	-6.19E+00	6.19E+00
ENSG00000006451	RALA	1.25	1.25	0.32	4.05E-02	6.19E+00	6.19E+00
ENSG00000065154	OAT	1.18	1.18	0.24	3.66E-02	6.18E+00	6.18E+00
ENSG00000257591	ZNF625	0.80	-1.25	-0.32	4.08E-02	-6.18E+00	6.18E+00
ENSG00000245060	CTC-205M6.2	0.80	-1.25	-0.33	4.12E-02	-6.18E+00	6.18E+00
ENSG00000177485	ZBTB33	1.24	1.24	0.31	4.03E-02	6.18E+00	6.18E+00
ENSG00000179476	C14orf28	1.27	1.27	0.35	4.25E-02	6.18E+00	6.18E+00
ENSG00000075945	KIFAP3	1.17	1.17	0.22	3.57E-02	6.17E+00	6.17E+00
ENSG00000090238	YPEL3	1.20	1.20	0.26	3.77E-02	6.17E+00	6.17E+00
ENSG00000142230	SAE1	0.85	-1.18	-0.24	3.65E-02	-6.17E+00	6.17E+00
ENSG00000181690	PLAG1	1.24	1.24	0.31	4.03E-02	6.16E+00	6.16E+00
ENSG00000101363	MANBAL	0.81	-1.24	-0.31	4.07E-02	-6.15E+00	6.15E+00
ENSG00000197150	ABCB8	0.84	-1.19	-0.25	3.77E-02	-6.15E+00	6.15E+00
ENSG00000151093	OXSM	1.19	1.19	0.25	3.74E-02	6.14E+00	6.14E+00
ENSG00000140280	LYSMD2	0.83	-1.21	-0.27	3.85E-02	-6.14E+00	6.14E+00
ENSG00000170962	PDGFD	1.22	1.22	0.29	3.95E-02	6.14E+00	6.14E+00
ENSG00000158201	ABHD3	0.83	-1.21	-0.27	3.86E-02	-6.14E+00	6.14E+00
ENSG00000137218	FRS3	1.32	1.32	0.40	4.65E-02	6.14E+00	6.14E+00
ENSG00000204560	DHX16	0.84	-1.18	-0.24	3.73E-02	-6.13E+00	6.13E+00
ENSG00000145780	FEM1C	1.17	1.17	0.23	3.65E-02	6.12E+00	6.12E+00
ENSG00000135829	DHX9	1.18	1.18	0.24	3.72E-02	6.11E+00	6.11E+00
ENSG00000272742	PAIP2	0.85	-1.17	-0.23	3.69E-02	-6.10E+00	6.10E+00
ENSG00000235162	C12orf75	0.85	-1.17	-0.23	3.68E-02	-6.10E+00	6.10E+00
ENSG00000174842	GLMN	0.85	-1.17	-0.23	3.70E-02	-6.10E+00	6.10E+00
ENSG00000173588	CCDC41	0.84	-1.20	-0.26	3.85E-02	-6.09E+00	6.09E+00
ENSG00000117174	ZNHIT6	0.84	-1.19	-0.24	3.80E-02	-6.08E+00	6.08E+00
ENSG00000169683	LRRC45	0.85	-1.17	-0.23	3.74E-02	-6.07E+00	6.07E+00
ENSG00000067704	IARS2	1.18	1.18	0.24	3.77E-02	6.06E+00	6.06E+00
ENSG00000182400	TRAPPC6B	1.17	1.17	0.23	3.73E-02	6.06E+00	6.06E+00
ENSG00000166889	PATL1	1.17	1.17	0.23	3.75E-02	6.05E+00	6.05E+00
ENSG00000009844	VTA1	1.18	1.18	0.24	3.79E-02	6.05E+00	6.05E+00
ENSG00000118655	DCLRE1B	0.85	-1.17	-0.23	3.76E-02	-6.04E+00	6.04E+00
ENSG00000184898	RBM43	0.84	-1.19	-0.25	3.88E-02	-6.04E+00	6.04E+00
ENSG00000165480	SKA3	0.85	-1.18	-0.24	3.81E-02	-6.04E+00	6.04E+00
ENSG00000103035	PSMD7	0.85	-1.18	-0.24	3.82E-02	-6.03E+00	6.03E+00
ENSG00000112365	ZBTB24	0.85	-1.18	-0.24	3.83E-02	-6.03E+00	6.03E+00
ENSG00000111850	C6orf162	0.82	-1.21	-0.28	4.06E-02	-6.03E+00	6.03E+00
ENSG00000120896	SORBS3	1.19	1.19	0.25	3.89E-02	6.01E+00	6.01E+00
ENSG00000108960	MMD	0.84	-1.19	-0.25	3.92E-02	-6.00E+00	6.00E+00
ENSG00000197586	ENTPD6	0.86	-1.17	-0.22	3.79E-02	-6.00E+00	6.00E+00
ENSG00000111328	CDK2AP1	1.17	1.17	0.23	3.82E-02	6.00E+00	6.00E+00
ENSG00000154027	AK5	0.85	-1.18	-0.24	3.87E-02	-6.00E+00	6.00E+00
ENSG00000165288	BRWD3	1.23	1.23	0.30	4.21E-02	6.00E+00	6.00E+00
ENSG00000165997	ARL5B	1.26	1.26	0.34	4.44E-02	5.99E+00	5.99E+00
ENSG00000139505	MTMR6	1.19	1.19	0.25	3.93E-02	5.99E+00	5.99E+00
ENSG00000099904	ZDHHC8	1.20	1.20	0.26	4.02E-02	5.99E+00	5.99E+00
ENSG00000092820	EZR	1.18	1.18	0.24	3.88E-02	5.98E+00	5.98E+00
ENSG00000166669	ATF7IP2	1.20	1.20	0.27	4.05E-02	5.98E+00	5.98E+00
ENSG00000102931	ARL2BP	0.84	-1.18	-0.24	3.93E-02	-5.98E+00	5.98E+00
ENSG00000074054	CLASP1	1.18	1.18	0.23	3.88E-02	5.98E+00	5.98E+00
ENSG00000056050	C4orf27	0.85	-1.18	-0.24	3.89E-02	-5.97E+00	5.97E+00
ENSG00000151779	NBAS	1.17	1.17	0.23	3.86E-02	5.97E+00	5.97E+00
ENSG00000090924	PLEKHG2	1.18	1.18	0.24	3.92E-02	5.97E+00	5.97E+00
ENSG00000084764	MAPRE3	1.33	1.33	0.42	5.00E-02	5.97E+00	5.97E+00
ENSG00000124788	ATXN1	1.18	1.18	0.24	3.93E-02	5.97E+00	5.97E+00
ENSG00000149313	AASDHPPT	1.19	1.19	0.25	3.99E-02	5.96E+00	5.96E+00
ENSG00000215883	CYB5RL	0.82	-1.22	-0.29	4.23E-02	-5.95E+00	5.95E+00
ENSG00000106069	CHN2	0.81	-1.24	-0.31	4.33E-02	-5.95E+00	5.95E+00
ENSG00000138777	PPA2	0.85	-1.18	-0.24	3.92E-02	-5.95E+00	5.95E+00
ENSG00000145868	FBXO38	1.17	1.17	0.23	3.88E-02	5.95E+00	5.95E+00
ENSG00000136643	RPS6KC1	1.17	1.17	0.22	3.86E-02	5.94E+00	5.94E+00
ENSG00000245532	NEAT1	1.18	1.18	0.24	3.93E-02	5.94E+00	5.94E+00
ENSG00000280102	RN7SL2	0.77	-1.30	-0.38	4.79E-02	-5.93E+00	5.93E+00
ENSG00000197217	ENTPD4	1.17	1.17	0.23	3.90E-02	5.92E+00	5.92E+00
ENSG00000214717	ZBED1	1.17	1.17	0.23	3.93E-02	5.92E+00	5.92E+00
ENSG00000140497	SCAMP2	1.17	1.17	0.23	3.92E-02	5.91E+00	5.91E+00
ENSG00000089009	RPL6	0.85	-1.18	-0.24	3.99E-02	-5.91E+00	5.91E+00
ENSG00000141756	FKBP10	0.85	-1.18	-0.24	4.01E-02	-5.90E+00	5.90E+00
ENSG00000137817	PARP6	0.85	-1.17	-0.23	3.95E-02	-5.90E+00	5.90E+00
ENSG00000136518	ACTL6A	0.85	-1.17	-0.23	3.94E-02	-5.90E+00	5.90E+00
ENSG00000189164	ZNF527	0.82	-1.22	-0.29	4.32E-02	-5.89E+00	5.89E+00
ENSG00000163946	FAM208A	1.23	1.23	0.30	4.38E-02	5.88E+00	5.88E+00
ENSG00000169020	ATP5I	0.82	-1.22	-0.29	4.34E-02	-5.88E+00	5.88E+00
ENSG00000104177	MYEF2	0.83	-1.21	-0.27	4.22E-02	-5.87E+00	5.87E+00
ENSG00000197756	RPL37A	0.82	-1.22	-0.28	4.30E-02	-5.87E+00	5.87E+00
ENSG00000159023	EPB41	1.17	1.17	0.23	4.01E-02	5.87E+00	5.87E+00
ENSG00000166734	CASC4	1.18	1.18	0.23	4.02E-02	5.87E+00	5.87E+00

ENSG00000160408	ST6GALNAC6	1.17	1.17	0.23	3.98E-02	5.86E+00	5.86E+00
ENSG00000160932	LY6E	0.85	-1.17	-0.23	4.00E-02	-5.86E+00	5.86E+00
ENSG00000183530	PRR14L	1.18	1.18	0.23	4.03E-02	5.86E+00	5.86E+00
ENSG00000175866	BAIAP2	0.79	-1.26	-0.34	4.66E-02	-5.86E+00	5.86E+00
ENSG00000060491	OGFR	1.16	1.16	0.22	3.94E-02	5.85E+00	5.85E+00
ENSG00000113569	NUP155	0.85	-1.18	-0.23	4.04E-02	-5.85E+00	5.85E+00
ENSG00000159322	ADPGK	0.86	-1.17	-0.22	3.99E-02	-5.84E+00	5.84E+00
ENSG00000248124	RRN3P1	0.83	-1.20	-0.26	4.21E-02	-5.84E+00	5.84E+00
ENSG00000104517	UBR5	1.18	1.18	0.23	4.06E-02	5.83E+00	5.83E+00
ENSG00000133318	RTN3	0.85	-1.18	-0.23	4.06E-02	-5.83E+00	5.83E+00
ENSG00000075426	FOSL2	1.18	1.18	0.24	4.13E-02	5.83E+00	5.83E+00
ENSG00000136504	KAT7	0.85	-1.18	-0.24	4.08E-02	-5.83E+00	5.83E+00
ENSG00000107281	NPDC1	0.86	-1.16	-0.22	3.99E-02	-5.82E+00	5.82E+00
ENSG00000221990	C5orf55	1.29	1.29	0.37	4.91E-02	5.82E+00	5.82E+00
ENSG00000137200	FTSJD2	0.86	-1.17	-0.22	4.02E-02	-5.81E+00	5.81E+00
ENSG00000003147	ICA1	0.86	-1.16	-0.22	4.02E-02	-5.81E+00	5.81E+00
ENSG00000120509	PDZD11	0.86	-1.16	-0.22	4.01E-02	-5.81E+00	5.81E+00
ENSG00000136930	PSMB7	1.17	1.17	0.23	4.10E-02	5.80E+00	5.80E+00
ENSG00000173992	CCS	0.85	-1.18	-0.23	4.11E-02	-5.80E+00	5.80E+00
ENSG00000118894	FAM86A	0.84	-1.18	-0.24	4.17E-02	-5.80E+00	5.80E+00
ENSG00000182831	C16orf72	1.17	1.17	0.23	4.08E-02	5.80E+00	5.80E+00
ENSG00000111799	COL12A1	1.20	1.20	0.26	4.30E-02	5.79E+00	5.79E+00
ENSG00000158006	PAFAH2	0.84	-1.19	-0.25	4.22E-02	-5.79E+00	5.79E+00
ENSG00000083828	ZNF586	0.84	-1.18	-0.24	4.18E-02	-5.79E+00	5.79E+00
ENSG00000149792	MRPL49	0.86	-1.17	-0.23	4.08E-02	-5.79E+00	5.79E+00
ENSG00000186312	CA5BP1	1.22	1.22	0.28	4.43E-02	5.79E+00	5.79E+00
ENSG00000090615	GOLGA3	1.17	1.17	0.23	4.12E-02	5.78E+00	5.78E+00
ENSG00000124151	NCOA3	0.85	-1.17	-0.23	4.12E-02	-5.77E+00	5.77E+00
ENSG00000116191	RALGPS2	1.22	1.22	0.29	4.49E-02	5.77E+00	5.77E+00
ENSG00000164008	C1orf50	0.84	-1.19	-0.25	4.23E-02	-5.77E+00	5.77E+00
ENSG00000148019	CEP78	0.85	-1.18	-0.23	4.16E-02	-5.77E+00	5.77E+00
ENSG00000130803	ZNF317	1.18	1.18	0.24	4.17E-02	5.77E+00	5.77E+00
ENSG00000165506	DNAAF2	1.17	1.17	0.23	4.15E-02	5.76E+00	5.76E+00
ENSG00000177303	CASKIN2	1.21	1.21	0.27	4.38E-02	5.76E+00	5.76E+00
ENSG00000086015	MAST2	0.86	-1.17	-0.23	4.13E-02	-5.76E+00	5.76E+00
ENSG00000183597	C22orf25	1.16	1.16	0.22	4.10E-02	5.75E+00	5.75E+00
ENSG00000108424	KPNB1	0.84	-1.19	-0.25	4.26E-02	-5.74E+00	5.74E+00
ENSG00000163961	RNF168	0.85	-1.18	-0.24	4.27E-02	-5.73E+00	5.73E+00
ENSG00000110080	ST3GAL4	1.20	1.20	0.26	4.39E-02	5.73E+00	5.73E+00
ENSG00000114853	ZBTB47	1.17	1.17	0.23	4.19E-02	5.72E+00	5.72E+00
ENSG00000100629	CEP128	0.80	-1.24	-0.31	4.73E-02	-5.71E+00	5.71E+00
ENSG00000132424	PNISR	0.85	-1.18	-0.24	4.29E-02	-5.71E+00	5.71E+00
ENSG00000178096	BOLA1	0.83	-1.21	-0.27	4.47E-02	-5.71E+00	5.71E+00
ENSG00000075151	EIF4G3	0.85	-1.17	-0.23	4.23E-02	-5.71E+00	5.71E+00
ENSG00000158480	SPATA2	1.24	1.24	0.32	4.76E-02	5.70E+00	5.70E+00
ENSG00000105483	CARD8	0.85	-1.18	-0.23	4.25E-02	-5.70E+00	5.70E+00
ENSG00000130244	FAM98C	0.81	-1.23	-0.30	4.65E-02	-5.70E+00	5.70E+00
ENSG00000109133	TMEM33	0.85	-1.17	-0.23	4.24E-02	-5.69E+00	5.69E+00
ENSG00000205758	CRYZL1	0.83	-1.20	-0.27	4.49E-02	-5.69E+00	5.69E+00
ENSG00000120008	WDR11	0.85	-1.17	-0.23	4.25E-02	-5.68E+00	5.68E+00
ENSG00000173757	STAT5B	1.17	1.17	0.22	4.23E-02	5.67E+00	5.67E+00
ENSG00000137177	KIF13A	0.85	-1.17	-0.23	4.25E-02	-5.67E+00	5.67E+00
ENSG00000178896	EXOSC4	0.84	-1.19	-0.25	4.38E-02	-5.67E+00	5.67E+00
ENSG00000166783	KIAA0430	1.17	1.17	0.23	4.28E-02	5.67E+00	5.67E+00
ENSG00000230453	ANKRD18B	0.79	-1.27	-0.34	4.98E-02	-5.67E+00	5.67E+00
ENSG00000106052	TAX1BP1	1.22	1.22	0.28	4.62E-02	5.66E+00	5.66E+00
ENSG00000159335	PTMS	1.18	1.18	0.24	4.34E-02	5.66E+00	5.66E+00
ENSG00000278259	MYO19	0.85	-1.17	-0.23	4.28E-02	-5.66E+00	5.66E+00
ENSG00000068394	GPKOW	0.85	-1.17	-0.23	4.29E-02	-5.65E+00	5.65E+00
ENSG00000164430	MB21D1	0.84	-1.19	-0.25	4.47E-02	-5.64E+00	5.64E+00
ENSG00000047621	C12orf4	1.25	1.25	0.32	4.88E-02	5.64E+00	5.64E+00
ENSG00000146676	PURB	1.18	1.18	0.24	4.38E-02	5.64E+00	5.64E+00
ENSG00000180182	MED14	0.86	-1.17	-0.22	4.30E-02	-5.64E+00	5.64E+00
ENSG00000133816	MICAL2	1.18	1.18	0.23	4.36E-02	5.63E+00	5.63E+00
ENSG00000213190	MLLT11	0.85	-1.17	-0.23	4.35E-02	-5.63E+00	5.63E+00
ENSG00000164867	NOS3	1.24	1.24	0.31	4.87E-02	5.63E+00	5.63E+00
ENSG00000135185	C7orf23	0.85	-1.17	-0.23	4.34E-02	-5.63E+00	5.63E+00
ENSG00000129595	EPB41L4A	0.85	-1.17	-0.23	4.34E-02	-5.63E+00	5.63E+00
ENSG00000163807	KIAA1143	0.86	-1.17	-0.22	4.29E-02	-5.63E+00	5.63E+00
ENSG00000169213	RAB3B	1.18	1.18	0.23	4.36E-02	5.63E+00	5.63E+00
ENSG00000204387	C6orf48	0.85	-1.18	-0.23	4.37E-02	-5.63E+00	5.63E+00
ENSG00000089094	KDM2B	1.16	1.16	0.21	4.26E-02	5.62E+00	5.62E+00
ENSG00000175611	LINC00476	0.80	-1.25	-0.32	4.94E-02	-5.61E+00	5.61E+00
ENSG00000146834	MEPCE	0.86	-1.16	-0.22	4.31E-02	-5.60E+00	5.60E+00
ENSG00000056586	RC3H2	1.19	1.19	0.25	4.51E-02	5.60E+00	5.60E+00
ENSG00000105889	STEAP1B	0.86	-1.16	-0.22	4.31E-02	-5.60E+00	5.60E+00
ENSG00000141503	MINK1	1.17	1.17	0.23	4.39E-02	5.60E+00	5.60E+00
ENSG00000185340	GAS2L1	1.20	1.20	0.26	4.58E-02	5.59E+00	5.59E+00
ENSG00000183856	IQGAP3	0.84	-1.20	-0.26	4.59E-02	-5.59E+00	5.59E+00

ENSG00000196923	PDLIM7	1.16	1.16	0.22	4.32E-02	5.58E+00	5.58E+00
ENSG00000182362	YBEY	1.23	1.23	0.30	4.84E-02	5.58E+00	5.58E+00
ENSG00000174231	PRPF8	1.19	1.19	0.25	4.56E-02	5.58E+00	5.58E+00
ENSG00000149925	ALDOA	0.82	-1.21	-0.28	4.74E-02	-5.57E+00	5.57E+00
ENSG00000125703	ATG4C	0.85	-1.18	-0.23	4.46E-02	-5.57E+00	5.57E+00
ENSG00000132879	FBXO44	1.19	1.19	0.25	4.57E-02	5.57E+00	5.57E+00
ENSG00000104325	DECR1	0.86	-1.17	-0.22	4.40E-02	-5.57E+00	5.57E+00
ENSG00000169371	SNUPN	0.84	-1.19	-0.25	4.57E-02	-5.56E+00	5.56E+00
ENSG00000142544	CTU1	1.23	1.23	0.30	4.90E-02	5.56E+00	5.56E+00
ENSG00000146416	AIG1	1.18	1.18	0.23	4.47E-02	5.56E+00	5.56E+00
ENSG00000163162	RNF149	1.19	1.19	0.25	4.58E-02	5.56E+00	5.56E+00
ENSG00000126261	UBA2	0.85	-1.17	-0.23	4.44E-02	-5.55E+00	5.55E+00
ENSG00000130638	ATXN10	1.16	1.16	0.22	4.39E-02	5.55E+00	5.55E+00
ENSG00000205181	LINC00654	0.83	-1.20	-0.27	4.69E-02	-5.55E+00	5.55E+00
ENSG00000113648	H2AFY	1.17	1.17	0.23	4.45E-02	5.55E+00	5.55E+00
ENSG00000141068	KSR1	0.84	-1.20	-0.26	4.66E-02	-5.55E+00	5.55E+00
ENSG00000127054	CPSF3L	0.86	-1.16	-0.22	4.42E-02	-5.54E+00	5.54E+00
ENSG00000164743	C8orf48	1.21	1.21	0.28	4.78E-02	5.54E+00	5.54E+00
ENSG00000171574	ZNF584	0.84	-1.20	-0.26	4.67E-02	-5.54E+00	5.54E+00
ENSG00000110851	PRDM4	1.16	1.16	0.22	4.41E-02	5.54E+00	5.54E+00
ENSG00000137821	LRRC49	1.22	1.22	0.29	4.85E-02	5.54E+00	5.54E+00
ENSG00000108691	CCL2	1.19	1.19	0.25	4.62E-02	5.54E+00	5.54E+00
ENSG00000183337	BCOR	1.19	1.19	0.25	4.60E-02	5.53E+00	5.53E+00
ENSG00000165476	REEP3	0.85	-1.17	-0.23	4.50E-02	-5.52E+00	5.52E+00
ENSG00000169057	MECP2	1.17	1.17	0.23	4.50E-02	5.52E+00	5.52E+00
ENSG00000023608	SNAPC1	0.86	-1.16	-0.21	4.44E-02	-5.51E+00	5.51E+00
ENSG00000119541	VPS4B	1.17	1.17	0.23	4.53E-02	5.50E+00	5.50E+00
ENSG00000109680	TBC1D19	0.83	-1.20	-0.26	4.75E-02	-5.50E+00	5.50E+00
ENSG00000112695	COX7A2	0.84	-1.20	-0.26	4.73E-02	-5.50E+00	5.50E+00
ENSG00000157077	ZFYVE9	0.85	-1.17	-0.23	4.55E-02	-5.50E+00	5.50E+00
ENSG00000138439	FAM117B	1.20	1.20	0.27	4.80E-02	5.49E+00	5.49E+00
ENSG00000150938	CRIM1	0.84	-1.19	-0.26	4.73E-02	-5.49E+00	5.49E+00
ENSG00000101460	MAP1LC3A	0.81	-1.23	-0.30	4.99E-02	-5.49E+00	5.49E+00
ENSG00000171612	SLC25A33	0.84	-1.19	-0.25	4.73E-02	-5.48E+00	5.48E+00
ENSG00000105393	BABAM1	0.86	-1.17	-0.22	4.56E-02	-5.47E+00	5.47E+00
ENSG00000137714	FDX1	0.85	-1.17	-0.23	4.61E-02	-5.46E+00	5.46E+00
ENSG00000117054	ACADM	0.85	-1.17	-0.23	4.63E-02	-5.46E+00	5.46E+00
ENSG00000091164	TXNL1	0.85	-1.17	-0.23	4.62E-02	-5.46E+00	5.46E+00
ENSG00000075218	GTSE1	0.86	-1.16	-0.22	4.53E-02	-5.45E+00	5.45E+00
ENSG00000136238	RAC1	1.17	1.17	0.23	4.62E-02	5.45E+00	5.45E+00
ENSG00000197261	C6orf141	1.17	1.17	0.23	4.62E-02	5.45E+00	5.45E+00
ENSG00000157637	SLC38A10	1.21	1.21	0.28	4.95E-02	5.45E+00	5.45E+00
ENSG00000125347	IRF1	0.85	-1.17	-0.23	4.64E-02	-5.44E+00	5.44E+00
ENSG00000183520	UTP11L	0.86	-1.16	-0.22	4.58E-02	-5.44E+00	5.44E+00
ENSG00000171813	PWWP2B	1.21	1.21	0.27	4.92E-02	5.44E+00	5.44E+00
ENSG00000115241	PPM1G	0.85	-1.17	-0.23	4.65E-02	-5.43E+00	5.43E+00
ENSG00000113719	ERGIC1	1.17	1.17	0.23	4.69E-02	5.42E+00	5.42E+00
ENSG00000213203	GIMAP1	0.86	-1.16	-0.21	4.57E-02	-5.42E+00	5.42E+00
ENSG00000254093	PINX1	1.17	1.17	0.23	4.67E-02	5.42E+00	5.42E+00
ENSG00000124659	TBCC	1.16	1.16	0.22	4.60E-02	5.41E+00	5.41E+00
ENSG00000100138	NHP2L1	0.85	-1.17	-0.23	4.69E-02	-5.41E+00	5.41E+00
ENSG00000130818	ZNF426	1.16	1.16	0.22	4.60E-02	5.41E+00	5.41E+00
ENSG00000159685	CHCHD6	0.83	-1.21	-0.27	4.97E-02	-5.41E+00	5.41E+00
ENSG00000205155	PSENEN	0.84	-1.19	-0.25	4.86E-02	-5.41E+00	5.41E+00
ENSG00000130958	SLC35D2	1.16	1.16	0.21	4.59E-02	5.41E+00	5.41E+00
ENSG00000107186	MPDZ	1.20	1.20	0.26	4.91E-02	5.40E+00	5.40E+00
ENSG00000110917	MLEC	0.85	-1.18	-0.24	4.76E-02	-5.40E+00	5.40E+00
ENSG00000172375	C2CD2L	1.19	1.19	0.25	4.84E-02	5.40E+00	5.40E+00
ENSG00000104886	PLEKHJ1	0.86	-1.17	-0.22	4.68E-02	-5.40E+00	5.40E+00
ENSG00000163026	C2orf44	0.86	-1.16	-0.21	4.59E-02	-5.40E+00	5.40E+00
ENSG00000148498	PARD3	1.16	1.16	0.22	4.65E-02	5.39E+00	5.39E+00
ENSG00000100600	LGMN	0.86	-1.16	-0.21	4.64E-02	-5.38E+00	5.38E+00
ENSG00000175756	AURKAIP1	0.84	-1.19	-0.25	4.87E-02	-5.38E+00	5.38E+00
ENSG00000186908	ZDHHC17	0.84	-1.19	-0.25	4.88E-02	-5.38E+00	5.38E+00
ENSG00000150760	DOCK1	1.17	1.17	0.23	4.75E-02	5.37E+00	5.37E+00
ENSG00000197858	GPAA1	1.17	1.17	0.23	4.77E-02	5.37E+00	5.37E+00
ENSG00000115459	ELMOD3	1.17	1.17	0.23	4.74E-02	5.37E+00	5.37E+00
ENSG00000163389	POGLUT1	0.86	-1.17	-0.22	4.73E-02	-5.37E+00	5.37E+00
ENSG00000084070	SMAP2	0.85	-1.18	-0.24	4.81E-02	-5.37E+00	5.37E+00
ENSG00000143951	WDPCP	1.20	1.20	0.26	4.98E-02	5.36E+00	5.36E+00
ENSG00000131462	TUBG1	1.19	1.19	0.25	4.92E-02	5.36E+00	5.36E+00
ENSG00000144802	NFKBIZ	1.16	1.16	0.22	4.71E-02	5.36E+00	5.36E+00
ENSG00000196072	BLOC1S2	1.17	1.17	0.23	4.79E-02	5.35E+00	5.35E+00
ENSG00000133943	C14orf159	0.86	-1.16	-0.21	4.70E-02	-5.35E+00	5.35E+00
ENSG00000116266	STXBP3	0.85	-1.18	-0.24	4.87E-02	-5.33E+00	5.33E+00
ENSG00000168734	PKIG	1.18	1.18	0.23	4.87E-02	5.33E+00	5.33E+00
ENSG00000136870	ZNF189	1.16	1.16	0.21	4.71E-02	5.33E+00	5.33E+00
ENSG00000055332	EIF2AK2	0.85	-1.17	-0.23	4.87E-02	-5.32E+00	5.32E+00
ENSG00000187713	TMEM203	1.16	1.16	0.21	4.76E-02	5.31E+00	5.31E+00

ENSG00000162972	C2orf47	0.85	-1.17	-0.23	4.88E-02	-5.30E+00	5.30E+00
ENSG00000168438	CDC40	0.86	-1.16	-0.21	4.82E-02	-5.28E+00	5.28E+00
ENSG00000113732	ATP6V0E1	1.18	1.18	0.24	4.97E-02	5.28E+00	5.28E+00
ENSG00000181104	F2R	1.17	1.17	0.22	4.89E-02	5.28E+00	5.28E+00
ENSG00000240972	MIF	0.86	-1.17	-0.23	4.91E-02	-5.28E+00	5.28E+00
ENSG00000197562	RAB40C	0.85	-1.18	-0.23	4.97E-02	-5.27E+00	5.27E+00
ENSG00000093144	ECHDC1	1.17	1.17	0.23	4.95E-02	5.27E+00	5.27E+00
ENSG00000108669	CYTH1	1.17	1.17	0.23	4.95E-02	5.27E+00	5.27E+00
ENSG00000157869	RAB28	0.86	-1.16	-0.21	4.92E-02	-5.23E+00	5.23E+00
ENSG00000185753	CXorf38	0.86	-1.16	-0.22	4.96E-02	-5.22E+00	5.22E+00
ENSG00000197905	TEAD4	1.16	1.16	0.22	4.96E-02	5.21E+00	5.21E+00
ENSG00000120306	CYSTM1	1.16	1.16	0.21	4.98E-02	5.20E+00	5.20E+00
ENSG00000117691	NENF	1.16	1.16	0.22	4.99E-02	5.20E+00	5.20E+00
ENSG00000181191	PJA1	1.16	1.16	0.21	4.95E-02	5.19E+00	5.19E+00
ENSG00000141219	C17orf80	1.16	1.16	0.21	4.98E-02	5.19E+00	5.19E+00
ENSG00000105851	PIK3CG	1.16	1.16	0.21	4.99E-02	5.18E+00	5.18E+00
