# Supplementary material for: Aging-regulated PNUTS maintains endothelial barrier function via SEMA3B suppression
Source: Commun Biol. 2024 May 7;7:541. doi: 10.1038/s42003-024-06230-5 (PMC11076560; doi:10.1038/s42003-024-06230-5)
Supplement: Supplementary file 6 — Reporting Summary [file 42003_2024_6230_MOESM6_ESM.pdf]

nature portfolio

Corresponding author(s): Reinier Boon

Last updated by author(s): Apr 12, 2024

Reporting Summary

Nature Portfolio wishes to improve the reproducibility of the work that we publish. This form provides structure for consistency and transparency in reporting. For further information on Nature Portfolio policies, see our Editorial Policies and the Editorial Policy Checklist.

Statistics

For all statistical analyses, confirm that the following items are present in the figure legend, table legend, main text, or Methods section.

n/a	Confirmed	
☐	☒	The exact sample size (*n*) for each experimental group/condition, given as a discrete number and unit of measurement
☐	☒	A statement on whether measurements were taken from distinct samples or whether the same sample was measured repeatedly
☐	☒	The statistical test(s) used AND whether they are one- or two-sided<br>*Only common tests should be described solely by name; describe more complex techniques in the Methods section.*
☐	☒	A description of all covariates tested
☐	☒	A description of any assumptions or corrections, such as tests of normality and adjustment for multiple comparisons
☐	☒	A full description of the statistical parameters including central tendency (e.g. means) or other basic estimates (e.g. regression coefficient) AND variation (e.g. standard deviation) or associated estimates of uncertainty (e.g. confidence intervals)
☐	☒	For null hypothesis testing, the test statistic (e.g. *F*, *t*, *r*) with confidence intervals, effect sizes, degrees of freedom and *P* value noted<br>*Give P values as exact values whenever suitable.*
☒	☐	For Bayesian analysis, information on the choice of priors and Markov chain Monte Carlo settings
☒	☐	For hierarchical and complex designs, identification of the appropriate level for tests and full reporting of outcomes
☒	☐	Estimates of effect sizes (e.g. Cohen's *d*, Pearson's *r*), indicating how they were calculated

Our web collection on statistics for biologists contains articles on many of the points above.

Software and code

Policy information about availability of computer code

Data collection	*Provide a description of all commercial, open source and custom code used to collect the data in this study, specifying the version used OR state that no software was used.*
Data analysis	GraphPad Prism 7

For manuscripts utilizing custom algorithms or software that are central to the research but not yet described in published literature, software must be made available to editors and reviewers. We strongly encourage code deposition in a community repository (e.g. GitHub). See the Nature Portfolio guidelines for submitting code & software for further information.

Data

Policy information about availability of data

All manuscripts must include a data availability statement. This statement should provide the following information, where applicable:

- Accession codes, unique identifiers, or web links for publicly available datasets
- A description of any restrictions on data availability
- For clinical datasets or third party data, please ensure that the statement adheres to our policy

All data generated or analysed during this study are included in this published article (and its supplementary information files)

Research involving human participants, their data, or biological material

Policy information about studies with human participants or human data. See also policy information about sex, gender (identity/presentation), and sexual orientation and race, ethnicity and racism.

Reporting on sex and gender	N/A
Reporting on race, ethnicity, or other socially relevant groupings	N/A
Population characteristics	N/A
Recruitment	N/A
Ethics oversight	N/A

Note that full information on the approval of the study protocol must also be provided in the manuscript.

Field-specific reporting

Please select the one below that is the best fit for your research. If you are not sure, read the appropriate sections before making your selection.

☒ Life sciences ☐ Behavioural & social sciences ☐ Ecological, evolutionary & environmental sciences

For a reference copy of the document with all sections, see nature.com/documents/nr-reporting-summary-flat.pdf

Life sciences study design

All studies must disclose on these points even when the disclosure is negative.

Sample size	*Describe how sample size was determined, detailing any statistical methods used to predetermine sample size OR if no sample-size calculation was performed, describe how sample sizes were chosen and provide a rationale for why these sample sizes are sufficient.*
Data exclusions	*Describe any data exclusions. If no data were excluded from the analyses, state so OR if data were excluded, describe the exclusions and the rationale behind them, indicating whether exclusion criteria were pre-established.*
Replication	*Describe the measures taken to verify the reproducibility of the experimental findings. If all attempts at replication were successful, confirm this OR if there are any findings that were not replicated or cannot be reproduced, note this and describe why.*
Randomization	*Describe how samples/organisms/participants were allocated into experimental groups. If allocation was not random, describe how covariates were controlled OR if this is not relevant to your study, explain why.*
Blinding	*Describe whether the investigators were blinded to group allocation during data collection and/or analysis. If blinding was not possible, describe why OR explain why blinding was not relevant to your study.*

Reporting for specific materials, systems and methods

We require information from authors about some types of materials, experimental systems and methods used in many studies. Here, indicate whether each material, system or method listed is relevant to your study. If you are not sure if a list item applies to your research, read the appropriate section before selecting a response.

Materials & experimental systems

n/a	Involved in the study	
☐	☒	Antibodies
☐	☒	Eukaryotic cell lines
☒	☐	Palaeontology and archaeology
☐	☒	Animals and other organisms
☒	☐	Clinical data
☒	☐	Dual use research of concern
☒	☐	Plants

Methods

n/a	Involved in the study	
☒	☐	ChIP-seq
☐	☒	Flow cytometry
☒	☐	MRI-based neuroimaging

Antibodies

Antibodies used	anti-PNUTS (R&D Systems, AF21581), anti-CD31 (Invitrogen, 37-0700), anti-VE-Cadherin (Sigma-Aldrich, V1514), anti-MYC (CST, 9402), anti-phospho-MYC-T58 (abcam, ab185655), anti-tubulin (Thermo Fisher, RB-9281-P), anti-β-actin (Sigma-Aldrich, A-5441), anti-CD31 (BD Biosciences 550389), anti-VE-Cadherin (CST 2500XP), anti-CD31 antibody (Dianova, DIA-310)
Validation	anti-PNUTS (R&D Systems, AF21581): validated by manufacturer by WB of cell lysates; Boon et al, Nature, 2013; and experimental validation by WB (band disappears after siPNUTS treatment in Fig 1 and appears after PNUTS-overexpression experiments in Fig 4).<br>anti-CD31 (Invitrogen, 37-0700): validated by manufacturer by immunofluorescence and WB of HUVECs lysates.<br>anti-VE-Cadherin (Sigma-Aldrich, V1514): validated by manufacturer by WB of HUVECs lysates and immunohistochemistry in frozen sections of human tonsil<br>anti-MYC (CST, 9402): validated by manufacturer by Western blot analysis of extracts from HeLa cells 48 hours following mock transfection, transfection with nonspecific (control) siRNA or transfection with c-Myc siRNA<br>anti-phospho-MYC-T58 (abcam, ab185655): validated by manufacturer by WB analysis of HeLa cell lysate treated with 200nM Calyculin A and 1uM Okadaic Acid for 60 minutes.<br>anti-tubulin (Thermo Fisher, RB-9281-P): validated by manufacturer by WB analysis of human Cell-18 lysates<br>anti-β-actin (Sigma-Aldrich, A-5441): validated by manufacturer by WB using cultured human or chicken fibroblast cell extracts<br>anti-CD31 (BD Biosciences 550389): validated by manufacturer by immunohistochemistry of frozen sections of normal human thymus, then visualized with Biotin Goat Anti-Mouse Ig.<br>anti-VE-Cadherin (CST 2500XP): validated by manufacturer by immunofluorescent analysis of HUVECs and HeLa cells<br>anti-CD31 antibody (Dianova, DIA-310): validated by manufacturer by immunohistochemistry of formalin-fixed paraffin-embedded mouse tissue sections.

Eukaryotic cell lines

Policy information about cell lines and Sex and Gender in Research

Cell line source(s)	Primary human umbilical vein endothelial cells (HUVEC) were purchased from Lonza.
Authentication	Cells were tested after purchase for expression of endothelial markers by qPCR and immunofluorescence and for angiogenic sprouting capability
Mycoplasma contamination	Cells were tested for mycoplasma contamination every two weeks.
Commonly misidentified lines (See ICLAC register)	*Name any commonly misidentified cell lines used in the study and provide a rationale for their use.*

Animals and other research organisms

Policy information about studies involving animals; ARRIVE guidelines recommended for reporting animal research, and Sex and Gender in Research

Laboratory animals	Male c57Bl/6j mice, age 12-80 weeks, strain Cdh5-CreERT2xPNUTSfl/fl or PNUTSfl/fl
Wild animals	N/A
Reporting on sex	Male mice were used. No conclusions can be made regarding potential sex-specific phenotypes.
Field-collected samples	N/A
Ethics oversight	All mice experiments were carried out in accordance with the principles of laboratory animal care as well as according to the German and Dutch national laws. The studies have been approved by the local ethical committees and performed in accordance with the ethical standards laid down in the Declaration of Helsinki.

Note that full information on the approval of the study protocol must also be provided in the manuscript.

Flow Cytometry

Plots

Confirm that:

- [x] The axis labels state the marker and fluorochrome used (e.g. CD4-FITC).
- [x] The axis scales are clearly visible. Include numbers along axes only for bottom left plot of group (a 'group' is an analysis of identical markers).
- [ ] All plots are contour plots with outliers or pseudocolor plots.
- [x] A numerical value for number of cells or percentage (with statistics) is provided.

Methodology

Sample preparation	Transfected HUVECs were detached with Accutase and washed in cold incubation buffer (0.1% BSA in PBS). Cells were blocked (5% BSA in PBS) for 30 min on ice and subsequently incubated with fluorophore-labeled antibodies for 30 min on ice.
Instrument	FACSCalibur™ device (BD Biosciences)
Software	BD FACSDiva™ software and FlowJo
Cell population abundance	*Describe the abundance of the relevant cell populations within post-sort fractions, providing details on the purity of the samples and how it was determined.*
Gating strategy	*Describe the gating strategy used for all relevant experiments, specifying the preliminary FSC/SSC gates of the starting cell population, indicating where boundaries between "positive" and "negative" staining cell populations are defined.*

☒ Tick this box to confirm that a figure exemplifying the gating strategy is provided in the Supplementary Information.
